# The transcriptome-wide association search for genes and genetic variants which associate with BMI and gestational weight gain in women with type 1 diabetes

**DOI:** 10.1186/s10020-020-00266-z

**Published:** 2021-01-20

**Authors:** Agnieszka H. Ludwig-Słomczyńska, Michał T. Seweryn, Przemysław Kapusta, Ewelina Pitera, Urszula Mantaj, Katarzyna Cyganek, Paweł Gutaj, Łucja Dobrucka, Ewa Wender-Ożegowska, Maciej T. Małecki, Paweł P. Wołkow

**Affiliations:** 1 Center for Medical Genomics OMICRON, Jagiellonian University Medical College, Kraków, Poland; 2 Department of Cancer Biology and Genetics, The Ohio State University Wexner Medical Center, Columbus, OH USA; 3 Department of Reproduction, Poznan University of Medical Sciences, Poznan, Poland; 4 Department of Metabolic Diseases, University Hospital Kraków, Kraków, Poland; 5 Department of Metabolic Diseases, Jagiellonian University Medical College, Kraków, Poland

**Keywords:** Obesity, BMI, Gestational weight gain (GWG), Transcriptomic-wide association study (TWAS), Association analysis

## Abstract

**Background:**

Clinical data suggest that BMI and gestational weight gain (GWG) are strongly interconnected phenotypes; however, the genetic basis of the latter is rather unclear. Here we aim to find genes and genetic variants which influence BMI and/or GWG.

**Methods:**

We have genotyped 316 type 1 diabetics using Illumina Infinium Omni Express Exome-8 v1.4 arrays. The GIANT, ARIC and T2D-GENES summary statistics were used for TWAS (performed with PrediXcan) in adipose tissue. Next, the analysis of association of imputed expression with BMI in the general and diabetic cohorts (Analysis 1 and 2) or GWG (Analysis 3 and 4) was performed, followed by variant association analysis (1 Mb around identified loci) with the mentioned phenotypes.

**Results:**

In Analysis 1 we have found 175 BMI associated genes and 19 variants (p < 10^–4^) which influenced GWG, with the strongest association for rs11465293 in *CCL24* (p = 3.18E−06). Analysis 2, with diabetes included in the model, led to discovery of 1812 BMI associated loci and 207 variants (p < 10^–4^) influencing GWG, with the strongest association for rs9690213 in *PODXL* (p = 9.86E−07). In Analysis 3, among 648 GWG associated loci, 2091 variants were associated with BMI (FDR < 0.05). In Analysis 4, 7 variants in GWG associated loci influenced BMI in the ARIC cohort.

**Conclusions:**

Here, we have shown that loci influencing BMI might have an impact on GWG and GWG associated loci might influence BMI, both in the general and T1DM cohorts. The results suggest that both phenotypes are related to insulin signaling, glucose homeostasis, mitochondrial metabolism, ubiquitinoylation and inflammatory responses.

## Introduction

In the recent years, significant attention was paid to transcriptomic-wide association studies (TWAS), which enable the prediction of gene-level associations between the 'imputed expression based on genetic data' and complex phenotypes (Gamazon et al. 2015). This is possible, since it was shown that a proportion of GWAS risk variants co-localize with genetic variants that regulate gene expression (i.e. expression quantitative trait loci, eQTL) (Hormozdiari et al. 2016). The prediction of gene expression based on genotype removes noise created by environmental factors as well as potential reverse causation (when the trait affects gene expression), therefore the TWAS analysis not only increases the statistical power, but also enables focusing on regions for which finding a functional interpretation of the associations is often easier (Mancuso et al. 2017; Li et al. 2018).

Gestational weight gain (GWG) has been extensively studied over the last several years as it was suggested that its inadequacy may lead to adverse both maternal (preeclampsia, hypertension, obesity later in life, cesarean section) and neonatal (preterm birth, stillbirth, inadequate neonatal weight—small or large for gestational age, obesity later in life) outcomes (Kominiarek and Peaceman 2017; Voerman et al. 2019). Simultaneously, clinicians tried to optimize pregnancy care for women with type 1 diabetes whose pregnancy outcomes (including developmental abnormalities, spontaneous abortions, neonatal hyperglycemia, neonatal hyperinsulinemia, maternal retinopathy, maternal nephropathy, preeclampsia) are far worse than in women from the general population (Celia et al. 2016). For a long time it was believed that it stemmed from maternal hyperglycemia; however, even though their glycemic goals have been achieved (HbA1c < 6.0%), large for gestational age (LGA) or macrosomic neonates births are more frequent among these women than in the general population (Scifres et al. 2014; Bashir et al. 2019; Dori-Dayan et al. 2020). Thus, there must be other potential contributors to adverse maternal and fetal outcomes (Secher et al. 2014; Mastella et al. 2018; Rys et al. 2018). Among them are also those which affect the general population—maternal body lipids, pre-pregnancy BMI and GWG (McWhorter et al. 2018).

Even though GWG can have an impact on the health of future generations, to date, most of the performed studies are retrospective or observational and their main goal was to assess the relationship between GWG and environmental factors (Nunnery et al. 2018; Siega-Riz et al. 2020), while the genetic risks for inadequate GWG have been scarcely analyzed. In a recent paper, the authors show that 43% and 26% of the variation in GWG can be explained by genetic factors in the first pregnancy and second pregnancy, respectively (Andersson et al. 2015). The first GWAS study on GWG in the general, multiethnic cohort was performed in 2018. The study has shown that 20% of the variability in GWG can be explained by maternal genetic variants. Unfortunately, it did not find significant associations of GWG with any genomic loci (Warrington et al. 2018).

Since both environmental and genetic data show that there is a correlation between pre-pregnancy BMI and GWG (Luecke et al. 2019) we took the TWAS approach to study the genetic correlation between BMI and GWG—in particular, to find loci and variants which are associated with GWG and BMI, or (on the contrary) only with GWG. We find this particularly important, as the reports on the overlap of genes and/or variants which influence both phenotypes appear to be conflicting (Lawlor et al. 2011; Kawai et al. 2019).

**Fig. 1:**
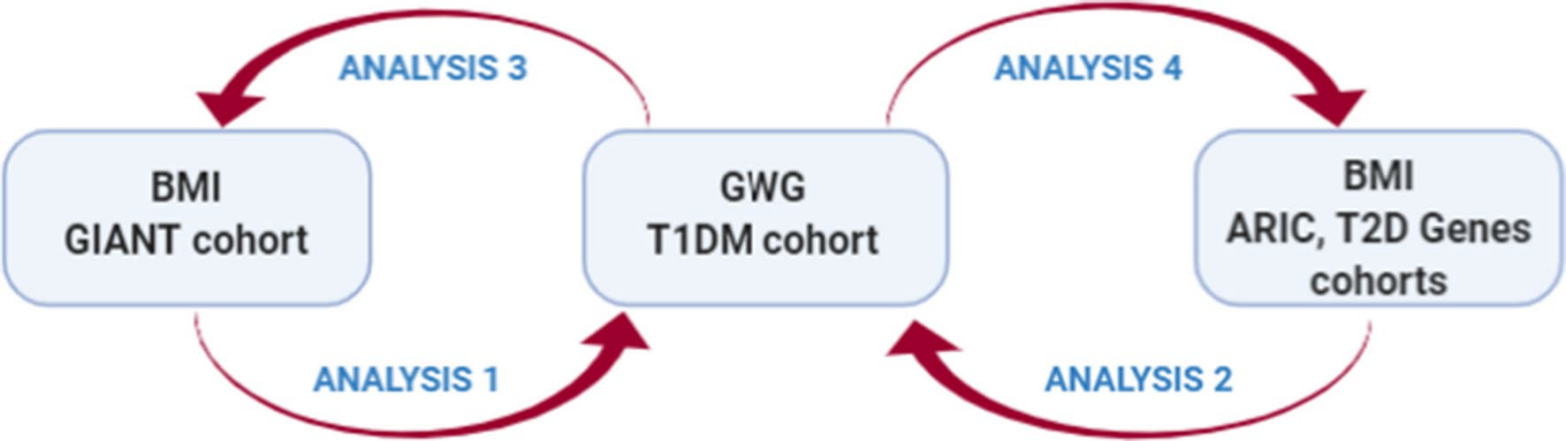
The scheme of the workflow. TWAS analyses to find genes associated with BMI in the general cohort (GIANT) and two diabetic cohorts (T2D-GENES, ARIC) as well as with GWG in the T1DM cohort, followed by variant associations between the two phenotypes

In this study, we aimed to investigate the genetic factors associated with GWG from the perspective of the genetics of obesity. To this aim, we performed TWAS analyses on the GIANT cohort representing the general population and two diabetic cohorts—T2D-GENES and ARIC—to find genes associated with BMI. The ARIC and T2D-GENES cohorts were used since diabetes mellitus itself might influence BMI and creates a special metabolic context, while no cohort with patients with type 1 diabetes was available. Only regions which were associated with BMI in these cohorts were subjected to the analysis of association with GWG in T1DM patients. Secondly, we also searched for reverse associations and checked whether genes in TWAS associated with GWG in the T1DM cohort might affect BMI in the ARIC or GIANT cohorts. If so, we decided to search for genes which imputed expression correlated with BMI in order to restrict the analysis of genetic association with GWG to these genes only, and conversely to check whether the genes which correlate with GWG (at the imputed expression level) do influence BMI. Thus, this work comprises 4 different analyses, as presented in Fig. 1.

## Methods

### Patients

Patients were recruited either in the Department of Metabolic Diseases University Hospital in Krakow or in the Division of Reproduction Department of Obstetrics, Gynecology and Gynecological Oncology, Poznan University of Medical Sciences, two important academic referral centers. Both cohorts included all consecutive pregnant women with pre-existing T1DM. The Kraków cohort included women who were referred to the clinic between the years 1998 and 2018, while the Poznań cohort of women who attended it between years 2006 and 2019. The data were collected at the time of clinic attendance. The women were either pregnant (no later than 1st trimester in Krakow, no later than 3rd trimester in Poznań) or planned pregnancy. Their clinical diagnosis of T1DM was established at least one year prior to conception. All women with diabetes who entered the pregnancy planning program received intensive diabetes management in the clinic. Forty percent of women in both cohorts planned their pregnancy. Women who did not plan their pregnancies entered the intensive diabetes care program after conception, at the first clinic visit. Pre-pregnancy care program includes education in the field of diet, self-monitoring of blood-glucose, glycemic targets, and self-adjustment of insulin doses, supplementation of folic acid and modification of therapy to improve glycemic control. The clinical characteristics in these women reflect the effects of routine diabetes management in non-pregnant patients. Two insulin regimens were used before pregnancy: multiple daily injections (MDI) or continuous subcutaneous insulin infusion (CSII) with a personal insulin pump. These patients were followed through the pregnancy. All women were registered in the clinic and medical data was created. Clinical characteristics of the women, information on the course of pregnancy and glycemic control markers were collected during regular clinic visits. Their pre-pregnancy weight was measured on the visit before pregnancy during preparation to conception. Women with unplanned pregnancy self-reported pre-pregnancy weight on first pregnancy visit in the clinic. The gestational weight gain was calculated as a difference between the weight collected on the last visit before delivery and pre-pregnancy weight. The partial clinical characteristics of these cohorts was published before (Cyganek et al. 2017; Gutaj et al. 2017). The prevalence of retinopathy was 25.6%. None of the patients showed evidence of chronic kidney disease of stage 3 or higher. Among the patients from the Poznań cohort the prevalence of retinopathy was 18%, nephropathy 13%. 16.8% of patients were hypertensive. The exclusion criteria were as follows: miscarriage, stillbirth, lack of data caused by not taking part in intensive diabetes management care or lack of regular out-patient visits in the clinic. We included only singleton pregnancies in the study. Whole blood samples were drawn and stored at − 80 °C. This study was approved by the Bioethical Committees of Jagiellonian University and Poznan University of Medical Sciences and performed according to the Helsinki Declaration. Written informed consent was collected from all patients.

### Genotyping

DNA was extracted from whole blood with the use of automated nucleic acid extraction system Maxwell (Promega). Five hundred twenty-seven samples were genotyped on Illumina Infinium Omni Express Exome-8 v1.4 arrays. Only in-term live births with information regarding age, pre-pregnancy BMI, GWG, diabetes duration, treatment method and daily insulin dose available were included in the final analysis.

### Data processing and imputation

The detailed protocol of the data processing, QC analysis and imputation is presented in (Ludwig-Słomczyńska et al. 2020).

### Genotype data and the analysis of genetic variants

The GWAS analysis on the T1DM cohort was performed on a group of 316 females with complete data on: age, parity, insulin dose prior pregnancy, pre-pregnancy BMI and GWG. The mixed-effects model approach was used as implemented in the package GENESIS in R. The random effect was associated with the individual ID and the genetic relatedness matrix was estimated via the PCARelate method in package GENESIS in R. The 'null' mixed effects model was considered as:

$$GWG\sim age + parity + pre - pregnancy\_BMI + pre - pregnancy\_InsDose + OriginOfSample + \left( {1|ID} \right),$$

where the OriginOfSample is an indicator of the sample being collected in the Division of Reproduction Department of Obstetrics, Gynecology and Gynecological Oncology, Poznan University of Medical Sciences. To the aim of testing significance of the (additive) effect of the genotype, the Wald's test statistics was used.

The summary statistics for BMI tested in the GIANT Consortium cohort were downloaded from https://portals.broadinstitute.org/collaboration/giant/images/1/15/SNP_gwas_mc_merge_nogc.tbl.uniq.gz.

The genotype and phenotype data for the ARIC cohort (GENEVA study) were accessed via dbGaP. As far as the genotype data is concerned, the imputed data for participants of European ancestry were used as deposited under the phg000248.v1 code. Per-chromosome genotype probability data were transformed to dosages. From these dosages, genotype relatedness matrices were estimated on a per-chromosome basis and merged with the aid of the SNPRelate package in R. For the GWAS analysis the GENESIS package was used. As far as the phenotype files are concerned, these were accessed under the phs000090.v3 study code. The 'null' mixed effects model was defined as:

$$BMI\sim age + sex + diabetes\_status + \left( {1|ID} \right)$$

As GENEVA is a longitudinal study, for diabetic participants, we used the BMI and age measurement at the first time when the status of the participant was 'diabetic', whereas for the non-diabetic participants we used the measurements at the study entry.

The T2Dgenes genotype files were accessed via dbGaP under the phg000573.v1 code. The raw vcf files were transformed to dosages prior analysis. The phenotype data were accessed from the phs000462.v2 study code. Similarly to the data analysis in the ARIC cohort, we recorded BMI and age at the first time when the diabetic status was positive or at the study entry, otherwise.

The TWAS analysis was performed via the PrediXcan and MetaXcan software with Adipose as the tissue of interest. For the GIANT Cohort data, the MetaXcan framework was implemented based on the GTEx.v7 models. For the ARIC, T2Dgenes and T1DM cohorts the PrediXcan software was used also with GTEx.v7 models. In the TWAS analyses in the ARIC and T1DM cohorts the same independent variables were used as with the GWAS models defined above—with the exception that the classical linear models were used as implemented in the limma package (with no random effects). For the T2Dgenes TWAS analysis, from the 'predicted expression' matrix the principal components were first estimated and the first component was added to the models as it did not correlate with any independent variable—and it differed between families (as tested with the Kruskal–Wallis test). As before, the target analysis in the T2Dgenes cohort was performed with the aid of linear models as implemented in the limma package.

The FUMA analysis was performed through the webserver at fuma.ctglab.nl.

The COJO and fast-BAT software were used as implemented by in the GTCA framework. The LD structure was estimated via the 1000 genomes data.

GO enrichment analysis was performed as implemented in the topGO package in R.

The flow of the subjects through the study as well as the accession numbers for the primary datasets used by us is present in Additional file 1: Data S1.

## Results

### Gestational weight gain analysis in T1DM cohort

Our analysis comprised 316 women with T1DM for whom full phenotype data were available. The basic characterization of patients included in the analysis is presented in Table 1.

**Table 1:** The clinical characteristics of the T1DM cohort

	Med; IQR
Age [years]	29; (25.75, 32)
Pre-pregnancy BMI [kg/m^2^]	23.41; (21.09, 26.2)
GWG [kg]	14; (10.5, 18)
Diabetes duration [years]	12; (6, 18)
Treatment method—CSII [%]	0.55
Daily insulin dose before pregnancy [U]	38; (28, 50)
Parity	1; (1, 2)

PrediXcan was used to impute gene expression in subcutaneous and visceral adipose tissue based on imputed genotype data, and the gene-based search for associations with GWG and BMI was performed using a linear model approach. Due to small cohort size, genes nominally significantly associated with GWG (442 genes in subcutaneous and 328 in visceral adipose tissue) and BMI (416 genes in subcutaneous and 326 in visceral adipose tissue) were considered for further analysis. It is worth noting that only sixty genes overlapped between the two phenotypes (Additional file 2: Table S1). We further explored this phenomenon by looking at the strength of the association between gene expression for these two traits. We found a negative correlation between logFCs of genes associated with GWG and BMI. This is consistent with clinical recommendations as patients with high pregestational BMI are advised to restrict their GWG; however, we believe that the genetic background might also play a role.

### PrediXcan analysis on BMI in the general population (GIANT cohort) and variant associations with GWG in T1DM cohort—Analysis 1

**Fig. 2:**
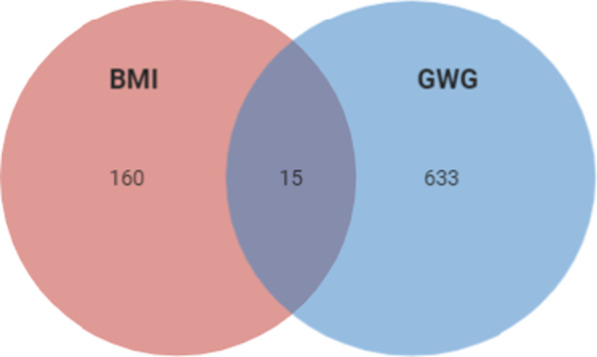
Venn diagram of the association of imputed gene expression which influence BMI in the Giant cohort, GWG in T1DM cohort, and both phenotypes

Gene expression prediction in visceral and subcutaneous adipose tissue in the GIANT cohort (234,069 patients (Vinet and Zhedanov 2011)) was performed using PrediXcan software. One hundred seventy-five genes significantly associated (p < 1E−04) with BMI in the adipose (subcutaneous and visceral combined) tissue were found (Additional file 3: Table S2). The Venn diagram of genes which affected BMI, GWG or both phenotypes is presented in Additional file 4: Table S3. Only 15 genes influenced both phenotypes and 160 influenced only BMI while 633 had an impact on GWG only (Fig. 2).

We further analyzed the loci of interest and searched for variants (within the 1 Mb window around the gene) which influence BMI and/or GWG. We first mapped variants in 175 BMI associated genes (± 500,000 bp) and applied these restricted variant panels to the analysis of the association with GWG in the T1DM cohort. We filtered the results by MAF > 5% and p-value < 1E−04 and found an association with GWG for 19 variants in the T1DM cohort (Table 2). We also performed the analysis of the association with GWG only for 15 genes which showed an overlap in TWAS. These data are presented in Additional file 5: Data S2 (marked as subAnalysis 1).

**Table 2:** The list of variants localized to genes associated with BMI in the GIANT cohort which associate with GWG

SNP ID	chr	MAF [%]	B	p-value	Gene/nearest gene	Localization/type
rs11465293	7	5.7	4.46	3.18E−06	*CCL24*	Missense
rs1978202	7	8.7	3.51	4.53E−06	*CCL26*	Intergenic
rs9347707	6	35.1	1.94	4.74E−05	*PACRG*	Intron
rs72849841	17	17.1	− 2.19	1.25E−04	*RNF213*	Missense
rs11807240	1	10.4	2.58	1.61E−04	*FUBP1*	Intron
rs9659938	1	10.4	2.58	1.61E−04	*NEXN204*	Intron
rs13340504	7	14.4	2.32	2.29E−04	*CCL24*	Intergenic
rs1541725	2	46.0	1.61	3.95E−04	*AC009502.4*	Intergenic
rs2136682	1	9.3	2.54	4.25E−04	*GIPC2*	Intron
rs4329088	6	46.2	1.63	4.33E−04	*PACRG*	Intron
rs886131	12	41.0	− 1.60	4.78E−04	*RPL29P25*	Noncoding transcript
rs11037234	11	33.1	1.66	5.34E−04	*RP11-111A24.2*	Intergenic
rs8122282	20	45.7	− 1.49	6.44E−04	*C20orf166*	Intron
rs4808209	17	6.9	3.00	7.13E−04	*ZNF101*	Start loss
rs9352745	6	26.1	1.70	7.27E−04	*LCA5*	Intron
rs876373	12	43.4	1.50	7.29E−04	*RPL29P25*	Intron
rs6062267	20	45.3	− 1.49	7.94E−04	*C20orf166*	Intron
rs7961894	12	8.5	2.75	8.88E−04	*WDR66*	Intron
rs7564856	2	34.5	− 1.59	9.62E−04	*SPEG*	Intergenic

The FUMA GWAS method further prioritized the variants obtained in the analysis. Twelve leading SNPs were detected. Three of them (rs7564856, rs11465293 and rs7961894) were associated with several traits in the GWAS Catalog, mainly blood parameters, but none significantly with metabolic outcome. At the same time, however, these were also associated at the level of significance p-value < 1E−03 with LDL cholesterol levels, nonalcoholic fatty liver disease, body mass index, mean arterial pressure or metabolite levels. These variants were eQTLs for 40 genes in several tissues, while rs9659938, rs11807240, rs2136682 (*NEXN*, *NEXN-AS1*), rs7564856 (*SPEG*), rs9352745 (*SH3BGRL2*), rs13340504 (*CCL24*), rs11465293 (*CCL24*), rs886131 (*RPL29P25*) influenced gene expression in both subcutaneous and visceral adipose. Thus, we conclude that there is a subset of genes which influence both BMI and GWG as well as genetic variants associated with GWG which co-localize to these loci.

Next, we tried to determine whether the signals for the two traits in the genes of interest are correlated. Thus, we checked whether the genetic variants associated with BMI and GWG are in linkage disequilibrium or they belong to different haplotypes. We performed the analysis of LD for all genes with variants significantly associated with GWG. However, below we present only the most interesting examples. In the gene *GPN3*, we have found 2 bins of eQTLs which are not in LD either with BMI or GWG variants. Variants associated with BMI also create a cluster, which is separate from a small cluster of 2 variants associated with GWG. One of GWG associated variants (rs876373) is in moderate LD with the BMI cluster (Additional file 6: Figure S1a). In another gene, *PMS2P3*, we found a cluster of 3 variants which are associated with GWG, all in LD with one eQTL (rs707395). At the same time, variants associated with BMI form a separate bin which is in moderate LD with a subset of eQTLs for this gene. This cluster is not in LD with the GWG bin or rs707395 (Additional file 6: Figure S1b). The third example is the *STAG3L1* gene in which three clusters—GWG associated variants, BMI associated variants and eQTLs can be seen (r^2^), however, in each, few members of any cluster are in LD with members of other clusters (D’) making them rather dependent on each other (Additional file 6: Figure S1c).

### PrediXcan analysis on BMI in the diabetic cohorts (ARIC and T2D-GENES) and variant associations with GWG in T1DM cohort—Analysis 2

**Fig. 3:**
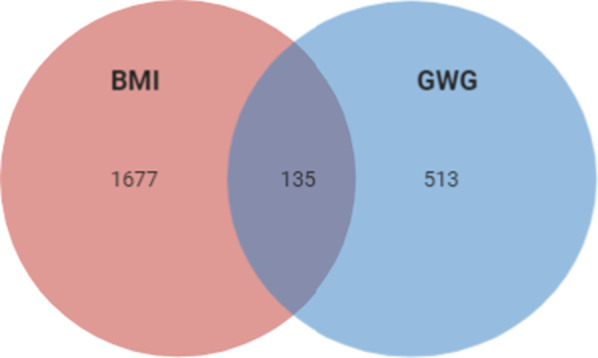
Overlap of the correlation of imputed expression of genes which influence BMI in ARIC and T2D-GENES cohorts, GWG in T1DM cohort and both phenotypes

Since it is known that diabetes and its treatment might impact BMI, we searched for variants that might affect BMI in the context of diabetes status. Since no T1DM cohort was available, we used T2D patients’ cohorts—T2D-GENES (590 patients) and ARIC (8746 patients) (being aware that the two phenotypes obesity and diabetes are interconnected and T2D patients are predisposed to obesity, while T1DM patients are not). After adjusting for diabetes, we found that 1812 genes were associated with BMI p < 0.05 (Additional file 7: Table S4). Among them, 135 influenced both phenotypes, 1677 influenced only BMI, and 513 influenced GWG (Additional file 8: Table S5, Fig. 3).

Again, we mapped variants to 1812 BMI associated genes (± 500000 bp) and used this panel to look for associations with GWG in T1DM cohort. When filtered by MAF > 5% and p-value < 1E−04 the analysis has shown that GWG correlates with 207 variants (Additional file 9: Table S6). Table 3 shows 20 variants which were significant at the level of p-value < 1E−05. We also performed the analysis of the association with GWG only for 135 genes which showed an overlap in TWAS. These data are presented in Additional file 5: Data 2 (marked as subAnalysis 2).

**Table 3:** The list of variants localized to genes associated with BMI in T2D-GENES and ARIC cohorts which associate with GWG

SNP ID	chr	MAF [%]	B	p-value	Gene/nearest gene	Localization/type
rs9690213	7	18.4	2.66	9.86E−07	*PODXL*	Regulatory region variant
rs11465293	7	5.7	4.46	3.17E−06	*CCL24*	Missense
rs9393623	6	31.0	2.18	4.11E−06	*CMAHP*	Intron
rs4796675	17	22.2	2.38	4.40E−06	*LINC00974*	Noncoding transcript exon variant
rs1978202	7	8.7	3.51	4.53E−06	*CCL26*	Intergenic
rs8080053	17	23.1	2.33	5.57E−06	*LINC00974*	Noncoding transcript exon variant
rs12534221	7	17.7	2.55	5.85E−06	*PODXL*	Intergenic
rs6747327	2	35.6	2.04	1.35E−05	*CAPN13*	Intron
rs4742339	9	37.8	1.97	2.97E−05	*KANK1*	Intergenic
rs11714248	3	43.0	− 1.86	4.40E−05	*TRANK1*	Intron
rs9347707	6	35.1	1.94	4.74E−05	*PACRG*	Intron
rs1398095	3	25.6	2.15	4.85E−05	*CAPDS*	Intron
rs9940705	16	6.2	3.61	6.91E−05	*FAM92B*	Intron
rs2421052	5	43.1	− 1.86	7.63E−05	*AC008691*	Intron
rs6815557	4	39.2	1.82	8.24E−05	*LOC107986225*	Intergenic
rs12788347	11	10.8	2.92	8.72E−05	*AL137224*	Intron
rs11235519	11	24.2	− 2.02	8.93E−05	*AP005019.4*	Intergenic
rs6546217	2	11.7	2.64	9.89E−05	*AC118345.1*	Intergenic
rs7609526	2	11.1	2.68	9.97E−05	*AC118345.1*	Intergenic

Most of the variants which influenced both GWG and BMI (p-value < 1E−05) were eQTLs associated with the expression of 13 genes (*LRRFIP2, MLH1, GOLGA4, CCL26, RHBDD2, AC004980, POR, CCL26, GTF2IRD2, UPK3B, ART2P, LINC00974, KTP15, KRT17*) in 14 tissues (Thyroid, Skeletal Muscle, Whole Blood, Artery, Cultured Fibroblasts, Tibial Nerve, Testis, Skin (Unexposed), Visceral Adipose Tissue, Minor Salivary Gland, Small Intestine, Colon, Brain (several regions).

FUMA analysis showed that among variants significant for association with GWG (p-value < 1E−04), 18 were listed for associations with multiple phenotypes in the GWAS Catalog. The KEGG analysis showed enrichment for the autoimmunological disease systemic lupus erythematosus (adj. p-value = 3.32E−07) and taste transduction (adj. p-value = 2.18E−02). In the molecular functions GO enrichment analysis we found these variants to be responsible, among others, for carbohydrate binding (adj. p-value = 3.15E−08), bitter taste receptor activity (adj. p-value = 4.51E−05), trace amine receptor activity (adj. p-value = 9.69E−05) and taste receptor activity (adj. p-value = 9.04E−04) (Additional file 10: Figure S2).

Finally, when comparing the lists of variants obtained in Analyses 1 and 2, we found a significant overlap, which supports our hypothesis that loci associated with BMI, both in the general population and diabetic cohorts, may also associate with GWG. Several SNPs—rs7564856 (*SPEG*), rs1541725 (*AC009502.4*), rs9347707 (*PCARG*), rs4329088 (*PCARG*), rs1978202 (*CCL26*), rs13340504 (*CCL24*), rs11465293 (*CCL24*), rs11037234 (*RP11-111A24.2*), rs876373 (*RPL29P25*), rs886131 (*RPL29P25*), rs7961894 (*WDR66*), rs4808209 (*ZNF101*) were found to influence GWG in T1DM, ARIC, T2D-GENES and GIANT cohorts. We conclude, that despite the relatively large overlap between traits at the gene level, the genetic variants which localize with these loci most often are linked to a single phenotype only.

Next, we aimed to study the “reverse association”—i.e. we asked whether genes associated with GWG influence BMI as well.

### PrediXcan analysis on GWG in T1DM cohort and variant associations with BMI in the general population (GIANT cohort)—Analysis 3

Variants were mapped to 648 GWG associated genes (± 500,000 bp) (Additional file 1: Table S1) and a search for associations with BMI in the GIANT cohort was performed. Among 394,149 variants analyzed, 2091 were significantly associated with BMI (at FDR < 0.05). These significant SNPs encompassed 0.53% of the whole list of variants analyzed. In comparison, the significant SNPs in the analysis of BMI in the GIANT cohort make up 0.41%, which means that genes which influence GWG are significantly enriched in the association test for BMI.

**Fig. 4:**
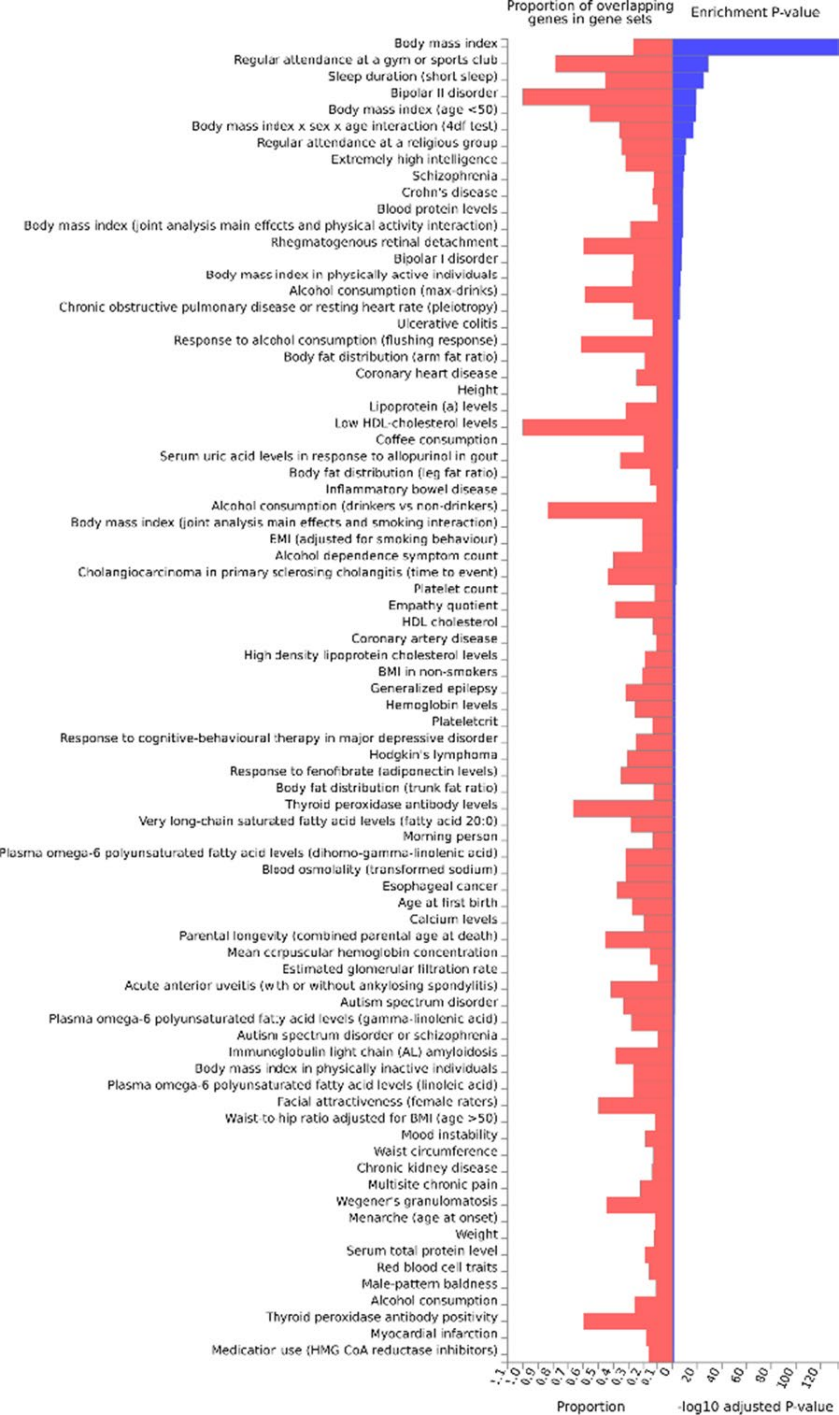
The enrichment analysis of variants localized to genes associated with GWG among those associated with BMI in the GIANT cohort

We used the FUMA tool to prioritize these 2091 variants. Twelve lead SNPs and 12 genomic risk loci were found. Not surprisingly, the GWAS Catalog analysis has shown a significant enrichment for genes associated with BMI, body fat distribution, attendance to gym or sport groups, sleep duration, height, lipoprotein levels, low HDL-level cholesterol etc. as presented in Fig. 4 and Additional file 11: Table S7.

Thus, we find a significant enrichment of variants associated with BMI in the general population within loci associated with GWG.

### PrediXcan analysis on GWG in T1DM cohort and variant associations with BMI in the diabetic cohort (ARIC)—Analysis 4

A GWAS analysis on BMI in the ARIC cohort was performed. Due to a large number of variants in the analysis we searched for lead SNPs using LD score regression. COJO analysis has returned 15 variants statistically significantly associated with the trait of interest (Table 4A).

**Table 4:** The list of variants localized to genes associated with BMI (A) and GWG (B) in ARIC cohort

SNP ID	chr	MAF [%]	B	p-value	Gene/nearest gene	Localization/type
A						
rs138445632	2	7.92	− 2.88	4.54E−12	*AC021851.2*	Intron
rs73646471	9	11.40	− 5.59	2.60E−10	*LOC105375974*	Intergenic
rs72703233	9	8.47	− 1.57	1.31E−07	*DMRT2*	Noncoding transcript exon variant
rs78231573	2	6.14	− 6.02	2.62E−07	*LOC105373643*	Intergenic
rs144768710	2	5.40	2.77	3.30E−07	*CHN1*	Intron
rs73419031	11	5.72	− 6.34	6.96E−07	*SOX6*	Intron
rs6456353	6	90.25	42.14	7.10E−07	*E2F3*	Intron
rs1486942	5	8.15	− 2.66	1.61E−06	*LOC112267929*	Intergenic
rs74036632	14	7.05	− 2.85	3.69E−06	*NYNRIN*	3′UTR
rs77346003	1	10.58	− 17.55	4.12E−06	*ADIPOR1*	Intergenic
rs73382735	9	5.68	4.49	4.28E−06	*LOC105375955*	Intergenic
rs12685009	9	8.42	− 8.94	7.74E−06	*SLC24A2*	Intergenic
rs34427781	22	19.46	− 2.54	8.47E−06	*TCF20*	Intron
rs13359900	5	15.98	− 27.84	8.95E−06	*MCTP1*	Intron
rs4131847	12	5.86	44.27	9.31E−06	*CCDC60*	Intergenic
B						
rs7875240	9	10.53	− 3.08	1.21E−06	*PTPRD*	Intron
rs1486942	5	8.15	− 2.66	1.61E−06	*LOC112267929*	Intergenic
rs74036632	14	7.05	− 2.85	3.69E−06	*NYNRIN*	3′UTR
rs34427781	22	19.46	− 2.54	8.47E−06	*TCF20*	Intron

Only 4 variants from this list were in GWG associated genes in PrediXcan, three of which overlapped with those associated with BMI (Table 4B). None of the variants were reported in GWAS Catalog or PheWas for the association with any trait. 6 variants were eQTLs—two for genes in which they were localized rs13359900 and rs73419031 for *MCTP1* and *SOX6*, respectively; rs144768710 for *ATP5G3*, rs74036632 for *ZFHX2-AS1*, rs4131847 for *HSPB8,* while rs34427781 for several genes all localized 200000 bp upstream or downstream from the variant.

Since the COJO analysis was performed on loci associated with GWG (± 500000 bp around the gene), it was possible that the most important variants influencing BMI were located outside those windows. Thus, we used a different tool, fastBAT, to find genes associated with BMI. The analysis revealed 8 genes which nominally associate with the trait of interest (Table 5).

**Table 5:** The list of variants localized to genes associated with BMI in ARIC cohort and associated with GWG as shown by fast-BAT

Gene/nearest gene	chr	Localization	# of SNPs	p-value	Top SNP p-value	Top SNP
*HLD-DQB*	6	Intergenic	4654	0.016	1.02E−06	rs115258523
*GATAD2*	19	Intron	1256	0.017	6.23E−05	rs145702982
*AC099791.3*	1	Intron	1800	0.020	8.85E−05	rs2485748
*LINC01141*	1	Intron	1501	0.027	9.95E−05	rs12027661
*HLA-DRB*	6	Intergenic	9354	0.029	1.89E−05	rs139964305
*HLA-B*	6	Stop gained	4017	0.044	2.11E−04	rs149512147
*LHFPL6*	13	Intron	1648	0.047	3.34E−06	rs6563700

Again, none the of variants was previously mentioned to be significantly associated with any trait in GWAS Catalog or PheWas. Two were eQTLs—rs6563700 for *LHFPL6*, while rs12027661 for *CAMK2N1*.

## Discussion

In this work we performed TWAS as well as single variant associations to determine the loci related to GWG and/or BMI. About 15% of loci overlap between BMI and GWG at the TWAS level. In the general population (GIANT cohort) the pathway analysis has shown that loci which contribute to both phenotypes affect mainly energy metabolism. Among the enriched pathways are regulation of mitochondrion (GO:1903749, GO:1903747, GO:0010822, GO:0070585, GO:0010821, GO:0006839) and Golgi apparatus (GO:0051683, GO:0048313, GO:0051645). There are also few pathways which affect transcription, polyadenylation or methylation of RNA (GO:1903311, GO:0016071, GO:1900363, GO:0080009). The analysis of the loci which TWAS associates with GWG only leads to necrosis (GO:0070266, GO:0097300, GO:0070265), protein kinases signaling (GO:0046330, GO:0043507, GO:0032874, GO:0070304, GO:0043506, GO:0007256) and metabolism of sugars (GO:0034033, GO:0034030, GO:0033866, GO:0009226). The BMI associated loci enrich cell cycle progression pathways (GO:0010972, GO:1902750) host to pathogen signaling (GO:0043921, GO:0052312, GO:0052472) and macromolecule metabolism (GO:0010604, GO:0034641, GO:0006139, GO:0009308, GO:0046483) and insulin signaling (GO:0046626, GO:1900076). The results of the GO enrichment analysis are presented in Additional file 12: Table S8 and Additional file 13: Figures S3a, b.

The analysis of the overlap between BMI and GWG associated loci in the ARIC cohort and T1DM cohort respectively showed enrichment in the TGFβ signaling pathway (GO:0071559, GO:0071560), regulation of stem cell differentiation (GO:1901532, GO:1902036, GO:0060218, GO:2000736) and polyol pathway (GO:0019751, GO:0046173). Pathways associated with GWG were similar to those enriched in the general population, however the important contribution of genes which affect antigen presentation could be seen (GO:0002495, GO:0002504, GO:0019886). The pathways characteristic for BMI associated loci were very much involved into response to environmental stimuli i.e. temperature (GO:0009266, GO:0009408), oxygen levels (GO:0070482, GO:0001666, GO:0036293) and sensory perception (GO:0050954, GO:0007600). The results of the GO enrichment analysis are presented in Additional file 14: Table S9 and Additional file 15: Figures S4a, b.

**Fig. 5:**
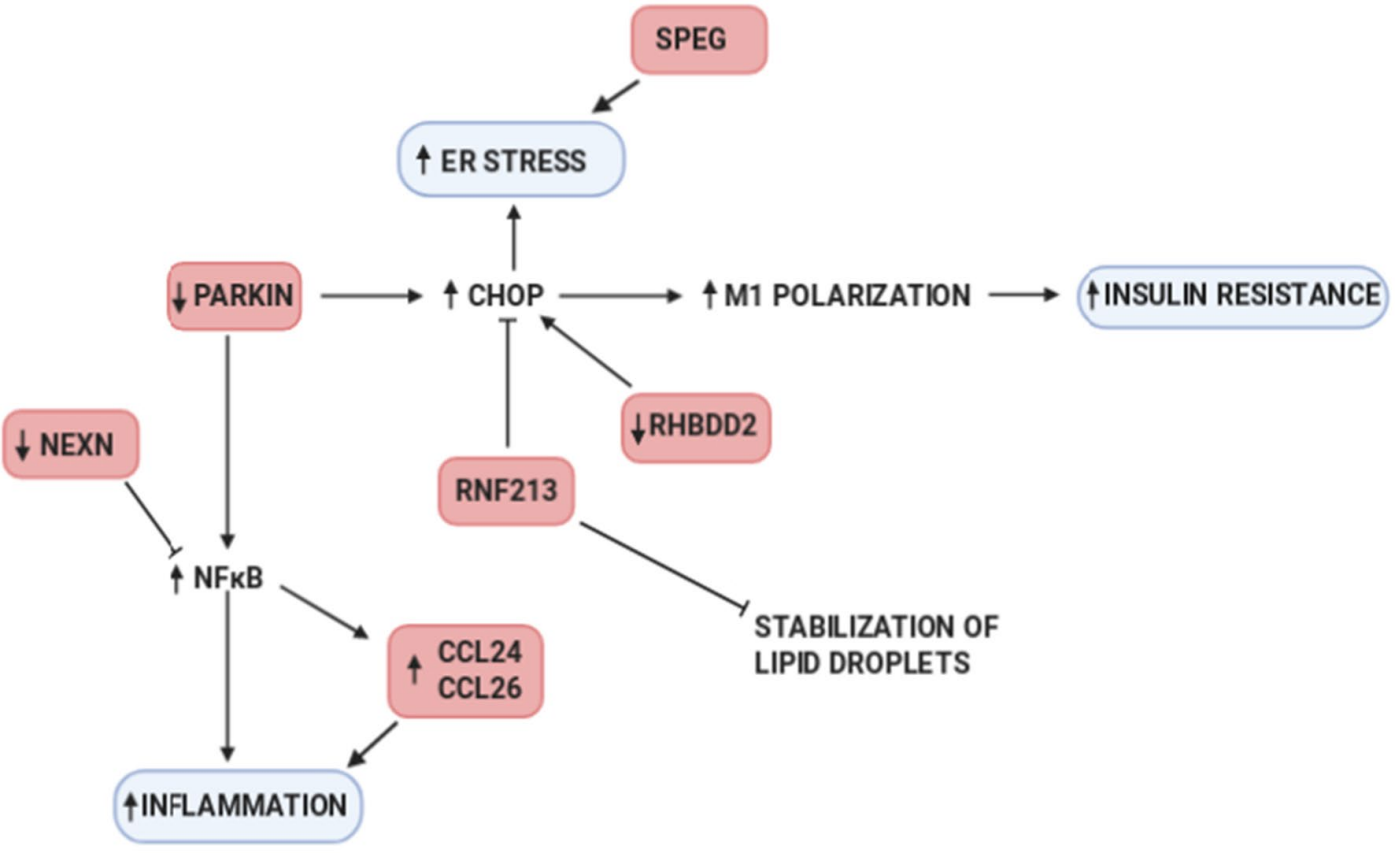
The schematic representation of pathways (blue) affected by genes which variants associate with GWG (pink)

**Fig. 6:**
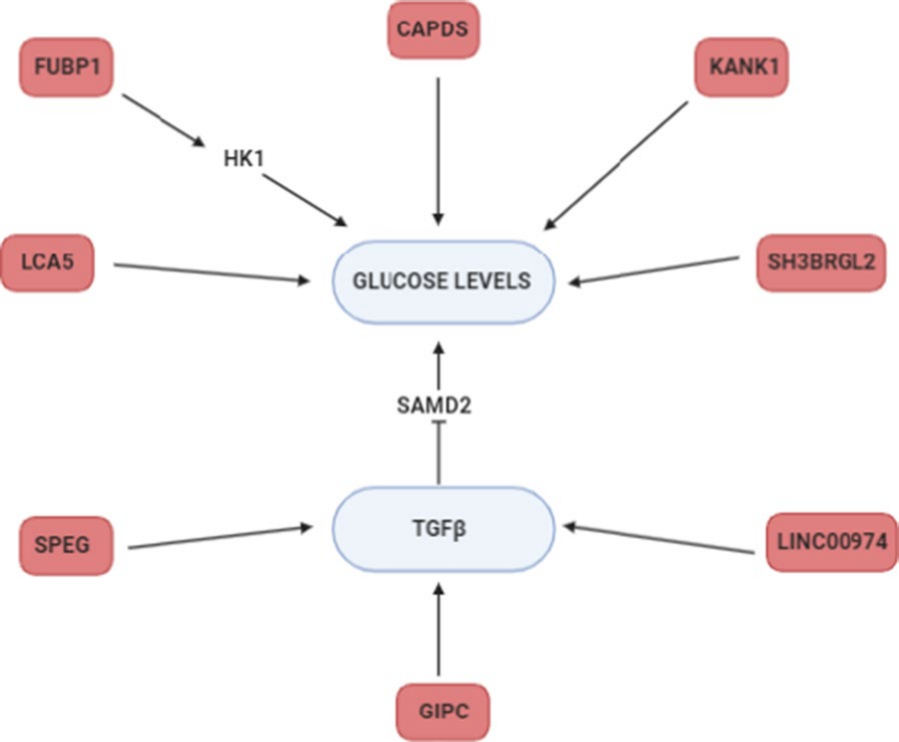
Genes which variants associate with GWG (pink) and that were reported to affect glucose levels or TGFβ signaling (blue)

At the genetic variant level, we detected SNPs in BMI associated loci that are related to GWG, and variants associated with both GWG and BMI (Analysis 1). At the same time, we performed a similar analysis with diabetes adjusted BMI and GWG, which led us to congruent findings (Analysis 2). These results point us towards inflammatory response, TGFβ signaling, ER stress and glucose homeostasis. Subsequently, we investigated the association of SNPs in loci which influenced GWG (based on TWAS) with BMI and found 2091 variants in the GIANT cohort (Analysis 3). A parallel analysis was done on diabetes adjusted BMI (in the ARIC cohort)—resulting in 15 variants localized to GWG associated loci (Analysis 4). The results of these analyses show an impact of lipid biosynthesis, appetite regulation, Ca^2+^ homeostasis (and ER stress) and inflammatory response on obesity. Our results point to the source of the genetic correlation between GWG and BMI and confirms the interconnection of the phenotypes. We compared the results of our gene focused analysis with GWAS on GWG published in 2018 (Warrington et al. 2018); even though variants from the discovery cohort did not replicate, their potential functional roles overlap with those found in our study. *TMEM163* is one of the best known genes associated with obesity, *LCORL* was shown to associate with height, *UGDH* with TGFβ signaling, *HLA-C* with autoimmune response, *HSD17B3* with fatty acids, *GLRX3* with oxidative stress, *RBM19* with ribosomal biogenesis, *SYT4* with Ca^2+^ binding and pancreatic functioning, *PSG5* with pregnancy development, and *NTS4* with neutrophilin signaling pathway. Our results are also congruent with those obtained in the computational search for functional annotation of 445 loci associated with obesity (Cheng et al. 2018). Therefore, we hypothesize that a further search for clinical phenotypes which affect GWG might allow identification of other loci that associate with it. Below we shed more light on the biological interpretation of our results and links between them (Figs. 5, 6).

In analyses 1 and 2, the strongest association with GWG was observed for SNPs in chemokine receptor ligands—rs1978202—*CCL26* and rs13340504, rs11465293—*CCL24*. Apart from variants located in these genes we identified several other (rs4742339, rs4796675, rs11235519) which serve as eQTLs for them. Chemokines are chemoattractant with the proinflammatory role. Their receptor expression was shown to correlate with BMI (being higher in obese individuals) (Ignacio et al. 2016) and BMI changes (decreased after bariatric surgery) (Gentili et al. 2016). CCL26 is also involved in adipose tissue beiging in response to cold (Finlin et al. 2017). At the same time, we find three variants rs9659938, rs11807240, rs2136682 associated with GWG in the *NEXN* gene (or serving as eQTL for it) also involved in the inflammatory response. Of note, the augmentation of NEXN antisense RNA (*NEXN-AS1*) inhibits TLR4 oligomerization and NFκB activity and leads to suppression of proinflammatory response (Hu et al. 2019). NEXN is also known for its abundant expression in striated muscles (Zhu et al. 2018) and cardiomyocytes; its expression is higher under high glucose conditions (Barbati et al. 2017) (Fig. 5).

Interestingly, we find GWG associated variants in genes involved in TGFβ signaling: *SPEG, LINC000974, GIPC2*. The *GIPC2* gene (rs2136682 of which associates with GWG), belongs to the GIPC family known for regulating proliferation, cytokinesis or migration and involved in the trafficking of various transmembrane proteins. GIPC1 is necessary for cell-surface expression of transmembrane receptors, such as IGF1R and TGFβR3 (Katoh 2013; Song et al. 2016). The inhibition of *LINC000974* leads to TGFβ secretion and repression of phosphorylated SMAD2 expression (Tang et al. 2014; Fang et al. 2019). Both TGFβ and SMADs are involved in insulin expression and signaling; SMAD4 deficiency was shown to improve glucose tolerance and glucose induced insulin release (Lin et al. 2009). Of note, three GWG associated variants (rs9690213, rs9393623, rs1978202) are eQTLs for *LINC000974* and four (rs8080053, rs1978202, rs4796675, rs8080053) are located in the gene itself (Fig. 6).

The rs7564856, negatively associated with GWG is located in *SPEG*, and its expression is significantly greater in endurance than in power athletes (Kusić et al. 2020). Also, at the protein level, SPEG differs between muscles of high-response vs low response rat trainers. The coimmunoprecipitation experiment with SPEG in the former showed a significant number of proteins involved in JNK and TGFβ signaling (Kusić et al. 2020), which, as said before, is known to influence insulin signaling and ER stress response (Liu et al. 2019) (Figs. 4, 5).

Two other variants—rs9347707 and rs4329088—located in *PACRG1* gene—are known to be involved in the insulin pathway. In *C. elegans* PACRG mutants have reduced insulin signaling. Also, PCARG plays a key role in the regulation of mitophagy (Stephenson et al. 2018) and ubiquitynylation (Loucks et al. 2016), its knockout leads to defective NFκB signaling (Meschede et al. 2020) and CHOP upregulation (Han et al. 2017), which in adipose tissue result in increased proinflammatory macrophage polarization (M1) and insulin resistance (Suzuki et al. 2017). Thus, we note that PACRG is also involved in ER stress. The *RHBDD2* gene is also involved in this biological process, with six eQTLs (rs4742339, rs11235519, rs1978202, rs13340504, rs4796675 and rs13340504) associated with GWG. It is worth noting that silencing of *RHBDD2* leads to increased expression of ATF6, IRE1, PERK, CRT, BiP, ATF4 and CHOP (Fig. 4).

The association of rs72849841 (in *RNF213*) with GWG draws our particular attention. Namely, in Akita mice (which develop diabetes spontaneously) the knockout of *RNF213* lowers glucose tolerance by 20% and leads to increased insulin contents in pancreases (by 150%) compared to wild type animals. This is obtained via the inhibition of ubiquitinoylation (RNF213 is a ubiquitin ligase) as shown by the 30% lower percentage of CHOP positive B-cells (Kobayashi et al. 2013). The depletion of *RNF213* protects cells from lipotoxicity (Piccolis et al. 2019), reduces palmitate-induced cell death and modifies non-mitochondrial oxygen consumption rate. Moreover, RNF213 is targeted to lipid droplets, which are specialized for neutral lipid storage and increases their abundance and stability in cells through the elimination of adipose triglyceride lipase (ATGL) (Sugihara et al. 2019). It is interesting to note that the latter is also negatively regulated by insulin (Kershaw et al. 2006). *RNF213* variants in GWAS analyses suggestively associated (p-value ~ E−03) with diabetic polyneuropathy, portal hypertension, diabetic retinopathy and abnormal glucose (Fig. 4). Its expression differed between cyclic and pregnant heifers (Forde et al. 2012).

We identified GWG associated loci involved in glucose homeostasis or pancreatic functioning (Fig. 5)—e.g. rs11807240 in *FUBP1*, which upregulates the mRNA levels of the two hexokinase genes *Hk1* and *Hk2*—the rate limiting enzymes of glycolysis. A positive correlation between *FUBP1* mRNA and both of the hexokinases was found in several types of cancers (Kang et al. 2019). Variants in *LCA5* gene were shown to modify glucose response in a clinical trial of insulin and potassium (GIK) infusion in acute coronary syndromes (Ellis et al. 2015). The rs9352745 associated with GWG is an eQTL for *SH3BRGL2*, whose expression is associated with diabetes (type 1, type 2 and gestational) in a meta-analysis study. An interaction between *SH3BRGL2* SNP and total fat intake was found to affect LDL-PPD (Rudkowska et al. 2015). We identified GWG associated SNP rs7609526 in *KANK1* related to fasting proinsulin (Huyghe et al. 2013) and fasting plasma glucose (Hwang et al. 2015) in GWAS. *CADPS* (with GWG associated SNP rs1398095) plays a role in insulin secretion (Speidel et al. 2008). Cg14451730 located near *CADPS* is differentially methylated with HbA1c levels in the Taiwan Biobank cohort (Hsiung et al. 2020). The suggestive association with fasting plasma glucose of rs487321 in the *CADPS* gene was found in an Arabian population of T2D patients (Hebbar et al. 2020). We also found a variant correlating with GWG in *RPL29P25* gene. The presence of anti-60S ribosomal protein L29 (RPL29) antibody in human serum was shown to inhibit the proliferation of pancreatic cancer cells in various cancers and is believed to be a novel candidate for a prognostic marker for unrespectable pancreatic cancer (Muro et al. 2015). The knockdown of *RPL29* leads to suppression of cell proliferation, induces cell arrest (at G0/G1phase), enhances cell apoptosis and decreases intracellular ROS generation (Li et al. 2012). The rs4808209 (in *ZNF1* gene) is an eQTL for *LPAR2*—the receptor for a signaling lipid LPA. It is a part of the LPAR2/Gab1/PI3K/Akt pathway, which influences glucose uptake (Rodriguez-Araujo et al. 2013) and is differentially expressed in fatty vs normal liver in the extreme obese cohort (DiStefano et al. 2014). Its expression correlates with the invasiveness of pancreatic cancers (Gong et al. 2012) as well as gynecological disorders (Yu et al. 2016; Wasniewski and Woclawek-Potocka 2018; Fujii et al. 2019; Kowalczyk-Zieba et al. 2019).

In Analyses 3 and 4, we set to determine whether genes associated with GWG also impact BMI. Basing on the summary results from the GIANT cohort we show a number of such genes. Some of their SNPs were previously associated with BMI, body fat distribution, attendance to gym or sport groups, sleep duration, height, lipoprotein levels, and low HDL-level cholesterol. At the same time, in a much smaller ARIC cohort we find 15 variants associated with diabetes adjusted BMI. None of them is listed in GWAS Catalog or PheWas; however, other variants in these genes were already mentioned to be associated with BMI.

Our analysis shows association with rs12685009 in the *SLC24A2* loci, which belongs to the family of K^+^ dependent Na^+^/Ca^2+^ exchanger (Schnetkamp 2013). A member of the family, SLC4 influences MC4R dependent satiety and the loss of MC4R function leads to prolonged obesity and reduced energy expenditure, while mice lacking *SLC4* display anorexia (Li and Lytton 2014). Also, variants in *SOX6*, a transcription factor shown to be involved in the promotion of adipogenesis (Iguchi et al. 2005; Leow et al. 2016), were previously related to obesity (Liu et al. 2009; Correa-Rodríguez et al. 2018). The rs77346003 in the *ADIPOR1* loci—a receptor for adiponectin known to regulate glucose metabolism and fatty acid oxidation—associates with BMI in our study. Its variants in GWAS are related to obesity (Beckers et al. 2013; Peters et al. 2013; Keustermans et al. 2017) as well as fetal weight (Fensterseifer et al. 2018; Muñoz-Muñoz et al. 2018). ADIPOR1 expression correlates with improved insulin sensitivity and PGC-1A expression—a master regulator of mitochondrial gene expression (Za’don et al. 2019). Moreover, our analysis identifies eQTLs for mitochondria associated genes *ATP5G3* (rs144768710) and *HSPB8* (rs4131847). ATP5G3 is involved in the proton pathway and acts as an energy-driving motor (Huang et al. 2013; He et al. 2017; Spataru et al. 2019). HSPB8 is known to prevent oxidative tissue damage and its expression in serum is used as a biomarker for virus induced type 1 diabetes (Karthik et al. 2012; Li et al. 2017; Yu et al. 2019). We also identified rs34427781—an eQTL for several genes (*CYP2D8P, NDUFA6-AS1, CYP2D6, CCDC134, CENPM, CHADL, FAM109B, NAGA, NDUFA6, OGFRP1, OLA1P1, SEPT3, SHISA8, SLC25A5P1, SMDT1, SREBF2, TNFRSF13C, WBP2NL*)—to be associated with BMI. It is worth noting that a subset of these genes is involved in lipid metabolism (*NAGA, SREBP*) or mitochondrial signaling (*SLC25A5P1, NDUFA6, SMDT1*). SREBP is a master regulator of lipid and sterol biosynthesis (Düvel et al. 2010); the gene–gene interactions between variants in the INSIG-SCAP-SREBP pathway are associated with risk of obesity in Chinese children (Liu et al. 2014). The *SMDT1* gene builds the mitochondrial calcium uniporter subunit (mtCU) localized in the inner mitochondrial membrane and is responsible for the Ca^2+^ transport to the mitochondrial matrix (Pendin et al. 2014), while its expression was shown to associate with fetal development (Vishnyakova et al. 2019). *SLC25* genes, members of SLC channels mentioned above, are known to be subunits of another complex essential for proper mitochondrial dynamics and energy production—ANC. ANC is responsible for mitochondrial/cytoplasmic ADP/ATP exchange (Clémençon et al. 2013) and its expression was shown to be consistently upregulated in obesity (Padilla et al. 2014). NDUFA6 is part of the first complex of the mitochondrial respiratory chain, which expression impacts oxidative stress and energy production efficacy (Fiedorczuk and Sazanov 2018). Lastly, the *TNFRSF13C* (*BAFF*) gene regulates insulin sensitivity (Kawasaki et al. 2013) and is associated with autoimmune diseases (Moisini and Davidson 2009).

The above results are congruent with the gene-based fBAT analysis that points us towards genes which correlate with BMI (*GATAD2, LHFPL6*) and immune response/autoimmunity (*HLA-DQB, HLA-DRB, HLA-B*). GATAD2A is involved in embryonic development (Wang et al. 2017) and associates with obesity (Saxena et al. 2012). A copy number variation of *LHFPL6* associates with average daily gain in cattle (Xu et al. 2019), while its rs4073643 associates with systemic lupus erythematosus in the Chinese population (Zhang et al. 2016). While the association between HLA variants and T1DM is very well known (Zhao et al. 2016), HLA loci are linked to other phenotypes (Karnes et al. 2017): type 2 diabetes (Ng et al. 2014), fatty liver disease (Doganay et al. 2014) as well as BMI (Shen et al. 2018) or waist-to-hip circumference (Wen et al. 2016). More importantly, these variants associate with trends in fetal birth weight (Capittini et al. 2009), level of inflammation in visceral adipose tissue in pregnant women (Eyerahi et al. 2018) and different mRNA levels in visceral omental adipose tissue of pregnant women with gestational diabetes (Deng et al. 2018). The *HLA-DQA1*, *HLADQB1* are differentially methylated in siblings born before vs after maternal bariatric surgery (Berglind et al. 2016).

## Conclusions

In this study we identified several loci which contribute to the genetic correlation between BMI and GWG. Variants identified in those loci are associated with genes linked to insulin signaling, glucose homeostasis, mitochondrial metabolism, ubiquitinylation and inflammatory responses and placenta functioning, not only in the diabetic cohorts, but also in the general population. The genetic contribution to GWG is clearly connected with BMI associated loci.

## Supplementary Information

**Additional file 1: Data S1.** The Table presenting the flow of the subject through the analysis process.

**Additional file 2: Table S1a.** The results of PrediXcan on BMI in T1D cohort in Subcutaneous Adipose Tissue. b. The results of PrediXcan on BMI in T1D cohort in Visceral Adipose Tissue. c. The results of PrediXcan on GWG in T1D cohort in Subcutaneous Adipose Tissue. d. The results of PrediXcan on GWG in T1D cohort in Visceral Adipose Tissue.

**Additional file 3: Table S2.** The overlap of PrediXcan results in Subcutaneous and Visceral Adipose Tissue in the Giant cohort.

**Additional file 4: Table S3a.** The results of PrediXcan on Giant cohort on BMI only. b. The results of PrediXcan on Giant cohort on BMI and GWG. c. The results of PrediXcan on Giant cohort on GWG only.

**Additional file 5: Data S2.** The subanalyses performed for genes in the overlap between GWG and BMI only.

**Additional file 6: Figure S1a.** The LD analysis for GPN3 gene. b. The LD analysis for PMS2P3 gene. c. The LD analysis for STAG3L1 gene.

**Additional file 7: Table S4a.**The results of PrediXcan on BMI in T2D cohort in Subcutaneous Adipose Tissue. b. The results of PrediXcan on BMI in T2D cohort in Visceral Adipose Tissue. c. The results of PrediXcan on BMI in ARIC cohort in Subcutaneous Adipose Tissue. d. The results of PrediXcan on BMI in ARIC cohort in Visceral Adipose Tissue.

**Additional file 8: Table S5a.** The results of PrediXcan on T2D&ARIC cohorts on BMI only. b. The results of PrediXcan on T2D&ARIC cohorts on BMI and GWG. c. The results of PrediXcan on T2D&ARIC cohorts on GWG only.

**Additional file 9: Table S6.** The list of variants associated with GWG in BMI associated genes in T2D&ARIC cohorts.

**Additional file 10: Figure S2.** The KEGG analysis on GWG associated variants in BMI associated genes in T2D&ARIC cohorts.

**Additional file 11: Table S7.** The FUMA analysis on BMI associated variants in GWG associated genes in T1DM cohort.

**Additional file 12:Table S8a.** Go Enrichment analysis on the overlap between BMI and GWG genes in the Giant cohort. b. Go Enrichment analysis on BMI only associated genes in the Giant cohort. c. Go Enrichment analysis on GWG only associated genes in the Giant cohort.

**Additional file 13: Figure S3a.** Go Enrichment analysis on GWG only associated genes in the Giant cohort. b. Go Enrichment analysis on the overlap between BMI and GWG genes in the Giant cohort.

**Additional file 14: Table S9a.** Go Enrichment analysis on the overlap between BMI and GWG genes in T2D and ARIC cohorts.b. Go Enrichment analysis on BMI only associated genes in T2D and ARIC cohorts. c. Go Enrichment analysis on GWG only associated genes in T2D and ARIC cohorts.

**Additional file 15: Figure S4a.** Go Enrichment analysis on GWG only associated genes in T2D and ARIC cohorts. b. Go Enrichment analysis on the overlap between BMI and GWG genes in T2D and ARIC cohorts.

## Data Availability

Sequence data has been deposited at the European Genome-phenome Archive (EGA), which is hosted by the EBI and the CRG, under accession number EGAS00001004408. Further information about EGA can be found on https://ega-archive.org. “The European Genome-phenome Archive of human data consented for biomedical research” (http://www.nature.com/ng/journal/v47/n7/full/ng.3312.html).

## Abbreviations

TWAS: Transcriptomic-wide association study
GWAS: Genome-wide association study
BMI: Body mass index
GWG: Gestational weight gain
eQTL: Expression quantitative trait loci
T1DM: Type 1 diabetes
