# Supplementary material for: The transcriptome-wide association search for genes and genetic variants which associate with BMI and gestational weight gain in women with type 1 diabetes
Source: Mol Med. 2021 Jan 20;27:6. doi: 10.1186/s10020-020-00266-z (PMC7818927; doi:10.1186/s10020-020-00266-z)
Supplement: Supplementary file 1 — Additional file 1: Data S1. The Table presenting the flow of the subject through the analysis process. [file 10020_2020_266_MOESM1_ESM.pdf]

Analysis 1				
**Type of analysis**	**Dataset**	**Number of patients**	**Tissues**	**Accession number for the primary dataset**
TWAS analysis for BMI	GIANT	234069 patients	Visceral and subcutaneous adipose tissue (genotypes from blood sample)	https://portals.broadinstitute.org/collaboration/giant/images/1/15/SNP_gwas_mc_merge_nogc.tbl.uniq.gz
Association analysis for GWG of variants in genes which in TWAS showed association with BMI (+/- 500000 bp)	T1DM	316 patients	n.a.	EGAS00001004408
**Analysis 2**				
**Type of analysis**	**Dataset**	**Number of patients**	**Tissues**	**Accession number for the primary dataset**
TWAS analysis for BMI	T2D-GENES and ARIC	T2D-GENES – 590 patients<br>ARIC – 8746 patients	Visceral and subcutaneous adipose tissue (genotypes from blood sample)	phs000462 and phs000090
Association analysis for GWG of variants in genes which in TWAS showed association with BMI (+/- 500000 bp)	T1DM	316 patients	n.a.	EGAS00001004408.
**Analysis 3**				
**Type of analysis**	**Dataset**	**Number of patients**	**Tissues**	**Accession number for the primary dataset**
TWAS for GWG	T1DM	316 patients	Visceral and subcutaneous adipose tissue (genotypes from blood sample)	EGAS00001004408

Association analysis for BMI of variants in genes which in TWAS showed association with BMI (+/- 500000 bp)	GIANT	234069 patients	n.a	https://portals.broadinstitute.org/collaboration/giant/images/1/15/SNP_gwas_mc_merge_nogc.tbl.uniq.gz
**Analysis 4**				
**Type of analysis**	**Dataset**	**Number of patients**	**Tissues**	**Accession number for the primary dataset**
TWAS for GWG	T1DM	316 patients	Visceral and subcutaneous adipose tissue (genotypes from blood sample)	EGAS00001004408
Association analysis for BMI of variants in genes which in TWAS showed association with BMI (+/- 500000 bp)	ARIC	8746 patients	n.a.	phs000090
