# Supplementary material for: The transcriptome-wide association search for genes and genetic variants which associate with BMI and gestational weight gain in women with type 1 diabetes
Source: Mol Med. 2021 Jan 20;27:6. doi: 10.1186/s10020-020-00266-z (PMC7818927; doi:10.1186/s10020-020-00266-z)
Supplement: Supplementary file 2 — Additional file 2: Table S1a. The results of PrediXcan on BMI in T1D cohort in Subcutaneous Adipose Tissue. b. The results of PrediXcan on BMI in T1D cohort in Visceral Adipose Tissue. c. The results of PrediXcan on GWG in T1D cohort in Subcutaneous Adipose Tissue. d. The results of PrediXcan on GWG in T1D cohort in Visceral Adipose Tissue. [file 10020_2020_266_MOESM2_ESM.zip › Table S1a.pdf]

geneID	logFC	AveExpr	t	P.Value	adj.P.Val
ENSG0000(	-0,006256	0,6410333	-3,912956	0,0001116	0,7459214
ENSG0000(	0,0051341	0,0757664	3,7171071	0,0002381	0,7459214
ENSG0000(	-0,003138	0,0039857	-3,6381	0,0003205	0,7459214
ENSG0000(	0,0040141	-0,034803	3,5960262	0,0003746	0,7459214
ENSG0000(	-0,003532	-0,199263	-3,495076	0,0005414	0,7459214
ENSG0000(	-0,006816	-0,37862	-3,4946	0,0005423	0,7459214
ENSG0000(	0,0053605	-0,100292	3,4479513	0,0006411	0,7558144
ENSG0000(	-0,008821	-0,323208	-3,377377	0,0008229	0,7823791
ENSG0000(	0,0037684	-0,187996	3,3122953	0,0010322	0,7823791
ENSG0000(	-0,006532	0,1600234	-3,271747	0,0011865	0,7823791
ENSG0000(	-0,010326	0,0277261	-3,26904	0,0011975	0,7823791
ENSG0000(	0,0062138	0,3104081	3,2650884	0,0012138	0,7823791
ENSG0000(	0,0072371	0,3368309	3,2516949	0,0012705	0,7823791
ENSG0000(	-0,018756	-0,278461	-3,219325	0,0014179	0,7823791
ENSG0000(	0,0010848	-0,049646	3,2159347	0,0014342	0,7823791
ENSG0000(	0,0033282	0,093864	3,1992998	0,0015168	0,7823791
ENSG0000(	-0,004808	0,0679016	-3,165296	0,0016995	0,8250625
ENSG0000(	-0,00205	0,0730004	-3,0836	0,0022247	0,8710043
ENSG0000(	0,0011758	0,0221059	3,078179	0,0022644	0,8710043
ENSG0000(	0,0042503	0,0247118	3,055617	0,0024366	0,8710043
ENSG0000(	-0,016446	-1,118148	-3,03921	0,0025692	0,8710043
ENSG0000(	-0,005888	0,0107987	-3,029385	0,0026518	0,8710043
ENSG0000(	0,0059425	-0,396	3,0239927	0,0026982	0,8710043
ENSG0000(	-0,004852	0,0182326	-3,022834	0,0027082	0,8710043
ENSG0000(	-0,015887	-0,097225	-3,015307	0,0027744	0,8710043
ENSG0000(	0,0037956	0,17786	3,0055923	0,002862	0,8710043
ENSG0000(	-0,007983	-0,089808	-2,994741	0,0029629	0,8710043
ENSG0000(	-0,011608	0,2877501	-2,984433	0,0030617	0,8710043
ENSG0000(	-0,003041	-0,022507	-2,919789	0,0037533	0,8710043
ENSG0000(	-0,028318	-1,02467	-2,919354	0,0037584	0,8710043
ENSG0000(	0,0047144	-0,010874	2,9028282	0,003957	0,8710043
ENSG0000(	-0,008064	0,2444339	-2,893542	0,0040728	0,8710043
ENSG0000(	-0,003815	0,000944	-2,892198	0,0040898	0,8710043
ENSG0000(	-0,014489	-0,523584	-2,873194	0,0043373	0,8710043
ENSG0000(	-0,004476	-0,01039	-2,867052	0,0044202	0,8710043
ENSG0000(	0,005073	-0,090701	2,8647533	0,0044516	0,8710043
ENSG0000(	-0,003617	-0,056725	-2,863324	0,0044712	0,8710043
ENSG0000(	-0,004855	-0,151685	-2,862364	0,0044844	0,8710043
ENSG0000(	-0,004319	0,1186282	-2,858573	0,004537	0,8710043
ENSG0000(	0,0061558	-0,146667	2,846582	0,0047069	0,8710043
ENSG0000(	0,0038468	-0,07273	2,8463799	0,0047098	0,8710043
ENSG0000(	-0,005324	-0,25473	-2,841967	0,0047738	0,8710043
ENSG0000(	-0,006825	0,0967081	-2,837898	0,0048336	0,8710043
ENSG0000(	-0,005616	0,0593388	-2,825447	0,0050206	0,8710043
ENSG0000(	-0,005956	-0,336847	-2,81659	0,0051576	0,8710043
ENSG0000(	-0,005774	0,0947427	-2,815725	0,0051711	0,8710043
ENSG0000(	-0,009733	-0,540222	-2,801107	0,0054053	0,8710043
ENSG0000(	-0,006036	0,059998	-2,795126	0,0055038	0,8710043

ENSG00000	0,0094148	-0,128908	2,7946603	0,0055115	0,8710043
ENSG00000	0,003892	0,1208641	2,789403	0,0055996	0,8710043
ENSG00000	-0,004855	-0,057204	-2,785466	0,0056664	0,8710043
ENSG00000	-0,013154	0,6951315	-2,783883	0,0056934	0,8710043
ENSG00000	0,0016282	0,0117746	2,782913	0,0057101	0,8710043
ENSG00000	0,015181	0,6540676	2,7802276	0,0057564	0,8710043
ENSG00000	-0,001336	0,0337175	-2,76773	0,0059763	0,8710043
ENSG00000	0,0033612	0,0566798	2,7634086	0,0060541	0,8710043
ENSG00000	-0,004503	0,1097526	-2,747384	0,0063506	0,8710043
ENSG00000	-0,009042	-0,003662	-2,741019	0,006472	0,8710043
ENSG00000	0,0059749	0,4220187	2,7356984	0,006575	0,8710043
ENSG00000	-0,008574	0,3935604	-2,733758	0,006613	0,8710043
ENSG00000	-0,004553	0,0223958	-2,732461	0,0066385	0,8710043
ENSG00000	0,0040928	0,0366008	2,7315307	0,0066568	0,8710043
ENSG00000	-0,014692	-0,665038	-2,726769	0,0067514	0,8710043
ENSG00000	-0,005969	0,1430344	-2,726615	0,0067544	0,8710043
ENSG00000	-0,008304	-0,126638	-2,698864	0,0073302	0,9300817
ENSG00000	-0,002338	0,0921081	-2,685759	0,0076172	0,9300817
ENSG00000	0,0043466	0,0639371	2,6838766	0,0076592	0,9300817
ENSG00000	0,0031144	0,0193566	2,6819421	0,0077026	0,9300817
ENSG00000	0,0031205	0,1248518	2,6770647	0,0078131	0,9300817
ENSG00000	-0,006284	0,2262804	-2,672446	0,007919	0,9300817
ENSG00000	-0,003266	-0,146732	-2,668892	0,0080014	0,9300817
ENSG00000	-0,013536	0,5191483	-2,648159	0,0084976	0,9729065
ENSG00000	-0,010869	0,6239411	-2,639894	0,008703	0,9729065
ENSG00000	0,0050494	-0,301345	2,6291891	0,0089756	0,9729065
ENSG00000	-0,004146	-0,302822	-2,623443	0,009125	0,9729065
ENSG00000	0,0027834	-0,189582	2,6177663	0,0092749	0,9729065
ENSG00000	-0,004848	-0,351281	-2,616837	0,0092996	0,9729065
ENSG00000	0,0046434	-0,180767	2,6128392	0,0094068	0,9729065
ENSG00000	-0,005314	0,3335944	-2,606597	0,0095762	0,9729065
ENSG00000	-0,005567	-0,045888	-2,606234	0,0095862	0,9729065
ENSG00000	0,0048072	-0,364403	2,6013057	0,009722	0,9729065
ENSG00000	-0,003639	-0,031759	-2,594447	0,0099139	0,9729065
ENSG00000	0,0014328	0,0029887	2,5897154	0,0100483	0,9729065
ENSG00000	-0,006981	0,3822751	-2,585599	0,0101665	0,9729065
ENSG00000	0,0037659	-0,0023	2,5805907	0,010312	0,9729065
ENSG00000	0,0068657	0,3052733	2,5778143	0,0103935	0,9729065
ENSG00000	-0,007339	-0,050911	-2,577035	0,0104165	0,9729065
ENSG00000	0,0062912	0,2853162	2,5716811	0,0105755	0,9729065
ENSG00000	-0,002858	0,0165588	-2,566299	0,0107376	0,9729065
ENSG00000	-0,003114	-0,100938	-2,561712	0,0108774	0,9729065
ENSG00000	-0,002192	-0,07436	-2,560308	0,0109206	0,9729065
ENSG00000	-0,005407	0,1958194	-2,554764	0,0110924	0,9729065
ENSG00000	0,0032855	-0,015159	2,547516	0,0113207	0,9729065
ENSG00000	0,0037439	-0,003867	2,5416403	0,0115088	0,9729065
ENSG00000	0,0044434	-0,160836	2,5368032	0,0116657	0,9729065
ENSG00000	0,0049185	-0,056501	2,5330552	0,0117886	0,9729065

ENSG00000(	-0,003676	0,0830859	-2,529484	0,0119068	0,9729065
ENSG00000(	0,0042908	0,0915559	2,5293613	0,0119109	0,9729065
ENSG00000(	0,0042762	0,1469153	2,5249244	0,0120593	0,9729065
ENSG00000(	-0,002056	-0,045571	-2,523047	0,0121225	0,9729065
ENSG00000(	-0,003509	-0,038781	-2,517569	0,0123089	0,9729065
ENSG00000(	-0,005995	-0,419319	-2,513925	0,0124342	0,9729065
ENSG00000(	0,0008833	-0,05938	2,5113912	0,012522	0,9729065
ENSG00000(	0,0038949	0,0639491	2,5111106	0,0125318	0,9729065
ENSG00000(	-0,008756	0,0498482	-2,508362	0,0126277	0,9729065
ENSG00000(	-0,00603	-0,15719	-2,507261	0,0126663	0,9729065
ENSG00000(	-0,005447	0,0616659	-2,497549	0,0130116	0,9729065
ENSG00000(	0,0129664	-0,921417	2,4971	0,0130278	0,9729065
ENSG00000(	0,0054878	0,3966196	2,4888199	0,013329	0,9729065
ENSG00000(	0,0045441	0,0284702	2,4886484	0,0133353	0,9729065
ENSG00000(	0,0022382	0,017558	2,4857286	0,0134431	0,9729065
ENSG00000(	-0,00245	0,1669204	-2,480773	0,0136277	0,9729065
ENSG00000(	-0,004948	0,3747242	-2,480498	0,013638	0,9729065
ENSG00000(	-0,003947	0,3351219	-2,464793	0,0142385	0,9729065
ENSG00000(	-0,008734	0,4881661	-2,460349	0,0144126	0,9729065
ENSG00000(	-0,005719	0,0092145	-2,455924	0,0145879	0,9729065
ENSG00000(	0,0045674	0,116924	2,4524688	0,014726	0,9729065
ENSG00000(	-0,003159	-0,001646	-2,447855	0,0149123	0,9729065
ENSG00000(	0,0045688	0,245	2,4415584	0,0151699	0,9729065
ENSG00000(	0,0052728	0,1206025	2,4381809	0,0153097	0,9729065
ENSG00000(	-0,004202	0,113957	-2,43666	0,015373	0,9729065
ENSG00000(	-0,011156	-0,274592	-2,434375	0,0154686	0,9729065
ENSG00000(	-0,002393	0,1510328	-2,433812	0,0154922	0,9729065
ENSG00000(	-0,008185	0,1745581	-2,431371	0,015595	0,9729065
ENSG00000(	0,0093139	-0,177243	2,4309902	0,0156111	0,9729065
ENSG00000(	-0,011218	0,3492591	-2,430035	0,0156515	0,9729065
ENSG00000(	0,0132378	-0,488931	2,4292978	0,0156828	0,9729065
ENSG00000(	0,0048079	-0,318056	2,4285791	0,0157133	0,9729065
ENSG00000(	0,0025802	0,0783862	2,4277536	0,0157485	0,9729065
ENSG00000(	0,0049351	-0,496288	2,4255973	0,0158406	0,9729065
ENSG00000(	-0,006367	0,1099785	-2,423219	0,0159427	0,9729065
ENSG00000(	-0,008719	-0,090504	-2,421661	0,01601	0,9729065
ENSG00000(	-0,001848	-0,045174	-2,421221	0,016029	0,9729065
ENSG00000(	0,0015319	-0,066366	2,4167252	0,0162246	0,9729065
ENSG00000(	-0,003796	-0,094096	-2,41173	0,0164444	0,9729065
ENSG00000(	0,0047979	0,1943586	2,4113857	0,0164596	0,9729065
ENSG00000(	0,0052435	-0,049269	2,4088577	0,016572	0,9729065
ENSG00000(	-0,003174	-0,155257	-2,406609	0,0166725	0,9729065
ENSG00000(	-0,005489	0,3505217	-2,405326	0,01673	0,9729065
ENSG00000(	-0,004141	0,2680887	-2,395294	0,0171863	0,9729065
ENSG00000(	0,0035805	-0,030482	2,3877663	0,0175358	0,9729065
ENSG00000(	0,0076969	0,5850117	2,3867472	0,0175836	0,9729065
ENSG00000(	0,0025366	0,1371941	2,3858029	0,017628	0,9729065
ENSG00000(	-0,00718	0,2934514	-2,384275	0,0177001	0,9729065

ENSG0000(	0,0024919	-0,071995	2,3755256	0,0181176	0,9729065
ENSG0000(	0,0036157	0,1661843	2,3754337	0,018122	0,9729065
ENSG0000(	-0,003177	0,1577893	-2,374597	0,0181624	0,9729065
ENSG0000(	0,005503	0,154975	2,3695997	0,0184053	0,9729065
ENSG0000(	0,0032252	0,0907004	2,3621715	0,0187716	0,9729065
ENSG0000(	-0,00564	0,2544379	-2,360029	0,0188784	0,9729065
ENSG0000(	0,0042684	0,3378487	2,3581116	0,0189744	0,9729065
ENSG0000(	0,0040923	0,0644512	2,3570667	0,019027	0,9729065
ENSG0000(	0,0030181	-0,097956	2,3563335	0,0190639	0,9729065
ENSG0000(	-0,012869	-0,571204	-2,355547	0,0191036	0,9729065
ENSG0000(	-0,00629	0,2052961	-2,35541	0,0191105	0,9729065
ENSG0000(	-0,00618	0,2603571	-2,353673	0,0191985	0,9729065
ENSG0000(	0,0057096	-0,03816	2,3476824	0,0195045	0,9729065
ENSG0000(	0,0056795	0,0783621	2,3433898	0,0197264	0,9729065
ENSG0000(	0,0056146	-0,553495	2,3432669	0,0197328	0,9729065
ENSG0000(	0,0023705	-0,072654	2,339038	0,0199536	0,9729065
ENSG0000(	-0,002079	0,0619621	-2,338848	0,0199636	0,9729065
ENSG0000(	0,0018894	-0,061411	2,3325282	0,0202978	0,9729065
ENSG0000(	-0,003858	0,0267552	-2,33116	0,0203709	0,9729065
ENSG0000(	-0,00461	0,9256922	-2,331055	0,0203764	0,9729065
ENSG0000(	-0,004639	0,3775583	-2,331016	0,0203785	0,9729065
ENSG0000(	-0,009654	-0,368189	-2,330539	0,020404	0,9729065
ENSG0000(	-0,003981	-0,021768	-2,323701	0,0207729	0,9729065
ENSG0000(	-0,005481	-0,183598	-2,32183	0,0208748	0,9729065
ENSG0000(	0,0080367	-0,041982	2,3211639	0,0209112	0,9729065
ENSG0000(	-0,003501	-0,216446	-2,320571	0,0209436	0,9729065
ENSG0000(	0,0069334	-0,257927	2,3174976	0,0211125	0,9729065
ENSG0000(	0,0072322	0,2747618	2,3169341	0,0211436	0,9729065
ENSG0000(	-0,002715	0,1887842	-2,316813	0,0211503	0,9729065
ENSG0000(	0,0051077	0,2477536	2,3147455	0,0212647	0,9729065
ENSG0000(	0,0052913	0,2986103	2,3130296	0,0213601	0,9729065
ENSG0000(	0,0014165	0,0743708	2,3120667	0,0214138	0,9729065
ENSG0000(	-0,002378	-0,118908	-2,310413	0,0215063	0,9729065
ENSG0000(	-0,007607	-0,225056	-2,308855	0,0215938	0,9729065
ENSG0000(	-0,005824	0,0236319	-2,302694	0,0219427	0,9729065
ENSG0000(	0,0054718	0,8697901	2,3000793	0,0220923	0,9729065
ENSG0000(	0,0063343	-0,364322	2,2952745	0,0223694	0,9729065
ENSG0000(	0,0047284	-0,4832	2,294844	0,0223944	0,9729065
ENSG0000(	0,0033937	0,0273266	2,2893162	0,0227173	0,9729065
ENSG0000(	0,003825	0,3645551	2,2883185	0,022776	0,9729065
ENSG0000(	0,0045763	-0,039648	2,2815688	0,0231768	0,9729065
ENSG0000(	-0,001471	0,0185464	-2,280972	0,0232125	0,9729065
ENSG0000(	-0,004542	-0,314156	-2,279371	0,0233085	0,9729065
ENSG0000(	0,0041497	0,296913	2,2775297	0,0234195	0,9729065
ENSG0000(	0,0036077	-0,100358	2,2754954	0,0235426	0,9729065
ENSG0000(	-0,010084	-0,429574	-2,274949	0,0235757	0,9729065
ENSG0000(	-0,003152	0,062274	-2,274094	0,0236277	0,9729065
ENSG0000(	0,0015306	0,0447058	2,2733917	0,0236705	0,9729065

ENSG00000(	0,0036036	0,017261	2,2724604	0,0237273	0,9729065
ENSG00000(	-0,001587	-0,014443	-2,27029	0,0238602	0,9729065
ENSG00000(	0,0074256	-0,762865	2,2702572	0,0238621	0,9729065
ENSG00000(	0,0077079	0,1723903	2,2679828	0,0240021	0,9729065
ENSG00000(	-0,003072	0,3404827	-2,267167	0,0240524	0,9729065
ENSG00000(	-0,003249	0,0439139	-2,266453	0,0240966	0,9729065
ENSG00000(	-0,001584	-0,028108	-2,266092	0,0241189	0,9729065
ENSG00000(	0,0064363	-0,019116	2,2641667	0,0242384	0,9729065
ENSG00000(	0,0032998	0,1904747	2,2634459	0,0242833	0,9729065
ENSG00000(	-0,008012	-0,243964	-2,262357	0,0243512	0,9729065
ENSG00000(	-0,005714	0,0208263	-2,261066	0,024432	0,9729065
ENSG00000(	0,0037009	0,0491465	2,2593408	0,0245402	0,9729065
ENSG00000(	-0,002675	0,1722857	-2,255804	0,0247635	0,9729065
ENSG00000(	0,0035721	-0,021557	2,2528927	0,0249486	0,9729065
ENSG00000(	0,0042967	0,2376224	2,2513777	0,0250454	0,9729065
ENSG00000(	0,0049465	0,1041166	2,247197	0,0253142	0,9729065
ENSG00000(	0,0079563	-0,469365	2,2470409	0,0253243	0,9729065
ENSG00000(	0,0014653	-0,090966	2,2395547	0,0258121	0,9729065
ENSG00000(	-0,003587	-0,169467	-2,239221	0,0258341	0,9729065
ENSG00000(	0,0037359	-0,257312	2,2348717	0,0261214	0,9729065
ENSG00000(	0,0024515	0,0504099	2,2348039	0,0261259	0,9729065
ENSG00000(	0,0053578	0,0337103	2,2334574	0,0262155	0,9729065
ENSG00000(	-0,004255	-0,026984	-2,231913	0,0263185	0,9729065
ENSG00000(	0,0079393	0,1043292	2,2318775	0,0263208	0,9729065
ENSG00000(	-0,004017	0,1024226	-2,231547	0,0263429	0,9729065
ENSG00000(	-0,006234	-0,467085	-2,229759	0,0264627	0,9729065
ENSG00000(	-0,002713	-0,023082	-2,22952	0,0264788	0,9729065
ENSG00000(	0,0127175	-0,690802	2,2289938	0,0265142	0,9729065
ENSG00000(	0,0028646	0,1268175	2,2288712	0,0265224	0,9729065
ENSG00000(	-0,005541	-0,357712	-2,226369	0,0266912	0,9729065
ENSG00000(	-0,002216	-0,053708	-2,224921	0,0267893	0,9729065
ENSG00000(	-0,006247	0,2828768	-2,220129	0,0271162	0,9729065
ENSG00000(	0,0043236	0,1117175	2,2196627	0,0271482	0,9729065
ENSG00000(	-0,004062	0,3782501	-2,218106	0,0272552	0,9729065
ENSG00000(	-0,004597	-0,620831	-2,21506	0,0274657	0,9729065
ENSG00000(	-0,009682	0,5355756	-2,21364	0,0275644	0,9729065
ENSG00000(	-0,005198	-0,364089	-2,212719	0,0276285	0,9729065
ENSG00000(	-0,007171	0,5551157	-2,206935	0,0280342	0,9729065
ENSG00000(	-0,004644	-0,037687	-2,206622	0,0280563	0,9729065
ENSG00000(	0,0070899	-0,010298	2,2043512	0,028217	0,9729065
ENSG00000(	0,0026816	0,2051901	2,2041645	0,0282303	0,9729065
ENSG00000(	0,0062134	0,3478176	2,2037887	0,028257	0,9729065
ENSG00000(	-0,004496	0,0010152	-2,202241	0,0283671	0,9729065
ENSG00000(	0,003174	0,0589484	2,201771	0,0284007	0,9729065
ENSG00000(	-0,004124	-0,254537	-2,198178	0,0286582	0,9729065
ENSG00000(	0,0036071	0,0152442	2,1975769	0,0287015	0,9729065
ENSG00000(	0,0026445	0,313158	2,1973107	0,0287206	0,9729065
ENSG00000(	-0,009101	-0,266088	-2,195361	0,0288615	0,9729065

ENSG0000(	0,003093	0,0378741	2,1946412	0,0289136	0,9729065
ENSG0000(	-0,007681	-0,439231	-2,193057	0,0290286	0,9729065
ENSG0000(	0,0021188	0,5360011	2,1930365	0,0290301	0,9729065
ENSG0000(	0,0032365	0,0899139	2,1902302	0,0292349	0,9729065
ENSG0000(	-0,001953	0,0829998	-2,186345	0,0295205	0,9729065
ENSG0000(	0,0087959	-0,035287	2,1860132	0,029545	0,9729065
ENSG0000(	0,005898	-0,007965	2,183149	0,0297572	0,9729065
ENSG0000(	0,0017478	-0,079807	2,1807559	0,0299355	0,9729065
ENSG0000(	-0,002827	-0,040103	-2,179299	0,0300445	0,9729065
ENSG0000(	-0,003151	0,0900344	-2,178947	0,0300709	0,9729065
ENSG0000(	0,0032846	0,031093	2,1787483	0,0300858	0,9729065
ENSG0000(	0,0032659	-0,009801	2,1773293	0,0301924	0,9729065
ENSG0000(	0,0045663	0,1271701	2,1771996	0,0302022	0,9729065
ENSG0000(	0,0013761	0,2069042	2,1762253	0,0302756	0,9729065
ENSG0000(	0,003035	-0,196794	2,1743587	0,0304167	0,9729065
ENSG0000(	0,0066416	0,0577599	2,1729783	0,0305214	0,9729065
ENSG0000(	-0,003431	-0,009943	-2,172261	0,0305759	0,9729065
ENSG0000(	-0,002566	0,131357	-2,170874	0,0306816	0,9729065
ENSG0000(	0,0019721	0,0117676	2,1673403	0,0309523	0,9729065
ENSG0000(	0,0039563	0,0455301	2,1668134	0,0309928	0,9729065
ENSG0000(	-0,000693	-0,010302	-2,166626	0,0310073	0,9729065
ENSG0000(	-0,003419	0,2972884	-2,16636	0,0310278	0,9729065
ENSG0000(	0,0114861	0,22644	2,163799	0,0312256	0,9729065
ENSG0000(	-0,005116	-0,503198	-2,163663	0,0312361	0,9729065
ENSG0000(	-0,003033	-0,045201	-2,163619	0,0312396	0,9729065
ENSG0000(	0,0033105	-0,077193	2,1597902	0,0315375	0,9735698
ENSG0000(	0,0045234	-0,177868	2,1590131	0,0315983	0,9735698
ENSG0000(	0,0042014	0,0534388	2,1585747	0,0316326	0,9735698
ENSG0000(	-0,002935	-0,058301	-2,156189	0,03182	0,9735698
ENSG0000(	0,0010801	0,0540083	2,1542384	0,0319739	0,9735698
ENSG0000(	-0,003168	-0,195003	-2,152393	0,0321201	0,9735698
ENSG0000(	-0,004754	-0,008301	-2,147947	0,0324748	0,9735698
ENSG0000(	-0,004295	0,7340335	-2,14608	0,0326246	0,9735698
ENSG0000(	0,0037088	0,1722066	2,1453479	0,0326836	0,9735698
ENSG0000(	0,0045278	0,0459026	2,1414923	0,0329955	0,9735698
ENSG0000(	-0,003843	0,2809563	-2,141115	0,0330262	0,9735698
ENSG0000(	0,0026265	-0,371321	2,1344848	0,0335691	0,9735698
ENSG0000(	-0,00175	-0,134466	-2,133499	0,0336505	0,9735698
ENSG0000(	0,0031327	-0,144607	2,1334968	0,0336506	0,9735698
ENSG0000(	0,0087061	-0,671998	2,1332129	0,0336741	0,9735698
ENSG0000(	0,0052349	-0,05966	2,131548	0,033812	0,9735698
ENSG0000(	0,0072016	-0,369082	2,1315091	0,0338152	0,9735698
ENSG0000(	-0,007488	-0,164675	-2,130787	0,0338752	0,9735698
ENSG0000(	0,0022975	-0,235925	2,126414	0,0342402	0,9735698
ENSG0000(	0,0015693	-0,091635	2,1245638	0,0343957	0,9735698
ENSG0000(	-0,001484	0,0628811	-2,124382	0,034411	0,9735698
ENSG0000(	-0,005912	-0,34198	-2,123745	0,0344647	0,9735698
ENSG0000(	0,005459	0,3953206	2,1222745	0,0345889	0,9735698

ENSG0000(	0,002051	0,1130394	2,1197983	0,0347989	0,9735698
ENSG0000(	0,0036089	-0,005998	2,119788	0,0347998	0,9735698
ENSG0000(	0,0051836	0,0299365	2,1194752	0,0348264	0,9735698
ENSG0000(	-0,007341	-0,533172	-2,119318	0,0348398	0,9735698
ENSG0000(	0,0085541	-0,190881	2,1141727	0,0352802	0,9735698
ENSG0000(	-0,009252	-0,326961	-2,113649	0,0353253	0,9735698
ENSG0000(	-0,002759	0,5927553	-2,113624	0,0353275	0,9735698
ENSG0000(	0,0011608	0,0478466	2,1119272	0,0354739	0,9735698
ENSG0000(	0,0013865	-0,055414	2,1111008	0,0355454	0,9735698
ENSG0000(	-0,001623	-0,050783	-2,107542	0,0358547	0,9735698
ENSG0000(	0,0073933	0,3025736	2,1050128	0,036076	0,9735698
ENSG0000(	-0,002043	0,0514435	-2,104005	0,0361645	0,9735698
ENSG0000(	0,0041278	-0,099335	2,1026415	0,0362845	0,9735698
ENSG0000(	-0,002122	0,0653087	-2,099219	0,0365873	0,9735698
ENSG0000(	0,0087638	0,1774919	2,0987735	0,0366268	0,9735698
ENSG0000(	0,003207	-0,031762	2,0978806	0,0367062	0,9735698
ENSG0000(	-0,002097	-0,013176	-2,095863	0,0368862	0,9735698
ENSG0000(	-0,008335	0,3762152	-2,095339	0,0369331	0,9735698
ENSG0000(	0,0024814	-0,047906	2,093214	0,0371236	0,9735698
ENSG0000(	0,0040117	-0,026576	2,0918292	0,0372483	0,9735698
ENSG0000(	0,0034406	-0,064922	2,0911218	0,0373121	0,9735698
ENSG0000(	-0,002548	-0,009006	-2,089423	0,0374658	0,9735698
ENSG0000(	0,0021352	0,0288177	2,0893539	0,037472	0,9735698
ENSG0000(	0,004386	0,4537303	2,0886476	0,037536	0,9735698
ENSG0000(	0,0024658	0,2103312	2,0885167	0,0375479	0,9735698
ENSG0000(	0,0050591	0,1169153	2,087954	0,037599	0,9735698
ENSG0000(	0,0031135	0,291738	2,0858764	0,0377882	0,9735698
ENSG0000(	0,0027818	0,8415544	2,0835595	0,0380001	0,9735698
ENSG0000(	0,0045644	-0,217794	2,0829453	0,0380565	0,9735698
ENSG0000(	0,0100501	-0,680726	2,082405	0,0381061	0,9735698
ENSG0000(	0,0094614	0,6743745	2,0804038	0,0382905	0,9735698
ENSG0000(	0,0031832	-0,208654	2,0777534	0,0385357	0,9735698
ENSG0000(	0,0112609	-0,636883	2,0772824	0,0385795	0,9735698
ENSG0000(	-0,003831	0,2245492	-2,07615	0,0386848	0,9735698
ENSG0000(	0,0071567	-0,316231	2,075851	0,0387126	0,9735698
ENSG0000(	-0,003352	0,104316	-2,075361	0,0387583	0,9735698
ENSG0000(	-0,009212	-0,234426	-2,069858	0,0392744	0,9735698
ENSG0000(	-0,004359	0,3813907	-2,068913	0,0393637	0,9735698
ENSG0000(	-0,003489	0,1095797	-2,06734	0,0395125	0,9735698
ENSG0000(	-0,00149	-0,057001	-2,065898	0,0396494	0,9735698
ENSG0000(	-0,004605	-0,160191	-2,064119	0,0398189	0,9735698
ENSG0000(	0,0022382	-0,090182	2,0637125	0,0398577	0,9735698
ENSG0000(	0,0012007	-0,03862	2,0635905	0,0398693	0,9735698
ENSG0000(	0,0099959	0,5669547	2,0635604	0,0398722	0,9735698
ENSG0000(	-0,004018	0,0572443	-2,063256	0,0399013	0,9735698
ENSG0000(	0,0096579	0,2279227	2,060822	0,0401346	0,9735698
ENSG0000(	-0,006204	-0,335569	-2,05835	0,0403727	0,9735698
ENSG0000(	-0,003692	-0,089917	-2,057396	0,0404649	0,9735698

ENSG0000(	0,0051137	-0,285174	2,0572382	0,0404801	0,9735698
ENSG0000(	0,00375	-0,018708	2,0568887	0,040514	0,9735698
ENSG0000(	-0,003215	-0,145495	-2,056074	0,040593	0,9735698
ENSG0000(	-0,003275	-0,072268	-2,055189	0,0406789	0,9735698
ENSG0000(	0,0042837	-0,013424	2,051442	0,0410444	0,9735698
ENSG0000(	-0,002401	-0,08646	-2,050725	0,0411147	0,9735698
ENSG0000(	-0,002176	0,028985	-2,047358	0,041446	0,9735698
ENSG0000(	0,0022986	0,0377926	2,0462303	0,0415575	0,9735698
ENSG0000(	0,0047733	0,1228406	2,0451607	0,0416634	0,9735698
ENSG0000(	-0,003104	0,0820985	-2,044073	0,0417714	0,9735698
ENSG0000(	0,0048849	0,3240185	2,0420584	0,0419721	0,9735698
ENSG0000(	0,0041409	0,059254	2,0411512	0,0420627	0,9735698
ENSG0000(	0,0063089	-0,012465	2,0397731	0,0422007	0,9735698
ENSG0000(	-0,002846	-0,040063	-2,039368	0,0422413	0,9735698
ENSG0000(	0,005345	0,0209532	2,0393566	0,0422425	0,9735698
ENSG0000(	-0,005546	-0,237944	-2,038876	0,0422907	0,9735698
ENSG0000(	-0,003125	0,1105252	-2,036655	0,0425144	0,9735698
ENSG0000(	0,0017247	-0,106776	2,0364463	0,0425354	0,9735698
ENSG0000(	0,0051471	-0,188434	2,0351992	0,0426615	0,9735698
ENSG0000(	0,0044895	0,1583172	2,0350274	0,0426788	0,9735698
ENSG0000(	-0,003864	-0,196103	-2,03482	0,0426998	0,9735698
ENSG0000(	0,0021506	-0,016915	2,034105	0,0427723	0,9735698
ENSG0000(	0,0035594	0,0967092	2,0330007	0,0428844	0,9735698
ENSG0000(	-0,001825	0,0727981	-2,031538	0,0430333	0,9735698
ENSG0000(	0,0037182	0,115631	2,0314065	0,0430467	0,9735698
ENSG0000(	0,002649	-0,18806	2,0311447	0,0430734	0,9735698
ENSG0000(	-0,004031	0,2301045	-2,030302	0,0431595	0,9735698
ENSG0000(	0,0027657	0,0301042	2,0291561	0,0432767	0,9735698
ENSG0000(	-0,003646	-0,529012	-2,028939	0,0432989	0,9735698
ENSG0000(	-0,007088	-0,098063	-2,028766	0,0433167	0,9735698
ENSG0000(	0,0033584	0,1783555	2,0287599	0,0433173	0,9735698
ENSG0000(	0,0073079	0,1571853	2,0253965	0,0436633	0,9735698
ENSG0000(	0,009606	0,6137924	2,0245529	0,0437505	0,9735698
ENSG0000(	0,0042049	-0,152915	2,0239262	0,0438153	0,9735698
ENSG0000(	0,0039533	-0,200415	2,0218797	0,0440276	0,9735698
ENSG0000(	0,0074568	-0,312218	2,0205084	0,0441703	0,9735698
ENSG0000(	0,0060192	-0,148118	2,020483	0,0441729	0,9735698
ENSG0000(	0,0033518	0,1750783	2,0201962	0,0442028	0,9735698
ENSG0000(	0,0034564	0,0831226	2,014924	0,0447556	0,9735698
ENSG0000(	0,0043175	0,057432	2,0144866	0,0448017	0,9735698
ENSG0000(	-0,005552	-0,293354	-2,012038	0,0450607	0,9735698
ENSG0000(	0,0106107	0,3700348	2,0112929	0,0451397	0,9735698
ENSG0000(	0,0034566	-0,022022	2,0102514	0,0452504	0,9735698
ENSG0000(	0,0054295	-0,285206	2,0100993	0,0452666	0,9735698
ENSG0000(	-0,000521	0,002113	-2,009982	0,045279	0,9735698
ENSG0000(	-0,002025	-0,511952	-2,009742	0,0453046	0,9735698
ENSG0000(	-0,002064	-0,136312	-2,009476	0,0453329	0,9735698
ENSG0000(	-0,010114	0,0126574	-2,007036	0,0455936	0,9735698

ENSG00000(	0,0031901	0,0784232	2,006894	0,0456088	0,9735698
ENSG00000(	0,0051188	0,2970325	2,006575	0,0456429	0,9735698
ENSG00000(	0,0043927	0,0392398	2,0052859	0,0457813	0,9735698
ENSG00000(	-0,001473	0,0266861	-2,004419	0,0458744	0,9735698
ENSG00000(	0,003686	0,112484	2,0042877	0,0458886	0,9735698
ENSG00000(	-0,002972	-0,503376	-2,002504	0,046081	0,9750037
ENSG00000(	0,0019496	-0,141567	2,0007302	0,0462729	0,9750037
ENSG00000(	0,0035687	0,2009999	1,9991239	0,0464474	0,9750037
ENSG00000(	0,0021668	-0,037236	1,9984587	0,0465198	0,9750037
ENSG00000(	0,0012213	0,008608	1,9982096	0,0465469	0,9750037
ENSG00000(	-0,003279	-0,120718	-1,995867	0,0468027	0,9751521
ENSG00000(	0,0073636	0,050627	1,9958321	0,0468066	0,9751521
ENSG00000(	0,0064397	-0,078141	1,9949022	0,0469084	0,9751521
ENSG00000(	-0,00231	-0,173045	-1,992314	0,047193	0,9774668
ENSG00000(	0,0065169	0,1977794	1,991737	0,0472567	0,9774668
ENSG00000(	-0,011819	-0,232597	-1,987547	0,047721	0,9788698
ENSG00000(	0,0025354	-0,154231	1,9866457	0,0478214	0,9788698
ENSG00000(	-0,007986	-0,064365	-1,982417	0,0482948	0,9788698
ENSG00000(	-0,00731	-0,122582	-1,980672	0,0484912	0,9788698
ENSG00000(	-0,002058	-0,126203	-1,980523	0,048508	0,9788698
ENSG00000(	-0,003164	-0,036199	-1,980154	0,0485496	0,9788698
ENSG00000(	-0,001329	-0,043131	-1,979916	0,0485766	0,9788698
ENSG00000(	0,0037253	-0,102157	1,9792907	0,0486472	0,9788698
ENSG00000(	-0,006039	-0,315087	-1,97925	0,0486518	0,9788698
ENSG00000(	-0,004526	0,2596726	-1,979224	0,0486547	0,9788698
ENSG00000(	0,0075464	0,4586523	1,978152	0,0487761	0,9788698
ENSG00000(	0,0008468	0,3813735	1,9764381	0,0489707	0,9788698
ENSG00000(	0,0025787	-0,12982	1,9762182	0,0489957	0,9788698
ENSG00000(	-0,009593	-0,468049	-1,975564	0,0490703	0,9788698
ENSG00000(	0,0090721	0,7599186	1,9752711	0,0491036	0,9788698
ENSG00000(	0,0026788	-0,331223	1,9738048	0,049271	0,9798397
ENSG00000(	0,0035107	0,1391027	1,9675079	0,0499953	0,981648

geneID	logFC	AveExpr	t	P.Value	adj.P.Val
ENSG0000(	-0,004368	0,0552769	-3,490755	0,0005498	0,9512444
ENSG0000(	0,0161776	0,7222968	3,3324548	0,0009625	0,9512444
ENSG0000(	-0,00728	0,0946943	-3,287465	0,0011243	0,9512444
ENSG0000(	0,0057199	-0,138436	3,2616086	0,0012283	0,9512444
ENSG0000(	-0,00396	0,1446057	-3,195361	0,001537	0,9512444
ENSG0000(	-0,004432	-0,220306	-3,192631	0,0015511	0,9512444
ENSG0000(	0,0050378	0,0959801	3,1354629	0,0018763	0,9512444
ENSG0000(	-0,009178	-0,296935	-3,091923	0,002165	0,9512444
ENSG0000(	-0,002773	0,2624972	-3,034189	0,0026111	0,9512444
ENSG0000(	-0,004316	0,2028125	-3,004077	0,0028759	0,9512444
ENSG0000(	-0,002739	-0,11562	-3,000278	0,002911	0,9512444
ENSG0000(	0,0024585	-0,136419	2,9916569	0,0029921	0,9512444
ENSG0000(	0,0053989	-0,175995	2,9672535	0,003233	0,9512444
ENSG0000(	-0,001596	-0,047097	-2,95887	0,0033198	0,9512444
ENSG0000(	-0,004793	-0,144734	-2,935467	0,0035735	0,9512444
ENSG0000(	-0,007011	-0,656663	-2,923424	0,0037109	0,9512444
ENSG0000(	-0,003688	0,1398046	-2,914485	0,0038159	0,9512444
ENSG0000(	-0,004552	0,1490001	-2,905281	0,0039269	0,9512444
ENSG0000(	0,0037791	0,0863215	2,9038525	0,0039444	0,9512444
ENSG0000(	-0,001997	-0,020623	-2,886654	0,0041606	0,9512444
ENSG0000(	-0,003994	0,1535144	-2,85631	0,0045686	0,9512444
ENSG0000(	0,0059276	-0,191384	2,8416793	0,004778	0,9512444
ENSG0000(	0,0070816	0,097853	2,8382634	0,0048281	0,9512444
ENSG0000(	0,0053929	0,0038463	2,8336999	0,0048958	0,9512444
ENSG0000(	-0,004359	0,1279647	-2,831719	0,0049255	0,9512444
ENSG0000(	0,0087885	-0,136694	2,8253178	0,0050225	0,9512444
ENSG0000(	-0,004922	-0,014796	-2,822061	0,0050725	0,9512444
ENSG0000(	0,0078214	0,2865626	2,8114577	0,0052385	0,9512444
ENSG0000(	-0,017323	-0,418059	-2,802212	0,0053872	0,9512444
ENSG0000(	-0,014476	0,4286522	-2,79447	0,0055146	0,9512444
ENSG0000(	-0,00562	-0,09098	-2,792414	0,0055489	0,9512444
ENSG0000(	-0,003682	-0,085538	-2,776744	0,0058168	0,9512444
ENSG0000(	0,0045487	0,093469	2,7639148	0,0060448	0,9512444
ENSG0000(	0,004653	0,0589206	2,7414604	0,0064634	0,9512444
ENSG0000(	0,0017928	0,0180798	2,7285291	0,0067162	0,9512444
ENSG0000(	0,0062085	0,0221891	2,7169894	0,0069493	0,9512444
ENSG0000(	0,0039638	0,2794842	2,7084969	0,0071254	0,9512444
ENSG0000(	-0,005835	-0,204733	-2,703743	0,0072258	0,9512444
ENSG0000(	0,0072074	-0,204372	2,70279	0,0072461	0,9512444
ENSG0000(	0,0070337	0,5656473	2,6960725	0,0073904	0,9512444
ENSG0000(	0,0031545	0,068341	2,6943732	0,0074273	0,9512444
ENSG0000(	0,0125014	-0,754311	2,691942	0,0074805	0,9512444
ENSG0000(	-0,008185	-1,152406	-2,679043	0,0077681	0,9512444
ENSG0000(	-0,002301	-0,048818	-2,679022	0,0077685	0,9512444
ENSG0000(	-0,004772	0,3358495	-2,672599	0,0079154	0,9512444
ENSG0000(	-0,006367	0,1401691	-2,666656	0,0080536	0,9512444
ENSG0000(	-0,002736	-0,043891	-2,659827	0,008215	0,9512444
ENSG0000(	0,0027622	-0,152552	2,6445331	0,0085871	0,9512444

ENSG0000(	-0,016727	-0,568733	-2,642161	0,0086461	0,9512444
ENSG0000(	-0,008211	-0,224945	-2,641156	0,0086712	0,9512444
ENSG0000(	0,0058448	-0,035921	2,6345311	0,0088385	0,9512444
ENSG0000(	-0,002671	0,0069684	-2,62877	0,0089863	0,9512444
ENSG0000(	0,0040711	-0,033853	2,619239	0,0092357	0,9512444
ENSG0000(	0,0051492	-0,368105	2,6129204	0,0094045	0,9512444
ENSG0000(	0,0050708	0,0732172	2,6031486	0,0096709	0,9512444
ENSG0000(	0,0039243	0,1847974	2,6025873	0,0096864	0,9512444
ENSG0000(	-0,00258	0,083193	-2,600661	0,0097398	0,9512444
ENSG0000(	0,0033659	-0,105161	2,5880744	0,0100952	0,9512444
ENSG0000(	0,0092345	-0,324033	2,5875613	0,0101099	0,9512444
ENSG0000(	-0,010027	-0,609068	-2,586077	0,0101526	0,9512444
ENSG0000(	0,0044042	-0,119718	2,5842782	0,0102046	0,9512444
ENSG0000(	0,0032807	0,0261058	2,5784552	0,0103746	0,9512444
ENSG0000(	0,0021233	-0,000934	2,5755499	0,0104603	0,9512444
ENSG0000(	0,0026421	-0,084554	2,5754969	0,0104618	0,9512444
ENSG0000(	0,0045271	-0,012965	2,568342	0,0106757	0,9512444
ENSG0000(	-0,002441	0,0585578	-2,564027	0,0108065	0,9512444
ENSG0000(	-0,010644	0,2833217	-2,555333	0,0110745	0,9512444
ENSG0000(	-0,00546	-0,09611	-2,543174	0,0114593	0,9512444
ENSG0000(	0,0043187	0,0652286	2,5420915	0,0114941	0,9512444
ENSG0000(	0,0052157	0,1639209	2,5397736	0,011569	0,9512444
ENSG0000(	0,0030258	0,0573482	2,5333823	0,0117778	0,9512444
ENSG0000(	-0,007656	-0,398468	-2,531182	0,0118504	0,9512444
ENSG0000(	0,0041335	0,0908761	2,5306695	0,0118674	0,9512444
ENSG0000(	-0,008114	-0,064903	-2,525283	0,0120471	0,9512444
ENSG0000(	0,0046576	-0,220113	2,5148609	0,0124018	0,9512444
ENSG0000(	-0,002601	0,0637376	-2,514608	0,0124105	0,9512444
ENSG0000(	-0,005207	0,5509794	-2,503636	0,0127942	0,9512444
ENSG0000(	-0,00742	-0,294078	-2,500848	0,0128933	0,9512444
ENSG0000(	0,0016727	0,0078261	2,4942413	0,013131	0,9512444
ENSG0000(	-0,006038	0,2216809	-2,489948	0,0132875	0,9512444
ENSG0000(	-0,006484	-0,113914	-2,489594	0,0133005	0,9512444
ENSG0000(	-0,003913	-0,023307	-2,48366	0,0135197	0,9512444
ENSG0000(	-0,002386	-0,095221	-2,477147	0,0137641	0,9512444
ENSG0000(	-0,008179	-0,217648	-2,47511	0,0138413	0,9512444
ENSG0000(	-0,005456	-0,304497	-2,47115	0,0139926	0,9512444
ENSG0000(	0,0084619	-0,062103	2,4694918	0,0140563	0,9512444
ENSG0000(	0,003202	0,2212667	2,4675878	0,0141299	0,9512444
ENSG0000(	0,0054516	0,1555651	2,4660701	0,0141887	0,9512444
ENSG0000(	-0,005037	-0,041201	-2,464158	0,0142632	0,9512444
ENSG0000(	0,008646	0,9012904	2,4611562	0,0143808	0,9512444
ENSG0000(	0,0021613	-0,029934	2,4597271	0,014437	0,9512444
ENSG0000(	-0,010011	-0,203589	-2,459556	0,0144438	0,9512444
ENSG0000(	0,0028877	0,1484133	2,4541144	0,01466	0,9512444
ENSG0000(	0,0037147	0,2294326	2,4498566	0,0148311	0,9512444
ENSG0000(	-0,003058	-0,02308	-2,448802	0,0148738	0,9512444
ENSG0000(	-0,002525	0,0297449	-2,444963	0,01503	0,9512444

ENSG0000(	0,0051699	-0,002403	2,4449245	0,0150316	0,9512444
ENSG0000(	0,0058466	0,1926672	2,4446336	0,0150435	0,9512444
ENSG0000(	0,0047028	-0,140289	2,4434838	0,0150906	0,9512444
ENSG0000(	0,0035011	0,1137446	2,4356599	0,0154146	0,9512444
ENSG0000(	0,0038216	-0,003722	2,4333487	0,0155115	0,9512444
ENSG0000(	-0,003044	0,2375029	-2,432842	0,0155328	0,9512444
ENSG0000(	0,0028637	0,518676	2,4319988	0,0155684	0,9512444
ENSG0000(	-0,003091	0,3491753	-2,427947	0,0157401	0,9512444
ENSG0000(	-0,008098	-0,214978	-2,426539	0,0158002	0,9512444
ENSG0000(	0,0027671	-0,01098	2,4222919	0,0159826	0,9512444
ENSG0000(	-0,003654	-0,290192	-2,421389	0,0160216	0,9512444
ENSG0000(	-0,002869	0,0232175	-2,418603	0,0161425	0,9512444
ENSG0000(	-0,003058	0,0649905	-2,415217	0,0162905	0,9512444
ENSG0000(	0,0030453	-0,197814	2,4150309	0,0162987	0,9512444
ENSG0000(	-0,00289	-0,461463	-2,414409	0,0163261	0,9512444
ENSG0000(	0,0097158	0,1679121	2,4110972	0,0164723	0,9512444
ENSG0000(	-0,009175	0,2687874	-2,408972	0,0165668	0,9512444
ENSG0000(	-0,007041	0,1165269	-2,39952	0,0169927	0,9512444
ENSG0000(	0,001766	0,0523283	2,3994884	0,0169941	0,9512444
ENSG0000(	-0,001944	0,2313529	-2,398632	0,0170331	0,9512444
ENSG0000(	0,0048496	0,1443105	2,384843	0,0176731	0,9512444
ENSG0000(	0,0027213	0,0461011	2,3804712	0,0178804	0,9512444
ENSG0000(	-0,00197	0,0246703	-2,380092	0,0178985	0,9512444
ENSG0000(	0,0050187	0,2353767	2,3787938	0,0179605	0,9512444
ENSG0000(	-0,007357	-0,086886	-2,377257	0,0180342	0,9512444
ENSG0000(	0,0033859	-0,017001	2,3769396	0,0180494	0,9512444
ENSG0000(	0,0062515	0,1409968	2,3705535	0,0183586	0,9512444
ENSG0000(	-0,004258	-0,487532	-2,364537	0,0186541	0,9512444
ENSG0000(	0,0121892	1,1106405	2,3642698	0,0186673	0,9512444
ENSG0000(	0,0036588	0,0784249	2,3608466	0,0188374	0,9512444
ENSG0000(	0,0059597	-0,249304	2,3576423	0,0189979	0,9512444
ENSG0000(	0,0043875	0,121941	2,3575451	0,0190028	0,9512444
ENSG0000(	-0,009665	-0,611809	-2,3572	0,0190201	0,9512444
ENSG0000(	-0,003087	0,0439181	-2,352945	0,0192353	0,9512444
ENSG0000(	-0,005909	0,3196418	-2,352069	0,0192798	0,9512444
ENSG0000(	0,0027093	0,0928496	2,3420227	0,0197974	0,9512444
ENSG0000(	-0,002457	-0,044918	-2,340193	0,019893	0,9512444
ENSG0000(	-0,003698	0,0822574	-2,336646	0,0200794	0,9512444
ENSG0000(	-0,002178	0,0329721	-2,334316	0,0202027	0,9512444
ENSG0000(	0,0028642	0,0004179	2,3337289	0,0202338	0,9512444
ENSG0000(	-0,002619	0,0316698	-2,328675	0,0205039	0,9512444
ENSG0000(	0,0051595	0,1517077	2,3276721	0,0205578	0,9512444
ENSG0000(	0,0044075	-0,077529	2,326286	0,0206326	0,9512444
ENSG0000(	0,0033733	-0,237972	2,326075	0,020644	0,9512444
ENSG0000(	0,0043617	0,1015903	2,3193613	0,0210098	0,9512444
ENSG0000(	-0,002117	0,0292412	-2,314856	0,0212585	0,9512444
ENSG0000(	0,004712	0,3066208	2,3134274	0,0213378	0,9512444
ENSG0000(	0,0049769	0,0624082	2,3073478	0,0216785	0,9512444

ENSG0000(	-0,001899	0,2362961	-2,303398	0,0219025	0,9512444
ENSG0000(	0,0021253	0,2354214	2,2974527	0,0222433	0,9512444
ENSG0000(	0,0049359	-0,102634	2,2965934	0,0222929	0,9512444
ENSG0000(	0,0056856	0,374132	2,2941426	0,022435	0,9512444
ENSG0000(	-0,003654	0,1964471	-2,290995	0,0226187	0,9512444
ENSG0000(	-0,006723	-0,154693	-2,28719	0,0228425	0,9512444
ENSG0000(	-0,003662	0,1085658	-2,285874	0,0229203	0,9512444
ENSG0000(	0,0040905	0,0389714	2,2844258	0,0230063	0,9512444
ENSG0000(	0,006277	-0,266555	2,2836052	0,0230551	0,9512444
ENSG0000(	0,0040132	-0,251096	2,2801078	0,0232642	0,9512444
ENSG0000(	-0,005462	-0,176495	-2,279309	0,0233122	0,9512444
ENSG0000(	-0,001576	-0,030637	-2,278509	0,0233603	0,9512444
ENSG0000(	0,0029854	-0,041772	2,2764632	0,0234838	0,9512444
ENSG0000(	-0,00256	-0,006489	-2,272292	0,0237374	0,9512444
ENSG0000(	-0,004377	-0,086304	-2,269665	0,0238983	0,9512444
ENSG0000(	0,0022959	-0,19994	2,2687306	0,0239558	0,9512444
ENSG0000(	-0,012199	0,4589613	-2,267528	0,02403	0,9512444
ENSG0000(	-0,004184	1,4725094	-2,266259	0,0241084	0,9512444
ENSG0000(	0,0027858	0,0377291	2,2650435	0,0241838	0,9512444
ENSG0000(	0,0084593	-0,491732	2,2643639	0,024226	0,9512444
ENSG0000(	-0,003595	-0,090975	-2,263027	0,0243093	0,9512444
ENSG0000(	0,0056087	-0,312357	2,2619053	0,0243793	0,9512444
ENSG0000(	0,0043102	0,2403515	2,2611885	0,0244242	0,9512444
ENSG0000(	-0,002792	-0,056225	-2,258479	0,0245943	0,9512444
ENSG0000(	0,0033568	-0,076727	2,2518195	0,025017	0,9512444
ENSG0000(	-0,002706	0,0624768	-2,251653	0,0250277	0,9512444
ENSG0000(	0,003028	0,1777568	2,2498137	0,0251455	0,9512444
ENSG0000(	0,0026635	0,1165803	2,2492832	0,0251796	0,9512444
ENSG0000(	0,0045597	0,4320812	2,2457677	0,0254066	0,9512444
ENSG0000(	-0,003852	0,7765107	-2,241662	0,0256739	0,9512444
ENSG0000(	-0,001556	-0,016505	-2,237315	0,0259595	0,9512444
ENSG0000(	-0,007907	1,0590295	-2,236966	0,0259825	0,9512444
ENSG0000(	-0,001594	-0,054184	-2,236563	0,0260092	0,9512444
ENSG0000(	-0,002759	0,0567439	-2,235484	0,0260806	0,9512444
ENSG0000(	-0,004171	-0,04917	-2,232301	0,0262924	0,9512444
ENSG0000(	-0,009368	-0,404322	-2,228173	0,0265692	0,9512444
ENSG0000(	0,0094625	-0,669071	2,2277007	0,0266011	0,9512444
ENSG0000(	-0,008278	-0,280698	-2,221619	0,027014	0,9512444
ENSG0000(	-0,006008	0,3903186	-2,219073	0,0271885	0,9512444
ENSG0000(	0,0061335	0,4480378	2,2190638	0,0271892	0,9512444
ENSG0000(	-0,002391	0,0475367	-2,217558	0,0272929	0,9512444
ENSG0000(	-0,002316	-0,106507	-2,214823	0,027482	0,9512444
ENSG0000(	0,00284	0,8592729	2,2139478	0,0275428	0,9512444
ENSG0000(	-0,010973	-0,372422	-2,20649	0,0280654	0,9512444
ENSG0000(	0,0050812	0,1700535	2,2045272	0,0282044	0,9512444
ENSG0000(	0,0022732	-0,052279	2,2045114	0,0282055	0,9512444
ENSG0000(	0,0051198	-0,257503	2,2018482	0,028395	0,9512444
ENSG0000(	-0,016436	-2,402554	-2,199567	0,0285582	0,9512444

ENSG0000(	-0,002965	0,0524201	-2,198768	0,0286156	0,9512444
ENSG0000(	0,0087112	-0,246842	2,1933196	0,0290094	0,9512444
ENSG0000(	0,0075391	0,2936658	2,1924176	0,029075	0,9512444
ENSG0000(	0,0079112	-0,393926	2,1902399	0,029234	0,9512444
ENSG0000(	0,0030835	-0,014413	2,1888682	0,0293346	0,9512444
ENSG0000(	-0,002194	0,0411921	-2,187514	0,0294341	0,9512444
ENSG0000(	0,006988	0,1162334	2,187414	0,0294415	0,9512444
ENSG0000(	0,0037399	-0,168256	2,1822445	0,0298243	0,9512444
ENSG0000(	-0,003189	-0,169217	-2,181563	0,0298751	0,9512444
ENSG0000(	-0,003807	-0,19403	-2,17937	0,030039	0,9512444
ENSG0000(	0,0048146	0,0735923	2,1771424	0,0302063	0,9512444
ENSG0000(	-0,004837	0,3872611	-2,176029	0,0302902	0,9512444
ENSG0000(	0,0096493	0,7815303	2,175961	0,0302954	0,9512444
ENSG0000(	0,0048328	-0,269941	2,1752179	0,0303515	0,9512444
ENSG0000(	0,0059449	0,2717448	2,1747531	0,0303867	0,9512444
ENSG0000(	-0,003501	0,330784	-2,174062	0,030439	0,9512444
ENSG0000(	-0,002166	0,1139631	-2,17334	0,0304938	0,9512444
ENSG0000(	0,0010442	0,0067732	2,1729853	0,0305207	0,9512444
ENSG0000(	-0,003961	0,2749997	-2,169689	0,030772	0,9512444
ENSG0000(	-0,007039	-0,166892	-2,169221	0,0308078	0,9512444
ENSG0000(	-0,002338	-0,110971	-2,16328	0,0312657	0,9512444
ENSG0000(	0,0043484	0,8867564	2,159367	0,0315704	0,9512444
ENSG0000(	-0,004789	-0,003345	-2,157855	0,0316889	0,9512444
ENSG0000(	-0,00335	-0,137804	-2,155178	0,0318996	0,9512444
ENSG0000(	0,0035062	-0,125427	2,1534019	0,03204	0,9512444
ENSG0000(	-0,003404	0,0741836	-2,152776	0,0320896	0,9512444
ENSG0000(	0,0070509	-0,100707	2,1523684	0,0321219	0,9512444
ENSG0000(	-0,007406	0,7759442	-2,150128	0,0323002	0,9512444
ENSG0000(	0,0032293	-0,153974	2,1492327	0,0323717	0,9512444
ENSG0000(	-0,004622	0,3930971	-2,146063	0,0326258	0,9512444
ENSG0000(	0,005284	0,1629918	2,1440431	0,0327887	0,9512444
ENSG0000(	-0,005331	0,1079091	-2,143714	0,0328153	0,9512444
ENSG0000(	0,003128	0,7238295	2,1427816	0,0328908	0,9512444
ENSG0000(	0,0016004	-0,111015	2,1414692	0,0329973	0,9512444
ENSG0000(	-0,003929	0,2072343	-2,135538	0,0334822	0,9512444
ENSG0000(	0,0042096	0,233198	2,1354401	0,0334902	0,9512444
ENSG0000(	0,003743	0,478199	2,1353955	0,0334939	0,9512444
ENSG0000(	0,0019306	0,1674506	2,1348579	0,0335382	0,9512444
ENSG0000(	0,0046995	-0,331981	2,133281	0,0336683	0,9512444
ENSG0000(	-0,00618	-0,334375	-2,132108	0,0337654	0,9512444
ENSG0000(	-0,002975	-0,295113	-2,129796	0,0339574	0,9512444
ENSG0000(	0,0066425	0,7152882	2,1295675	0,0339765	0,9512444
ENSG0000(	-0,008475	0,6807191	-2,125487	0,0343179	0,9512444
ENSG0000(	0,0038024	-0,092723	2,1252232	0,0343401	0,9512444
ENSG0000(	0,0082691	-0,550201	2,1250043	0,0343585	0,9512444
ENSG0000(	-0,003152	-0,181152	-2,124369	0,0344119	0,9512444
ENSG0000(	0,0029675	-0,145602	2,1201742	0,0347668	0,9570335
ENSG0000(	0,0025959	0,0027006	2,116199	0,035106	0,9623442

ENSG0000(	-0,001884	-0,03334	-2,107786	0,0358333	0,9679528
ENSG0000(	0,0089599	0,1140736	2,1068493	0,035915	0,9679528
ENSG0000(	-0,001903	-0,008537	-2,105474	0,0360354	0,9679528
ENSG0000(	0,005163	0,0610184	2,1044233	0,0361275	0,9679528
ENSG0000(	0,0099024	-0,073878	2,1022514	0,0363187	0,9679528
ENSG0000(	0,0089782	-0,238114	2,0996036	0,0365529	0,9679528
ENSG0000(	0,0066811	0,187741	2,0978306	0,0367105	0,9679528
ENSG0000(	0,0048683	0,0975636	2,0971216	0,0367736	0,9679528
ENSG0000(	-0,001896	-0,024305	-2,096177	0,036858	0,9679528
ENSG0000(	-0,002373	-0,098456	-2,094131	0,0370411	0,9679528
ENSG0000(	0,002178	-0,053725	2,0910222	0,0373209	0,9679528
ENSG0000(	-0,002481	-0,060465	-2,090355	0,0373812	0,9679528
ENSG0000(	-0,005281	0,2315068	-2,089429	0,037465	0,9679528
ENSG0000(	0,0048693	-0,09444	2,0878132	0,0376116	0,9679528
ENSG0000(	-0,002716	-0,015378	-2,086709	0,0377121	0,9679528
ENSG0000(	-0,002778	0,0422653	-2,085585	0,0378146	0,9679528
ENSG0000(	0,0065593	-0,253233	2,0851686	0,0378527	0,9679528
ENSG0000(	0,0017348	-0,012152	2,0811072	0,0382254	0,9679528
ENSG0000(	0,005589	0,4721543	2,0772718	0,0385803	0,9679528
ENSG0000(	-0,003836	0,1771069	-2,077068	0,0385992	0,9679528
ENSG0000(	-0,002774	1,0668816	-2,075061	0,0387861	0,9679528
ENSG0000(	-0,00223	-0,029306	-2,075015	0,0387904	0,9679528
ENSG0000(	0,0088233	0,3990088	2,0746575	0,0388238	0,9679528
ENSG0000(	0,0019588	0,0067833	2,0744658	0,0388417	0,9679528
ENSG0000(	0,0034329	0,21646	2,069768	0,0392827	0,9703474
ENSG0000(	0,0031316	0,0369582	2,0683582	0,0394159	0,9703474
ENSG0000(	0,0037872	-0,162519	2,0629368	0,0399317	0,9703474
ENSG0000(	-0,003881	-0,053478	-2,062596	0,0399643	0,9703474
ENSG0000(	0,0038042	0,1432393	2,0607656	0,0401398	0,9703474
ENSG0000(	-0,001968	0,0018536	-2,060667	0,0401493	0,9703474
ENSG0000(	-0,003263	0,1584812	-2,060583	0,0401574	0,9703474
ENSG0000(	0,0094431	-0,569408	2,0574719	0,0404574	0,9703474
ENSG0000(	-0,001821	0,1233847	-2,057365	0,0404677	0,9703474
ENSG0000(	-0,002822	0,0839523	-2,05536	0,0406621	0,9703474
ENSG0000(	-0,003439	-0,383944	-2,051991	0,0409905	0,9703474
ENSG0000(	-0,004674	-0,138627	-2,050491	0,0411374	0,9703474
ENSG0000(	0,0048943	0,1853563	2,0484938	0,0413338	0,9703474
ENSG0000(	-0,002852	-0,285318	-2,046403	0,0415402	0,9703474
ENSG0000(	-0,010886	0,7553576	-2,040306	0,0421472	0,9703474
ENSG0000(	0,0016262	-0,264593	2,0396763	0,0422103	0,9703474
ENSG0000(	-0,004595	-0,11761	-2,039424	0,0422356	0,9703474
ENSG0000(	-0,004842	0,1488034	-2,037259	0,0424533	0,9703474
ENSG0000(	-0,003017	0,3906828	-2,035811	0,0425994	0,9703474
ENSG0000(	-0,003363	0,238945	-2,035334	0,0426476	0,9703474
ENSG0000(	-0,009397	0,0128087	-2,032865	0,0428981	0,9703474
ENSG0000(	-0,002292	0,0211027	-2,032854	0,0428992	0,9703474
ENSG0000(	-0,003122	-0,202992	-2,031356	0,0430517	0,9703474
ENSG0000(	0,0048118	-0,313155	2,0303552	0,0431539	0,9703474

ENSG0000(	0,0035731	0,2410255	2,0275239	0,043444	0,9703474
ENSG0000(	0,0023809	0,1245763	2,0269197	0,0435062	0,9703474
ENSG0000(	0,0046953	0,3643245	2,0263163	0,0435683	0,9703474
ENSG0000(	-0,004738	-0,20532	-2,025837	0,0436177	0,9703474
ENSG0000(	0,0100459	0,6294721	2,0258334	0,0436181	0,9703474
ENSG0000(	0,0043788	-0,217802	2,0247206	0,0437329	0,9703474
ENSG0000(	-0,002651	0,4449681	-2,024609	0,0437445	0,9703474
ENSG0000(	0,002563	0,0896029	2,0245962	0,0437458	0,9703474
ENSG0000(	-0,00399	-0,106103	-2,024024	0,043805	0,9703474
ENSG0000(	-0,004222	-0,062699	-2,021125	0,0441059	0,9737338
ENSG0000(	-0,005763	-0,045837	-2,012444	0,0450175	0,9888604
ENSG0000(	0,003382	-0,131241	2,0109141	0,0451798	0,9888604
ENSG0000(	0,0069676	0,2716367	2,0103288	0,045242	0,9888604
ENSG0000(	-0,004116	0,5063487	-2,00522	0,0457881	0,9888748
ENSG0000(	0,0028832	-0,056815	2,0022062	0,046113	0,9888748
ENSG0000(	0,0053887	0,1807174	1,9971581	0,0466614	0,9888748
ENSG0000(	0,0037888	-0,009915	1,9968745	0,0466924	0,9888748
ENSG0000(	0,0042246	-0,386845	1,9934198	0,0470711	0,9888748
ENSG0000(	-0,004231	0,4830289	-1,991917	0,0472366	0,9888748
ENSG0000(	0,0045978	0,1281808	1,9916528	0,0472658	0,9888748
ENSG0000(	-0,003431	0,2108524	-1,990727	0,0473681	0,9888748
ENSG0000(	-0,004685	0,6936023	-1,987813	0,0476912	0,9888748
ENSG0000(	-0,002173	0,157969	-1,986557	0,0478311	0,9888748
ENSG0000(	0,0047788	0,1447665	1,9851622	0,0479868	0,9888748
ENSG0000(	0,002465	0,0724013	1,9831263	0,0482149	0,9888748
ENSG0000(	0,0034273	-0,080209	1,981743	0,0483704	0,9888748
ENSG0000(	-0,00466	0,5301915	-1,981246	0,0484263	0,9888748
ENSG0000(	-0,002207	0,0218934	-1,981177	0,0484341	0,9888748
ENSG0000(	-0,004563	-0,051047	-1,978376	0,0487506	0,9888748
ENSG0000(	-0,007782	-0,249345	-1,978042	0,0487885	0,9888748
ENSG0000(	-0,003587	-0,014649	-1,975321	0,0490978	0,9888748
ENSG0000(	-0,004564	-0,094045	-1,975189	0,0491127	0,9888748
ENSG0000(	0,0011254	0,1342947	1,9748744	0,0491487	0,9888748
ENSG0000(	-0,002368	0,1332695	-1,974069	0,0492406	0,9888748
ENSG0000(	-0,004167	0,1399335	-1,971293	0,0495587	0,9888748
ENSG0000(	-0,002151	-0,233526	-1,970482	0,049652	0,9888748
ENSG0000(	-0,008248	-1,36829	-1,968651	0,049863	0,9888748
ENSG0000(	0,0031712	-0,07715	1,9681362	0,0499224	0,9888748

geneID	logFC	AveExpr	t	P.Value	adj.P.Val
ENSG0000	0,007447	-0,26552	3,862047	0,000137	0,797999
ENSG0000	0,002776	0,041767	3,53209	0,000475	0,797999
ENSG0000	-0,008561	-0,142115	-3,455654	0,000625	0,797999
ENSG0000	0,005116	-0,246029	3,412255	0,000729	0,797999
ENSG0000	-0,005827	0,206865	-3,323629	0,000995	0,797999
ENSG0000	-0,004791	-0,255457	-3,315669	0,001022	0,797999
ENSG0000	-0,002611	0,114419	-3,20185	0,001507	0,797999
ENSG0000	0,002169	-0,034241	3,198948	0,001521	0,797999
ENSG0000	-0,007287	0,178368	-3,192995	0,001552	0,797999
ENSG0000	-0,0027	0,02619	-3,184158	0,001599	0,797999
ENSG0000	-0,003069	-0,101266	-3,141188	0,001844	0,797999
ENSG0000	-0,01462	-0,012685	-3,09823	0,002124	0,797999
ENSG0000	0,00845	0,511053	3,096681	0,002135	0,797999
ENSG0000	-0,002152	-0,037161	-3,091322	0,002173	0,797999
ENSG0000	-0,003591	0,081651	-3,088356	0,002194	0,797999
ENSG0000	-0,004345	-0,082859	-3,082511	0,002236	0,797999
ENSG0000	0,004013	0,116597	3,059061	0,002413	0,797999
ENSG0000	0,00651	-0,258181	3,039292	0,002572	0,797999
ENSG0000	-0,005065	0,077631	-3,032164	0,002632	0,797999
ENSG0000	0,005135	0,102511	3,025552	0,002689	0,797999
ENSG0000	-0,004927	-0,568903	-3,008133	0,002843	0,797999
ENSG0000	-0,002751	0,086049	-2,978737	0,003122	0,797999
ENSG0000	0,007631	-0,22583	2,973326	0,003176	0,797999
ENSG0000	0,004531	0,305219	2,958645	0,003327	0,797999
ENSG0000	-0,001698	-0,077165	-2,958621	0,003327	0,797999
ENSG0000	0,004225	-0,026409	2,937573	0,003555	0,797999
ENSG0000	0,00343	0,222784	2,928855	0,003653	0,797999
ENSG0000	0,005779	0,227879	2,926886	0,003676	0,797999
ENSG0000	0,009351	-0,037848	2,908609	0,003892	0,797999
ENSG0000	0,009919	0,304682	2,905458	0,00393	0,797999
ENSG0000	-0,001996	0,258474	-2,904657	0,00394	0,797999
ENSG0000	0,001603	-0,071941	2,894648	0,004064	0,797999
ENSG0000	0,003005	0,02105	2,886457	0,004168	0,797999
ENSG0000	0,004036	-0,166016	2,882836	0,004215	0,797999
ENSG0000	0,004979	-0,35652	2,874234	0,004329	0,797999
ENSG0000	0,005877	-0,723037	2,862645	0,004486	0,797999
ENSG0000	-0,005694	0,205863	-2,860645	0,004514	0,797999
ENSG0000	-0,002983	0,042892	-2,853621	0,004612	0,797999
ENSG0000	-0,004853	-0,267937	-2,850392	0,004658	0,797999
ENSG0000	0,008982	-0,639703	2,847694	0,004697	0,797999
ENSG0000	0,002451	-0,017221	2,845343	0,00473	0,797999
ENSG0000	-0,004475	0,212177	-2,830343	0,004952	0,797999
ENSG0000	0,002528	-0,007514	2,826965	0,005003	0,797999
ENSG0000	0,005212	0,225559	2,821987	0,00508	0,797999
ENSG0000	-0,004741	-0,327465	-2,821387	0,005089	0,797999
ENSG0000	0,006005	-0,319965	2,818971	0,005126	0,797999
ENSG0000	-0,002513	0,203577	-2,812268	0,005232	0,797999
ENSG0000	0,010358	-0,140689	2,80591	0,005333	0,797999

ENSG0000	-0,005482	0,110258	-2,802575	0,005387	0,797999
ENSG0000	-0,004471	0,489109	-2,798331	0,005457	0,797999
ENSG0000	-0,002044	0,126429	-2,787938	0,005631	0,797999
ENSG0000	0,00292	-0,181956	2,786531	0,005655	0,797999
ENSG0000	-0,008774	-1,313865	-2,775649	0,005842	0,797999
ENSG0000	0,004095	0,245198	2,775087	0,005852	0,797999
ENSG0000	-0,002307	0,095161	-2,772404	0,0059	0,797999
ENSG0000	0,005562	-0,380417	2,75568	0,006202	0,797999
ENSG0000	0,001698	-0,057958	2,750132	0,006305	0,797999
ENSG0000	0,001697	0,053631	2,741776	0,006464	0,797999
ENSG0000	-0,003974	-0,317068	-2,738018	0,006537	0,797999
ENSG0000	-0,003148	0,124588	-2,736499	0,006566	0,797999
ENSG0000	0,005648	0,220626	2,736158	0,006573	0,797999
ENSG0000	-0,002574	0,05944	-2,727479	0,006744	0,797999
ENSG0000	-0,002294	-0,121114	-2,725523	0,006783	0,797999
ENSG0000	0,005801	0,069392	2,724788	0,006798	0,797999
ENSG0000	-0,001276	0,058139	-2,720739	0,00688	0,797999
ENSG0000	-0,00294	-0,011814	-2,720079	0,006893	0,797999
ENSG0000	0,006564	-0,190595	2,717963	0,006936	0,797999
ENSG0000	-0,001914	0,096091	-2,714918	0,006999	0,797999
ENSG0000	0,004157	-0,032099	2,712176	0,007056	0,797999
ENSG0000	-0,00312	-0,041071	-2,7072	0,00716	0,797999
ENSG0000	-0,001518	-0,072656	-2,704275	0,007222	0,797999
ENSG0000	-0,010797	0,418726	-2,702158	0,007267	0,797999
ENSG0000	-0,005343	0,329454	-2,701469	0,007282	0,797999
ENSG0000	0,002687	-0,114303	2,698519	0,007345	0,797999
ENSG0000	-0,003034	-0,208561	-2,696201	0,007395	0,797999
ENSG0000	-0,001641	-0,134666	-2,692676	0,007472	0,797999
ENSG0000	-0,006557	-0,148673	-2,689795	0,007535	0,797999
ENSG0000	0,004228	0,166875	2,68808	0,007573	0,797999
ENSG0000	-0,009812	0,651691	-2,685128	0,007639	0,797999
ENSG0000	0,003536	0,429193	2,679741	0,00776	0,800523
ENSG0000	0,006244	0,266018	2,67141	0,007951	0,805608
ENSG0000	-0,005077	-0,474423	-2,669094	0,008004	0,805608
ENSG0000	-0,008753	0,441698	-2,661538	0,008182	0,811467
ENSG0000	0,002997	-0,153592	2,658304	0,008259	0,811467
ENSG0000	-0,003665	0,449266	-2,641839	0,008662	0,819394
ENSG0000	0,003773	0,171687	2,637909	0,008761	0,819394
ENSG0000	-0,004722	0,27723	-2,636098	0,008807	0,819394
ENSG0000	0,00377	0,259442	2,635807	0,008814	0,819394
ENSG0000	-0,002907	-0,014924	-2,62964	0,008972	0,819394
ENSG0000	-0,003695	-0,153976	-2,627256	0,009034	0,819394
ENSG0000	-0,004653	0,459077	-2,623736	0,009126	0,819394
ENSG0000	-0,004904	0,213135	-2,61791	0,009279	0,819394
ENSG0000	-0,003632	0,077923	-2,61391	0,009386	0,819394
ENSG0000	-0,006058	-0,089501	-2,613511	0,009397	0,819394
ENSG0000	-0,005008	-0,405824	-2,61081	0,00947	0,819394
ENSG0000	0,003193	-0,157202	2,607008	0,009573	0,819394

ENSG0000	0,006884	0,278231	2,602431	0,009699	0,819394
ENSG0000	0,001587	0,186529	2,599197	0,009789	0,819394
ENSG0000	-0,008728	-0,771923	-2,597766	0,009829	0,819394
ENSG0000	-0,003274	-0,016486	-2,592227	0,009985	0,820478
ENSG0000	0,001549	-0,301508	2,590273	0,010041	0,820478
ENSG0000	0,002322	0,14007	2,583818	0,010227	0,827464
ENSG0000	-0,002225	0,097574	-2,554875	0,011098	0,868739
ENSG0000	-0,003762	0,182459	-2,551602	0,011201	0,868739
ENSG0000	-0,003857	0,400204	-2,548888	0,011286	0,868739
ENSG0000	0,006431	0,095824	2,547671	0,011325	0,868739
ENSG0000	0,010164	0,079391	2,544306	0,011432	0,868739
ENSG0000	0,007068	0,488733	2,537185	0,011663	0,868739
ENSG0000	0,003784	0,050971	2,535271	0,011725	0,868739
ENSG0000	-0,009202	-0,172473	-2,533661	0,011778	0,868739
ENSG0000	-0,00116	0,072754	-2,531792	0,01184	0,868739
ENSG0000	0,00326	0,008236	2,528701	0,011942	0,868739
ENSG0000	0,003349	0,147597	2,525367	0,012054	0,868739
ENSG0000	0,004086	-0,027646	2,513331	0,012464	0,868739
ENSG0000	-0,002588	0,004904	-2,510959	0,012547	0,868739
ENSG0000	-0,003132	0,042674	-2,507532	0,012667	0,868739
ENSG0000	0,003482	0,021628	2,507313	0,012674	0,868739
ENSG0000	-0,010656	-1,062928	-2,505649	0,012733	0,868739
ENSG0000	0,003253	0,130219	2,50514	0,012751	0,868739
ENSG0000	0,006563	0,159909	2,500094	0,01293	0,868739
ENSG0000	0,003424	-0,131423	2,498565	0,012985	0,868739
ENSG0000	0,002644	0,003204	2,495193	0,013106	0,868739
ENSG0000	0,001547	0,031743	2,491349	0,013246	0,868739
ENSG0000	0,002071	-0,093228	2,490667	0,013271	0,868739
ENSG0000	-0,004322	-0,121975	-2,484682	0,013492	0,868739
ENSG0000	0,001356	0,068023	2,48331	0,013543	0,868739
ENSG0000	-0,002501	0,369429	-2,483026	0,013554	0,868739
ENSG0000	0,004325	-0,150401	2,479049	0,013703	0,868739
ENSG0000	0,003731	0,27308	2,478588	0,01372	0,868739
ENSG0000	0,005695	-0,327512	2,477714	0,013753	0,868739
ENSG0000	0,001003	-0,029142	2,476746	0,01379	0,868739
ENSG0000	0,008998	-0,644659	2,467589	0,01414	0,872666
ENSG0000	-0,002453	0,155728	-2,463515	0,014299	0,872666
ENSG0000	-0,001323	-0,118387	-2,462348	0,014344	0,872666
ENSG0000	0,00265	0,186198	2,461509	0,014377	0,872666
ENSG0000	0,004455	-0,144732	2,457324	0,014543	0,872666
ENSG0000	0,002462	-0,032185	2,456718	0,014567	0,872666
ENSG0000	0,003341	-0,024952	2,455569	0,014612	0,872666
ENSG0000	0,006771	0,297828	2,452591	0,014732	0,872666
ENSG0000	0,005239	0,052925	2,449859	0,014842	0,872666
ENSG0000	0,003285	-0,135043	2,44819	0,014909	0,872666
ENSG0000	0,004439	0,144865	2,444761	0,015049	0,873662
ENSG0000	0,001822	0,062006	2,438057	0,015325	0,873662
ENSG0000	-0,005713	-0,483379	-2,436032	0,01541	0,873662

ENSG0000	0,01051	0,767973	2,434518	0,015473	0,873662
ENSG0000	-0,002138	-0,092865	-2,432245	0,015569	0,873662
ENSG0000	0,00305	0,196675	2,428051	0,015747	0,873662
ENSG0000	-0,002436	-0,154	-2,426306	0,015821	0,873662
ENSG0000	-0,004584	0,388053	-2,420046	0,016091	0,873662
ENSG0000	0,004266	0,077784	2,415181	0,016303	0,873662
ENSG0000	0,003259	0,01548	2,410262	0,01652	0,873662
ENSG0000	0,003964	0,113769	2,408885	0,016582	0,873662
ENSG0000	0,00271	0,081165	2,405434	0,016736	0,873662
ENSG0000	0,005878	-0,688659	2,405249	0,016745	0,873662
ENSG0000	0,003401	-0,189729	2,403656	0,016816	0,873662
ENSG0000	0,003246	0,379051	2,401946	0,016894	0,873662
ENSG0000	-0,004588	-0,101362	-2,400604	0,016955	0,873662
ENSG0000	0,00283	-0,053604	2,399474	0,017006	0,873662
ENSG0000	0,003502	-0,001892	2,396407	0,017146	0,873662
ENSG0000	0,006812	0,173571	2,396	0,017165	0,873662
ENSG0000	0,001557	-0,096967	2,391165	0,017389	0,873662
ENSG0000	-0,002917	0,113824	-2,389869	0,017449	0,873662
ENSG0000	-0,002409	0,012416	-2,388427	0,017516	0,873662
ENSG0000	-0,007997	0,697695	-2,386602	0,017602	0,873662
ENSG0000	-0,003468	-0,004716	-2,375025	0,018153	0,873662
ENSG0000	-0,010032	-0,577094	-2,37493	0,018158	0,873662
ENSG0000	0,003555	0,353151	2,372102	0,018295	0,873662
ENSG0000	0,008099	0,22745	2,369647	0,018415	0,873662
ENSG0000	-0,001786	-0,063568	-2,368696	0,018461	0,873662
ENSG0000	0,003105	0,04401	2,368503	0,018471	0,873662
ENSG0000	-0,005174	-0,326249	-2,366625	0,018563	0,873662
ENSG0000	0,00349	-0,25479	2,365735	0,018607	0,873662
ENSG0000	-0,004483	-0,156707	-2,362995	0,018742	0,873662
ENSG0000	0,001703	-0,337202	2,362147	0,018784	0,873662
ENSG0000	-0,001417	-0,089557	-2,361503	0,018817	0,873662
ENSG0000	0,002684	-0,037801	2,358652	0,018959	0,873662
ENSG0000	-0,003799	0,521157	-2,356684	0,019058	0,873662
ENSG0000	0,005023	0,02764	2,355474	0,019119	0,873662
ENSG0000	-0,002825	0,167587	-2,354358	0,019176	0,873662
ENSG0000	0,008523	-0,008109	2,35412	0,019188	0,873662
ENSG0000	0,007075	-0,168759	2,352563	0,019267	0,873662
ENSG0000	-0,006762	0,482177	-2,347685	0,019516	0,873662
ENSG0000	-0,004737	0,502107	-2,347431	0,019529	0,873662
ENSG0000	-0,002459	0,09972	-2,345461	0,019631	0,873662
ENSG0000	0,005802	0,061347	2,342656	0,019777	0,873662
ENSG0000	0,003441	-0,146301	2,340983	0,019864	0,873662
ENSG0000	0,006562	0,758324	2,339284	0,019953	0,873662
ENSG0000	-0,002417	-0,062753	-2,338736	0,019982	0,873662
ENSG0000	0,002023	0,131197	2,338241	0,020008	0,873662
ENSG0000	-0,001796	0,029265	-2,334465	0,020207	0,877726
ENSG0000	0,002224	0,203998	2,326514	0,020633	0,884378
ENSG0000	0,006808	0,128194	2,326301	0,020644	0,884378

ENSG0000	-0,003696	-0,252034	-2,325609	0,020682	0,884378
ENSG0000	0,006824	0,304554	2,322779	0,020835	0,886358
ENSG0000	-0,005758	-0,351994	-2,318326	0,021079	0,88946
ENSG0000	-0,006476	0,360641	-2,317011	0,021152	0,88946
ENSG0000	-0,00243	0,049703	-2,315567	0,021232	0,88946
ENSG0000	0,00161	-0,031076	2,313224	0,021362	0,890392
ENSG0000	-0,00285	0,031839	-2,305853	0,021776	0,897706
ENSG0000	-0,003393	0,216564	-2,303042	0,021935	0,897706
ENSG0000	-0,002192	0,056065	-2,299408	0,022143	0,897706
ENSG0000	-0,00114	-0,066633	-2,29922	0,022154	0,897706
ENSG0000	-0,001348	-0,013107	-2,2988	0,022178	0,897706
ENSG0000	-0,002063	0,118974	-2,298602	0,02219	0,897706
ENSG0000	-0,003137	-0,142795	-2,27846	0,023376	0,90628
ENSG0000	-0,003346	0,006284	-2,27667	0,023484	0,90628
ENSG0000	0,00296	-0,174939	2,27381	0,023658	0,90628
ENSG0000	-0,006258	0,330071	-2,269807	0,023903	0,90628
ENSG0000	0,006404	0,089659	2,265281	0,024182	0,90628
ENSG0000	0,003083	0,369442	2,264979	0,024201	0,90628
ENSG0000	-0,002846	-0,215367	-2,264528	0,024229	0,90628
ENSG0000	0,001891	-0,077643	2,261491	0,024418	0,90628
ENSG0000	0,005115	0,23689	2,259397	0,02455	0,90628
ENSG0000	0,006527	-0,344715	2,258416	0,024612	0,90628
ENSG0000	-0,008146	-0,276192	-2,258371	0,024614	0,90628
ENSG0000	-0,004682	0,303363	-2,254244	0,024876	0,90628
ENSG0000	-0,006003	0,163066	-2,25216	0,025009	0,90628
ENSG0000	-0,004564	0,042713	-2,250102	0,02514	0,90628
ENSG0000	-0,002575	0,031356	-2,249862	0,025156	0,90628
ENSG0000	-0,001765	0,093213	-2,249472	0,025181	0,90628
ENSG0000	0,002276	0,004889	2,24938	0,025187	0,90628
ENSG0000	-0,008015	0,333487	-2,247377	0,025316	0,90628
ENSG0000	0,005662	0,50117	2,244698	0,025489	0,90628
ENSG0000	-0,005785	-0,331166	-2,242305	0,025645	0,90628
ENSG0000	-0,001801	-0,063434	-2,24137	0,025706	0,90628
ENSG0000	0,002351	0,069801	2,240016	0,025795	0,90628
ENSG0000	0,002009	0,024972	2,239227	0,025847	0,90628
ENSG0000	-0,001958	0,166	-2,237405	0,025967	0,90628
ENSG0000	0,006674	-0,039926	2,236557	0,026023	0,90628
ENSG0000	0,004635	-0,619632	2,236092	0,026054	0,90628
ENSG0000	0,001157	-0,003023	2,232398	0,0263	0,90628
ENSG0000	0,001582	-0,148059	2,231038	0,026391	0,90628
ENSG0000	0,004005	0,306278	2,228418	0,026566	0,90628
ENSG0000	0,002857	0,105011	2,227903	0,026601	0,90628
ENSG0000	-0,00628	-0,033276	-2,227645	0,026619	0,90628
ENSG0000	-0,006341	-0,090012	-2,227332	0,02664	0,90628
ENSG0000	0,004738	0,025548	2,225027	0,026796	0,90628
ENSG0000	-0,006282	0,061171	-2,22358	0,026894	0,90628
ENSG0000	0,003455	0,164266	2,222579	0,026962	0,90628
ENSG0000	-0,001332	0,012201	-2,221476	0,027038	0,90628

ENSG0000	-0,002622	0,015784	-2,220848	0,027081	0,90628
ENSG0000	0,005342	-0,143396	2,21415	0,027543	0,90628
ENSG0000	0,002153	0,05274	2,212496	0,027658	0,90628
ENSG0000	-0,003235	0,355349	-2,208943	0,027907	0,90628
ENSG0000	-0,005547	-0,608859	-2,208231	0,027957	0,90628
ENSG0000	-0,002497	-0,071503	-2,207549	0,028005	0,90628
ENSG0000	0,001522	0,173806	2,206984	0,028045	0,90628
ENSG0000	-0,002947	0,257939	-2,206667	0,028067	0,90628
ENSG0000	0,00518	1,020508	2,206006	0,028114	0,90628
ENSG0000	-0,004156	0,273785	-2,203923	0,028261	0,90628
ENSG0000	0,009899	-1,117361	2,202954	0,02833	0,90628
ENSG0000	-0,006776	-0,499186	-2,201987	0,028399	0,90628
ENSG0000	0,003217	0,24581	2,198263	0,028666	0,90628
ENSG0000	-0,00306	-0,091307	-2,197064	0,028752	0,90628
ENSG0000	-0,007269	-0,176973	-2,194235	0,028957	0,90628
ENSG0000	-0,00263	0,005328	-2,19415	0,028963	0,90628
ENSG0000	0,002414	-0,041009	2,191708	0,029141	0,90628
ENSG0000	0,001733	-0,001486	2,190315	0,029243	0,90628
ENSG0000	-0,003864	-0,473485	-2,189624	0,029293	0,90628
ENSG0000	-0,001643	0,127311	-2,189601	0,029295	0,90628
ENSG0000	0,001123	0,08248	2,189548	0,029299	0,90628
ENSG0000	0,001509	-0,103549	2,188401	0,029383	0,90628
ENSG0000	-0,003018	0,099382	-2,187858	0,029423	0,90628
ENSG0000	-0,007293	0,140616	-2,185798	0,029575	0,90628
ENSG0000	-0,004001	0,003017	-2,185319	0,02961	0,90628
ENSG0000	-0,005063	-0,074534	-2,184641	0,029661	0,90628
ENSG0000	-0,003365	-0,040192	-2,184168	0,029696	0,90628
ENSG0000	-0,006811	0,375285	-2,183964	0,029711	0,90628
ENSG0000	0,003499	-1,30753	2,183903	0,029715	0,90628
ENSG0000	-0,005538	0,094821	-2,182033	0,029854	0,90628
ENSG0000	0,003407	-0,423762	2,179837	0,030018	0,90628
ENSG0000	-0,002961	0,1409	-2,179701	0,030029	0,90628
ENSG0000	-0,00692	0,167362	-2,179548	0,03004	0,90628
ENSG0000	0,000964	-0,163024	2,178901	0,030089	0,90628
ENSG0000	0,003776	-0,118527	2,176655	0,030257	0,908052
ENSG0000	-0,002452	0,100379	-2,174675	0,030407	0,909236
ENSG0000	-0,003934	-0,163356	-2,171726	0,030631	0,912625
ENSG0000	0,00318	0,023467	2,161874	0,03139	0,918442
ENSG0000	0,002854	-0,256924	2,161193	0,031443	0,918442
ENSG0000	-0,004116	0,136119	-2,159924	0,031542	0,918442
ENSG0000	-0,005564	-0,764898	-2,157211	0,031754	0,918442
ENSG0000	-0,009673	-0,690401	-2,1561	0,031842	0,918442
ENSG0000	-0,007586	0,519187	-2,155916	0,031856	0,918442
ENSG0000	-0,005318	-0,478779	-2,154672	0,031954	0,918442
ENSG0000	0,004543	-0,117771	2,153247	0,032067	0,918442
ENSG0000	0,003119	0,50561	2,150203	0,032309	0,918442
ENSG0000	-0,009326	0,459993	-2,149862	0,032336	0,918442
ENSG0000	0,001656	0,068816	2,149061	0,0324	0,918442

ENSG0000	-0,002361	-0,020437	-2,14805	0,032481	0,918442
ENSG0000	-0,002429	0,057648	-2,14787	0,032496	0,918442
ENSG0000	0,002077	0,106763	2,146931	0,032571	0,918442
ENSG0000	-0,006287	-0,222597	-2,145719	0,032668	0,918442
ENSG0000	0,002755	-0,217436	2,144058	0,032802	0,918442
ENSG0000	0,001982	-0,356973	2,143218	0,03287	0,918442
ENSG0000	-0,004387	-0,348014	-2,142031	0,032967	0,918442
ENSG0000	-0,003196	0,413662	-2,141005	0,03305	0,918442
ENSG0000	-0,003907	0,54577	-2,13599	0,03346	0,918442
ENSG0000	-0,002473	-0,041016	-2,135497	0,033501	0,918442
ENSG0000	-0,00089	0,068027	-2,13502	0,03354	0,918442
ENSG0000	-0,004856	-0,108927	-2,133621	0,033655	0,918442
ENSG0000	0,003896	0,549459	2,132018	0,033788	0,918442
ENSG0000	-0,003332	-0,353363	-2,131478	0,033833	0,918442
ENSG0000	0,004363	0,306014	2,128248	0,034102	0,918442
ENSG0000	0,001251	-0,09527	2,127994	0,034123	0,918442
ENSG0000	-0,003709	0,255773	-2,125804	0,034306	0,918442
ENSG0000	-0,003661	-0,023305	-2,125732	0,034312	0,918442
ENSG0000	0,001259	0,041247	2,125159	0,034361	0,918442
ENSG0000	-0,002148	-0,11833	-2,123498	0,0345	0,918442
ENSG0000	0,002841	0,532607	2,121783	0,034645	0,918442
ENSG0000	0,002811	-0,067481	2,121084	0,034705	0,918442
ENSG0000	-0,006989	-0,949884	-2,12095	0,034716	0,918442
ENSG0000	0,000664	0,07911	2,12089	0,034721	0,918442
ENSG0000	0,002968	-0,083702	2,117237	0,035032	0,921017
ENSG0000	0,003513	0,25434	2,11589	0,035148	0,921017
ENSG0000	-0,004652	0,144957	-2,114969	0,035227	0,921017
ENSG0000	-0,003997	0,024002	-2,114281	0,035286	0,921017
ENSG0000	0,004565	-0,618279	2,112007	0,035482	0,921017
ENSG0000	0,008344	-0,653022	2,109578	0,035693	0,921017
ENSG0000	0,002008	-0,000928	2,109478	0,035701	0,921017
ENSG0000	-0,003282	0,122557	-2,108791	0,035761	0,921017
ENSG0000	0,005669	0,148026	2,104556	0,036131	0,921017
ENSG0000	0,004797	-0,223278	2,100597	0,03648	0,921017
ENSG0000	0,005527	-0,256442	2,100586	0,036481	0,921017
ENSG0000	-0,001877	-0,100739	-2,095894	0,036899	0,921017
ENSG0000	0,005105	-0,190005	2,094907	0,036987	0,921017
ENSG0000	0,003394	-0,069966	2,094564	0,037018	0,921017
ENSG0000	-0,000423	0,001971	-2,093504	0,037113	0,921017
ENSG0000	0,003981	0,003233	2,09145	0,037298	0,921017
ENSG0000	0,002948	0,182558	2,088419	0,037572	0,921017
ENSG0000	0,004815	0,320105	2,087949	0,037615	0,921017
ENSG0000	-0,004119	0,3852	-2,085909	0,037801	0,921017
ENSG0000	-0,001545	0,046468	-2,081793	0,038178	0,921017
ENSG0000	-0,006857	0,164201	-2,081074	0,038244	0,921017
ENSG0000	0,002636	-0,201181	2,078225	0,038507	0,921017
ENSG0000	-0,001284	0,008356	-2,077046	0,038617	0,921017
ENSG0000	-0,002044	0,177009	-2,074763	0,03883	0,921017

ENSG0000	0,004543	0,125532	2,073299	0,038967	0,921017
ENSG0000	0,000434	-0,030691	2,07238	0,039053	0,921017
ENSG0000	-0,002031	-0,104608	-2,072054	0,039083	0,921017
ENSG0000	-0,004545	0,307851	-2,071955	0,039093	0,921017
ENSG0000	-0,001618	-0,091584	-2,071952	0,039093	0,921017
ENSG0000	-0,005602	0,184083	-2,071424	0,039143	0,921017
ENSG0000	0,002257	-0,030417	2,070046	0,039272	0,921017
ENSG0000	0,002739	-0,330555	2,069007	0,03937	0,921017
ENSG0000	0,004725	0,317435	2,068639	0,039405	0,921017
ENSG0000	0,00148	-0,102411	2,067411	0,039521	0,921017
ENSG0000	0,00237	-0,01465	2,064471	0,039801	0,921017
ENSG0000	0,004443	0,003401	2,064066	0,03984	0,921017
ENSG0000	0,002803	0,018556	2,063835	0,039862	0,921017
ENSG0000	0,002286	0,149844	2,063024	0,039939	0,921017
ENSG0000	-0,001489	-0,02929	-2,062123	0,040025	0,921017
ENSG0000	-0,003026	-0,192781	-2,060454	0,040186	0,921017
ENSG0000	-0,003391	-0,165561	-2,060045	0,040225	0,921017
ENSG0000	-0,002323	0,009615	-2,05967	0,040261	0,921017
ENSG0000	0,004851	-0,044794	2,057806	0,040441	0,921017
ENSG0000	0,001364	-0,218882	2,057206	0,040499	0,921017
ENSG0000	-0,005319	0,298944	-2,057024	0,040517	0,921017
ENSG0000	0,001858	-0,106187	2,055672	0,040648	0,921017
ENSG0000	-0,001392	0,107914	-2,05136	0,041068	0,921017
ENSG0000	0,002021	0,017034	2,048971	0,041303	0,921017
ENSG0000	0,001293	0,074568	2,048156	0,041383	0,921017
ENSG0000	-0,004694	-0,157808	-2,047882	0,04141	0,921017
ENSG0000	-0,006139	0,450251	-2,047473	0,041451	0,921017
ENSG0000	0,003601	0,157233	2,045569	0,041639	0,921017
ENSG0000	0,004166	-0,028966	2,045435	0,041652	0,921017
ENSG0000	0,00161	-0,157898	2,044208	0,041774	0,921017
ENSG0000	-0,001737	0,01295	-2,03775	0,04242	0,921017
ENSG0000	-0,00403	0,160417	-2,037431	0,042452	0,921017
ENSG0000	-0,003779	-0,300544	-2,037259	0,042469	0,921017
ENSG0000	0,002571	0,151308	2,036425	0,042554	0,921017
ENSG0000	-0,009151	-0,415111	-2,035418	0,042655	0,921017
ENSG0000	0,002238	-0,13217	2,034555	0,042743	0,921017
ENSG0000	0,002341	-0,455621	2,033813	0,042818	0,921017
ENSG0000	-0,003206	-0,314314	-2,032679	0,042933	0,921017
ENSG0000	0,004533	0,203289	2,030691	0,043136	0,921017
ENSG0000	0,001987	0,079196	2,030042	0,043202	0,921017
ENSG0000	-0,005363	0,387795	-2,029831	0,043224	0,921017
ENSG0000	0,003689	-0,414593	2,025209	0,043699	0,921017
ENSG0000	0,004722	0,382045	2,020781	0,044158	0,921017
ENSG0000	-0,001681	0,044217	-2,019923	0,044248	0,921017
ENSG0000	-0,002709	0,09019	-2,019286	0,044314	0,921017
ENSG0000	-0,004218	-0,367886	-2,018384	0,044408	0,921017
ENSG0000	-0,002341	0,328364	-2,017594	0,044491	0,921017
ENSG0000	0,000871	0,047648	2,01616	0,044642	0,921017

ENSG0000	-0,004724	-0,413183	-2,013197	0,044954	0,921017
ENSG0000	-0,003411	0,346968	-2,012839	0,044992	0,921017
ENSG0000	0,001524	0,043987	2,012442	0,045034	0,921017
ENSG0000	0,003031	0,250747	2,008211	0,045484	0,921017
ENSG0000	0,005399	-0,339773	2,007277	0,045584	0,921017
ENSG0000	0,002327	0,03812	2,006816	0,045633	0,921017
ENSG0000	-0,002921	-0,092783	-2,00656	0,045661	0,921017
ENSG0000	0,003572	0,049615	2,005121	0,045815	0,921017
ENSG0000	-0,001622	0,416322	-2,004803	0,04585	0,921017
ENSG0000	0,003633	0,14099	2,004531	0,045879	0,921017
ENSG0000	0,002162	0,000655	2,004106	0,045925	0,921017
ENSG0000	-0,001689	-0,181122	-2,002562	0,046091	0,921017
ENSG0000	-0,001828	-0,079007	-2,002302	0,046119	0,921017
ENSG0000	-0,001624	-0,119361	-2,001495	0,046206	0,921017
ENSG0000	0,003208	-0,389772	2,001481	0,046208	0,921017
ENSG0000	-0,001954	-0,178315	-2,001049	0,046255	0,921017
ENSG0000	-0,004983	0,08739	-2,000841	0,046277	0,921017
ENSG0000	0,007924	-0,522468	2,00076	0,046286	0,921017
ENSG0000	0,00144	0,024556	2,000501	0,046314	0,921017
ENSG0000	-0,00203	-0,113909	-2,000077	0,04636	0,921017
ENSG0000	0,004778	-0,201614	1,999539	0,046419	0,921017
ENSG0000	0,002486	-0,263498	1,999291	0,046446	0,921017
ENSG0000	0,006821	-0,388084	1,996758	0,046722	0,921017
ENSG0000	-0,001615	-0,13585	-1,996273	0,046775	0,921017
ENSG0000	-0,006413	-0,162791	-1,995918	0,046814	0,921017
ENSG0000	-0,004202	0,092174	-1,995398	0,046871	0,921017
ENSG0000	0,005618	-0,774593	1,995171	0,046895	0,921017
ENSG0000	0,002494	0,25724	1,994769	0,046939	0,921017
ENSG0000	-0,001007	0,029645	-1,994489	0,04697	0,921017
ENSG0000	0,002837	-0,010515	1,993803	0,047046	0,921017
ENSG0000	0,003663	0,2801	1,991985	0,047246	0,921017
ENSG0000	0,001142	-0,117718	1,991828	0,047263	0,921017
ENSG0000	-0,003042	-0,059166	-1,991778	0,047269	0,921017
ENSG0000	-0,002347	-0,162389	-1,990158	0,047448	0,921017
ENSG0000	-0,003525	0,582962	-1,989827	0,047484	0,921017
ENSG0000	0,001142	-0,067216	1,989801	0,047487	0,921017
ENSG0000	-0,001462	0,045836	-1,98975	0,047493	0,921017
ENSG0000	0,000888	-0,007091	1,986817	0,047819	0,921017
ENSG0000	0,002528	0,160847	1,986453	0,047859	0,921017
ENSG0000	0,007064	0,301081	1,985226	0,047996	0,921017
ENSG0000	-0,003543	-0,028612	-1,98489	0,048034	0,921017
ENSG0000	0,005548	-0,834788	1,98454	0,048073	0,921017
ENSG0000	-0,006295	0,542846	-1,982297	0,048325	0,921017
ENSG0000	0,007079	-0,094301	1,980705	0,048504	0,921017
ENSG0000	0,002685	0,105125	1,979965	0,048588	0,921017
ENSG0000	-0,004435	-0,695327	-1,978899	0,048708	0,921017
ENSG0000	-0,003886	-0,231564	-1,977006	0,048923	0,921017
ENSG0000	-0,002775	-0,382817	-1,974578	0,049199	0,921017

ENSG0000	0,001983	-0,053619	1,973287	0,049347	0,921017
ENSG0000	0,005759	0,950136	1,972551	0,049431	0,921017
ENSG0000	0,003705	-0,555006	1,972114	0,049481	0,921017
ENSG0000	-0,00073	-0,031037	-1,970827	0,049629	0,921017
ENSG0000	-0,003478	-0,017224	-1,970788	0,049634	0,921017
ENSG0000	-0,003347	-0,14206	-1,969255	0,04981	0,921017
ENSG0000	0,002614	0,334141	1,968749	0,049869	0,921017
ENSG0000	-0,005595	0,271549	-1,968507	0,049897	0,921017
ENSG0000	0,004788	0,538365	1,968039	0,049951	0,921017
ENSG0000	-0,001077	-0,105939	-1,967959	0,04996	0,921017

geneID	logFC	AveExpr	t	P.Value	adj.P.Val
ENSG00000162614.14	-0,003739	0,126372	-3,756342	0,000206	0,742593
ENSG00000229212.3	0,007267	-0,33791	3,49365	0,000545	0,742593
ENSG00000162704.11	-0,004836	0,163204	-3,413534	0,000726	0,742593
ENSG00000242498.3	0,005511	-0,221296	3,380459	0,000816	0,742593
ENSG00000133101.5	-0,006801	0,325748	-3,363393	0,000866	0,742593
ENSG00000131697.13	-0,004956	0,077476	-3,34969	0,000908	0,742593
ENSG00000269001.1	0,005239	-0,252369	3,340739	0,000937	0,742593
ENSG00000129467.9	-0,00471	-0,150502	-3,315901	0,001021	0,742593
ENSG00000197586.8	-0,005517	0,129044	-3,277661	0,001165	0,742593
ENSG00000116701.10	0,00492	-0,030445	3,272815	0,001184	0,742593
ENSG00000269051.1	0,008215	-0,246553	3,216561	0,001434	0,742593
ENSG00000272316.1	0,003992	0,247092	3,183271	0,001603	0,742593
ENSG00000267018.1	-0,001936	0,016639	-3,135826	0,001877	0,742593
ENSG00000166359.6	0,003073	0,095878	3,11461	0,002013	0,742593
ENSG00000241106.2	-0,012958	-0,012886	-3,10368	0,002087	0,742593
ENSG00000063127.11	-0,005852	0,069438	-3,100139	0,002111	0,742593
ENSG00000129455.11	-0,004923	-0,17043	-3,093291	0,002159	0,742593
ENSG00000241058.1	-0,010533	0,462089	-3,088037	0,002196	0,742593
ENSG00000064545.10	0,00434	-0,020975	3,082097	0,002239	0,742593
ENSG00000093167.13	0,0038	-0,101884	3,079601	0,002257	0,742593
ENSG00000138600.5	0,006293	0,10537	3,061095	0,002397	0,750777
ENSG00000168778.7	0,010448	0,252687	3,036733	0,002594	0,750777
ENSG00000122873.7	0,006434	-0,401339	3,003286	0,002887	0,750777
ENSG00000204267.9	0,006447	0,067636	3,001903	0,0029	0,750777
ENSG00000239650.3	0,002554	0,138721	2,941954	0,003506	0,750777
ENSG00000105982.12	-0,002988	0,445438	-2,936147	0,003571	0,750777
ENSG00000272541.1	-0,006402	-0,481523	-2,933574	0,0036	0,750777
ENSG00000035687.9	0,003375	-0,131546	2,933283	0,003603	0,750777
ENSG00000132823.6	-0,003048	-0,107816	-2,926505	0,00368	0,750777
ENSG00000109929.5	-0,001769	-0,041605	-2,914635	0,003819	0,750777
ENSG00000271390.1	-0,004389	0,179575	-2,911135	0,003861	0,750777
ENSG00000133103.12	-0,008352	-0,282046	-2,906398	0,003918	0,750777
ENSG00000226629.1	-0,001633	0,079904	-2,895577	0,004052	0,750777
ENSG00000101197.8	0,009108	0,344209	2,886255	0,004171	0,750777
ENSG00000157150.4	0,00852	0,202979	2,880528	0,004245	0,750777
ENSG00000157823.12	-0,008408	-0,991505	-2,87943	0,00426	0,750777
ENSG00000225706.1	0,010787	-0,611903	2,878975	0,004266	0,750777
ENSG00000157017.11	-0,003784	0,113203	-2,873657	0,004336	0,750777
ENSG00000124875.5	-0,001985	0,02584	-2,854749	0,004596	0,757882
ENSG00000054219.9	-0,009954	0,686122	-2,847571	0,004698	0,757882
ENSG00000254333.1	0,005569	0,086237	2,845848	0,004723	0,757882
ENSG00000171476.17	-0,004933	0,386838	-2,832023	0,004927	0,771736
ENSG00000162600.7	-0,0033	-0,098185	-2,814645	0,005194	0,775686
ENSG00000076650.2	0,004758	0,10082	2,80573	0,005336	0,775686
ENSG00000065457.6	-0,011296	0,478726	-2,803253	0,005376	0,775686
ENSG00000104894.7	0,001826	-0,136141	2,800359	0,005424	0,775686
ENSG00000171700.9	0,005175	0,324789	2,77615	0,005834	0,785048
ENSG00000272690.1	0,003019	0,177065	2,761321	0,006098	0,785048
ENSG00000155016.13	-0,006063	-0,15548	-2,760235	0,006118	0,785048
ENSG00000161609.5	0,007868	-0,392939	2,758603	0,006148	0,785048
ENSG00000271584.1	-0,009142	-0,029362	-2,757589	0,006167	0,785048
ENSG00000213903.4	0,002956	0,241993	2,749111	0,006325	0,785048

ENSG00000272864.1	0,010828	-0,810154	2,737644	0,006544	0,785048
ENSG00000089063.10	-0,006001	0,336244	-2,734958	0,006596	0,785048
ENSG00000165806.15	0,010299	-0,58962	2,734354	0,006608	0,785048
ENSG00000182054.5	0,002549	0,12435	2,730586	0,006682	0,785048
ENSG00000100994.7	-0,007928	0,149544	-2,705768	0,00719	0,815579
ENSG00000110063.4	-0,002189	0,066737	-2,698223	0,007351	0,815579
ENSG00000104901.2	0,006413	-0,297689	2,696689	0,007384	0,815579
ENSG00000188163.6	-0,005506	-0,468029	-2,687016	0,007597	0,815579
ENSG00000151883.12	0,004599	0,060442	2,67736	0,007814	0,815579
ENSG00000160321.10	-0,004151	0,114034	-2,671832	0,007941	0,815579
ENSG00000233916.1	-0,001267	-0,085924	-2,667805	0,008034	0,815579
ENSG00000174004.5	0,004888	-0,40818	2,667769	0,008035	0,815579
ENSG00000164068.11	0,005495	-0,206691	2,666799	0,008058	0,815579
ENSG00000103035.6	-0,00146	-0,155233	-2,657043	0,008289	0,820345
ENSG00000259205.2	-0,006338	-0,32838	-2,649933	0,008462	0,820345
ENSG00000196961.8	0,004011	-0,069191	2,645252	0,008577	0,820345
ENSG00000188282.8	-0,001482	0,013648	-2,644181	0,008604	0,820345
ENSG00000108219.10	0,003053	-0,004859	2,624302	0,009111	0,85627
ENSG00000163684.7	-0,002894	-0,133249	-2,614934	0,009359	0,863291
ENSG00000092094.6	0,007537	0,679356	2,601914	0,009713	0,863291
ENSG00000170092.10	-0,005368	-0,277139	-2,595455	0,009894	0,863291
ENSG00000270127.1	-0,007053	-0,29531	-2,592533	0,009977	0,863291
ENSG00000175749.11	-0,003588	-0,230053	-2,592128	0,009988	0,863291
ENSG00000198093.6	0,004987	-0,678677	2,584113	0,010218	0,863291
ENSG00000214826.4	-0,006853	0,162043	-2,583037	0,010249	0,863291
ENSG00000173145.7	0,00152	0,057778	2,58089	0,010312	0,863291
ENSG00000175305.12	0,004084	0,187084	2,578735	0,010375	0,863291
ENSG00000177432.6	0,002536	0,04331	2,57431	0,010506	0,863291
ENSG00000244026.2	0,007073	-0,265862	2,545587	0,011391	0,863291
ENSG00000184207.8	0,003855	0,073439	2,54485	0,011415	0,863291
ENSG00000234073.1	-0,002097	-0,055063	-2,529187	0,011926	0,863291
ENSG00000269552.1	0,002423	-0,348573	2,526401	0,012019	0,863291
ENSG00000182472.4	-0,005529	0,575145	-2,526035	0,012031	0,863291
ENSG00000087206.12	0,001847	-0,567561	2,520454	0,01222	0,863291
ENSG00000198919.8	-0,003999	0,165048	-2,514747	0,012415	0,863291
ENSG00000152223.8	-0,002857	-0,202925	-2,514175	0,012435	0,863291
ENSG00000077238.9	-0,002567	0,025699	-2,507791	0,012657	0,863291
ENSG00000138459.4	0,004096	0,003448	2,506771	0,012693	0,863291
ENSG00000075975.11	0,006273	0,259978	2,505745	0,012729	0,863291
ENSG00000053108.12	-0,005211	-0,114232	-2,504473	0,012774	0,863291
ENSG00000272444.1	0,002444	-0,121533	2,50265	0,012839	0,863291
ENSG00000124678.13	-0,004508	-0,437876	-2,501846	0,012867	0,863291
ENSG00000267013.1	-0,005076	0,389149	-2,501364	0,012885	0,863291
ENSG00000113621.10	-0,001838	-0,092882	-2,499887	0,012937	0,863291
ENSG00000137106.13	0,008703	0,33636	2,499158	0,012964	0,863291
ENSG00000179397.13	-0,001951	0,061308	-2,498191	0,012998	0,863291
ENSG00000245468.3	0,004637	0,105643	2,483733	0,013527	0,863291
ENSG00000114670.9	0,004943	-0,259239	2,482006	0,013591	0,863291
ENSG00000078898.6	-0,002299	-0,326878	-2,478574	0,01372	0,863291
ENSG00000225490.1	0,00433	-0,270619	2,478319	0,01373	0,863291
ENSG00000219435.3	0,002511	0,061479	2,477879	0,013747	0,863291
ENSG00000269067.1	-0,002219	-0,10466	-2,469411	0,01407	0,863291
ENSG00000231952.3	-0,002251	-0,064365	-2,469146	0,01408	0,863291

ENSG00000185838.9	-0,006597	-0,098261	-2,468977	0,014086	0,863291
ENSG00000129480.8	-0,00397	-0,06115	-2,466209	0,014194	0,863291
ENSG00000182103.3	0,003013	0,057822	2,46313	0,014314	0,863291
ENSG00000237419.1	-0,004765	-0,312677	-2,457973	0,014517	0,863291
ENSG00000244753.2	0,001794	0,167035	2,457468	0,014537	0,863291
ENSG00000204228.3	0,002906	-0,206223	2,456487	0,014576	0,863291
ENSG00000162604.8	0,001791	0,085074	2,451901	0,014759	0,863291
ENSG00000197948.6	0,003271	0,114945	2,450199	0,014828	0,863291
ENSG00000162366.3	-0,001652	0,176429	-2,446572	0,014975	0,864208
ENSG00000258231.1	-0,002337	0,141846	-2,436594	0,015386	0,876085
ENSG00000156603.10	-0,00477	0,372828	-2,432516	0,015557	0,876085
ENSG00000182255.6	-0,001907	0,085749	-2,431744	0,01559	0,876085
ENSG00000112799.4	-0,00329	0,262603	-2,428829	0,015713	0,876085
ENSG00000173214.5	0,004745	-0,325317	2,419108	0,016131	0,885161
ENSG00000104714.9	0,003661	0,195977	2,415639	0,016283	0,885161
ENSG00000104331.4	0,002016	-0,050658	2,415111	0,016306	0,885161
ENSG00000145439.7	0,005908	-0,043305	2,411117	0,016482	0,885161
ENSG00000235641.3	0,006202	-0,65902	2,409622	0,016549	0,885161
ENSG00000114904.8	0,003929	-0,600552	2,399915	0,016986	0,901206
ENSG00000153814.7	0,003932	0,461359	2,390254	0,017431	0,917418
ENSG00000164136.12	0,003365	0,177275	2,385685	0,017645	0,921311
ENSG00000250317.4	-0,002208	-0,066351	-2,382717	0,017785	0,921321
ENSG00000178381.7	0,004628	-0,367538	2,377927	0,018013	0,925404
ENSG00000230611.1	-0,002971	0,070523	-2,375189	0,018145	0,925404
ENSG00000177483.7	0,009042	0,129103	2,368655	0,018463	0,928361
ENSG00000196263.3	-0,002972	-0,20284	-2,367212	0,018534	0,928361
ENSG00000188687.11	-0,004166	-0,057199	-2,363668	0,018709	0,928361
ENSG00000089169.10	0,003303	0,189835	2,360885	0,018847	0,928361
ENSG00000254685.2	-0,006006	-0,183625	-2,359654	0,018909	0,928361
ENSG00000146109.3	0,004325	-0,206434	2,356181	0,019083	0,92999
ENSG00000101198.10	0,001682	0,026957	2,337992	0,02002	0,951355
ENSG00000127957.12	0,004332	-0,031428	2,334356	0,020213	0,951355
ENSG00000185324.17	-0,008214	-0,248293	-2,33124	0,020379	0,951355
ENSG00000128791.7	-0,00628	-0,126173	-2,331055	0,020388	0,951355
ENSG00000224557.3	-0,006601	0,298742	-2,330237	0,020432	0,951355
ENSG00000219200.6	-0,003223	-0,069244	-2,329888	0,020451	0,951355
ENSG00000130758.3	0,005549	-0,17681	2,3258	0,020671	0,951355
ENSG00000269973.1	-0,005041	0,116595	-2,324792	0,020726	0,951355
ENSG00000133597.5	0,003688	0,157674	2,321608	0,020899	0,951355
ENSG00000105607.8	0,001141	0,072239	2,320353	0,020968	0,951355
ENSG00000081320.6	-0,002161	-0,132406	-2,311685	0,021447	0,954312
ENSG00000151690.10	0,002628	0,048984	2,308992	0,021598	0,954312
ENSG00000126467.6	0,002543	-0,173323	2,30858	0,021622	0,954312
ENSG00000273033.1	-0,001445	0,116067	-2,305301	0,021807	0,954312
ENSG00000137672.8	0,003057	-0,100911	2,301335	0,022033	0,954312
ENSG00000139187.5	0,006105	0,273796	2,301	0,022052	0,954312
ENSG00000137275.9	0,004581	-0,589191	2,298859	0,022175	0,954312
ENSG00000230772.1	-0,002407	0,340118	-2,294851	0,022406	0,954312
ENSG00000103168.12	-0,0095	-0,557614	-2,292146	0,022564	0,954312
ENSG00000135702.10	-0,00494	0,17044	-2,286142	0,022917	0,954312
ENSG00000033100.10	-0,002018	0,068167	-2,284034	0,023042	0,954312
ENSG00000124508.12	0,00469	0,080014	2,283959	0,023047	0,954312
ENSG00000117090.10	0,002933	-0,307686	2,282908	0,023109	0,954312

ENSG00000260973.1	-0,005718	0,158376	-2,272805	0,023719	0,954312
ENSG00000204767.3	0,004265	-0,065352	2,271864	0,023777	0,954312
ENSG00000256667.2	0,003029	0,116796	2,27172	0,023785	0,954312
ENSG00000145824.8	-0,003196	0,332823	-2,271635	0,023791	0,954312
ENSG00000181523.8	-0,001782	0,000872	-2,270637	0,023852	0,954312
ENSG00000180385.4	-0,002355	0,096718	-2,265912	0,024143	0,954312
ENSG00000178802.13	0,003088	0,229075	2,257332	0,02468	0,954312
ENSG00000198682.8	-0,003273	-0,139658	-2,25639	0,024739	0,954312
ENSG00000104522.11	-0,002323	-0,028058	-2,252754	0,024971	0,954312
ENSG00000271538.1	0,003578	-0,251499	2,249056	0,025208	0,954312
ENSG00000050327.10	0,004161	-0,454452	2,248302	0,025256	0,954312
ENSG00000112137.12	-0,003398	-0,313873	-2,246044	0,025402	0,954312
ENSG00000134873.5	0,004843	-0,018083	2,244794	0,025483	0,954312
ENSG00000165684.3	-0,003111	0,029701	-2,244633	0,025493	0,954312
ENSG00000063438.12	-0,001772	0,06233	-2,243837	0,025545	0,954312
ENSG00000149743.9	-0,003099	0,269433	-2,24159	0,025692	0,954312
ENSG00000136982.5	0,006636	0,202238	2,241135	0,025722	0,954312
ENSG00000170954.7	0,003229	-0,084524	2,233626	0,026218	0,954312
ENSG00000213221.4	0,008573	0,129525	2,232946	0,026263	0,954312
ENSG00000163904.8	0,006867	0,551101	2,228326	0,026573	0,954312
ENSG00000089327.10	-0,008101	-0,051519	-2,224318	0,026844	0,954312
ENSG00000138050.10	-0,003086	-0,107425	-2,223191	0,02692	0,954312
ENSG00000248587.2	-0,00103	-0,134463	-2,222368	0,026977	0,954312
ENSG00000198750.7	0,000537	0,022311	2,22191	0,027008	0,954312
ENSG00000164889.8	0,002325	0,185832	2,221225	0,027055	0,954312
ENSG00000072694.14	-0,00358	0,445796	-2,217163	0,027334	0,954312
ENSG00000170689.8	0,001733	0,06512	2,211684	0,027714	0,954312
ENSG00000204843.8	0,002538	-0,032949	2,20937	0,027876	0,954312
ENSG00000171421.8	0,004779	-0,230766	2,205225	0,028169	0,954312
ENSG00000171103.6	0,006433	0,104192	2,202793	0,028342	0,954312
ENSG00000231742.1	0,004693	0,248186	2,200244	0,028524	0,954312
ENSG00000196199.9	0,002511	-0,369852	2,193708	0,028995	0,954312
ENSG00000204592.5	0,002711	0,305577	2,190154	0,029254	0,954312
ENSG00000184115.12	0,003871	-0,425024	2,189974	0,029268	0,954312
ENSG00000146386.7	0,003899	-0,766305	2,187595	0,029442	0,954312
ENSG00000213853.5	-0,002294	-0,035649	-2,186867	0,029496	0,954312
ENSG00000133704.5	0,00263	-0,093958	2,186045	0,029557	0,954312
ENSG00000137513.5	-0,002543	0,306695	-2,17976	0,030024	0,954312
ENSG00000230305.2	-0,007726	0,371221	-2,178087	0,03015	0,954312
ENSG00000111271.10	-0,001154	-0,005955	-2,177118	0,030222	0,954312
ENSG00000163554.7	-0,00561	0,033581	-2,176417	0,030275	0,954312
ENSG00000079689.9	0,006441	-0,221939	2,172805	0,030549	0,954312
ENSG00000099251.10	0,002856	0,092673	2,172771	0,030551	0,954312
ENSG00000129484.9	0,004392	0,070489	2,171515	0,030647	0,954312
ENSG00000176209.7	-0,00653	-0,389433	-2,167416	0,030961	0,954312
ENSG00000204709.4	-0,003874	0,025879	-2,166147	0,031058	0,954312
ENSG00000184752.8	-0,004942	-0,090254	-2,163248	0,031283	0,954312
ENSG00000064309.10	-0,002122	0,592745	-2,16322	0,031285	0,954312
ENSG00000161944.12	-0,00267	-0,070792	-2,16312	0,031293	0,954312
ENSG00000178397.8	-0,001403	-0,090505	-2,161883	0,031389	0,954312
ENSG00000100234.11	0,002086	0,078464	2,155821	0,031863	0,954312
ENSG00000090054.9	0,004161	0,099392	2,154323	0,031982	0,954312
ENSG00000197279.3	0,002878	-0,058421	2,153244	0,032067	0,954312

ENSG00000226874.1	0,004927	-0,741017	2,149383	0,032374	0,954312
ENSG00000169035.7	-0,002013	-0,261787	-2,148413	0,032452	0,954312
ENSG00000178425.9	-0,002307	-0,078873	-2,14705	0,032561	0,954312
ENSG00000079974.13	0,006552	0,601467	2,146572	0,0326	0,954312
ENSG00000078269.9	0,005296	0,538895	2,1465	0,032605	0,954312
ENSG00000163568.9	-0,001826	-0,224375	-2,143582	0,032841	0,954312
ENSG00000105497.3	0,002813	0,081217	2,143465	0,03285	0,954312
ENSG00000182141.5	-0,004184	-0,122841	-2,143053	0,032883	0,954312
ENSG00000187987.5	-0,006575	0,385299	-2,13651	0,033417	0,954312
ENSG00000141639.7	0,001314	-0,038554	2,136408	0,033425	0,954312
ENSG00000196167.5	-0,003697	-0,00871	-2,135624	0,03349	0,954312
ENSG00000102580.10	0,003368	0,096157	2,133391	0,033674	0,954312
ENSG00000072736.14	-0,002422	-0,061063	-2,128609	0,034071	0,954312
ENSG00000230373.4	0,007029	-0,3061	2,128437	0,034086	0,954312
ENSG00000162545.5	0,004782	-0,684223	2,128222	0,034104	0,954312
ENSG00000205084.6	0,00423	-0,042028	2,128145	0,03411	0,954312
ENSG00000204351.7	0,003945	0,201528	2,125331	0,034346	0,954312
ENSG00000224132.2	-0,002094	0,273641	-2,124097	0,03445	0,954312
ENSG00000143702.11	0,001542	0,100137	2,121993	0,034628	0,954312
ENSG00000168995.9	-0,001627	0,010851	-2,120222	0,034778	0,954312
ENSG00000250778.1	-0,007301	0,11359	-2,119135	0,03487	0,954312
ENSG00000185742.6	0,003107	0,143908	2,118733	0,034904	0,954312
ENSG00000125144.9	-0,002988	-0,349085	-2,118379	0,034935	0,954312
ENSG00000205583.9	0,008943	0,758408	2,116645	0,035083	0,954312
ENSG00000260454.1	-0,00131	-0,061251	-2,1164	0,035104	0,954312
ENSG00000166825.9	-0,004698	-0,254513	-2,115516	0,03518	0,954312
ENSG00000180353.6	0,002121	0,099745	2,113919	0,035317	0,954312
ENSG00000133243.4	-0,003471	0,050788	-2,111708	0,035508	0,954312
ENSG00000100129.13	0,004814	0,318939	2,111232	0,035549	0,954312
ENSG00000188707.4	0,002811	-0,209405	2,105101	0,036083	0,954312
ENSG00000248290.1	-0,004598	-0,079238	-2,102106	0,036347	0,954312
ENSG00000187824.4	-0,004617	0,371939	-2,096677	0,036829	0,954312
ENSG00000272777.1	0,009528	0,058223	2,09312	0,037147	0,954312
ENSG00000108799.8	0,001866	0,096561	2,091396	0,037303	0,954312
ENSG00000187151.3	0,003658	-0,065916	2,091286	0,037312	0,954312
ENSG00000148296.5	0,004921	0,066333	2,089597	0,037465	0,954312
ENSG00000203872.6	-0,004421	-0,259939	-2,085065	0,037878	0,954312
ENSG00000257239.1	0,003406	0,348851	2,083663	0,038006	0,954312
ENSG00000102699.5	0,002651	0,315484	2,083338	0,038036	0,954312
ENSG00000101306.6	-0,003855	0,055094	-2,08133	0,03822	0,954312
ENSG00000088833.13	0,002959	-0,119189	2,081021	0,038249	0,954312
ENSG00000151665.8	-0,001371	0,029664	-2,080207	0,038324	0,954312
ENSG00000113719.11	0,00157	0,002055	2,079792	0,038362	0,954312
ENSG00000216901.1	-0,007158	0,363555	-2,079759	0,038365	0,954312
ENSG00000225422.3	-0,00122	0,059478	-2,07957	0,038383	0,954312
ENSG00000148908.10	-0,001248	0,391008	-2,078627	0,03847	0,954312
ENSG00000204920.6	-0,003916	-0,346857	-2,076068	0,038708	0,954312
ENSG00000164342.8	0,002742	-0,272842	2,075679	0,038744	0,954312
ENSG00000205595.3	0,005636	0,133716	2,075447	0,038766	0,954312
ENSG00000227755.1	0,002454	0,007027	2,07058	0,039222	0,954312
ENSG00000090857.9	-0,001647	-0,26602	-2,06592	0,039663	0,954312
ENSG00000247157.2	-0,009279	-0,475831	-2,065197	0,039732	0,954312
ENSG00000258429.1	-0,002732	-0,133131	-2,059045	0,040321	0,954312

ENSG00000260459.2	-0,003329	0,502716	-2,056157	0,0406	0,954312
ENSG00000121406.4	0,000781	0,007022	2,055823	0,040633	0,954312
ENSG00000144230.12	0,006771	0,735355	2,055498	0,040664	0,954312
ENSG00000134962.6	-0,003923	-0,376733	-2,053461	0,040863	0,954312
ENSG00000124357.8	-0,003613	-0,21437	-2,052455	0,040961	0,954312
ENSG00000225507.1	-0,001731	0,003716	-2,047871	0,041411	0,954312
ENSG00000151893.10	0,005317	-0,454355	2,047576	0,04144	0,954312
ENSG00000271590.1	0,001342	-0,049177	2,046069	0,041589	0,954312
ENSG00000108244.12	-0,003679	-0,049506	-2,046008	0,041595	0,954312
ENSG00000178917.10	0,003652	0,019202	2,044592	0,041736	0,954312
ENSG00000185880.8	-0,005907	-0,134038	-2,044396	0,041755	0,954312
ENSG00000119446.9	-0,003575	0,93851	-2,044101	0,041784	0,954312
ENSG00000058085.10	0,002659	0,11413	2,042421	0,041952	0,954312
ENSG00000115310.13	-0,003118	-0,139343	-2,041376	0,042056	0,954312
ENSG00000272902.1	-0,00327	-0,026221	-2,039661	0,042228	0,954312
ENSG00000175220.7	-0,003154	0,346616	-2,03812	0,042383	0,954312
ENSG00000132128.12	-0,001364	-0,092782	-2,033909	0,042808	0,954312
ENSG00000243566.2	0,007233	-0,426348	2,032558	0,042945	0,954312
ENSG00000187634.6	0,001938	0,224612	2,03022	0,043184	0,954312
ENSG00000122643.14	0,003184	0,073347	2,029759	0,043231	0,954312
ENSG00000154122.8	0,001702	0,055218	2,029189	0,043289	0,954312
ENSG00000185040.11	-0,002978	0,079143	-2,029053	0,043303	0,954312
ENSG00000196922.6	-0,005161	0,135131	-2,028368	0,043373	0,954312
ENSG00000272130.1	-0,000763	-0,111346	-2,028199	0,043391	0,954312
ENSG00000145029.7	-0,003561	0,411043	-2,026156	0,043601	0,954312
ENSG00000259005.1	0,003483	0,22076	2,025247	0,043695	0,954312
ENSG00000163331.6	-0,00267	-0,222604	-2,024916	0,043729	0,954312
ENSG00000016602.8	-0,002327	-0,22682	-2,022339	0,043996	0,954312
ENSG00000188295.10	0,003637	0,111184	2,020433	0,044194	0,954312
ENSG00000243710.3	0,008263	-0,815088	2,019982	0,044241	0,954312
ENSG00000156170.8	0,00168	0,106985	2,018331	0,044414	0,954312
ENSG00000196460.8	0,005519	0,647866	2,018092	0,044439	0,954312
ENSG00000170571.7	-0,001638	0,133797	-2,017454	0,044506	0,954312
ENSG00000213453.3	0,002355	0,235613	2,013871	0,044883	0,954312
ENSG00000204428.8	0,004472	-0,238201	2,013226	0,044951	0,954312
ENSG00000114841.13	0,002181	0,134397	2,012516	0,045026	0,954312
ENSG00000113119.8	0,003151	0,164157	2,011547	0,045129	0,954312
ENSG00000227671.3	-0,00374	-0,118338	-2,008006	0,045506	0,954312
ENSG00000227558.4	-0,001727	0,60828	-2,007943	0,045513	0,954312
ENSG00000229891.1	0,005934	-0,655594	2,004733	0,045857	0,954312
ENSG00000197614.6	0,002346	0,038197	2,002502	0,046097	0,954312
ENSG00000125912.6	-0,004851	-0,296646	-2,001243	0,046234	0,954312
ENSG00000122085.12	-0,004049	0,406853	-2,000621	0,046301	0,954312
ENSG00000106070.13	-0,001416	0,089265	-2,000602	0,046303	0,954312
ENSG00000021762.15	-0,003732	0,183228	-1,999154	0,04646	0,954312
ENSG00000272195.1	-0,006859	0,614026	-1,998397	0,046543	0,954312
ENSG00000152601.13	-0,000886	-0,030737	-1,996478	0,046752	0,954312
ENSG00000180881.15	-0,003025	-0,059424	-1,994514	0,046967	0,954312
ENSG00000076924.7	-0,002453	-0,147875	-1,992664	0,047171	0,954312
ENSG00000113946.3	0,003178	-0,267454	1,992095	0,047234	0,954312
ENSG00000178226.6	-0,003466	-0,394779	-1,991094	0,047344	0,954312
ENSG00000170837.2	-0,005242	-0,075083	-1,990593	0,047399	0,954312
ENSG00000256673.1	-0,005965	0,23391	-1,990558	0,047403	0,954312

ENSG00000160325.10	-0,002041	-0,164047	-1,990197	0,047443	0,954312
ENSG00000071243.11	-0,002176	-0,285353	-1,98907	0,047568	0,954312
ENSG00000182685.3	0,001447	0,044954	1,987522	0,04774	0,954312
ENSG00000168404.8	-0,002189	0,182167	-1,984442	0,048084	0,954312
ENSG00000214402.6	-0,003147	-0,593286	-1,984239	0,048107	0,954312
ENSG00000105894.7	0,001644	0,113458	1,981059	0,048464	0,954312
ENSG00000101158.8	0,006236	-0,311799	1,980883	0,048484	0,954312
ENSG00000132406.7	0,004286	0,688558	1,979629	0,048625	0,954312
ENSG00000172014.11	-0,002304	-0,032223	-1,976089	0,049027	0,954312
ENSG00000163737.3	-0,002091	0,043567	-1,972639	0,049421	0,954312
ENSG00000078053.12	0,006884	0,089382	1,970773	0,049635	0,954312
