# Supplementary material for: The transcriptome-wide association search for genes and genetic variants which associate with BMI and gestational weight gain in women with type 1 diabetes
Source: Mol Med. 2021 Jan 20;27:6. doi: 10.1186/s10020-020-00266-z (PMC7818927; doi:10.1186/s10020-020-00266-z)
Supplement: Supplementary file 2 — Additional file 2: Table S1a. The results of PrediXcan on BMI in T1D cohort in Subcutaneous Adipose Tissue. b. The results of PrediXcan on BMI in T1D cohort in Visceral Adipose Tissue. c. The results of PrediXcan on GWG in T1D cohort in Subcutaneous Adipose Tissue. d. The results of PrediXcan on GWG in T1D cohort in Visceral Adipose Tissue. [file 10020_2020_266_MOESM2_ESM.zip › Table S1b.pdf]

| geneID | logFC | AveExpr | t | P.Value | adj.P.Val |
|---|---|---|---|---|---|
| ENSG00000 | -0,006256 | 0,6410333 | -3,912956 | 0,0001116 | 0,7459214 |
| ENSG00000 | 0,0051341 | 0,0757664 | 3,7171071 | 0,0002381 | 0,7459214 |
| ENSG00000 | -0,003138 | 0,0039857 | -3,6381 | 0,0003205 | 0,7459214 |
| ENSG00000 | 0,0040141 | -0,034803 | 3,5960262 | 0,0003746 | 0,7459214 |
| ENSG00000 | -0,003532 | -0,199263 | -3,495076 | 0,0005414 | 0,7459214 |
| ENSG00000 | -0,006816 | -0,37862 | -3,4946 | 0,0005423 | 0,7459214 |
| ENSG00000 | 0,0053605 | -0,100292 | 3,4479513 | 0,0006411 | 0,7558144 |
| ENSG00000 | -0,008821 | -0,323208 | -3,377377 | 0,0008229 | 0,7823791 |
| ENSG00000 | 0,0037684 | -0,187996 | 3,3122953 | 0,0010322 | 0,7823791 |
| ENSG00000 | -0,006532 | 0,1600234 | -3,271747 | 0,0011865 | 0,7823791 |
| ENSG00000 | -0,010326 | 0,0277261 | -3,26904 | 0,0011975 | 0,7823791 |
| ENSG00000 | 0,0062138 | 0,3104081 | 3,2650884 | 0,0012138 | 0,7823791 |
| ENSG00000 | 0,0072371 | 0,3368309 | 3,2516949 | 0,0012705 | 0,7823791 |
| ENSG00000 | -0,018756 | -0,278461 | -3,219325 | 0,0014179 | 0,7823791 |
| ENSG00000 | 0,0010848 | -0,049646 | 3,2159347 | 0,0014342 | 0,7823791 |
| ENSG00000 | 0,0033282 | 0,093864 | 3,1992998 | 0,0015168 | 0,7823791 |
| ENSG00000 | -0,004808 | 0,0679016 | -3,165296 | 0,0016995 | 0,8250625 |
| ENSG00000 | -0,00205 | 0,0730004 | -3,0836 | 0,0022247 | 0,8710043 |
| ENSG00000 | 0,0011758 | 0,0221059 | 3,078179 | 0,0022644 | 0,8710043 |
| ENSG00000 | 0,0042503 | 0,0247118 | 3,055617 | 0,0024366 | 0,8710043 |
| ENSG00000 | -0,016446 | -1,118148 | -3,03921 | 0,0025692 | 0,8710043 |
| ENSG00000 | -0,005888 | 0,0107987 | -3,029385 | 0,0026518 | 0,8710043 |
| ENSG00000 | 0,0059425 | -0,396 | 3,0239927 | 0,0026982 | 0,8710043 |
| ENSG00000 | -0,004852 | 0,0182326 | -3,022834 | 0,0027082 | 0,8710043 |
| ENSG00000 | -0,015887 | -0,097225 | -3,015307 | 0,0027744 | 0,8710043 |
| ENSG00000 | 0,0037956 | 0,17786 | 3,0055923 | 0,002862 | 0,8710043 |
| ENSG00000 | -0,007983 | -0,089808 | -2,994741 | 0,0029629 | 0,8710043 |
| ENSG00000 | -0,011608 | 0,2877501 | -2,984433 | 0,0030617 | 0,8710043 |
| ENSG00000 | -0,003041 | -0,022507 | -2,919789 | 0,0037533 | 0,8710043 |
| ENSG00000 | -0,028318 | -1,02467 | -2,919354 | 0,0037584 | 0,8710043 |
| ENSG00000 | 0,0047144 | -0,010874 | 2,9028282 | 0,003957 | 0,8710043 |
| ENSG00000 | -0,008064 | 0,2444339 | -2,893542 | 0,0040728 | 0,8710043 |
| ENSG00000 | -0,003815 | 0,000944 | -2,892198 | 0,0040898 | 0,8710043 |
| ENSG00000 | -0,014489 | -0,523584 | -2,873194 | 0,0043373 | 0,8710043 |
| ENSG00000 | -0,004476 | -0,01039 | -2,867052 | 0,0044202 | 0,8710043 |
| ENSG00000 | 0,005073 | -0,090701 | 2,8647533 | 0,0044516 | 0,8710043 |
| ENSG00000 | -0,003617 | -0,056725 | -2,863324 | 0,0044712 | 0,8710043 |
| ENSG00000 | -0,004855 | -0,151685 | -2,862364 | 0,0044844 | 0,8710043 |
| ENSG00000 | -0,004319 | 0,1186282 | -2,858573 | 0,004537 | 0,8710043 |
| ENSG00000 | 0,0061558 | -0,146667 | 2,846582 | 0,0047069 | 0,8710043 |
| ENSG00000 | 0,0038468 | -0,07273 | 2,8463799 | 0,0047098 | 0,8710043 |
| ENSG00000 | -0,005324 | -0,25473 | -2,841967 | 0,0047738 | 0,8710043 |
| ENSG00000 | -0,006825 | 0,0967081 | -2,837898 | 0,0048336 | 0,8710043 |
| ENSG00000 | -0,005616 | 0,0593388 | -2,825447 | 0,0050206 | 0,8710043 |
| ENSG00000 | -0,005956 | -0,336847 | -2,81659 | 0,0051576 | 0,8710043 |
| ENSG00000 | -0,005774 | 0,0947427 | -2,815725 | 0,0051711 | 0,8710043 |
| ENSG00000 | -0,009733 | -0,540222 | -2,801107 | 0,0054053 | 0,8710043 |
| ENSG00000 | -0,006036 | 0,059998 | -2,795126 | 0,0055038 | 0,8710043 |

| | | | | | |
|---|---|---|---|---|---|
| ENSG0000( | 0,0094148 | -0,128908 | 2,7946603 | 0,0055115 | 0,8710043 |
| ENSG0000( | 0,003892 | 0,1208641 | 2,789403 | 0,0055996 | 0,8710043 |
| ENSG0000( | -0,004855 | -0,057204 | -2,785466 | 0,0056664 | 0,8710043 |
| ENSG0000( | -0,013154 | 0,6951315 | -2,783883 | 0,0056934 | 0,8710043 |
| ENSG0000( | 0,0016282 | 0,0117746 | 2,782913 | 0,0057101 | 0,8710043 |
| ENSG0000( | 0,015181 | 0,6540676 | 2,7802276 | 0,0057564 | 0,8710043 |
| ENSG0000( | -0,001336 | 0,0337175 | -2,76773 | 0,0059763 | 0,8710043 |
| ENSG0000( | 0,0033612 | 0,0566798 | 2,7634086 | 0,0060541 | 0,8710043 |
| ENSG0000( | -0,004503 | 0,1097526 | -2,747384 | 0,0063506 | 0,8710043 |
| ENSG0000( | -0,009042 | -0,003662 | -2,741019 | 0,006472 | 0,8710043 |
| ENSG0000( | 0,0059749 | 0,4220187 | 2,7356984 | 0,006575 | 0,8710043 |
| ENSG0000( | -0,008574 | 0,3935604 | -2,733758 | 0,006613 | 0,8710043 |
| ENSG0000( | -0,004553 | 0,0223958 | -2,732461 | 0,0066385 | 0,8710043 |
| ENSG0000( | 0,0040928 | 0,0366008 | 2,7315307 | 0,0066568 | 0,8710043 |
| ENSG0000( | -0,014692 | -0,665038 | -2,726769 | 0,0067514 | 0,8710043 |
| ENSG0000( | -0,005969 | 0,1430344 | -2,726615 | 0,0067544 | 0,8710043 |
| ENSG0000( | -0,008304 | -0,126638 | -2,698864 | 0,0073302 | 0,9300817 |
| ENSG0000( | -0,002338 | 0,0921081 | -2,685759 | 0,0076172 | 0,9300817 |
| ENSG0000( | 0,0043466 | 0,0639371 | 2,6838766 | 0,0076592 | 0,9300817 |
| ENSG0000( | 0,0031144 | 0,0193566 | 2,6819421 | 0,0077026 | 0,9300817 |
| ENSG0000( | 0,0031205 | 0,1248518 | 2,6770647 | 0,0078131 | 0,9300817 |
| ENSG0000( | -0,006284 | 0,2262804 | -2,672446 | 0,007919 | 0,9300817 |
| ENSG0000( | -0,003266 | -0,146732 | -2,668892 | 0,0080014 | 0,9300817 |
| ENSG0000( | -0,013536 | 0,5191483 | -2,648159 | 0,0084976 | 0,9729065 |
| ENSG0000( | -0,010869 | 0,6239411 | -2,639894 | 0,008703 | 0,9729065 |
| ENSG0000( | 0,0050494 | -0,301345 | 2,6291891 | 0,0089756 | 0,9729065 |
| ENSG0000( | -0,004146 | -0,302822 | -2,623443 | 0,009125 | 0,9729065 |
| ENSG0000( | 0,0027834 | -0,189582 | 2,6177663 | 0,0092749 | 0,9729065 |
| ENSG0000( | -0,004848 | -0,351281 | -2,616837 | 0,0092996 | 0,9729065 |
| ENSG0000( | 0,0046434 | -0,180767 | 2,6128392 | 0,0094068 | 0,9729065 |
| ENSG0000( | -0,005314 | 0,3335944 | -2,606597 | 0,0095762 | 0,9729065 |
| ENSG0000( | -0,005567 | -0,045888 | -2,606234 | 0,0095862 | 0,9729065 |
| ENSG0000( | 0,0048072 | -0,364403 | 2,6013057 | 0,009722 | 0,9729065 |
| ENSG0000( | -0,003639 | -0,031759 | -2,594447 | 0,0099139 | 0,9729065 |
| ENSG0000( | 0,0014328 | 0,0029887 | 2,5897154 | 0,0100483 | 0,9729065 |
| ENSG0000( | -0,006981 | 0,3822751 | -2,585599 | 0,0101665 | 0,9729065 |
| ENSG0000( | 0,0037659 | -0,0023 | 2,5805907 | 0,010312 | 0,9729065 |
| ENSG0000( | 0,0068657 | 0,3052733 | 2,5778143 | 0,0103935 | 0,9729065 |
| ENSG0000( | -0,007339 | -0,050911 | -2,577035 | 0,0104165 | 0,9729065 |
| ENSG0000( | 0,0062912 | 0,2853162 | 2,5716811 | 0,0105755 | 0,9729065 |
| ENSG0000( | -0,002858 | 0,0165588 | -2,566299 | 0,0107376 | 0,9729065 |
| ENSG0000( | -0,003114 | -0,100938 | -2,561712 | 0,0108774 | 0,9729065 |
| ENSG0000( | -0,002192 | -0,07436 | -2,560308 | 0,0109206 | 0,9729065 |
| ENSG0000( | -0,005407 | 0,1958194 | -2,554764 | 0,0110924 | 0,9729065 |
| ENSG0000( | 0,0032855 | -0,015159 | 2,547516 | 0,0113207 | 0,9729065 |
| ENSG0000( | 0,0037439 | -0,003867 | 2,5416403 | 0,0115088 | 0,9729065 |
| ENSG0000( | 0,0044434 | -0,160836 | 2,5368032 | 0,0116657 | 0,9729065 |
| ENSG0000( | 0,0049185 | -0,056501 | 2,5330552 | 0,0117886 | 0,9729065 |

| | | | | | |
|---|---|---|---|---|---|
| ENSG0000( | -0,003676 | 0,0830859 | -2,529484 | 0,0119068 | 0,9729065 |
| ENSG0000( | 0,0042908 | 0,0915559 | 2,5293613 | 0,0119109 | 0,9729065 |
| ENSG0000( | 0,0042762 | 0,1469153 | 2,5249244 | 0,0120593 | 0,9729065 |
| ENSG0000( | -0,002056 | -0,045571 | -2,523047 | 0,0121225 | 0,9729065 |
| ENSG0000( | -0,003509 | -0,038781 | -2,517569 | 0,0123089 | 0,9729065 |
| ENSG0000( | -0,005995 | -0,419319 | -2,513925 | 0,0124342 | 0,9729065 |
| ENSG0000( | 0,0008833 | -0,05938 | 2,5113912 | 0,012522 | 0,9729065 |
| ENSG0000( | 0,0038949 | 0,0639491 | 2,5111106 | 0,0125318 | 0,9729065 |
| ENSG0000( | -0,008756 | 0,0498482 | -2,508362 | 0,0126277 | 0,9729065 |
| ENSG0000( | -0,00603 | -0,15719 | -2,507261 | 0,0126663 | 0,9729065 |
| ENSG0000( | -0,005447 | 0,0616659 | -2,497549 | 0,0130116 | 0,9729065 |
| ENSG0000( | 0,0129664 | -0,921417 | 2,4971 | 0,0130278 | 0,9729065 |
| ENSG0000( | 0,0054878 | 0,3966196 | 2,4888199 | 0,013329 | 0,9729065 |
| ENSG0000( | 0,0045441 | 0,0284702 | 2,4886484 | 0,0133353 | 0,9729065 |
| ENSG0000( | 0,0022382 | 0,017558 | 2,4857286 | 0,0134431 | 0,9729065 |
| ENSG0000( | -0,00245 | 0,1669204 | -2,480773 | 0,0136277 | 0,9729065 |
| ENSG0000( | -0,004948 | 0,3747242 | -2,480498 | 0,013638 | 0,9729065 |
| ENSG0000( | -0,003947 | 0,3351219 | -2,464793 | 0,0142385 | 0,9729065 |
| ENSG0000( | -0,008734 | 0,4881661 | -2,460349 | 0,0144126 | 0,9729065 |
| ENSG0000( | -0,005719 | 0,0092145 | -2,455924 | 0,0145879 | 0,9729065 |
| ENSG0000( | 0,0045674 | 0,116924 | 2,4524688 | 0,014726 | 0,9729065 |
| ENSG0000( | -0,003159 | -0,001646 | -2,447855 | 0,0149123 | 0,9729065 |
| ENSG0000( | 0,0045688 | 0,245 | 2,4415584 | 0,0151699 | 0,9729065 |
| ENSG0000( | 0,0052728 | 0,1206025 | 2,4381809 | 0,0153097 | 0,9729065 |
| ENSG0000( | -0,004202 | 0,113957 | -2,43666 | 0,015373 | 0,9729065 |
| ENSG0000( | -0,011156 | -0,274592 | -2,434375 | 0,0154686 | 0,9729065 |
| ENSG0000( | -0,002393 | 0,1510328 | -2,433812 | 0,0154922 | 0,9729065 |
| ENSG0000( | -0,008185 | 0,1745581 | -2,431371 | 0,015595 | 0,9729065 |
| ENSG0000( | 0,0093139 | -0,177243 | 2,4309902 | 0,0156111 | 0,9729065 |
| ENSG0000( | -0,011218 | 0,3492591 | -2,430035 | 0,0156515 | 0,9729065 |
| ENSG0000( | 0,0132378 | -0,488931 | 2,4292978 | 0,0156828 | 0,9729065 |
| ENSG0000( | 0,0048079 | -0,318056 | 2,4285791 | 0,0157133 | 0,9729065 |
| ENSG0000( | 0,0025802 | 0,0783862 | 2,4277536 | 0,0157485 | 0,9729065 |
| ENSG0000( | 0,0049351 | -0,496288 | 2,4255973 | 0,0158406 | 0,9729065 |
| ENSG0000( | -0,006367 | 0,1099785 | -2,423219 | 0,0159427 | 0,9729065 |
| ENSG0000( | -0,008719 | -0,090504 | -2,421661 | 0,01601 | 0,9729065 |
| ENSG0000( | -0,001848 | -0,045174 | -2,421221 | 0,016029 | 0,9729065 |
| ENSG0000( | 0,0015319 | -0,066366 | 2,4167252 | 0,0162246 | 0,9729065 |
| ENSG0000( | -0,003796 | -0,094096 | -2,41173 | 0,0164444 | 0,9729065 |
| ENSG0000( | 0,0047979 | 0,1943586 | 2,4113857 | 0,0164596 | 0,9729065 |
| ENSG0000( | 0,0052435 | -0,049269 | 2,4088577 | 0,016572 | 0,9729065 |
| ENSG0000( | -0,003174 | -0,155257 | -2,406609 | 0,0166725 | 0,9729065 |
| ENSG0000( | -0,005489 | 0,3505217 | -2,405326 | 0,01673 | 0,9729065 |
| ENSG0000( | -0,004141 | 0,2680887 | -2,395294 | 0,0171863 | 0,9729065 |
| ENSG0000( | 0,0035805 | -0,030482 | 2,3877663 | 0,0175358 | 0,9729065 |
| ENSG0000( | 0,0076969 | 0,5850117 | 2,3867472 | 0,0175836 | 0,9729065 |
| ENSG0000( | 0,0025366 | 0,1371941 | 2,3858029 | 0,017628 | 0,9729065 |
| ENSG0000( | -0,00718 | 0,2934514 | -2,384275 | 0,0177001 | 0,9729065 |

| | | | | | |
|---|---|---|---|---|---|
| ENSG0000( | 0,0024919 | -0,071995 | 2,3755256 | 0,0181176 | 0,9729065 |
| ENSG0000( | 0,0036157 | 0,1661843 | 2,3754337 | 0,018122 | 0,9729065 |
| ENSG0000( | -0,003177 | 0,1577893 | -2,374597 | 0,0181624 | 0,9729065 |
| ENSG0000( | 0,005503 | 0,154975 | 2,3695997 | 0,0184053 | 0,9729065 |
| ENSG0000( | 0,0032252 | 0,0907004 | 2,3621715 | 0,0187716 | 0,9729065 |
| ENSG0000( | -0,00564 | 0,2544379 | -2,360029 | 0,0188784 | 0,9729065 |
| ENSG0000( | 0,0042684 | 0,3378487 | 2,3581116 | 0,0189744 | 0,9729065 |
| ENSG0000( | 0,0040923 | 0,0644512 | 2,3570667 | 0,019027 | 0,9729065 |
| ENSG0000( | 0,0030181 | -0,097956 | 2,3563335 | 0,0190639 | 0,9729065 |
| ENSG0000( | -0,012869 | -0,571204 | -2,355547 | 0,0191036 | 0,9729065 |
| ENSG0000( | -0,00629 | 0,2052961 | -2,35541 | 0,0191105 | 0,9729065 |
| ENSG0000( | -0,00618 | 0,2603571 | -2,353673 | 0,0191985 | 0,9729065 |
| ENSG0000( | 0,0057096 | -0,03816 | 2,3476824 | 0,0195045 | 0,9729065 |
| ENSG0000( | 0,0056795 | 0,0783621 | 2,3433898 | 0,0197264 | 0,9729065 |
| ENSG0000( | 0,0056146 | -0,553495 | 2,3432669 | 0,0197328 | 0,9729065 |
| ENSG0000( | 0,0023705 | -0,072654 | 2,339038 | 0,0199536 | 0,9729065 |
| ENSG0000( | -0,002079 | 0,0619621 | -2,338848 | 0,0199636 | 0,9729065 |
| ENSG0000( | 0,0018894 | -0,061411 | 2,3325282 | 0,0202978 | 0,9729065 |
| ENSG0000( | -0,003858 | 0,0267552 | -2,33116 | 0,0203709 | 0,9729065 |
| ENSG0000( | -0,00461 | 0,9256922 | -2,331055 | 0,0203764 | 0,9729065 |
| ENSG0000( | -0,004639 | 0,3775583 | -2,331016 | 0,0203785 | 0,9729065 |
| ENSG0000( | -0,009654 | -0,368189 | -2,330539 | 0,020404 | 0,9729065 |
| ENSG0000( | -0,003981 | -0,021768 | -2,323701 | 0,0207729 | 0,9729065 |
| ENSG0000( | -0,005481 | -0,183598 | -2,32183 | 0,0208748 | 0,9729065 |
| ENSG0000( | 0,0080367 | -0,041982 | 2,3211639 | 0,0209112 | 0,9729065 |
| ENSG0000( | -0,003501 | -0,216446 | -2,320571 | 0,0209436 | 0,9729065 |
| ENSG0000( | 0,0069334 | -0,257927 | 2,3174976 | 0,0211125 | 0,9729065 |
| ENSG0000( | 0,0072322 | 0,2747618 | 2,3169341 | 0,0211436 | 0,9729065 |
| ENSG0000( | -0,002715 | 0,1887842 | -2,316813 | 0,0211503 | 0,9729065 |
| ENSG0000( | 0,0051077 | 0,2477536 | 2,3147455 | 0,0212647 | 0,9729065 |
| ENSG0000( | 0,0052913 | 0,2986103 | 2,3130296 | 0,0213601 | 0,9729065 |
| ENSG0000( | 0,0014165 | 0,0743708 | 2,3120667 | 0,0214138 | 0,9729065 |
| ENSG0000( | -0,002378 | -0,118908 | -2,310413 | 0,0215063 | 0,9729065 |
| ENSG0000( | -0,007607 | -0,225056 | -2,308855 | 0,0215938 | 0,9729065 |
| ENSG0000( | -0,005824 | 0,0236319 | -2,302694 | 0,0219427 | 0,9729065 |
| ENSG0000( | 0,0054718 | 0,8697901 | 2,3000793 | 0,0220923 | 0,9729065 |
| ENSG0000( | 0,0063343 | -0,364322 | 2,2952745 | 0,0223694 | 0,9729065 |
| ENSG0000( | 0,0047284 | -0,4832 | 2,294844 | 0,0223944 | 0,9729065 |
| ENSG0000( | 0,0033937 | 0,0273266 | 2,2893162 | 0,0227173 | 0,9729065 |
| ENSG0000( | 0,003825 | 0,3645551 | 2,2883185 | 0,022776 | 0,9729065 |
| ENSG0000( | 0,0045763 | -0,039648 | 2,2815688 | 0,0231768 | 0,9729065 |
| ENSG0000( | -0,001471 | 0,0185464 | -2,280972 | 0,0232125 | 0,9729065 |
| ENSG0000( | -0,004542 | -0,314156 | -2,279371 | 0,0233085 | 0,9729065 |
| ENSG0000( | 0,0041497 | 0,296913 | 2,2775297 | 0,0234195 | 0,9729065 |
| ENSG0000( | 0,0036077 | -0,100358 | 2,2754954 | 0,0235426 | 0,9729065 |
| ENSG0000( | -0,010084 | -0,429574 | -2,274949 | 0,0235757 | 0,9729065 |
| ENSG0000( | -0,003152 | 0,062274 | -2,274094 | 0,0236277 | 0,9729065 |
| ENSG0000( | 0,0015306 | 0,0447058 | 2,2733917 | 0,0236705 | 0,9729065 |

| | | | | | |
|---|---|---|---|---|---|
| ENSG0000( | 0,0036036 | 0,017261 | 2,2724604 | 0,0237273 | 0,9729065 |
| ENSG0000( | -0,001587 | -0,014443 | -2,27029 | 0,0238602 | 0,9729065 |
| ENSG0000( | 0,0074256 | -0,762865 | 2,2702572 | 0,0238621 | 0,9729065 |
| ENSG0000( | 0,0077079 | 0,1723903 | 2,2679828 | 0,0240021 | 0,9729065 |
| ENSG0000( | -0,003072 | 0,3404827 | -2,267167 | 0,0240524 | 0,9729065 |
| ENSG0000( | -0,003249 | 0,0439139 | -2,266453 | 0,0240966 | 0,9729065 |
| ENSG0000( | -0,001584 | -0,028108 | -2,266092 | 0,0241189 | 0,9729065 |
| ENSG0000( | 0,0064363 | -0,019116 | 2,2641667 | 0,0242384 | 0,9729065 |
| ENSG0000( | 0,0032998 | 0,1904747 | 2,2634459 | 0,0242833 | 0,9729065 |
| ENSG0000( | -0,008012 | -0,243964 | -2,262357 | 0,0243512 | 0,9729065 |
| ENSG0000( | -0,005714 | 0,0208263 | -2,261066 | 0,024432 | 0,9729065 |
| ENSG0000( | 0,0037009 | 0,0491465 | 2,2593408 | 0,0245402 | 0,9729065 |
| ENSG0000( | -0,002675 | 0,1722857 | -2,255804 | 0,0247635 | 0,9729065 |
| ENSG0000( | 0,0035721 | -0,021557 | 2,2528927 | 0,0249486 | 0,9729065 |
| ENSG0000( | 0,0042967 | 0,2376224 | 2,2513777 | 0,0250454 | 0,9729065 |
| ENSG0000( | 0,0049465 | 0,1041166 | 2,247197 | 0,0253142 | 0,9729065 |
| ENSG0000( | 0,0079563 | -0,469365 | 2,2470409 | 0,0253243 | 0,9729065 |
| ENSG0000( | 0,0014653 | -0,090966 | 2,2395547 | 0,0258121 | 0,9729065 |
| ENSG0000( | -0,003587 | -0,169467 | -2,239221 | 0,0258341 | 0,9729065 |
| ENSG0000( | 0,0037359 | -0,257312 | 2,2348717 | 0,0261214 | 0,9729065 |
| ENSG0000( | 0,0024515 | 0,0504099 | 2,2348039 | 0,0261259 | 0,9729065 |
| ENSG0000( | 0,0053578 | 0,0337103 | 2,2334574 | 0,0262155 | 0,9729065 |
| ENSG0000( | -0,004255 | -0,026984 | -2,231913 | 0,0263185 | 0,9729065 |
| ENSG0000( | 0,0079393 | 0,1043292 | 2,2318775 | 0,0263208 | 0,9729065 |
| ENSG0000( | -0,004017 | 0,1024226 | -2,231547 | 0,0263429 | 0,9729065 |
| ENSG0000( | -0,006234 | -0,467085 | -2,229759 | 0,0264627 | 0,9729065 |
| ENSG0000( | -0,002713 | -0,023082 | -2,22952 | 0,0264788 | 0,9729065 |
| ENSG0000( | 0,0127175 | -0,690802 | 2,2289938 | 0,0265142 | 0,9729065 |
| ENSG0000( | 0,0028646 | 0,1268175 | 2,2288712 | 0,0265224 | 0,9729065 |
| ENSG0000( | -0,005541 | -0,357712 | -2,226369 | 0,0266912 | 0,9729065 |
| ENSG0000( | -0,002216 | -0,053708 | -2,224921 | 0,0267893 | 0,9729065 |
| ENSG0000( | -0,006247 | 0,2828768 | -2,220129 | 0,0271162 | 0,9729065 |
| ENSG0000( | 0,0043236 | 0,1117175 | 2,2196627 | 0,0271482 | 0,9729065 |
| ENSG0000( | -0,004062 | 0,3782501 | -2,218106 | 0,0272552 | 0,9729065 |
| ENSG0000( | -0,004597 | -0,620831 | -2,21506 | 0,0274657 | 0,9729065 |
| ENSG0000( | -0,009682 | 0,5355756 | -2,21364 | 0,0275644 | 0,9729065 |
| ENSG0000( | -0,005198 | -0,364089 | -2,212719 | 0,0276285 | 0,9729065 |
| ENSG0000( | -0,007171 | 0,5551157 | -2,206935 | 0,0280342 | 0,9729065 |
| ENSG0000( | -0,004644 | -0,037687 | -2,206622 | 0,0280563 | 0,9729065 |
| ENSG0000( | 0,0070899 | -0,010298 | 2,2043512 | 0,028217 | 0,9729065 |
| ENSG0000( | 0,0026816 | 0,2051901 | 2,2041645 | 0,0282303 | 0,9729065 |
| ENSG0000( | 0,0062134 | 0,3478176 | 2,2037887 | 0,028257 | 0,9729065 |
| ENSG0000( | -0,004496 | 0,0010152 | -2,202241 | 0,0283671 | 0,9729065 |
| ENSG0000( | 0,003174 | 0,0589484 | 2,201771 | 0,0284007 | 0,9729065 |
| ENSG0000( | -0,004124 | -0,254537 | -2,198178 | 0,0286582 | 0,9729065 |
| ENSG0000( | 0,0036071 | 0,0152442 | 2,1975769 | 0,0287015 | 0,9729065 |
| ENSG0000( | 0,0026445 | 0,313158 | 2,1973107 | 0,0287206 | 0,9729065 |
| ENSG0000( | -0,009101 | -0,266088 | -2,195361 | 0,0288615 | 0,9729065 |

| | | | | | |
|---|---|---|---|---|---|
| ENSG0000( | 0,003093 | 0,0378741 | 2,1946412 | 0,0289136 | 0,9729065 |
| ENSG0000( | -0,007681 | -0,439231 | -2,193057 | 0,0290286 | 0,9729065 |
| ENSG0000( | 0,0021188 | 0,5360011 | 2,1930365 | 0,0290301 | 0,9729065 |
| ENSG0000( | 0,0032365 | 0,0899139 | 2,1902302 | 0,0292349 | 0,9729065 |
| ENSG0000( | -0,001953 | 0,0829998 | -2,186345 | 0,0295205 | 0,9729065 |
| ENSG0000( | 0,0087959 | -0,035287 | 2,1860132 | 0,029545 | 0,9729065 |
| ENSG0000( | 0,005898 | -0,007965 | 2,183149 | 0,0297572 | 0,9729065 |
| ENSG0000( | 0,0017478 | -0,079807 | 2,1807559 | 0,0299355 | 0,9729065 |
| ENSG0000( | -0,002827 | -0,040103 | -2,179299 | 0,0300445 | 0,9729065 |
| ENSG0000( | -0,003151 | 0,0900344 | -2,178947 | 0,0300709 | 0,9729065 |
| ENSG0000( | 0,0032846 | 0,031093 | 2,1787483 | 0,0300858 | 0,9729065 |
| ENSG0000( | 0,0032659 | -0,009801 | 2,1773293 | 0,0301924 | 0,9729065 |
| ENSG0000( | 0,0045663 | 0,1271701 | 2,1771996 | 0,0302022 | 0,9729065 |
| ENSG0000( | 0,0013761 | 0,2069042 | 2,1762253 | 0,0302756 | 0,9729065 |
| ENSG0000( | 0,003035 | -0,196794 | 2,1743587 | 0,0304167 | 0,9729065 |
| ENSG0000( | 0,0066416 | 0,0577599 | 2,1729783 | 0,0305214 | 0,9729065 |
| ENSG0000( | -0,003431 | -0,009943 | -2,172261 | 0,0305759 | 0,9729065 |
| ENSG0000( | -0,002566 | 0,131357 | -2,170874 | 0,0306816 | 0,9729065 |
| ENSG0000( | 0,0019721 | 0,0117676 | 2,1673403 | 0,0309523 | 0,9729065 |
| ENSG0000( | 0,0039563 | 0,0455301 | 2,1668134 | 0,0309928 | 0,9729065 |
| ENSG0000( | -0,000693 | -0,010302 | -2,166626 | 0,0310073 | 0,9729065 |
| ENSG0000( | -0,003419 | 0,2972884 | -2,16636 | 0,0310278 | 0,9729065 |
| ENSG0000( | 0,0114861 | 0,22644 | 2,163799 | 0,0312256 | 0,9729065 |
| ENSG0000( | -0,005116 | -0,503198 | -2,163663 | 0,0312361 | 0,9729065 |
| ENSG0000( | -0,003033 | -0,045201 | -2,163619 | 0,0312396 | 0,9729065 |
| ENSG0000( | 0,0033105 | -0,077193 | 2,1597902 | 0,0315375 | 0,9735698 |
| ENSG0000( | 0,0045234 | -0,177868 | 2,1590131 | 0,0315983 | 0,9735698 |
| ENSG0000( | 0,0042014 | 0,0534388 | 2,1585747 | 0,0316326 | 0,9735698 |
| ENSG0000( | -0,002935 | -0,058301 | -2,156189 | 0,03182 | 0,9735698 |
| ENSG0000( | 0,0010801 | 0,0540083 | 2,1542384 | 0,0319739 | 0,9735698 |
| ENSG0000( | -0,003168 | -0,195003 | -2,152393 | 0,0321201 | 0,9735698 |
| ENSG0000( | -0,004754 | -0,008301 | -2,147947 | 0,0324748 | 0,9735698 |
| ENSG0000( | -0,004295 | 0,7340335 | -2,14608 | 0,0326246 | 0,9735698 |
| ENSG0000( | 0,0037088 | 0,1722066 | 2,1453479 | 0,0326836 | 0,9735698 |
| ENSG0000( | 0,0045278 | 0,0459026 | 2,1414923 | 0,0329955 | 0,9735698 |
| ENSG0000( | -0,003843 | 0,2809563 | -2,141115 | 0,0330262 | 0,9735698 |
| ENSG0000( | 0,0026265 | -0,371321 | 2,1344848 | 0,0335691 | 0,9735698 |
| ENSG0000( | -0,00175 | -0,134466 | -2,133499 | 0,0336505 | 0,9735698 |
| ENSG0000( | 0,0031327 | -0,144607 | 2,1334968 | 0,0336506 | 0,9735698 |
| ENSG0000( | 0,0087061 | -0,671998 | 2,1332129 | 0,0336741 | 0,9735698 |
| ENSG0000( | 0,0052349 | -0,05966 | 2,131548 | 0,033812 | 0,9735698 |
| ENSG0000( | 0,0072016 | -0,369082 | 2,1315091 | 0,0338152 | 0,9735698 |
| ENSG0000( | -0,007488 | -0,164675 | -2,130787 | 0,0338752 | 0,9735698 |
| ENSG0000( | 0,0022975 | -0,235925 | 2,126414 | 0,0342402 | 0,9735698 |
| ENSG0000( | 0,0015693 | -0,091635 | 2,1245638 | 0,0343957 | 0,9735698 |
| ENSG0000( | -0,001484 | 0,0628811 | -2,124382 | 0,034411 | 0,9735698 |
| ENSG0000( | -0,005912 | -0,34198 | -2,123745 | 0,0344647 | 0,9735698 |
| ENSG0000( | 0,005459 | 0,3953206 | 2,1222745 | 0,0345889 | 0,9735698 |

| | | | | | |
|---|---|---|---|---|---|
| ENSG0000( | 0,002051 | 0,1130394 | 2,1197983 | 0,0347989 | 0,9735698 |
| ENSG0000( | 0,0036089 | -0,005998 | 2,119788 | 0,0347998 | 0,9735698 |
| ENSG0000( | 0,0051836 | 0,0299365 | 2,1194752 | 0,0348264 | 0,9735698 |
| ENSG0000( | -0,007341 | -0,533172 | -2,119318 | 0,0348398 | 0,9735698 |
| ENSG0000( | 0,0085541 | -0,190881 | 2,1141727 | 0,0352802 | 0,9735698 |
| ENSG0000( | -0,009252 | -0,326961 | -2,113649 | 0,0353253 | 0,9735698 |
| ENSG0000( | -0,002759 | 0,5927553 | -2,113624 | 0,0353275 | 0,9735698 |
| ENSG0000( | 0,0011608 | 0,0478466 | 2,1119272 | 0,0354739 | 0,9735698 |
| ENSG0000( | 0,0013865 | -0,055414 | 2,1111008 | 0,0355454 | 0,9735698 |
| ENSG0000( | -0,001623 | -0,050783 | -2,107542 | 0,0358547 | 0,9735698 |
| ENSG0000( | 0,0073933 | 0,3025736 | 2,1050128 | 0,036076 | 0,9735698 |
| ENSG0000( | -0,002043 | 0,0514435 | -2,104005 | 0,0361645 | 0,9735698 |
| ENSG0000( | 0,0041278 | -0,099335 | 2,1026415 | 0,0362845 | 0,9735698 |
| ENSG0000( | -0,002122 | 0,0653087 | -2,099219 | 0,0365873 | 0,9735698 |
| ENSG0000( | 0,0087638 | 0,1774919 | 2,0987735 | 0,0366268 | 0,9735698 |
| ENSG0000( | 0,003207 | -0,031762 | 2,0978806 | 0,0367062 | 0,9735698 |
| ENSG0000( | -0,002097 | -0,013176 | -2,095863 | 0,0368862 | 0,9735698 |
| ENSG0000( | -0,008335 | 0,3762152 | -2,095339 | 0,0369331 | 0,9735698 |
| ENSG0000( | 0,0024814 | -0,047906 | 2,093214 | 0,0371236 | 0,9735698 |
| ENSG0000( | 0,0040117 | -0,026576 | 2,0918292 | 0,0372483 | 0,9735698 |
| ENSG0000( | 0,0034406 | -0,064922 | 2,0911218 | 0,0373121 | 0,9735698 |
| ENSG0000( | -0,002548 | -0,009006 | -2,089423 | 0,0374658 | 0,9735698 |
| ENSG0000( | 0,0021352 | 0,0288177 | 2,0893539 | 0,037472 | 0,9735698 |
| ENSG0000( | 0,004386 | 0,4537303 | 2,0886476 | 0,037536 | 0,9735698 |
| ENSG0000( | 0,0024658 | 0,2103312 | 2,0885167 | 0,0375479 | 0,9735698 |
| ENSG0000( | 0,0050591 | 0,1169153 | 2,087954 | 0,037599 | 0,9735698 |
| ENSG0000( | 0,0031135 | 0,291738 | 2,0858764 | 0,0377882 | 0,9735698 |
| ENSG0000( | 0,0027818 | 0,8415544 | 2,0835595 | 0,0380001 | 0,9735698 |
| ENSG0000( | 0,0045644 | -0,217794 | 2,0829453 | 0,0380565 | 0,9735698 |
| ENSG0000( | 0,0100501 | -0,680726 | 2,082405 | 0,0381061 | 0,9735698 |
| ENSG0000( | 0,0094614 | 0,6743745 | 2,0804038 | 0,0382905 | 0,9735698 |
| ENSG0000( | 0,0031832 | -0,208654 | 2,0777534 | 0,0385357 | 0,9735698 |
| ENSG0000( | 0,0112609 | -0,636883 | 2,0772824 | 0,0385795 | 0,9735698 |
| ENSG0000( | -0,003831 | 0,2245492 | -2,07615 | 0,0386848 | 0,9735698 |
| ENSG0000( | 0,0071567 | -0,316231 | 2,075851 | 0,0387126 | 0,9735698 |
| ENSG0000( | -0,003352 | 0,104316 | -2,075361 | 0,0387583 | 0,9735698 |
| ENSG0000( | -0,009212 | -0,234426 | -2,069858 | 0,0392744 | 0,9735698 |
| ENSG0000( | -0,004359 | 0,3813907 | -2,068913 | 0,0393637 | 0,9735698 |
| ENSG0000( | -0,003489 | 0,1095797 | -2,06734 | 0,0395125 | 0,9735698 |
| ENSG0000( | -0,00149 | -0,057001 | -2,065898 | 0,0396494 | 0,9735698 |
| ENSG0000( | -0,004605 | -0,160191 | -2,064119 | 0,0398189 | 0,9735698 |
| ENSG0000( | 0,0022382 | -0,090182 | 2,0637125 | 0,0398577 | 0,9735698 |
| ENSG0000( | 0,0012007 | -0,03862 | 2,0635905 | 0,0398693 | 0,9735698 |
| ENSG0000( | 0,0099959 | 0,5669547 | 2,0635604 | 0,0398722 | 0,9735698 |
| ENSG0000( | -0,004018 | 0,0572443 | -2,063256 | 0,0399013 | 0,9735698 |
| ENSG0000( | 0,0096579 | 0,2279227 | 2,060822 | 0,0401346 | 0,9735698 |
| ENSG0000( | -0,006204 | -0,335569 | -2,05835 | 0,0403727 | 0,9735698 |
| ENSG0000( | -0,003692 | -0,089917 | -2,057396 | 0,0404649 | 0,9735698 |

| | | | | | |
|---|---|---|---|---|---|
| ENSG0000( | 0,0051137 | -0,285174 | 2,0572382 | 0,0404801 | 0,9735698 |
| ENSG0000( | 0,00375 | -0,018708 | 2,0568887 | 0,040514 | 0,9735698 |
| ENSG0000( | -0,003215 | -0,145495 | -2,056074 | 0,040593 | 0,9735698 |
| ENSG0000( | -0,003275 | -0,072268 | -2,055189 | 0,0406789 | 0,9735698 |
| ENSG0000( | 0,0042837 | -0,013424 | 2,051442 | 0,0410444 | 0,9735698 |
| ENSG0000( | -0,002401 | -0,08646 | -2,050725 | 0,0411147 | 0,9735698 |
| ENSG0000( | -0,002176 | 0,028985 | -2,047358 | 0,041446 | 0,9735698 |
| ENSG0000( | 0,0022986 | 0,0377926 | 2,0462303 | 0,0415575 | 0,9735698 |
| ENSG0000( | 0,0047733 | 0,1228406 | 2,0451607 | 0,0416634 | 0,9735698 |
| ENSG0000( | -0,003104 | 0,0820985 | -2,044073 | 0,0417714 | 0,9735698 |
| ENSG0000( | 0,0048849 | 0,3240185 | 2,0420584 | 0,0419721 | 0,9735698 |
| ENSG0000( | 0,0041409 | 0,059254 | 2,0411512 | 0,0420627 | 0,9735698 |
| ENSG0000( | 0,0063089 | -0,012465 | 2,0397731 | 0,0422007 | 0,9735698 |
| ENSG0000( | -0,002846 | -0,040063 | -2,039368 | 0,0422413 | 0,9735698 |
| ENSG0000( | 0,005345 | 0,0209532 | 2,0393566 | 0,0422425 | 0,9735698 |
| ENSG0000( | -0,005546 | -0,237944 | -2,038876 | 0,0422907 | 0,9735698 |
| ENSG0000( | -0,003125 | 0,1105252 | -2,036655 | 0,0425144 | 0,9735698 |
| ENSG0000( | 0,0017247 | -0,106776 | 2,0364463 | 0,0425354 | 0,9735698 |
| ENSG0000( | 0,0051471 | -0,188434 | 2,0351992 | 0,0426615 | 0,9735698 |
| ENSG0000( | 0,0044895 | 0,1583172 | 2,0350274 | 0,0426788 | 0,9735698 |
| ENSG0000( | -0,003864 | -0,196103 | -2,03482 | 0,0426998 | 0,9735698 |
| ENSG0000( | 0,0021506 | -0,016915 | 2,034105 | 0,0427723 | 0,9735698 |
| ENSG0000( | 0,0035594 | 0,0967092 | 2,0330007 | 0,0428844 | 0,9735698 |
| ENSG0000( | -0,001825 | 0,0727981 | -2,031538 | 0,0430333 | 0,9735698 |
| ENSG0000( | 0,0037182 | 0,115631 | 2,0314065 | 0,0430467 | 0,9735698 |
| ENSG0000( | 0,002649 | -0,18806 | 2,0311447 | 0,0430734 | 0,9735698 |
| ENSG0000( | -0,004031 | 0,2301045 | -2,030302 | 0,0431595 | 0,9735698 |
| ENSG0000( | 0,0027657 | 0,0301042 | 2,0291561 | 0,0432767 | 0,9735698 |
| ENSG0000( | -0,003646 | -0,529012 | -2,028939 | 0,0432989 | 0,9735698 |
| ENSG0000( | -0,007088 | -0,098063 | -2,028766 | 0,0433167 | 0,9735698 |
| ENSG0000( | 0,0033584 | 0,1783555 | 2,0287599 | 0,0433173 | 0,9735698 |
| ENSG0000( | 0,0073079 | 0,1571853 | 2,0253965 | 0,0436633 | 0,9735698 |
| ENSG0000( | 0,009606 | 0,6137924 | 2,0245529 | 0,0437505 | 0,9735698 |
| ENSG0000( | 0,0042049 | -0,152915 | 2,0239262 | 0,0438153 | 0,9735698 |
| ENSG0000( | 0,0039533 | -0,200415 | 2,0218797 | 0,0440276 | 0,9735698 |
| ENSG0000( | 0,0074568 | -0,312218 | 2,0205084 | 0,0441703 | 0,9735698 |
| ENSG0000( | 0,0060192 | -0,148118 | 2,020483 | 0,0441729 | 0,9735698 |
| ENSG0000( | 0,0033518 | 0,1750783 | 2,0201962 | 0,0442028 | 0,9735698 |
| ENSG0000( | 0,0034564 | 0,0831226 | 2,014924 | 0,0447556 | 0,9735698 |
| ENSG0000( | 0,0043175 | 0,057432 | 2,0144866 | 0,0448017 | 0,9735698 |
| ENSG0000( | -0,005552 | -0,293354 | -2,012038 | 0,0450607 | 0,9735698 |
| ENSG0000( | 0,0106107 | 0,3700348 | 2,0112929 | 0,0451397 | 0,9735698 |
| ENSG0000( | 0,0034566 | -0,022022 | 2,0102514 | 0,0452504 | 0,9735698 |
| ENSG0000( | 0,0054295 | -0,285206 | 2,0100993 | 0,0452666 | 0,9735698 |
| ENSG0000( | -0,000521 | 0,002113 | -2,009982 | 0,045279 | 0,9735698 |
| ENSG0000( | -0,002025 | -0,511952 | -2,009742 | 0,0453046 | 0,9735698 |
| ENSG0000( | -0,002064 | -0,136312 | -2,009476 | 0,0453329 | 0,9735698 |
| ENSG0000( | -0,010114 | 0,0126574 | -2,007036 | 0,0455936 | 0,9735698 |

| | | | | | |
|---|---|---|---|---|---|
| ENSG0000( | 0,0031901 | 0,0784232 | 2,006894 | 0,0456088 | 0,9735698 |
| ENSG0000( | 0,0051188 | 0,2970325 | 2,006575 | 0,0456429 | 0,9735698 |
| ENSG0000( | 0,0043927 | 0,0392398 | 2,0052859 | 0,0457813 | 0,9735698 |
| ENSG0000( | -0,001473 | 0,0266861 | -2,004419 | 0,0458744 | 0,9735698 |
| ENSG0000( | 0,003686 | 0,112484 | 2,0042877 | 0,0458886 | 0,9735698 |
| ENSG0000( | -0,002972 | -0,503376 | -2,002504 | 0,046081 | 0,9750037 |
| ENSG0000( | 0,0019496 | -0,141567 | 2,0007302 | 0,0462729 | 0,9750037 |
| ENSG0000( | 0,0035687 | 0,2009999 | 1,9991239 | 0,0464474 | 0,9750037 |
| ENSG0000( | 0,0021668 | -0,037236 | 1,9984587 | 0,0465198 | 0,9750037 |
| ENSG0000( | 0,0012213 | 0,008608 | 1,9982096 | 0,0465469 | 0,9750037 |
| ENSG0000( | -0,003279 | -0,120718 | -1,995867 | 0,0468027 | 0,9751521 |
| ENSG0000( | 0,0073636 | 0,050627 | 1,9958321 | 0,0468066 | 0,9751521 |
| ENSG0000( | 0,0064397 | -0,078141 | 1,9949022 | 0,0469084 | 0,9751521 |
| ENSG0000( | -0,00231 | -0,173045 | -1,992314 | 0,047193 | 0,9774668 |
| ENSG0000( | 0,0065169 | 0,1977794 | 1,991737 | 0,0472567 | 0,9774668 |
| ENSG0000( | -0,011819 | -0,232597 | -1,987547 | 0,047721 | 0,9788698 |
| ENSG0000( | 0,0025354 | -0,154231 | 1,9866457 | 0,0478214 | 0,9788698 |
| ENSG0000( | -0,007986 | -0,064365 | -1,982417 | 0,0482948 | 0,9788698 |
| ENSG0000( | -0,00731 | -0,122582 | -1,980672 | 0,0484912 | 0,9788698 |
| ENSG0000( | -0,002058 | -0,126203 | -1,980523 | 0,048508 | 0,9788698 |
| ENSG0000( | -0,003164 | -0,036199 | -1,980154 | 0,0485496 | 0,9788698 |
| ENSG0000( | -0,001329 | -0,043131 | -1,979916 | 0,0485766 | 0,9788698 |
| ENSG0000( | 0,0037253 | -0,102157 | 1,9792907 | 0,0486472 | 0,9788698 |
| ENSG0000( | -0,006039 | -0,315087 | -1,97925 | 0,0486518 | 0,9788698 |
| ENSG0000( | -0,004526 | 0,2596726 | -1,979224 | 0,0486547 | 0,9788698 |
| ENSG0000( | 0,0075464 | 0,4586523 | 1,978152 | 0,0487761 | 0,9788698 |
| ENSG0000( | 0,0008468 | 0,3813735 | 1,9764381 | 0,0489707 | 0,9788698 |
| ENSG0000( | 0,0025787 | -0,12982 | 1,9762182 | 0,0489957 | 0,9788698 |
| ENSG0000( | -0,009593 | -0,468049 | -1,975564 | 0,0490703 | 0,9788698 |
| ENSG0000( | 0,0090721 | 0,7599186 | 1,9752711 | 0,0491036 | 0,9788698 |
| ENSG0000( | 0,0026788 | -0,331223 | 1,9738048 | 0,049271 | 0,9798397 |
| ENSG0000( | 0,0035107 | 0,1391027 | 1,9675079 | 0,0499953 | 0,981648 |

| geneID | logFC | AveExpr | t | P.Value | adj.P.Val |
|---|---|---|---|---|---|
| ENSG0000( | -0,004368 | 0,0552769 | -3,490755 | 0,0005498 | 0,9512444 |
| ENSG0000( | 0,0161776 | 0,7222968 | 3,3324548 | 0,0009625 | 0,9512444 |
| ENSG0000( | -0,00728 | 0,0946943 | -3,287465 | 0,0011243 | 0,9512444 |
| ENSG0000( | 0,0057199 | -0,138436 | 3,2616086 | 0,0012283 | 0,9512444 |
| ENSG0000( | -0,00396 | 0,1446057 | -3,195361 | 0,001537 | 0,9512444 |
| ENSG0000( | -0,004432 | -0,220306 | -3,192631 | 0,0015511 | 0,9512444 |
| ENSG0000( | 0,0050378 | 0,0959801 | 3,1354629 | 0,0018763 | 0,9512444 |
| ENSG0000( | -0,009178 | -0,296935 | -3,091923 | 0,002165 | 0,9512444 |
| ENSG0000( | -0,002773 | 0,2624972 | -3,034189 | 0,0026111 | 0,9512444 |
| ENSG0000( | -0,004316 | 0,2028125 | -3,004077 | 0,0028759 | 0,9512444 |
| ENSG0000( | -0,002739 | -0,11562 | -3,000278 | 0,002911 | 0,9512444 |
| ENSG0000( | 0,0024585 | -0,136419 | 2,9916569 | 0,0029921 | 0,9512444 |
| ENSG0000( | 0,0053989 | -0,175995 | 2,9672535 | 0,003233 | 0,9512444 |
| ENSG0000( | -0,001596 | -0,047097 | -2,95887 | 0,0033198 | 0,9512444 |
| ENSG0000( | -0,004793 | -0,144734 | -2,935467 | 0,0035735 | 0,9512444 |
| ENSG0000( | -0,007011 | -0,656663 | -2,923424 | 0,0037109 | 0,9512444 |
| ENSG0000( | -0,003688 | 0,1398046 | -2,914485 | 0,0038159 | 0,9512444 |
| ENSG0000( | -0,004552 | 0,1490001 | -2,905281 | 0,0039269 | 0,9512444 |
| ENSG0000( | 0,0037791 | 0,0863215 | 2,9038525 | 0,0039444 | 0,9512444 |
| ENSG0000( | -0,001997 | -0,020623 | -2,886654 | 0,0041606 | 0,9512444 |
| ENSG0000( | -0,003994 | 0,1535144 | -2,85631 | 0,0045686 | 0,9512444 |
| ENSG0000( | 0,0059276 | -0,191384 | 2,8416793 | 0,004778 | 0,9512444 |
| ENSG0000( | 0,0070816 | 0,097853 | 2,8382634 | 0,0048281 | 0,9512444 |
| ENSG0000( | 0,0053929 | 0,0038463 | 2,8336999 | 0,0048958 | 0,9512444 |
| ENSG0000( | -0,004359 | 0,1279647 | -2,831719 | 0,0049255 | 0,9512444 |
| ENSG0000( | 0,0087885 | -0,136694 | 2,8253178 | 0,0050225 | 0,9512444 |
| ENSG0000( | -0,004922 | -0,014796 | -2,822061 | 0,0050725 | 0,9512444 |
| ENSG0000( | 0,0078214 | 0,2865626 | 2,8114577 | 0,0052385 | 0,9512444 |
| ENSG0000( | -0,017323 | -0,418059 | -2,802212 | 0,0053872 | 0,9512444 |
| ENSG0000( | -0,014476 | 0,4286522 | -2,79447 | 0,0055146 | 0,9512444 |
| ENSG0000( | -0,00562 | -0,09098 | -2,792414 | 0,0055489 | 0,9512444 |
| ENSG0000( | -0,003682 | -0,085538 | -2,776744 | 0,0058168 | 0,9512444 |
| ENSG0000( | 0,0045487 | 0,093469 | 2,7639148 | 0,0060448 | 0,9512444 |
| ENSG0000( | 0,004653 | 0,0589206 | 2,7414604 | 0,0064634 | 0,9512444 |
| ENSG0000( | 0,0017928 | 0,0180798 | 2,7285291 | 0,0067162 | 0,9512444 |
| ENSG0000( | 0,0062085 | 0,0221891 | 2,7169894 | 0,0069493 | 0,9512444 |
| ENSG0000( | 0,0039638 | 0,2794842 | 2,7084969 | 0,0071254 | 0,9512444 |
| ENSG0000( | -0,005835 | -0,204733 | -2,703743 | 0,0072258 | 0,9512444 |
| ENSG0000( | 0,0072074 | -0,204372 | 2,70279 | 0,0072461 | 0,9512444 |
| ENSG0000( | 0,0070337 | 0,5656473 | 2,6960725 | 0,0073904 | 0,9512444 |
| ENSG0000( | 0,0031545 | 0,068341 | 2,6943732 | 0,0074273 | 0,9512444 |
| ENSG0000( | 0,0125014 | -0,754311 | 2,691942 | 0,0074805 | 0,9512444 |
| ENSG0000( | -0,008185 | -1,152406 | -2,679043 | 0,0077681 | 0,9512444 |
| ENSG0000( | -0,002301 | -0,048818 | -2,679022 | 0,0077685 | 0,9512444 |
| ENSG0000( | -0,004772 | 0,3358495 | -2,672599 | 0,0079154 | 0,9512444 |
| ENSG0000( | -0,006367 | 0,1401691 | -2,666656 | 0,0080536 | 0,9512444 |
| ENSG0000( | -0,002736 | -0,043891 | -2,659827 | 0,008215 | 0,9512444 |
| ENSG0000( | 0,0027622 | -0,152552 | 2,6445331 | 0,0085871 | 0,9512444 |

| | | | | | |
|---|---|---|---|---|---|
| ENSG0000( | -0,016727 | -0,568733 | -2,642161 | 0,0086461 | 0,9512444 |
| ENSG0000( | -0,008211 | -0,224945 | -2,641156 | 0,0086712 | 0,9512444 |
| ENSG0000( | 0,0058448 | -0,035921 | 2,6345311 | 0,0088385 | 0,9512444 |
| ENSG0000( | -0,002671 | 0,0069684 | -2,62877 | 0,0089863 | 0,9512444 |
| ENSG0000( | 0,0040711 | -0,033853 | 2,619239 | 0,0092357 | 0,9512444 |
| ENSG0000( | 0,0051492 | -0,368105 | 2,6129204 | 0,0094045 | 0,9512444 |
| ENSG0000( | 0,0050708 | 0,0732172 | 2,6031486 | 0,0096709 | 0,9512444 |
| ENSG0000( | 0,0039243 | 0,1847974 | 2,6025873 | 0,0096864 | 0,9512444 |
| ENSG0000( | -0,00258 | 0,083193 | -2,600661 | 0,0097398 | 0,9512444 |
| ENSG0000( | 0,0033659 | -0,105161 | 2,5880744 | 0,0100952 | 0,9512444 |
| ENSG0000( | 0,0092345 | -0,324033 | 2,5875613 | 0,0101099 | 0,9512444 |
| ENSG0000( | -0,010027 | -0,609068 | -2,586077 | 0,0101526 | 0,9512444 |
| ENSG0000( | 0,0044042 | -0,119718 | 2,5842782 | 0,0102046 | 0,9512444 |
| ENSG0000( | 0,0032807 | 0,0261058 | 2,5784552 | 0,0103746 | 0,9512444 |
| ENSG0000( | 0,0021233 | -0,000934 | 2,5755499 | 0,0104603 | 0,9512444 |
| ENSG0000( | 0,0026421 | -0,084554 | 2,5754969 | 0,0104618 | 0,9512444 |
| ENSG0000( | 0,0045271 | -0,012965 | 2,568342 | 0,0106757 | 0,9512444 |
| ENSG0000( | -0,002441 | 0,0585578 | -2,564027 | 0,0108065 | 0,9512444 |
| ENSG0000( | -0,010644 | 0,2833217 | -2,555333 | 0,0110745 | 0,9512444 |
| ENSG0000( | -0,00546 | -0,09611 | -2,543174 | 0,0114593 | 0,9512444 |
| ENSG0000( | 0,0043187 | 0,0652286 | 2,5420915 | 0,0114941 | 0,9512444 |
| ENSG0000( | 0,0052157 | 0,1639209 | 2,5397736 | 0,011569 | 0,9512444 |
| ENSG0000( | 0,0030258 | 0,0573482 | 2,5333823 | 0,0117778 | 0,9512444 |
| ENSG0000( | -0,007656 | -0,398468 | -2,531182 | 0,0118504 | 0,9512444 |
| ENSG0000( | 0,0041335 | 0,0908761 | 2,5306695 | 0,0118674 | 0,9512444 |
| ENSG0000( | -0,008114 | -0,064903 | -2,525283 | 0,0120471 | 0,9512444 |
| ENSG0000( | 0,0046576 | -0,220113 | 2,5148609 | 0,0124018 | 0,9512444 |
| ENSG0000( | -0,002601 | 0,0637376 | -2,514608 | 0,0124105 | 0,9512444 |
| ENSG0000( | -0,005207 | 0,5509794 | -2,503636 | 0,0127942 | 0,9512444 |
| ENSG0000( | -0,00742 | -0,294078 | -2,500848 | 0,0128933 | 0,9512444 |
| ENSG0000( | 0,0016727 | 0,0078261 | 2,4942413 | 0,013131 | 0,9512444 |
| ENSG0000( | -0,006038 | 0,2216809 | -2,489948 | 0,0132875 | 0,9512444 |
| ENSG0000( | -0,006484 | -0,113914 | -2,489594 | 0,0133005 | 0,9512444 |
| ENSG0000( | -0,003913 | -0,023307 | -2,48366 | 0,0135197 | 0,9512444 |
| ENSG0000( | -0,002386 | -0,095221 | -2,477147 | 0,0137641 | 0,9512444 |
| ENSG0000( | -0,008179 | -0,217648 | -2,47511 | 0,0138413 | 0,9512444 |
| ENSG0000( | -0,005456 | -0,304497 | -2,47115 | 0,0139926 | 0,9512444 |
| ENSG0000( | 0,0084619 | -0,062103 | 2,4694918 | 0,0140563 | 0,9512444 |
| ENSG0000( | 0,003202 | 0,2212667 | 2,4675878 | 0,0141299 | 0,9512444 |
| ENSG0000( | 0,0054516 | 0,1555651 | 2,4660701 | 0,0141887 | 0,9512444 |
| ENSG0000( | -0,005037 | -0,041201 | -2,464158 | 0,0142632 | 0,9512444 |
| ENSG0000( | 0,008646 | 0,9012904 | 2,4611562 | 0,0143808 | 0,9512444 |
| ENSG0000( | 0,0021613 | -0,029934 | 2,4597271 | 0,014437 | 0,9512444 |
| ENSG0000( | -0,010011 | -0,203589 | -2,459556 | 0,0144438 | 0,9512444 |
| ENSG0000( | 0,0028877 | 0,1484133 | 2,4541144 | 0,01466 | 0,9512444 |
| ENSG0000( | 0,0037147 | 0,2294326 | 2,4498566 | 0,0148311 | 0,9512444 |
| ENSG0000( | -0,003058 | -0,02308 | -2,448802 | 0,0148738 | 0,9512444 |
| ENSG0000( | -0,002525 | 0,0297449 | -2,444963 | 0,01503 | 0,9512444 |

| | | | | | |
|---|---|---|---|---|---|
| ENSG0000( | 0,0051699 | -0,002403 | 2,4449245 | 0,0150316 | 0,9512444 |
| ENSG0000( | 0,0058466 | 0,1926672 | 2,4446336 | 0,0150435 | 0,9512444 |
| ENSG0000( | 0,0047028 | -0,140289 | 2,4434838 | 0,0150906 | 0,9512444 |
| ENSG0000( | 0,0035011 | 0,1137446 | 2,4356599 | 0,0154146 | 0,9512444 |
| ENSG0000( | 0,0038216 | -0,003722 | 2,4333487 | 0,0155115 | 0,9512444 |
| ENSG0000( | -0,003044 | 0,2375029 | -2,432842 | 0,0155328 | 0,9512444 |
| ENSG0000( | 0,0028637 | 0,518676 | 2,4319988 | 0,0155684 | 0,9512444 |
| ENSG0000( | -0,003091 | 0,3491753 | -2,427947 | 0,0157401 | 0,9512444 |
| ENSG0000( | -0,008098 | -0,214978 | -2,426539 | 0,0158002 | 0,9512444 |
| ENSG0000( | 0,0027671 | -0,01098 | 2,4222919 | 0,0159826 | 0,9512444 |
| ENSG0000( | -0,003654 | -0,290192 | -2,421389 | 0,0160216 | 0,9512444 |
| ENSG0000( | -0,002869 | 0,0232175 | -2,418603 | 0,0161425 | 0,9512444 |
| ENSG0000( | -0,003058 | 0,0649905 | -2,415217 | 0,0162905 | 0,9512444 |
| ENSG0000( | 0,0030453 | -0,197814 | 2,4150309 | 0,0162987 | 0,9512444 |
| ENSG0000( | -0,00289 | -0,461463 | -2,414409 | 0,0163261 | 0,9512444 |
| ENSG0000( | 0,0097158 | 0,1679121 | 2,4110972 | 0,0164723 | 0,9512444 |
| ENSG0000( | -0,009175 | 0,2687874 | -2,408972 | 0,0165668 | 0,9512444 |
| ENSG0000( | -0,007041 | 0,1165269 | -2,39952 | 0,0169927 | 0,9512444 |
| ENSG0000( | 0,001766 | 0,0523283 | 2,3994884 | 0,0169941 | 0,9512444 |
| ENSG0000( | -0,001944 | 0,2313529 | -2,398632 | 0,0170331 | 0,9512444 |
| ENSG0000( | 0,0048496 | 0,1443105 | 2,384843 | 0,0176731 | 0,9512444 |
| ENSG0000( | 0,0027213 | 0,0461011 | 2,3804712 | 0,0178804 | 0,9512444 |
| ENSG0000( | -0,00197 | 0,0246703 | -2,380092 | 0,0178985 | 0,9512444 |
| ENSG0000( | 0,0050187 | 0,2353767 | 2,3787938 | 0,0179605 | 0,9512444 |
| ENSG0000( | -0,007357 | -0,086886 | -2,377257 | 0,0180342 | 0,9512444 |
| ENSG0000( | 0,0033859 | -0,017001 | 2,3769396 | 0,0180494 | 0,9512444 |
| ENSG0000( | 0,0062515 | 0,1409968 | 2,3705535 | 0,0183586 | 0,9512444 |
| ENSG0000( | -0,004258 | -0,487532 | -2,364537 | 0,0186541 | 0,9512444 |
| ENSG0000( | 0,0121892 | 1,1106405 | 2,3642698 | 0,0186673 | 0,9512444 |
| ENSG0000( | 0,0036588 | 0,0784249 | 2,3608466 | 0,0188374 | 0,9512444 |
| ENSG0000( | 0,0059597 | -0,249304 | 2,3576423 | 0,0189979 | 0,9512444 |
| ENSG0000( | 0,0043875 | 0,121941 | 2,3575451 | 0,0190028 | 0,9512444 |
| ENSG0000( | -0,009665 | -0,611809 | -2,3572 | 0,0190201 | 0,9512444 |
| ENSG0000( | -0,003087 | 0,0439181 | -2,352945 | 0,0192353 | 0,9512444 |
| ENSG0000( | -0,005909 | 0,3196418 | -2,352069 | 0,0192798 | 0,9512444 |
| ENSG0000( | 0,0027093 | 0,0928496 | 2,3420227 | 0,0197974 | 0,9512444 |
| ENSG0000( | -0,002457 | -0,044918 | -2,340193 | 0,019893 | 0,9512444 |
| ENSG0000( | -0,003698 | 0,0822574 | -2,336646 | 0,0200794 | 0,9512444 |
| ENSG0000( | -0,002178 | 0,0329721 | -2,334316 | 0,0202027 | 0,9512444 |
| ENSG0000( | 0,0028642 | 0,0004179 | 2,3337289 | 0,0202338 | 0,9512444 |
| ENSG0000( | -0,002619 | 0,0316698 | -2,328675 | 0,0205039 | 0,9512444 |
| ENSG0000( | 0,0051595 | 0,1517077 | 2,3276721 | 0,0205578 | 0,9512444 |
| ENSG0000( | 0,0044075 | -0,077529 | 2,326286 | 0,0206326 | 0,9512444 |
| ENSG0000( | 0,0033733 | -0,237972 | 2,326075 | 0,020644 | 0,9512444 |
| ENSG0000( | 0,0043617 | 0,1015903 | 2,3193613 | 0,0210098 | 0,9512444 |
| ENSG0000( | -0,002117 | 0,0292412 | -2,314856 | 0,0212585 | 0,9512444 |
| ENSG0000( | 0,004712 | 0,3066208 | 2,3134274 | 0,0213378 | 0,9512444 |
| ENSG0000( | 0,0049769 | 0,0624082 | 2,3073478 | 0,0216785 | 0,9512444 |

| | | | | | |
|---|---|---|---|---|---|
| ENSG0000( | -0,001899 | 0,2362961 | -2,303398 | 0,0219025 | 0,9512444 |
| ENSG0000( | 0,0021253 | 0,2354214 | 2,2974527 | 0,0222433 | 0,9512444 |
| ENSG0000( | 0,0049359 | -0,102634 | 2,2965934 | 0,0222929 | 0,9512444 |
| ENSG0000( | 0,0056856 | 0,374132 | 2,2941426 | 0,022435 | 0,9512444 |
| ENSG0000( | -0,003654 | 0,1964471 | -2,290995 | 0,0226187 | 0,9512444 |
| ENSG0000( | -0,006723 | -0,154693 | -2,28719 | 0,0228425 | 0,9512444 |
| ENSG0000( | -0,003662 | 0,1085658 | -2,285874 | 0,0229203 | 0,9512444 |
| ENSG0000( | 0,0040905 | 0,0389714 | 2,2844258 | 0,0230063 | 0,9512444 |
| ENSG0000( | 0,006277 | -0,266555 | 2,2836052 | 0,0230551 | 0,9512444 |
| ENSG0000( | 0,0040132 | -0,251096 | 2,2801078 | 0,0232642 | 0,9512444 |
| ENSG0000( | -0,005462 | -0,176495 | -2,279309 | 0,0233122 | 0,9512444 |
| ENSG0000( | -0,001576 | -0,030637 | -2,278509 | 0,0233603 | 0,9512444 |
| ENSG0000( | 0,0029854 | -0,041772 | 2,2764632 | 0,0234838 | 0,9512444 |
| ENSG0000( | -0,00256 | -0,006489 | -2,272292 | 0,0237374 | 0,9512444 |
| ENSG0000( | -0,004377 | -0,086304 | -2,269665 | 0,0238983 | 0,9512444 |
| ENSG0000( | 0,0022959 | -0,19994 | 2,2687306 | 0,0239558 | 0,9512444 |
| ENSG0000( | -0,012199 | 0,4589613 | -2,267528 | 0,02403 | 0,9512444 |
| ENSG0000( | -0,004184 | 1,4725094 | -2,266259 | 0,0241084 | 0,9512444 |
| ENSG0000( | 0,0027858 | 0,0377291 | 2,2650435 | 0,0241838 | 0,9512444 |
| ENSG0000( | 0,0084593 | -0,491732 | 2,2643639 | 0,024226 | 0,9512444 |
| ENSG0000( | -0,003595 | -0,090975 | -2,263027 | 0,0243093 | 0,9512444 |
| ENSG0000( | 0,0056087 | -0,312357 | 2,2619053 | 0,0243793 | 0,9512444 |
| ENSG0000( | 0,0043102 | 0,2403515 | 2,2611885 | 0,0244242 | 0,9512444 |
| ENSG0000( | -0,002792 | -0,056225 | -2,258479 | 0,0245943 | 0,9512444 |
| ENSG0000( | 0,0033568 | -0,076727 | 2,2518195 | 0,025017 | 0,9512444 |
| ENSG0000( | -0,002706 | 0,0624768 | -2,251653 | 0,0250277 | 0,9512444 |
| ENSG0000( | 0,003028 | 0,1777568 | 2,2498137 | 0,0251455 | 0,9512444 |
| ENSG0000( | 0,0026635 | 0,1165803 | 2,2492832 | 0,0251796 | 0,9512444 |
| ENSG0000( | 0,0045597 | 0,4320812 | 2,2457677 | 0,0254066 | 0,9512444 |
| ENSG0000( | -0,003852 | 0,7765107 | -2,241662 | 0,0256739 | 0,9512444 |
| ENSG0000( | -0,001556 | -0,016505 | -2,237315 | 0,0259595 | 0,9512444 |
| ENSG0000( | -0,007907 | 1,0590295 | -2,236966 | 0,0259825 | 0,9512444 |
| ENSG0000( | -0,001594 | -0,054184 | -2,236563 | 0,0260092 | 0,9512444 |
| ENSG0000( | -0,002759 | 0,0567439 | -2,235484 | 0,0260806 | 0,9512444 |
| ENSG0000( | -0,004171 | -0,04917 | -2,232301 | 0,0262924 | 0,9512444 |
| ENSG0000( | -0,009368 | -0,404322 | -2,228173 | 0,0265692 | 0,9512444 |
| ENSG0000( | 0,0094625 | -0,669071 | 2,2277007 | 0,0266011 | 0,9512444 |
| ENSG0000( | -0,008278 | -0,280698 | -2,221619 | 0,027014 | 0,9512444 |
| ENSG0000( | -0,006008 | 0,3903186 | -2,219073 | 0,0271885 | 0,9512444 |
| ENSG0000( | 0,0061335 | 0,4480378 | 2,2190638 | 0,0271892 | 0,9512444 |
| ENSG0000( | -0,002391 | 0,0475367 | -2,217558 | 0,0272929 | 0,9512444 |
| ENSG0000( | -0,002316 | -0,106507 | -2,214823 | 0,027482 | 0,9512444 |
| ENSG0000( | 0,00284 | 0,8592729 | 2,2139478 | 0,0275428 | 0,9512444 |
| ENSG0000( | -0,010973 | -0,372422 | -2,20649 | 0,0280654 | 0,9512444 |
| ENSG0000( | 0,0050812 | 0,1700535 | 2,2045272 | 0,0282044 | 0,9512444 |
| ENSG0000( | 0,0022732 | -0,052279 | 2,2045114 | 0,0282055 | 0,9512444 |
| ENSG0000( | 0,0051198 | -0,257503 | 2,2018482 | 0,028395 | 0,9512444 |
| ENSG0000( | -0,016436 | -2,402554 | -2,199567 | 0,0285582 | 0,9512444 |

| | | | | | |
|---|---|---|---|---|---|
| ENSG0000( | -0,002965 | 0,0524201 | -2,198768 | 0,0286156 | 0,9512444 |
| ENSG0000( | 0,0087112 | -0,246842 | 2,1933196 | 0,0290094 | 0,9512444 |
| ENSG0000( | 0,0075391 | 0,2936658 | 2,1924176 | 0,029075 | 0,9512444 |
| ENSG0000( | 0,0079112 | -0,393926 | 2,1902399 | 0,029234 | 0,9512444 |
| ENSG0000( | 0,0030835 | -0,014413 | 2,1888682 | 0,0293346 | 0,9512444 |
| ENSG0000( | -0,002194 | 0,0411921 | -2,187514 | 0,0294341 | 0,9512444 |
| ENSG0000( | 0,006988 | 0,1162334 | 2,187414 | 0,0294415 | 0,9512444 |
| ENSG0000( | 0,0037399 | -0,168256 | 2,1822445 | 0,0298243 | 0,9512444 |
| ENSG0000( | -0,003189 | -0,169217 | -2,181563 | 0,0298751 | 0,9512444 |
| ENSG0000( | -0,003807 | -0,19403 | -2,17937 | 0,030039 | 0,9512444 |
| ENSG0000( | 0,0048146 | 0,0735923 | 2,1771424 | 0,0302063 | 0,9512444 |
| ENSG0000( | -0,004837 | 0,3872611 | -2,176029 | 0,0302902 | 0,9512444 |
| ENSG0000( | 0,0096493 | 0,7815303 | 2,175961 | 0,0302954 | 0,9512444 |
| ENSG0000( | 0,0048328 | -0,269941 | 2,1752179 | 0,0303515 | 0,9512444 |
| ENSG0000( | 0,0059449 | 0,2717448 | 2,1747531 | 0,0303867 | 0,9512444 |
| ENSG0000( | -0,003501 | 0,330784 | -2,174062 | 0,030439 | 0,9512444 |
| ENSG0000( | -0,002166 | 0,1139631 | -2,17334 | 0,0304938 | 0,9512444 |
| ENSG0000( | 0,0010442 | 0,0067732 | 2,1729853 | 0,0305207 | 0,9512444 |
| ENSG0000( | -0,003961 | 0,2749997 | -2,169689 | 0,030772 | 0,9512444 |
| ENSG0000( | -0,007039 | -0,166892 | -2,169221 | 0,0308078 | 0,9512444 |
| ENSG0000( | -0,002338 | -0,110971 | -2,16328 | 0,0312657 | 0,9512444 |
| ENSG0000( | 0,0043484 | 0,8867564 | 2,159367 | 0,0315704 | 0,9512444 |
| ENSG0000( | -0,004789 | -0,003345 | -2,157855 | 0,0316889 | 0,9512444 |
| ENSG0000( | -0,00335 | -0,137804 | -2,155178 | 0,0318996 | 0,9512444 |
| ENSG0000( | 0,0035062 | -0,125427 | 2,1534019 | 0,03204 | 0,9512444 |
| ENSG0000( | -0,003404 | 0,0741836 | -2,152776 | 0,0320896 | 0,9512444 |
| ENSG0000( | 0,0070509 | -0,100707 | 2,1523684 | 0,0321219 | 0,9512444 |
| ENSG0000( | -0,007406 | 0,7759442 | -2,150128 | 0,0323002 | 0,9512444 |
| ENSG0000( | 0,0032293 | -0,153974 | 2,1492327 | 0,0323717 | 0,9512444 |
| ENSG0000( | -0,004622 | 0,3930971 | -2,146063 | 0,0326258 | 0,9512444 |
| ENSG0000( | 0,005284 | 0,1629918 | 2,1440431 | 0,0327887 | 0,9512444 |
| ENSG0000( | -0,005331 | 0,1079091 | -2,143714 | 0,0328153 | 0,9512444 |
| ENSG0000( | 0,003128 | 0,7238295 | 2,1427816 | 0,0328908 | 0,9512444 |
| ENSG0000( | 0,0016004 | -0,111015 | 2,1414692 | 0,0329973 | 0,9512444 |
| ENSG0000( | -0,003929 | 0,2072343 | -2,135538 | 0,0334822 | 0,9512444 |
| ENSG0000( | 0,0042096 | 0,233198 | 2,1354401 | 0,0334902 | 0,9512444 |
| ENSG0000( | 0,003743 | 0,478199 | 2,1353955 | 0,0334939 | 0,9512444 |
| ENSG0000( | 0,0019306 | 0,1674506 | 2,1348579 | 0,0335382 | 0,9512444 |
| ENSG0000( | 0,0046995 | -0,331981 | 2,133281 | 0,0336683 | 0,9512444 |
| ENSG0000( | -0,00618 | -0,334375 | -2,132108 | 0,0337654 | 0,9512444 |
| ENSG0000( | -0,002975 | -0,295113 | -2,129796 | 0,0339574 | 0,9512444 |
| ENSG0000( | 0,0066425 | 0,7152882 | 2,1295675 | 0,0339765 | 0,9512444 |
| ENSG0000( | -0,008475 | 0,6807191 | -2,125487 | 0,0343179 | 0,9512444 |
| ENSG0000( | 0,0038024 | -0,092723 | 2,1252232 | 0,0343401 | 0,9512444 |
| ENSG0000( | 0,0082691 | -0,550201 | 2,1250043 | 0,0343585 | 0,9512444 |
| ENSG0000( | -0,003152 | -0,181152 | -2,124369 | 0,0344119 | 0,9512444 |
| ENSG0000( | 0,0029675 | -0,145602 | 2,1201742 | 0,0347668 | 0,9570335 |
| ENSG0000( | 0,0025959 | 0,0027006 | 2,116199 | 0,035106 | 0,9623442 |

| | | | | | |
|---|---|---|---|---|---|
| ENSG0000( | -0,001884 | -0,03334 | -2,107786 | 0,0358333 | 0,9679528 |
| ENSG0000( | 0,0089599 | 0,1140736 | 2,1068493 | 0,035915 | 0,9679528 |
| ENSG0000( | -0,001903 | -0,008537 | -2,105474 | 0,0360354 | 0,9679528 |
| ENSG0000( | 0,005163 | 0,0610184 | 2,1044233 | 0,0361275 | 0,9679528 |
| ENSG0000( | 0,0099024 | -0,073878 | 2,1022514 | 0,0363187 | 0,9679528 |
| ENSG0000( | 0,0089782 | -0,238114 | 2,0996036 | 0,0365529 | 0,9679528 |
| ENSG0000( | 0,0066811 | 0,187741 | 2,0978306 | 0,0367105 | 0,9679528 |
| ENSG0000( | 0,0048683 | 0,0975636 | 2,0971216 | 0,0367736 | 0,9679528 |
| ENSG0000( | -0,001896 | -0,024305 | -2,096177 | 0,036858 | 0,9679528 |
| ENSG0000( | -0,002373 | -0,098456 | -2,094131 | 0,0370411 | 0,9679528 |
| ENSG0000( | 0,002178 | -0,053725 | 2,0910222 | 0,0373209 | 0,9679528 |
| ENSG0000( | -0,002481 | -0,060465 | -2,090355 | 0,0373812 | 0,9679528 |
| ENSG0000( | -0,005281 | 0,2315068 | -2,089429 | 0,037465 | 0,9679528 |
| ENSG0000( | 0,0048693 | -0,09444 | 2,0878132 | 0,0376116 | 0,9679528 |
| ENSG0000( | -0,002716 | -0,015378 | -2,086709 | 0,0377121 | 0,9679528 |
| ENSG0000( | -0,002778 | 0,0422653 | -2,085585 | 0,0378146 | 0,9679528 |
| ENSG0000( | 0,0065593 | -0,253233 | 2,0851686 | 0,0378527 | 0,9679528 |
| ENSG0000( | 0,0017348 | -0,012152 | 2,0811072 | 0,0382254 | 0,9679528 |
| ENSG0000( | 0,005589 | 0,4721543 | 2,0772718 | 0,0385803 | 0,9679528 |
| ENSG0000( | -0,003836 | 0,1771069 | -2,077068 | 0,0385992 | 0,9679528 |
| ENSG0000( | -0,002774 | 1,0668816 | -2,075061 | 0,0387861 | 0,9679528 |
| ENSG0000( | -0,00223 | -0,029306 | -2,075015 | 0,0387904 | 0,9679528 |
| ENSG0000( | 0,0088233 | 0,3990088 | 2,0746575 | 0,0388238 | 0,9679528 |
| ENSG0000( | 0,0019588 | 0,0067833 | 2,0744658 | 0,0388417 | 0,9679528 |
| ENSG0000( | 0,0034329 | 0,21646 | 2,069768 | 0,0392827 | 0,9703474 |
| ENSG0000( | 0,0031316 | 0,0369582 | 2,0683582 | 0,0394159 | 0,9703474 |
| ENSG0000( | 0,0037872 | -0,162519 | 2,0629368 | 0,0399317 | 0,9703474 |
| ENSG0000( | -0,003881 | -0,053478 | -2,062596 | 0,0399643 | 0,9703474 |
| ENSG0000( | 0,0038042 | 0,1432393 | 2,0607656 | 0,0401398 | 0,9703474 |
| ENSG0000( | -0,001968 | 0,0018536 | -2,060667 | 0,0401493 | 0,9703474 |
| ENSG0000( | -0,003263 | 0,1584812 | -2,060583 | 0,0401574 | 0,9703474 |
| ENSG0000( | 0,0094431 | -0,569408 | 2,0574719 | 0,0404574 | 0,9703474 |
| ENSG0000( | -0,001821 | 0,1233847 | -2,057365 | 0,0404677 | 0,9703474 |
| ENSG0000( | -0,002822 | 0,0839523 | -2,05536 | 0,0406621 | 0,9703474 |
| ENSG0000( | -0,003439 | -0,383944 | -2,051991 | 0,0409905 | 0,9703474 |
| ENSG0000( | -0,004674 | -0,138627 | -2,050491 | 0,0411374 | 0,9703474 |
| ENSG0000( | 0,0048943 | 0,1853563 | 2,0484938 | 0,0413338 | 0,9703474 |
| ENSG0000( | -0,002852 | -0,285318 | -2,046403 | 0,0415402 | 0,9703474 |
| ENSG0000( | -0,010886 | 0,7553576 | -2,040306 | 0,0421472 | 0,9703474 |
| ENSG0000( | 0,0016262 | -0,264593 | 2,0396763 | 0,0422103 | 0,9703474 |
| ENSG0000( | -0,004595 | -0,11761 | -2,039424 | 0,0422356 | 0,9703474 |
| ENSG0000( | -0,004842 | 0,1488034 | -2,037259 | 0,0424533 | 0,9703474 |
| ENSG0000( | -0,003017 | 0,3906828 | -2,035811 | 0,0425994 | 0,9703474 |
| ENSG0000( | -0,003363 | 0,238945 | -2,035334 | 0,0426476 | 0,9703474 |
| ENSG0000( | -0,009397 | 0,0128087 | -2,032865 | 0,0428981 | 0,9703474 |
| ENSG0000( | -0,002292 | 0,0211027 | -2,032854 | 0,0428992 | 0,9703474 |
| ENSG0000( | -0,003122 | -0,202992 | -2,031356 | 0,0430517 | 0,9703474 |
| ENSG0000( | 0,0048118 | -0,313155 | 2,0303552 | 0,0431539 | 0,9703474 |

| | | | | | |
|---|---|---|---|---|---|
| ENSG00000( | 0,0035731 | 0,2410255 | 2,0275239 | 0,043444 | 0,9703474 |
| ENSG00000( | 0,0023809 | 0,1245763 | 2,0269197 | 0,0435062 | 0,9703474 |
| ENSG00000( | 0,0046953 | 0,3643245 | 2,0263163 | 0,0435683 | 0,9703474 |
| ENSG00000( | -0,004738 | -0,20532 | -2,025837 | 0,0436177 | 0,9703474 |
| ENSG00000( | 0,0100459 | 0,6294721 | 2,0258334 | 0,0436181 | 0,9703474 |
| ENSG00000( | 0,0043788 | -0,217802 | 2,0247206 | 0,0437329 | 0,9703474 |
| ENSG00000( | -0,002651 | 0,4449681 | -2,024609 | 0,0437445 | 0,9703474 |
| ENSG00000( | 0,002563 | 0,0896029 | 2,0245962 | 0,0437458 | 0,9703474 |
| ENSG00000( | -0,00399 | -0,106103 | -2,024024 | 0,043805 | 0,9703474 |
| ENSG00000( | -0,004222 | -0,062699 | -2,021125 | 0,0441059 | 0,9737338 |
| ENSG00000( | -0,005763 | -0,045837 | -2,012444 | 0,0450175 | 0,9888604 |
| ENSG00000( | 0,003382 | -0,131241 | 2,0109141 | 0,0451798 | 0,9888604 |
| ENSG00000( | 0,0069676 | 0,2716367 | 2,0103288 | 0,045242 | 0,9888604 |
| ENSG00000( | -0,004116 | 0,5063487 | -2,00522 | 0,0457881 | 0,9888748 |
| ENSG00000( | 0,0028832 | -0,056815 | 2,0022062 | 0,046113 | 0,9888748 |
| ENSG00000( | 0,0053887 | 0,1807174 | 1,9971581 | 0,0466614 | 0,9888748 |
| ENSG00000( | 0,0037888 | -0,009915 | 1,9968745 | 0,0466924 | 0,9888748 |
| ENSG00000( | 0,0042246 | -0,386845 | 1,9934198 | 0,0470711 | 0,9888748 |
| ENSG00000( | -0,004231 | 0,4830289 | -1,991917 | 0,0472366 | 0,9888748 |
| ENSG00000( | 0,0045978 | 0,1281808 | 1,9916528 | 0,0472658 | 0,9888748 |
| ENSG00000( | -0,003431 | 0,2108524 | -1,990727 | 0,0473681 | 0,9888748 |
| ENSG00000( | -0,004685 | 0,6936023 | -1,987813 | 0,0476912 | 0,9888748 |
| ENSG00000( | -0,002173 | 0,157969 | -1,986557 | 0,0478311 | 0,9888748 |
| ENSG00000( | 0,0047788 | 0,1447665 | 1,9851622 | 0,0479868 | 0,9888748 |
| ENSG00000( | 0,002465 | 0,0724013 | 1,9831263 | 0,0482149 | 0,9888748 |
| ENSG00000( | 0,0034273 | -0,080209 | 1,981743 | 0,0483704 | 0,9888748 |
| ENSG00000( | -0,00466 | 0,5301915 | -1,981246 | 0,0484263 | 0,9888748 |
| ENSG00000( | -0,002207 | 0,0218934 | -1,981177 | 0,0484341 | 0,9888748 |
| ENSG00000( | -0,004563 | -0,051047 | -1,978376 | 0,0487506 | 0,9888748 |
| ENSG00000( | -0,007782 | -0,249345 | -1,978042 | 0,0487885 | 0,9888748 |
| ENSG00000( | -0,003587 | -0,014649 | -1,975321 | 0,0490978 | 0,9888748 |
| ENSG00000( | -0,004564 | -0,094045 | -1,975189 | 0,0491127 | 0,9888748 |
| ENSG00000( | 0,0011254 | 0,1342947 | 1,9748744 | 0,0491487 | 0,9888748 |
| ENSG00000( | -0,002368 | 0,1332695 | -1,974069 | 0,0492406 | 0,9888748 |
| ENSG00000( | -0,004167 | 0,1399335 | -1,971293 | 0,0495587 | 0,9888748 |
| ENSG00000( | -0,002151 | -0,233526 | -1,970482 | 0,049652 | 0,9888748 |
| ENSG00000( | -0,008248 | -1,36829 | -1,968651 | 0,049863 | 0,9888748 |
| ENSG00000( | 0,0031712 | -0,07715 | 1,9681362 | 0,0499224 | 0,9888748 |

| geneID | logFC | AveExpr | t | P.Value | adj.P.Val |
|---|---|---|---|---|---|
| ENSG0000 | 0,007447 | -0,26552 | 3,862047 | 0,000137 | 0,797999 |
| ENSG0000 | 0,002776 | 0,041767 | 3,53209 | 0,000475 | 0,797999 |
| ENSG0000 | -0,008561 | -0,142115 | -3,455654 | 0,000625 | 0,797999 |
| ENSG0000 | 0,005116 | -0,246029 | 3,412255 | 0,000729 | 0,797999 |
| ENSG0000 | -0,005827 | 0,206865 | -3,323629 | 0,000995 | 0,797999 |
| ENSG0000 | -0,004791 | -0,255457 | -3,315669 | 0,001022 | 0,797999 |
| ENSG0000 | -0,002611 | 0,114419 | -3,20185 | 0,001507 | 0,797999 |
| ENSG0000 | 0,002169 | -0,034241 | 3,198948 | 0,001521 | 0,797999 |
| ENSG0000 | -0,007287 | 0,178368 | -3,192995 | 0,001552 | 0,797999 |
| ENSG0000 | -0,0027 | 0,02619 | -3,184158 | 0,001599 | 0,797999 |
| ENSG0000 | -0,003069 | -0,101266 | -3,141188 | 0,001844 | 0,797999 |
| ENSG0000 | -0,01462 | -0,012685 | -3,09823 | 0,002124 | 0,797999 |
| ENSG0000 | 0,00845 | 0,511053 | 3,096681 | 0,002135 | 0,797999 |
| ENSG0000 | -0,002152 | -0,037161 | -3,091322 | 0,002173 | 0,797999 |
| ENSG0000 | -0,003591 | 0,081651 | -3,088356 | 0,002194 | 0,797999 |
| ENSG0000 | -0,004345 | -0,082859 | -3,082511 | 0,002236 | 0,797999 |
| ENSG0000 | 0,004013 | 0,116597 | 3,059061 | 0,002413 | 0,797999 |
| ENSG0000 | 0,00651 | -0,258181 | 3,039292 | 0,002572 | 0,797999 |
| ENSG0000 | -0,005065 | 0,077631 | -3,032164 | 0,002632 | 0,797999 |
| ENSG0000 | 0,005135 | 0,102511 | 3,025552 | 0,002689 | 0,797999 |
| ENSG0000 | -0,004927 | -0,568903 | -3,008133 | 0,002843 | 0,797999 |
| ENSG0000 | -0,002751 | 0,086049 | -2,978737 | 0,003122 | 0,797999 |
| ENSG0000 | 0,007631 | -0,22583 | 2,973326 | 0,003176 | 0,797999 |
| ENSG0000 | 0,004531 | 0,305219 | 2,958645 | 0,003327 | 0,797999 |
| ENSG0000 | -0,001698 | -0,077165 | -2,958621 | 0,003327 | 0,797999 |
| ENSG0000 | 0,004225 | -0,026409 | 2,937573 | 0,003555 | 0,797999 |
| ENSG0000 | 0,00343 | 0,222784 | 2,928855 | 0,003653 | 0,797999 |
| ENSG0000 | 0,005779 | 0,227879 | 2,926886 | 0,003676 | 0,797999 |
| ENSG0000 | 0,009351 | -0,037848 | 2,908609 | 0,003892 | 0,797999 |
| ENSG0000 | 0,009919 | 0,304682 | 2,905458 | 0,00393 | 0,797999 |
| ENSG0000 | -0,001996 | 0,258474 | -2,904657 | 0,00394 | 0,797999 |
| ENSG0000 | 0,001603 | -0,071941 | 2,894648 | 0,004064 | 0,797999 |
| ENSG0000 | 0,003005 | 0,02105 | 2,886457 | 0,004168 | 0,797999 |
| ENSG0000 | 0,004036 | -0,166016 | 2,882836 | 0,004215 | 0,797999 |
| ENSG0000 | 0,004979 | -0,35652 | 2,874234 | 0,004329 | 0,797999 |
| ENSG0000 | 0,005877 | -0,723037 | 2,862645 | 0,004486 | 0,797999 |
| ENSG0000 | -0,005694 | 0,205863 | -2,860645 | 0,004514 | 0,797999 |
| ENSG0000 | -0,002983 | 0,042892 | -2,853621 | 0,004612 | 0,797999 |
| ENSG0000 | -0,004853 | -0,267937 | -2,850392 | 0,004658 | 0,797999 |
| ENSG0000 | 0,008982 | -0,639703 | 2,847694 | 0,004697 | 0,797999 |
| ENSG0000 | 0,002451 | -0,017221 | 2,845343 | 0,00473 | 0,797999 |
| ENSG0000 | -0,004475 | 0,212177 | -2,830343 | 0,004952 | 0,797999 |
| ENSG0000 | 0,002528 | -0,007514 | 2,826965 | 0,005003 | 0,797999 |
| ENSG0000 | 0,005212 | 0,225559 | 2,821987 | 0,00508 | 0,797999 |
| ENSG0000 | -0,004741 | -0,327465 | -2,821387 | 0,005089 | 0,797999 |
| ENSG0000 | 0,006005 | -0,319965 | 2,818971 | 0,005126 | 0,797999 |
| ENSG0000 | -0,002513 | 0,203577 | -2,812268 | 0,005232 | 0,797999 |
| ENSG0000 | 0,010358 | -0,140689 | 2,80591 | 0,005333 | 0,797999 |

| | | | | | |
|---|---|---|---|---|---|
| ENSG0000 | -0,005482 | 0,110258 | -2,802575 | 0,005387 | 0,797999 |
| ENSG0000 | -0,004471 | 0,489109 | -2,798331 | 0,005457 | 0,797999 |
| ENSG0000 | -0,002044 | 0,126429 | -2,787938 | 0,005631 | 0,797999 |
| ENSG0000 | 0,00292 | -0,181956 | 2,786531 | 0,005655 | 0,797999 |
| ENSG0000 | -0,008774 | -1,313865 | -2,775649 | 0,005842 | 0,797999 |
| ENSG0000 | 0,004095 | 0,245198 | 2,775087 | 0,005852 | 0,797999 |
| ENSG0000 | -0,002307 | 0,095161 | -2,772404 | 0,0059 | 0,797999 |
| ENSG0000 | 0,005562 | -0,380417 | 2,75568 | 0,006202 | 0,797999 |
| ENSG0000 | 0,001698 | -0,057958 | 2,750132 | 0,006305 | 0,797999 |
| ENSG0000 | 0,001697 | 0,053631 | 2,741776 | 0,006464 | 0,797999 |
| ENSG0000 | -0,003974 | -0,317068 | -2,738018 | 0,006537 | 0,797999 |
| ENSG0000 | -0,003148 | 0,124588 | -2,736499 | 0,006566 | 0,797999 |
| ENSG0000 | 0,005648 | 0,220626 | 2,736158 | 0,006573 | 0,797999 |
| ENSG0000 | -0,002574 | 0,05944 | -2,727479 | 0,006744 | 0,797999 |
| ENSG0000 | -0,002294 | -0,121114 | -2,725523 | 0,006783 | 0,797999 |
| ENSG0000 | 0,005801 | 0,069392 | 2,724788 | 0,006798 | 0,797999 |
| ENSG0000 | -0,001276 | 0,058139 | -2,720739 | 0,00688 | 0,797999 |
| ENSG0000 | -0,00294 | -0,011814 | -2,720079 | 0,006893 | 0,797999 |
| ENSG0000 | 0,006564 | -0,190595 | 2,717963 | 0,006936 | 0,797999 |
| ENSG0000 | -0,001914 | 0,096091 | -2,714918 | 0,006999 | 0,797999 |
| ENSG0000 | 0,004157 | -0,032099 | 2,712176 | 0,007056 | 0,797999 |
| ENSG0000 | -0,00312 | -0,041071 | -2,7072 | 0,00716 | 0,797999 |
| ENSG0000 | -0,001518 | -0,072656 | -2,704275 | 0,007222 | 0,797999 |
| ENSG0000 | -0,010797 | 0,418726 | -2,702158 | 0,007267 | 0,797999 |
| ENSG0000 | -0,005343 | 0,329454 | -2,701469 | 0,007282 | 0,797999 |
| ENSG0000 | 0,002687 | -0,114303 | 2,698519 | 0,007345 | 0,797999 |
| ENSG0000 | -0,003034 | -0,208561 | -2,696201 | 0,007395 | 0,797999 |
| ENSG0000 | -0,001641 | -0,134666 | -2,692676 | 0,007472 | 0,797999 |
| ENSG0000 | -0,006557 | -0,148673 | -2,689795 | 0,007535 | 0,797999 |
| ENSG0000 | 0,004228 | 0,166875 | 2,68808 | 0,007573 | 0,797999 |
| ENSG0000 | -0,009812 | 0,651691 | -2,685128 | 0,007639 | 0,797999 |
| ENSG0000 | 0,003536 | 0,429193 | 2,679741 | 0,00776 | 0,800523 |
| ENSG0000 | 0,006244 | 0,266018 | 2,67141 | 0,007951 | 0,805608 |
| ENSG0000 | -0,005077 | -0,474423 | -2,669094 | 0,008004 | 0,805608 |
| ENSG0000 | -0,008753 | 0,441698 | -2,661538 | 0,008182 | 0,811467 |
| ENSG0000 | 0,002997 | -0,153592 | 2,658304 | 0,008259 | 0,811467 |
| ENSG0000 | -0,003665 | 0,449266 | -2,641839 | 0,008662 | 0,819394 |
| ENSG0000 | 0,003773 | 0,171687 | 2,637909 | 0,008761 | 0,819394 |
| ENSG0000 | -0,004722 | 0,27723 | -2,636098 | 0,008807 | 0,819394 |
| ENSG0000 | 0,00377 | 0,259442 | 2,635807 | 0,008814 | 0,819394 |
| ENSG0000 | -0,002907 | -0,014924 | -2,62964 | 0,008972 | 0,819394 |
| ENSG0000 | -0,003695 | -0,153976 | -2,627256 | 0,009034 | 0,819394 |
| ENSG0000 | -0,004653 | 0,459077 | -2,623736 | 0,009126 | 0,819394 |
| ENSG0000 | -0,004904 | 0,213135 | -2,61791 | 0,009279 | 0,819394 |
| ENSG0000 | -0,003632 | 0,077923 | -2,61391 | 0,009386 | 0,819394 |
| ENSG0000 | -0,006058 | -0,089501 | -2,613511 | 0,009397 | 0,819394 |
| ENSG0000 | -0,005008 | -0,405824 | -2,61081 | 0,00947 | 0,819394 |
| ENSG0000 | 0,003193 | -0,157202 | 2,607008 | 0,009573 | 0,819394 |

| | | | | | |
|---|---|---|---|---|---|
| ENSG0000 | 0,006884 | 0,278231 | 2,602431 | 0,009699 | 0,819394 |
| ENSG0000 | 0,001587 | 0,186529 | 2,599197 | 0,009789 | 0,819394 |
| ENSG0000 | -0,008728 | -0,771923 | -2,597766 | 0,009829 | 0,819394 |
| ENSG0000 | -0,003274 | -0,016486 | -2,592227 | 0,009985 | 0,820478 |
| ENSG0000 | 0,001549 | -0,301508 | 2,590273 | 0,010041 | 0,820478 |
| ENSG0000 | 0,002322 | 0,14007 | 2,583818 | 0,010227 | 0,827464 |
| ENSG0000 | -0,002225 | 0,097574 | -2,554875 | 0,011098 | 0,868739 |
| ENSG0000 | -0,003762 | 0,182459 | -2,551602 | 0,011201 | 0,868739 |
| ENSG0000 | -0,003857 | 0,400204 | -2,548888 | 0,011286 | 0,868739 |
| ENSG0000 | 0,006431 | 0,095824 | 2,547671 | 0,011325 | 0,868739 |
| ENSG0000 | 0,010164 | 0,079391 | 2,544306 | 0,011432 | 0,868739 |
| ENSG0000 | 0,007068 | 0,488733 | 2,537185 | 0,011663 | 0,868739 |
| ENSG0000 | 0,003784 | 0,050971 | 2,535271 | 0,011725 | 0,868739 |
| ENSG0000 | -0,009202 | -0,172473 | -2,533661 | 0,011778 | 0,868739 |
| ENSG0000 | -0,00116 | 0,072754 | -2,531792 | 0,01184 | 0,868739 |
| ENSG0000 | 0,00326 | 0,008236 | 2,528701 | 0,011942 | 0,868739 |
| ENSG0000 | 0,003349 | 0,147597 | 2,525367 | 0,012054 | 0,868739 |
| ENSG0000 | 0,004086 | -0,027646 | 2,513331 | 0,012464 | 0,868739 |
| ENSG0000 | -0,002588 | 0,004904 | -2,510959 | 0,012547 | 0,868739 |
| ENSG0000 | -0,003132 | 0,042674 | -2,507532 | 0,012667 | 0,868739 |
| ENSG0000 | 0,003482 | 0,021628 | 2,507313 | 0,012674 | 0,868739 |
| ENSG0000 | -0,010656 | -1,062928 | -2,505649 | 0,012733 | 0,868739 |
| ENSG0000 | 0,003253 | 0,130219 | 2,50514 | 0,012751 | 0,868739 |
| ENSG0000 | 0,006563 | 0,159909 | 2,500094 | 0,01293 | 0,868739 |
| ENSG0000 | 0,003424 | -0,131423 | 2,498565 | 0,012985 | 0,868739 |
| ENSG0000 | 0,002644 | 0,003204 | 2,495193 | 0,013106 | 0,868739 |
| ENSG0000 | 0,001547 | 0,031743 | 2,491349 | 0,013246 | 0,868739 |
| ENSG0000 | 0,002071 | -0,093228 | 2,490667 | 0,013271 | 0,868739 |
| ENSG0000 | -0,004322 | -0,121975 | -2,484682 | 0,013492 | 0,868739 |
| ENSG0000 | 0,001356 | 0,068023 | 2,48331 | 0,013543 | 0,868739 |
| ENSG0000 | -0,002501 | 0,369429 | -2,483026 | 0,013554 | 0,868739 |
| ENSG0000 | 0,004325 | -0,150401 | 2,479049 | 0,013703 | 0,868739 |
| ENSG0000 | 0,003731 | 0,27308 | 2,478588 | 0,01372 | 0,868739 |
| ENSG0000 | 0,005695 | -0,327512 | 2,477714 | 0,013753 | 0,868739 |
| ENSG0000 | 0,001003 | -0,029142 | 2,476746 | 0,01379 | 0,868739 |
| ENSG0000 | 0,008998 | -0,644659 | 2,467589 | 0,01414 | 0,872666 |
| ENSG0000 | -0,002453 | 0,155728 | -2,463515 | 0,014299 | 0,872666 |
| ENSG0000 | -0,001323 | -0,118387 | -2,462348 | 0,014344 | 0,872666 |
| ENSG0000 | 0,00265 | 0,186198 | 2,461509 | 0,014377 | 0,872666 |
| ENSG0000 | 0,004455 | -0,144732 | 2,457324 | 0,014543 | 0,872666 |
| ENSG0000 | 0,002462 | -0,032185 | 2,456718 | 0,014567 | 0,872666 |
| ENSG0000 | 0,003341 | -0,024952 | 2,455569 | 0,014612 | 0,872666 |
| ENSG0000 | 0,006771 | 0,297828 | 2,452591 | 0,014732 | 0,872666 |
| ENSG0000 | 0,005239 | 0,052925 | 2,449859 | 0,014842 | 0,872666 |
| ENSG0000 | 0,003285 | -0,135043 | 2,44819 | 0,014909 | 0,872666 |
| ENSG0000 | 0,004439 | 0,144865 | 2,444761 | 0,015049 | 0,873662 |
| ENSG0000 | 0,001822 | 0,062006 | 2,438057 | 0,015325 | 0,873662 |
| ENSG0000 | -0,005713 | -0,483379 | -2,436032 | 0,01541 | 0,873662 |

| | | | | | |
|---|---|---|---|---|---|
| ENSG0000 | 0,01051 | 0,767973 | 2,434518 | 0,015473 | 0,873662 |
| ENSG0000 | -0,002138 | -0,092865 | -2,432245 | 0,015569 | 0,873662 |
| ENSG0000 | 0,00305 | 0,196675 | 2,428051 | 0,015747 | 0,873662 |
| ENSG0000 | -0,002436 | -0,154 | -2,426306 | 0,015821 | 0,873662 |
| ENSG0000 | -0,004584 | 0,388053 | -2,420046 | 0,016091 | 0,873662 |
| ENSG0000 | 0,004266 | 0,077784 | 2,415181 | 0,016303 | 0,873662 |
| ENSG0000 | 0,003259 | 0,01548 | 2,410262 | 0,01652 | 0,873662 |
| ENSG0000 | 0,003964 | 0,113769 | 2,408885 | 0,016582 | 0,873662 |
| ENSG0000 | 0,00271 | 0,081165 | 2,405434 | 0,016736 | 0,873662 |
| ENSG0000 | 0,005878 | -0,688659 | 2,405249 | 0,016745 | 0,873662 |
| ENSG0000 | 0,003401 | -0,189729 | 2,403656 | 0,016816 | 0,873662 |
| ENSG0000 | 0,003246 | 0,379051 | 2,401946 | 0,016894 | 0,873662 |
| ENSG0000 | -0,004588 | -0,101362 | -2,400604 | 0,016955 | 0,873662 |
| ENSG0000 | 0,00283 | -0,053604 | 2,399474 | 0,017006 | 0,873662 |
| ENSG0000 | 0,003502 | -0,001892 | 2,396407 | 0,017146 | 0,873662 |
| ENSG0000 | 0,006812 | 0,173571 | 2,396 | 0,017165 | 0,873662 |
| ENSG0000 | 0,001557 | -0,096967 | 2,391165 | 0,017389 | 0,873662 |
| ENSG0000 | -0,002917 | 0,113824 | -2,389869 | 0,017449 | 0,873662 |
| ENSG0000 | -0,002409 | 0,012416 | -2,388427 | 0,017516 | 0,873662 |
| ENSG0000 | -0,007997 | 0,697695 | -2,386602 | 0,017602 | 0,873662 |
| ENSG0000 | -0,003468 | -0,004716 | -2,375025 | 0,018153 | 0,873662 |
| ENSG0000 | -0,010032 | -0,577094 | -2,37493 | 0,018158 | 0,873662 |
| ENSG0000 | 0,003555 | 0,353151 | 2,372102 | 0,018295 | 0,873662 |
| ENSG0000 | 0,008099 | 0,22745 | 2,369647 | 0,018415 | 0,873662 |
| ENSG0000 | -0,001786 | -0,063568 | -2,368696 | 0,018461 | 0,873662 |
| ENSG0000 | 0,003105 | 0,04401 | 2,368503 | 0,018471 | 0,873662 |
| ENSG0000 | -0,005174 | -0,326249 | -2,366625 | 0,018563 | 0,873662 |
| ENSG0000 | 0,00349 | -0,25479 | 2,365735 | 0,018607 | 0,873662 |
| ENSG0000 | -0,004483 | -0,156707 | -2,362995 | 0,018742 | 0,873662 |
| ENSG0000 | 0,001703 | -0,337202 | 2,362147 | 0,018784 | 0,873662 |
| ENSG0000 | -0,001417 | -0,089557 | -2,361503 | 0,018817 | 0,873662 |
| ENSG0000 | 0,002684 | -0,037801 | 2,358652 | 0,018959 | 0,873662 |
| ENSG0000 | -0,003799 | 0,521157 | -2,356684 | 0,019058 | 0,873662 |
| ENSG0000 | 0,005023 | 0,02764 | 2,355474 | 0,019119 | 0,873662 |
| ENSG0000 | -0,002825 | 0,167587 | -2,354358 | 0,019176 | 0,873662 |
| ENSG0000 | 0,008523 | -0,008109 | 2,35412 | 0,019188 | 0,873662 |
| ENSG0000 | 0,007075 | -0,168759 | 2,352563 | 0,019267 | 0,873662 |
| ENSG0000 | -0,006762 | 0,482177 | -2,347685 | 0,019516 | 0,873662 |
| ENSG0000 | -0,004737 | 0,502107 | -2,347431 | 0,019529 | 0,873662 |
| ENSG0000 | -0,002459 | 0,09972 | -2,345461 | 0,019631 | 0,873662 |
| ENSG0000 | 0,005802 | 0,061347 | 2,342656 | 0,019777 | 0,873662 |
| ENSG0000 | 0,003441 | -0,146301 | 2,340983 | 0,019864 | 0,873662 |
| ENSG0000 | 0,006562 | 0,758324 | 2,339284 | 0,019953 | 0,873662 |
| ENSG0000 | -0,002417 | -0,062753 | -2,338736 | 0,019982 | 0,873662 |
| ENSG0000 | 0,002023 | 0,131197 | 2,338241 | 0,020008 | 0,873662 |
| ENSG0000 | -0,001796 | 0,029265 | -2,334465 | 0,020207 | 0,877726 |
| ENSG0000 | 0,002224 | 0,203998 | 2,326514 | 0,020633 | 0,884378 |
| ENSG0000 | 0,006808 | 0,128194 | 2,326301 | 0,020644 | 0,884378 |

| | | | | | |
|---|---|---|---|---|---|
| ENSG0000 | -0,003696 | -0,252034 | -2,325609 | 0,020682 | 0,884378 |
| ENSG0000 | 0,006824 | 0,304554 | 2,322779 | 0,020835 | 0,886358 |
| ENSG0000 | -0,005758 | -0,351994 | -2,318326 | 0,021079 | 0,88946 |
| ENSG0000 | -0,006476 | 0,360641 | -2,317011 | 0,021152 | 0,88946 |
| ENSG0000 | -0,00243 | 0,049703 | -2,315567 | 0,021232 | 0,88946 |
| ENSG0000 | 0,00161 | -0,031076 | 2,313224 | 0,021362 | 0,890392 |
| ENSG0000 | -0,00285 | 0,031839 | -2,305853 | 0,021776 | 0,897706 |
| ENSG0000 | -0,003393 | 0,216564 | -2,303042 | 0,021935 | 0,897706 |
| ENSG0000 | -0,002192 | 0,056065 | -2,299408 | 0,022143 | 0,897706 |
| ENSG0000 | -0,00114 | -0,066633 | -2,29922 | 0,022154 | 0,897706 |
| ENSG0000 | -0,001348 | -0,013107 | -2,2988 | 0,022178 | 0,897706 |
| ENSG0000 | -0,002063 | 0,118974 | -2,298602 | 0,02219 | 0,897706 |
| ENSG0000 | -0,003137 | -0,142795 | -2,27846 | 0,023376 | 0,90628 |
| ENSG0000 | -0,003346 | 0,006284 | -2,27667 | 0,023484 | 0,90628 |
| ENSG0000 | 0,00296 | -0,174939 | 2,27381 | 0,023658 | 0,90628 |
| ENSG0000 | -0,006258 | 0,330071 | -2,269807 | 0,023903 | 0,90628 |
| ENSG0000 | 0,006404 | 0,089659 | 2,265281 | 0,024182 | 0,90628 |
| ENSG0000 | 0,003083 | 0,369442 | 2,264979 | 0,024201 | 0,90628 |
| ENSG0000 | -0,002846 | -0,215367 | -2,264528 | 0,024229 | 0,90628 |
| ENSG0000 | 0,001891 | -0,077643 | 2,261491 | 0,024418 | 0,90628 |
| ENSG0000 | 0,005115 | 0,23689 | 2,259397 | 0,02455 | 0,90628 |
| ENSG0000 | 0,006527 | -0,344715 | 2,258416 | 0,024612 | 0,90628 |
| ENSG0000 | -0,008146 | -0,276192 | -2,258371 | 0,024614 | 0,90628 |
| ENSG0000 | -0,004682 | 0,303363 | -2,254244 | 0,024876 | 0,90628 |
| ENSG0000 | -0,006003 | 0,163066 | -2,25216 | 0,025009 | 0,90628 |
| ENSG0000 | -0,004564 | 0,042713 | -2,250102 | 0,02514 | 0,90628 |
| ENSG0000 | -0,002575 | 0,031356 | -2,249862 | 0,025156 | 0,90628 |
| ENSG0000 | -0,001765 | 0,093213 | -2,249472 | 0,025181 | 0,90628 |
| ENSG0000 | 0,002276 | 0,004889 | 2,24938 | 0,025187 | 0,90628 |
| ENSG0000 | -0,008015 | 0,333487 | -2,247377 | 0,025316 | 0,90628 |
| ENSG0000 | 0,005662 | 0,50117 | 2,244698 | 0,025489 | 0,90628 |
| ENSG0000 | -0,005785 | -0,331166 | -2,242305 | 0,025645 | 0,90628 |
| ENSG0000 | -0,001801 | -0,063434 | -2,24137 | 0,025706 | 0,90628 |
| ENSG0000 | 0,002351 | 0,069801 | 2,240016 | 0,025795 | 0,90628 |
| ENSG0000 | 0,002009 | 0,024972 | 2,239227 | 0,025847 | 0,90628 |
| ENSG0000 | -0,001958 | 0,166 | -2,237405 | 0,025967 | 0,90628 |
| ENSG0000 | 0,006674 | -0,039926 | 2,236557 | 0,026023 | 0,90628 |
| ENSG0000 | 0,004635 | -0,619632 | 2,236092 | 0,026054 | 0,90628 |
| ENSG0000 | 0,001157 | -0,003023 | 2,232398 | 0,0263 | 0,90628 |
| ENSG0000 | 0,001582 | -0,148059 | 2,231038 | 0,026391 | 0,90628 |
| ENSG0000 | 0,004005 | 0,306278 | 2,228418 | 0,026566 | 0,90628 |
| ENSG0000 | 0,002857 | 0,105011 | 2,227903 | 0,026601 | 0,90628 |
| ENSG0000 | -0,00628 | -0,033276 | -2,227645 | 0,026619 | 0,90628 |
| ENSG0000 | -0,006341 | -0,090012 | -2,227332 | 0,02664 | 0,90628 |
| ENSG0000 | 0,004738 | 0,025548 | 2,225027 | 0,026796 | 0,90628 |
| ENSG0000 | -0,006282 | 0,061171 | -2,22358 | 0,026894 | 0,90628 |
| ENSG0000 | 0,003455 | 0,164266 | 2,222579 | 0,026962 | 0,90628 |
| ENSG0000 | -0,001332 | 0,012201 | -2,221476 | 0,027038 | 0,90628 |

| | | | | | |
|---|---|---|---|---|---|
| ENSG0000 | -0,002622 | 0,015784 | -2,220848 | 0,027081 | 0,90628 |
| ENSG0000 | 0,005342 | -0,143396 | 2,21415 | 0,027543 | 0,90628 |
| ENSG0000 | 0,002153 | 0,05274 | 2,212496 | 0,027658 | 0,90628 |
| ENSG0000 | -0,003235 | 0,355349 | -2,208943 | 0,027907 | 0,90628 |
| ENSG0000 | -0,005547 | -0,608859 | -2,208231 | 0,027957 | 0,90628 |
| ENSG0000 | -0,002497 | -0,071503 | -2,207549 | 0,028005 | 0,90628 |
| ENSG0000 | 0,001522 | 0,173806 | 2,206984 | 0,028045 | 0,90628 |
| ENSG0000 | -0,002947 | 0,257939 | -2,206667 | 0,028067 | 0,90628 |
| ENSG0000 | 0,00518 | 1,020508 | 2,206006 | 0,028114 | 0,90628 |
| ENSG0000 | -0,004156 | 0,273785 | -2,203923 | 0,028261 | 0,90628 |
| ENSG0000 | 0,009899 | -1,117361 | 2,202954 | 0,02833 | 0,90628 |
| ENSG0000 | -0,006776 | -0,499186 | -2,201987 | 0,028399 | 0,90628 |
| ENSG0000 | 0,003217 | 0,24581 | 2,198263 | 0,028666 | 0,90628 |
| ENSG0000 | -0,00306 | -0,091307 | -2,197064 | 0,028752 | 0,90628 |
| ENSG0000 | -0,007269 | -0,176973 | -2,194235 | 0,028957 | 0,90628 |
| ENSG0000 | -0,00263 | 0,005328 | -2,19415 | 0,028963 | 0,90628 |
| ENSG0000 | 0,002414 | -0,041009 | 2,191708 | 0,029141 | 0,90628 |
| ENSG0000 | 0,001733 | -0,001486 | 2,190315 | 0,029243 | 0,90628 |
| ENSG0000 | -0,003864 | -0,473485 | -2,189624 | 0,029293 | 0,90628 |
| ENSG0000 | -0,001643 | 0,127311 | -2,189601 | 0,029295 | 0,90628 |
| ENSG0000 | 0,001123 | 0,08248 | 2,189548 | 0,029299 | 0,90628 |
| ENSG0000 | 0,001509 | -0,103549 | 2,188401 | 0,029383 | 0,90628 |
| ENSG0000 | -0,003018 | 0,099382 | -2,187858 | 0,029423 | 0,90628 |
| ENSG0000 | -0,007293 | 0,140616 | -2,185798 | 0,029575 | 0,90628 |
| ENSG0000 | -0,004001 | 0,003017 | -2,185319 | 0,02961 | 0,90628 |
| ENSG0000 | -0,005063 | -0,074534 | -2,184641 | 0,029661 | 0,90628 |
| ENSG0000 | -0,003365 | -0,040192 | -2,184168 | 0,029696 | 0,90628 |
| ENSG0000 | -0,006811 | 0,375285 | -2,183964 | 0,029711 | 0,90628 |
| ENSG0000 | 0,003499 | -1,30753 | 2,183903 | 0,029715 | 0,90628 |
| ENSG0000 | -0,005538 | 0,094821 | -2,182033 | 0,029854 | 0,90628 |
| ENSG0000 | 0,003407 | -0,423762 | 2,179837 | 0,030018 | 0,90628 |
| ENSG0000 | -0,002961 | 0,1409 | -2,179701 | 0,030029 | 0,90628 |
| ENSG0000 | -0,00692 | 0,167362 | -2,179548 | 0,03004 | 0,90628 |
| ENSG0000 | 0,000964 | -0,163024 | 2,178901 | 0,030089 | 0,90628 |
| ENSG0000 | 0,003776 | -0,118527 | 2,176655 | 0,030257 | 0,908052 |
| ENSG0000 | -0,002452 | 0,100379 | -2,174675 | 0,030407 | 0,909236 |
| ENSG0000 | -0,003934 | -0,163356 | -2,171726 | 0,030631 | 0,912625 |
| ENSG0000 | 0,00318 | 0,023467 | 2,161874 | 0,03139 | 0,918442 |
| ENSG0000 | 0,002854 | -0,256924 | 2,161193 | 0,031443 | 0,918442 |
| ENSG0000 | -0,004116 | 0,136119 | -2,159924 | 0,031542 | 0,918442 |
| ENSG0000 | -0,005564 | -0,764898 | -2,157211 | 0,031754 | 0,918442 |
| ENSG0000 | -0,009673 | -0,690401 | -2,1561 | 0,031842 | 0,918442 |
| ENSG0000 | -0,007586 | 0,519187 | -2,155916 | 0,031856 | 0,918442 |
| ENSG0000 | -0,005318 | -0,478779 | -2,154672 | 0,031954 | 0,918442 |
| ENSG0000 | 0,004543 | -0,117771 | 2,153247 | 0,032067 | 0,918442 |
| ENSG0000 | 0,003119 | 0,50561 | 2,150203 | 0,032309 | 0,918442 |
| ENSG0000 | -0,009326 | 0,459993 | -2,149862 | 0,032336 | 0,918442 |
| ENSG0000 | 0,001656 | 0,068816 | 2,149061 | 0,0324 | 0,918442 |

| | | | | | |
|---|---|---|---|---|---|
| ENSG0000 | -0,002361 | -0,020437 | -2,14805 | 0,032481 | 0,918442 |
| ENSG0000 | -0,002429 | 0,057648 | -2,14787 | 0,032496 | 0,918442 |
| ENSG0000 | 0,002077 | 0,106763 | 2,146931 | 0,032571 | 0,918442 |
| ENSG0000 | -0,006287 | -0,222597 | -2,145719 | 0,032668 | 0,918442 |
| ENSG0000 | 0,002755 | -0,217436 | 2,144058 | 0,032802 | 0,918442 |
| ENSG0000 | 0,001982 | -0,356973 | 2,143218 | 0,03287 | 0,918442 |
| ENSG0000 | -0,004387 | -0,348014 | -2,142031 | 0,032967 | 0,918442 |
| ENSG0000 | -0,003196 | 0,413662 | -2,141005 | 0,03305 | 0,918442 |
| ENSG0000 | -0,003907 | 0,54577 | -2,13599 | 0,03346 | 0,918442 |
| ENSG0000 | -0,002473 | -0,041016 | -2,135497 | 0,033501 | 0,918442 |
| ENSG0000 | -0,00089 | 0,068027 | -2,13502 | 0,03354 | 0,918442 |
| ENSG0000 | -0,004856 | -0,108927 | -2,133621 | 0,033655 | 0,918442 |
| ENSG0000 | 0,003896 | 0,549459 | 2,132018 | 0,033788 | 0,918442 |
| ENSG0000 | -0,003332 | -0,353363 | -2,131478 | 0,033833 | 0,918442 |
| ENSG0000 | 0,004363 | 0,306014 | 2,128248 | 0,034102 | 0,918442 |
| ENSG0000 | 0,001251 | -0,09527 | 2,127994 | 0,034123 | 0,918442 |
| ENSG0000 | -0,003709 | 0,255773 | -2,125804 | 0,034306 | 0,918442 |
| ENSG0000 | -0,003661 | -0,023305 | -2,125732 | 0,034312 | 0,918442 |
| ENSG0000 | 0,001259 | 0,041247 | 2,125159 | 0,034361 | 0,918442 |
| ENSG0000 | -0,002148 | -0,11833 | -2,123498 | 0,0345 | 0,918442 |
| ENSG0000 | 0,002841 | 0,532607 | 2,121783 | 0,034645 | 0,918442 |
| ENSG0000 | 0,002811 | -0,067481 | 2,121084 | 0,034705 | 0,918442 |
| ENSG0000 | -0,006989 | -0,949884 | -2,12095 | 0,034716 | 0,918442 |
| ENSG0000 | 0,000664 | 0,07911 | 2,12089 | 0,034721 | 0,918442 |
| ENSG0000 | 0,002968 | -0,083702 | 2,117237 | 0,035032 | 0,921017 |
| ENSG0000 | 0,003513 | 0,25434 | 2,11589 | 0,035148 | 0,921017 |
| ENSG0000 | -0,004652 | 0,144957 | -2,114969 | 0,035227 | 0,921017 |
| ENSG0000 | -0,003997 | 0,024002 | -2,114281 | 0,035286 | 0,921017 |
| ENSG0000 | 0,004565 | -0,618279 | 2,112007 | 0,035482 | 0,921017 |
| ENSG0000 | 0,008344 | -0,653022 | 2,109578 | 0,035693 | 0,921017 |
| ENSG0000 | 0,002008 | -0,000928 | 2,109478 | 0,035701 | 0,921017 |
| ENSG0000 | -0,003282 | 0,122557 | -2,108791 | 0,035761 | 0,921017 |
| ENSG0000 | 0,005669 | 0,148026 | 2,104556 | 0,036131 | 0,921017 |
| ENSG0000 | 0,004797 | -0,223278 | 2,100597 | 0,03648 | 0,921017 |
| ENSG0000 | 0,005527 | -0,256442 | 2,100586 | 0,036481 | 0,921017 |
| ENSG0000 | -0,001877 | -0,100739 | -2,095894 | 0,036899 | 0,921017 |
| ENSG0000 | 0,005105 | -0,190005 | 2,094907 | 0,036987 | 0,921017 |
| ENSG0000 | 0,003394 | -0,069966 | 2,094564 | 0,037018 | 0,921017 |
| ENSG0000 | -0,000423 | 0,001971 | -2,093504 | 0,037113 | 0,921017 |
| ENSG0000 | 0,003981 | 0,003233 | 2,09145 | 0,037298 | 0,921017 |
| ENSG0000 | 0,002948 | 0,182558 | 2,088419 | 0,037572 | 0,921017 |
| ENSG0000 | 0,004815 | 0,320105 | 2,087949 | 0,037615 | 0,921017 |
| ENSG0000 | -0,004119 | 0,3852 | -2,085909 | 0,037801 | 0,921017 |
| ENSG0000 | -0,001545 | 0,046468 | -2,081793 | 0,038178 | 0,921017 |
| ENSG0000 | -0,006857 | 0,164201 | -2,081074 | 0,038244 | 0,921017 |
| ENSG0000 | 0,002636 | -0,201181 | 2,078225 | 0,038507 | 0,921017 |
| ENSG0000 | -0,001284 | 0,008356 | -2,077046 | 0,038617 | 0,921017 |
| ENSG0000 | -0,002044 | 0,177009 | -2,074763 | 0,03883 | 0,921017 |

| | | | | | |
|---|---|---|---|---|---|
| ENSG0000 | 0,004543 | 0,125532 | 2,073299 | 0,038967 | 0,921017 |
| ENSG0000 | 0,000434 | -0,030691 | 2,07238 | 0,039053 | 0,921017 |
| ENSG0000 | -0,002031 | -0,104608 | -2,072054 | 0,039083 | 0,921017 |
| ENSG0000 | -0,004545 | 0,307851 | -2,071955 | 0,039093 | 0,921017 |
| ENSG0000 | -0,001618 | -0,091584 | -2,071952 | 0,039093 | 0,921017 |
| ENSG0000 | -0,005602 | 0,184083 | -2,071424 | 0,039143 | 0,921017 |
| ENSG0000 | 0,002257 | -0,030417 | 2,070046 | 0,039272 | 0,921017 |
| ENSG0000 | 0,002739 | -0,330555 | 2,069007 | 0,03937 | 0,921017 |
| ENSG0000 | 0,004725 | 0,317435 | 2,068639 | 0,039405 | 0,921017 |
| ENSG0000 | 0,00148 | -0,102411 | 2,067411 | 0,039521 | 0,921017 |
| ENSG0000 | 0,00237 | -0,01465 | 2,064471 | 0,039801 | 0,921017 |
| ENSG0000 | 0,004443 | 0,003401 | 2,064066 | 0,03984 | 0,921017 |
| ENSG0000 | 0,002803 | 0,018556 | 2,063835 | 0,039862 | 0,921017 |
| ENSG0000 | 0,002286 | 0,149844 | 2,063024 | 0,039939 | 0,921017 |
| ENSG0000 | -0,001489 | -0,02929 | -2,062123 | 0,040025 | 0,921017 |
| ENSG0000 | -0,003026 | -0,192781 | -2,060454 | 0,040186 | 0,921017 |
| ENSG0000 | -0,003391 | -0,165561 | -2,060045 | 0,040225 | 0,921017 |
| ENSG0000 | -0,002323 | 0,009615 | -2,05967 | 0,040261 | 0,921017 |
| ENSG0000 | 0,004851 | -0,044794 | 2,057806 | 0,040441 | 0,921017 |
| ENSG0000 | 0,001364 | -0,218882 | 2,057206 | 0,040499 | 0,921017 |
| ENSG0000 | -0,005319 | 0,298944 | -2,057024 | 0,040517 | 0,921017 |
| ENSG0000 | 0,001858 | -0,106187 | 2,055672 | 0,040648 | 0,921017 |
| ENSG0000 | -0,001392 | 0,107914 | -2,05136 | 0,041068 | 0,921017 |
| ENSG0000 | 0,002021 | 0,017034 | 2,048971 | 0,041303 | 0,921017 |
| ENSG0000 | 0,001293 | 0,074568 | 2,048156 | 0,041383 | 0,921017 |
| ENSG0000 | -0,004694 | -0,157808 | -2,047882 | 0,04141 | 0,921017 |
| ENSG0000 | -0,006139 | 0,450251 | -2,047473 | 0,041451 | 0,921017 |
| ENSG0000 | 0,003601 | 0,157233 | 2,045569 | 0,041639 | 0,921017 |
| ENSG0000 | 0,004166 | -0,028966 | 2,045435 | 0,041652 | 0,921017 |
| ENSG0000 | 0,00161 | -0,157898 | 2,044208 | 0,041774 | 0,921017 |
| ENSG0000 | -0,001737 | 0,01295 | -2,03775 | 0,04242 | 0,921017 |
| ENSG0000 | -0,00403 | 0,160417 | -2,037431 | 0,042452 | 0,921017 |
| ENSG0000 | -0,003779 | -0,300544 | -2,037259 | 0,042469 | 0,921017 |
| ENSG0000 | 0,002571 | 0,151308 | 2,036425 | 0,042554 | 0,921017 |
| ENSG0000 | -0,009151 | -0,415111 | -2,035418 | 0,042655 | 0,921017 |
| ENSG0000 | 0,002238 | -0,13217 | 2,034555 | 0,042743 | 0,921017 |
| ENSG0000 | 0,002341 | -0,455621 | 2,033813 | 0,042818 | 0,921017 |
| ENSG0000 | -0,003206 | -0,314314 | -2,032679 | 0,042933 | 0,921017 |
| ENSG0000 | 0,004533 | 0,203289 | 2,030691 | 0,043136 | 0,921017 |
| ENSG0000 | 0,001987 | 0,079196 | 2,030042 | 0,043202 | 0,921017 |
| ENSG0000 | -0,005363 | 0,387795 | -2,029831 | 0,043224 | 0,921017 |
| ENSG0000 | 0,003689 | -0,414593 | 2,025209 | 0,043699 | 0,921017 |
| ENSG0000 | 0,004722 | 0,382045 | 2,020781 | 0,044158 | 0,921017 |
| ENSG0000 | -0,001681 | 0,044217 | -2,019923 | 0,044248 | 0,921017 |
| ENSG0000 | -0,002709 | 0,09019 | -2,019286 | 0,044314 | 0,921017 |
| ENSG0000 | -0,004218 | -0,367886 | -2,018384 | 0,044408 | 0,921017 |
| ENSG0000 | -0,002341 | 0,328364 | -2,017594 | 0,044491 | 0,921017 |
| ENSG0000 | 0,000871 | 0,047648 | 2,01616 | 0,044642 | 0,921017 |

| | | | | | |
|---|---|---|---|---|---|
| ENSG0000 | -0,004724 | -0,413183 | -2,013197 | 0,044954 | 0,921017 |
| ENSG0000 | -0,003411 | 0,346968 | -2,012839 | 0,044992 | 0,921017 |
| ENSG0000 | 0,001524 | 0,043987 | 2,012442 | 0,045034 | 0,921017 |
| ENSG0000 | 0,003031 | 0,250747 | 2,008211 | 0,045484 | 0,921017 |
| ENSG0000 | 0,005399 | -0,339773 | 2,007277 | 0,045584 | 0,921017 |
| ENSG0000 | 0,002327 | 0,03812 | 2,006816 | 0,045633 | 0,921017 |
| ENSG0000 | -0,002921 | -0,092783 | -2,00656 | 0,045661 | 0,921017 |
| ENSG0000 | 0,003572 | 0,049615 | 2,005121 | 0,045815 | 0,921017 |
| ENSG0000 | -0,001622 | 0,416322 | -2,004803 | 0,04585 | 0,921017 |
| ENSG0000 | 0,003633 | 0,14099 | 2,004531 | 0,045879 | 0,921017 |
| ENSG0000 | 0,002162 | 0,000655 | 2,004106 | 0,045925 | 0,921017 |
| ENSG0000 | -0,001689 | -0,181122 | -2,002562 | 0,046091 | 0,921017 |
| ENSG0000 | -0,001828 | -0,079007 | -2,002302 | 0,046119 | 0,921017 |
| ENSG0000 | -0,001624 | -0,119361 | -2,001495 | 0,046206 | 0,921017 |
| ENSG0000 | 0,003208 | -0,389772 | 2,001481 | 0,046208 | 0,921017 |
| ENSG0000 | -0,001954 | -0,178315 | -2,001049 | 0,046255 | 0,921017 |
| ENSG0000 | -0,004983 | 0,08739 | -2,000841 | 0,046277 | 0,921017 |
| ENSG0000 | 0,007924 | -0,522468 | 2,00076 | 0,046286 | 0,921017 |
| ENSG0000 | 0,00144 | 0,024556 | 2,000501 | 0,046314 | 0,921017 |
| ENSG0000 | -0,00203 | -0,113909 | -2,000077 | 0,04636 | 0,921017 |
| ENSG0000 | 0,004778 | -0,201614 | 1,999539 | 0,046419 | 0,921017 |
| ENSG0000 | 0,002486 | -0,263498 | 1,999291 | 0,046446 | 0,921017 |
| ENSG0000 | 0,006821 | -0,388084 | 1,996758 | 0,046722 | 0,921017 |
| ENSG0000 | -0,001615 | -0,13585 | -1,996273 | 0,046775 | 0,921017 |
| ENSG0000 | -0,006413 | -0,162791 | -1,995918 | 0,046814 | 0,921017 |
| ENSG0000 | -0,004202 | 0,092174 | -1,995398 | 0,046871 | 0,921017 |
| ENSG0000 | 0,005618 | -0,774593 | 1,995171 | 0,046895 | 0,921017 |
| ENSG0000 | 0,002494 | 0,25724 | 1,994769 | 0,046939 | 0,921017 |
| ENSG0000 | -0,001007 | 0,029645 | -1,994489 | 0,04697 | 0,921017 |
| ENSG0000 | 0,002837 | -0,010515 | 1,993803 | 0,047046 | 0,921017 |
| ENSG0000 | 0,003663 | 0,2801 | 1,991985 | 0,047246 | 0,921017 |
| ENSG0000 | 0,001142 | -0,117718 | 1,991828 | 0,047263 | 0,921017 |
| ENSG0000 | -0,003042 | -0,059166 | -1,991778 | 0,047269 | 0,921017 |
| ENSG0000 | -0,002347 | -0,162389 | -1,990158 | 0,047448 | 0,921017 |
| ENSG0000 | -0,003525 | 0,582962 | -1,989827 | 0,047484 | 0,921017 |
| ENSG0000 | 0,001142 | -0,067216 | 1,989801 | 0,047487 | 0,921017 |
| ENSG0000 | -0,001462 | 0,045836 | -1,98975 | 0,047493 | 0,921017 |
| ENSG0000 | 0,000888 | -0,007091 | 1,986817 | 0,047819 | 0,921017 |
| ENSG0000 | 0,002528 | 0,160847 | 1,986453 | 0,047859 | 0,921017 |
| ENSG0000 | 0,007064 | 0,301081 | 1,985226 | 0,047996 | 0,921017 |
| ENSG0000 | -0,003543 | -0,028612 | -1,98489 | 0,048034 | 0,921017 |
| ENSG0000 | 0,005548 | -0,834788 | 1,98454 | 0,048073 | 0,921017 |
| ENSG0000 | -0,006295 | 0,542846 | -1,982297 | 0,048325 | 0,921017 |
| ENSG0000 | 0,007079 | -0,094301 | 1,980705 | 0,048504 | 0,921017 |
| ENSG0000 | 0,002685 | 0,105125 | 1,979965 | 0,048588 | 0,921017 |
| ENSG0000 | -0,004435 | -0,695327 | -1,978899 | 0,048708 | 0,921017 |
| ENSG0000 | -0,003886 | -0,231564 | -1,977006 | 0,048923 | 0,921017 |
| ENSG0000 | -0,002775 | -0,382817 | -1,974578 | 0,049199 | 0,921017 |

| | | | | | |
|---|---|---|---|---|---|
| ENSG0000 | 0,001983 | -0,053619 | 1,973287 | 0,049347 | 0,921017 |
| ENSG0000 | 0,005759 | 0,950136 | 1,972551 | 0,049431 | 0,921017 |
| ENSG0000 | 0,003705 | -0,555006 | 1,972114 | 0,049481 | 0,921017 |
| ENSG0000 | -0,00073 | -0,031037 | -1,970827 | 0,049629 | 0,921017 |
| ENSG0000 | -0,003478 | -0,017224 | -1,970788 | 0,049634 | 0,921017 |
| ENSG0000 | -0,003347 | -0,14206 | -1,969255 | 0,04981 | 0,921017 |
| ENSG0000 | 0,002614 | 0,334141 | 1,968749 | 0,049869 | 0,921017 |
| ENSG0000 | -0,005595 | 0,271549 | -1,968507 | 0,049897 | 0,921017 |
| ENSG0000 | 0,004788 | 0,538365 | 1,968039 | 0,049951 | 0,921017 |
| ENSG0000 | -0,001077 | -0,105939 | -1,967959 | 0,04996 | 0,921017 |

| geneID | logFC | AveExpr | t | P.Value | adj.P.Val |
|---|---|---|---|---|---|
| ENSG00000162614.14 | -0,003739 | 0,126372 | -3,756342 | 0,000206 | 0,742593 |
| ENSG00000229212.3 | 0,007267 | -0,33791 | 3,49365 | 0,000545 | 0,742593 |
| ENSG00000162704.11 | -0,004836 | 0,163204 | -3,413534 | 0,000726 | 0,742593 |
| ENSG00000242498.3 | 0,005511 | -0,221296 | 3,380459 | 0,000816 | 0,742593 |
| ENSG00000133101.5 | -0,006801 | 0,325748 | -3,363393 | 0,000866 | 0,742593 |
| ENSG00000131697.13 | -0,004956 | 0,077476 | -3,34969 | 0,000908 | 0,742593 |
| ENSG00000269001.1 | 0,005239 | -0,252369 | 3,340739 | 0,000937 | 0,742593 |
| ENSG00000129467.9 | -0,00471 | -0,150502 | -3,315901 | 0,001021 | 0,742593 |
| ENSG00000197586.8 | -0,005517 | 0,129044 | -3,277661 | 0,001165 | 0,742593 |
| ENSG00000116701.10 | 0,00492 | -0,030445 | 3,272815 | 0,001184 | 0,742593 |
| ENSG00000269051.1 | 0,008215 | -0,246553 | 3,216561 | 0,001434 | 0,742593 |
| ENSG00000272316.1 | 0,003992 | 0,247092 | 3,183271 | 0,001603 | 0,742593 |
| ENSG00000267018.1 | -0,001936 | 0,016639 | -3,135826 | 0,001877 | 0,742593 |
| ENSG00000166359.6 | 0,003073 | 0,095878 | 3,11461 | 0,002013 | 0,742593 |
| ENSG00000241106.2 | -0,012958 | -0,012886 | -3,10368 | 0,002087 | 0,742593 |
| ENSG00000063127.11 | -0,005852 | 0,069438 | -3,100139 | 0,002111 | 0,742593 |
| ENSG00000129455.11 | -0,004923 | -0,17043 | -3,093291 | 0,002159 | 0,742593 |
| ENSG00000241058.1 | -0,010533 | 0,462089 | -3,088037 | 0,002196 | 0,742593 |
| ENSG00000064545.10 | 0,00434 | -0,020975 | 3,082097 | 0,002239 | 0,742593 |
| ENSG00000093167.13 | 0,0038 | -0,101884 | 3,079601 | 0,002257 | 0,742593 |
| ENSG00000138600.5 | 0,006293 | 0,10537 | 3,061095 | 0,002397 | 0,750777 |
| ENSG00000168778.7 | 0,010448 | 0,252687 | 3,036733 | 0,002594 | 0,750777 |
| ENSG00000122873.7 | 0,006434 | -0,401339 | 3,003286 | 0,002887 | 0,750777 |
| ENSG00000204267.9 | 0,006447 | 0,067636 | 3,001903 | 0,0029 | 0,750777 |
| ENSG00000239650.3 | 0,002554 | 0,138721 | 2,941954 | 0,003506 | 0,750777 |
| ENSG00000105982.12 | -0,002988 | 0,445438 | -2,936147 | 0,003571 | 0,750777 |
| ENSG00000272541.1 | -0,006402 | -0,481523 | -2,933574 | 0,0036 | 0,750777 |
| ENSG00000035687.9 | 0,003375 | -0,131546 | 2,933283 | 0,003603 | 0,750777 |
| ENSG00000132823.6 | -0,003048 | -0,107816 | -2,926505 | 0,00368 | 0,750777 |
| ENSG00000109929.5 | -0,001769 | -0,041605 | -2,914635 | 0,003819 | 0,750777 |
| ENSG00000271390.1 | -0,004389 | 0,179575 | -2,911135 | 0,003861 | 0,750777 |
| ENSG00000133103.12 | -0,008352 | -0,282046 | -2,906398 | 0,003918 | 0,750777 |
| ENSG00000226629.1 | -0,001633 | 0,079904 | -2,895577 | 0,004052 | 0,750777 |
| ENSG00000101197.8 | 0,009108 | 0,344209 | 2,886255 | 0,004171 | 0,750777 |
| ENSG00000157150.4 | 0,00852 | 0,202979 | 2,880528 | 0,004245 | 0,750777 |
| ENSG00000157823.12 | -0,008408 | -0,991505 | -2,87943 | 0,00426 | 0,750777 |
| ENSG00000225706.1 | 0,010787 | -0,611903 | 2,878975 | 0,004266 | 0,750777 |
| ENSG00000157017.11 | -0,003784 | 0,113203 | -2,873657 | 0,004336 | 0,750777 |
| ENSG00000124875.5 | -0,001985 | 0,02584 | -2,854749 | 0,004596 | 0,757882 |
| ENSG00000054219.9 | -0,009954 | 0,686122 | -2,847571 | 0,004698 | 0,757882 |
| ENSG00000254333.1 | 0,005569 | 0,086237 | 2,845848 | 0,004723 | 0,757882 |
| ENSG00000171476.17 | -0,004933 | 0,386838 | -2,832023 | 0,004927 | 0,771736 |
| ENSG00000162600.7 | -0,0033 | -0,098185 | -2,814645 | 0,005194 | 0,775686 |
| ENSG00000076650.2 | 0,004758 | 0,10082 | 2,80573 | 0,005336 | 0,775686 |
| ENSG00000065457.6 | -0,011296 | 0,478726 | -2,803253 | 0,005376 | 0,775686 |
| ENSG00000104894.7 | 0,001826 | -0,136141 | 2,800359 | 0,005424 | 0,775686 |
| ENSG00000171700.9 | 0,005175 | 0,324789 | 2,77615 | 0,005834 | 0,785048 |
| ENSG00000272690.1 | 0,003019 | 0,177065 | 2,761321 | 0,006098 | 0,785048 |
| ENSG00000155016.13 | -0,006063 | -0,15548 | -2,760235 | 0,006118 | 0,785048 |
| ENSG00000161609.5 | 0,007868 | -0,392939 | 2,758603 | 0,006148 | 0,785048 |
| ENSG00000271584.1 | -0,009142 | -0,029362 | -2,757589 | 0,006167 | 0,785048 |
| ENSG00000213903.4 | 0,002956 | 0,241993 | 2,749111 | 0,006325 | 0,785048 |

| | | | | | |
|---|---|---|---|---|---|
| ENSG00000272864.1 | 0,010828 | -0,810154 | 2,737644 | 0,006544 | 0,785048 |
| ENSG00000089063.10 | -0,006001 | 0,336244 | -2,734958 | 0,006596 | 0,785048 |
| ENSG00000165806.15 | 0,010299 | -0,58962 | 2,734354 | 0,006608 | 0,785048 |
| ENSG00000182054.5 | 0,002549 | 0,12435 | 2,730586 | 0,006682 | 0,785048 |
| ENSG00000100994.7 | -0,007928 | 0,149544 | -2,705768 | 0,00719 | 0,815579 |
| ENSG00000110063.4 | -0,002189 | 0,066737 | -2,698223 | 0,007351 | 0,815579 |
| ENSG00000104901.2 | 0,006413 | -0,297689 | 2,696689 | 0,007384 | 0,815579 |
| ENSG00000188163.6 | -0,005506 | -0,468029 | -2,687016 | 0,007597 | 0,815579 |
| ENSG00000151883.12 | 0,004599 | 0,060442 | 2,67736 | 0,007814 | 0,815579 |
| ENSG00000160321.10 | -0,004151 | 0,114034 | -2,671832 | 0,007941 | 0,815579 |
| ENSG00000233916.1 | -0,001267 | -0,085924 | -2,667805 | 0,008034 | 0,815579 |
| ENSG00000174004.5 | 0,004888 | -0,40818 | 2,667769 | 0,008035 | 0,815579 |
| ENSG00000164068.11 | 0,005495 | -0,206691 | 2,666799 | 0,008058 | 0,815579 |
| ENSG00000103035.6 | -0,00146 | -0,155233 | -2,657043 | 0,008289 | 0,820345 |
| ENSG00000259205.2 | -0,006338 | -0,32838 | -2,649933 | 0,008462 | 0,820345 |
| ENSG00000196961.8 | 0,004011 | -0,069191 | 2,645252 | 0,008577 | 0,820345 |
| ENSG00000188282.8 | -0,001482 | 0,013648 | -2,644181 | 0,008604 | 0,820345 |
| ENSG00000108219.10 | 0,003053 | -0,004859 | 2,624302 | 0,009111 | 0,85627 |
| ENSG00000163684.7 | -0,002894 | -0,133249 | -2,614934 | 0,009359 | 0,863291 |
| ENSG00000092094.6 | 0,007537 | 0,679356 | 2,601914 | 0,009713 | 0,863291 |
| ENSG00000170092.10 | -0,005368 | -0,277139 | -2,595455 | 0,009894 | 0,863291 |
| ENSG00000270127.1 | -0,007053 | -0,29531 | -2,592533 | 0,009977 | 0,863291 |
| ENSG00000175749.11 | -0,003588 | -0,230053 | -2,592128 | 0,009988 | 0,863291 |
| ENSG00000198093.6 | 0,004987 | -0,678677 | 2,584113 | 0,010218 | 0,863291 |
| ENSG00000214826.4 | -0,006853 | 0,162043 | -2,583037 | 0,010249 | 0,863291 |
| ENSG00000173145.7 | 0,00152 | 0,057778 | 2,58089 | 0,010312 | 0,863291 |
| ENSG00000175305.12 | 0,004084 | 0,187084 | 2,578735 | 0,010375 | 0,863291 |
| ENSG00000177432.6 | 0,002536 | 0,04331 | 2,57431 | 0,010506 | 0,863291 |
| ENSG00000244026.2 | 0,007073 | -0,265862 | 2,545587 | 0,011391 | 0,863291 |
| ENSG00000184207.8 | 0,003855 | 0,073439 | 2,54485 | 0,011415 | 0,863291 |
| ENSG00000234073.1 | -0,002097 | -0,055063 | -2,529187 | 0,011926 | 0,863291 |
| ENSG00000269552.1 | 0,002423 | -0,348573 | 2,526401 | 0,012019 | 0,863291 |
| ENSG00000182472.4 | -0,005529 | 0,575145 | -2,526035 | 0,012031 | 0,863291 |
| ENSG00000087206.12 | 0,001847 | -0,567561 | 2,520454 | 0,01222 | 0,863291 |
| ENSG00000198919.8 | -0,003999 | 0,165048 | -2,514747 | 0,012415 | 0,863291 |
| ENSG00000152223.8 | -0,002857 | -0,202925 | -2,514175 | 0,012435 | 0,863291 |
| ENSG00000077238.9 | -0,002567 | 0,025699 | -2,507791 | 0,012657 | 0,863291 |
| ENSG00000138459.4 | 0,004096 | 0,003448 | 2,506771 | 0,012693 | 0,863291 |
| ENSG00000075975.11 | 0,006273 | 0,259978 | 2,505745 | 0,012729 | 0,863291 |
| ENSG00000053108.12 | -0,005211 | -0,114232 | -2,504473 | 0,012774 | 0,863291 |
| ENSG00000272444.1 | 0,002444 | -0,121533 | 2,50265 | 0,012839 | 0,863291 |
| ENSG00000124678.13 | -0,004508 | -0,437876 | -2,501846 | 0,012867 | 0,863291 |
| ENSG00000267013.1 | -0,005076 | 0,389149 | -2,501364 | 0,012885 | 0,863291 |
| ENSG00000113621.10 | -0,001838 | -0,092882 | -2,499887 | 0,012937 | 0,863291 |
| ENSG00000137106.13 | 0,008703 | 0,33636 | 2,499158 | 0,012964 | 0,863291 |
| ENSG00000179397.13 | -0,001951 | 0,061308 | -2,498191 | 0,012998 | 0,863291 |
| ENSG00000245468.3 | 0,004637 | 0,105643 | 2,483733 | 0,013527 | 0,863291 |
| ENSG00000114670.9 | 0,004943 | -0,259239 | 2,482006 | 0,013591 | 0,863291 |
| ENSG00000078898.6 | -0,002299 | -0,326878 | -2,478574 | 0,01372 | 0,863291 |
| ENSG00000225490.1 | 0,00433 | -0,270619 | 2,478319 | 0,01373 | 0,863291 |
| ENSG00000219435.3 | 0,002511 | 0,061479 | 2,477879 | 0,013747 | 0,863291 |
| ENSG00000269067.1 | -0,002219 | -0,10466 | -2,469411 | 0,01407 | 0,863291 |
| ENSG00000231952.3 | -0,002251 | -0,064365 | -2,469146 | 0,01408 | 0,863291 |

| | | | | | |
|---|---|---|---|---|---|
| ENSG00000185838.9 | -0,006597 | -0,098261 | -2,468977 | 0,014086 | 0,863291 |
| ENSG00000129480.8 | -0,00397 | -0,06115 | -2,466209 | 0,014194 | 0,863291 |
| ENSG00000182103.3 | 0,003013 | 0,057822 | 2,46313 | 0,014314 | 0,863291 |
| ENSG00000237419.1 | -0,004765 | -0,312677 | -2,457973 | 0,014517 | 0,863291 |
| ENSG00000244753.2 | 0,001794 | 0,167035 | 2,457468 | 0,014537 | 0,863291 |
| ENSG00000204228.3 | 0,002906 | -0,206223 | 2,456487 | 0,014576 | 0,863291 |
| ENSG00000162604.8 | 0,001791 | 0,085074 | 2,451901 | 0,014759 | 0,863291 |
| ENSG00000197948.6 | 0,003271 | 0,114945 | 2,450199 | 0,014828 | 0,863291 |
| ENSG00000162366.3 | -0,001652 | 0,176429 | -2,446572 | 0,014975 | 0,864208 |
| ENSG00000258231.1 | -0,002337 | 0,141846 | -2,436594 | 0,015386 | 0,876085 |
| ENSG00000156603.10 | -0,00477 | 0,372828 | -2,432516 | 0,015557 | 0,876085 |
| ENSG00000182255.6 | -0,001907 | 0,085749 | -2,431744 | 0,01559 | 0,876085 |
| ENSG00000112799.4 | -0,00329 | 0,262603 | -2,428829 | 0,015713 | 0,876085 |
| ENSG00000173214.5 | 0,004745 | -0,325317 | 2,419108 | 0,016131 | 0,885161 |
| ENSG00000104714.9 | 0,003661 | 0,195977 | 2,415639 | 0,016283 | 0,885161 |
| ENSG00000104331.4 | 0,002016 | -0,050658 | 2,415111 | 0,016306 | 0,885161 |
| ENSG00000145439.7 | 0,005908 | -0,043305 | 2,411117 | 0,016482 | 0,885161 |
| ENSG00000235641.3 | 0,006202 | -0,65902 | 2,409622 | 0,016549 | 0,885161 |
| ENSG00000114904.8 | 0,003929 | -0,600552 | 2,399915 | 0,016986 | 0,901206 |
| ENSG00000153814.7 | 0,003932 | 0,461359 | 2,390254 | 0,017431 | 0,917418 |
| ENSG00000164136.12 | 0,003365 | 0,177275 | 2,385685 | 0,017645 | 0,921311 |
| ENSG00000250317.4 | -0,002208 | -0,066351 | -2,382717 | 0,017785 | 0,921321 |
| ENSG00000178381.7 | 0,004628 | -0,367538 | 2,377927 | 0,018013 | 0,925404 |
| ENSG00000230611.1 | -0,002971 | 0,070523 | -2,375189 | 0,018145 | 0,925404 |
| ENSG00000177483.7 | 0,009042 | 0,129103 | 2,368655 | 0,018463 | 0,928361 |
| ENSG00000196263.3 | -0,002972 | -0,20284 | -2,367212 | 0,018534 | 0,928361 |
| ENSG00000188687.11 | -0,004166 | -0,057199 | -2,363668 | 0,018709 | 0,928361 |
| ENSG00000089169.10 | 0,003303 | 0,189835 | 2,360885 | 0,018847 | 0,928361 |
| ENSG00000254685.2 | -0,006006 | -0,183625 | -2,359654 | 0,018909 | 0,928361 |
| ENSG00000146109.3 | 0,004325 | -0,206434 | 2,356181 | 0,019083 | 0,92999 |
| ENSG00000101198.10 | 0,001682 | 0,026957 | 2,337992 | 0,02002 | 0,951355 |
| ENSG00000127957.12 | 0,004332 | -0,031428 | 2,334356 | 0,020213 | 0,951355 |
| ENSG00000185324.17 | -0,008214 | -0,248293 | -2,33124 | 0,020379 | 0,951355 |
| ENSG00000128791.7 | -0,00628 | -0,126173 | -2,331055 | 0,020388 | 0,951355 |
| ENSG00000224557.3 | -0,006601 | 0,298742 | -2,330237 | 0,020432 | 0,951355 |
| ENSG00000219200.6 | -0,003223 | -0,069244 | -2,329888 | 0,020451 | 0,951355 |
| ENSG00000130758.3 | 0,005549 | -0,17681 | 2,3258 | 0,020671 | 0,951355 |
| ENSG00000269973.1 | -0,005041 | 0,116595 | -2,324792 | 0,020726 | 0,951355 |
| ENSG00000133597.5 | 0,003688 | 0,157674 | 2,321608 | 0,020899 | 0,951355 |
| ENSG00000105607.8 | 0,001141 | 0,072239 | 2,320353 | 0,020968 | 0,951355 |
| ENSG00000081320.6 | -0,002161 | -0,132406 | -2,311685 | 0,021447 | 0,954312 |
| ENSG00000151690.10 | 0,002628 | 0,048984 | 2,308992 | 0,021598 | 0,954312 |
| ENSG00000126467.6 | 0,002543 | -0,173323 | 2,30858 | 0,021622 | 0,954312 |
| ENSG00000273033.1 | -0,001445 | 0,116067 | -2,305301 | 0,021807 | 0,954312 |
| ENSG00000137672.8 | 0,003057 | -0,100911 | 2,301335 | 0,022033 | 0,954312 |
| ENSG00000139187.5 | 0,006105 | 0,273796 | 2,301 | 0,022052 | 0,954312 |
| ENSG00000137275.9 | 0,004581 | -0,589191 | 2,298859 | 0,022175 | 0,954312 |
| ENSG00000230772.1 | -0,002407 | 0,340118 | -2,294851 | 0,022406 | 0,954312 |
| ENSG00000103168.12 | -0,0095 | -0,557614 | -2,292146 | 0,022564 | 0,954312 |
| ENSG00000135702.10 | -0,00494 | 0,17044 | -2,286142 | 0,022917 | 0,954312 |
| ENSG00000033100.10 | -0,002018 | 0,068167 | -2,284034 | 0,023042 | 0,954312 |
| ENSG00000124508.12 | 0,00469 | 0,080014 | 2,283959 | 0,023047 | 0,954312 |
| ENSG00000117090.10 | 0,002933 | -0,307686 | 2,282908 | 0,023109 | 0,954312 |

| | | | | | |
|---|---|---|---|---|---|
| ENSG00000260973.1 | -0,005718 | 0,158376 | -2,272805 | 0,023719 | 0,954312 |
| ENSG00000204767.3 | 0,004265 | -0,065352 | 2,271864 | 0,023777 | 0,954312 |
| ENSG00000256667.2 | 0,003029 | 0,116796 | 2,27172 | 0,023785 | 0,954312 |
| ENSG00000145824.8 | -0,003196 | 0,332823 | -2,271635 | 0,023791 | 0,954312 |
| ENSG00000181523.8 | -0,001782 | 0,000872 | -2,270637 | 0,023852 | 0,954312 |
| ENSG00000180385.4 | -0,002355 | 0,096718 | -2,265912 | 0,024143 | 0,954312 |
| ENSG00000178802.13 | 0,003088 | 0,229075 | 2,257332 | 0,02468 | 0,954312 |
| ENSG00000198682.8 | -0,003273 | -0,139658 | -2,25639 | 0,024739 | 0,954312 |
| ENSG00000104522.11 | -0,002323 | -0,028058 | -2,252754 | 0,024971 | 0,954312 |
| ENSG00000271538.1 | 0,003578 | -0,251499 | 2,249056 | 0,025208 | 0,954312 |
| ENSG00000050327.10 | 0,004161 | -0,454452 | 2,248302 | 0,025256 | 0,954312 |
| ENSG00000112137.12 | -0,003398 | -0,313873 | -2,246044 | 0,025402 | 0,954312 |
| ENSG00000134873.5 | 0,004843 | -0,018083 | 2,244794 | 0,025483 | 0,954312 |
| ENSG00000165684.3 | -0,003111 | 0,029701 | -2,244633 | 0,025493 | 0,954312 |
| ENSG00000063438.12 | -0,001772 | 0,06233 | -2,243837 | 0,025545 | 0,954312 |
| ENSG00000149743.9 | -0,003099 | 0,269433 | -2,24159 | 0,025692 | 0,954312 |
| ENSG00000136982.5 | 0,006636 | 0,202238 | 2,241135 | 0,025722 | 0,954312 |
| ENSG00000170954.7 | 0,003229 | -0,084524 | 2,233626 | 0,026218 | 0,954312 |
| ENSG00000213221.4 | 0,008573 | 0,129525 | 2,232946 | 0,026263 | 0,954312 |
| ENSG00000163904.8 | 0,006867 | 0,551101 | 2,228326 | 0,026573 | 0,954312 |
| ENSG00000089327.10 | -0,008101 | -0,051519 | -2,224318 | 0,026844 | 0,954312 |
| ENSG00000138050.10 | -0,003086 | -0,107425 | -2,223191 | 0,02692 | 0,954312 |
| ENSG00000248587.2 | -0,00103 | -0,134463 | -2,222368 | 0,026977 | 0,954312 |
| ENSG00000198750.7 | 0,000537 | 0,022311 | 2,22191 | 0,027008 | 0,954312 |
| ENSG00000164889.8 | 0,002325 | 0,185832 | 2,221225 | 0,027055 | 0,954312 |
| ENSG00000072694.14 | -0,00358 | 0,445796 | -2,217163 | 0,027334 | 0,954312 |
| ENSG00000170689.8 | 0,001733 | 0,06512 | 2,211684 | 0,027714 | 0,954312 |
| ENSG00000204843.8 | 0,002538 | -0,032949 | 2,20937 | 0,027876 | 0,954312 |
| ENSG00000171421.8 | 0,004779 | -0,230766 | 2,205225 | 0,028169 | 0,954312 |
| ENSG00000171103.6 | 0,006433 | 0,104192 | 2,202793 | 0,028342 | 0,954312 |
| ENSG00000231742.1 | 0,004693 | 0,248186 | 2,200244 | 0,028524 | 0,954312 |
| ENSG00000196199.9 | 0,002511 | -0,369852 | 2,193708 | 0,028995 | 0,954312 |
| ENSG00000204592.5 | 0,002711 | 0,305577 | 2,190154 | 0,029254 | 0,954312 |
| ENSG00000184115.12 | 0,003871 | -0,425024 | 2,189974 | 0,029268 | 0,954312 |
| ENSG00000146386.7 | 0,003899 | -0,766305 | 2,187595 | 0,029442 | 0,954312 |
| ENSG00000213853.5 | -0,002294 | -0,035649 | -2,186867 | 0,029496 | 0,954312 |
| ENSG00000133704.5 | 0,00263 | -0,093958 | 2,186045 | 0,029557 | 0,954312 |
| ENSG00000137513.5 | -0,002543 | 0,306695 | -2,17976 | 0,030024 | 0,954312 |
| ENSG00000230305.2 | -0,007726 | 0,371221 | -2,178087 | 0,03015 | 0,954312 |
| ENSG00000111271.10 | -0,001154 | -0,005955 | -2,177118 | 0,030222 | 0,954312 |
| ENSG00000163554.7 | -0,00561 | 0,033581 | -2,176417 | 0,030275 | 0,954312 |
| ENSG00000079689.9 | 0,006441 | -0,221939 | 2,172805 | 0,030549 | 0,954312 |
| ENSG00000099251.10 | 0,002856 | 0,092673 | 2,172771 | 0,030551 | 0,954312 |
| ENSG00000129484.9 | 0,004392 | 0,070489 | 2,171515 | 0,030647 | 0,954312 |
| ENSG00000176209.7 | -0,00653 | -0,389433 | -2,167416 | 0,030961 | 0,954312 |
| ENSG00000204709.4 | -0,003874 | 0,025879 | -2,166147 | 0,031058 | 0,954312 |
| ENSG00000184752.8 | -0,004942 | -0,090254 | -2,163248 | 0,031283 | 0,954312 |
| ENSG00000064309.10 | -0,002122 | 0,592745 | -2,16322 | 0,031285 | 0,954312 |
| ENSG00000161944.12 | -0,00267 | -0,070792 | -2,16312 | 0,031293 | 0,954312 |
| ENSG00000178397.8 | -0,001403 | -0,090505 | -2,161883 | 0,031389 | 0,954312 |
| ENSG00000100234.11 | 0,002086 | 0,078464 | 2,155821 | 0,031863 | 0,954312 |
| ENSG00000090054.9 | 0,004161 | 0,099392 | 2,154323 | 0,031982 | 0,954312 |
| ENSG00000197279.3 | 0,002878 | -0,058421 | 2,153244 | 0,032067 | 0,954312 |

| | | | | | |
|---|---|---|---|---|---|
| ENSG00000226874.1 | 0,004927 | -0,741017 | 2,149383 | 0,032374 | 0,954312 |
| ENSG00000169035.7 | -0,002013 | -0,261787 | -2,148413 | 0,032452 | 0,954312 |
| ENSG00000178425.9 | -0,002307 | -0,078873 | -2,14705 | 0,032561 | 0,954312 |
| ENSG00000079974.13 | 0,006552 | 0,601467 | 2,146572 | 0,0326 | 0,954312 |
| ENSG00000078269.9 | 0,005296 | 0,538895 | 2,1465 | 0,032605 | 0,954312 |
| ENSG00000163568.9 | -0,001826 | -0,224375 | -2,143582 | 0,032841 | 0,954312 |
| ENSG00000105497.3 | 0,002813 | 0,081217 | 2,143465 | 0,03285 | 0,954312 |
| ENSG00000182141.5 | -0,004184 | -0,122841 | -2,143053 | 0,032883 | 0,954312 |
| ENSG00000187987.5 | -0,006575 | 0,385299 | -2,13651 | 0,033417 | 0,954312 |
| ENSG00000141639.7 | 0,001314 | -0,038554 | 2,136408 | 0,033425 | 0,954312 |
| ENSG00000196167.5 | -0,003697 | -0,00871 | -2,135624 | 0,03349 | 0,954312 |
| ENSG00000102580.10 | 0,003368 | 0,096157 | 2,133391 | 0,033674 | 0,954312 |
| ENSG00000072736.14 | -0,002422 | -0,061063 | -2,128609 | 0,034071 | 0,954312 |
| ENSG00000230373.4 | 0,007029 | -0,3061 | 2,128437 | 0,034086 | 0,954312 |
| ENSG00000162545.5 | 0,004782 | -0,684223 | 2,128222 | 0,034104 | 0,954312 |
| ENSG00000205084.6 | 0,00423 | -0,042028 | 2,128145 | 0,03411 | 0,954312 |
| ENSG00000204351.7 | 0,003945 | 0,201528 | 2,125331 | 0,034346 | 0,954312 |
| ENSG00000224132.2 | -0,002094 | 0,273641 | -2,124097 | 0,03445 | 0,954312 |
| ENSG00000143702.11 | 0,001542 | 0,100137 | 2,121993 | 0,034628 | 0,954312 |
| ENSG00000168995.9 | -0,001627 | 0,010851 | -2,120222 | 0,034778 | 0,954312 |
| ENSG00000250778.1 | -0,007301 | 0,11359 | -2,119135 | 0,03487 | 0,954312 |
| ENSG00000185742.6 | 0,003107 | 0,143908 | 2,118733 | 0,034904 | 0,954312 |
| ENSG00000125144.9 | -0,002988 | -0,349085 | -2,118379 | 0,034935 | 0,954312 |
| ENSG00000205583.9 | 0,008943 | 0,758408 | 2,116645 | 0,035083 | 0,954312 |
| ENSG00000260454.1 | -0,00131 | -0,061251 | -2,1164 | 0,035104 | 0,954312 |
| ENSG00000166825.9 | -0,004698 | -0,254513 | -2,115516 | 0,03518 | 0,954312 |
| ENSG00000180353.6 | 0,002121 | 0,099745 | 2,113919 | 0,035317 | 0,954312 |
| ENSG00000133243.4 | -0,003471 | 0,050788 | -2,111708 | 0,035508 | 0,954312 |
| ENSG00000100129.13 | 0,004814 | 0,318939 | 2,111232 | 0,035549 | 0,954312 |
| ENSG00000188707.4 | 0,002811 | -0,209405 | 2,105101 | 0,036083 | 0,954312 |
| ENSG00000248290.1 | -0,004598 | -0,079238 | -2,102106 | 0,036347 | 0,954312 |
| ENSG00000187824.4 | -0,004617 | 0,371939 | -2,096677 | 0,036829 | 0,954312 |
| ENSG00000272777.1 | 0,009528 | 0,058223 | 2,09312 | 0,037147 | 0,954312 |
| ENSG00000108799.8 | 0,001866 | 0,096561 | 2,091396 | 0,037303 | 0,954312 |
| ENSG00000187151.3 | 0,003658 | -0,065916 | 2,091286 | 0,037312 | 0,954312 |
| ENSG00000148296.5 | 0,004921 | 0,066333 | 2,089597 | 0,037465 | 0,954312 |
| ENSG00000203872.6 | -0,004421 | -0,259939 | -2,085065 | 0,037878 | 0,954312 |
| ENSG00000257239.1 | 0,003406 | 0,348851 | 2,083663 | 0,038006 | 0,954312 |
| ENSG00000102699.5 | 0,002651 | 0,315484 | 2,083338 | 0,038036 | 0,954312 |
| ENSG00000101306.6 | -0,003855 | 0,055094 | -2,08133 | 0,03822 | 0,954312 |
| ENSG00000088833.13 | 0,002959 | -0,119189 | 2,081021 | 0,038249 | 0,954312 |
| ENSG00000151665.8 | -0,001371 | 0,029664 | -2,080207 | 0,038324 | 0,954312 |
| ENSG00000113719.11 | 0,00157 | 0,002055 | 2,079792 | 0,038362 | 0,954312 |
| ENSG00000216901.1 | -0,007158 | 0,363555 | -2,079759 | 0,038365 | 0,954312 |
| ENSG00000225422.3 | -0,00122 | 0,059478 | -2,07957 | 0,038383 | 0,954312 |
| ENSG00000148908.10 | -0,001248 | 0,391008 | -2,078627 | 0,03847 | 0,954312 |
| ENSG00000204920.6 | -0,003916 | -0,346857 | -2,076068 | 0,038708 | 0,954312 |
| ENSG00000164342.8 | 0,002742 | -0,272842 | 2,075679 | 0,038744 | 0,954312 |
| ENSG00000205595.3 | 0,005636 | 0,133716 | 2,075447 | 0,038766 | 0,954312 |
| ENSG00000227755.1 | 0,002454 | 0,007027 | 2,07058 | 0,039222 | 0,954312 |
| ENSG00000090857.9 | -0,001647 | -0,26602 | -2,06592 | 0,039663 | 0,954312 |
| ENSG00000247157.2 | -0,009279 | -0,475831 | -2,065197 | 0,039732 | 0,954312 |
| ENSG00000258429.1 | -0,002732 | -0,133131 | -2,059045 | 0,040321 | 0,954312 |

| | | | | | |
|---|---|---|---|---|---|
| ENSG00000260459.2 | -0,003329 | 0,502716 | -2,056157 | 0,0406 | 0,954312 |
| ENSG00000121406.4 | 0,000781 | 0,007022 | 2,055823 | 0,040633 | 0,954312 |
| ENSG00000144230.12 | 0,006771 | 0,735355 | 2,055498 | 0,040664 | 0,954312 |
| ENSG00000134962.6 | -0,003923 | -0,376733 | -2,053461 | 0,040863 | 0,954312 |
| ENSG00000124357.8 | -0,003613 | -0,21437 | -2,052455 | 0,040961 | 0,954312 |
| ENSG00000225507.1 | -0,001731 | 0,003716 | -2,047871 | 0,041411 | 0,954312 |
| ENSG00000151893.10 | 0,005317 | -0,454355 | 2,047576 | 0,04144 | 0,954312 |
| ENSG00000271590.1 | 0,001342 | -0,049177 | 2,046069 | 0,041589 | 0,954312 |
| ENSG00000108244.12 | -0,003679 | -0,049506 | -2,046008 | 0,041595 | 0,954312 |
| ENSG00000178917.10 | 0,003652 | 0,019202 | 2,044592 | 0,041736 | 0,954312 |
| ENSG00000185880.8 | -0,005907 | -0,134038 | -2,044396 | 0,041755 | 0,954312 |
| ENSG00000119446.9 | -0,003575 | 0,93851 | -2,044101 | 0,041784 | 0,954312 |
| ENSG00000058085.10 | 0,002659 | 0,11413 | 2,042421 | 0,041952 | 0,954312 |
| ENSG00000115310.13 | -0,003118 | -0,139343 | -2,041376 | 0,042056 | 0,954312 |
| ENSG00000272902.1 | -0,00327 | -0,026221 | -2,039661 | 0,042228 | 0,954312 |
| ENSG00000175220.7 | -0,003154 | 0,346616 | -2,03812 | 0,042383 | 0,954312 |
| ENSG00000132128.12 | -0,001364 | -0,092782 | -2,033909 | 0,042808 | 0,954312 |
| ENSG00000243566.2 | 0,007233 | -0,426348 | 2,032558 | 0,042945 | 0,954312 |
| ENSG00000187634.6 | 0,001938 | 0,224612 | 2,03022 | 0,043184 | 0,954312 |
| ENSG00000122643.14 | 0,003184 | 0,073347 | 2,029759 | 0,043231 | 0,954312 |
| ENSG00000154122.8 | 0,001702 | 0,055218 | 2,029189 | 0,043289 | 0,954312 |
| ENSG00000185040.11 | -0,002978 | 0,079143 | -2,029053 | 0,043303 | 0,954312 |
| ENSG00000196922.6 | -0,005161 | 0,135131 | -2,028368 | 0,043373 | 0,954312 |
| ENSG00000272130.1 | -0,000763 | -0,111346 | -2,028199 | 0,043391 | 0,954312 |
| ENSG00000145029.7 | -0,003561 | 0,411043 | -2,026156 | 0,043601 | 0,954312 |
| ENSG00000259005.1 | 0,003483 | 0,22076 | 2,025247 | 0,043695 | 0,954312 |
| ENSG00000163331.6 | -0,00267 | -0,222604 | -2,024916 | 0,043729 | 0,954312 |
| ENSG00000016602.8 | -0,002327 | -0,22682 | -2,022339 | 0,043996 | 0,954312 |
| ENSG00000188295.10 | 0,003637 | 0,111184 | 2,020433 | 0,044194 | 0,954312 |
| ENSG00000243710.3 | 0,008263 | -0,815088 | 2,019982 | 0,044241 | 0,954312 |
| ENSG00000156170.8 | 0,00168 | 0,106985 | 2,018331 | 0,044414 | 0,954312 |
| ENSG00000196460.8 | 0,005519 | 0,647866 | 2,018092 | 0,044439 | 0,954312 |
| ENSG00000170571.7 | -0,001638 | 0,133797 | -2,017454 | 0,044506 | 0,954312 |
| ENSG00000213453.3 | 0,002355 | 0,235613 | 2,013871 | 0,044883 | 0,954312 |
| ENSG00000204428.8 | 0,004472 | -0,238201 | 2,013226 | 0,044951 | 0,954312 |
| ENSG00000114841.13 | 0,002181 | 0,134397 | 2,012516 | 0,045026 | 0,954312 |
| ENSG00000113119.8 | 0,003151 | 0,164157 | 2,011547 | 0,045129 | 0,954312 |
| ENSG00000227671.3 | -0,00374 | -0,118338 | -2,008006 | 0,045506 | 0,954312 |
| ENSG00000227558.4 | -0,001727 | 0,60828 | -2,007943 | 0,045513 | 0,954312 |
| ENSG00000229891.1 | 0,005934 | -0,655594 | 2,004733 | 0,045857 | 0,954312 |
| ENSG00000197614.6 | 0,002346 | 0,038197 | 2,002502 | 0,046097 | 0,954312 |
| ENSG00000125912.6 | -0,004851 | -0,296646 | -2,001243 | 0,046234 | 0,954312 |
| ENSG00000122085.12 | -0,004049 | 0,406853 | -2,000621 | 0,046301 | 0,954312 |
| ENSG00000106070.13 | -0,001416 | 0,089265 | -2,000602 | 0,046303 | 0,954312 |
| ENSG00000021762.15 | -0,003732 | 0,183228 | -1,999154 | 0,04646 | 0,954312 |
| ENSG00000272195.1 | -0,006859 | 0,614026 | -1,998397 | 0,046543 | 0,954312 |
| ENSG00000152601.13 | -0,000886 | -0,030737 | -1,996478 | 0,046752 | 0,954312 |
| ENSG00000180881.15 | -0,003025 | -0,059424 | -1,994514 | 0,046967 | 0,954312 |
| ENSG00000076924.7 | -0,002453 | -0,147875 | -1,992664 | 0,047171 | 0,954312 |
| ENSG00000113946.3 | 0,003178 | -0,267454 | 1,992095 | 0,047234 | 0,954312 |
| ENSG00000178226.6 | -0,003466 | -0,394779 | -1,991094 | 0,047344 | 0,954312 |
| ENSG00000170837.2 | -0,005242 | -0,075083 | -1,990593 | 0,047399 | 0,954312 |
| ENSG00000256673.1 | -0,005965 | 0,23391 | -1,990558 | 0,047403 | 0,954312 |

| | | | | | |
|---|---|---|---|---|---|
| ENSG00000160325.10 | -0,002041 | -0,164047 | -1,990197 | 0,047443 | 0,954312 |
| ENSG00000071243.11 | -0,002176 | -0,285353 | -1,98907 | 0,047568 | 0,954312 |
| ENSG00000182685.3 | 0,001447 | 0,044954 | 1,987522 | 0,04774 | 0,954312 |
| ENSG00000168404.8 | -0,002189 | 0,182167 | -1,984442 | 0,048084 | 0,954312 |
| ENSG00000214402.6 | -0,003147 | -0,593286 | -1,984239 | 0,048107 | 0,954312 |
| ENSG00000105894.7 | 0,001644 | 0,113458 | 1,981059 | 0,048464 | 0,954312 |
| ENSG00000101158.8 | 0,006236 | -0,311799 | 1,980883 | 0,048484 | 0,954312 |
| ENSG00000132406.7 | 0,004286 | 0,688558 | 1,979629 | 0,048625 | 0,954312 |
| ENSG00000172014.11 | -0,002304 | -0,032223 | -1,976089 | 0,049027 | 0,954312 |
| ENSG00000163737.3 | -0,002091 | 0,043567 | -1,972639 | 0,049421 | 0,954312 |
| ENSG00000078053.12 | 0,006884 | 0,089382 | 1,970773 | 0,049635 | 0,954312 |
