# Supplementary material for: The transcriptome-wide association search for genes and genetic variants which associate with BMI and gestational weight gain in women with type 1 diabetes
Source: Mol Med. 2021 Jan 20;27:6. doi: 10.1186/s10020-020-00266-z (PMC7818927; doi:10.1186/s10020-020-00266-z)
Supplement: Supplementary file 3 — Additional file 3: Table S2. The overlap of PrediXcan results in Subcutaneous and Visceral Adipose Tissue in the Giant cohort. [file 10020_2020_266_MOESM3_ESM.pdf]

"gene"	"gene_name"	"subc_beta"	"subc_pval"	"subc_r2"	"visc_beta"	"visc_pval"	"visc_r2"
"ENSG00000251417.1"	"RP11-1348G14.4"	"-0.06667691	"3.0931112950	"0.1820481	"-0.101255838	"9.453851184	"0.10147939147314"
"ENSG00000271623.1"	"RP11-435I10.5"	"-0.13348873	"1.1540476559	"0.0626857	"NA"	"NA"	"NA"
"ENSG00000138092.6"	"CENPO"	"0.134757201	"1.7445077848	"0.0694877	"0.108589709	"1.140445222	"0.0617553805819642"
"ENSG00000172247.3"	"C1QTNF4"	"-0.03331838	"9.3412243193	"0.3961509	"-0.051798493	"3.746067480	"0.179711844107764"
"ENSG00000205609.8"	"EIF3CL"	"0.279336095	"1.0130516603	"0.0140185	"NA"	"NA"	"NA"
"ENSG00000138031.10"	"ADCY3"	"0.068319005	"1.5729319166	"0.1476662	"0.096172253	"8.296498222	"0.0886567846109578"
"ENSG00000178952.4"	"TUFM"	"0.055122293	"2.8917716161	"0.1492952	"0.048169638	"1.727256902	"0.122322050766206"
"ENSG00000197165.6"	"SULT1A2"	"0.042624307	"5.1457866357	"0.3258897	"0.055635596	"9.602047378	"0.332229074890563"
"ENSG00000165915.9"	"SLC39A13"	"0.152460598	"1.1704370828	"0.0453425	"0.098590207	"3.096786623	"0.0664381628259576"
"ENSG00000178188.10"	"SH2B1"	"-0.08338043	"1.1199598481	"0.1457478	"-0.181617310	"1.341414227	"0.0539087682758526"
"ENSG00000259982.1"	"CDC37P1"	"0.062078039	"4.4617502118	"0.2250959	"0.050288000	"1.435340145	"0.194395633120258"
"ENSG00000184110.10"	"EIF3C"	"0.064088329	"7.2721754302	"0.1047589	"NA"	"NA"	"NA"
"ENSG00000270424.1"	"RP11-1348G14.6"	"0.133398612	"1.8966798406	"0.0748002	"NA"	"NA"	"NA"
"ENSG00000188779.6"	"SKOR1"	"0.040213052	"3.7742362224	"0.2052055	"0.060525709	"1.842198434	"0.0867209557154057"
"ENSG00000134575.5"	"ACP2"	"0.057008538	"1.0418539877	"0.0658173	"0.045795779	"0.000504105	"0.0658247027825383"
"ENSG00000233232.2"	"NPIPB7"	"-0.12244556	"1.0556624098	"0.0832560	"-0.250698733	"2.359598679	"0.0216539526281683"
"ENSG00000137764.15"	"MAP2K5"	"0.032329923	"2.4033825766	"0.2557380	"0.053810646	"1.035864650	"0.147445257188787"
"ENSG00000163431.11"	"LMOD1"	"-0.03961446	"4.7378161078	"0.1816524	"-0.053525041	"0.000384565	"0.0585647552000455"
"ENSG00000213277.3"	"MARCKSL1P1"	"0.065357824	"8.6513658743	"0.0609632	"NA"	"NA"	"NA"
"ENSG00000168488.14"	"ATXN2L"	"-0.08144572	"9.9940643712	"0.0372463	"NA"	"NA"	"NA"
"ENSG00000141452.5"	"C18orf8"	"-0.03327526	"1.2815825623	"0.2052262	"-0.059992799	"2.784023938	"0.17530739093144"
"ENSG00000152359.10"	"POC5"	"-0.03858647	"2.4404779641	"0.1597380	"-0.023586844	"4.935728372	"0.163543278234978"
"ENSG00000141458.8"	"NPC1"	"-0.02505271	"6.2480620430	"0.3705401	"-0.036407798	"2.964528811	"0.232721295065458"
"ENSG00000124562.5"	"SNRPC"	"-0.04385111	"7.7177139708	"0.1837089	"-0.054278450	"4.324025976	"0.104134630833732"
"ENSG00000170092.10"	"AC018720.10"	"0.050133534	"1.0756011828	"0.1574482	"0.053962357	"6.986709176	"0.16995830542368"
"ENSG00000103550.9"	"KNOP1"	"-0.02005146	"1.6737167486	"0.4083283	"-0.018500091	"3.780608759	"0.495986659984754"
"ENSG00000169592.10"	"INO80E"	"-0.04910622	"1.7949993778	"0.1991673	"-0.071620994	"3.370099347	"0.185861506907159"
"ENSG00000205482.7"	"AC007000.12"	"-0.06676290	"1.9301971397	"0.0686964	"-0.057083507	"6.493367588	"0.0982723157558894"
"ENSG00000127957.12"	"PMS2P3"	"-0.07083377	"2.3126233430	"0.2019110	"-0.052724824	"3.403703074	"0.15015673381797"
"ENSG00000065060.12"	"UHRF1BP1"	"0.021062002	"2.5653003531	"0.4459560	"0.023777629	"2.134188454	"0.420793009186235"
"ENSG00000103510.15"	"KAT8"	"0.029934714	"2.8648761791	"0.2883132	"0.033444913	"2.310132574	"0.2619403448489"
"ENSG00000005955.8"	"GGNBP2"	"-0.04744568	"4.7617944035	"0.1935548	"-0.067992107	"1.264466432	"0.0695162076436004"
"ENSG00000162613.12"	"FUBP1"	"-0.05526195	"4.9068804313	"0.0631050	"NA"	"NA"	"NA"

"ENSG00000242435.1"	"UPK3BP1"	"0.038444909"	"5.2862351565"	"0.2439231"	"NA"	"NA"	"NA"
"ENSG00000175426.6"	"PCSK1"	"-0.06740249"	"5.4690375175"	"0.0523189"	"NA"	"NA"	"NA"
"ENSG00000112530.7"	"PACRG"	"-0.04659581"	"5.8711550424"	"0.1714551"	"-0.070880863"	"1.389469345"	"0.068979294200447"
"ENSG00000141140.12"	"MYO19"	"0.052654440"	"6.6313260383"	"0.1268367"	"0.060721432"	"3.604199057"	"0.20126348006017"
"ENSG00000169047.5"	"IRS1"	"0.039727276"	"7.8741788305"	"0.1059304"	"0.026378230"	"0.012100275"	"0.0278353800470032"
"ENSG00000186704.8"	"DTX2P1"	"0.045235441"	"8.0111695152"	"0.2222070"	"0.039033100"	"4.416790296"	"0.151714780317636"
"ENSG00000174243.5"	"DDX23"	"0.092403340"	"8.0504315424"	"0.0405622"	"NA"	"NA"	"NA"
"ENSG00000196502.7"	"SULT1A1"	"-0.03033964"	"8.6778501260"	"0.0322606"	"-0.061729963"	"1.419768053"	"0.0591828140506442"
"ENSG00000213542.3"	"RP11-467H10.1"	"0.086343603"	"1.0783773575"	"0.0484221"	"NA"	"NA"	"NA"
"ENSG00000272622.1"	"RP11-395N3.2"	"0.078426394"	"1.1153028017"	"0.0729026"	"0.072089953"	"2.583331089"	"0.0534490921715385"
"ENSG00000148346.7"	"LCN2"	"0.103221967"	"1.3682568852"	"0.0144570"	"NA"	"NA"	"NA"
"ENSG00000179195.11"	"ZNF664"	"0.054640230"	"1.4158660517"	"0.0677999"	"0.106546758"	"8.172247315"	"0.064079511494546"
"ENSG00000188211.4"	"NCR3LG1"	"0.080588487"	"1.4257819094"	"0.0720176"	"0.061544563"	"0.006997621"	"0.0675072466760056"
"ENSG00000136944.13"	"LMX1B"	"-0.06648606"	"1.4338716906"	"0.0879152"	"NA"	"NA"	"NA"
"ENSG00000167118.6"	"URM1"	"-0.04087731"	"1.5691666188"	"0.1491153"	"-0.042938659"	"4.835641065"	"0.133173093758787"
"ENSG00000198909.3"	"MAP3K3"	"-0.08471874"	"2.0013532416"	"0.0851970"	"-0.065362594"	"9.613026785"	"0.0520762504164482"
"ENSG00000247400.3"	"DNAJC3-AS1"	"-0.07541593"	"2.0537388859"	"0.0844157"	"-0.041357877"	"0.013237876"	"0.0710324301942108"
"ENSG00000090238.7"	"YPEL3"	"0.032699645"	"2.1095804916"	"0.4512655"	"0.055082548"	"0.000159688"	"0.233484272468641"
"ENSG00000273281.1"	"RP11-339B21.14"	"-0.06902241"	"2.2242255684"	"0.0725609"	"-0.022933102"	"0.119060719"	"0.0352761123281821"
"ENSG00000117569.14"	"PTBP2"	"0.068225019"	"2.5753518295"	"0.1167434"	"0.064619899"	"0.000226410"	"0.0832485115430199"
"ENSG00000119242.4"	"CCDC92"	"0.029796637"	"2.6538121577"	"0.1851025"	"0.019577004"	"0.000788195"	"0.246427260686863"
"ENSG00000135213.8"	"POM121C"	"-0.10136867"	"2.7554199887"	"0.0606035"	"-0.077366816"	"4.750181809"	"0.0480477971693398"
"ENSG00000106992.13"	"AK1"	"0.056032247"	"2.9347674046"	"0.0283183"	"NA"	"NA"	"NA"
"ENSG00000103351.8"	"CLUAP1"	"0.024890204"	"2.9610444540"	"0.3858153"	"0.023343983"	"0.000139480"	"0.335902791396587"
"ENSG00000165916.4"	"PSMC3"	"0.035728323"	"4.0573857443"	"0.1107514"	"0.024337190"	"0.318205243"	"0.0477723798238283"
"ENSG00000198892.6"	"SHISA4"	"-0.04922429"	"4.0674467287"	"0.0713150"	"-0.123325732"	"2.275476244"	"0.0622038000932466"
"ENSG00000105726.12"	"ATP13A1"	"-0.04197791"	"5.1911658402"	"0.1191778"	"NA"	"NA"	"NA"
"ENSG00000175161.9"	"CADM2"	"0.033984198"	"6.1753081450"	"0.1134007"	"0.053468253"	"0.000866769"	"0.0644281011268776"
"ENSG00000075413.13"	"MARK3"	"-0.05193571"	"7.9160220642"	"0.0708853"	"NA"	"NA"	"NA"
"ENSG00000132563.11"	"REEP2"	"-0.05137202"	"8.1073746858"	"0.0866359"	"0.001368830"	"0.976309929"	"0.0171122697288911"
"ENSG00000169203.12"	"RP11-231C14.4"	"0.026974436"	"8.2680842665"	"0.1999081"	"0.030661456"	"0.012481349"	"0.0668502083634367"
"ENSG00000261056.2"	"RP11-454F8.2"	"0.057209396"	"9.0292625121"	"0.0548971"	"NA"	"NA"	"NA"
"ENSG00000162877.8"	"PM20D1"	"0.020442793"	"0.0001096487"	"0.4077297"	"0.016808692"	"8.708661046"	"0.486404369155442"
"ENSG00000188976.6"	"NOC2L"	"-0.11895919"	"0.0001134606"	"0.1852550"	"NA"	"NA"	"NA"

"ENSG00000072736.14"	"NFATC3"	"-0.06411035	"0.0001145585	"0.0731154	"-0.060452476	"4.231065897	"0.0387937120076033"
"ENSG00000115556.9"	"PLCD4"	"-0.14681094	"0.0001171089	"0.0311693	"NA"	"NA"	"NA"
"ENSG00000064995.12"	"TAF11"	"-0.03943837	"0.0001177409	"0.0437410	"NA"	"NA"	"NA"
"ENSG00000004534.10"	"RBM6"	"-0.01842695	"0.0001187327	"0.5213719	"-0.024496634	"0.000123514	"0.45180501292702"
"ENSG00000259207.3"	"ITGB3"	"-0.04527753	"0.0001188434	"0.0842316	"-0.031019084	"0.002011383	"0.0347462577246825"
"ENSG00000122025.10"	"FLT3"	"0.088818456	"0.0001227325	"0.0589477	"0.028759900	"0.139830493	"0.0188499571257914"
"ENSG00000122008.11"	"POLK"	"-0.05723787	"0.0001243598	"0.0262094	"NA"	"NA"	"NA"
"ENSG00000259347.1"	"RP11-798K3.2"	"0.031812876	"0.0001257913	"0.0226819	"NA"	"NA"	"NA"
"ENSG00000251288.2"	"RP11-10L12.2"	"0.039078262	"0.0001265625	"0.1034674	"NA"	"NA"	"NA"
"ENSG00000149084.7"	"HSD17B12"	"-0.01603796	"0.0001293558	"0.5031861	"-0.013129986	"0.000597886	"0.571636342663567"
"ENSG00000205583.9"	"STAG3L1"	"-0.03328455	"0.0001329276	"0.4136618	"-0.026199553	"0.000766088	"0.387792612768363"
"ENSG00000230069.3"	"LRRC37A15P"	"0.032978788	"0.0001481723	"0.1337500	"0.023825232	"0.000474154	"0.152272346605457"
"ENSG00000157322.12"	"CLEC18A"	"-0.01641468	"0.0001536006	"0.4824477	"-0.019642756	"0.000171113	"0.477523249159963"
"ENSG00000187145.10"	"MRPS21"	"0.044924433	"0.0001570331	"0.0424163	"0.025737029	"4.420899762	"0.218495214099117"
"ENSG00000161904.7"	"LEMD2"	"-0.05496561	"0.0001825905	"0.0774297	"-0.091366185	"0.001628028	"0.0194239555934345"
"ENSG00000135913.6"	"USP37"	"0.042531072	"0.0001933317	"0.1309568	"0.038032923	"0.001149181	"0.0776458154250986"
"ENSG00000101193.6"	"GID8"	"-0.11349807	"0.0001947462	"0.0253256	"0.006601063	"0.590617935	"0.039523323013825"
"ENSG00000198917.7"	"C9orf114"	"0.049708207	"0.0002055337	"0.1657059	"0.023259633	"0.006211145	"0.195957729949005"
"ENSG00000162517.8"	"PEF1"	"0.059869059	"0.0002250070	"0.0612829	"NA"	"NA"	"NA"
"ENSG00000240280.2"	"TCAM1P"	"0.027977293	"0.0002256463	"0.2319374	"0.024750645	"0.007713682	"0.155171211369222"
"ENSG00000198646.9"	"NCOA6"	"-0.08421803	"0.0002502594	"0.0356806	"-0.047142144	"0.001119639	"0.0939724279750285"
"ENSG00000174482.6"	"LINGO2"	"0.143331918	"0.0002503384	"0.0301494	"NA"	"NA"	"NA"
"ENSG00000069275.12"	"NUCKS1"	"0.046379463	"0.0002570484	"0.0876486	"0.023024387	"0.007126575	"0.056217668440048"
"ENSG00000136930.8"	"PSMB7"	"-0.04141874	"0.0002600925	"0.0858600	"NA"	"NA"	"NA"
"ENSG00000033030.9"	"ZCCHC8"	"0.156190131	"0.0002614571	"0.0449902	"NA"	"NA"	"NA"
"ENSG00000103599.15"	"IQCH"	"0.043060008	"0.0002631545	"0.0991533	"0.050240655	"0.000254255	"0.0651907210137005"
"ENSG00000110881.7"	"ASIC1"	"-0.05808988	"0.0002930156	"0.0286449	"0.008629272	"0.683845292	"0.0699670018124355"
"ENSG00000174915.7"	"PTDSS2"	"0.057244170	"0.0003070719	"0.0616084	"0.056341876	"0.000240789	"0.0629912483305046"
"ENSG00000269963.1"	"RP11-73M18.9"	"-0.02257154	"0.0003078149	"0.3572635	"-0.018355811	"0.000713058	"0.321052491049284"
"ENSG00000166436.11"	"TRIM66"	"0.017699574	"0.0003154376	"0.5899782	"0.021884205	"8.533845293	"0.462742209192958"
"ENSG00000224997.1"	"AL049840.1"	"-0.01728459	"0.0003386090	"0.4425799	"-0.022895420	"0.000246722	"0.344014687924206"
"ENSG00000120093.7"	"HOXB3"	"0.048132324	"0.0003485487	"0.0720768	"0.118173157	"0.122350773	"0.0579128330458406"
"ENSG00000248971.2"	"KRT8P46"	"0.042272043	"0.0003499975	"0.0822627	"0.029831881	"0.001073213	"0.0832319488578109"
"ENSG00000198478.6"	"SH3BGRL2"	"0.039715759	"0.0003547553	"0.0924454	"0.038256611	"0.000428651	"0.15362200237689"

"ENSG00000169972.7"	"PUSL1"	"-0.13190556	"0.0003572368	"0.0598775	"NA"	"NA"	"NA"	
"ENSG00000260302.1"	"RP11-973H7.1"	"-0.03216569	"0.0003623215	"0.1395457	"-0.068819716	"2.631460599	"0.0240455330795346"	
"ENSG00000138380.13"	"CARF"	"0.027181875	"0.0003681126	"0.1414803	"NA"	"NA"	"NA"	
"ENSG00000242193.5"	"RP11-568K15.1"	"-0.03326376	"0.0003827287	"0.1783710	"-0.042577925	"0.001555712	"0.0586001491968955"	
"ENSG00000130779.15"	"CLIP1"	"0.074708402	"0.0003941907	"0.0578381	"NA"	"NA"	"NA"	
"ENSG00000108592.12"	"FTSJ3"	"-0.01710303	"0.0004121725	"0.5629079	"-0.018797358	"0.000438876	"0.474101684716796"	
"ENSG00000130520.6"	"LSM4"	"0.029100121	"0.0004234854	"0.1742598	"0.0020961991	"0.975273905	"0.0986672315049699"	
"ENSG00000232810.3"	"TNF"	"-0.14088513	"0.0004266595	"0.0144652	"NA"	"NA"	"NA"	
"ENSG00000159640.10"	"ACE"	"0.055306739	"0.0004817048	"0.0457366	"0.117532178	"0.000264377	"0.0408739385048923"	
"ENSG00000111237.14"	"VPS29"	"0.059372543	"0.0004897596	"0.0453185	"0.043159949	"2.154914583	"0.137480750690102"	
"ENSG00000129197.10"	"RPAIN"	"-0.04074045	"0.0004979959	"0.1491742	"-0.030509106	"0.008061637	"0.1392222137112"	
"ENSG00000243477.1"	"NAT6"	"0.070013924	"0.0005226230	"0.0571406	"NA"	"NA"	"NA"	
"ENSG00000173511.5"	"VEGFB"	"0.031577181	"0.0005283847	"0.1682705	"NA"	"NA"	"NA"	
"ENSG00000166166.8"	"TRMT61A"	"-0.03557252	"0.0005310686	"0.0621576	"-0.031873338	"0.299210884	"0.0209235408240039"	
"ENSG00000260911.1"	"RP11-196G11.2"	"0.040530308	"0.0005390719	"0.2365104	"0.059819446	"0.000469495	"0.209834473968504"	
"ENSG00000187764.7"	"SEMA4D"	"-0.05273314	"0.0005446581	"0.0694583	"NA"	"NA"	"NA"	
"ENSG00000197622.8"	"CDC42SE1"	"0.042246104	"0.0005482348	"0.0468489	"NA"	"NA"	"NA"	
"ENSG00000124614.9"	"RPS10"	"0.068647892	"0.0005642055	"0.1308610	"0.007728961	"0.802067725	"0.0473618101052752"	
"ENSG00000085063.10"	"CD59"	"0.022250864	"0.0005648354	"0.2480896	"0.022935725	"0.004813643	"0.151163683100419"	
"ENSG00000245719.1"	"RP11-34F13.2"	"0.031549332	"0.0005697534	"0.0948863	"NA"	"NA"	"NA"	
"ENSG00000187953.6"	"PMS2CL"	"-0.02616464	"0.0005927252	"0.2047222	"-0.011252138	"0.143379010	"0.260096161666985"	
"ENSG00000119509.8"	"INVS"	"0.026766567	"0.0006067594	"0.1366930	"0.019767550	"0.000160425	"0.325706293373861"	
"ENSG00000176155.14"	"CCDC57"	"-0.02623857	"0.0006242972	"0.1975021	"-0.080541587	"0.000258097	"0.159722222549558"	
"ENSG00000141570.6"	"CBX8"	"0.091339538	"0.0006441055	"0.2104902	"0.036650458	"0.000677391	"0.235289241263964"	
"ENSG00000108604.11"	"SMARCD2"	"-0.11531001	"0.0006692102	"0.0336484	"NA"	"NA"	"NA"	
"ENSG00000198231.8"	"DDX42"	"0.104404839	"0.0006983387	"0.0591169	"0.107591516	"0.000650803	"0.0455203464228193"	
"ENSG00000115137.7"	"DNAJC27"	"0.060370008	"0.0007130933	"0.0429977	"NA"	"NA"	"NA"	
"ENSG00000076003.4"	"MCM6"	"-0.04739690	"0.0007198725	"0.0169503	"-0.048160264	"0.024542666	"0.027317379750262"	
"ENSG00000138175.8"	"ARL3"	"-0.03865703	"0.0007401733	"0.0842578	"-0.022804796	"0.002873459	"0.122725334084249"	
"ENSG00000160685.9"	"ZBTB7B"	"-0.12886961	"0.0007482998	"0.0526935	"-0.055104375	"0.224515544	"0.0146155186866186"	
"ENSG00000213889.6"	"PPM1N"	"0.086438922	"0.0007548252	"0.1198397	"0.039616356	"0.008887750	"0.122567188424307"	
"ENSG00000197576.9"	"HOXA4"	"-0.05087879	"0.0007721308	"0.0609979	"NA"	"NA"	"NA"	
"ENSG00000158321.11"	"AUTS2"	"-0.07889250	"0.0007740847	"0.0286558	"NA"	"NA"	"NA"	
"ENSG00000001617.7"	"SEMA3F"	"-0.05182375	"0.0007887001	"0.0332230	"-0.108483528	"0.004090334	"0.0392725749559869"	

"ENSG00000164989.11"	"CCDC171"	"-0.03062093	"0.0008289744"	"0.0759290"	"-0.001181345"	"0.946829984"	"0.117056593502566"
"ENSG00000076685.14"	"NT5C2"	"0.033436865"	"0.0008423095"	"0.0930196"	"0.031737460"	"0.000567046"	"0.141697061436479"
"ENSG00000197262.8"	"CCL4L2"	"-0.04211101	"0.0008476823"	"0.0204495"	"-0.054356897"	"0.056810262"	"0.0370032879267747"
"ENSG00000138002.10"	"IFT172"	"0.055889390"	"0.0008751778"	"0.0153725"	"NA"	"NA"	"NA"
"ENSG00000166136.11"	"NDUFB8"	"-0.04918913	"0.0008892912"	"0.0259397"	"NA"	"NA"	"NA"
"ENSG00000143434.11"	"SEMA6C"	"0.027327740"	"0.0009098037"	"0.1592064"	"0.075488630"	"0.000564315"	"0.029707704627046"
"ENSG00000164944.7"	"KIAA1429"	"0.086199806"	"0.0009330982"	"0.0164437"	"NA"	"NA"	"NA"
"ENSG00000250091.2"	"DNAH10OS"	"0.034601170"	"0.0009856568"	"0.1152739"	"0.039107124"	"0.002532254"	"0.100152183383834"
"ENSG00000165917.5"	"SAE1"	"NA"	"NA"	"NA"	"-0.108585170"	"1.403805865"	"0.0476385633885708"
"ENSG00000142230.7"	"RP11-339B21.15"	"-0.01100803	"0.290213070"	"0.0372299"	"-0.183791086"	"2.809602489"	"0.0369045604712336"
"ENSG00000272960.1"	"YWHAZ"	"-0.06964866	"0.0013781453"	"0.0271028"	"-0.132644150"	"7.750401389"	"0.0701762728361747"
"ENSG00000164924.13"	"L3MBTL3"	"-0.04041195	"0.0011017680"	"0.2356890"	"-0.077786689"	"1.925155548"	"0.156197503674218"
"ENSG00000119522.11"	"TBX6"	"NA"	"NA"	"NA"	"0.121238496"	"2.143572793"	"0.0662526904737407"
"ENSG00000124006.10"	"GPN3"	"NA"	"NA"	"NA"	"-0.364177993"	"6.403430244"	"0.027624136071762"
"ENSG00000198945.3"	"PMS2P9"	"0.020375975"	"0.0015644455"	"0.2301817"	"0.021376691"	"8.122341214"	"0.230292281651083"
"ENSG00000149922.6"	"ATXN3"	"0.034814664"	"0.0080333447"	"0.0555229"	"0.030654231"	"0.000101379"	"0.150466779859239"
"ENSG00000196993.4"	"STX17"	"NA"	"NA"	"NA"	"-0.054244399"	"0.000146112"	"0.0668370650973813"
"ENSG00000111231.4"	"STK33"	"-0.00455941	"0.9975640828"	"0.0129048"	"-0.152731136"	"0.000200472"	"0.0191027934869054"
"ENSG00000233448.2"	"LMBR1L"	"-0.02997265	"0.0046440008"	"0.2975480"	"-0.025389734"	"0.000200880"	"0.249208469181555"
"ENSG00000066427.17"	"FTOP1"	"0.027001700"	"0.0073125598"	"0.2218645"	"0.032781421"	"0.000210743"	"0.096601170336098"
"ENSG00000136874.6"	"PSMC5"	"0.024632326"	"0.0037412800"	"0.1862931"	"0.032412295"	"0.000220500"	"0.219751328981754"
"ENSG00000132128.12"	"ENDOG"	"NA"	"NA"	"NA"	"-0.095471928"	"0.000220793"	"0.017164652765968"
"ENSG00000130413.11"	"NRBP1"	"0.006870175"	"0.396524933"	"0.0464242"	"0.035491929"	"0.000221752"	"0.0835581383320405"
"ENSG00000139636.11"	"POM121B"	"-7.47231081	"0.795608700"	"0.1032372"	"-0.062765306"	"0.000255959"	"0.0891533260743145"
"ENSG00000226491.1"	"CDK5RAP3"	"-0.03120080	"0.0011247129"	"0.0860132"	"-0.084851783"	"0.000262826"	"0.0248069698529667"
"ENSG00000087191.8"	"RALGPS2"	"-0.03024915	"0.0012890682"	"0.1792976"	"-0.060229364"	"0.000280881"	"0.0718524498808238"
"ENSG00000167136.6"	"NT5DC2"	"0.026634805"	"0.0028597554"	"0.2891293"	"0.041146477"	"0.000412864"	"0.114576800820889"
"ENSG00000248280.1"	"CCDC116"	"NA"	"NA"	"NA"	"0.034749841"	"0.000539350"	"0.104567425172387"
"ENSG00000115216.9"	"SKIV2L"	"-0.02610944	"0.0039079433"	"0.1383096"	"-0.027606492"	"0.000543849"	"0.0555876433275509"
"ENSG00000205578.4"	"TM6SF2"	"-0.03751687	"0.0163052376"	"0.0299837"	"-0.052160217"	"0.000557179"	"0.120772019594373"
"ENSG00000108465.10"	"PDZK1IP1"	"-0.02482160	"0.0016125221"	"0.1528281"	"-0.021486211"	"0.000573377"	"0.141369693517862"
"ENSG00000253704.1"	"RAPSN"	"NA"	"NA"	"NA"	"-0.052860853"	"0.000587167"	"0.0662185309165735"
"ENSG00000116191.13"	"DENND1A"	"0.005806912"	"0.405995693"	"0.2721506"	"-0.026253164"	"0.000646196"	"0.0994502315987171"
"ENSG00000175938.6"	"OBSL1"	"NA"	"NA"	"NA"	"0.052961276"	"0.000667273"	"0.0206566512586812"

"ENSG00000236829.5"	"NPIPB9"	"NA"	"NA"	"NA"	"-0.045505930	"0.00067339(	"0.0688891669109507"
"ENSG00000168268.6"	"LRRC41"	"-0.01416939	"0.3217568481	"0.0493802	"-0.043566517	"0.000680079	"0.0463561722844343"
"ENSG00000161180.6"	"RP11-33B1.2"	"-0.01497345	"0.1386177154	"0.0651708	"-0.037197910	"0.000704115	"0.130713992950893"
"ENSG00000204351.7"	"RP11-267M23.4"	"-0.01433778	"0.0040502418	"0.3936300	"-0.023800142	"0.000753272	"0.211233102423472"
"ENSG00000213996.8"	"ORAI3"	"-0.06899024	"0.0012352124	"0.0162676	"-0.066469208	"0.000820555	"0.0496312576975815"
"ENSG00000162366.3"	"Z97634.3"	"-0.03475022	"0.0060389901	"0.0586533	"-0.078206392	"0.000842102	"0.0175431727240219"
