# Supplementary material for: The transcriptome-wide association search for genes and genetic variants which associate with BMI and gestational weight gain in women with type 1 diabetes
Source: Mol Med. 2021 Jan 20;27:6. doi: 10.1186/s10020-020-00266-z (PMC7818927; doi:10.1186/s10020-020-00266-z)
Supplement: Supplementary file 4 — Additional file 4: Table S3a. The results of PrediXcan on Giant cohort on BMI only. b. The results of PrediXcan on Giant cohort on BMI and GWG. c. The results of PrediXcan on Giant cohort on GWG only. [file 10020_2020_266_MOESM4_ESM.zip › Table S3a.pdf]

1,"ENSG00000244405"
2,"ENSG00000138031"
3,"ENSG00000178952"
4,"ENSG00000138092"
5,"ENSG00000172260"
6,"ENSG00000178188"
7,"ENSG00000177200"
8,"ENSG00000168488"
9,"ENSG00000198156"
10,"ENSG00000176953"
11,"ENSG00000205213"
12,"ENSG00000188322"
13,"ENSG00000233232"
14,"ENSG00000109919"
15,"ENSG00000197165"
16,"ENSG00000172247"
17,"ENSG00000134343"
18,"ENSG00000176046"
19,"ENSG00000115137"
20,"ENSG00000205609"
21,"ENSG00000197272"
22,"ENSG00000188779"
23,"ENSG00000196502"
24,"ENSG00000213057"
25,"ENSG00000165917"
26,"ENSG00000134571"
27,"ENSG00000196296"
28,"ENSG00000109920"
29,"ENSG00000213658"
30,"ENSG00000184110"
31,"ENSG00000161791"
32,"ENSG00000030066"
33,"ENSG00000130202"
34,"ENSG00000213619"
35,"ENSG00000023330"
36,"ENSG00000101751"
37,"ENSG00000197728"
38,"ENSG00000167985"
39,"ENSG00000104112"
40,"ENSG00000065029"
41,"ENSG00000131409"
42,"ENSG00000112981"
43,"ENSG00000049323"
44,"ENSG00000174915"
45,"ENSG00000164078"
46,"ENSG00000204264"
47,"ENSG00000011485"
48,"ENSG00000197445"

49,"ENSG00000141294"
50,"ENSG00000179909"
51,"ENSG00000152784"
52,"ENSG00000139620"
53,"ENSG00000164574"
54,"ENSG00000131781"
55,"ENSG00000166762"
56,"ENSG00000197980"
57,"ENSG00000163320"
58,"ENSG00000176124"
59,"ENSG00000177595"
60,"ENSG00000103496"
61,"ENSG00000204852"
62,"ENSG00000133818"
63,"ENSG00000145730"
64,"ENSG00000151640"
65,"ENSG00000178222"
66,"ENSG00000241973"
67,"ENSG00000172728"
68,"ENSG00000122008"
69,"ENSG00000145014"
70,"ENSG00000188559"
71,"ENSG00000047188"
72,"ENSG00000118197"
73,"ENSG00000019505"
74,"ENSG00000182319"
75,"ENSG00000106686"
76,"ENSG00000104142"
77,"ENSG00000119401"
78,"ENSG00000110046"
79,"ENSG00000137309"
80,"ENSG00000107566"
81,"ENSG00000176155"
82,"ENSG00000178971"
83,"ENSG00000154269"
84,"ENSG00000219626"
85,"ENSG00000100997"
86,"ENSG00000110092"
87,"ENSG00000156253"
88,"ENSG00000205174"
89,"ENSG00000037474"
90,"ENSG00000133703"
91,"ENSG00000163131"
92,"ENSG00000129673"
93,"ENSG00000136874"
94,"ENSG00000102879"
95,"ENSG00000123352"
96,"ENSG00000187257"

97,"ENSG00000067836"
98,"ENSG00000127399"
99,"ENSG00000126522"
100,"ENSG00000063761"
101,"ENSG00000169689"
102,"ENSG00000077380"
103,"ENSG00000137462"
104,"ENSG00000037637"
105,"ENSG00000100197"
106,"ENSG00000102967"
107,"ENSG00000146477"
108,"ENSG00000133069"
109,"ENSG00000178297"
110,"ENSG00000254093"
111,"ENSG00000114790"
112,"ENSG00000140104"
113,"ENSG00000204442"
114,"ENSG00000157240"
115,"ENSG00000159199"
116,"ENSG00000123179"
117,"ENSG00000113273"
118,"ENSG00000000971"
119,"ENSG00000180891"
120,"ENSG00000181444"
121,"ENSG00000186184"
122,"ENSG00000176428"
123,"ENSG00000128563"
124,"ENSG00000169031"
125,"ENSG00000137707"
126,"ENSG00000084676"
127,"ENSG00000135454"
128,"ENSG00000089220"
129,"ENSG00000100591"
130,"ENSG00000137709"
131,"ENSG00000082258"
132,"ENSG00000167395"
133,"ENSG00000163069"
134,"ENSG00000188976"
135,"ENSG00000171695"
136,"ENSG00000138172"
137,"ENSG00000185022"
138,"ENSG00000197935"
139,"ENSG00000186806"
140,"ENSG00000115944"
141,"ENSG00000182771"
142,"ENSG00000115561"
143,"ENSG00000124067"
144,"ENSG00000150938"

145,"ENSG00000163141"
146,"ENSG00000261701"
147,"ENSG00000183684"
148,"ENSG00000159210"
149,"ENSG00000159433"
150,"ENSG00000122696"
151,"ENSG00000180979"
152,"ENSG00000178568"
153,"ENSG00000183114"
154,"ENSG00000155903"
155,"ENSG00000138111"
156,"ENSG00000094916"
157,"ENSG00000144199"
158,"ENSG00000176723"
159,"ENSG00000115234"
160,"ENSG00000108469"
161,"ENSG00000172543"
162,"ENSG00000111670"
163,"ENSG00000134871"
164,"ENSG00000198467"
165,"ENSG00000138231"
166,"ENSG00000172548"
167,"ENSG00000126870"
168,"ENSG00000164167"
169,"ENSG00000146555"
170,"ENSG00000135250"
171,"ENSG00000168813"
172,"ENSG00000116151"
173,"ENSG00000151806"
174,"ENSG00000165060"
175,"ENSG00000128699"
176,"ENSG00000075131"
177,"ENSG00000156398"
178,"ENSG00000033627"
179,"ENSG00000152944"
180,"ENSG00000118705"
181,"ENSG00000152457"
182,"ENSG00000101337"
183,"ENSG00000213246"
184,"ENSG00000110768"
185,"ENSG00000159723"
186,"ENSG00000127463"
187,"ENSG00000172922"
188,"ENSG00000164307"
189,"ENSG00000128203"
190,"ENSG00000185052"
191,"ENSG00000183597"
192,"ENSG00000012061"

193,"ENSG00000198270"
194,"ENSG00000125898"
195,"ENSG00000112365"
196,"ENSG00000129204"
197,"ENSG00000171202"
198,"ENSG00000174564"
199,"ENSG00000105393"
200,"ENSG00000186205"
201,"ENSG00000237541"
202,"ENSG00000189091"
203,"ENSG00000117480"
204,"ENSG00000129933"
205,"ENSG00000213398"
206,"ENSG00000204536"
207,"ENSG00000113555"
208,"ENSG00000145723"
209,"ENSG00000160221"
210,"ENSG00000127995"
211,"ENSG00000186399"
212,"ENSG00000162687"
213,"ENSG00000183570"
214,"ENSG00000085491"
215,"ENSG00000131931"
216,"ENSG00000176623"
217,"ENSG00000124207"
218,"ENSG00000100603"
219,"ENSG00000072858"
220,"ENSG00000095370"
221,"ENSG00000215305"
222,"ENSG00000094975"
223,"ENSG00000137815"
224,"ENSG00000135127"
225,"ENSG00000198805"
226,"ENSG00000198089"
227,"ENSG00000137857"
228,"ENSG00000168016"
229,"ENSG00000155755"
230,"ENSG00000103042"
231,"ENSG00000139597"
232,"ENSG00000063176"
233,"ENSG00000185245"
234,"ENSG00000115138"
235,"ENSG00000159917"
236,"ENSG00000228716"
237,"ENSG00000108465"
238,"ENSG00000099338"
239,"ENSG00000085644"
240,"ENSG00000187097"

241,"ENSG00000122390"
242,"ENSG00000161328"
243,"ENSG00000121542"
244,"ENSG00000109956"
245,"ENSG00000137216"
246,"ENSG00000198040"
247,"ENSG00000138131"
248,"ENSG00000204967"
249,"SPPL2B"
250,"ENSG00000232774"
251,"ENSG00000105556"
252,"ENSG00000103375"
253,"ENSG00000173209"
254,"ENSG00000103489"
255,"ENSG00000169203"
256,"ENSG00000184232"
257,"ENSG00000160685"
258,"ENSG00000154710"
259,"ENSG00000146205"
260,"ENSG00000198796"
261,"ENSG00000162623"
262,"ENSG00000198300"
263,"ENSG00000158865"
264,"ENSG00000206344"
265,"ENSG00000176402"
266,"ENSG00000008838"
267,"ENSG00000182628"
268,"ENSG00000153207"
269,"ENSG00000134058"
270,"ENSG00000139055"
271,"ENSG00000141858"
272,"ENSG00000119328"
273,"ENSG00000186470"
274,"ENSG00000099246"
275,"ENSG00000155858"
276,"ENSG00000102595"
277,"ENSG00000162461"
278,"ENSG00000181754"
279,"ENSG00000198959"
280,"ENSG00000138744"
281,"ENSG00000135976"
282,"ENSG00000125633"
283,"ENSG00000010282"
284,"ENSG00000078674"
285,"ENSG00000176386"
286,"ENSG00000164122"
287,"ENSG00000174891"
288,"ENSG00000139572"

289,"ENSG00000167394"
290,"ENSG00000137804"
291,"ENSG00000104412"
292,"ENSG00000181873"
293,"ENSG00000137145"
294,"ENSG00000130477"
295,"ENSG00000132677"
296,"ENSG00000148498"
297,"ENSG00000167637"
298,"ENSG00000149262"
299,"ENSG00000168056"
300,"ENSG00000124201"
301,"ENSG00000162174"
302,"ENSG00000169903"
303,"ENSG00000188385"
304,"ENSG00000039068"
305,"ENSG00000166145"
306,"ENSG00000110975"
307,"ENSG00000085831"
308,"ENSG00000234745"
309,"ENSG00000139405"
310,"ENSG00000213024"
311,"ENSG00000119599"
312,"ENSG00000129355"
313,"ENSG00000105928"
314,"ENSG00000131437"
315,"ENSG00000243646"
316,"ENSG00000226742"
317,"ENSG00000189120"
318,"ENSG00000089250"
319,"ENSG00000169919"
320,"ENSG00000164124"
321,"ENSG00000084764"
322,"ENSG00000155393"
323,"ENSG00000135148"
324,"ENSG00000100764"
325,"ENSG00000019995"
326,"ENSG00000163138"
327,"ENSG00000083828"
328,"ENSG00000108556"
329,"ENSG00000198130"
330,"ENSG00000005486"
331,"ENSG00000141101"
332,"ENSG00000167483"
333,"ENSG00000177697"
334,"ENSG00000168032"
335,"ENSG00000140105"
336,"ENSG00000156831"

337,"ENSG00000254122"
338,"ENSG00000115761"
339,"ENSG00000087086"
340,"ENSG00000164603"
341,"ENSG00000134697"
342,"ENSG00000175322"
343,"ENSG00000031691"
344,"ENSG00000168300"
345,"ENSG00000198964"
346,"ENSG00000165799"
347,"ENSG00000185272"
348,"ENSG00000114395"
349,"ENSG00000124257"
350,"ENSG00000063660"
351,"ENSG00000171444"
352,"ENSG00000104299"
353,"ENSG00000169684"
354,"ENSG00000135547"
355,"ENSG00000157303"
356,"ENSG00000147724"
357,"ENSG00000185551"
358,"ENSG00000169299"
359,"ENSG00000091640"
360,"ENSG00000180758"
361,"ENSG00000100106"
362,"ENSG00000185627"
363,"ENSG00000221890"
364,"ENSG00000123545"
365,"ENSG00000182568"
366,"ENSG00000171962"
367,"ENSG00000006282"
368,"ENSG00000214517"
369,"ENSG00000141140"
370,"ENSG00000025772"
371,"ENSG00000115947"
372,"ENSG00000108823"
373,"ENSG00000257017"
374,"ENSG00000172159"
375,"ENSG00000103018"
376,"ENSG00000141452"
377,"ENSG00000133641"
378,"ENSG00000091536"
379,"ENSG00000165113"
380,"ENSG00000110514"
381,"ENSG00000153006"
382,"ENSG00000188554"
383,"ENSG00000115677"
384,"ENSG00000166263"

385,"ENSG00000171469"
386,"ENSG00000137996"
387,"ENSG00000149531"
388,"ENSG00000141854"
389,"ENSG00000149212"
390,"ENSG00000122674"
391,"ENSG00000187741"
392,"ENSG00000005513"
393,"ENSG00000138286"
394,"ENSG00000120675"
395,"ENSG00000143436"
396,"ENSG00000166949"
397,"ENSG00000142892"
398,"ENSG00000156103"
399,"ENSG00000152475"
400,"ENSG00000107164"
401,"ENSG00000205078"
402,"ENSG00000138430"
403,"ENSG00000143882"
404,"ENSG00000186318"
405,"ENSG00000169436"
406,"ENSG00000103479"
407,"ENSG00000177374"
408,"ENSG00000114735"
409,"ENSG00000164338"
410,"ENSG00000213015"
411,"ENSG00000101310"
412,"ENSG00000214530"
413,"ENSG00000196648"
414,"ENSG00000133961"
415,"ENSG00000135390"
416,"ENSG00000109572"
417,"ENSG00000185043"
418,"ENSG00000177700"
419,"ENSG00000131737"
420,"ENSG00000138768"
421,"ENSG00000041357"
422,"ENSG00000037042"
423,"ENSG00000063854"
424,"ENSG00000205464"
425,"ENSG00000101596"
426,"ENSG00000149292"
427,"ENSG00000197958"
428,"ENSG00000136827"
429,"ENSG00000140464"
430,"ENSG00000170091"
431,"ENSG00000169871"
432,"ENSG00000182240"

433,"ENSG00000196456"
434,"ENSG00000119686"
435,"ENSG00000197345"
436,"ENSG00000012048"
437,"ENSG00000100053"
438,"ENSG00000171914"
439,"ENSG00000119973"
440,"ENSG00000141562"
441,"ENSG00000109576"
442,"ENSG00000204287"
443,"ENSG00000157557"
444,"ENSG00000068028"
445,"ENSG00000154767"
446,"ENSG00000178078"
447,"ENSG00000099331"
448,"ENSG00000126602"
449,"ENSG00000159202"
450,"ENSG00000171763"
451,"ENSG00000136052"
452,"ENSG00000054654"
453,"ENSG00000149634"
454,"ENSG00000189129"
455,"ENSG00000153246"
456,"ENSG00000148429"
457,"ENSG00000134020"
458,"ENSG00000186652"
459,"ENSG00000148600"
460,"ENSG00000140995"
461,"ENSG00000165948"
462,"ENSG00000186364"
463,"ENSG00000186281"
464,"ENSG00000169660"
465,"ENSG00000103257"
466,"ENSG00000157837"
467,"ENSG00000176678"
468,"ENSG00000180801"
469,"ENSG00000181381"
470,"ENSG00000166573"
471,"ENSG00000099956"
472,"ENSG00000196418"
473,"ENSG00000114698"
474,"ENSG00000173473"
475,"ENSG00000189180"
476,"ENSG00000111676"
477,"ENSG00000156475"
478,"ENSG00000163349"
479,"ENSG00000170367"
480,"ENSG00000170430"

481,"ENSG00000170653"
482,"ENSG00000167261"
483,"ENSG00000257949"
484,"ENSG00000156140"
485,"ENSG00000104897"
486,"ENSG00000203772"
487,"ENSG00000155034"
488,"ENSG00000160233"
489,"ENSG00000213676"
490,"ENSG00000188011"
491,"ENSG00000198890"
492,"ENSG00000109072"
493,"ENSG00000155975"
494,"ENSG00000175470"
495,"ENSG00000134077"
496,"ENSG00000166582"
497,"ENSG00000186204"
498,"ENSG00000054690"
499,"ENSG00000179456"
500,"ENSG00000141750"
501,"ENSG00000214063"
502,"ENSG00000029725"
503,"ENSG00000149273"
504,"ENSG00000072958"
505,"ENSG00000188573"
506,"ENSG00000149328"
507,"ENSG00000139304"
508,"ENSG00000076242"
509,"ENSG00000105501"
510,"ENSG00000169926"
511,"ENSG00000116741"
512,"ENSG00000110435"
513,"ENSG00000135974"
514,"ENSG00000145781"
515,"ENSG00000028528"
516,"ENSG00000143442"
517,"ENSG00000134115"
518,"ENSG00000172349"
519,"ENSG00000197548"
520,"ENSG00000268171"
521,"ENSG00000179119"
522,"ENSG00000107854"
523,"ENSG00000135899"
524,"ENSG00000100505"
525,"ENSG00000213445"
526,"ENSG00000203791"
527,"ENSG00000259207"
528,"ENSG00000204657"

529,"ENSG00000056487"
530,"ENSG00000101307"
531,"ENSG00000185436"
532,"ENSG00000134184"
533,"ENSG00000188051"
534,"ENSG00000166796"
535,"ENSG00000204616"
536,"ENSG00000144224"
537,"ENSG00000137221"
538,"ENSG00000163704"
539,"ENSG00000143977"
540,"ENSG00000100014"
541,"ENSG00000198554"
542,"ENSG00000147416"
543,"ENSG00000187922"
544,"ENSG00000101000"
545,"ENSG00000173566"
546,"ENSG00000143162"
547,"ENSG00000099326"
548,"ENSG00000137747"
549,"ENSG00000164346"
550,"ENSG00000165233"
551,"WDR45L"
552,"ENSG00000138785"
553,"ENSG00000187758"
554,"ENSG00000182196"
555,"ENSG00000168234"
556,"ENSG00000145936"
557,"ENSG00000085514"
558,"ENSG00000106868"
559,"ENSG00000128918"
560,"ENSG00000170381"
561,"ENSG00000167780"
562,"ENSG00000168288"
563,"ENSG00000106976"
564,"ENSG00000198844"
565,"ENSG00000139737"
566,"ENSG00000006747"
567,"ENSG00000138386"
568,"ENSG00000135828"
569,"ENSG00000155592"
570,"ENSG00000198952"
571,"ENSG00000198954"
572,"ENSG00000110801"
573,"ENSG00000172938"
574,"ENSG00000244607"
575,"ENSG00000185149"
576,"ENSG00000064313"

577,"ENSG00000172171"
578,"ENSG00000204149"
579,"ENSG00000182150"
580,"ENSG00000167670"
581,"ENSG00000154380"
582,"ENSG00000136810"
583,"ENSG00000106799"
584,"ENSG00000105617"
585,"ENSG00000174606"
586,"ENSG00000099817"
587,"ENSG00000113721"
588,"ENSG00000048471"
589,"ENSG00000253719"
590,"ENSG00000196620"
591,"ENSG00000166426"
592,"ENSG00000172493"
593,"ENSG00000112212"
594,"ENSG00000152049"
595,"ENSG00000222040"
596,"ENSG00000164053"
597,"ENSG00000241484"
598,"ENSG00000183688"
599,"ENSG00000165322"
600,"ENSG00000129219"
601,"ENSG00000168566"
602,"ENSG00000148344"
603,"ENSG00000105357"
604,"ENSG00000068784"
605,"ENSG00000240563"
606,"ENSG00000086666"
607,"ENSG00000235978"
608,"ENSG00000133056"
609,"ENSG00000143149"
610,"ENSG00000198502"
611,"ENSG00000171840"
612,"ENSG00000162368"
613,"ENSG00000043462"
614,"ENSG00000165637"
615,"ENSG00000183431"
616,"ENSG00000134909"
617,"ENSG00000167978"
618,"ENSG00000103534"
619,"ENSG00000180817"
620,"ENSG00000151790"
621,"ENSG00000103024"
622,"ENSG00000198015"
623,"ENSG00000189366"
624,"ENSG00000237172"

625,"ENSG00000213347"
626,"ENSG00000204305"
627,"ENSG00000147862"
628,"ENSG00000158987"
629,"ENSG00000169981"
630,"ENSG00000221955"
631,"ENSG00000140939"
632,"ENSG00000079785"
633,"ENSG00000144339"
634,"ENSG00000196503"
635,"ENSG00000144118"
636,"ENSG00000007168"
637,"ENSG00000155111"
638,"ENSG00000214753"
639,"ENSG00000228146"
640,"ENSG00000136881"
641,"ENSG00000183230"
642,"ENSG00000227345"
643,"ENSG00000087884"
644,"ENSG00000095739"
645,"ENSG00000196091"
646,"ENSG00000131748"
647,"ENSG00000145416"
648,"ENSG00000102871"
649,"ENSG00000089639"
650,"ENSG00000092203"
651,"ENSG00000124614"
652,"ENSG00000117525"
653,"ENSG00000146147"
654,"ENSG00000159231"
655,"ENSG00000099795"
656,"ENSG00000109472"
657,"ENSG00000160588"
658,"ENSG00000116874"
659,"ENSG00000112305"
660,"ENSG00000103342"
661,"ENSG00000179981"
662,"ENSG00000117748"
663,"ENSG00000204169"
664,"ENSG00000163492"
665,"ENSG00000204540"
666,"ENSG00000171596"
667,"ENSG00000137843"
668,"ENSG00000141252"
669,"ENSG00000049192"
670,"ENSG00000138375"
671,"ENSG00000119938"
672,"ENSG00000144320"

673,"ENSG00000186230"
674,"ENSG00000136819"
675,"ENSG00000144455"
676,"ENSG00000141905"
677,"ENSG00000231389"
678,"ENSG00000170779"
679,"ENSG00000204538"
680,"ENSG00000137871"
681,"ENSG00000041515"
682,"ENSG00000175110"
683,"ENSG00000135736"
684,"ENSG00000111652"
685,"ENSG00000152642"
686,"ENSG00000171051"
687,"ENSG00000116237"
688,"ENSG00000077616"
689,"ENSG00000114902"
690,"ENSG00000256269"
691,"ENSG00000169418"
692,"ENSG00000188191"
693,"ENSG00000176597"
694,"ENSG00000099385"
695,"ENSG00000187105"
696,"ENSG00000154415"
697,"ENSG00000134852"
698,"ENSG00000142632"
699,"ENSG00000160014"
700,"ENSG00000147894"
701,"ENSG00000153094"
702,"ENSG00000159212"
703,"ENSG00000104093"
704,"ENSG00000181016"
705,"ENSG00000105699"
706,"ENSG00000168000"
707,"ENSG00000138030"
708,"ENSG00000157833"
709,"ENSG00000104369"
710,"ENSG00000148300"
711,"ENSG00000163029"
712,"ENSG00000095203"
713,"ENSG00000138207"
714,"ENSG00000204869"
715,"ENSG00000133318"
716,"ENSG00000153015"
717,"ENSG00000111335"
718,"ENSG00000088756"
719,"ENSG00000187764"
720,"ENSG00000174371"

721,"ENSG00000121577"
722,"ENSG00000158714"
723,"ENSG00000142675"
724,"ENSG00000145244"
725,"ENSG00000149527"
726,"ENSG00000175029"
727,"ENSG00000095917"
728,"ENSG00000114547"
729,"ENSG00000243811"
730,"ENSG00000107223"
731,"ENSG00000171310"
732,"ENSG00000160741"
733,"ENSG00000172985"
734,"ENSG00000185187"
735,"ENSG00000171861"
736,"ENSG00000110169"
737,"ENSG00000249693"
738,"ENSG00000139567"
739,"ENSG00000135506"
740,"ENSG00000188486"
741,"ENSG00000188501"
742,"ENSG00000119965"
743,"ENSG00000053254"
744,"ENSG00000180185"
745,"ENSG00000157782"
746,"ENSG00000226479"
747,"ENSG00000176390"
748,"ENSG00000213240"
749,"ENSG00000155760"
750,"ENSG00000198925"
751,"ENSG00000090615"
752,"ENSG00000081019"
753,"ENSG00000204176"
754,"ENSG00000140650"
755,"ENSG00000150275"
756,"ENSG00000163281"
757,"ENSG00000107104"
758,"ENSG00000141391"
759,"ENSG00000181396"
760,"ENSG00000136240"
761,"ENSG00000152582"
762,"ENSG00000138735"
763,"ENSG00000089101"
764,"ENSG00000131845"
765,"ENSG00000136108"
766,"ENSG00000103723"
767,"ENSG00000066322"
768,"ENSG00000161992"

769,"ENSG00000053328"
770,"ENSG00000177465"
771,"ENSG00000170027"
772,"ENSG00000102781"
773,"ENSG00000074755"
774,"ENSG00000254827"
775,"ENSG00000128285"
776,"ENSG00000049245"
777,"ENSG00000101745"
778,"ENSG00000184999"
779,"ENSG00000182749"
780,"ENSG00000198780"
781,"ENSG00000160949"
782,"ENSG00000128683"
783,"ENSG00000147853"
784,"ENSG00000130811"
785,"ENSG00000175166"
786,"ENSG00000258365"
787,"ENSG00000112902"
788,"ENSG00000086991"
789,"ENSG00000142233"
790,"ENSG00000115935"
791,"ENSG00000183340"
792,"ENSG00000139269"
793,"ENSG00000183255"
794,"ENSG00000142082"
795,"ENSG00000169439"
796,"ENSG00000103966"
797,"ENSG00000131355"
798,"ENSG00000145088"
799,"ENSG00000120370"
800,"ENSG00000171503"
801,"ENSG00000186453"
802,"ENSG00000188004"
803,"ENSG00000127084"
804,"ENSG00000146433"
805,"ENSG00000215252"
806,"ENSG00000196141"
807,"ENSG00000154485"
808,"ENSG00000173262"
809,"ENSG00000104760"
810,"ENSG00000203780"
811,"ENSG00000196365"
812,"ENSG00000214954"
813,"ENSG00000198520"
814,"ENSG00000146221"
815,"ENSG00000101695"
816,"ENSG00000144647"

817,"ENSG00000120314"
818,"ENSG00000152464"
819,"ENSG00000133612"
820,"ENSG00000186231"
821,"ENSG00000182993"
822,"ENSG00000105127"
823,"ENSG00000136271"
824,"ENSG00000161996"
825,"ENSG00000183250"
826,"ENSG00000151917"
827,"ENSG00000035720"
828,"ENSG00000115073"
829,"ENSG00000168754"
830,"ENSG00000143727"
831,"ENSG00000128973"
832,"ENSG00000165119"
833,"ENSG00000186529"
834,"ENSG00000198912"
835,"ENSG00000145907"
836,"ENSG00000028203"
837,"ENSG00000160271"
838,"ENSG00000144488"
839,"ENSG00000221947"
840,"ENSG00000140859"
841,"ENSG00000102547"
842,"ENSG00000187122"
843,"ENSG00000151748"
844,"ENSG00000196189"
845,"ENSG00000104936"
846,"ENSG00000169129"
847,"ENSG00000165238"
848,"ENSG00000158008"
849,"ENSG00000204174"
850,"ENSG00000134539"
851,"ENSG00000115368"
852,"ENSG00000145945"
853,"ENSG00000249087"
854,"ENSG00000100300"
855,"ENSG00000076258"
856,"ENSG00000107819"
857,"ENSG00000126460"
858,"ENSG00000008735"
859,"ENSG00000137473"
860,"ENSG00000138326"
861,"ENSG00000011275"
862,"ENSG00000062524"
863,"ENSG00000104881"
864,"ENSG00000187815"

865,"ENSG00000104825"
866,"ENSG00000139343"
867,"ENSG00000127955"
868,"ENSG00000141349"
869,"ENSG00000135631"
870,"ENSG00000179941"
871,"ENSG00000165410"
872,"ENSG00000118503"
873,"ENSG00000100490"
874,"ENSG00000135686"
875,"ENSG00000134490"
876,"ENSG00000102543"
877,"ENSG00000091262"
878,"ENSG00000189403"
879,"ENSG00000118137"
880,"ENSG00000166946"
881,"ENSG00000185158"
882,"ENSG00000118997"
883,"ENSG00000196743"
884,"ENSG00000007341"
885,"ENSG00000136250"
886,"ENSG00000141959"
887,"ENSG00000203666"
888,"ENSG00000162040"
889,"ENSG00000112031"
890,"ENSG00000170264"
891,"ENSG00000156052"
892,"ENSG00000178789"
893,"ENSG00000106367"
894,"ENSG00000181333"
895,"ENSG00000138750"
896,"ENSG00000001460"
897,"ENSG00000214872"
898,"ENSG00000124613"
899,"ENSG00000147905"
900,"ENSG00000143942"
901,"ENSG00000140830"
902,"ENSG00000145476"
903,"ENSG00000184434"
904,"ENSG00000215021"
905,"ENSG00000181409"
906,"ENSG00000197415"
907,"ENSG00000084710"
908,"ENSG00000176532"
909,"ENSG00000108848"
910,"ENSG00000120820"
911,"ENSG00000172725"
912,"ENSG00000121690"

913,"ENSG00000072682"
914,"ZNF252"
915,"ENSG00000101247"
916,"ENSG00000243317"
917,"ENSG00000165271"
918,"ENSG00000125900"
919,"ENSG00000142188"
920,"ENSG00000137509"
921,"ENSG00000111790"
922,"ENSG00000165916"
923,"ENSG00000114503"
924,"ENSG00000165269"
925,"ENSG00000112701"
926,"ENSG00000152556"
927,"ENSG00000073111"
928,"ENSG00000140990"
929,"ENSG00000142459"
930,"ENSG00000164125"
931,"ENSG00000162520"
932,"ENSG00000001631"
933,"ENSG00000067798"
934,"ENSG00000158806"
935,"ENSG00000127481"
936,"ENSG00000146535"
937,"ENSG00000182512"
938,"ENSG00000151502"
939,"ENSG00000138801"
940,"ENSG00000173267"
941,"ENSG00000106290"
942,"ENSG00000089723"
943,"ENSG00000112164"
944,"ENSG00000075856"
945,"ENSG00000130943"
946,"ENSG00000138767"
947,"ENSG00000140386"
948,"ENSG00000188611"
949,"ENSG00000003436"
950,"ENSG00000100228"
951,"ENSG00000197020"
952,"ENSG00000181938"
953,"ENSG00000187609"
954,"ENSG00000167280"
955,"ENSG00000151240"
956,"ENSG00000105258"
957,"ENSG00000006607"
958,"ENSG00000204231"
959,"ENSG00000214700"
960,"ENSG00000214140"

961,"ENSG00000076248"
962,"ENSG00000154678"
963,"ENSG00000166135"
964,"ENSG00000213214"
965,"ENSG00000113269"
966,"ENSG00000170035"
967,"ENSG00000154240"
968,"ENSG00000108591"
969,"ENSG00000105251"
970,"ENSG00000112159"
971,"ENSG00000126217"
972,"ENSG00000105383"
973,"ENSG00000185669"
974,"ENSG00000124098"
975,"ENSG00000170619"
976,"ENSG00000066230"
977,"ENSG00000101282"
978,"ENSG00000111358"
979,"ENSG00000166926"
980,"ENSG00000158023"
981,"ENSG00000142856"
982,"ENSG00000124116"
983,"ENSG00000180376"
984,"ENSG00000156787"
985,"ENSG00000143622"
986,"ENSG00000204947"
987,"ENSG00000172175"
988,"ENSG00000014824"
989,"ENSG00000148459"
990,"ENSG00000073910"
991,"ENSG00000130508"
992,"ENSG00000179165"
993,"ENSG00000188690"
994,"ENSG00000132394"
995,"ENSG00000189171"
996,"ENSG00000043514"
997,"ENSG00000174527"
998,"ENSG00000183386"
999,"ENSG00000149231"
1000,"ENSG00000153157"
1001,"ENSG00000228157"
1002,"ENSG00000185414"
1003,"ENSG00000131558"
1004,"ENSG00000221946"
1005,"ENSG00000147689"
1006,"ENSG00000160392"
1007,"ENSG00000118804"
1008,"ENSG00000158825"

1009,"ENSG00000196352"
1010,"ENSG00000145569"
1011,"ENSG00000215845"
1012,"ENSG00000108774"
1013,"ENSG00000203546"
1014,"ENSG00000180611"
1015,"ENSG00000137135"
1016,"ENSG00000187535"
1017,"ENSG00000099290"
1018,"ENSG00000134247"
1019,"ENSG00000141738"
1020,"ENSG00000107201"
1021,"ENSG00000130313"
1022,"ENSG00000181449"
1023,"ENSG00000125484"
1024,"ENSG00000168792"
1025,"ENSG00000149679"
1026,"ENSG00000106617"
1027,"ENSG00000116212"
1028,"ENSG00000048405"
1029,"ENSG00000005961"
1030,"ENSG00000136040"
1031,"ENSG00000119844"
1032,"ENSG00000198060"
1033,"ENSG00000167711"
1034,"ENSG00000135404"
1035,"ENSG00000105509"
1036,"ENSG00000099960"
1037,"ENSG00000141258"
1038,"ENSG00000167085"
1039,"ENSG00000197775"
1040,"ENSG00000132912"
1041,"ENSG00000175390"
1042,"ENSG00000059122"
1043,"ENSG00000066735"
1044,"ENSG00000154511"
1045,"ENSG00000164972"
1046,"ENSG00000130175"
1047,"ENSG00000067208"
1048,"ENSG00000196678"
1049,"ENSG00000048540"
1050,"ENSG00000170153"
1051,"ENSG00000168528"
1052,"ENSG00000146223"
1053,"ENSG00000204219"
1054,"ENSG00000164303"
1055,"ENSG00000167920"
1056,"ENSG00000079999"

1057,"ENSG00000112667"
1058,"ENSG00000138101"
1059,"ENSG00000127990"
1060,"ENSG00000130518"
1061,"ENSG00000142973"
1062,"ENSG00000148384"
1063,"ENSG00000196683"
1064,"ENSG00000181315"
1065,"ENSG00000189409"
1066,"ENSG00000187642"
1067,"ENSG00000205863"
1068,"ENSG00000126226"
1069,"ENSG00000198336"
1070,"ENSG00000113638"
1071,"ENSG00000140043"
1072,"ENSG00000150995"
1073,"ENSG00000085788"
1074,"ENSG00000141568"
1075,"ENSG00000096006"
1076,"ENSG00000141433"
1077,"ENSG00000083067"
1078,"ENSG00000244187"
1079,"ENSG00000178980"
1080,"ENSG00000125734"
1081,"ENSG00000142623"
1082,"ENSG00000112486"
1083,"ENSG00000166483"
1084,"ENSG00000228696"
1085,"ENSG00000164171"
1086,"ENSG00000120805"
1087,"ENSG00000132906"
1088,"ENSG00000124783"
1089,"ENSG00000189007"
1090,"ENSG00000159251"
1091,"ENSG00000214087"
1092,"ENSG00000204977"
1093,"ENSG00000110888"
1094,"ENSG00000102858"
1095,"ENSG00000166965"
1096,"ENSG00000165055"
1097,"ENSG00000108379"
1098,"ENSG00000105618"
1099,"ENSG00000073605"
1100,"ENSG00000168671"
1101,"ENSG00000088280"
1102,"ENSG00000025423"
1103,"ENSG00000164946"
1104,"ENSG00000112118"

1105,"ENSG00000100266"
1106,"ENSG00000162849"
1107,"ENSG00000087301"
1108,"ENSG00000183066"
1109,"ENSG00000241233"
1110,"ENSG00000164008"
1111,"ENSG00000198242"
1112,"ENSG00000198400"
1113,"ENSG00000161243"
1114,"ENSG00000198754"
1115,"ENSG00000176945"
1116,"ENSG00000198182"
1117,"ENSG00000243244"
1118,"ENSG00000121057"
1119,"ENSG00000100304"
1120,"ENSG00000119878"
1121,"ENSG00000144021"
1122,"ENSG00000116954"
1123,"ENSG00000196329"
1124,"ENSG00000159685"
1125,"ENSG00000109501"
1126,"ENSG00000127578"
1127,"ENSG00000132716"
1128,"ENSG00000130414"
1129,"ENSG00000174442"
1130,"ENSG00000169136"
1131,"ENSG00000049239"
1132,"ENSG00000168904"
1133,"ENSG00000146833"
1134,"ENSG00000163714"
1135,"ENSG00000151067"
1136,"ENSG00000148700"
1137,"ENSG00000163947"
1138,"ENSG00000135637"
1139,"ENSG00000065600"
1140,"ENSG00000015171"
1141,"ENSG00000132681"
1142,"ENSG00000143376"
1143,"ENSG00000115221"
1144,"ENSG00000255837"
1145,"ENSG00000099785"
1146,"ENSG00000090621"
1147,"ENSG00000171302"
1148,"ENSG00000187266"
1149,"ENSG00000226887"
1150,"C20orf20"
1151,"ENSG00000135953"
1152,"ENSG00000125409"

1153,"ENSG00000173960"
1154,"ENSG00000175106"
1155,"ENSG00000011451"
1156,"ENSG00000172530"
1157,"ENSG00000197226"
1158,"ENSG00000240764"
1159,"ENSG00000080815"
1160,"ENSG00000181264"
1161,"ENSG00000166183"
1162,"ENSG00000101204"
1163,"ENSG00000163378"
1164,"ENSG00000089022"
1165,"ENSG00000165702"
1166,"ENSG00000118514"
1167,"ENSG00000115204"
1168,"ENSG00000055211"
1169,"ENSG00000070404"
1170,"ENSG00000197451"
1171,"ENSG00000069966"
1172,"ENSG00000034713"
1173,"ENSG00000161533"
1174,"ENSG00000100033"
1175,"ENSG00000154917"
1176,"ENSG00000109182"
1177,"ENSG00000133027"
1178,"ENSG00000171160"
1179,"ENSG00000167657"
1180,"ENSG00000175697"
1181,"ENSG00000054983"
1182,"ENSG00000143106"
1183,"ENSG00000129625"
1184,"ENSG00000162881"
1185,"ENSG00000128383"
1186,"ENSG00000101181"
1187,"ENSG00000102743"
1188,"ENSG00000133657"
1189,"ENSG00000155542"
1190,"ENSG00000166928"
1191,"ENSG00000167840"
1192,"ENSG00000160305"
1193,"ENSG00000121879"
1194,"ENSG00000131979"
1195,"ENSG00000146910"
1196,"ENSG00000032389"
1197,"ENSG00000105281"
1198,"ENSG00000076555"
1199,"ENSG00000103811"
1200,"ENSG00000057704"

1201,"ENSG00000175518"
1202,"ENSG00000155438"
1203,"ENSG00000154277"
1204,"ENSG00000118600"
1205,"ENSG00000203697"
1206,"ENSG00000167965"
1207,"ENSG00000103202"
1208,"ENSG00000159110"
1209,"ENSG00000095321"
1210,"ENSG00000111801"
1211,"ENSG00000100376"
1212,"ENSG00000119383"
1213,"ENSG00000164707"
1214,"ENSG00000117500"
1215,"ENSG00000106484"
1216,"ENSG00000150636"
1217,"ENSG00000140067"
1218,"ENSG00000180245"
1219,"ENSG00000125863"
1220,"ENSG00000135596"
1221,"ENSG00000104695"
1222,"ENSG00000105676"
1223,"ENSG00000162873"
1224,"ENSG00000165650"
1225,"ENSG00000106069"
1226,"ENSG00000123892"
1227,"ENSG00000104361"
1228,"ENSG00000197168"
1229,"ENSG00000166352"
1230,"ENSG00000178996"
1231,"ENSG00000173947"
1232,"ENSG00000178343"
1233,"ENSG00000189068"
1234,"ENSG00000188100"
1235,"ENSG00000010626"
1236,"ENSG00000073060"
1237,"ENSG00000125630"
1238,"ENSG00000100373"
1239,"ENSG00000106462"
1240,"ENSG00000143157"
1241,"ENSG00000172893"
1242,"ENSG00000173915"
1243,"ENSG00000205038"
1244,"ENSG00000079435"
1245,"ENSG00000134001"
1246,"ENSG00000132467"
1247,"ENSG00000120160"
1248,"ENSG00000133119"

1249,"ENSG00000171566"
1250,"ENSG00000155130"
1251,"ENSG00000182272"
1252,"ENSG00000163536"
1253,"ENSG00000234444"
1254,"ENSG00000175928"
1255,"ENSG00000185271"
1256,"ENSG00000138400"
1257,"ENSG00000157741"
1258,"ENSG00000196126"
1259,"ENSG00000206432"
1260,"ENSG00000096088"
1261,"ENSG00000133104"
1262,"ENSG00000163083"
1263,"ENSG00000066455"
1264,"ENSG00000111450"
1265,"ENSG00000091039"
1266,"ENSG00000184903"
1267,"ENSG00000005001"
1268,"ENSG00000125551"
1269,"ENSG00000189058"
1270,"ENSG00000171135"
1271,"ENSG00000139998"
1272,"ENSG00000101440"
1273,"ENSG00000120697"
1274,"ENSG00000198108"
1275,"ENSG00000135452"
1276,"ENSG00000184454"
1277,"ENSG00000104059"
1278,"ENSG00000099974"
1279,"ENSG00000172936"
1280,"ENSG00000162645"
1281,"ENSG00000100243"
1282,"ENSG00000139641"
1283,"ENSG00000109016"
1284,"ENSG00000169851"
1285,"ENSG00000166225"
1286,"ENSG00000109255"
1287,"ENSG00000141580"
1288,"ENSG00000165512"
1289,"ENSG00000147789"
1290,"ENSG00000139178"
1291,"ENSG00000160951"
1292,"ENSG00000185344"
1293,"ENSG00000197555"
1294,"ENSG00000173226"
1295,"ENSG00000198075"
1296,"ENSG00000156958"

1297,"ENSG00000161057"
1298,"ENSG00000114771"
1299,"ENSG00000099860"
1300,"ENSG00000142065"
1301,"ENSG00000135100"
1302,"ENSG00000170819"
1303,"ENSG00000085982"
1304,"ENSG00000101346"
1305,"ENSG00000197256"
1306,"ENSG00000159082"
1307,"ENSG00000238083"
1308,"ENSG00000137875"
1309,"ENSG00000154945"
1310,"ENSG00000135747"
1311,"ENSG00000100348"
1312,"ENSG00000125843"
1313,"ENSG00000182931"
1314,"ENSG00000182253"
1315,"ENSG00000177000"
1316,"ENSG00000136011"
1317,"ENSG00000115252"
1318,"ENSG00000068383"
1319,"ENSG00000082805"
1320,"ENSG00000008128"
1321,"ENSG00000089050"
1322,"ENSG00000253710"
1323,"ENSG00000065427"
1324,"ENSG00000240230"
1325,"ENSG00000129657"
1326,"ENSG00000143368"
1327,"ENSG00000174453"
1328,"ENSG00000137760"
1329,"ENSG00000106571"
1330,"ENSG00000183850"
1331,"ENSG00000153406"
1332,"ENSG00000162888"
1333,"ENSG00000128272"
1334,"ENSG00000186998"
1335,"ENSG00000196247"
1336,"ENSG00000133661"
1337,"ENSG00000162636"
1338,"ENSG00000120322"
1339,"ENSG00000169598"
1340,"ENSG00000174007"
1341,"ENSG00000107897"
1342,"ENSG00000125170"
1343,"ENSG00000154803"
1344,"ENSG00000104888"

1345,"ENSG00000169016"
1346,"ENSG00000114316"
1347,"ENSG00000239713"
1348,"ENSG00000166451"
1349,"ENSG00000188992"
1350,"ENSG00000134317"
1351,"ENSG00000183605"
1352,"ENSG00000052749"
1353,"ENSG00000169612"
1354,"ENSG00000148826"
1355,"ENSG00000123395"
1356,"ENSG00000147576"
1357,"ENSG00000148396"
1358,"ENSG00000066739"
1359,"ENSG00000165633"
1360,"ENSG00000165730"
1361,"ENSG00000140254"
1362,"ENSG00000158485"
1363,"ENSG00000174177"
1364,"ENSG00000150527"
1365,"ENSG00000176903"
1366,"ENSG00000114124"
1367,"ENSG00000205281"
1368,"ENSG00000071282"
1369,"ENSG00000183426"
1370,"ENSG00000137574"
1371,"ENSG00000111671"
1372,"ENSG00000198835"
1373,"ENSG00000072864"
1374,"ENSG00000176986"
1375,"ENSG00000105197"
1376,"ENSG00000138443"
1377,"ENSG00000198826"
1378,"ENSG00000126243"
1379,"ENSG00000107521"
1380,"ENSG00000139174"
1381,"ENSG00000163807"
1382,"ENSG00000114416"
1383,"ENSG00000135916"
1384,"ENSG00000162620"
1385,"ENSG00000242372"
1386,"ENSG00000171148"
1387,"ENSG00000135773"
1388,"ENSG00000164080"
1389,"ENSG00000218537"
1390,"ENSG00000182957"
1391,"ENSG00000172183"
1392,"ENSG00000172061"

1393,"ENSG00000115998"
1394,"ENSG00000148824"
1395,"ENSG00000103047"
1396,"ENSG00000166912"
1397,"ENSG00000205581"
1398,"ENSG00000167552"
1399,"ENSG00000197841"
1400,"ENSG00000164440"
1401,"ENSG00000109205"
1402,"ENSG00000108107"
1403,"ENSG00000100979"
1404,"ENSG00000157870"
1405,"ENSG00000105668"
1406,"ENSG00000216921"
1407,"ENSG00000140807"
1408,"ENSG00000138798"
1409,"ENSG00000135744"
1410,"ENSG00000100201"
1411,"ENSG00000103160"
1412,"ENSG00000125449"
1413,"ENSG00000139155"
1414,"ENSG00000172081"
1415,"ENSG00000167759"
1416,"ENSG00000187950"
1417,"ENSG00000066379"
1418,"ENSG00000105202"
1419,"ENSG00000175711"
1420,"ENSG00000065882"
1421,"ENSG00000101447"
1422,"ENSG00000268175"
1423,"ENSG00000118680"
1424,"ENSG00000138593"
1425,"ENSG00000121594"
1426,"ENSG00000120694"
1427,"ENSG00000203667"
1428,"ENSG00000152527"
1429,"ENSG00000177868"
1430,"ENSG00000124772"
1431,"ENSG00000171109"
1432,"ENSG00000151773"
1433,"ENSG00000085377"
1434,"ENSG00000226124"
1435,"ENSG00000085721"
1436,"ENSG00000162980"
1437,"ENSG00000128951"
1438,"ENSG00000144681"
1439,"ENSG00000108798"
1440,"ENSG00000131591"

1441,"ENSG00000139719"
1442,"ENSG00000125731"
1443,"ENSG00000151632"
1444,"ENSG00000166509"
1445,"ENSG00000135677"
1446,"ENSG00000245317"
1447,"ENSG00000138621"
1448,"ENSG00000005884"
1449,"ENSG00000164172"
1450,"ENSG00000173848"
1451,"ENSG00000258366"
1452,"ENSG00000166482"
1453,"ENSG00000055732"
1454,"ENSG00000188372"
1455,"ENSG00000155307"
1456,"ENSG00000154645"
1457,"ENSG00000167077"
1458,"ENSG00000184428"
1459,"ENSG00000119977"
1460,"ENSG00000198189"
1461,"ENSG00000160785"
1462,"ENSG00000182919"
1463,"ENSG00000112837"
1464,"ENSG00000138698"
1465,"ENSG00000163319"
1466,"ENSG00000139865"
1467,"ENSG00000187624"
1468,"ENSG00000174791"
1469,"ENSG00000088766"
1470,"ENSG00000185519"
1471,"ENSG00000145990"
1472,"ENSG00000120832"
1473,"ENSG00000165188"
1474,"ENSG00000259649"
1475,"ENSG00000189269"
1476,"ENSG00000186235"
1477,"ENSG00000138035"
1478,"ENSG00000103199"
1479,"ENSG00000171729"
1480,"ENSG00000134318"
1481,"ENSG00000114383"
1482,"ENSG00000198453"
1483,"ENSG00000172340"
1484,"ENSG00000162746"
1485,"ENSG00000167964"
1486,"ENSG00000107290"
1487,"ENSG00000162104"
1488,"ENSG00000182325"

1489,"ENSG00000002587"
1490,"ENSG00000174021"
1491,"ENSG00000139324"
1492,"ENSG00000078246"
1493,"ENSG00000131759"
1494,"ENSG00000104731"
1495,"ENSG00000073050"
1496,"ENSG00000163006"
1497,"ENSG00000143067"
1498,"ENSG00000184945"
1499,"ENSG00000119673"
1500,"ENSG00000069345"
1501,"ENSG00000114857"
1502,"ENSG00000175806"
1503,"ENSG00000177885"
1504,"ENSG00000163510"
1505,"ENSG00000149294"
1506,"ENSG00000120008"
1507,"ENSG00000256053"
1508,"ENSG00000003987"
1509,"ENSG00000150893"
1510,"ENSG00000128254"
1511,"ENSG00000089818"
1512,"ENSG00000182534"
1513,"ENSG00000152213"
1514,"ENSG00000163697"
1515,"ENSG00000105479"
1516,"ENSG00000197054"
1517,"ENSG00000165643"
1518,"ENSG00000136546"
1519,"ENSG00000134030"
1520,"ENSG00000138795"
1521,"ENSG00000137098"
1522,"ENSG00000115274"
1523,"ENSG00000100599"
1524,"ENSG00000022556"
1525,"ENSG00000130005"
1526,"ENSG00000113318"
1527,"ENSG00000105426"
1528,"ENSG00000161981"
1529,"ENSG00000182324"
1530,"ENSG00000163512"
1531,"ENSG00000163430"
1532,"ENSG00000105171"
1533,"ENSG00000108448"
1534,"ENSG00000092051"
1535,"ENSG00000180902"
1536,"ENSG00000128284"

1537,"ENSG00000162244"
1538,"ENSG00000185842"
1539,"ENSG00000090581"
1540,"ENSG00000166333"
1541,"ENSG00000126464"
1542,"ENSG00000247626"
1543,"ENSG00000162642"
1544,"ENSG00000125743"
1545,"ENSG00000175782"
1546,"ENSG00000111540"
1547,"ENSG00000102554"
1548,"ENSG00000008516"
1549,"ENSG00000143194"
1550,"ENSG00000196642"
1551,"ENSG00000165828"
1552,"ENSG00000217702"
1553,"ENSG00000161888"
1554,"ENSG00000101457"
1555,"ENSG00000134905"
1556,"ENSG00000066027"
1557,"ENSG00000147854"
1558,"ENSG00000196937"
1559,"ENSG00000184923"
1560,"ENSG00000006118"
1561,"ENSG00000122012"
1562,"ENSG00000100353"
1563,"ENSG00000101200"
1564,"ENSG00000124713"
1565,"ENSG00000124253"
1566,"ENSG00000187990"
1567,"ENSG00000168255"
1568,"C8orf73"
1569,"ENSG00000185088"
1570,"ENSG00000050438"
1571,"ENSG00000124145"
1572,"ENSG00000116497"
1573,"ENSG00000151552"
1574,"ENSG00000151208"
1575,"ENSG00000115091"
1576,"ENSG00000050426"
1577,"ENSG00000105755"
1578,"ENSG00000273173"
1579,"ABHD16B"
1580,"ENSG00000159184"
1581,"ENSG00000170270"
1582,"ENSG00000184227"
1583,"ENSG00000196591"
1584,"ENSG00000124275"

1585,"ENSG00000105939"
1586,"ENSG00000141068"
1587,"ENSG00000010256"
1588,"ENSG00000103460"
1589,"ENSG00000197982"
1590,"ENSG00000145868"
1591,"ENSG00000101605"
1592,"ENSG00000141198"
1593,"ENSG00000138495"
1594,"ENSG00000165804"
1595,"ENSG00000198707"
1596,"ENSG00000177951"
1597,"ENSG00000187607"
1598,"ENSG00000026652"
1599,"ENSG00000132561"
1600,"ENSG00000129197"
1601,"ENSG00000186889"
1602,"ENSG00000144741"
1603,"ENSG00000099866"
1604,"ENSG00000184809"
1605,"ENSG00000006606"
1606,"ENSG00000136381"
1607,"ENSG00000048028"
1608,"ENSG00000099904"
1609,"ENSG00000170949"
1610,"ENSG00000106689"
1611,"ENSG00000133250"
1612,"ENSG00000181240"
1613,"ENSG00000100296"
1614,"ENSG00000181656"
1615,"ENSG00000170486"
1616,"ENSG00000166411"
1617,"ENSG00000180190"
1618,"ENSG00000143036"
1619,"ENSG00000196072"
1620,"ENSG00000205336"
1621,"ENSG00000132676"
1622,"ENSG00000100883"
1623,"ENSG00000137731"
1624,"ENSG00000180730"
1625,"ENSG00000162782"
1626,"ENSG00000215440"
1627,"ENSG00000162078"
1628,"ENSG00000188177"
1629,"ENSG00000197416"
1630,"ENSG00000185988"
1631,"ENSG00000141698"
1632,"ENSG00000049249"

1633,"ENSG00000151651"
1634,"ENSG00000088827"
1635,"ENSG00000138119"
1636,"ENSG00000181195"
1637,"ENSG00000225399"
1638,"ENSG00000105518"
1639,"ENSG00000173762"
1640,"ENSG00000211455"
1641,"ENSG00000196586"
1642,"ENSG00000169570"
1643,"ENSG00000257218"
1644,"ENSG00000174950"
1645,"ENSG00000086827"
1646,"ENSG00000047579"
1647,"ENSG00000236279"
1648,"ENSG00000142330"
1649,"ENSG00000053918"
1650,"ENSG00000100068"
1651,"ENSG00000171044"
1652,"ENSG00000161813"
1653,"ENSG00000179195"
1654,"ENSG00000149806"
1655,"ENSG00000196693"
1656,"ENSG00000146858"
1657,"ENSG00000122417"
1658,"ENSG00000164535"
1659,"ENSG00000141519"
1660,"ENSG00000170074"
1661,"ENSG00000196071"
1662,"ENSG00000117533"
1663,"ENSG00000153303"
1664,"ENSG00000111196"
1665,"ENSG00000197016"
1666,"ENSG00000181856"
1667,"ENSG00000116698"
1668,"ENSG00000172262"
1669,"ENSG00000135094"
1670,"ENSG00000138303"
1671,"ENSG00000232013"
1672,"ENSG00000224186"
1673,"ENSG00000160310"
1674,"ENSG00000125999"
1675,"ENSG00000179950"
1676,"ENSG00000198794"
1677,"ENSG00000162627"
1678,"ENSG00000182759"
1679,"ENSG00000173264"
1680,"ENSG00000108829"

1681,"ENSG00000160190"
1682,"ENSG00000184599"
1683,"ENSG00000134248"
1684,"ENSG00000129465"
1685,"ENSG00000173376"
1686,"ENSG00000125386"
1687,"ENSG00000162779"
1688,"ENSG00000137942"
1689,"ENSG00000175416"
1690,"ENSG00000175084"
1691,"ENSG00000186517"
1692,"ENSG00000094755"
1693,"ENSG00000137713"
1694,"ENSG00000124743"
1695,"ENSG00000126231"
1696,"ENSG00000140931"
1697,"ENSG00000183696"
1698,"ENSG00000174695"
1699,"ENSG00000139410"
1700,"ENSG00000010704"
1701,"ENSG00000160570"
1702,"ENSG00000131711"
1703,"ENSG00000123505"
1704,"ENSG00000204084"
1705,"ENSG00000075399"
1706,"ENSG00000175040"
1707,"ENSG00000170881"
1708,"ENSG00000100211"
1709,"ENSG00000204866"
1710,"ENSG00000144407"
1711,"ENSG00000198250"
1712,"ENSG00000189339"
1713,"ENSG00000112294"
1714,"ENSG00000088053"
1715,"ENSG00000196812"
1716,"ENSG00000066279"
1717,"ENSG00000100100"
1718,"ENSG00000107099"
1719,"ENSG00000171806"
1720,"ENSG00000111665"
1721,"ENSG00000225526"
1722,"ENSG00000124256"
1723,"ENSG00000156463"
1724,"ENSG00000163879"
1725,"ENSG00000151532"
1726,"ENSG00000127561"
1727,"ENSG00000135540"
1728,"ENSG00000169967"

1729,"ENSG00000132313"
1730,"ENSG00000168092"
1731,"ENSG00000149485"
1732,"ENSG00000180785"
1733,"ENSG00000128944"
1734,"ENSG00000189280"
1735,"ENSG00000153976"
1736,"ENSG00000002745"
1737,"ENSG00000116560"
1738,"ENSG00000121005"
1739,"ENSG00000084110"
1740,"ENSG00000129595"
1741,"ENSG00000172986"
1742,"ENSG00000124006"
1743,"ENSG00000108691"
1744,"ENSG00000198521"
1745,"ENSG00000167173"
1746,"ENSG00000139651"
1747,"ENSG00000185585"
1748,"ENSG00000129116"
1749,"ENSG00000055957"
1750,"ENSG00000149300"
1751,"ENSG00000213533"
1752,"ENSG00000144868"
1753,"ENSG00000165526"
1754,"ENSG00000108819"
1755,"ENSG00000077152"
1756,"ENSG00000165905"
1757,"ENSG00000107902"
1758,"ENSG00000165131"
1759,"ENSG00000198937"
1760,"ENSG00000142669"
1761,"ENSG00000157800"
1762,"ENSG00000113593"
1763,"ENSG00000136485"
1764,"ENSG00000248874"
1765,"ENSG00000023909"
1766,"ENSG00000176022"
1767,"ENSG00000085733"
1768,"ENSG00000163050"
1769,"ENSG00000143412"
1770,"ENSG00000166228"
1771,"ENSG00000180999"
1772,"ENSG00000125945"
1773,"ENSG00000099250"
1774,"ENSG00000184374"
1775,"ENSG00000255374"
1776,"ENSG00000177990"

1777,"ENSG00000206384"
1778,"ENSG00000124251"
1779,"ENSG00000095932"
1780,"ENSG00000100721"
1781,"ENSG00000178904"
1782,"ENSG00000158246"
1783,"ENSG00000081760"
1784,"ENSG00000183977"
1785,"ENSG00000183258"
1786,"ENSG00000130713"
1787,"ENSG00000188820"
1788,"ENSG00000100104"
1789,"ENSG00000121848"
1790,"ENSG00000137393"
1791,"ENSG00000174373"
1792,"ENSG00000168411"
1793,"ENSG00000145623"
1794,"ENSG00000165476"
1795,"ENSG00000132507"
1796,"ENSG00000106113"
1797,"ENSG00000087258"
1798,"ENSG00000086232"
1799,"ENSG00000110074"
1800,"ENSG00000116406"
1801,"ENSG00000130731"
1802,"ENSG00000197584"
1803,"ENSG00000240771"
1804,"ENSG00000183186"
1805,"ENSG00000260362"
1806,"ENSG00000113520"
1807,"ENSG00000152952"
1808,"ENSG00000254999"
1809,"ENSG00000178028"
1810,"ENSG00000168876"
1811,"ENSG00000135912"
1812,"ENSG00000171517"
1813,"ENSG00000095002"
1814,"ENSG00000183354"
1815,"ENSG00000183621"
1816,"ENSG00000165819"
1817,"ENSG00000244094"
1818,"ENSG00000117519"
1819,"ENSG00000131473"
1820,"ENSG00000079263"
1821,"ENSG00000254614"
1822,"ENSG00000164237"
1823,"ENSG00000145757"
1824,"ENSG00000146856"

1825,"ENSG00000170382"
1826,"ENSG00000011243"
1827,"ENSG00000078699"
1828,"ENSG00000212807"
1829,"ENSG00000059145"
1830,"ENSG00000026103"
1831,"ENSG00000115266"
1832,"ENSG00000116977"
1833,"ENSG00000145214"
1834,"ENSG00000069956"
1835,"ENSG00000186298"
1836,"ENSG00000159023"
1837,"ENSG00000090266"
1838,"ENSG00000196109"
1839,"ENSG00000214194"
1840,"ENSG00000169836"
1841,"ENSG00000106392"
1842,"ENSG00000197467"
1843,"ENSG00000038210"
1844,"ENSG00000163041"
1845,"ENSG00000132305"
1846,"ENSG00000144026"
1847,"ENSG00000122970"
1848,"ENSG00000127054"
1849,"ENSG00000136738"
1850,"ENSG00000204859"
1851,"ENSG00000137364"
1852,"ENSG00000057657"
1853,"ENSG00000186575"
1854,"ENSG00000150756"
1855,"ENSG00000090539"
1856,"ENSG00000258890"
1857,"ENSG00000167555"
1858,"ENSG00000178038"
1859,"ENSG00000171150"
1860,"ENSG00000141569"
1861,"ENSG00000120333"
1862,"ENSG00000013275"
1863,"ENSG00000171813"
1864,"ENSG00000160282"
1865,"ENSG00000185033"
1866,"ENSG00000181458"
1867,"ENSG00000151575"
1868,"ENSG00000171189"
1869,"ENSG00000111906"
1870,"ENSG00000182446"
1871,"ENSG00000048740"
1872,"ENSG00000164040"

1873,"ENSG00000044459"
1874,"ENSG00000183576"
1875,"ENSG00000107745"
1876,"ENSG00000080493"
1877,"ENSG00000145949"
1878,"ENSG00000180998"
1879,"ENSG00000132259"
1880,"ENSG00000139880"
1881,"ENSG00000134283"
1882,"ENSG00000166321"
1883,"ENSG00000160201"
1884,"ENSG00000178055"
1885,"ENSG00000197859"
1886,"ENSG00000142102"
1887,"ENSG00000101246"
1888,"ENSG00000179636"
1889,"ENSG00000184497"
1890,"ENSG00000065526"
1891,"ENSG00000169062"
1892,"ENSG00000164825"
1893,"ENSG00000165443"
1894,"ENSG00000168101"
1895,"ENSG00000120332"
1896,"ENSG00000206177"
1897,"ENSG00000182372"
1898,"ENSG00000138834"
1899,"ENSG00000166035"
1900,"ENSG00000187010"
1901,"ENSG00000104671"
1902,"ENSG00000181885"
1903,"ENSG00000107779"
1904,"ENSG00000113328"
1905,"ENSG00000123360"
1906,"ENSG00000213672"
1907,"ENSG00000006194"
1908,"ENSG00000154134"
1909,"ENSG00000164366"
1910,"ENSG00000169992"
1911,"ENSG00000184347"
1912,"ENSG00000090316"
1913,"ENSG00000006744"
1914,"ENSG00000173467"
1915,"ENSG00000151233"
1916,"ENSG00000135074"
1917,"ENSG00000172201"
1918,"ENSG00000188000"
1919,"ENSG00000151693"
1920,"ENSG00000132010"

1921,"ENSG00000011083"
1922,"ENSG00000166710"
1923,"ENSG00000112763"
1924,"ENSG00000188906"
1925,"ENSG00000261652"
1926,"ENSG00000116288"
1927,"ENSG00000148204"
1928,"ENSG00000129437"
1929,"ENSG00000213551"
1930,"ENSG00000118690"
1931,"ENSG00000197362"
1932,"ENSG00000179869"
1933,"ENSG00000172346"
1934,"ENSG00000213366"
1935,"ENSG00000184786"
1936,"ENSG00000110427"
1937,"ENSG00000104518"
1938,"ENSG00000083290"
1939,"ENSG00000133812"
1940,"ENSG00000163600"
1941,"ENSG00000165943"
1942,"ENSG00000182180"
1943,"ENSG00000150401"
1944,"ENSG00000125834"
1945,"ENSG00000168806"
1946,"ENSG00000106560"
1947,"ENSG00000111802"
1948,"ENSG00000168394"
1949,"ENSG00000111321"
1950,"ENSG00000145040"
1951,"ENSG00000142937"
1952,"ENSG00000124380"
1953,"ENSG00000145736"
1954,"ENSG00000159228"
1955,"ENSG00000064199"
1956,"ENSG00000143756"
1957,"ENSG00000164597"
1958,"ENSG00000114744"
1959,"ENSG00000090447"
1960,"ENSG00000173728"
1961,"ENSG00000198774"
1962,"ENSG00000204519"
1963,"ENSG00000131019"
1964,"ENSG00000006652"
1965,"ENSG00000189190"
1966,"ENSG00000002746"
1967,"ENSG00000196476"
1968,"ENSG00000112110"

1969,"ENSG00000100575"
1970,"ENSG00000105376"
1971,"ENSG00000162654"
1972,"ENSG00000099942"
1973,"ENSG00000143631"
1974,"ENSG00000147485"
1975,"ENSG00000185760"
1976,"ENSG00000131375"
1977,"ENSG00000183734"
1978,"ENSG00000003393"
1979,"ENSG00000212864"
1980,"ENSG00000104938"
1981,"ENSG00000135241"
1982,"ENSG00000075391"
1983,"ENSG00000117335"
1984,"ENSG00000140406"
1985,"ENSG00000155158"
1986,"ENSG00000082175"
1987,"ENSG00000111144"
1988,"ENSG00000091844"
1989,"ENSG00000112679"
1990,"ENSG00000184897"
1991,"ENSG00000204514"
1992,"ENSG00000130812"
1993,"ENSG00000071054"
1994,"ENSG00000154359"
1995,"ENSG00000177663"
1996,"ENSG00000173261"
1997,"ENSG00000188386"
1998,"ENSG00000105669"
1999,"ENSG00000124535"
2000,"ENSG00000103365"
2001,"ENSG00000181588"
2002,"ENSG00000183798"
2003,"ENSG00000100749"
2004,"ENSG00000066777"
2005,"ENSG00000144792"
2006,"ENSG00000052795"
2007,"ENSG00000123384"
2008,"ENSG00000132199"
2009,"ENSG00000135269"
2010,"ENSG00000182979"
2011,"ENSG00000092969"
2012,"ENSG00000124780"
2013,"ENSG00000137831"
2014,"ENSG00000213901"
2015,"ENSG00000062282"
2016,"ENSG00000245017"

2017,"ENSG00000105185"
2018,"ENSG00000239704"
2019,"ENSG00000099977"
2020,"ENSG00000167037"
2021,"ENSG00000164976"
2022,"ENSG00000085265"
2023,"ENSG00000167766"
2024,"ENSG00000105323"
2025,"ENSG00000049283"
2026,"ENSG00000160886"
2027,"ENSG00000125375"
2028,"ENSG00000143815"
2029,"ENSG00000119723"
2030,"ENSG00000111845"
2031,"ENSG00000171174"
2032,"ENSG00000161249"
2033,"ENSG00000090863"
2034,"ENSG00000123268"
2035,"ENSG00000178809"
2036,"ENSG00000005100"
2037,"ENSG00000142794"
2038,"ENSG00000169629"
2039,"ENSG00000153107"
2040,"ENSG00000107147"
2041,"ENSG00000167103"
2042,"ENSG00000145649"
2043,"ENSG00000213782"
2044,"ENSG00000197147"
2045,"ENSG00000066117"
2046,"ENSG00000170892"
2047,"ENSG00000162444"
2048,"ENSG00000118816"
2049,"ENSG00000174574"
2050,"ENSG00000168386"
2051,"ENSG00000104866"
2052,"ENSG00000067560"
2053,"ENSG00000127337"
2054,"ENSG00000183648"
2055,"ENSG00000141576"
2056,"ENSG00000204577"
2057,"ENSG00000172209"
2058,"ENSG00000013810"
2059,"ENSG00000105248"
2060,"ENSG00000181513"
2061,"ENSG00000127980"
2062,"ENSG00000218739"
2063,"ENSG00000158417"
2064,"ENSG00000124587"

2065,"ENSG00000157214"
2066,"ENSG00000196236"
2067,"ENSG00000015592"
2068,"ENSG00000092295"
2069,"ENSG00000105063"
2070,"ENSG00000131400"
2071,"ENSG00000164855"
2072,"ENSG00000159387"
2073,"ENSG00000175970"
2074,"ENSG00000146904"
2075,"ENSG00000027075"
2076,"ENSG00000158158"
2077,"ENSG00000239961"
2078,"ENSG00000196814"
2079,"ENSG00000187821"
2080,"ENSG00000085465"
2081,"ENSG00000109189"
2082,"ENSG00000196233"
2083,"ENSG00000186160"
2084,"ENSG00000154889"
2085,"ENSG00000106809"
2086,"ENSG00000116132"
2087,"ENSG00000125458"
2088,"ENSG00000135248"
2089,"ENSG00000129990"
2090,"ENSG00000151014"
2091,"ENSG00000101417"
2092,"ENSG00000168906"
2093,"ENSG00000215529"
2094,"ENSG00000119314"
2095,"ENSG00000104529"
2096,"ENSG00000159267"
2097,"ENSG00000234719"
2098,"ENSG00000137074"
2099,"ENSG00000106263"
2100,"ENSG00000153162"
2101,"ENSG00000133800"
2102,"ENSG00000160180"
2103,"ENSG00000215193"
2104,"ENSG00000196411"
2105,"ENSG00000152766"
2106,"ENSG00000196498"
2107,"ENSG00000124664"
2108,"ENSG00000168743"
2109,"ENSG00000135930"
2110,"ENSG00000267596"
2111,"ENSG00000205670"
2112,"ENSG00000211456"

2113,"ENSG00000203805"
2114,"ENSG00000184925"
2115,"ENSG00000108846"
2116,"ENSG00000116266"
2117,"ENSG00000110665"
2118,"ENSG00000011105"
2119,"ENSG00000148814"
2120,"ENSG00000128487"
2121,"ENSG00000162496"
2122,"ENSG00000149474"
2123,"ENSG00000072364"
2124,"ENSG00000178053"
2125,"ENSG00000161649"
2126,"ENSG00000178586"
2127,"ENSG00000140526"
2128,"ENSG00000174332"
2129,"ENSG00000242515"
2130,"ENSG00000154845"
2131,"ENSG00000137411"
2132,"ENSG00000130363"
2133,"ENSG00000108239"
2134,"ENSG00000129055"
2135,"ENSG00000165995"
2136,"ENSG00000188636"
2137,"ENSG00000196935"
2138,"ENSG00000162711"
2139,"ENSG00000146425"
2140,"ENSG00000150457"
2141,"ENSG00000256294"
2142,"ENSG00000175077"
2143,"ENSG00000136274"
2144,"ENSG00000100216"
2145,"ENSG00000163703"
2146,"ENSG00000133055"
2147,"ENSG00000143971"
2148,"ENSG00000234409"
2149,"ENSG00000057294"
2150,"ENSG00000178950"
2151,"ENSG00000167526"
2152,"ENSG00000139579"
2153,"ENSG00000158816"
2154,"ENSG00000188629"
2155,"ENSG00000163870"
2156,"ENSG00000163249"
2157,"ENSG00000100330"
2158,"ENSG00000166224"
2159,"ENSG00000134809"
2160,"ENSG00000134278"

2161,"ENSG00000135632"
2162,"ENSG00000144619"
2163,"ENSG00000172482"
2164,"ENSG00000117697"
2165,"ENSG00000171295"
2166,"ENSG00000125878"
2167,"ENSG00000164880"
2168,"ENSG00000138316"
2169,"ENSG00000171595"
2170,"ENSG00000162396"
2171,"ENSG00000168229"
2172,"ENSG00000115415"
2173,"ENSG00000175264"
2174,"ENSG00000173621"
2175,"ENSG00000117602"
2176,"ENSG00000197723"
2177,"ENSG00000145022"
2178,"ENSG00000166869"
2179,"ENSG00000143437"
2180,"ENSG00000161980"
2181,"ENSG00000079313"
2182,"ENSG00000175130"
2183,"ENSG00000145692"
2184,"ENSG00000169085"
2185,"ENSG00000093217"
2186,"ENSG00000055163"
2187,"ENSG00000142945"
2188,"ENSG00000178199"
2189,"ENSG00000125337"
2190,"ENSG00000171873"
2191,"ENSG00000129007"
2192,"ENSG00000221962"
2193,"ENSG00000065485"
2194,"ENSG00000152672"
2195,"ENSG00000171217"
2196,"ENSG00000143653"
2197,"ENSG00000164111"
2198,"ENSG00000206127"
2199,"ENSG00000189134"
2200,"ENSG00000166452"
2201,"ENSG00000172915"
2202,"ENSG00000011465"
2203,"ENSG00000225885"
2204,"ENSG00000130584"
2205,"ENSG00000147912"
2206,"ENSG00000198168"
2207,"ENSG00000147488"
2208,"ENSG00000102763"

2209,"ENSG00000163002"
2210,"ENSG00000204520"
2211,"ENSG00000228300"
2212,"ENSG00000182944"
2213,"ENSG00000161265"
2214,"ENSG00000153060"
2215,"ENSG00000253293"
2216,"ENSG00000136059"
2217,"ENSG00000164344"
2218,"ENSG00000128805"
2219,"ENSG00000143322"
2220,"ENSG00000120438"
2221,"ENSG00000155918"
2222,"ENSG00000175643"
2223,"ENSG00000149480"
2224,"ENSG00000152076"
2225,"ENSG00000148444"
2226,"GCFC1"
2227,"ENSG00000158747"
2228,"ENSG00000134744"
2229,"ENSG00000125503"
2230,"ENSG00000106018"
2231,"ENSG00000144736"
2232,"ENSG00000139190"
2233,"ENSG00000256660"
2234,"ENSG00000116459"
2235,"ENSG00000105738"
2236,"ENSG00000197798"
2237,"ENSG00000205560"
2238,"ENSG00000180596"
2239,"ENSG00000156574"
2240,"ENSG00000174226"
2241,"ENSG00000188015"
2242,"ENSG00000078668"
2243,"ENSG00000133739"
2244,"ENSG00000183773"
2245,"ENSG00000005238"
2246,"ENSG00000151623"
2247,"ENSG00000172939"
2248,"ENSG00000165152"
2249,"ENSG00000114279"
2250,"ENSG00000104490"
2251,"ENSG00000155749"
2252,"ENSG00000131626"
2253,"ENSG00000134470"
2254,"ENSG00000105655"
2255,"ENSG00000163161"
2256,"ENSG00000163389"

2257,"ENSG00000241186"
2258,"ENSG00000169490"
2259,"ENSG00000168569"
2260,"ENSG00000175575"
2261,"ENSG00000119718"
2262,"C6orf97"
2263,"ENSG00000164961"
2264,"ENSG00000126822"
2265,"ENSG00000080007"
2266,"ENSG00000160013"
2267,"ENSG00000146250"
2268,"ENSG00000154957"
2269,"ENSG00000176894"
2270,"ENSG00000101353"
2271,"ENSG00000102189"
2272,"ENSG00000146918"
2273,"ENSG00000130529"
2274,"ENSG00000101298"
2275,"ENSG00000170322"
2276,"ENSG00000125871"
2277,"ENSG00000105223"
2278,"ENSG00000170855"
2279,"ENSG00000126003"
2280,"ENSG00000157796"
2281,"ENSG00000137414"
2282,"ENSG00000111581"
2283,"ENSG00000159173"
2284,"ENSG00000154027"
2285,"ENSG00000137675"
2286,"ENSG00000128923"
2287,"ENSG00000124491"
2288,"ENSG00000185972"
2289,"ENSG00000116176"
2290,"ENSG00000070526"
2291,"ENSG00000072274"
2292,"ENSG00000064205"
2293,"ENSG00000124782"
2294,"ENSG00000110203"
2295,"ENSG00000113732"
2296,"ENSG00000197375"
2297,"ENSG00000171522"
2298,"ENSG00000156875"
2299,"ENSG00000172731"
2300,"ENSG00000221882"
2301,"ENSG00000118762"
2302,"ENSG00000184674"
2303,"ENSG00000125966"
2304,"ENSG00000110484"

2305,"ENSG00000148229"
2306,"ENSG00000171385"
2307,"ENSG00000162688"
2308,"ENSG00000225830"
2309,"ENSG00000119471"
2310,"ENSG00000144645"
2311,"ENSG00000136731"
2312,"ENSG00000099998"
2313,"ENSG00000109927"
2314,"ENSG00000178974"
2315,"ENSG00000069943"
2316,"ENSG00000132881"
2317,"ENSG00000131871"
2318,"ENSG00000163251"
2319,"ENSG00000243667"
2320,"ENSG00000225614"
2321,"ENSG00000145741"
2322,"ENSG00000132932"
2323,"ENSG00000213463"
2324,"ENSG00000197140"
2325,"ENSG00000178187"
2326,"ENSG00000100941"
2327,"ENSG00000171206"
2328,"ENSG00000239672"
2329,"ENSG00000132646"
2330,"ENSG00000111696"
2331,"ENSG00000140093"
2332,"ENSG00000139624"
2333,"ENSG00000162769"
2334,"ENSG00000176244"
2335,"C8orf84"
2336,"ENSG00000230567"
2337,"ENSG00000146215"
2338,"ENSG00000178722"
2339,"ENSG00000061337"
2340,"ENSG00000102900"
2341,"ENSG00000112246"
2342,"ENSG00000197969"
2343,"ENSG00000130164"
2344,"ENSG00000178913"
2345,"ENSG00000138592"
2346,"ENSG00000140538"
2347,"ENSG00000188778"
2348,"ENSG00000115421"
2349,"ENSG00000205639"
2350,"ENSG00000082397"
2351,"ENSG00000148688"
2352,"ENSG00000104852"

2353,"ENSG00000138376"
2354,"ENSG00000162398"
2355,"ENSG00000122863"
2356,"ENSG00000121905"
2357,"ENSG00000151388"
2358,"ENSG00000153815"
2359,"ENSG00000159086"
2360,"ENSG00000107614"
2361,"ENSG00000113360"
2362,"ENSG00000167895"
2363,"ENSG00000021574"
2364,"ENSG00000128185"
2365,"ENSG00000185437"
2366,"ENSG00000162384"
2367,"ENSG00000095713"
2368,"ENSG00000111615"
2369,"ENSG00000118292"
2370,"ENSG00000112357"
2371,"ENSG00000072849"
2372,"ENSG00000166716"
2373,"ENSG00000198739"
2374,"ENSG00000160058"
2375,"ENSG00000130921"
2376,"ENSG00000175329"
2377,"ENSG00000060762"
2378,"ENSG00000187715"
2379,"ENSG00000136783"
2380,"ENSG00000100568"
2381,"ENSG00000164251"
2382,"ENSG00000011422"
2383,"ENSG00000047315"
2384,"ENSG00000204969"
2385,"ENSG00000145041"
2386,"ENSG00000133794"
2387,"ENSG00000170615"
2388,"ENSG00000094804"
2389,"ENSG00000114779"
2390,"ENSG00000204962"
2391,"ENSG00000128585"
2392,"ENSG00000185774"
2393,"ENSG00000183617"
2394,"ENSG00000146352"
2395,"C20orf94"
2396,"ENSG00000165929"
2397,"ENSG00000164334"
2398,"ENSG00000140279"
2399,"ENSG00000118971"
2400,"ENSG00000197013"

2401,"ENSG00000271303"
2402,"ENSG00000138669"
2403,"ENSG00000173193"
2404,"ENSG00000146054"
2405,"ENSG00000107485"
2406,"ENSG00000204644"
2407,"ENSG00000168913"
2408,"ENSG00000133195"
2409,"ENSG00000165409"
2410,"ENSG00000065970"
2411,"ENSG00000198585"
2412,"ENSG00000242616"
2413,"ENSG00000104953"
2414,"ENSG00000124749"
2415,"ENSG00000092871"
2416,"ENSG00000121691"
2417,"ENSG00000150477"
2418,"ENSG00000221983"
2419,"ENSG00000071246"
2420,"ENSG00000143320"
2421,"ENSG00000142512"
2422,"ENSG00000251664"
2423,"ENSG00000164294"
2424,"ENSG00000174059"
2425,"ENSG00000059758"
2426,"ENSG00000167987"
2427,"ENSG00000229676"
2428,"ENSG00000005075"
2429,"ENSG00000137767"
2430,"ENSG00000090263"
2431,"ENSG00000132965"
2432,"ENSG00000162976"
2433,"ENSG00000100225"
2434,"ENSG00000167723"
2435,"ENSG00000103353"
2436,"ENSG00000137142"
2437,"ENSG00000092531"
2438,"ENSG00000078295"
2439,"ENSG00000118777"
2440,"ENSG00000172366"
2441,"ENSG00000197562"
2442,"ENSG00000074966"
2443,"ENSG00000077348"
2444,"ENSG00000155629"
2445,"ENSG00000085231"
2446,"ENSG00000108509"
2447,"ENSG00000163312"
2448,"ENSG00000156411"

2449,"ENSG00000256436"
2450,"ENSG00000063978"
2451,"ENSG00000164610"
2452,"ENSG00000167380"
2453,"ENSG00000097007"
2454,"ENSG00000214309"
2455,"ENSG00000107020"
2456,"ENSG00000143079"
2457,"ENSG00000131471"
2458,"ENSG00000173801"
2459,"ENSG00000169583"
2460,"ENSG00000188157"
2461,"ENSG00000107798"
2462,"ENSG00000120733"
2463,"ENSG00000134940"
2464,"ENSG00000112182"
2465,"ENSG00000269558"
2466,"ENSG00000250588"
2467,"ENSG00000115942"
2468,"ENSG00000173421"
2469,"ENSG00000129353"
2470,"ENSG00000073792"
2471,"ENSG00000107738"
2472,"ENSG00000196417"
2473,"ENSG00000151718"
2474,"ENSG00000101255"
2475,"ENSG00000119699"
2476,"ENSG00000136235"
2477,"ENSG00000112782"
2478,"ENSG00000166803"
2479,"ENSG00000224383"
2480,"ENSG00000154736"
2481,"ENSG00000140265"
2482,"ENSG00000253251"
2483,"ENSG00000154099"
2484,"ENSG00000038945"
2485,"ENSG00000107077"
2486,"ENSG00000221963"
2487,"ENSG00000139505"
2488,"ENSG00000188938"
2489,"ENSG00000071794"
2490,"ENSG00000158717"
2491,"ENSG00000184584"
2492,"ENSG00000152926"
2493,"ENSG00000149050"
2494,"ENSG00000163812"
2495,"ENSG00000183044"
2496,"ENSG00000014164"

2497,"ENSG00000165985"
2498,"ENSG00000101493"
2499,"ENSG00000172716"
2500,"ENSG00000105559"
2501,"ENSG00000140829"
2502,"ENSG00000159674"
2503,"ENSG00000021826"
2504,"ENSG00000114473"
2505,"ENSG00000156990"
2506,"ENSG00000112514"
2507,"ENSG00000171121"
2508,"ENSG00000170448"
2509,"ENSG00000185304"
2510,"ENSG00000186049"
2511,"ENSG00000127527"
2512,"ENSG00000132541"
2513,"ENSG00000147592"
2514,"ENSG00000196268"
2515,"ENSG00000184545"
2516,"ENSG00000167702"
2517,"ENSG00000257591"
2518,"ENSG00000177889"
2519,"ENSG00000197702"
2520,"ENSG00000157106"
2521,"ENSG00000205426"
2522,"ENSG00000133048"
2523,"ENSG00000104228"
2524,"ENSG00000030304"
2525,"ENSG00000205476"
2526,"ENSG00000198113"
2527,"ENSG00000146700"
2528,"ENSG00000182134"
2529,"ENSG00000151360"
2530,"ENSG00000073008"
2531,"ENSG00000189195"
2532,"ENSG00000162598"
2533,"ENSG00000117834"
2534,"ENSG00000257335"
2535,"ENSG00000271605"
2536,"ENSG00000166260"
2537,"ENSG00000173714"
2538,"ENSG00000163444"
2539,"ENSG00000198848"
2540,"ENSG00000135776"
2541,"ENSG00000154928"
2542,"ENSG00000174013"
2543,"ENSG00000230778"
2544,"ENSG00000108582"

2545,"ENSG00000163040"
2546,"ENSG00000127328"
2547,"ENSG00000142046"
2548,"ENSG00000172992"
2549,"ENSG00000116670"
2550,"ENSG00000196431"
2551,"ENSG00000188039"
2552,"ENSG00000164144"
2553,"ENSG00000125485"
2554,"ENSG00000166478"
2555,"ENSG00000161850"
2556,"ENSG00000146281"
2557,"ENSG00000213995"
2558,"ENSG00000110700"
2559,"ENSG00000178814"
2560,"ENSG00000147324"
2561,"ENSG00000126861"
2562,"ENSG00000135617"
2563,"ENSG00000138182"
2564,"ENSG00000146285"
2565,"ENSG00000144867"
2566,"ENSG00000080200"
2567,"ENSG00000162585"
2568,"ENSG00000106565"
2569,"ENSG00000248458"
2570,"ENSG00000164073"
2571,"ENSG00000197008"
2572,"ENSG00000139112"
2573,"ENSG00000164556"
2574,"ENSG00000159409"
2575,"ENSG00000268996"
2576,"ENSG00000176593"
2577,"ENSG00000115896"
2578,"ENSG00000159063"
2579,"ENSG00000188747"
2580,"ENSG00000182963"
2581,"ENSG00000180071"
2582,"ENSG00000169641"
2583,"ENSG00000164929"
2584,"ENSG00000136379"
2585,"ENSG00000161513"
2586,"ENSG00000177971"
2587,"ENSG00000135766"
2588,"ENSG00000101019"
2589,"ENSG00000154252"
2590,"ENSG00000115525"
2591,"ENSG00000198720"
2592,"ENSG00000204839"

2593,"ENSG00000105499"
2594,"ENSG00000179855"
2595,"ENSG00000185829"
2596,"ENSG00000155875"
2597,"ENSG00000164776"
2598,"ENSG00000125166"
2599,"ENSG00000177042"
2600,"ENSG00000140157"
2601,"ENSG00000130347"
2602,"ENSG00000080189"
2603,"ENSG00000141219"
2604,"ENSG00000162817"
2605,"ENSG00000176399"
2606,"ENSG00000105072"
2607,"ENSG00000118307"
2608,"ENSG00000183172"
2609,"ENSG00000153404"
2610,"ENSG00000109680"
2611,"ENSG00000117906"
2612,"ENSG00000234511"
2613,"ENSG00000012124"
2614,"ENSG00000150347"
2615,"ENSG00000204659"
2616,"ENSG00000153832"
2617,"ENSG00000119787"
2618,"ENSG00000089057"
2619,"ENSG00000021300"
2620,"ENSG00000187688"
2621,"ENSG00000198018"
2622,"ENSG00000113248"
2623,"ENSG00000124374"
2624,"ENSG00000106477"
2625,"ENSG00000135480"
2626,"ENSG00000213977"
2627,"ENSG00000180953"
2628,"ENSG00000076604"
2629,"ENSG00000183813"
2630,"ENSG00000136908"
2631,"ENSG00000128604"
2632,"ENSG00000166261"
2633,"ENSG00000130348"
2634,"ENSG00000166896"
2635,"ENSG00000075945"
2636,"ENSG00000141086"
2637,"ENSG00000172115"
2638,"ENSG00000135678"
2639,"ENSG00000185787"
2640,"ENSG00000152061"

2641,"ENSG00000171234"
2642,"ENSG00000065621"
2643,"ENSG00000140287"
2644,"ENSG00000040487"
2645,"ENSG00000188846"
2646,"ENSG00000168763"
2647,"ENSG00000014919"
2648,"ENSG00000180370"
2649,"ENSG00000140543"
2650,"ENSG00000007171"
2651,"ENSG00000170903"
2652,"ENSG00000100987"
2653,"ENSG00000258289"
2654,"ENSG00000134086"
2655,"ENSG00000153214"
2656,"ENSG00000170889"
2657,"ENSG00000120256"
2658,"ENSG00000153820"
2659,"ENSG00000196735"
2660,"ENSG00000058729"
2661,"ENSG00000077585"
2662,"ENSG00000127603"
2663,"ENSG00000143643"
2664,"ENSG00000008513"
2665,"ENSG00000143153"
2666,"ENSG00000044090"
2667,"ENSG00000025039"
2668,"ENSG00000130703"
2669,"ENSG00000131100"
2670,"ENSG00000230873"
2671,"ENSG00000187800"
2672,"ENSG00000167325"
2673,"ENSG00000105193"
2674,"ENSG00000177191"
2675,"ENSG00000116198"
2676,"ENSG00000143155"
2677,"ENSG00000163466"
2678,"ENSG00000144559"
2679,"ENSG00000123838"
2680,"ENSG00000169567"
2681,"ENSG00000187013"
2682,"ENSG00000147852"
2683,"ENSG00000184144"
2684,"ENSG00000214078"
2685,"ENSG00000182362"
2686,"ENSG00000136932"
2687,"ENSG00000164010"
2688,"ENSG00000144451"

2689,"ENSG00000176208"
2690,"ENSG00000003509"
2691,"ENSG00000075785"
2692,"ENSG00000072786"
2693,"ENSG00000106344"
2694,"ENSG00000167693"
2695,"ENSG00000197498"
2696,"ENSG00000101557"
2697,"ENSG00000023171"
2698,"ENSG00000013503"
2699,"ENSG00000145494"
2700,"ENSG00000268688"
2701,"ENSG00000093134"
2702,"ENSG00000128271"
2703,"ENSG00000105254"
2704,"ENSG00000153113"
2705,"ENSG00000065609"
2706,"ENSG00000100029"
2707,"ENSG00000111254"
2708,"ENSG00000197928"
2709,"ENSG00000164241"
2710,"ENSG00000196535"
2711,"ENSG00000060718"
2712,"ENSG00000091483"
2713,"ENSG00000070882"
2714,"ENSG00000124641"
2715,"ENSG00000179335"
2716,"ENSG00000036672"
2717,"ENSG00000064687"
2718,"ENSG00000187242"
2719,"ENSG00000171747"
2720,"ENSG00000143498"
2721,"ENSG00000135778"
2722,"ENSG00000167210"
2723,"ENSG00000176692"
2724,"ENSG00000183508"
2725,"ENSG00000165097"
2726,"ENSG00000104679"
2727,"ENSG00000169087"
2728,"ENSG00000105993"
2729,"ENSG00000151445"
2730,"ENSG00000214212"
2731,"ENSG00000104976"
2732,"ENSG00000063241"
2733,"ENSG00000174775"
2734,"ENSG00000010072"
2735,"ENSG00000166927"
2736,"ENSG00000183251"

2737,"ENSG00000182923"
2738,"ENSG00000118420"
2739,"ENSG00000135318"
2740,"ENSG00000145020"
2741,"ENSG00000120215"
2742,"ENSG00000106236"
2743,"ENSG00000047932"
2744,"ENSG00000175911"
2745,"ENSG00000144959"
2746,"ENSG00000167615"
2747,"ENSG00000168397"
2748,"ENSG00000169180"
2749,"ENSG00000163882"
2750,"ENSG00000125388"
2751,"ENSG00000205981"
2752,"ENSG00000179580"
2753,"ENSG00000204104"
2754,"ENSG00000171130"
2755,"ENSG00000101162"
2756,"ENSG00000132840"
2757,"ENSG00000182117"
2758,"ENSG00000119042"
2759,"ENSG00000106948"
2760,"ENSG00000188321"
2761,"ENSG00000010292"
2762,"ENSG00000133116"
2763,"ENSG00000109062"
2764,"ENSG00000073849"
2765,"ENSG00000176971"
2766,"ENSG00000145817"
2767,"ENSG00000013392"
2768,"ENSG00000066084"
2769,"ENSG00000107742"
2770,"ENSG00000183155"
2771,"ENSG00000124523"
2772,"ENSG00000038002"
2773,"ENSG00000153902"
2774,"ENSG00000127586"
2775,"ENSG00000185561"
2776,"ENSG00000104689"
2777,"ENSG00000104763"
2778,"ENSG00000204060"
2779,"ENSG00000157500"
2780,"ENSG00000005020"
2781,"ENSG00000129315"
2782,"ENSG00000144035"
2783,"ENSG00000151948"
2784,"ENSG00000100288"

2785,"ENSG00000234949"
2786,"ENSG00000103995"
2787,"ENSG00000152749"
2788,"ENSG00000203907"
2789,"ENSG00000111275"
2790,"ENSG00000040608"
2791,"ENSG00000100276"
2792,"ENSG00000125779"
2793,"ENSG00000161798"
2794,"ENSG00000182870"
2795,"ENSG00000150455"
2796,"ENSG00000105419"
2797,"ENSG00000163145"
2798,"ENSG00000241127"
2799,"ENSG00000099992"
2800,"ENSG00000188672"
2801,"ENSG00000126878"
2802,"ENSG00000073921"
2803,"ENSG00000144535"
2804,"ENSG00000120915"
2805,"ENSG00000171067"
2806,"ENSG00000129158"
2807,"ENSG00000134243"
2808,"ENSG00000132952"
2809,"ENSG00000123975"
2810,"ENSG00000198551"
2811,"ENSG00000072121"
2812,"ENSG00000122484"
2813,"ENSG00000080839"
2814,"ENSG00000027001"
2815,"ENSG00000079739"
2816,"ENSG00000107929"
2817,"ENSG00000165480"
2818,"ENSG00000167941"
2819,"ENSG00000143632"
2820,"ENSG00000144711"
2821,"ENSG00000130766"
2822,"ENSG00000188171"
2823,"ENSG00000121774"
2824,"ENSG00000128617"
2825,"ENSG00000121410"
2826,"ENSG00000123689"
2827,"ENSG00000169247"
2828,"ENSG00000115084"
2829,"ENSG00000140750"
2830,"ENSG00000224712"
2831,"ENSG00000164118"
2832,"ENSG00000097046"

2833,"ENSG00000204628"
2834,"ENSG00000198700"
2835,"ENSG00000182836"
2836,"ENSG00000170956"
2837,"ENSG00000111291"
2838,"ENSG00000135040"
2839,"ENSG00000181163"
2840,"ENSG00000213865"
2841,"ENSG00000107036"
2842,"ENSG00000197885"
2843,"ENSG00000181827"
2844,"ENSG00000143740"
2845,"ENSG00000167747"
2846,"ENSG00000120158"
2847,"ENSG00000117650"
2848,"ENSG00000198435"
2849,"ENSG00000158411"
2850,"ENSG00000136243"
2851,"ENSG00000167130"
2852,"ENSG00000164404"
2853,"ENSG00000154162"
2854,"ENSG00000256229"
2855,"ENSG00000185100"
2856,"ENSG00000135847"
2857,"ENSG00000152822"
2858,"ENSG00000159069"
2859,"ENSG00000106991"
2860,"ENSG00000188730"
2861,"ENSG00000140527"
2862,"ENSG00000096092"
2863,"ENSG00000171033"
2864,"ENSG00000003056"
2865,"ENSG00000183833"
2866,"ENSG00000168903"
2867,"ENSG00000119771"
2868,"ENSG00000075240"
2869,"ENSG00000158296"
2870,"ENSG00000160867"
2871,"ENSG00000189144"
2872,"ENSG00000163625"
2873,"ENSG00000159167"
2874,"ENSG00000124831"
2875,"ENSG00000027847"
2876,"ENSG00000129518"
2877,"ENSG00000156017"
2878,"ENSG00000173113"
2879,"ENSG00000167216"
2880,"ENSG00000087095"

2881,"ENSG00000171492"
2882,"ENSG00000021645"
2883,"ENSG00000153575"
2884,"ENSG00000122557"
2885,"LRRC33"
2886,"ENSG00000138041"
2887,"ENSG00000160226"
2888,"ENSG00000072401"
2889,"ENSG00000103404"
2890,"ENSG00000124181"
2891,"ENSG00000102904"
2892,"ENSG00000167962"
2893,"ENSG00000150165"
2894,"ENSG00000164663"
2895,"ENSG00000163701"
2896,"ENSG00000100249"
2897,"ENSG00000132485"
2898,"ENSG00000129538"
2899,"ENSG00000109944"
2900,"ENSG00000196465"
2901,"ENSG00000178462"
2902,"ENSG00000179832"
2903,"ENSG00000136718"
2904,"ENSG00000142515"
2905,"ENSG00000107554"
2906,"ENSG00000155275"
2907,"ENSG00000167207"
2908,"ENSG00000243056"
2909,"ENSG00000164023"
2910,"ENSG00000010270"
2911,"ENSG00000183978"
2912,"ENSG00000163785"
2913,"ENSG00000143303"
2914,"ENSG00000156639"
2915,"ENSG00000119969"
2916,"ENSG00000158769"
2917,"ENSG00000142149"
2918,"ENSG00000153029"
2919,"ENSG00000164494"
2920,"ENSG00000074047"
2921,"ENSG00000067221"
2922,"ENSG00000145331"
2923,"ENSG00000111834"
2924,"ENSG00000171703"
2925,"ENSG00000205307"
2926,"ENSG00000136807"
2927,"ENSG00000132423"
2928,"ENSG00000182450"

2929,"ENSG00000181778"
2930,"ENSG00000139344"
2931,"ENSG00000173715"
2932,"ENSG00000102978"
2933,"ENSG00000173208"
2934,"ENSG00000249915"
2935,"ENSG00000162365"
2936,"ENSG00000127329"
2937,"ENSG00000182218"
2938,"ENSG00000157999"
2939,"ENSG00000185745"
2940,"ENSG00000183914"
2941,"ENSG00000132819"
2942,"ENSG00000010278"
2943,"ENSG00000169902"
2944,"ENSG00000086062"
2945,"ENSG00000197557"
2946,"ENSG00000100796"
2947,"ENSG00000125498"
2948,"ENSG00000134769"
2949,"ENSG00000161714"
2950,"ENSG00000087299"
2951,"ENSG00000163428"
2952,"ENSG00000113407"
2953,"ENSG00000112304"
2954,"ENSG00000182247"
2955,"ENSG00000158882"
2956,"ENSG00000134815"
2957,"ENSG00000131966"
2958,"ENSG00000142494"
2959,"ENSG00000185917"
2960,"ENSG00000159788"
2961,"ENSG00000162069"
2962,"ENSG00000141682"
2963,"ENSG00000121892"
2964,"ENSG00000083817"
2965,"ENSG00000197763"
2966,"ENSG00000117481"
2967,"ENSG00000169469"
2968,"ENSG00000203782"
2969,"ENSG00000099910"
2970,"ENSG00000116514"
2971,"ENSG00000159593"
2972,"ENSG00000103091"
2973,"ENSG00000110628"
2974,"ENSG00000087253"
2975,"ENSG00000143198"
2976,"ENSG00000162367"

2977,"ENSG00000242441"
2978,"ENSG00000123358"
2979,"ENSG00000100577"
2980,"ENSG00000117598"
2981,"ENSG00000161091"
2982,"ENSG00000136449"
2983,"ENSG00000188312"
2984,"ENSG00000163950"
2985,"ENSG00000123992"
2986,"ENSG00000182544"
2987,"ENSG00000164695"
2988,"ENSG00000174720"
2989,"ENSG00000002933"
2990,"ENSG00000057019"
2991,"ENSG00000174358"
2992,"ENSG00000243709"
2993,"ENSG00000031003"
2994,"ENSG00000169131"
2995,"ENSG00000148362"
2996,"ENSG00000196262"
2997,"ENSG00000163558"
2998,"ENSG00000212916"
2999,"ENSG00000136425"
3000,"ENSG00000179588"
3001,"ENSG00000177380"
3002,"ENSG00000148288"
3003,"ENSG00000118513"
3004,"ENSG00000141644"
3005,"ENSG00000137691"
3006,"ENSG00000183751"
3007,"ENSG00000130449"
3008,"ENSG00000160293"
3009,"ENSG00000168090"
3010,"ENSG00000137269"
3011,"ENSG00000100884"
3012,"ENSG00000148218"
3013,"ENSG00000074416"
3014,"ENSG00000144791"
3015,"ENSG00000198203"
3016,"ENSG00000160716"
3017,"ENSG00000214022"
3018,"ENSG00000140280"
3019,"ENSG00000042317"
3020,"ENSG00000116032"
3021,"ENSG00000155313"
3022,"ENSG00000169738"
3023,"ENSG00000151468"
3024,"ENSG00000145604"

3025,"ENSG00000089163"
3026,"ENSG00000106538"
3027,"ENSG00000010165"
3028,"ENSG00000142686"
3029,"ENSG00000163754"
3030,"ENSG00000168434"
3031,"ENSG00000121966"
3032,"ENSG00000057608"
3033,"ENSG00000065328"
3034,"ENSG00000100811"
3035,"ENSG00000218891"
3036,"ENSG00000101004"
3037,"ENSG00000154760"
3038,"ENSG00000183748"
3039,"ENSG00000173818"
3040,"ENSG00000147813"
3041,"ENSG00000165899"
3042,"ENSG00000136840"
3043,"ENSG00000174749"
3044,"ENSG00000106603"
3045,"ENSG00000180113"
3046,"ENSG00000116120"
3047,"ENSG00000129028"
3048,"ENSG00000100364"
3049,"ENSG00000163517"
3050,"ENSG00000197563"
3051,"ENSG00000083123"
3052,"ENSG00000154174"
3053,"ENSG00000163814"
3054,"ENSG00000159556"
3055,"ENSG00000175115"
3056,"ENSG00000124126"
3057,"ENSG00000164379"
3058,"ENSG00000008853"
3059,"ENSG00000117505"
3060,"ENSG00000115446"
3061,"ENSG00000221829"
3062,"ENSG00000117226"
3063,"ENSG00000165502"
3064,"ENSG00000160209"
3065,"ENSG00000074621"
3066,"ENSG00000137204"
3067,"ENSG00000175170"
3068,"ENSG00000196684"
3069,"ENSG00000113790"
3070,"ENSG00000110786"
3071,"ENSG00000156973"
3072,"ENSG00000175582"

3073,"ENSG00000163406"
3074,"ENSG00000096060"
3075,"ENSG00000132341"
3076,"ENSG00000166347"
3077,"ENSG00000184517"
3078,"ENSG00000213132"
3079,"ENSG00000103313"
3080,"ENSG00000146707"
3081,"ENSG00000121064"
3082,"ENSG00000126088"
3083,"ENSG00000139433"
3084,"ENSG00000164576"
3085,"ENSG00000137285"
3086,"ENSG00000137547"
3087,"ENSG00000140319"
3088,"ENSG00000105771"
3089,"ENSG00000100427"
3090,"ENSG00000076662"
3091,"ENSG00000115020"
3092,"ENSG00000099139"
3093,"ENSG00000138039"
3094,"ENSG00000197696"
3095,"ENSG00000261794"
3096,"ENSG00000128829"
3097,"ENSG00000243284"
3098,"ENSG00000107864"
3099,"ENSG00000130045"
3100,"ENSG00000237515"
3101,"ENSG00000148290"
3102,"ENSG00000128253"
3103,"ENSG00000125851"
3104,"ENSG00000177283"
3105,"ENSG00000141933"
3106,"ENSG00000196345"
3107,"ENSG00000220205"
3108,"ENSG00000023191"
3109,"ENSG00000132436"
3110,"ENSG00000166343"
3111,"ENSG00000196275"
3112,"ENSG00000072609"
3113,"ENSG00000025434"
3114,"ENSG00000170634"
3115,"ENSG00000105708"
3116,"ENSG00000004534"
3117,"ENSG00000188735"
3118,"ENSG00000164631"
3119,"ENSG00000090339"
3120,"ENSG00000145354"

3121,"ENSG00000067369"
3122,"ENSG00000106004"
3123,"ENSG00000166845"
3124,"ENSG00000132781"
3125,"ENSG00000134330"
3126,"ENSG00000168071"
3127,"ENSG00000164989"
3128,"ENSG00000106526"
3129,"ENSG00000108559"
3130,"ENSG00000053900"
3131,"ENSG00000145220"
3132,"ENSG00000159592"
3133,"ENSG00000168237"
3134,"ENSG00000150783"
3135,"ENSG00000101463"
3136,"ENSG00000140623"
3137,"ENSG00000162882"
3138,"ENSG00000133059"
3139,"ENSG00000159322"
3140,"ENSG00000175745"
3141,"ENSG00000100815"
3142,"ENSG00000149922"
3143,"ENSG00000164081"
3144,"ENSG00000186792"
3145,"ENSG00000213047"
3146,"ENSG00000177981"
3147,"ENSG00000116675"
3148,"ENSG00000117477"
3149,"ENSG00000176920"
3150,"ENSG00000146083"
3151,"ENSG00000088808"
3152,"ENSG00000171877"
3153,"ENSG00000145194"
3154,"ENSG00000117569"
3155,"ENSG00000239665"
3156,"ENSG00000135519"
3157,"ENSG00000164096"
3158,"ENSG00000058453"
3159,"ENSG00000108433"
3160,"ENSG00000182264"
3161,"ENSG00000124198"
3162,"ENSG00000127580"
3163,"ENSG00000101624"
3164,"ENSG00000066427"
3165,"ENSG00000105705"
3166,"ENSG00000082641"
3167,"ENSG00000177455"
3168,"ENSG00000139636"

3169,"ENSG00000135114"
3170,"ENSG00000214113"
3171,"ENSG00000146574"
3172,"ENSG00000204851"
3173,"ENSG00000183549"
3174,"ENSG00000134802"
3175,"ENSG00000160208"
3176,"ENSG00000123219"
3177,"ENSG00000167580"
3178,"ENSG00000088930"
3179,"ENSG00000149179"
3180,"ENSG00000143418"
3181,"ENSG00000033327"
3182,"ENSG00000181991"
3183,"ENSG00000134779"
3184,"ENSG00000204257"
3185,"ENSG00000129282"
3186,"ENSG00000016391"
3187,"ENSG00000157168"
3188,"ENSG00000182600"
3189,"ENSG00000173486"
3190,"ENSG00000183571"
3191,"ENSG00000162267"
3192,"ENSG00000177098"
3193,"ENSG00000114209"
3194,"ENSG00000161929"
3195,"ENSG00000180210"
3196,"ENSG00000108344"
3197,"ENSG00000187049"
3198,"ENSG00000005249"
3199,"ENSG00000185947"
3200,"ENSG00000140675"
3201,"ENSG00000185019"
3202,"ENSG00000073803"
3203,"ENSG00000180209"
3204,"ENSG00000138175"
3205,"ENSG00000211448"
3206,"ENSG00000170365"
3207,"ENSG00000182179"
3208,"ENSG00000156050"
3209,"ENSG00000164736"
3210,"ENSG00000170473"
3211,"ENSG00000197312"
3212,"ENSG00000140497"
3213,"ENSG00000071967"
3214,"ENSG00000129932"
3215,"ENSG00000167977"
3216,"ENSG00000137218"

3217,"ENSG00000118418"
3218,"ENSG00000148357"
3219,"ENSG00000113658"
3220,"ENSG00000090487"
3221,"ENSG00000204463"
3222,"ENSG00000204314"
3223,"ENSG00000163584"
3224,"ENSG00000114127"
3225,"ENSG00000172878"
3226,"ENSG00000106615"
3227,"ENSG00000171858"
3228,"ENSG00000122687"
3229,"ENSG00000120896"
3230,"ENSG00000068976"
3231,"ENSG00000103269"
3232,"ENSG00000100226"
3233,"ENSG00000101052"
3234,"ENSG00000119227"
3235,"ENSG00000256515"
3236,"ENSG00000204525"
3237,"ENSG00000127081"
3238,"ENSG00000013306"
3239,"ENSG00000148834"
3240,"ENSG00000139531"
3241,"ENSG00000168890"
3242,"ENSG00000162522"
3243,"ENSG00000148200"
3244,"FAM22A"
3245,"ENSG00000239887"
3246,"ENSG00000143486"
3247,"ENSG00000149582"
3248,"ENSG00000138138"
3249,"ENSG00000156466"
3250,"ENSG00000204248"
3251,"ENSG00000136238"
3252,"ENSG00000198746"
3253,"ENSG00000180739"
3254,"ENSG00000110721"
3255,"ENSG00000255284"
3256,"ENSG00000177752"
3257,"ENSG00000092931"
3258,"ENSG00000138078"
3259,"ENSG00000153291"
3260,"ENSG00000151838"
3261,"ENSG00000073711"
3262,"ENSG00000170961"
3263,"ENSG00000130305"
3264,"ENSG00000204022"

3265,"ENSG00000110042"
3266,"ENSG00000152520"
3267,"ENSG00000167183"
3268,"ENSG00000115523"
3269,"ENSG00000175895"
3270,"ENSG00000134444"
3271,"ENSG00000100767"
3272,"ENSG00000186867"
3273,"ENSG00000171557"
3274,"ENSG00000169762"
3275,"ENSG00000110880"
3276,"ENSG00000099260"
3277,"ENSG00000242574"
3278,"ENSG00000174586"
3279,"ENSG00000204653"
3280,"ENSG00000197619"
3281,"ENSG00000150048"
3282,"ENSG00000109452"
3283,"ENSG00000110871"
3284,"ENSG00000126562"
3285,"ENSG00000065268"
3286,"ENSG00000049860"
3287,"ENSG00000205356"
3288,"ENSG00000103671"
3289,"ENSG00000099308"
3290,"ENSG00000146070"
3291,"ENSG00000249459"
3292,"ENSG00000139835"
3293,"ENSG00000143889"
3294,"ENSG00000068120"
3295,"ENSG00000114978"
3296,"ENSG00000055955"
3297,"ENSG00000175344"
3298,"ENSG00000132824"
3299,"ENSG00000135679"
3300,"ENSG00000158623"
3301,"ENSG00000176974"
3302,"ENSG00000104783"
3303,"ENSG00000179476"
3304,"ENSG00000133106"
3305,"ENSG00000141497"
3306,"ENSG00000120948"
3307,"ENSG00000111249"
3308,"ENSG00000186185"
3309,"ENSG00000162601"
3310,"ENSG00000170175"
3311,"ENSG00000171282"
3312,"ENSG00000078369"

3313,"ENSG00000109911"
3314,"ENSG00000158104"
3315,"ENSG00000129354"
3316,"ENSG00000174672"
3317,"ENSG00000100393"
3318,"ENSG00000164941"
3319,"ENSG00000117407"
3320,"ENSG00000147481"
3321,"ENSG00000167785"
3322,"ENSG00000002726"
3323,"ENSG00000151148"
3324,"ENSG00000163702"
3325,"ENSG00000164713"
3326,"ENSG00000172766"
3327,"ENSG00000140941"
3328,"ENSG00000149639"
3329,"ENSG00000108395"
3330,"ENSG00000178202"
3331,"ENSG00000189292"
3332,"ENSG00000070061"
3333,"ENSG00000120539"
3334,"ENSG00000124074"
3335,"ENSG00000100023"
3336,"ENSG00000167792"
3337,"ENSG00000125827"
3338,"ENSG00000188883"
3339,"ENSG00000036257"
3340,"ENSG00000114796"
3341,"ENSG00000157895"
3342,"ENSG00000253958"
3343,"ENSG00000151150"
3344,"ENSG00000152433"
3345,"ENSG00000184047"
3346,"ENSG00000165646"
3347,"ENSG00000162493"
3348,"ENSG00000189367"
3349,"ENSG00000198920"
3350,"ENSG00000174780"
3351,"ENSG00000143028"
3352,"ENSG00000186063"
3353,"ENSG00000078070"
3354,"ENSG00000136247"
3355,"ENSG00000185666"
3356,"ENSG00000100372"
3357,"ENSG00000156171"
3358,"ENSG00000048707"
3359,"ENSG00000267795"
3360,"ENSG00000135972"

3361,"ENSG00000172345"
3362,"ENSG00000100711"
3363,"ENSG00000160294"
3364,"ENSG00000189067"
3365,"ENSG00000153714"
3366,"ENSG00000149489"
3367,"ENSG00000070388"
3368,"ENSG00000188997"
3369,"ENSG00000267796"
3370,"ENSG00000163191"
3371,"ENSG00000167377"
3372,"ENSG00000127418"
3373,"ENSG00000171346"
3374,"ENSG00000106052"
3375,"ENSG00000119900"
3376,"ENSG00000214013"
3377,"ENSG00000114544"
3378,"ENSG00000117691"
3379,"ENSG00000213949"
3380,"ENSG00000139977"
3381,"ENSG00000157315"
3382,"ENSG00000115762"
3383,"ENSG00000101639"
3384,"ENSG00000181649"
3385,"ENSG00000184983"
3386,"ENSG00000267680"
3387,"ENSG00000105656"
3388,"ENSG00000158525"
3389,"C1orf187"
3390,"ENSG00000111785"
3391,"ENSG00000188152"
3392,"ENSG00000130707"
3393,"ENSG00000171530"
3394,"ENSG00000083444"
3395,"ENSG00000196653"
3396,"ENSG00000179979"
3397,"ENSG00000166828"
3398,"ENSG00000166086"
3399,"ENSG00000157992"
3400,"ENSG00000187474"
3401,"ENSG00000187554"
3402,"ENSG00000105825"
3403,"ENSG00000167632"
3404,"ENSG00000154328"
3405,"ENSG00000108557"
3406,"ENSG00000130702"
3407,"ENSG00000134265"
3408,"ENSG00000079337"

3409,"ENSG00000254997"
3410,"ENSG00000132153"
3411,"ENSG00000172775"
3412,"ENSG00000148985"
3413,"ENSG00000165115"
3414,"ENSG00000197930"
3415,"ENSG00000139722"
3416,"ENSG00000111364"
3417,"ENSG00000138587"
3418,"ENSG00000157353"
3419,"ENSG00000100387"
3420,"ENSG00000119865"
3421,"ENSG00000114026"
3422,"ENSG00000149115"
3423,"ENSG00000169962"
3424,"ENSG00000123610"
3425,"ENSG00000077235"
3426,"ENSG00000062370"
3427,"ENSG00000185298"
3428,"ENSG00000080166"
3429,"ENSG00000168329"
3430,"ENSG00000148484"
3431,"ENSG00000137441"
3432,"ENSG00000101546"
3433,"ENSG00000164485"
3434,"ENSG00000164074"
3435,"ENSG00000170017"
3436,"ENSG00000146072"
3437,"ENSG00000157014"
3438,"ENSG00000159111"
3439,"ENSG00000100583"
3440,"ENSG00000169925"
3441,"ENSG00000133808"
3442,"ENSG00000197857"
3443,"ENSG00000154146"
3444,"ENSG00000054148"
3445,"ENSG00000163803"
3446,"ENSG00000112852"
3447,"ENSG00000137944"
3448,"ENSG00000172932"
3449,"ENSG00000158488"
3450,"ENSG00000122786"
3451,"ENSG00000183891"
3452,"ENSG00000185404"
3453,"ENSG00000164902"
3454,"ENSG00000197457"
3455,"ENSG00000010318"
3456,"ENSG00000111339"

3457,"ENSG00000168256"
3458,"ENSG00000139921"
3459,"ENSG00000214290"
3460,"ENSG00000151778"
3461,"ENSG00000125534"
3462,"ENSG00000134007"
3463,"ENSG00000135002"
3464,"ENSG00000124882"
3465,"ENSG00000187672"
3466,"ENSG00000137474"
3467,"ENSG00000104442"
3468,"ENSG00000119943"
3469,"ENSG00000187091"
3470,"ENSG00000160551"
3471,"ENSG00000235109"
3472,"ENSG00000139517"
3473,"ENSG00000204186"
3474,"ENSG00000176340"
3475,"ENSG00000213722"
3476,"ENSG00000169994"
3477,"ENSG00000172167"
3478,"ENSG00000155729"
3479,"ENSG00000187492"
3480,"ENSG00000171735"
3481,"ENSG00000120137"
3482,"ENSG00000189227"
3483,"ENSG00000198858"
3484,"ENSG00000206562"
3485,"ENSG00000226524"
3486,"C1orf201"
3487,"ENSG00000131470"
3488,"ENSG00000075790"
3489,"ENSG00000112308"
3490,"ENSG00000116819"
3491,"ENSG00000168653"
3492,"ENSG00000108423"
3493,"ENSG00000221931"
3494,"ENSG00000111077"
3495,"ENSG00000039139"
3496,"ENSG00000125611"
3497,"ENSG00000110446"
3498,"ENSG00000109832"
3499,"ENSG00000087274"
3500,"ENSG00000083937"
3501,"ENSG00000157869"
3502,"ENSG00000137817"
3503,"ENSG00000101574"
3504,"ENSG00000139220"

3505,"ENSG00000184451"
3506,"ENSG00000166016"
3507,"ENSG00000112584"
3508,"ENSG00000128645"
3509,"ENSG00000145390"
3510,"ENSG00000139173"
3511,"ENSG00000102796"
3512,"ENSG00000161955"
3513,"ENSG00000167720"
3514,"ENSG00000144120"
3515,"ENSG00000167740"
3516,"ENSG00000172795"
3517,"ENSG00000119285"
3518,"ENSG00000115594"
3519,"ENSG00000206530"
3520,"ENSG00000177409"
3521,"ENSG00000155368"
3522,"ENSG00000049167"
3523,"ENSG00000105671"
3524,"ENSG00000123485"
3525,"ENSG00000167264"
3526,"ENSG00000111796"
3527,"ENSG00000065413"
3528,"ENSG00000147459"
3529,"ENSG00000151553"
3530,"ENSG00000184117"
3531,"ENSG00000148488"
3532,"ENSG00000242802"
3533,"ENSG00000121680"
3534,"ENSG00000142166"
3535,"ENSG00000178934"
3536,"ENSG00000103423"
3537,"ENSG00000183207"
3538,"ENSG00000127952"
3539,"ENSG00000135093"
3540,"ENSG00000090382"
3541,"ENSG00000100055"
3542,"ENSG00000100079"
3543,"ENSG00000134014"
3544,"ENSG00000124177"
3545,"ENSG00000095777"
3546,"ENSG00000161573"
3547,"ENSG00000204642"
3548,"ENSG00000121236"
3549,"ENSG00000182934"
3550,"ENSG00000157445"
3551,"ENSG00000152580"
3552,"ENSG00000146278"

3553,"ENSG00000162695"
3554,"ENSG00000119632"
3555,"ENSG00000221926"
3556,"ENSG00000096433"
3557,"ENSG00000123552"
3558,"ENSG00000112218"
3559,"ENSG00000092036"
3560,"ENSG00000173988"
3561,"ENSG00000130487"
3562,"ENSG00000177989"
3563,"ENSG00000088386"
3564,"ENSG00000145217"
3565,"ENSG00000135870"
3566,"ENSG00000144401"
3567,"ENSG00000165895"
3568,"ENSG00000141556"
3569,"ENSG00000188603"
3570,"ENSG00000079246"
3571,"ENSG00000162994"
3572,"ENSG00000043591"
3573,"ENSG00000172785"
3574,"ENSG00000104899"
3575,"ENSG00000175104"
3576,"ENSG00000169230"
3577,"ENSG00000188001"
3578,"ENSG00000100395"
3579,"ENSG00000095015"
3580,"ENSG00000153774"
3581,"ENSG00000204970"
3582,"ENSG00000064419"
3583,"ENSG00000144908"
3584,"ENSG00000116133"
3585,"ENSG00000124786"
3586,"ENSG00000104883"
3587,"ENSG00000152683"
3588,"ENSG00000142657"
3589,"ENSG00000162521"
3590,"ENSG00000160213"
3591,"ENSG00000160767"
3592,"ENSG00000148572"
3593,"ENSG00000163545"
3594,"ENSG00000092820"
3595,"ENSG00000152127"
3596,"ENSG00000064042"
3597,"ENSG00000093072"
3598,"ENSG00000198929"
3599,"ENSG00000139372"
3600,"ENSG00000108561"

3601,"ENSG00000117791"
3602,"ENSG00000106524"
3603,"ENSG00000164692"
3604,"ENSG00000105327"
3605,"ENSG00000106608"
3606,"ENSG00000176919"
3607,"ENSG00000160999"
3608,"ENSG00000186510"
3609,"ENSG00000130300"
3610,"ENSG00000131495"
3611,"ENSG00000139292"
3612,"ENSG00000268324"
3613,"ENSG00000178057"
3614,"ENSG00000089876"
3615,"ENSG00000148841"
3616,"ENSG00000260456"
3617,"ENSG00000204315"
3618,"ENSG00000164051"
3619,"ENSG00000185909"
3620,"ENSG00000198668"
3621,"ENSG00000019485"
3622,"ENSG00000214694"
3623,"ENSG00000128581"
3624,"ENSG00000066248"
3625,"ENSG00000141295"
3626,"ENSG00000095539"
3627,"ENSG00000110436"
3628,"ENSG00000258839"
3629,"ENSG00000096093"
3630,"ENSG00000078902"
3631,"ENSG00000087191"
3632,"ENSG00000084092"
3633,"ENSG00000135723"
3634,"ENSG00000182670"
3635,"ENSG00000138439"
3636,"ENSG00000150471"
3637,"ENSG00000145321"
3638,"ENSG00000100221"
3639,"ENSG00000184178"
3640,"ENSG00000158106"
3641,"ENSG00000128463"
3642,"ENSG00000197566"
3643,"ENSG00000185274"
3644,"ENSG00000242612"
3645,"ENSG00000116852"
3646,"ENSG00000129347"
3647,"ENSG00000152078"
3648,"ENSG00000123685"

3649,"ENSG00000139626"
3650,"ENSG00000152092"
3651,"ENSG00000156026"
3652,"ENSG00000138639"
3653,"ENSG00000174899"
3654,"ENSG00000136286"
3655,"ENSG00000164465"
3656,"ENSG00000125901"
3657,"ENSG00000253598"
3658,"ENSG00000188086"
3659,"ENSG00000246705"
3660,"ENSG00000104728"
3661,"ENSG00000163347"
3662,"ENSG00000198464"
3663,"ENSG00000118257"
3664,"ENSG00000164916"
3665,"ENSG00000256530"
3666,"ENSG00000124657"
3667,"ENSG00000154229"
3668,"ENSG00000167646"
3669,"ENSG00000157734"
3670,"ENSG00000180871"
3671,"ENSG00000088205"
3672,"ENSG00000177666"
3673,"ENSG00000146648"
3674,"ENSG00000065518"
3675,"ENSG00000152439"
3676,"ENSG00000227471"
3677,"ENSG00000089737"
3678,"ENSG00000100731"
3679,"ENSG00000198099"
3680,"ENSG00000089847"
3681,"ENSG00000127472"
3682,"ENSG00000108784"
3683,"ENSG00000136999"
3684,"ENSG00000152969"
3685,"ENSG00000020922"
3686,"ENSG00000073670"
3687,"ENSG00000106635"
3688,"ENSG00000125868"
3689,"ENSG00000132554"
3690,"ENSG00000079387"
3691,"ENSG00000087494"
3692,"ENSG00000176393"
3693,"ENSG00000169877"
3694,"ENSG00000182752"
3695,"ENSG00000198208"
3696,"ENSG00000130699"

3697,"ENSG00000109794"
3698,"ENSG00000161016"
3699,"ENSG00000038219"
3700,"ENSG00000212719"
3701,"ENSG00000236699"
3702,"ENSG00000168116"
3703,"ENSG00000077009"
3704,"ENSG00000132185"
3705,"ENSG00000269396"
3706,"ENSG00000184845"
3707,"ENSG00000183077"
3708,"ENSG00000138653"
3709,"ENSG00000135956"
3710,"ENSG00000115275"
3711,"ENSG00000196963"
3712,"ENSG00000148840"
3713,"ENSG00000140598"
3714,"ENSG00000092529"
3715,"ENSG00000140545"
3716,"ENSG00000175137"
3717,"ENSG00000163605"
3718,"ENSG00000141560"
3719,"ENSG00000010932"
3720,"ENSG00000267855"
3721,"ENSG00000248905"
3722,"ENSG00000165810"
3723,"ENSG00000117597"
3724,"ENSG00000136146"
3725,"ENSG00000149499"
3726,"ENSG00000125648"
3727,"ENSG00000050130"
3728,"ENSG00000176273"
3729,"ENSG00000122435"
3730,"ENSG00000130023"
3731,"ENSG00000081870"
3732,"ENSG00000130649"
3733,"ENSG00000255468"
3734,"ENSG00000268040"
3735,"ENSG00000114631"
3736,"ENSG00000151229"
3737,"ENSG00000256771"
3738,"ENSG00000057663"
3739,"ENSG00000185475"
3740,"ENSG00000142207"
3741,"ENSG00000125457"
3742,"ENSG00000170606"
3743,"ENSG00000106460"
3744,"ENSG00000137073"

3745,"ENSG00000215067"
3746,"ENSG00000174607"
3747,"ENSG00000269858"
3748,"ENSG00000128578"
3749,"ENSG00000155100"
3750,"ENSG00000135097"
3751,"ENSG00000141756"
3752,"ENSG00000239264"
3753,"ENSG00000240038"
3754,"ENSG00000243896"
3755,"ENSG00000168209"
3756,"ENSG00000186603"
3757,"ENSG00000105875"
3758,"ENSG00000171045"
3759,"ENSG00000158201"
3760,"ENSG00000107771"
3761,"ENSG00000108296"
3762,"ENSG00000060709"
3763,"ENSG00000235878"
3764,"ENSG00000145743"
3765,"ENSG00000160961"
3766,"ENSG00000128849"
3767,"ENSG00000171928"
3768,"ENSG00000204387"
3769,"ENSG00000171119"
3770,"ENSG00000173218"
3771,"ENSG00000167244"
3772,"ENSG00000105722"
3773,"ENSG00000197714"
3774,"ENSG00000037897"
3775,"ENSG00000101134"
3776,"ENSG00000134489"
3777,"ENSG00000161551"
3778,"ENSG00000213085"
3779,"ENSG00000168268"
3780,"ENSG00000185015"
3781,"ENSG00000175581"
3782,"ENSG00000088538"
3783,"ENSG00000174799"
3784,"ENSG00000106688"
3785,"ENSG00000151500"
3786,"ENSG00000151093"
3787,"ENSG00000164002"
3788,"ENSG00000138095"
3789,"ENSG00000162819"
3790,"ENSG00000124570"
3791,"ENSG00000113595"
3792,"ENSG00000010818"

3793,"ENSG00000213402"
3794,"ENSG00000153898"
3795,"ENSG00000163472"
3796,"ENSG00000150627"
3797,"ENSG00000171222"
3798,"ENSG00000249109"
3799,"ENSG00000140403"
3800,"ENSG00000215712"
3801,"ENSG00000155085"
3802,"ENSG00000115459"
3803,"ENSG00000187735"
3804,"ENSG00000255098"
3805,"ENSG00000135698"
3806,"ENSG00000061455"
3807,"ENSG00000198569"
3808,"ENSG00000206503"
3809,"ENSG00000168884"
3810,"ENSG00000154743"
3811,"ENSG00000146112"
3812,"ENSG00000005194"
3813,"ENSG00000138772"
3814,"ENSG00000216937"
3815,"ENSG00000128482"
3816,"ENSG00000160799"
3817,"ENSG00000011376"
3818,"ENSG00000060642"
3819,"ENSG00000152104"
3820,"ENSG00000161940"
3821,"ENSG00000156414"
3822,"ENSG00000115085"
3823,"ENSG00000125818"
3824,"ENSG00000125378"
3825,"ENSG00000130638"
3826,"ENSG00000221988"
3827,"ENSG00000072832"
3828,"ENSG00000229833"
3829,"ENSG00000121073"
3830,"ENSG00000160801"
3831,"ENSG00000244752"
3832,"ENSG00000186908"
3833,"ENSG00000108599"
3834,"ENSG00000197448"
3835,"ENSG00000188807"
3836,"ENSG00000126453"
3837,"ENSG00000155621"
3838,"ENSG00000104290"
3839,"ENSG00000171804"
3840,"ENSG00000132323"

3841,"ENSG00000125885"
3842,"ENSG00000178209"
3843,"ENSG00000250565"
3844,"ENSG00000123096"
3845,"ENSG00000099284"
3846,"ENSG00000141994"
3847,"ENSG00000075711"
3848,"ENSG00000163933"
3849,"ENSG00000177294"
3850,"ENSG00000183665"
3851,"ENSG00000159055"
3852,"ENSG00000100949"
3853,"ENSG00000230510"
3854,"ENSG00000148735"
3855,"ENSG00000107338"
3856,"ENSG00000107611"
3857,"ENSG00000007923"
3858,"ENSG00000124203"
3859,"ENSG00000170315"
3860,"ENSG00000131236"
3861,"ENSG00000248485"
3862,"ENSG00000099330"
3863,"ENSG00000132432"
3864,"ENSG00000125735"
3865,"ENSG00000116138"
3866,"ENSG00000175283"
3867,"ENSG00000122375"
3868,"ENSG00000169862"
3869,"ENSG00000136872"
3870,"ENSG00000134516"
3871,"ENSG00000096872"
3872,"ENSG00000157103"
3873,"ENSG00000168491"
3874,"ENSG00000163634"
3875,"ENSG00000082153"
3876,"ENSG00000112276"
3877,"ENSG00000204531"
3878,"ENSG00000150093"
3879,"ENSG00000167107"
3880,"ENSG00000198722"
3881,"ENSG00000185565"
3882,"ENSG00000112419"
3883,"ENSG00000134107"
3884,"ENSG00000121552"
3885,"ENSG00000185453"
3886,"ENSG00000123843"
3887,"ENSG00000175471"
3888,"ENSG00000136542"

3889,"ENSG00000156642"
3890,"ENSG00000036054"
3891,"ENSG00000141316"
3892,"ENSG00000222033"
3893,"ENSG00000183844"
3894,"ENSG00000163993"
3895,"ENSG00000178896"
3896,"ENSG00000163975"
3897,"ENSG00000266258"
3898,"ENSG00000075914"
3899,"ENSG00000197937"
3900,"ENSG00000140400"
3901,"ENSG00000152234"
3902,"ENSG00000142089"
3903,"ENSG00000187866"
3904,"ENSG00000111537"
3905,"ENSG00000114686"
3906,"ENSG00000168395"
3907,"ENSG00000186952"
3908,"ENSG00000122512"
3909,"ENSG00000165219"
3910,"ENSG00000088035"
3911,"ENSG00000149488"
3912,"ENSG00000166938"
3913,"ENSG00000186074"
3914,"ENSG00000165494"
3915,"ENSG00000269190"
3916,"ENSG00000149948"
3917,"ENSG00000159733"
3918,"ENSG00000103549"
3919,"ENSG00000101150"
3920,"ENSG00000184220"
3921,"ENSG00000163746"
3922,"ENSG00000184887"
3923,"ENSG00000129003"
3924,"ENSG00000104472"
3925,"ENSG00000013725"
3926,"ENSG00000116299"
3927,"ENSG00000121775"
3928,"ENSG00000198752"
3929,"ENSG00000185359"
3930,"ENSG00000215012"
3931,"ENSG00000171115"
3932,"ENSG00000178150"
3933,"ENSG00000102910"
3934,"ENSG00000235034"
3935,"ENSG00000103335"
3936,"ENSG00000134222"

3937,"ENSG00000119979"
3938,"ENSG00000145113"
3939,"ENSG00000153982"
3940,"ENSG00000167136"
3941,"ENSG00000257365"
3942,"ENSG00000139131"
3943,"ENSG00000166192"
3944,"ENSG00000009413"
3945,"ENSG00000125629"
3946,"ENSG00000147439"
3947,"ENSG00000183092"
3948,"ENSG00000171862"
3949,"ENSG00000204178"
3950,"ENSG00000196326"
3951,"ENSG00000119729"
3952,"ENSG00000187775"
3953,"ENSG00000163686"
3954,"ENSG00000188659"
3955,"ENSG00000164896"
3956,"ENSG00000013441"
3957,"ENSG00000146243"
3958,"ENSG00000134061"
3959,"ENSG00000185189"
3960,"ENSG00000222038"
3961,"ENSG00000163257"
3962,"ENSG00000010361"
3963,"ENSG00000149547"
3964,"ENSG00000175600"
3965,"ENSG00000243989"
3966,"ENSG00000135124"
3967,"ENSG00000137558"
3968,"ENSG00000116062"
3969,"ENSG00000040531"
3970,"ENSG00000167081"
3971,"ENSG00000180398"
3972,"ENSG00000165006"
3973,"ENSG00000158109"
3974,"ENSG00000140471"
3975,"ENSG00000138769"
3976,"ENSG00000170291"
3977,"ENSG00000184898"
3978,"ENSG00000070031"
3979,"ENSG00000172671"
3980,"ENSG00000185340"
3981,"ENSG00000182901"
3982,"ENSG00000113441"
3983,"ENSG00000217455"
3984,"ENSG00000129566"

3985,"ENSG00000113758"
3986,"ENSG00000116761"
3987,"ENSG00000128016"
3988,"ENSG00000161692"
3989,"ENSG00000141150"
3990,"ENSG00000170464"
3991,"ENSG00000145687"
3992,"ENSG00000113205"
3993,"ENSG00000133265"
3994,"ENSG00000162004"
3995,"ENSG00000144036"
3996,"ENSG00000119684"
3997,"ENSG00000221923"
3998,"ENSG00000168297"
3999,"ENSG00000114626"
4000,"ENSG00000105447"
4001,"ENSG00000122585"
4002,"ENSG00000171298"
4003,"ENSG00000171097"
4004,"ENSG00000114850"
4005,"ENSG00000129925"
4006,"ENSG00000138115"
4007,"ENSG00000071242"
4008,"ENSG00000132003"
4009,"ENSG00000167653"
4010,"ENSG00000117601"
4011,"ENSG00000187773"
4012,"ENSG00000255587"
4013,"ENSG00000165280"
4014,"ENSG00000140521"
4015,"ENSG00000160111"
4016,"ENSG00000176472"
4017,"ENSG00000182093"
4018,"ENSG00000020633"
4019,"ENSG00000089692"
4020,"ENSG00000111728"
4021,"ENSG00000048649"
4022,"ENSG00000165699"
4023,"ENSG00000197568"
4024,"ENSG00000197915"
4025,"ENSG00000263002"
4026,"ENSG00000172818"
4027,"ENSG00000111877"
4028,"ENSG00000065000"
4029,"ENSG00000137449"
4030,"ENSG00000112685"
4031,"ENSG00000100399"
4032,"ENSG00000144597"

4033,"ENSG00000174307"
4034,"ENSG00000134864"
4035,"ENSG00000133488"
4036,"ENSG00000156689"
4037,"ENSG00000177239"
4038,"ENSG00000169258"
4039,"ENSG00000179921"
4040,"ENSG00000239605"
4041,"ENSG00000170836"
4042,"ENSG00000203711"
4043,"ENSG00000196151"
4044,"ENSG00000164142"
4045,"ENSG00000102805"
4046,"ENSG00000118939"
4047,"ENSG00000169504"
4048,"ENSG00000255874"
4049,"ENSG00000196177"
4050,"ENSG00000213922"
4051,"ENSG00000143771"
4052,"ENSG00000148935"
4053,"ENSG00000057252"
4054,"ENSG00000147434"
4055,"ENSG00000171984"
4056,"ENSG00000100298"
4057,"ENSG00000189241"
4058,"ENSG00000138347"
4059,"ENSG00000197360"
4060,"ENSG00000177156"
4061,"ENSG00000105136"
4062,"ENSG00000111245"
4063,"ENSG00000164512"
4064,"ENSG00000174567"
4065,"ENSG00000130598"
4066,"ENSG00000111276"
4067,"ENSG00000111664"
4068,"ENSG00000109606"
4069,"ENSG00000160200"
4070,"ENSG00000167695"
4071,"ENSG00000175356"
4072,"ENSG00000185721"
4073,"ENSG00000103249"
4074,"ENSG00000125772"
4075,"ENSG00000154721"
4076,"ENSG00000250506"
4077,"ENSG00000137463"
4078,"ENSG00000100151"
4079,"ENSG00000214193"
4080,"ENSG00000197880"

4081,"ENSG00000188958"
4082,"ENSG00000130770"
4083,"ENSG00000198931"
4084,"ENSG00000188542"
4085,"ENSG00000127870"
4086,"ENSG00000091972"
4087,"ENSG00000170632"
4088,"ENSG00000130700"
4089,"ENSG00000167904"
4090,"ENSG00000259511"
4091,"ENSG00000164332"
4092,"ENSG00000172469"
4093,"ENSG00000174437"
4094,"ENSG00000099810"
4095,"ENSG00000170854"
4096,"ENSG00000143801"
4097,"ENSG00000153707"
4098,"ENSG00000234776"
4099,"ENSG00000165996"
4100,"ENSG00000072571"
4101,"ENSG00000167272"
4102,"ENSG00000100722"
4103,"ENSG00000078043"
4104,"ENSG00000006210"
4105,"ENSG00000196843"
4106,"ENSG00000182022"
4107,"ENSG00000176658"
4108,"ENSG00000170242"
4109,"ENSG00000094796"
4110,"ENSG00000176236"
4111,"ENSG00000179988"
4112,"ENSG00000197381"
4113,"ENSG00000122507"
4114,"ENSG00000187522"
4115,"ENSG00000107719"
4116,"ENSG00000161618"
4117,"ENSG00000120699"
4118,"ENSG00000157765"
4119,"ENSG00000149150"
4120,"ENSG00000196407"
4121,"ENSG00000100036"
4122,"ENSG00000231925"
4123,"ENSG00000170374"
4124,"ENSG00000178935"
4125,"ENSG00000231500"
4126,"ENSG00000163794"
4127,"ENSG00000162511"
4128,"ENSG00000103356"

4129,"ENSG00000163293"
4130,"ENSG00000099917"
4131,"ENSG00000254415"
4132,"ENSG00000138185"
4133,"ENSG00000157927"
4134,"ENSG00000178562"
4135,"ENSG00000161921"
4136,"ENSG00000183715"
4137,"ENSG00000198478"
4138,"ENSG00000090432"
4139,"ENSG00000163362"
4140,"ENSG00000179344"
4141,"ENSG00000170727"
4142,"ENSG00000181220"
4143,"ENSG00000232859"
4144,"ENSG00000129696"
4145,"ENSG00000197580"
4146,"ENSG00000102996"
4147,"ENSG00000227051"
4148,"ENSG00000170465"
4149,"ENSG00000112877"
4150,"ENSG00000111206"
4151,"ENSG00000198563"
4152,"ENSG00000115112"
4153,"ENSG00000172426"
4154,"ENSG00000158467"
4155,"ENSG00000152894"
4156,"ENSG00000259803"
4157,"ENSG00000205116"
4158,"ENSG00000040199"
4159,"ENSG00000172339"
4160,"ENSG00000185518"
4161,"ENSG00000163435"
4162,"ENSG00000106211"
4163,"ENSG00000112232"
4164,"ENSG00000013374"
4165,"ENSG00000130956"
4166,"ENSG00000241553"
4167,"ENSG00000143297"
4168,"ENSG00000187796"
4169,"ENSG00000083099"
4170,"ENSG00000130159"
4171,"ENSG00000122483"
4172,"ENSG00000090376"
4173,"ENSG00000136877"
4174,"ENSG00000141012"
4175,"ENSG00000196260"
4176,"ENSG00000127325"

4177,"ENSG00000172680"
4178,"ENSG00000128513"
4179,"ENSG00000187514"
4180,"ENSG00000162413"
4181,"ENSG00000136237"
4182,"ENSG00000050748"
4183,"ENSG00000172478"
4184,"ENSG00000134146"
4185,"ENSG00000136371"
4186,"ENSG00000099821"
4187,"ENSG00000107863"
4188,"ENSG00000104979"
4189,"ENSG00000174106"
4190,"ENSG00000139697"
4191,"ENSG00000180855"
4192,"ENSG00000133030"
4193,"ENSG00000131669"
4194,"ENSG00000140832"
4195,"ENSG00000173153"
4196,"ENSG00000177542"
4197,"ENSG00000204120"
4198,"ENSG00000124243"
4199,"ENSG00000213927"
4200,"ENSG00000205212"
4201,"ENSG00000183569"
4202,"ENSG00000187957"
4203,"ENSG00000244057"
4204,"ENSG00000131730"
4205,"ENSG00000148426"
4206,"ENSG00000198646"
4207,"ENSG00000111361"
4208,"ENSG00000181085"
4209,"ENSG00000183421"
4210,"ENSG00000104889"
4211,"ENSG00000185966"
4212,"ENSG00000099377"
4213,"ENSG00000144810"
4214,"ENSG00000196839"
4215,"ENSG00000140943"
4216,"ENSG00000162676"
4217,"ENSG00000188803"
4218,"ENSG00000147799"
4219,"ENSG00000197903"
4220,"ENSG00000107249"
4221,"ENSG00000114480"
4222,"ENSG00000118096"
4223,"ENSG00000203668"
4224,"ENSG00000262621"

4225,"ENSG00000197782"
4226,"ENSG00000155254"
4227,"ENSG00000157881"
4228,"ENSG00000196565"
4229,"ENSG00000187650"
4230,"ENSG00000117228"
4231,"ENSG00000176715"
4232,"ENSG00000121440"
4233,"ENSG00000182885"
4234,"ENSG00000170791"
4235,"ENSG00000126705"
4236,"ENSG00000118194"
4237,"ENSG00000257184"
4238,"ENSG00000108947"
4239,"ENSG00000015532"
4240,"ENSG00000138399"
4241,"ENSG00000140506"
4242,"ENSG00000018189"
4243,"ENSG00000197181"
4244,"ENSG00000128714"
4245,"ENSG00000169885"
4246,"ENSG00000170906"
4247,"ENSG00000162383"
4248,"ENSG00000144354"
4249,"ENSG00000125861"
4250,"ENSG00000142173"
4251,"ENSG00000135968"
4252,"ENSG00000087085"
4253,"ENSG00000183323"
4254,"ENSG00000117139"
4255,"ENSG00000151715"
4256,"ENSG00000128245"
4257,"ENSG00000116783"
4258,"ENSG00000127252"
4259,"ENSG00000134262"
4260,"ENSG00000206560"
4261,"ENSG00000124839"
4262,"ENSG00000143641"
4263,"ENSG00000150782"
4264,"ENSG00000163866"
4265,"ENSG00000168827"
4266,"ENSG00000178445"
4267,"ENSG00000134287"
4268,"ENSG00000120645"
4269,"ENSG00000249115"
4270,"ENSG00000151458"
4271,"ENSG00000160345"
4272,"ENSG00000176371"

4273,"ENSG00000074695"
4274,"ENSG00000104915"
4275,"ENSG00000104687"
4276,"ENSG00000183060"
4277,"ENSG00000141485"
4278,"ENSG00000244482"
4279,"ENSG00000112964"
4280,"ENSG00000165392"
4281,"ENSG00000008083"
4282,"ENSG00000088832"
4283,"ENSG00000047365"
4284,"ENSG00000164609"
4285,"ENSG00000105058"
4286,"ENSG00000240184"
4287,"ENSG00000164897"
4288,"ENSG00000166402"
4289,"ENSG00000157538"
4290,"ENSG00000141934"
4291,"ENSG00000196966"
4292,"ENSG00000075651"
4293,"ENSG00000140955"
4294,"ENSG00000250673"
4295,"ENSG00000149218"
4296,"ENSG00000113889"
4297,"ENSG00000160298"
4298,"ENSG00000138670"
4299,"ENSG00000130024"
4300,"ENSG00000178074"
4301,"ENSG00000112234"
4302,"ENSG00000099994"
4303,"ENSG00000257923"
4304,"ENSG00000111215"
4305,"ENSG00000135144"
4306,"ENSG00000161040"
4307,"ENSG00000151338"
4308,"ENSG00000111897"
4309,"ENSG00000198818"
4310,"ENSG00000170369"
4311,"ENSG00000141429"
4312,"ENSG00000157613"
4313,"ENSG00000214711"
4314,"ENSG00000136205"
4315,"ENSG00000124209"
4316,"ENSG00000089693"
4317,"ENSG00000123243"
4318,"ENSG00000134884"
4319,"ENSG00000134324"
4320,"ENSG00000245680"

4321,"ENSG00000187045"
4322,"ENSG00000204099"
4323,"ENSG00000070182"
4324,"ENSG00000152404"
4325,"ENSG00000144677"
4326,"ENSG00000149196"
4327,"ENSG00000049759"
4328,"ENSG00000133392"
4329,"ENSG00000110675"
4330,"ENSG00000124107"
4331,"ENSG00000007372"
4332,"ENSG00000090674"
4333,"ENSG00000134897"
4334,"ENSG00000161677"
4335,"ENSG00000240204"
4336,"ENSG00000174446"
4337,"ENSG00000137968"
4338,"ENSG00000015520"
4339,"ENSG00000169972"
4340,"ENSG00000111052"
4341,"ENSG00000141506"
4342,"ENSG00000160194"
4343,"ENSG00000171812"
4344,"ENSG00000104970"
4345,"ENSG00000205021"
4346,"ENSG00000152467"
4347,"ENSG00000165495"
4348,"ENSG00000178665"
4349,"ENSG00000197927"
4350,"ENSG00000111961"
4351,"ENSG00000198513"
4352,"ENSG00000119431"
4353,"ENSG00000153208"
4354,"ENSG00000162433"
4355,"ENSG00000178685"
4356,"ENSG00000206077"
4357,"ENSG00000185442"
4358,"ENSG00000084628"
4359,"ENSG00000196975"
4360,"ENSG00000109063"
4361,"ENSG00000100365"
4362,"ENSG00000130669"
4363,"ENSG00000114956"
4364,"ENSG00000102468"
4365,"ENSG00000114812"
4366,"ENSG00000107551"
4367,"ENSG00000163945"
4368,"ENSG00000086102"

4369,"ENSG00000129244"
4370,"ENSG00000119048"
4371,"ENSG00000108443"
4372,"ENSG00000178031"
4373,"ENSG00000167644"
4374,"ENSG00000151092"
4375,"ENSG00000122477"
4376,"ENSG00000173480"
4377,"ENSG00000178093"
4378,"ENSG00000106733"
4379,"ENSG00000069011"
4380,"ENSG00000158483"
4381,"ENSG00000170266"
4382,"ENSG00000102780"
4383,"ENSG00000124588"
4384,"ENSG00000125877"
4385,"ENSG00000197681"
4386,"ENSG00000185250"
4387,"ENSG00000011454"
4388,"ENSG00000008710"
4389,"ENSG00000198218"
4390,"ENSG00000007866"
4391,"ENSG00000186501"
4392,"ENSG00000121361"
4393,"ENSG00000153944"
4394,"ENSG00000259363"
4395,"ENSG00000128218"
4396,"ENSG00000146476"
4397,"ENSG00000025708"
4398,"ENSG00000157578"
4399,"ENSG00000144485"
4400,"ENSG00000137207"
4401,"ENSG00000167604"
4402,"ENSG00000107130"
4403,"ENSG00000130881"
4404,"ENSG00000100099"
4405,"ENSG00000139044"
4406,"ENSG00000166024"
4407,"ENSG00000149177"
4408,"ENSG00000168447"
4409,"ENSG00000183090"
4410,"ENSG00000020256"
4411,"ENSG00000111644"
4412,"ENSG00000143158"
4413,"ENSG00000236320"
4414,"ENSG00000105048"
4415,"ENSG00000179057"
4416,"ENSG00000033800"

4417,"ENSG00000178636"
4418,"ENSG00000134900"
4419,"ENSG00000087152"
4420,"ENSG00000170310"
4421,"ENSG00000158234"
4422,"ENSG00000163755"
4423,"ENSG00000110060"
4424,"ENSG00000128573"
4425,"ENSG00000196584"
4426,"ENSG00000125821"
4427,"ENSG00000038274"
4428,"ENSG00000095587"
4429,"ENSG00000131051"
4430,"ENSG00000228278"
4431,"ENSG00000168936"
4432,"ENSG00000129317"
4433,"ENSG00000164733"
4434,"ENSG00000018236"
4435,"ENSG00000163219"
4436,"ENSG00000129654"
4437,"ENSG00000176490"
4438,"ENSG00000088726"
4439,"ENSG00000114491"
4440,"ENSG00000237190"
4441,"ENSG00000263264"
4442,"ENSG00000156886"
4443,"ENSG00000189376"
4444,"ENSG00000159450"
4445,"ENSG00000057468"
4446,"ENSG00000123374"
4447,"ENSG00000127483"
4448,"ENSG00000106415"
4449,"ENSG00000159445"
4450,"ENSG00000039560"
4451,"ENSG00000006453"
4452,"ENSG00000100439"
4453,"ENSG00000198915"
4454,"ENSG00000162670"
4455,"ENSG00000122692"
4456,"ENSG00000163486"
4457,"ENSG00000212670"
4458,"ENSG00000109684"
4459,"ENSG00000167881"
4460,"ENSG00000162086"
4461,"ENSG00000161634"
4462,"ENSG00000076706"
4463,"ENSG00000198874"
4464,"ENSG00000158457"

4465,"ENSG00000182575"
4466,"ENSG00000165688"
4467,"ENSG00000165695"
4468,"ENSG00000166847"
4469,"ENSG00000196152"
4470,"ENSG00000145536"
4471,"ENSG00000131849"
4472,"ENSG00000185483"
4473,"ENSG00000153786"
4474,"ENSG00000141668"
4475,"ENSG00000171860"
4476,"ENSG00000144395"
4477,"ENSG00000139218"
4478,"ENSG00000176125"
4479,"ENSG00000177234"
4480,"ENSG00000170802"
4481,"ENSG00000114739"
4482,"ENSG00000179111"
4483,"ENSG00000102531"
4484,"ENSG00000178826"
4485,"ENSG00000104221"
4486,"ENSG00000177694"
4487,"ENSG00000163960"
4488,"ENSG00000148057"
4489,"ENSG00000145919"
4490,"ENSG00000106100"
4491,"ENSG00000086589"
4492,"ENSG00000167733"
4493,"ENSG00000240654"
4494,"ENSG00000107731"
4495,"ENSG00000170525"
4496,"ENSG00000174194"
4497,"ENSG00000171243"
4498,"ENSG00000162641"
4499,"ENSG00000154310"
4500,"ENSG00000060749"
4501,"ENSG00000110315"
4502,"ENSG00000156502"
4503,"ENSG00000102452"
4504,"ENSG00000166188"
4505,"ENSG00000259495"
4506,"ENSG00000159399"
4507,"ENSG00000095209"
4508,"ENSG00000170866"
4509,"ENSG00000155545"
4510,"ENSG00000224877"
4511,"ENSG00000168944"
4512,"ENSG00000160783"

4513,"ENSG00000100219"
4514,"ENSG00000181135"
4515,"ENSG00000163046"
4516,"ENSG00000185420"
4517,"ENSG00000072415"
4518,"ENSG00000120800"
4519,"ENSG00000136867"
4520,"ENSG00000105321"
4521,"ENSG00000103319"
4522,"ENSG00000144043"
4523,"ENSG00000168348"
4524,"ENSG00000099984"
4525,"ENSG00000136457"
4526,"ENSG00000154258"
4527,"ENSG00000198331"
4528,"ENSG00000170786"
4529,"ENSG00000090238"
4530,"ENSG00000143842"
4531,"ENSG00000149021"
4532,"ENSG00000158815"
4533,"ENSG00000167554"
4534,"ENSG00000160323"
4535,"ENSG00000104611"
4536,"ENSG00000163606"
4537,"ENSG00000101265"
4538,"ENSG00000068078"
4539,"ENSG00000183111"
4540,"ENSG00000105887"
4541,"ENSG00000168765"
4542,"ENSG00000007255"
4543,"ENSG00000214413"
4544,"ENSG00000170989"
4545,"ENSG00000143514"
4546,"ENSG00000092020"
4547,"ENSG00000099256"
4548,"ENSG00000177519"
4549,"ENSG00000196335"
4550,"ENSG00000077150"
4551,"ENSG00000132518"
4552,"ENSG00000172215"
4553,"ENSG00000269343"
4554,"ENSG00000068781"
4555,"ENSG00000138759"
4556,"ENSG00000160714"
4557,"ENSG00000103222"
4558,"ENSG00000107736"
4559,"ENSG00000197409"
4560,"ENSG00000033050"

4561,"ENSG00000124635"
4562,"ENSG00000214226"
4563,"ENSG00000183742"
4564,"ENSG00000126860"
4565,"ENSG00000113812"
4566,"ENSG00000120860"
4567,"ENSG00000180875"
4568,"ENSG00000140632"
4569,"ENSG00000186567"
4570,"ENSG00000117154"
4571,"ENSG00000180357"
4572,"ENSG00000166526"
4573,"ENSG00000204655"
4574,"ENSG00000112299"
4575,"ENSG00000258875"
4576,"ENSG00000165678"
4577,"ENSG00000243749"
4578,"ENSG00000155324"
4579,"ENSG00000138463"
4580,"ENSG00000061918"
4581,"ENSG00000101222"
4582,"ENSG00000206172"
4583,"ENSG00000162482"
4584,"ENSG00000160932"
4585,"ENSG00000167601"
4586,"ENSG00000160049"
4587,"ENSG00000243335"
4588,"ENSG00000108262"
4589,"ENSG00000115109"
4590,"ENSG00000105609"
4591,"ENSG00000183775"
4592,"ENSG00000171853"
4593,"ENSG00000176697"
4594,"ENSG00000074370"
4595,"ENSG00000185155"
4596,"ENSG00000118961"
4597,"ENSG00000158428"
4598,"ENSG00000204316"
4599,"ENSG00000203797"
4600,"ENSG00000196118"
4601,"ENSG00000112130"
4602,"ENSG00000135338"
4603,"ENSG00000215277"
4604,"ENSG00000159648"
4605,"ENSG00000197056"
4606,"ENSG00000116205"
4607,"ENSG00000101752"
4608,"ENSG00000197860"

4609,"ENSG00000069431"
4610,"ENSG00000183873"
4611,"ENSG00000127311"
4612,"ENSG00000172572"
4613,"ENSG00000166507"
4614,"ENSG00000132170"
4615,"ENSG00000070476"
4616,"ENSG00000170627"
4617,"ENSG00000185758"
4618,"ENSG00000214338"
4619,"ENSG00000100299"
4620,"ENSG00000172551"
4621,"ENSG00000163468"
4622,"ENSG00000182853"
4623,"ENSG00000173077"
4624,"ENSG00000130810"
4625,"ENSG00000187902"
4626,"ENSG00000166676"
4627,"ENSG00000176018"
4628,"ENSG00000196323"
4629,"ENSG00000174282"
4630,"ENSG00000090372"
4631,"ENSG00000180332"
4632,"ENSG00000160185"
4633,"ENSG00000136002"
4634,"ENSG00000254852"
4635,"ENSG00000184445"
4636,"ENSG00000177963"
4637,"ENSG00000110057"
4638,"ENSG00000128274"
4639,"ENSG00000149503"
4640,"ENSG00000013288"
4641,"ENSG00000188368"
4642,"ENSG00000178252"
4643,"ENSG00000244242"
4644,"ENSG00000095261"
4645,"ENSG00000205268"
4646,"ENSG00000166535"
4647,"ENSG00000171777"
4648,"ENSG00000173809"
4649,"ENSG00000107362"
4650,"ENSG00000162231"
4651,"ENSG00000107537"
4652,"ENSG00000174886"
4653,"ENSG00000162572"
4654,"ENSG00000172890"
4655,"ENSG00000138650"
4656,"ENSG00000119013"

4657,"ENSG00000116922"
4658,"ENSG00000150556"
4659,"ENSG00000186532"
4660,"ENSG00000059377"
4661,"ENSG00000124571"
4662,"ENSG00000065154"
4663,"ENSG00000090920"
4664,"ENSG00000246223"
4665,"ENSG00000155380"
4666,"ENSG00000171094"
4667,"ENSG00000081791"
4668,"ENSG00000214106"
4669,"ENSG00000184838"
4670,"ENSG00000174837"
4671,"ENSG00000140307"
4672,"ENSG00000163110"
4673,"ENSG00000165685"
4674,"ENSG00000095951"
4675,"ENSG00000124019"
4676,"ENSG00000084207"
4677,"ENSG00000121454"
4678,"ENSG00000108984"
4679,"ENSG00000115504"
4680,"ENSG00000023892"
4681,"ENSG00000150656"
4682,"ENSG00000205309"
4683,"ENSG00000123191"
4684,"ENSG00000087008"
4685,"ENSG00000157426"
4686,"ENSG00000132434"
4687,"ENSG00000134108"
4688,"ENSG00000169340"
4689,"ENSG00000170485"
4690,"ENSG00000166398"
4691,"ENSG00000006071"
4692,"ENSG00000197822"
4693,"ENSG00000129103"
4694,"ENSG00000114867"
4695,"ENSG00000167641"
4696,"ENSG00000132359"
4697,"ENSG00000184271"
4698,"ENSG00000105726"
4699,"ENSG00000146414"
4700,"ENSG00000187024"
4701,"ENSG00000137310"
4702,"ENSG00000140488"
4703,"ENSG00000182263"
4704,"ENSG00000170234"

4705,"ENSG00000130844"
4706,"ENSG00000024862"
4707,"ENSG00000198535"
4708,"ENSG00000169908"
4709,"ENSG00000135046"
4710,"ENSG00000197043"
4711,"ENSG00000198730"
4712,"ENSG00000169413"
4713,"ENSG00000121289"
4714,"ENSG00000163576"
4715,"ENSG00000165406"
4716,"ENSG00000158604"
4717,"ENSG00000160087"
4718,"ENSG00000067533"
4719,"ENSG00000102935"
4720,"ENSG00000154655"
4721,"ENSG00000113282"
4722,"ENSG00000205060"
4723,"ENSG00000168306"
4724,"ENSG00000074319"
4725,"ENSG00000090661"
4726,"ENSG00000167193"
4727,"ENSG00000189350"
4728,"ENSG00000171843"
4729,"ENSG00000243678"
4730,"ENSG00000158869"
4731,"ENSG00000269305"
4732,"ENSG00000139899"
4733,"ENSG00000171533"
4734,"ENSG00000151364"
4735,"ENSG00000075336"
4736,"ENSG00000241852"
4737,"ENSG00000182533"
4738,"ENSG00000140853"
4739,"ENSG00000115665"
4740,"ENSG00000174788"
4741,"ENSG00000205853"
4742,"ENSG00000221953"
4743,"ENSG00000139197"
4744,"ENSG00000138162"
4745,"ENSG00000187398"
4746,"ENSG00000059573"
4747,"ENSG00000180869"
4748,"ENSG00000198574"
4749,"ENSG00000026508"
4750,"ENSG00000166272"
4751,"ENSG00000156587"
4752,"ENSG00000178585"

4753,"ENSG00000162669"
4754,"ENSG00000116793"
4755,"ENSG00000089094"
4756,"ENSG00000107938"
4757,"ENSG00000160229"
4758,"ENSG00000159346"
4759,"ENSG00000120688"
4760,"ENSG00000122952"
4761,"ENSG00000106153"
4762,"ENSG00000156858"
4763,"ENSG00000175229"
4764,"ENSG00000018699"
4765,"ENSG00000159189"
4766,"ENSG00000120254"
4767,"ENSG00000139496"
4768,"ENSG00000177728"
4769,"ENSG00000168495"
4770,"ENSG00000197771"
4771,"ENSG00000138829"
4772,"ENSG00000113389"
4773,"ENSG00000205937"
4774,"ENSG00000196511"
4775,"ENSG00000184949"
4776,"ENSG00000178951"
4777,"ENSG00000128989"
4778,"ENSG00000157107"
4779,"ENSG00000104722"
4780,"ENSG00000177272"
4781,"ENSG00000146530"
4782,"ENSG00000154479"
4783,"ENSG00000177084"
4784,"ENSG00000101439"
4785,"ENSG00000176714"
4786,"ENSG00000204365"
4787,"ENSG00000157916"
4788,"ENSG00000173706"
4789,"ENSG00000166888"
4790,"ENSG00000181541"
4791,"ENSG00000174903"
4792,"ENSG00000156795"
4793,"ENSG00000113303"
4794,"ENSG00000164032"
4795,"ENSG00000142192"
4796,"ENSG00000205045"
4797,"ENSG00000204308"
4798,"ENSG00000189046"
4799,"ENSG00000140006"
4800,"ENSG00000135414"

4801,"ENSG00000184402"
4802,"ENSG00000099957"
4803,"ENSG00000163239"
4804,"ENSG00000108883"
4805,"ENSG00000233927"
4806,"ENSG00000122367"
4807,"ENSG00000213585"
4808,"ENSG00000137076"
4809,"ENSG00000215915"
4810,"ENSG00000060069"
4811,"ENSG00000253731"
4812,"ENSG00000213614"
4813,"ENSG00000059728"
4814,"ENSG00000183208"
4815,"ENSG00000108551"
4816,"ENSG00000250120"
4817,"ENSG00000160255"
4818,"ENSG00000112796"
4819,"ENSG00000153561"
4820,"ENSG00000213123"
4821,"ENSG00000140575"
4822,"ENSG00000174080"
4823,"ENSG00000173088"
4824,"ENSG00000033178"
4825,"ENSG00000181234"
4826,"ENSG00000148110"
4827,"ENSG00000101224"
4828,"ENSG00000198003"
4829,"ENSG00000136141"
4830,"ENSG00000120334"
4831,"ENSG00000173065"
4832,"ENSG00000149295"
4833,"ENSG00000162643"
4834,"ENSG00000106049"
4835,"ENSG00000184635"
4836,"ENSG00000125245"
4837,"ENSG00000110002"
4838,"ENSG00000253831"
4839,"ENSG00000119715"
4840,"ENSG00000166268"
4841,"ENSG00000108950"
4842,"ENSG00000113296"
4843,"ENSG00000142444"
4844,"ENSG00000155052"
4845,"ENSG00000148082"
4846,"ENSG00000110958"
4847,"ENSG00000174482"
4848,"ENSG00000108424"

4849,"ENSG00000127920"
4850,"ENSG00000179454"
4851,"ENSG00000164649"
4852,"ENSG00000120910"
4853,"ENSG00000088682"
4854,"ENSG00000272047"
4855,"ENSG00000186283"
4856,"ENSG00000105287"
4857,"ENSG00000156011"
4858,"ENSG00000105464"
4859,"ENSG00000178026"
4860,"ENSG00000154447"
4861,"ENSG00000175183"
4862,"ENSG00000176890"
4863,"ENSG00000132958"
4864,"ENSG00000170631"
4865,"ENSG00000159713"
4866,"ENSG00000181585"
4867,"ENSG00000165555"
4868,"ENSG00000104951"
4869,"ENSG00000196372"
4870,"ENSG00000101003"
4871,"ENSG00000007264"
4872,"ENSG00000123815"
4873,"ENSG00000103415"
4874,"ENSG00000100601"
4875,"ENSG00000143420"
4876,"ENSG00000184985"
4877,"ENSG00000165355"
4878,"ENSG00000168994"
4879,"ENSG00000241962"
4880,"ENSG00000128891"
4881,"ENSG00000091583"
4882,"ENSG00000182463"
4883,"ENSG00000163964"
4884,"ENSG00000108264"
4885,"ENSG00000198053"
4886,"ENSG00000181031"
4887,"ENSG00000185896"
4888,"ENSG00000168661"
4889,"ENSG00000166822"
4890,"ENSG00000115641"
4891,"ENSG00000213160"
4892,"ENSG00000198732"
4893,"ENSG00000143751"
4894,"ENSG00000079150"
4895,"ENSG00000064225"
4896,"ENSG00000188800"

4897,"ENSG00000095485"
4898,"ENSG00000151176"
4899,"ENSG00000176978"
4900,"ENSG00000213390"
4901,"ENSG00000041353"
4902,"ENSG00000100038"
4903,"ENSG00000106554"
4904,"ENSG00000132604"
4905,"ENSG00000120088"
4906,"ENSG00000106536"
4907,"ENSG00000198885"
4908,"ENSG00000078140"
4909,"ENSG00000167384"
4910,"ENSG00000198369"
4911,"ENSG00000168594"
4912,"ENSG00000127249"
4913,"ENSG00000133710"
4914,"ENSG00000128294"
4915,"ENSG00000161542"
4916,"ENSG00000197385"
4917,"ENSG00000152492"
4918,"ENSG00000222018"
4919,"ENSG00000136527"
4920,"ENSG00000135821"
4921,"ENSG00000173272"
4922,"ENSG00000132640"
4923,"ENSG00000112874"
4924,"ENSG00000148154"
4925,"ENSG00000155744"
4926,"ENSG00000242852"
4927,"ENSG00000146373"
4928,"ENSG00000178015"
4929,"ENSG00000122729"
4930,"ENSG00000198900"
4931,"ENSG00000010030"
4932,"ENSG00000103226"
4933,"ENSG00000090924"
4934,"ENSG00000154781"
4935,"ENSG00000006468"
4936,"ENSG00000124788"
4937,"ENSG00000112149"
4938,"ENSG00000126804"
4939,"ENSG00000185024"
4940,"ENSG00000139180"
4941,"ENSG00000140470"
4942,"ENSG00000076201"
4943,"ENSG00000179299"
4944,"ENSG00000120235"

4945,"ENSG00000013364"
4946,"ENSG00000140326"
4947,"ENSG00000138134"
4948,"ENSG00000112280"
4949,"ENSG00000132031"
4950,"ENSG00000215271"
4951,"ENSG00000170390"
4952,"ENSG00000100425"
4953,"ENSG00000196405"
4954,"ENSG00000184277"
4955,"ENSG00000198721"
4956,"ENSG00000145216"
4957,"ENSG00000181788"
4958,"ENSG00000153395"
4959,"ENSG00000164398"
4960,"ENSG00000166558"
4961,"ENSG00000107443"
4962,"ENSG00000188549"
4963,"ENSG00000178602"
4964,"ENSG00000213064"
4965,"ENSG00000122490"
4966,"ENSG00000255062"
4967,"ENSG00000122678"
4968,"ENSG00000187961"
4969,"ENSG00000108932"
4970,"ENSG00000117411"
4971,"ENSG00000122971"
4972,"ENSG00000118402"
4973,"ENSG00000084070"
4974,"ENSG00000184361"
4975,"ENSG00000129151"
4976,"ENSG00000172137"
4977,"ENSG00000116199"
4978,"ENSG00000162869"
4979,"ENSG00000112293"
4980,"ENSG00000179774"
4981,"ENSG00000145107"
4982,"ENSG00000073756"
4983,"ENSG00000162714"
4984,"ENSG00000204446"
4985,"ENSG00000153721"
4986,"ENSG00000156234"
4987,"ENSG00000112062"
4988,"ENSG00000108061"
4989,"ENSG00000106823"
4990,"ENSG00000004660"
4991,"ENSG00000166415"
4992,"ENSG00000163618"

4993,"ENSG00000154813"
4994,"ENSG00000164252"
4995,"ENSG00000111639"
4996,"ENSG00000125775"
4997,"ENSG00000159640"
4998,"ENSG00000035681"
4999,"ENSG00000137502"
5000,"ENSG00000174197"
5001,"ENSG00000105619"
5002,"ENSG00000157379"
5003,"ENSG00000120159"
5004,"ENSG00000130311"
5005,"ENSG00000197520"
5006,"ENSG00000156521"
5007,"ENSG00000142677"
5008,"ENSG00000249437"
5009,"ENSG00000135272"
5010,"ENSG00000148400"
5011,"ENSG00000152726"
5012,"ENSG00000183023"
5013,"OVCA2"
5014,"ENSG00000242419"
5015,"ENSG00000185104"
5016,"ENSG00000197879"
5017,"ENSG00000088899"
5018,"ENSG00000213341"
5019,"ENSG00000175198"
5020,"ENSG00000180509"
5021,"WDR85"
5022,"ENSG00000253276"
5023,"ENSG00000162852"
5024,"ENSG00000166685"
5025,"ENSG00000019582"
5026,"ENSG00000068654"
5027,"ENSG00000204740"
5028,"ENSG00000138675"
5029,"ENSG00000011566"
5030,"ENSG00000222014"
5031,"ENSG00000185650"
5032,"ENSG00000161640"
5033,"ENSG00000186628"
5034,"ENSG00000150672"
5035,"ENSG00000133193"
5036,"ENSG00000068745"
5037,"ENSG00000137976"
5038,"ENSG00000154222"
5039,"ENSG00000260300"
5040,"ENSG00000158864"

5041,"ENSG00000173085"
5042,"ENSG00000143228"
5043,"ENSG00000142784"
5044,"ENSG00000203813"
5045,"ENSG00000152990"
5046,"ENSG00000059915"
5047,"ENSG00000214253"
5048,"ENSG00000143321"
5049,"ENSG00000162981"
5050,"ENSG00000165283"
5051,"ENSG00000131504"
5052,"ENSG00000109775"
5053,"ENSG00000100316"
5054,"ENSG00000091947"
5055,"ENSG00000131910"
5056,"ENSG00000137693"
5057,"ENSG00000172059"
5058,"ENSG00000111846"
5059,"ENSG00000111057"
5060,"ENSG00000025800"
5061,"ENSG00000118246"
5062,"ENSG00000131153"
5063,"ENSG00000125520"
5064,"ENSG00000213096"
5065,"ENSG00000080824"
5066,"ENSG00000150456"
5067,"ENSG00000173457"
5068,"ENSG00000164024"
5069,"ENSG00000187944"
5070,"ENSG00000117174"
5071,"ENSG00000136444"
5072,"ENSG00000213516"
5073,"ENSG00000221909"
5074,"ENSG00000162148"
5075,"ENSG00000136352"
5076,"ENSG00000206549"
5077,"ENSG00000086289"
5078,"ENSG00000165733"
5079,"ENSG00000135476"
5080,"ENSG00000126106"
5081,"ENSG00000141627"
5082,"ENSG00000178338"
5083,"ENSG00000214435"
5084,"ENSG00000115718"
5085,"ENSG00000102572"
5086,"ENSG00000172967"
5087,"ENSG00000140553"
5088,"ENSG00000150316"

5089,"ENSG00000177984"
5090,"ENSG00000116857"
5091,"ENSG00000163162"
5092,"ENSG00000170890"
5093,"ENSG00000131944"
5094,"ENSG00000203877"
5095,"ENSG00000103064"
5096,"ENSG00000122033"
5097,"ENSG00000187848"
5098,"ENSG00000137331"
5099,"ENSG00000185591"
5100,"ENSG00000162877"
5101,"ENSG00000198836"
5102,"ENSG00000138468"
5103,"ENSG00000112855"
5104,"ENSG00000132749"
5105,"ENSG00000266714"
5106,"ENSG00000152315"
5107,"ENSG00000182986"
5108,"ENSG00000166436"
5109,"ENSG00000030110"
5110,"ENSG00000078687"
5111,"ENSG00000219607"
5112,"ENSG00000110075"
5113,"ENSG00000179796"
5114,"ENSG00000198046"
5115,"ENSG00000149716"
5116,"ENSG00000143013"
5117,"ENSG00000127366"
5118,"ENSG00000135845"
5119,"ENSG00000177646"
5120,"ENSG00000169752"
5121,"ENSG00000204963"
5122,"ENSG00000101336"
5123,"ENSG00000119919"
5124,"ENSG00000186818"
5125,"ENSG00000133067"
5126,"ENSG00000179008"
5127,"ENSG00000100504"
5128,"ENSG00000205693"
5129,"ENSG00000106771"
5130,"ENSG00000129187"
5131,"ENSG00000100368"
5132,"ENSG00000136122"
5133,"ENSG00000134452"
5134,"ENSG00000034152"
5135,"ENSG00000179841"
5136,"ENSG00000153914"

5137,"ENSG00000117091"
5138,"ENSG00000004866"
5139,"ENSG00000110455"
5140,"ENSG00000166477"
5141,"ENSG00000154734"
5142,"ENSG00000165533"
5143,"ENSG00000153904"
5144,"ENSG00000204366"
5145,"ENSG00000152705"
5146,"ENSG00000177076"
5147,"ENSG00000123427"
5148,"ENSG00000121318"
5149,"ENSG00000143776"
5150,"ENSG00000254531"
5151,"ENSG00000173660"
5152,"ENSG00000159871"
5153,"ENSG00000131238"
5154,"ENSG00000138109"
5155,"ENSG00000011405"
5156,"ENSG00000100714"
5157,"ENSG00000113916"
5158,"ENSG00000116983"
5159,"ENSG00000093010"
5160,"ENSG00000175899"
5161,"ENSG00000167765"
5162,"ENSG00000166326"
5163,"ENSG00000003147"
5164,"ENSG00000205517"
5165,"ENSG00000172809"
5166,"ENSG00000116141"
5167,"ENSG00000127528"
5168,"ENSG00000154764"
5169,"ENSG00000152503"
5170,"ENSG00000100726"
5171,"ENSG00000151702"
5172,"ENSG00000130545"
5173,"ENSG00000166295"
5174,"ENSG00000134698"
5175,"ENSG00000143314"
5176,"ENSG00000065361"
5177,"ENSG00000070669"
5178,"ENSG00000164197"
5179,"ENSG00000112242"
5180,"ENSG00000227124"
5181,"ENSG00000172432"
5182,"ENSG00000138686"
5183,"ENSG00000109445"
5184,"ENSG00000233276"

5185,"ENSG00000198742"
5186,"ENSG00000108854"
5187,"ENSG00000165655"
5188,"ENSG00000100416"
5189,"ENSG00000084636"
5190,"ENSG00000012983"
5191,"ENSG00000047617"
5192,"ENSG00000255748"
5193,"ENSG00000166548"
5194,"ENSG00000178467"
5195,"ENSG00000226180"
5196,"ENSG00000176473"
5197,"ENSG00000156381"
5198,"ENSG00000166546"
5199,"ENSG00000167671"
5200,"ENSG00000180938"
5201,"ENSG00000164754"
5202,"ENSG00000198105"
5203,"ENSG00000241697"
5204,"ENSG00000137818"
5205,"ENSG00000162928"
5206,"ENSG00000130787"
5207,"ENSG00000169826"
5208,"ENSG00000197808"
5209,"ENSG00000150540"
5210,"ENSG00000132694"
5211,"ENSG00000135549"
5212,"ENSG00000120451"

1,"ENSG00000162704"
2,"ENSG00000255298"
3,"ENSG00000186814"
4,"ENSG00000101197"
5,"ENSG00000251247"
6,"ENSG00000166359"
7,"ENSG00000163684"
8,"ENSG00000084090"
9,"ENSG00000135702"
10,"ENSG00000122873"
11,"ENSG00000134996"
12,"ENSG00000162614"
13,"ENSG00000155660"
14,"ENSG00000162998"
15,"ENSG00000111850"
16,"ENSG00000125510"
17,"ENSG00000166341"
18,"ENSG00000213221"
19,"ENSG00000108381"
20,"ENSG00000188282"
21,"ENSG00000065665"
22,"ENSG00000186150"
23,"ENSG00000073331"
24,"ENSG00000182511"
25,"ENSG00000132016"
26,"ENSG00000065457"
27,"ENSG00000133466"
28,"ENSG00000054219"
29,"ENSG00000182054"
30,"ENSG00000241058"
31,"ENSG00000153814"
32,"ENSG00000111142"
33,"ENSG00000112799"
34,"ENSG00000203880"
35,"ENSG00000064545"
36,"ENSG00000133313"
37,"ENSG00000151883"
38,"ENSG00000168778"
39,"ENSG00000092094"
40,"ENSG00000185324"
41,"ENSG00000178802"
42,"ENSG00000157823"
43,"ENSG00000124508"
44,"ENSG00000179115"
45,"ENSG00000114770"
46,"ENSG00000182606"
47,"ENSG00000168291"
48,"ENSG00000146540"

49,"ENSG00000213903"
50,"ENSG00000196867"
51,"ENSG00000152240"
52,"ENSG00000154096"
53,"ENSG00000127903"
54,"ENSG00000144214"
55,"ENSG00000075975"
56,"ENSG00000172348"
57,"ENSG00000137106"
58,"ENSG00000242715"
59,"ENSG00000186832"
60,"ENSG00000107959"
61,"ENSG00000167525"
62,"ENSG00000171476"
63,"ENSG00000136982"
64,"ENSG00000089063"
65,"ENSG00000104324"
66,"ENSG00000179218"
67,"ENSG00000178750"
68,"ENSG00000154719"
69,"ENSG00000243566"
70,"ENSG00000132563"
71,"ENSG00000156603"
72,"ENSG00000100994"
73,"ENSG00000118058"
74,"ENSG00000128607"
75,"ENSG00000139187"
76,"ENSG00000130758"
77,"ENSG00000133103"
78,"ENSG00000079974"
79,"ENSG00000076924"
80,"ENSG00000133983"
81,"ENSG00000170776"
82,"ENSG00000113734"
83,"ENSG00000150403"
84,"ENSG00000173548"
85,"ENSG00000164924"
86,"ENSG00000119669"
87,"ENSG00000104450"
88,"ENSG00000128791"
89,"ENSG00000166510"
90,"ENSG00000163938"
91,"ENSG00000187987"
92,"ENSG00000115750"
93,"ENSG00000163155"
94,"ENSG00000165832"
95,"ENSG00000167562"
96,"ENSG00000136319"

97,"ENSG00000103168"
98,"ENSG00000065060"
99,"ENSG00000130489"
100,"ENSG00000185619"
101,"ENSG00000100814"
102,"ENSG00000100065"
103,"ENSG00000144134"
104,"ENSG00000108666"
105,"ENSG00000015133"
106,"ENSG00000105298"
107,"ENSG00000007516"
108,"ENSG00000188687"
109,"ENSG00000228570"
110,"ENSG00000186642"
111,"ENSG00000108963"
112,"ENSG00000204428"
113,"ENSG00000105607"
114,"ENSG00000124532"
115,"ENSG00000009950"
116,"ENSG00000176681"
117,"ENSG00000168404"
118,"ENSG00000164944"
119,"ENSG00000100116"
120,"ENSG00000184602"
121,"ENSG00000161203"
122,"ENSG00000114446"
123,"ENSG00000109113"
124,"ENSG00000119326"
125,"ENSG00000175395"
126,"ENSG00000118004"
127,"ENSG00000126749"
128,"ENSG00000188493"
129,"ENSG00000125826"
130,"ENSG00000108511"
131,"ENSG00000176834"
132,"ENSG00000175003"
133,"ENSG00000113558"
134,"ENSG00000138050"
135,"ENSG00000137806"
136,"ENSG00000148832"
137,"ENSG00000176209"
138,"ENSG00000145029"
139,"ENSG00000155016"
140,"ENSG00000160953"
141,"ENSG00000145725"
142,"ENSG00000165806"
143,"ENSG00000080345"
144,"ENSG00000177311"

145,"ENSG00000124299"
146,"ENSG00000196821"
147,"ENSG00000184206"
148,"ENSG00000144230"
149,"ENSG00000163568"
150,"ENSG00000179698"
151,"ENSG00000196663"
152,"ENSG00000113068"
153,"ENSG00000163554"
154,"ENSG00000118507"
155,"ENSG00000242498"
156,"ENSG00000197586"
157,"ENSG00000093167"
158,"ENSG00000138600"
159,"ENSG00000132823"
160,"ENSG00000157150"
161,"ENSG00000110063"
162,"ENSG00000160321"
163,"ENSG00000173145"
164,"ENSG00000152223"
165,"ENSG00000077238"
166,"ENSG00000124678"
167,"ENSG00000113621"
168,"ENSG00000114670"
169,"ENSG00000185838"
170,"ENSG00000129480"
171,"ENSG00000162366"
172,"ENSG00000104714"
173,"ENSG00000145439"
174,"ENSG00000151690"
175,"ENSG00000137275"
176,"ENSG00000181523"
177,"ENSG00000050327"
178,"ENSG00000134873"
179,"ENSG00000149743"
180,"ENSG00000170954"
181,"ENSG00000204843"
182,"ENSG00000171103"
183,"ENSG00000213853"
184,"ENSG00000133704"
185,"ENSG00000064309"
186,"ENSG00000169035"
187,"ENSG00000078269"
188,"ENSG00000105497"
189,"ENSG00000102580"
190,"ENSG00000072736"
191,"ENSG00000125144"
192,"ENSG00000133243"

193,"ENSG00000187824"
194,"ENSG00000164342"
195,"ENSG00000134962"
196,"ENSG00000124357"
197,"ENSG00000151893"
198,"ENSG00000108244"
199,"ENSG00000185880"
200,"ENSG00000119446"
201,"ENSG00000115310"
202,"ENSG00000175220"
203,"ENSG00000122643"
204,"ENSG00000154122"
205,"ENSG00000163331"
206,"ENSG00000188295"
207,"ENSG00000196460"
208,"ENSG00000197614"
209,"ENSG00000125912"
210,"ENSG00000122085"
211,"ENSG00000113946"
212,"ENSG00000214402"
213,"ENSG00000101158"
214,"ENSG00000078053"

1,"ENSG00000269051"
2,"ENSG00000229694"
3,"ENSG00000204709"
4,"ENSG00000263508"
5,"ENSG00000225127"
6,"ENSG00000205057"
7,"ENSG00000271390"
8,"ENSG00000214900"
9,"ENSG00000241106"
10,"ENSG00000120306"
11,"ENSG00000179397"
12,"ENSG00000229212"
13,"ENSG00000169116"
14,"ENSG00000272316"
15,"ENSG00000156162"
16,"ENSG00000172466"
17,"ENSG00000178075"
18,"ENSG00000204267"
19,"ENSG00000177483"
20,"ENSG00000258359"
21,"ENSG00000146109"
22,"ENSG00000100889"
23,"ENSG00000235641"
24,"ENSG00000170571"
25,"ENSG00000076554"
26,"ENSG00000225706"
27,"ENSG00000116701"
28,"ENSG00000100554"
29,"ENSG00000180773"
30,"ENSG00000267838"
31,"ENSG00000154127"
32,"ENSG00000116260"
33,"ENSG00000174325"
34,"ENSG00000185339"
35,"ENSG00000213906"
36,"ENSG00000177946"
37,"ENSG00000105894"
38,"ENSG00000071564"
39,"ENSG00000181555"
40,"ENSG00000176635"
41,"ENSG00000152487"
42,"ENSG00000265139"
43,"ENSG00000254333"
44,"ENSG00000166913"
45,"ENSG00000269001"
46,"ENSG00000176101"
47,"ENSG00000242220"
48,"ENSG00000170260"

49,"ENSG00000257315"
50,"ENSG00000188738"
51,"ENSG00000197948"
52,"ENSG00000174004"
53,"ENSG00000197768"
54,"ENSG00000141255"
55,"ENSG00000061936"
56,"ENSG00000161281"
57,"ENSG00000239650"
58,"ENSG00000267013"
59,"ENSG00000004468"
60,"ENSG00000229205"
61,"ENSG00000129467"
62,"ENSG00000108064"
63,"ENSG00000272541"
64,"ENSG00000272091"
65,"ENSG00000230147"
66,"ENSG00000223561"
67,"ENSG00000234232"
68,"ENSG00000170561"
69,"ENSG00000165752"
70,"ENSG00000204348"
71,"ENSG00000262583"
72,"ENSG00000213985"
73,"ENSG00000143315"
74,"ENSG00000188868"
75,"ENSG00000184752"
76,"ENSG00000167972"
77,"ENSG00000102699"
78,"ENSG00000205084"
79,"ENSG00000091436"
80,"ENSG00000162729"
81,"ENSG00000162458"
82,"ENSG00000249601"
83,"ENSG00000187151"
84,"ENSG00000165478"
85,"ENSG00000177425"
86,"ENSG00000229891"
87,"ENSG00000265750"
88,"ENSG00000082068"
89,"ENSG00000100034"
90,"ENSG00000035687"
91,"ENSG00000169609"
92,"ENSG00000148719"
93,"ENSG00000260027"
94,"ENSG00000242110"
95,"ENSG00000204252"
96,"ENSG00000169228"

97,"ENSG00000227877"
98,"ENSG00000112992"
99,"ENSG00000170915"
100,"ENSG00000258405"
101,"ENSG00000104894"
102,"ENSG00000233473"
103,"ENSG00000104901"
104,"ENSG00000225329"
105,"ENSG00000205595"
106,"ENSG00000148482"
107,"ENSG00000166262"
108,"ENSG00000182472"
109,"ENSG00000197265"
110,"ENSG00000103148"
111,"ENSG00000257239"
112,"ENSG00000171056"
113,"ENSG00000215808"
114,"ENSG00000259205"
115,"ENSG00000226642"
116,"ENSG00000250427"
117,"ENSG00000247121"
118,"ENSG00000164068"
119,"ENSG00000260265"
120,"ENSG00000270237"
121,"ENSG00000010610"
122,"ENSG00000123143"
123,"ENSG00000251169"
124,"ENSG00000256612"
125,"ENSG00000265933"
126,"ENSG00000126785"
127,"ENSG00000258593"
128,"ENSG00000254901"
129,"ENSG00000158528"
130,"ENSG00000106443"
131,"ENSG00000161944"
132,"ENSG00000266173"
133,"ENSG00000150977"
134,"ENSG00000216901"
135,"ENSG00000188986"
136,"ENSG00000183486"
137,"ENSG00000220161"
138,"ENSG00000256667"
139,"ENSG00000264176"
140,"ENSG00000271584"
141,"ENSG00000146457"
142,"ENSG00000165091"
143,"ENSG00000228600"
144,"ENSG00000223508"

145,"ENSG00000224557"
146,"ENSG00000177025"
147,"ENSG00000197245"
148,"ENSG00000254667"
149,"ENSG00000134375"
150,"ENSG00000146386"
151,"ENSG00000251000"
152,"ENSG00000270409"
153,"ENSG00000251532"
154,"ENSG00000162604"
155,"ENSG00000198682"
156,"ENSG00000270127"
157,"ENSG00000175749"
158,"ENSG00000254731"
159,"ENSG00000177764"
160,"ENSG00000266935"
161,"ENSG00000111186"
162,"ENSG00000126500"
163,"ENSG00000131778"
164,"ENSG00000105270"
165,"ENSG00000243710"
166,"ENSG00000178761"
167,"ENSG00000089327"
168,"ENSG00000222009"
169,"ENSG00000038532"
170,"ENSG00000270093"
171,"ENSG00000234918"
172,"ENSG00000198860"
173,"ENSG00000164062"
174,"ENSG00000131941"
175,"ENSG00000162600"
176,"ENSG00000122870"
177,"ENSG00000176095"
178,"ENSG00000141577"
179,"ENSG00000063127"
180,"ENSG00000196922"
181,"ENSG00000223501"
182,"ENSG00000137185"
183,"ENSG00000081320"
184,"ENSG00000272275"
185,"ENSG00000163728"
186,"ENSG00000161180"
187,"ENSG00000106591"
188,"ENSG00000108813"
189,"ENSG00000167617"
190,"ENSG00000237595"
191,"ENSG00000270020"
192,"ENSG00000088888"

193,"ENSG00000273139"
194,"ENSG00000131401"
195,"ENSG00000204778"
196,"ENSG00000100523"
197,"ENSG00000226676"
198,"ENSG00000271581"
199,"ENSG00000167799"
200,"ENSG00000151151"
201,"ENSG00000155761"
202,"ENSG00000226015"
203,"ENSG00000164588"
204,"ENSG00000188599"
205,"ENSG00000232721"
206,"ENSG00000158805"
207,"ENSG00000180257"
208,"ENSG00000230487"
209,"ENSG00000272864"
210,"ENSG00000180385"
211,"ENSG00000079689"
212,"ENSG00000132600"
213,"ENSG00000091490"
214,"ENSG00000266648"
215,"ENSG00000161202"
216,"ENSG00000231742"
217,"ENSG00000188517"
218,"ENSG00000072163"
219,"ENSG00000272523"
220,"ENSG00000111300"
221,"ENSG00000148296"
222,"ENSG00000225490"
223,"ENSG00000187984"
224,"ENSG00000119698"
225,"ENSG00000204228"
226,"ENSG00000142230"
227,"ENSG00000173540"
228,"ENSG00000143434"
229,"ENSG00000008441"
230,"ENSG00000166407"
231,"ENSG00000150867"
232,"ENSG00000267939"
233,"ENSG00000213983"
234,"ENSG00000197062"
235,"ENSG00000197157"
236,"ENSG00000141337"
237,"ENSG00000162076"
238,"ENSG00000179776"
239,"ENSG00000269973"
240,"ENSG00000023445"

241,"ENSG00000260075"
242,"ENSG00000197279"
243,"ENSG00000250412"
244,"ENSG00000267088"
245,"ENSG00000271590"
246,"ENSG00000214826"
247,"ENSG00000157017"
248,"ENSG00000104129"
249,"ENSG00000224014"
250,"ENSG00000197951"
251,"ENSG00000271538"
252,"ENSG00000143811"
253,"ENSG00000272360"
254,"ENSG00000101198"
255,"ENSG00000231702"
256,"ENSG00000137714"
257,"ENSG00000251383"
258,"ENSG00000249258"
259,"ENSG00000137824"
260,"ENSG00000196961"
261,"ENSG00000237624"
262,"ENSG00000106610"
263,"ENSG00000156876"
264,"ENSG00000221823"
265,"ENSG00000115295"
266,"ENSG00000140320"
267,"ENSG00000260804"
268,"ENSG00000270547"
269,"ENSG00000063245"
270,"ENSG00000168995"
271,"ENSG00000140263"
272,"ENSG00000151692"
273,"ENSG00000170647"
274,"ENSG00000259630"
275,"ENSG00000254772"
276,"ENSG00000178538"
277,"ENSG00000140612"
278,"ENSG00000143921"
279,"ENSG00000197045"
280,"ENSG00000244480"
281,"ENSG00000110717"
282,"ENSG00000100156"
283,"ENSG00000226942"
284,"ENSG00000162545"
285,"ENSG00000173805"
286,"ENSG00000165684"
287,"ENSG00000120093"
288,"ENSG00000146909"

289,"ENSG00000133101"
290,"ENSG00000131697"
291,"ENSG00000267018"
292,"ENSG00000129455"
293,"ENSG00000105982"
294,"ENSG00000109929"
295,"ENSG00000226629"
296,"ENSG00000124875"
297,"ENSG00000076650"
298,"ENSG00000171700"
299,"ENSG00000272690"
300,"ENSG00000161609"
301,"ENSG00000188163"
302,"ENSG00000233916"
303,"ENSG00000103035"
304,"ENSG00000108219"
305,"ENSG00000170092"
306,"ENSG00000198093"
307,"ENSG00000175305"
308,"ENSG00000177432"
309,"ENSG00000244026"
310,"ENSG00000184207"
311,"ENSG00000234073"
312,"ENSG00000269552"
313,"ENSG00000087206"
314,"ENSG00000198919"
315,"ENSG00000138459"
316,"ENSG00000053108"
317,"ENSG00000272444"
318,"ENSG00000245468"
319,"ENSG00000078898"
320,"ENSG00000219435"
321,"ENSG00000269067"
322,"ENSG00000231952"
323,"ENSG00000182103"
324,"ENSG00000237419"
325,"ENSG00000244753"
326,"ENSG00000258231"
327,"ENSG00000182255"
328,"ENSG00000173214"
329,"ENSG00000104331"
330,"ENSG00000114904"
331,"ENSG00000164136"
332,"ENSG00000250317"
333,"ENSG00000178381"
334,"ENSG00000230611"
335,"ENSG00000196263"
336,"ENSG00000089169"

337,"ENSG00000254685"
338,"ENSG00000127957"
339,"ENSG00000219200"
340,"ENSG00000133597"
341,"ENSG00000126467"
342,"ENSG00000273033"
343,"ENSG00000137672"
344,"ENSG00000230772"
345,"ENSG00000033100"
346,"ENSG00000117090"
347,"ENSG00000260973"
348,"ENSG00000204767"
349,"ENSG00000145824"
350,"ENSG00000104522"
351,"ENSG00000112137"
352,"ENSG00000063438"
353,"ENSG00000163904"
354,"ENSG00000248587"
355,"ENSG00000198750"
356,"ENSG00000164889"
357,"ENSG00000072694"
358,"ENSG00000170689"
359,"ENSG00000171421"
360,"ENSG00000196199"
361,"ENSG00000204592"
362,"ENSG00000184115"
363,"ENSG00000137513"
364,"ENSG00000230305"
365,"ENSG00000111271"
366,"ENSG00000099251"
367,"ENSG00000129484"
368,"ENSG00000178397"
369,"ENSG00000100234"
370,"ENSG00000090054"
371,"ENSG00000226874"
372,"ENSG00000178425"
373,"ENSG00000182141"
374,"ENSG00000141639"
375,"ENSG00000196167"
376,"ENSG00000230373"
377,"ENSG00000204351"
378,"ENSG00000224132"
379,"ENSG00000143702"
380,"ENSG00000250778"
381,"ENSG00000185742"
382,"ENSG00000205583"
383,"ENSG00000260454"
384,"ENSG00000166825"

385,"ENSG00000180353"
386,"ENSG00000100129"
387,"ENSG00000188707"
388,"ENSG00000248290"
389,"ENSG00000272777"
390,"ENSG00000108799"
391,"ENSG00000203872"
392,"ENSG00000101306"
393,"ENSG00000088833"
394,"ENSG00000151665"
395,"ENSG00000113719"
396,"ENSG00000225422"
397,"ENSG00000148908"
398,"ENSG00000204920"
399,"ENSG00000227755"
400,"ENSG00000090857"
401,"ENSG00000247157"
402,"ENSG00000258429"
403,"ENSG00000260459"
404,"ENSG00000121406"
405,"ENSG00000225507"
406,"ENSG00000178917"
407,"ENSG00000058085"
408,"ENSG00000272902"
409,"ENSG00000132128"
410,"ENSG00000187634"
411,"ENSG00000185040"
412,"ENSG00000272130"
413,"ENSG00000259005"
414,"ENSG00000016602"
415,"ENSG00000156170"
416,"ENSG00000213453"
417,"ENSG00000114841"
418,"ENSG00000113119"
419,"ENSG00000227671"
420,"ENSG00000227558"
421,"ENSG00000106070"
422,"ENSG00000021762"
423,"ENSG00000272195"
424,"ENSG00000152601"
425,"ENSG00000180881"
426,"ENSG00000178226"
427,"ENSG00000170837"
428,"ENSG00000256673"
429,"ENSG00000160325"
430,"ENSG00000071243"
431,"ENSG00000182685"
432,"ENSG00000132406"

433,"ENSG00000172014"
434,"ENSG00000163737"
