# Supplementary material for: The transcriptome-wide association search for genes and genetic variants which associate with BMI and gestational weight gain in women with type 1 diabetes
Source: Mol Med. 2021 Jan 20;27:6. doi: 10.1186/s10020-020-00266-z (PMC7818927; doi:10.1186/s10020-020-00266-z)
Supplement: Supplementary file 5 — Additional file 5: Data S2. The subanalyses performed for genes in the overlap between GWG and BMI only. [file 10020_2020_266_MOESM5_ESM.pdf]

Supplementary Data 2

SubAnalysis 1

In analysis 1, 15 genes influenced both GWG and BMI. In the subAnalysis 1, we searched for variants within the 1Mb window centered around these 15 genes only. Among 2993 variants with MAF >5%, 204 were nominally associated with GWG. The list of those variants along with their p-values is presented below. Out of 19 variants presented in Table 2, three - rs11465293 (CCL24), rs1978202 (CCL26) and rs13340504 (CCL24) - were found in this subAnalysis 1 confined to genes in the overlap only. FUMA did not find any lead SNPs or GWAS Catalog variants.

SNP_ID	Est_G	Joint_p-value
rs11465293	4,15	0,000025
rs1978202	3,86	0,000045
rs1052607	1,40	0,002102
rs13340504	2,33	0,002737
rs17171904	0,95	0,002902
rs5756306	-1,75	0,003508
rs3133539	-0,15	0,003690
rs7519040	-1,07	0,004471
rs3093998	-0,31	0,004724
rs1267308	-1,04	0,004746
rs4276654	1,60	0,005191
rs4793870	-1,67	0,006512
rs1374498	-1,45	0,006998
rs885125	-1,47	0,008149
rs1752380	0,24	0,008446
rs6964720	-1,94	0,008660
rs2248372	-0,50	0,010873
rs16898906	-0,51	0,010991
rs9933029	1,93	0,011028
rs2844479	1,45	0,011217
rs2298265	0,90	0,011946
rs3748545	0,90	0,011946
rs1780586	0,90	0,011946
rs1752382	0,90	0,011946
rs6588397	-2,22	0,012133
rs239917	-1,78	0,013508
rs2894239	-0,73	0,014115
rs2894240	-0,73	0,014115
rs3115573	-0,73	0,014115
rs3130315	-0,73	0,014115
rs4793854	-1,44	0,014225

rs6429601	-1,14	0,014800
rs1264466	0,75	0,014865
rs8073963	-1,37	0,015271
rs1264471	0,70	0,015456
rs9904760	-1,30	0,015594
rs4793889	-1,30	0,015594
rs2248907	1,46	0,016132
rs4783619	1,78	0,016177
rs755714	1,46	0,016430
rs805297	1,46	0,016430
rs707919	1,46	0,016430
rs2476163	-1,33	0,016461
rs1868158	1,71	0,016758
rs9267956	-0,71	0,017261
rs1129152	0,90	0,017308
rs2516400	0,05	0,017658
rs4835796	1,60	0,018122
rs3798153	1,60	0,018122
rs2261033	-1,23	0,018164
rs3094014	-0,37	0,018244
rs4835646	1,09	0,018878
rs17703660	-1,23	0,018885
rs2515226	-1,03	0,019128
rs13340490	1,56	0,019552
rs7777461	2,62	0,019806
rs4793598	-1,04	0,019950
rs3107295	-1,33	0,020324
rs2769265	0,52	0,020493
rs7207109	-1,25	0,020669
rs10424953	-1,31	0,020881
rs2040862	1,76	0,020967
rs2516408	0,08	0,021082
rs3828903	0,45	0,021172
rs3828912	0,45	0,021172
rs3810291	-1,39	0,021683
rs2303487	-1,18	0,021704
rs3107293	-1,32	0,021754
rs10072318	1,96	0,021824
rs13361619	1,96	0,021824
rs10073922	1,96	0,021824
rs3737741	-1,06	0,021935
rs12939811	-1,11	0,021951
rs2890805	0,82	0,022630
rs7211412	-1,56	0,022873
rs7461991	0,72	0,023377
rs1042815	-1,39	0,023381
rs7186693	-1,11	0,023571

rs718079	-1,26	0,023736
rs2763979	-1,19	0,024310
rs1054072	-1,23	0,024481
rs2326017	-1,22	0,025099
rs2855812	0,33	0,025632
rs10280802	-1,98	0,025731
rs239960	-0,99	0,026415
rs6978214	2,61	0,026560
rs2844502	0,22	0,026745
rs2395488	0,22	0,026745
rs2243429	0,22	0,026745
rs2596536	0,22	0,026745
rs2248617	0,22	0,026745
rs1879110	-1,47	0,027014
rs4793980	-1,53	0,027047
rs2516424	0,20	0,027059
rs4728533	-0,92	0,027748
rs890435	-0,93	0,027824
rs1264478	-0,13	0,027845
rs1264477	-0,13	0,027845
rs2863978	1,67	0,028113
rs3803650	1,67	0,028113
rs2143462	-0,81	0,028770
rs2844475	1,05	0,029060
rs942249	-0,89	0,030272
rs2857697	1,04	0,030650
rs3018899	-1,89	0,030845
rs3096648	-0,84	0,030852
rs9651181	-1,22	0,030864
rs9267947	-1,06	0,031008
rs3757598	-1,08	0,031036
rs2736171	1,03	0,031164
rs3094228	-0,46	0,031245
rs9267431	0,08	0,031450
rs10504938	-0,10	0,031863
rs2273019	-0,96	0,033030
rs2863981	1,63	0,033521
rs1033500	-0,78	0,033756
rs2395114	-0,78	0,033770
rs9268132	-0,78	0,033770
rs4713518	-0,78	0,033770
rs537757	-0,78	0,033770
rs502626	-0,78	0,033770
rs547261	-0,78	0,033770
rs531094	-0,78	0,033770
rs485774	-0,78	0,033770
rs552339	-0,78	0,033770

rs533885	-0,78	0,033770
rs9368713	-0,78	0,033770
rs9405090	-0,78	0,033770
rs742582	-0,78	0,033770
rs10807100	-0,78	0,033770
rs6930681	-0,78	0,033770
rs3817982	-0,78	0,033770
rs2143465	-0,78	0,033770
rs4576282	-0,78	0,033770
rs4959096	-0,78	0,033770
rs2076538	-0,78	0,033770
rs9268326	-0,78	0,033770
rs9268368	-0,78	0,033770
rs2073046	-0,78	0,033770
rs9268384	-0,78	0,033770
rs1033498	-0,78	0,033844
rs10956928	-0,17	0,034298
rs3813321	1,53	0,034664
rs6987256	-0,13	0,034834
rs2395110	-0,68	0,034837
rs563412	-0,68	0,034862
rs3132935	-0,93	0,034965
rs740076	-1,50	0,035060
rs3020644	0,88	0,035445
rs7729723	1,53	0,035865
rs3096686	-1,01	0,037647
rs1264461	0,48	0,037782
rs2469652	-1,42	0,038435
rs2555110	-0,20	0,038918
rs454748	-0,56	0,038938
rs7830822	0,30	0,039365
rs206019	0,73	0,039675
rs206016	0,73	0,039675
rs206015	0,73	0,039675
rs660550	-0,96	0,039686
rs4451990	0,25	0,040148
rs660594	-0,94	0,040999
rs644827	-0,94	0,040999
rs644774	-0,94	0,040999
rs2242664	-0,94	0,040999
rs3115560	-1,04	0,041073
rs12951790	-1,59	0,042481
rs3132945	-1,01	0,042548
rs8056893	1,60	0,043245
rs2071286	0,47	0,043409
rs240067	-1,55	0,043691
rs9268055	-1,01	0,043898

rs3096673	-1,01	0,043898
rs3130340	-1,01	0,043898
rs3115553	-1,01	0,043898
rs3115552	-1,01	0,043898
rs7751896	-1,01	0,043898
rs6935269	-1,01	0,043898
rs3749966	-1,01	0,043898
rs6909427	-1,01	0,043898
rs3864302	-1,01	0,043898
rs204993	-0,62	0,043937
rs3096681	-1,00	0,044181
rs10890412	1,27	0,044566
rs240068	-1,59	0,045628
rs7835534	1,28	0,045639
rs464694	-0,15	0,046116
rs4734518	-0,50	0,046385
rs7840123	0,62	0,046504
rs3809773	-1,40	0,046562
rs7887	0,81	0,047248
rs13169864	-1,30	0,047895
rs4735307	0,48	0,047941
rs2252532	-1,02	0,048173
rs13184188	1,75	0,048255
rs9935025	1,53	0,048544
rs2844498	0,00	0,048776
rs6684085	0,46	0,049215
rs1141538	-1,29	0,049377
rs1125979	-1,29	0,049377
rs4835678	-1,48	0,049706
rs10040792	-1,48	0,049706
rs7554936	0,95	0,049751

SubAnalysis 2

An analogous subAnalysis 2, in which we searched for variants influencing GWG only in 135 genes (500 kb upstream and downstream) which showed an overlap in Analysis 2, yielded 29776 variants with MAF > 5%. Among them 1557 showed nominal association with GWG (presented below). Six variants – rs11465293 (CCL24), rs4796675 (LINC000974), rs1978202 (CCL26), rs8080053 (LINC000974), rs11714248 (TRANK1), rs11235519 (AP005019.4) - were found among 19 presented in Table 3. Three of these also associated with GWG in Analysis 1 and subAnalysis 1. FUMA prioritization showed that variants enriched the alternative pathway of fetal androgen synthesis and classical pathway of

steroidogenesis, with POR and MIR4651 overlapping in these pathways. Twelve variants were found in GWAS Catalog, however none were associated with metabolic phenotypes (10 variants with response to cognitive behavioral therapy in major depressive disorder and 3 with coffee consumption). Variants in two genes - ZP3 and POMZP3 - showed an association with acrosin binding in GO Molecular Functions. We also found that 33 variants were eQTLs for 21 genes. One lead SNP rs11465293 in CCL24 gene was found. It was the same variant which showed an association with GWG in Analysis 1, subAnalysis 1, Analysis 2 and subAnalysis 2. In GWAS Catalog it is associated with eosinophil count.

SNP_ID	Est_G	Joint_p-value
rs11465293	4,15	0,000025
rs1978202	3,86	0,000045
rs11714248	-1,36	0,000104
rs4796675	2,31	0,000176
rs8080053	2,17	0,000194
rs3127468	-2,36	0,000380
rs6132792	1,89	0,000386
rs4982312	-0,48	0,000392
rs2641805	-0,96	0,000423
rs2738808	-0,96	0,000423
rs1064416	-2,18	0,000480
rs6564267	-0,49	0,000514
rs16940689	-0,83	0,000520
rs2550886	-0,83	0,000576
rs2550887	-0,83	0,000576
rs2550911	-0,80	0,000618
rs10416524	-1,49	0,000643
rs7199936	-0,75	0,000665
rs3123711	2,20	0,000691
rs2641804	-0,78	0,000697
rs11233170	0,57	0,000824
rs28280	-1,80	0,000852
rs11235519	-2,27	0,000854
rs12060035	-3,42	0,000896
rs8046531	-1,16	0,000949
rs8046531	-1,16	0,000949
rs732428	-3,20	0,000984
rs6768108	-1,26	0,001005
rs3132550	-0,20	0,001007
rs3094220	-0,13	0,001032

rs6769400	-1,46	0,001063
rs11926768	-1,21	0,001123
rs665265	-3,20	0,001191
rs1569196	1,48	0,001215
rs7664681	-2,21	0,001345
rs1317122	-1,35	0,001375
rs10134002	-0,84	0,001399
rs3003301	-2,18	0,001445
rs11867808	-1,11	0,001494
rs17338569	-1,82	0,001575
rs2021399	0,18	0,001620
rs7254995	0,18	0,001620
rs12363011	1,01	0,001628
rs3130977	-0,40	0,001654
rs10897991	0,54	0,001805
rs12321232	-2,58	0,001811
rs11761049	-1,94	0,001825
rs2308974	-1,25	0,001826
rs2180451	1,83	0,001868
rs12985137	1,87	0,001878
rs3822223	1,92	0,001916
rs4888108	-1,64	0,001953
rs2472200	-0,52	0,001956
rs202841	2,86	0,001984
rs11030828	1,68	0,001986
rs12591359	-1,74	0,002017
rs3774161	-2,26	0,002041
rs12048859	-2,14	0,002108
rs2641801	-0,57	0,002121
rs10843050	-2,54	0,002143
rs1043720	-2,12	0,002154
rs775022	-1,93	0,002221
rs2641806	-0,56	0,002235
rs8046977	-1,16	0,002278
rs34881	-1,56	0,002344
rs2089979	-0,43	0,002352
rs10924924	2,09	0,002362
rs9325377	-0,27	0,002367
rs4392201	0,57	0,002369
rs154977	-1,88	0,002399
rs7208811	1,98	0,002465
rs7208811	1,98	0,002465
rs7081104	0,80	0,002482
rs12238387	3,42	0,002494
rs16907311	3,42	0,002494

rs7950814	-2,13	0,002575
rs10487756	0,16	0,002637
rs4697106	2,20	0,002659
rs10835566	0,95	0,002685
rs10835567	0,95	0,002685
rs299982	-1,83	0,002693
rs4678554	-1,10	0,002731
rs4678553	-1,10	0,002733
rs7646422	-1,10	0,002733
rs13340504	2,33	0,002737
rs6442169	2,01	0,002791
rs3745437	0,94	0,002805
rs4916555	-0,29	0,002816
rs8112051	0,87	0,002843
rs8048818	-1,11	0,002883
rs751122	-1,18	0,002991
rs569629	-1,69	0,003024
rs964632	-0,34	0,003034
rs4758272	1,21	0,003154
rs2240998	-0,44	0,003302
rs3761072	-1,63	0,003327
rs925135	1,74	0,003391
rs321913	-1,93	0,003432
rs7189895	-0,46	0,003484
rs5756306	-1,75	0,003508
rs4802787	-0,25	0,003560
rs10402912	-0,43	0,003673
rs6956301	0,39	0,003727
rs302838	1,58	0,003767
rs1448087	-1,96	0,003781
rs11658099	0,73	0,003797
rs7213337	0,73	0,003797
rs7213435	0,73	0,003797
rs3789818	-0,35	0,003804
rs3130982	-0,22	0,003860
rs1054485	1,70	0,003910
rs1133327	1,70	0,003910
rs3814	1,70	0,003910
rs1009401	2,77	0,003927
rs12483496	1,73	0,003927
rs7199100	1,07	0,003934
rs9311137	-0,93	0,003984
rs4813406	-1,97	0,004050
rs11854123	0,24	0,004067
rs4473307	1,05	0,004103

rs2270190	0,84	0,004108
rs11749241	3,39	0,004211
rs3844080	2,08	0,004223
rs10835608	-1,56	0,004260
rs3784932	-0,93	0,004267
rs4658537	1,34	0,004274
rs2392581	1,23	0,004305
rs4149501	-0,93	0,004385
rs7202717	-0,93	0,004385
rs10154916	-1,30	0,004394
rs1046512	-1,30	0,004394
rs1558529	-1,30	0,004394
rs3172297	-1,30	0,004394
rs6550457	-1,30	0,004394
rs6809297	-1,30	0,004394
rs7611106	-1,30	0,004394
rs9846039	-1,30	0,004394
rs9869432	-1,30	0,004394
rs753414	-2,06	0,004422
rs11655211	-1,52	0,004424
rs3745181	-1,39	0,004537
rs16944155	0,01	0,004646
rs12741729	0,71	0,004699
rs3093998	-0,31	0,004724
rs9793102	2,04	0,004726
rs7194028	1,12	0,004741
rs12462953	1,01	0,004745
rs7132930	-2,41	0,004791
rs983618	-2,17	0,004844
rs3786656	0,01	0,004845
rs736574	0,29	0,004868
rs2130357	-0,89	0,004872
rs2077955	-1,79	0,004916
rs9878419	-1,14	0,004922
rs6106989	1,25	0,004975
rs4897509	2,19	0,004995
rs9483252	2,19	0,004995
rs9483253	2,19	0,004995
rs7002094	1,99	0,005081
rs4620627	-2,17	0,005085
rs2086140	-1,14	0,005115
rs10466280	-1,56	0,005122
rs1053403	-1,56	0,005122
rs10906090	-1,56	0,005122
rs9633765	-1,56	0,005122

rs721575	-1,16	0,005171
rs10484220	2,30	0,005319
rs2451994	1,78	0,005379
rs9492908	2,12	0,005430
rs3130983	-0,10	0,005488
rs2054191	-1,37	0,005495
rs1042126	-0,08	0,005502
rs1042134	-0,08	0,005502
rs1042147	-0,08	0,005502
rs3094212	-0,08	0,005502
rs3094214	-0,08	0,005502
rs3094217	-0,08	0,005502
rs1471886	0,66	0,005611
rs10416187	0,08	0,005630
rs321938	1,35	0,005679
rs852214	1,21	0,005705
rs12626864	2,53	0,005709
rs2956173	-0,58	0,005713
rs12973596	0,21	0,005754
rs4238908	1,44	0,005874
rs4238909	1,44	0,005874
rs4238909	1,44	0,005874
rs1419094	-1,64	0,005917
rs1954407	-1,64	0,005917
rs3959644	-1,97	0,006023
rs1494676	0,34	0,006050
rs9428953	-1,14	0,006121
rs10929225	1,15	0,006156
rs2111751	-1,60	0,006161
rs1799977	-1,40	0,006164
rs16894184	-1,46	0,006219
rs3744395	1,65	0,006231
rs2136282	1,64	0,006321
rs10459592	-1,44	0,006332
rs2561045	1,06	0,006372
rs8111845	-0,71	0,006434
rs218671	-0,27	0,006500
rs3130564	-0,10	0,006519
rs17643897	3,19	0,006566
rs906482	1,61	0,006583
rs725027	-1,48	0,006624
rs7219746	-1,11	0,006636
rs12465759	1,08	0,006666
rs2669864	1,71	0,006733
rs3123710	-1,98	0,006798

rs10903010	-2,56	0,006799
rs150306	1,59	0,006812
rs7025567	2,81	0,006836
rs9873742	-1,21	0,006883
rs4566975	0,22	0,006899
rs4629710	-1,98	0,006909
rs10897988	0,76	0,006922
rs1939634	0,76	0,006933
rs11973301	0,12	0,006945
rs3789541	3,26	0,007045
rs9904113	1,44	0,007050
rs3733545	-1,29	0,007087
rs11048434	0,72	0,007193
rs9821223	-1,09	0,007210
rs10233502	-1,34	0,007276
rs446821	0,40	0,007339
rs446821	0,40	0,007339
rs4981720	2,56	0,007349
rs1706804	-1,74	0,007380
rs576063	-1,92	0,007432
rs184596	-1,30	0,007572
rs1144539	0,46	0,007626
rs2431776	0,46	0,007626
rs4944403	0,36	0,007643
rs6830679	-1,69	0,007660
rs12592697	-1,54	0,007662
rs2414095	-1,54	0,007662
rs12039141	-1,48	0,007690
rs883823	-0,65	0,007812
rs7868771	2,92	0,007875
rs9483254	2,07	0,008055
rs7578007	1,98	0,008062
rs978763	-2,25	0,008107
rs7240655	-2,63	0,008188
rs1412470	1,90	0,008204
rs6684324	-1,49	0,008206
rs11714766	-1,10	0,008264
rs11718848	-1,10	0,008264
rs12892774	1,55	0,008302
rs678456	2,82	0,008398
rs4796691	0,88	0,008420
rs11084857	-0,65	0,008436
rs391224	1,55	0,008614
rs4624519	-1,04	0,008652
rs6964720	-1,94	0,008660

rs2239511	1,26	0,008683
rs10412454	2,16	0,008689
rs2215564	-1,73	0,008711
rs12460438	1,18	0,008764
rs7246529	1,18	0,008764
rs9917042	-0,68	0,008767
rs8072340	1,64	0,008852
rs9662967	-1,31	0,008858
rs9606990	-0,08	0,008891
rs2665316	-0,40	0,008913
rs917914	-2,04	0,008960
rs3094	1,11	0,009010
rs567688	-2,03	0,009015
rs808183	-2,03	0,009015
rs1806190	-1,26	0,009082
rs3745920	1,71	0,009153
rs910527	1,23	0,009174
rs4517893	0,63	0,009240
rs4801677	1,85	0,009243
rs1425172	-1,38	0,009247
rs7131784	-0,63	0,009312
rs6115024	1,08	0,009326
rs3127464	-2,02	0,009368
rs10042507	-1,39	0,009412
rs497082	1,45	0,009464
rs1867485	-1,28	0,009531
rs12987284	2,04	0,009534
rs17803366	-0,24	0,009569
rs7255193	-1,91	0,009583
rs4916546	-0,21	0,009601
rs817361	-0,48	0,009605
rs10401807	0,42	0,009606
rs10415969	0,42	0,009606
rs484459	0,48	0,009651
rs3754047	1,34	0,009679
rs10224856	0,47	0,009695
rs17805954	-0,88	0,009716
rs256259	-2,03	0,009730
rs10049181	-1,21	0,009775
rs1058656	-1,31	0,009805
rs133708	0,17	0,009848
rs3895614	-2,92	0,009978
rs4575769	-0,32	0,010009
rs4406174	-2,31	0,010015
rs6978657	-0,81	0,010048

rs10275580	-2,40	0,010058
rs4801676	-1,86	0,010210
rs4234258	-0,98	0,010234
rs34878	-1,57	0,010293
rs7573476	-0,63	0,010305
rs7247178	0,86	0,010325
rs1034597	-0,63	0,010360
rs1376406	-1,52	0,010515
rs11868896	1,69	0,010556
rs2074989	-1,54	0,010602
rs11541061	1,09	0,010621
rs8081465	3,04	0,010839
rs2248372	-0,50	0,010873
rs7253698	-0,91	0,010950
rs10058874	-1,24	0,010971
rs12260965	1,37	0,011012
rs9933029	1,93	0,011028
rs11031084	1,42	0,011030
rs980561	1,42	0,011030
rs9674202	0,85	0,011042
rs868344	0,64	0,011124
rs1549431	3,18	0,011144
rs12704962	-1,92	0,011162
rs16974196	-1,19	0,011201
rs2844479	1,45	0,011217
rs1345197	-1,73	0,011261
rs11149841	-0,89	0,011268
rs17823065	0,63	0,011367
rs1961205	-0,14	0,011396
rs10142601	-2,53	0,011418
rs12608027	-0,17	0,011509
rs12162264	1,99	0,011545
rs11079002	1,41	0,011555
rs321921	0,72	0,011743
rs336601	1,79	0,011763
rs11041816	0,29	0,011767
rs929708	-2,03	0,011788
rs5750428	0,33	0,011861
rs11671438	-0,52	0,011861
rs2863244	0,95	0,011917
rs34888	-1,47	0,011941
rs2961040	-1,52	0,011942
rs10906083	-1,56	0,011979
rs16970295	0,64	0,011993
rs17245276	-0,68	0,012021

rs321948	-0,03	0,012031
rs965991	1,69	0,012042
rs4098386	0,91	0,012090
rs2208985	1,04	0,012143
rs6503653	-0,98	0,012169
rs2908197	-1,51	0,012220
rs302827	1,97	0,012249
rs1868844	-1,29	0,012378
rs10512206	2,38	0,012402
rs7025775	2,38	0,012402
rs10803657	-0,56	0,012468
rs2876142	1,68	0,012495
rs6511863	-1,89	0,012574
rs11084088	-0,34	0,012581
rs1036292	-0,93	0,012582
rs3784828	-2,64	0,012599
rs13159894	2,14	0,012704
rs4995289	1,36	0,012713
rs10424440	-1,88	0,012734
rs6542668	2,07	0,012736
rs17775666	-0,96	0,012766
rs30007	-0,79	0,012793
rs354021	-0,79	0,012793
rs2305670	1,87	0,012831
rs11049326	1,61	0,012910
rs2240850	1,39	0,012924
rs3807411	-1,29	0,013001
rs2302503	-1,11	0,013019
rs11050581	3,09	0,013034
rs3735933	-0,39	0,013037
rs6602563	-1,53	0,013040
rs300029	0,85	0,013056
rs4656879	-2,00	0,013058
rs11127185	0,14	0,013121
rs4926279	1,64	0,013166
rs2055566	0,31	0,013308
rs4815366	1,06	0,013333
rs7129903	1,52	0,013384
rs703913	-1,14	0,013389
rs11762854	-1,60	0,013393
rs1840549	1,46	0,013433
rs239917	-1,78	0,013508
rs354020	-0,79	0,013534
rs354026	-0,79	0,013534
rs12453963	0,95	0,013612

rs1648302	-1,75	0,013649
rs12972487	-1,73	0,013699
rs12450574	-1,75	0,013746
rs10461515	2,13	0,013790
rs1016499	-2,34	0,013812
rs2174649	-1,75	0,013820
rs11869824	0,73	0,013879
rs11563459	-1,41	0,013880
rs17865261	-1,41	0,013880
rs529444	-2,03	0,013921
rs808182	-2,03	0,013921
rs533495	-1,18	0,013961
rs12701661	0,34	0,014003
rs13235997	0,34	0,014003
rs187675	-1,23	0,014032
rs741495	-1,14	0,014059
rs1673087	0,69	0,014066
rs1673087	0,69	0,014066
rs2894239	-0,73	0,014115
rs2894240	-0,73	0,014115
rs3115573	-0,73	0,014115
rs3130315	-0,73	0,014115
rs40180	-1,18	0,014158
rs12026068	0,46	0,014241
rs12993525	0,88	0,014297
rs7959272	0,41	0,014437
rs7821637	1,77	0,014493
rs9969775	2,88	0,014495
rs10754800	0,10	0,014508
rs7912899	0,74	0,014547
rs3108612	1,15	0,014813
rs2242144	-2,29	0,014921
rs10500320	-0,62	0,014929
rs4452278	-1,03	0,014940
rs10418924	0,28	0,015002
rs3121478	0,73	0,015029
rs3127106	0,73	0,015029
rs10116326	2,30	0,015068
rs2001917	2,30	0,015068
rs6443732	-1,19	0,015103
rs1559451	-1,04	0,015112
rs1865258	-1,15	0,015117
rs8018826	-1,69	0,015186
rs10411772	0,14	0,015303
rs11878327	3,80	0,015325

rs17679495	3,80	0,015325
rs8112488	3,80	0,015325
rs12670489	-1,56	0,015372
rs7251590	1,10	0,015373
rs17176375	-2,52	0,015399
rs2109857	-1,46	0,015428
rs1516801	1,27	0,015451
rs896136	-0,75	0,015470
rs2861412	-1,05	0,015501
rs3748338	0,37	0,015571
rs2419851	-0,37	0,015606
rs2028209	1,34	0,015677
rs3752861	1,77	0,015709
rs7256494	-0,45	0,015725
rs1534415	-2,07	0,015747
rs306504	-1,70	0,015815
rs4744055	1,38	0,015850
rs4801965	-1,15	0,015868
rs302837	1,51	0,015902
rs8008227	-2,91	0,015919
rs3757340	-1,64	0,015956
rs11674746	0,50	0,016019
rs8076916	1,52	0,016023
rs10518287	1,12	0,016070
rs12616328	-1,00	0,016080
rs10170348	1,32	0,016096
rs731034	1,32	0,016096
rs16941867	0,97	0,016105
rs17703883	-1,69	0,016113
rs13191519	-0,08	0,016122
rs4658609	0,70	0,016136
rs4783619	1,78	0,016177
rs10409409	-2,58	0,016180
rs12653946	-0,81	0,016233
rs4001921	-0,97	0,016243
rs8102683	-0,97	0,016243
rs8105704	-0,97	0,016243
rs2099361	1,18	0,016285
rs2207631	1,13	0,016424
rs707919	1,46	0,016430
rs755714	1,46	0,016430
rs805297	1,46	0,016430
rs805297	1,46	0,016430
rs11635236	-0,32	0,016495
rs1908970	-1,68	0,016495

rs6502697	2,36	0,016509
rs2247788	-0,55	0,016607
rs2292467	-1,64	0,016612
rs10484554	-0,06	0,016617
rs12609640	1,33	0,016626
rs16964183	0,50	0,016631
rs13073459	-1,44	0,016633
rs10177762	1,88	0,016637
rs7554246	0,25	0,016669
rs1868158	1,71	0,016758
rs34293605	-1,34	0,016891
rs17035429	0,44	0,016900
rs7146867	1,31	0,016916
rs11133917	-0,85	0,016936
rs2289273	-1,33	0,016939
rs6801189	-1,35	0,016977
rs3114329	-1,11	0,017023
rs8301	0,78	0,017033
rs3786544	-0,31	0,017145
rs735152	1,16	0,017161
rs2217228	1,47	0,017174
rs8073904	1,66	0,017176
rs2430342	1,17	0,017247
rs9267956	-0,71	0,017261
rs17090907	-0,72	0,017281
rs11673436	0,94	0,017327
rs2229375	0,94	0,017327
rs2047551	-1,30	0,017364
rs1059684	0,95	0,017449
rs7247847	0,95	0,017449
rs740423	0,95	0,017449
rs7032571	2,10	0,017454
rs1129376	-0,63	0,017491
rs2967490	-0,63	0,017491
rs718880	1,52	0,017513
rs4758259	-1,67	0,017536
rs13343928	-0,71	0,017554
rs13096176	-1,33	0,017602
rs2516400	0,05	0,017658
rs439142	-1,72	0,017672
rs6994164	-0,99	0,017682
rs8776	0,47	0,017731
rs3763355	-1,82	0,017760
rs6496649	-0,82	0,017878
rs4855015	-1,33	0,017952

rs11771771	-1,52	0,017970
rs8179078	2,54	0,017982
rs6492971	-1,01	0,018041
rs8126818	2,32	0,018074
rs151338	-0,88	0,018096
rs17794612	1,33	0,018123
rs2261033	-1,23	0,018164
rs11684304	1,50	0,018173
rs10771407	-1,74	0,018178
rs11233368	-1,69	0,018198
rs10889286	-0,71	0,018211
rs1507882	0,51	0,018224
rs3094014	-0,37	0,018244
rs2275007	-1,67	0,018257
rs5754521	-1,50	0,018288
rs17805805	-1,11	0,018298
rs10924919	1,63	0,018335
rs6426220	1,83	0,018336
rs11233045	0,00	0,018402
rs6805151	1,73	0,018406
rs816947	-1,20	0,018429
rs4783597	-0,01	0,018445
rs3814837	1,81	0,018457
rs2560996	-1,40	0,018507
rs3862761	0,16	0,018521
rs10761133	1,67	0,018594
rs2012160	0,61	0,018639
rs7359414	-1,10	0,018743
rs11867232	2,57	0,018786
rs956796	0,50	0,018816
rs9889514	2,58	0,018830
rs1948690	-1,30	0,018858
rs12441886	-1,85	0,018888
rs4926278	1,66	0,019015
rs10044567	-1,53	0,019017
rs4345919	1,48	0,019032
rs7220116	-0,37	0,019113
rs10853858	-1,11	0,019219
rs1039402	-2,12	0,019225
rs10247414	1,68	0,019266
rs10248665	1,68	0,019266
rs7251374	0,87	0,019305
rs17771942	0,64	0,019306
rs281477	-0,08	0,019324
rs9647441	1,76	0,019329

rs2561143	0,96	0,019345
rs3769080	2,03	0,019392
rs6547862	0,69	0,019401
rs10934962	-1,15	0,019433
rs4487219	-1,15	0,019433
rs1861085	-0,01	0,019444
rs11125091	0,85	0,019472
rs2458236	-1,53	0,019514
rs13340490	1,56	0,019552
rs2242	1,80	0,019630
rs7227276	-2,18	0,019648
rs10422961	1,13	0,019679
rs11948984	-1,91	0,019693
rs10425196	-1,27	0,019782
rs826278	0,46	0,019793
rs7777461	2,62	0,019806
rs16983718	2,63	0,019809
rs9928066	2,53	0,019839
rs6448380	1,84	0,019935
rs7418709	-0,71	0,019942
rs2232376	-1,23	0,019958
rs4815390	1,68	0,020000
rs11744749	1,67	0,020035
rs7976	1,44	0,020081
rs3778929	0,72	0,020115
rs13019382	-1,16	0,020156
rs2242089	-1,79	0,020206
rs17875093	-2,19	0,020268
rs4629018	1,50	0,020277
rs10927011	-1,05	0,020336
rs9469220	-1,00	0,020363
rs9903342	-1,41	0,020384
rs3127469	-1,86	0,020449
rs3745180	1,45	0,020495
rs9806724	0,60	0,020560
rs719318	-2,90	0,020568
rs7197382	-0,50	0,020579
rs1648311	-1,53	0,020627
rs1065778	-1,22	0,020652
rs700518	-1,22	0,020652
rs10775558	-1,11	0,020722
rs13194781	-0,55	0,020769
rs13199772	-0,55	0,020769
rs13212651	-0,55	0,020769
rs34706883	-0,55	0,020769

rs3754706	1,72	0,020795
rs596134	0,54	0,020806
rs13338503	2,36	0,020808
rs285529	1,16	0,020811
rs3863502	-0,50	0,020885
rs7218964	-0,50	0,020902
rs4975713	-0,57	0,020926
rs386234	-1,46	0,020936
rs6572635	1,19	0,021004
rs938883	-1,50	0,021006
rs6115248	2,87	0,021027
rs2516408	0,08	0,021082
rs17716681	-1,87	0,021092
rs6756738	0,55	0,021114
rs3828903	0,45	0,021172
rs3828912	0,45	0,021172
rs4807120	-1,80	0,021259
rs1121693	2,63	0,021290
rs1125620	-2,67	0,021353
rs1147238	1,20	0,021368
rs887782	-1,98	0,021415
rs6965558	1,28	0,021451
rs3779537	-2,09	0,021457
rs282546	1,26	0,021548
rs3848368	0,58	0,021574
rs2278445	1,04	0,021662
rs7622114	1,05	0,021665
rs7251313	0,79	0,021705
rs10835549	0,53	0,021738
rs1122729	1,46	0,021763
rs352826	-0,96	0,021775
rs2070481	-2,23	0,021784
rs13030	1,74	0,021792
rs12044746	-0,70	0,021811
rs11771651	1,61	0,021933
rs13160153	-1,44	0,022012
rs897414	0,62	0,022062
rs1347684	-1,33	0,022066
rs7131589	0,17	0,022113
rs1468033	-1,20	0,022121
rs299993	-1,18	0,022134
rs218489	0,23	0,022143
rs17173651	-1,42	0,022146
rs3787958	-1,55	0,022151
rs2112826	0,71	0,022165

rs2616949	0,71	0,022165
rs9692932	-0,40	0,022183
rs17763551	-2,22	0,022206
rs11166389	1,42	0,022213
rs12146139	1,42	0,022213
rs6699449	1,42	0,022213
rs34872	1,37	0,022228
rs2523591	-0,30	0,022233
rs9636113	-1,62	0,022235
rs887611	-0,05	0,022238
rs887614	-0,05	0,022238
rs16991758	2,37	0,022243
rs2076037	-0,23	0,022270
rs6599536	-0,56	0,022487
rs11761211	-3,12	0,022493
rs11851004	-0,16	0,022515
rs7558419	-1,20	0,022523
rs1059495	-0,24	0,022546
rs11233363	-1,81	0,022571
rs6734610	-0,84	0,022584
rs17749927	-0,36	0,022659
rs12896466	-1,21	0,022663
rs4324103	0,22	0,022674
rs11084943	1,17	0,022727
rs233697	-1,25	0,022736
rs12925157	-1,38	0,022801
rs11654241	1,19	0,022825
rs16877296	-1,43	0,022880
rs2074560	1,54	0,022882
rs4969260	-1,62	0,022914
rs7094186	-0,27	0,023043
rs883105	-1,94	0,023044
rs9650539	1,97	0,023152
rs11746674	-0,14	0,023174
rs9265797	-0,58	0,023183
rs17537048	-1,80	0,023190
rs1805761	0,82	0,023271
rs2319375	-1,81	0,023278
rs10767843	-0,06	0,023279
rs11125055	0,08	0,023421
rs28113	1,65	0,023494
rs9606993	-0,64	0,023528
rs2274890	1,97	0,023543
rs2838057	0,90	0,023566
rs7186693	-1,11	0,023571

rs7342665	0,50	0,023574
rs4783598	-0,76	0,023762
rs16990824	2,37	0,023765
rs4883203	-2,01	0,023770
rs13246622	1,73	0,023812
rs17113112	-0,04	0,023860
rs2285337	-0,94	0,023896
rs1355384	-1,67	0,023913
rs7253270	-0,06	0,023913
rs1704492	-0,83	0,023930
rs11650388	1,33	0,024009
rs11653862	1,33	0,024009
rs12940147	1,33	0,024009
rs3744784	1,33	0,024009
rs28382590	-1,10	0,024022
rs28382609	-1,10	0,024022
rs1459826	0,90	0,024050
rs13385	0,78	0,024081
rs13410076	-3,29	0,024082
rs690111	0,66	0,024153
rs8013858	2,69	0,024165
rs2763979	-1,19	0,024310
rs7651477	-0,89	0,024318
rs2116640	-1,55	0,024343
rs9468877	-1,14	0,024496
rs2275293	0,46	0,024518
rs1534476	0,53	0,024539
rs2059137	1,31	0,024556
rs2112798	1,31	0,024556
rs9902034	1,44	0,024575
rs8078571	1,71	0,024585
rs1148395	0,71	0,024652
rs150311	-1,42	0,024669
rs17714284	-1,71	0,024727
rs6978712	-1,71	0,024727
rs807293	1,20	0,024796
rs11645180	-0,42	0,024803
rs321930	1,63	0,024814
rs16973232	1,04	0,024834
rs9311138	-0,72	0,024837
rs7250857	-0,36	0,024839
rs48524	-0,51	0,024880
rs2440592	0,74	0,024930
rs4775932	-1,16	0,024940
rs6493489	-1,16	0,024940

rs10262232	-1,26	0,024960
rs2249380	1,66	0,025115
rs4015533	-0,06	0,025137
rs10046	-1,18	0,025216
rs8113651	1,05	0,025249
rs7155104	1,28	0,025260
rs432519	0,98	0,025263
rs1015166	-1,11	0,025326
rs2162683	-1,16	0,025332
rs3757188	-1,14	0,025344
rs3757188	-1,14	0,025344
rs4713139	-1,14	0,025344
rs4713140	-1,14	0,025344
rs9380064	-1,14	0,025344
rs1609254	1,52	0,025372
rs2837860	2,25	0,025396
rs10165099	0,91	0,025399
rs10865541	0,91	0,025399
rs12983952	-0,86	0,025440
rs12165814	2,03	0,025588
rs1894529	2,03	0,025588
rs6509632	-1,77	0,025614
rs2459553	2,86	0,025618
rs2855812	0,33	0,025632
rs11692425	0,91	0,025646
rs1122713	0,11	0,025703
rs7219088	1,26	0,025725
rs10280802	-1,98	0,025731
rs2240803	-1,95	0,025733
rs3852872	-0,27	0,025763
rs11667458	-1,83	0,025767
rs4624520	-0,89	0,025809
rs6137194	-1,39	0,025812
rs16877508	0,16	0,025841
rs1109813	-1,06	0,025901
rs11129756	-0,86	0,025958
rs2531811	-1,29	0,025986
rs2374042	-0,34	0,026054
rs4815007	1,31	0,026055
rs4729435	0,12	0,026067
rs17739110	-0,65	0,026070
rs2392644	-0,76	0,026080
rs16956134	-1,91	0,026136
rs3094691	0,23	0,026145
rs7557193	-0,91	0,026205

rs6122232	-0,91	0,026242
rs11772568	-1,21	0,026264
rs927203	1,48	0,026277
rs3120073	0,47	0,026305
rs12152512	-0,82	0,026331
rs1552244	-0,82	0,026331
rs2272124	-0,82	0,026331
rs6789156	-0,82	0,026331
rs7647987	-0,82	0,026331
rs9811771	-0,82	0,026331
rs9849434	-0,82	0,026331
rs2068031	-0,19	0,026333
rs16944179	1,02	0,026382
rs239960	-0,99	0,026415
rs305998	-0,91	0,026498
rs11085908	-0,80	0,026501
rs11666622	-0,80	0,026501
rs4808319	-0,80	0,026501
rs4678953	-0,85	0,026520
rs6978214	2,61	0,026560
rs853676	-1,52	0,026601
rs853679	-1,52	0,026601
rs853685	-1,52	0,026601
rs1004320	-0,90	0,026619
rs9368676	-1,41	0,026621
rs10952955	-2,59	0,026644
rs8140859	0,72	0,026645
rs327518	0,58	0,026652
rs7750269	-1,47	0,026652
rs7760988	-1,47	0,026652
rs9368675	-1,47	0,026652
rs10064525	-3,01	0,026730
rs2243429	0,22	0,026745
rs2248617	0,22	0,026745
rs2395488	0,22	0,026745
rs2596536	0,22	0,026745
rs2844502	0,22	0,026745
rs7196165	1,52	0,026761
rs10991820	1,61	0,026789
rs9380238	-0,84	0,026850
rs7518025	-0,40	0,026897
rs302868	-0,93	0,026931
rs7189606	1,06	0,026955
rs6651030	-0,50	0,026973
rs10224728	-1,39	0,026983

rs10260986	-1,39	0,026983
rs12972967	-0,59	0,027027
rs2516424	0,20	0,027059
rs1765132	0,01	0,027065
rs10405154	1,42	0,027129
rs10421748	1,42	0,027129
rs11673561	1,37	0,027132
rs2279345	1,20	0,027157
rs6508965	1,20	0,027157
rs12551140	1,41	0,027169
rs1419391	0,91	0,027180
rs7643836	-1,09	0,027225
rs10851498	-1,17	0,027279
rs12907866	-1,18	0,027332
rs8057453	1,29	0,027428
rs6479339	2,02	0,027465
rs11642783	-1,47	0,027526
rs17120799	-0,89	0,027556
rs2354025	-1,34	0,027562
rs2091181	1,50	0,027565
rs2239694	-1,78	0,027618
rs1939633	0,43	0,027628
rs1010222	0,95	0,027677
rs4728533	-0,92	0,027748
rs11876	1,92	0,027762
rs13173678	1,54	0,027829
rs2102454	1,54	0,027829
rs28445612	1,54	0,027829
rs12919935	-1,21	0,027841
rs2249742	0,38	0,027856
rs10499602	-1,24	0,027924
rs1353622	-0,15	0,027927
rs10492108	1,38	0,027972
rs7199663	-0,11	0,028059
rs10407043	1,57	0,028069
rs2863978	1,67	0,028113
rs3803650	1,67	0,028113
rs7214863	0,90	0,028126
rs11650969	0,45	0,028149
rs1951244	1,77	0,028158
rs1579333	0,01	0,028160
rs4923889	-0,93	0,028171
rs7611218	-0,78	0,028217
rs2974750	0,94	0,028330
rs7736642	-0,53	0,028346

rs6502998	-1,69	0,028355
rs11652437	1,28	0,028356
rs10075553	1,42	0,028396
rs2112502	1,09	0,028416
rs2546020	1,09	0,028416
rs1042357	-1,64	0,028427
rs2962061	-1,38	0,028460
rs570901	-1,14	0,028468
rs1126667	-1,64	0,028504
rs434473	-1,64	0,028504
rs27267	1,57	0,028511
rs12828464	-0,37	0,028574
rs503832	-1,14	0,028661
rs11006	0,75	0,028687
rs1034593	1,62	0,028725
rs8065475	1,62	0,028725
rs2143462	-0,81	0,028770
rs17440466	-1,17	0,028775
rs521371	0,70	0,028840
rs6690236	-1,07	0,028887
rs4850000	-0,52	0,028906
rs13064158	-1,76	0,028982
rs2844475	1,05	0,029060
rs7612650	1,00	0,029098
rs1612501	1,60	0,029132
rs4503883	0,71	0,029152
rs10927025	-0,25	0,029176
rs7235189	1,00	0,029194
rs7777477	1,39	0,029256
rs1590287	0,94	0,029319
rs2166799	0,34	0,029394
rs10416717	-0,13	0,029395
rs12289723	0,81	0,029457
rs7732589	-0,60	0,029461
rs10767831	-0,83	0,029473
rs2163819	-0,80	0,029482
rs10488823	0,19	0,029508
rs1019537	-0,86	0,029513
rs1019539	-0,86	0,029513
rs1003531	-1,57	0,029559
rs6740539	0,65	0,029566
rs7503417	0,02	0,029599
rs16991717	1,75	0,029600
rs292828	-1,55	0,029682
rs3108176	0,88	0,029738

rs3745779	0,88	0,029738
rs150304	-1,29	0,029743
rs9368677	-1,41	0,029841
rs4490097	-1,59	0,029865
rs2303728	0,46	0,029869
rs12439639	-2,44	0,029877
rs1018224	-1,02	0,029914
rs3944083	-0,02	0,029918
rs16941642	-1,06	0,029963
rs10160238	0,69	0,030015
rs6572633	1,55	0,030111
rs13319597	-0,73	0,030116
rs2272123	-0,73	0,030116
rs6442150	-0,73	0,030116
rs12930993	0,99	0,030168
rs538815	-1,28	0,030278
rs8059599	-1,17	0,030300
rs4405662	0,87	0,030373
rs1057899	-3,20	0,030393
rs13404342	-3,20	0,030393
rs2034360	0,25	0,030464
rs2303279	-1,07	0,030466
rs9366717	-1,16	0,030490
rs9380069	-1,16	0,030490
rs2070590	-1,62	0,030519
rs312466	-1,62	0,030519
rs2903813	0,00	0,030520
rs10151120	0,41	0,030556
rs3769058	1,16	0,030578
rs733579	1,16	0,030578
rs10640	-1,43	0,030596
rs10640	-1,43	0,030596
rs11706370	-1,43	0,030596
rs11715915	-1,43	0,030596
rs17080528	-1,43	0,030596
rs3811697	-1,43	0,030596
rs3811699	-1,43	0,030596
rs4625	-1,43	0,030596
rs6997	-1,43	0,030596
rs2857697	1,04	0,030650
rs7792547	1,10	0,030696
rs9461425	-1,10	0,030735
rs12995190	1,59	0,030771
rs8111895	-1,57	0,030774
rs2278168	0,83	0,030948

rs16974799	-0,39	0,030976
rs8101756	-0,39	0,030976
rs9267947	-1,06	0,031008
rs7255497	-0,43	0,031012
rs4953442	0,88	0,031025
rs3757598	-1,08	0,031036
rs12445094	1,18	0,031044
rs2242263	0,53	0,031059
rs6509936	1,70	0,031090
rs12911554	-1,24	0,031130
rs11912647	1,88	0,031152
rs13053913	1,88	0,031152
rs749326	1,88	0,031152
rs2736171	1,03	0,031164
rs17779789	-1,06	0,031165
rs569	0,70	0,031185
rs2899292	-1,59	0,031195
rs12459408	-1,83	0,031226
rs9905704	-0,93	0,031243
rs3094228	-0,46	0,031245
rs8074184	1,60	0,031271
rs540	0,36	0,031294
rs2430984	-0,12	0,031311
rs1709127	1,84	0,031340
rs32376	0,96	0,031431
rs9267431	0,08	0,031450
rs1317548	0,14	0,031471
rs176649	-1,49	0,031478
rs9888812	0,33	0,031488
rs3850818	1,63	0,031515
rs304272	-0,92	0,031641
rs994211	1,75	0,031669
rs1444997	0,65	0,031680
rs10419880	-0,54	0,031705
rs1105488	0,92	0,031775
rs1105489	0,92	0,031775
rs548577	-1,65	0,031847
rs2269879	0,99	0,031885
rs10131141	1,03	0,032007
rs7200646	0,20	0,032113
rs1534625	-0,26	0,032115
rs4274551	-1,30	0,032133
rs2071800	0,95	0,032136
rs35439528	0,95	0,032136
rs16952712	2,34	0,032179

rs2267807	1,45	0,032183
rs11233471	0,86	0,032199
rs1017079	-0,02	0,032222
rs1061303	0,47	0,032282
rs10139335	1,16	0,032328
rs17032943	1,79	0,032509
rs10512656	-2,16	0,032523
rs2140631	-1,36	0,032549
rs1508518	0,43	0,032661
rs6577156	1,29	0,032771
rs7515676	1,29	0,032771
rs11041994	1,27	0,032921
rs446079	-1,87	0,032970
rs11692447	1,99	0,032987
rs2244632	-1,08	0,032992
rs4802602	1,38	0,033013
rs2273019	-0,96	0,033030
rs4787289	1,24	0,033036
rs247456	-0,12	0,033213
rs11081311	-1,83	0,033243
rs2029771	-1,48	0,033249
rs11648529	-1,48	0,033323
rs10846594	2,08	0,033409
rs11057424	2,08	0,033409
rs10408012	-1,67	0,033417
rs1439616	-1,37	0,033434
rs2619508	-1,40	0,033434
rs2863981	1,63	0,033521
rs13084370	-1,12	0,033542
rs774227	1,80	0,033713
rs1033500	-0,78	0,033756
rs12459227	-2,03	0,033759
rs10807100	-0,78	0,033770
rs2073046	-0,78	0,033770
rs2076538	-0,78	0,033770
rs2143465	-0,78	0,033770
rs2395114	-0,78	0,033770
rs3817982	-0,78	0,033770
rs4576282	-0,78	0,033770
rs4713518	-0,78	0,033770
rs485774	-0,78	0,033770
rs4959096	-0,78	0,033770
rs502626	-0,78	0,033770
rs531094	-0,78	0,033770
rs533885	-0,78	0,033770

rs537757	-0,78	0,033770
rs547261	-0,78	0,033770
rs552339	-0,78	0,033770
rs6930681	-0,78	0,033770
rs742582	-0,78	0,033770
rs9268132	-0,78	0,033770
rs9268326	-0,78	0,033770
rs9268368	-0,78	0,033770
rs9268384	-0,78	0,033770
rs9368713	-0,78	0,033770
rs9405090	-0,78	0,033770
rs4092050	-0,81	0,033822
rs12512256	1,93	0,033841
rs6564809	-0,33	0,033841
rs1033498	-0,78	0,033844
rs10803143	0,96	0,033892
rs1000620	0,74	0,033970
rs3830160	0,43	0,034002
rs4926465	0,15	0,034075
rs9304870	-0,54	0,034080
rs11535536	1,44	0,034277
rs6509544	-0,72	0,034438
rs3751830	-0,91	0,034508
rs1939645	0,47	0,034518
rs10274639	-0,97	0,034519
rs7565800	-0,47	0,034519
rs9832138	-1,02	0,034650
rs10232298	0,53	0,034674
rs2156875	-0,55	0,034721
rs2157188	0,08	0,034733
rs621993	-1,63	0,034745
rs17442186	1,94	0,034761
rs4526148	-1,74	0,034777
rs6865765	-1,74	0,034777
rs10773019	-3,64	0,034780
rs10992142	1,37	0,034799
rs11869351	0,60	0,034828
rs2008018	1,64	0,034829
rs2395110	-0,68	0,034837
rs563412	-0,68	0,034862
rs12821842	0,69	0,034928
rs2377672	0,69	0,034928
rs4309200	0,69	0,034928
rs1190792	1,71	0,034940
rs13038639	0,82	0,034951

rs3132935	-0,93	0,034965
rs10414225	0,51	0,034970
rs1652042	-1,55	0,034989
rs9304764	-1,06	0,035010
rs33929	0,24	0,035017
rs741623	0,44	0,035108
rs756481	2,81	0,035131
rs17729239	0,08	0,035162
rs6766131	-1,37	0,035186
rs2543163	-0,31	0,035213
rs4938619	1,19	0,035220
rs12150116	1,55	0,035263
rs1760944	-1,72	0,035296
rs3793333	-0,53	0,035298
rs3744549	0,60	0,035302
rs7975557	-1,42	0,035374
rs10228030	-0,51	0,035424
rs10416626	1,70	0,035438
rs3020644	0,88	0,035445
rs12144733	1,55	0,035558
rs7210438	0,70	0,035608
rs2199619	0,38	0,035624
rs1939616	0,10	0,035722
rs1552223	1,14	0,035830
rs12989971	-0,22	0,035845
rs163607	-1,25	0,035873
rs1229014	-1,78	0,035875
rs247445	-0,76	0,035936
rs6972955	-0,70	0,035966
rs9436636	-1,07	0,036094
rs7131534	0,71	0,036137
rs12939500	-0,96	0,036153
rs3132486	0,07	0,036176
rs1271297	1,57	0,036192
rs2232430	-1,36	0,036221
rs10411036	1,05	0,036224
rs2545990	1,05	0,036224
rs2651125	1,05	0,036224
rs10227687	0,58	0,036255
rs916598	0,81	0,036289
rs4849159	-0,07	0,036294
rs10840024	-1,49	0,036308
rs10840025	-1,49	0,036308
rs2036839	-1,55	0,036315
rs11125110	-1,03	0,036315

rs6898102	1,37	0,036317
rs12468922	1,22	0,036352
rs1654313	1,39	0,036422
rs4843437	1,19	0,036467
rs10246472	1,05	0,036512
rs11684978	1,84	0,036541
rs8055977	-0,37	0,036583
rs12913123	-1,58	0,036586
rs12915027	-1,58	0,036586
rs2270616	1,55	0,036693
rs979203	-0,43	0,036733
rs12704984	0,85	0,036746
rs2302507	-1,08	0,036810
rs6789043	-1,08	0,036810
rs7159832	1,46	0,036824
rs33236	0,15	0,036853
rs4237772	0,49	0,037085
rs2118960	-1,36	0,037169
rs6806158	1,41	0,037181
rs10262191	-0,43	0,037199
rs6539968	0,93	0,037226
rs4148853	1,15	0,037299
rs6979784	-0,49	0,037355
rs11257506	-0,72	0,037472
rs2062587	-0,70	0,037476
rs1121276	1,24	0,037501
rs4900077	-1,26	0,037582
rs447205	-0,94	0,037588
rs3096686	-1,01	0,037647
rs7141747	1,28	0,037676
rs757102	-0,90	0,037681
rs4624466	-0,31	0,037713
rs1949074	-0,06	0,037726
rs6509874	-1,58	0,037778
rs4300683	2,16	0,037814
rs6502691	2,16	0,037814
rs11648609	-3,82	0,037831
rs852211	1,12	0,037865
rs9918633	-0,89	0,037968
rs885388	-0,46	0,037979
rs9607033	0,79	0,037993
rs1295107	-1,25	0,038030
rs4482520	0,83	0,038074
rs8070086	-0,62	0,038128
rs241433	-1,49	0,038169

rs10517086	1,43	0,038203
rs4926469	0,32	0,038205
rs4801980	0,84	0,038217
rs6115202	1,84	0,038218
rs10898880	0,67	0,038226
rs3130782	-0,42	0,038252
rs2611774	-0,83	0,038343
rs2469652	-1,42	0,038435
rs687513	1,29	0,038496
rs2023953	-1,21	0,038711
rs2303925	1,08	0,038734
rs4148850	1,08	0,038734
rs327510	-0,57	0,038744
rs9998340	-0,79	0,038763
rs482466	1,29	0,038845
rs1374163	1,32	0,038904
rs454748	-0,56	0,038938
rs9385571	0,31	0,039013
rs561119	1,29	0,039070
rs938890	-1,40	0,039118
rs6511148	0,25	0,039134
rs12984428	-1,42	0,039154
rs2254556	-0,13	0,039162
rs2596548	-0,13	0,039162
rs2596549	-0,13	0,039162
rs999197	0,52	0,039179
rs1693960	-0,70	0,039241
rs296614	1,78	0,039273
rs13205911	-0,96	0,039292
rs13218875	-0,96	0,039292
rs34859	-0,83	0,039320
rs11233366	-1,43	0,039323
rs338779	0,85	0,039323
rs2237803	-0,18	0,039452
rs17720293	-0,87	0,039453
rs577935	-1,42	0,039463
rs2541594	0,69	0,039494
rs886004	-0,17	0,039523
rs1205315	-1,41	0,039563
rs206015	0,73	0,039675
rs206016	0,73	0,039675
rs206019	0,73	0,039675
rs660550	-0,96	0,039686
rs1234256	0,76	0,039727
rs12221133	0,17	0,039735

rs5756825	-1,12	0,039744
rs12332927	-1,31	0,039806
rs904532	-1,07	0,039806
rs4820294	-1,55	0,039840
rs656734	-1,82	0,039875
rs732446	0,20	0,039967
rs155528	-0,95	0,039976
rs941764	-1,58	0,040005
rs4148852	1,06	0,040012
rs7281147	-1,66	0,040022
rs953874	0,91	0,040078
rs2955760	0,59	0,040169
rs2292350	1,60	0,040216
rs8065201	0,16	0,040261
rs3889806	1,12	0,040320
rs10416706	0,13	0,040330
rs545610	1,03	0,040333
rs4802810	0,20	0,040334
rs1446986	1,67	0,040358
rs233511	-0,70	0,040370
rs13051588	1,95	0,040401
rs10843223	1,57	0,040501
rs7575628	1,20	0,040507
rs2368575	-1,61	0,040551
rs4479364	-1,61	0,040551
rs755260	1,36	0,040575
rs2806440	-1,97	0,040585
rs296610	-0,65	0,040656
rs399703	1,24	0,040745
rs6138379	0,71	0,040764
rs2353222	1,41	0,040798
rs11666227	0,83	0,040801
rs8104612	-1,16	0,040843
rs2159444	0,16	0,040947
rs2862783	0,75	0,040947
rs3800795	-1,37	0,040965
rs2242664	-0,94	0,040999
rs644774	-0,94	0,040999
rs644827	-0,94	0,040999
rs660594	-0,94	0,040999
rs6735101	0,58	0,041037
rs17833838	-0,20	0,041050
rs3115560	-1,04	0,041073
rs919209	-0,96	0,041198
rs2613339	-1,27	0,041258

rs8071251	-0,59	0,041262
rs12932862	0,27	0,041263
rs2969123	-1,31	0,041302
rs176648	-1,54	0,041315
rs1005273	-0,56	0,041369
rs1005273	-0,56	0,041369
rs4574552	0,59	0,041497
rs6479395	2,44	0,041506
rs10265223	1,11	0,041552
rs10940346	1,54	0,041592
rs11780823	-1,86	0,041641
rs7780509	-1,29	0,041654
rs7039002	2,26	0,041875
rs12911753	-2,74	0,041918
rs4843429	-1,14	0,042031
rs9640291	-0,91	0,042032
rs2270210	-0,10	0,042089
rs6550471	-0,66	0,042155
rs13172746	1,63	0,042244
rs12913645	-1,01	0,042272
rs2413769	-1,01	0,042272
rs6505300	1,53	0,042294
rs12463274	1,32	0,042333
rs4808564	1,32	0,042333
rs213233	-0,49	0,042348
rs728379	-0,14	0,042353
rs10851421	-1,51	0,042499
rs3132945	-1,01	0,042548
rs4716650	1,55	0,042553
rs10802474	1,54	0,042637
rs10191556	0,76	0,042680
rs7420703	0,76	0,042680
rs4716739	1,21	0,042741
rs12927739	-0,44	0,042762
rs2267366	-1,18	0,042808
rs2076088	0,65	0,042813
rs7249235	1,43	0,042815
rs12912909	1,23	0,042843
rs750142	0,30	0,042890
rs2963059	1,23	0,042909
rs12151335	-1,59	0,042915
rs2239942	0,55	0,042982
rs2101955	0,38	0,043013
rs1056393	0,28	0,043092
rs6509795	0,28	0,043092

rs4798450	-3,33	0,043126
rs12453124	-0,95	0,043220
rs755102	1,46	0,043235
rs1783575	1,19	0,043235
rs8056893	1,60	0,043245
rs11636410	-1,47	0,043277
rs16941017	-1,47	0,043277
rs2413786	-1,47	0,043277
rs3759895	-1,47	0,043277
rs2293683	0,90	0,043283
rs296646	-0,74	0,043300
rs6062344	-1,29	0,043353
rs10495941	-0,87	0,043392
rs2071286	0,47	0,043409
rs1009965	-1,36	0,043525
rs13266503	-1,55	0,043557
rs6739975	1,39	0,043570
rs7350878	-1,79	0,043608
rs2432055	-0,87	0,043615
rs2965214	0,88	0,043625
rs2187557	-1,66	0,043657
rs240067	-1,55	0,043691
rs12115088	-0,37	0,043737
rs1756383	1,72	0,043821
rs1805105	0,35	0,043863
rs3096673	-1,01	0,043898
rs3115552	-1,01	0,043898
rs3115553	-1,01	0,043898
rs3130340	-1,01	0,043898
rs3749966	-1,01	0,043898
rs3864302	-1,01	0,043898
rs6909427	-1,01	0,043898
rs6935269	-1,01	0,043898
rs7751896	-1,01	0,043898
rs9268055	-1,01	0,043898
rs204993	-0,62	0,043937
rs11871523	2,23	0,043970
rs2858331	-0,48	0,043987
rs11154605	1,57	0,043990
rs3810114	-0,36	0,044066
rs4796555	1,64	0,044074
rs4821667	-0,52	0,044090
rs3096681	-1,00	0,044181
rs1574529	-2,38	0,044217
rs10451496	0,89	0,044245

rs2569432	-0,17	0,044260
rs7029539	2,08	0,044271
rs11910387	2,57	0,044289
rs4818237	2,57	0,044289
rs11768251	-0,25	0,044343
rs11652076	1,25	0,044355
rs922749	0,04	0,044358
rs6467157	0,77	0,044376
rs16986351	-0,75	0,044410
rs200482	-0,03	0,044483
rs200484	-0,03	0,044483
rs200490	-0,03	0,044483
rs200501	-0,03	0,044483
rs200948	-0,03	0,044483
rs200953	-0,03	0,044483
rs200989	-0,03	0,044483
rs200990	-0,03	0,044483
rs200995	-0,03	0,044483
rs201002	-0,03	0,044483
rs2747054	-0,03	0,044483
rs370155	-0,03	0,044483
rs1029236	-0,89	0,044502
rs4796360	1,75	0,044512
rs2075760	0,19	0,044566
rs10853347	-2,32	0,044581
rs8188203	1,44	0,044675
rs10068258	-0,97	0,044723
rs876932	-0,97	0,044723
rs9896378	1,24	0,044743
rs2104978	3,01	0,044746
rs816951	-0,27	0,044785
rs6564270	-0,93	0,044810
rs7247514	0,45	0,044851
rs2174097	0,61	0,044870
rs2200559	0,53	0,044902
rs684634	-1,57	0,045002
rs4076033	1,15	0,045010
rs11737953	-1,02	0,045019
rs6814637	-0,75	0,045088
rs7939444	-0,33	0,045105
rs7213247	1,86	0,045131
rs1039797	1,41	0,045194
rs6350	-1,64	0,045324
rs4324213	0,69	0,045366
rs1042098	0,78	0,045442

rs4580814	1,03	0,045521
rs1128723	0,27	0,045530
rs4758233	-0,25	0,045532
rs11719906	-0,67	0,045540
rs8113506	-0,15	0,045547
rs2074840	0,54	0,045610
rs240068	-1,59	0,045628
rs169851	-1,17	0,045654
rs11761679	-1,27	0,045716
rs17151970	0,27	0,045735
rs1939641	-0,55	0,045859
rs12190030	-1,33	0,045884
rs2244158	-0,78	0,045887
rs7513496	-1,45	0,045899
rs7526237	-1,45	0,045899
rs9920421	-1,54	0,045901
rs16850073	-1,21	0,045928
rs10111	0,56	0,046003
rs464694	-0,15	0,046116
rs6738639	0,28	0,046159
rs6758509	-2,30	0,046192
rs6917366	-0,18	0,046199
rs11575687	-0,85	0,046300
rs2470450	-1,42	0,046307
rs3889130	-0,74	0,046322
rs6511429	-0,74	0,046322
rs2269913	0,42	0,046348
rs4808608	1,32	0,046373
rs9468379	-1,03	0,046395
rs9468379	-1,03	0,046395
rs1408320	0,95	0,046432
rs6730178	-1,88	0,046449
rs12517292	0,67	0,046476
rs4145361	1,74	0,046495
rs11085825	0,87	0,046645
rs12962844	-2,27	0,046678
rs3127482	1,38	0,046681
rs6903016	-0,12	0,046891
rs2399786	-1,31	0,046895
rs6686	-1,31	0,046950
rs12541726	-2,44	0,046987
rs1316371	1,06	0,046999
rs752335	1,06	0,046999
rs752336	1,06	0,046999
rs5000660	-0,49	0,047023

rs7606684	0,06	0,047067
rs11965538	-1,42	0,047129
rs7887	0,81	0,047248
rs10479054	1,42	0,047262
rs4843180	0,87	0,047265
rs2523457	0,41	0,047285
rs563726	-1,67	0,047288
rs7187438	1,16	0,047370
rs1008805	-1,25	0,047440
rs713835	-1,52	0,047499
rs2325788	0,93	0,047539
rs6888605	2,28	0,047660
rs2857161	-1,12	0,047712
rs10903009	-0,33	0,047714
rs2657940	-0,29	0,047736
rs10401344	1,29	0,047749
rs17173197	-1,04	0,047773
rs218669	0,25	0,047782
rs6113094	-0,35	0,047825
rs1332057	1,44	0,047847
rs139895	-1,07	0,047854
rs3860879	0,94	0,047941
rs9366694	-0,11	0,048034
rs4915770	1,06	0,048053
rs11041820	0,61	0,048090
rs276984	0,16	0,048110
rs9630874	-0,98	0,048181
rs10790239	0,38	0,048270
rs11465897	0,81	0,048311
rs12372906	0,63	0,048384
rs2366559	1,15	0,048389
rs2975758	-1,75	0,048435
rs11593647	-0,50	0,048449
rs6753628	-1,03	0,048490
rs7107322	0,00	0,048508
rs9935025	1,53	0,048544
rs2890103	1,45	0,048564
rs2305556	1,84	0,048662
rs11620733	-0,90	0,048673
rs8083385	0,64	0,048734
rs2844498	0,00	0,048776
rs9817842	0,96	0,048809
rs1384507	0,16	0,048841
rs7254083	-0,77	0,048868
rs250425	1,74	0,048912

rs941672	1,15	0,048934
rs1018100	0,27	0,048973
rs1805562	-1,06	0,049063
rs1805564	-1,06	0,049063
rs12670999	-0,99	0,049127
rs10773020	-3,39	0,049142
rs11057310	-3,39	0,049142
rs11057312	-3,39	0,049142
rs2292702	-3,39	0,049142
rs5998881	1,44	0,049170
rs17706531	0,88	0,049218
rs12730072	1,77	0,049286
rs2233966	0,18	0,049319
rs1728394	-1,31	0,049322
rs10456362	-1,39	0,049362
rs1419183	-1,39	0,049362
rs1679709	-1,39	0,049362
rs1778508	-1,39	0,049362
rs11248931	0,54	0,049383
rs11641742	0,54	0,049383
rs2071915	0,54	0,049383
rs12203410	-1,22	0,049499
rs12919	0,23	0,049540
rs2302443	0,23	0,049540
rs2302445	0,23	0,049540
rs3087905	0,23	0,049540
rs7803794	0,23	0,049540
rs11084849	-0,56	0,049613
rs1478457	-1,22	0,049618
rs2884017	-1,42	0,049619
rs10403164	0,34	0,049626
rs1699487	1,89	0,049653
rs1699487	1,89	0,049653
rs8084409	-2,27	0,049711
rs9304684	1,39	0,049730
rs7772245	-0,49	0,049749
rs6548154	-0,39	0,049916
rs7006527	-1,85	0,049920
rs10412187	0,34	0,049926
rs7283002	1,40	0,049956
rs2281558	1,71	0,049960
