# Supplementary material for: The transcriptome-wide association search for genes and genetic variants which associate with BMI and gestational weight gain in women with type 1 diabetes
Source: Mol Med. 2021 Jan 20;27:6. doi: 10.1186/s10020-020-00266-z (PMC7818927; doi:10.1186/s10020-020-00266-z)
Supplement: Supplementary file 6 — Additional file 6: Figure S1a. The LD analysis for GPN3 gene. b. The LD analysis for PMS2P3 gene. c. The LD analysis for STAG3L1 gene. [file 10020_2020_266_MOESM6_ESM.zip › Fig S1c.pdf]

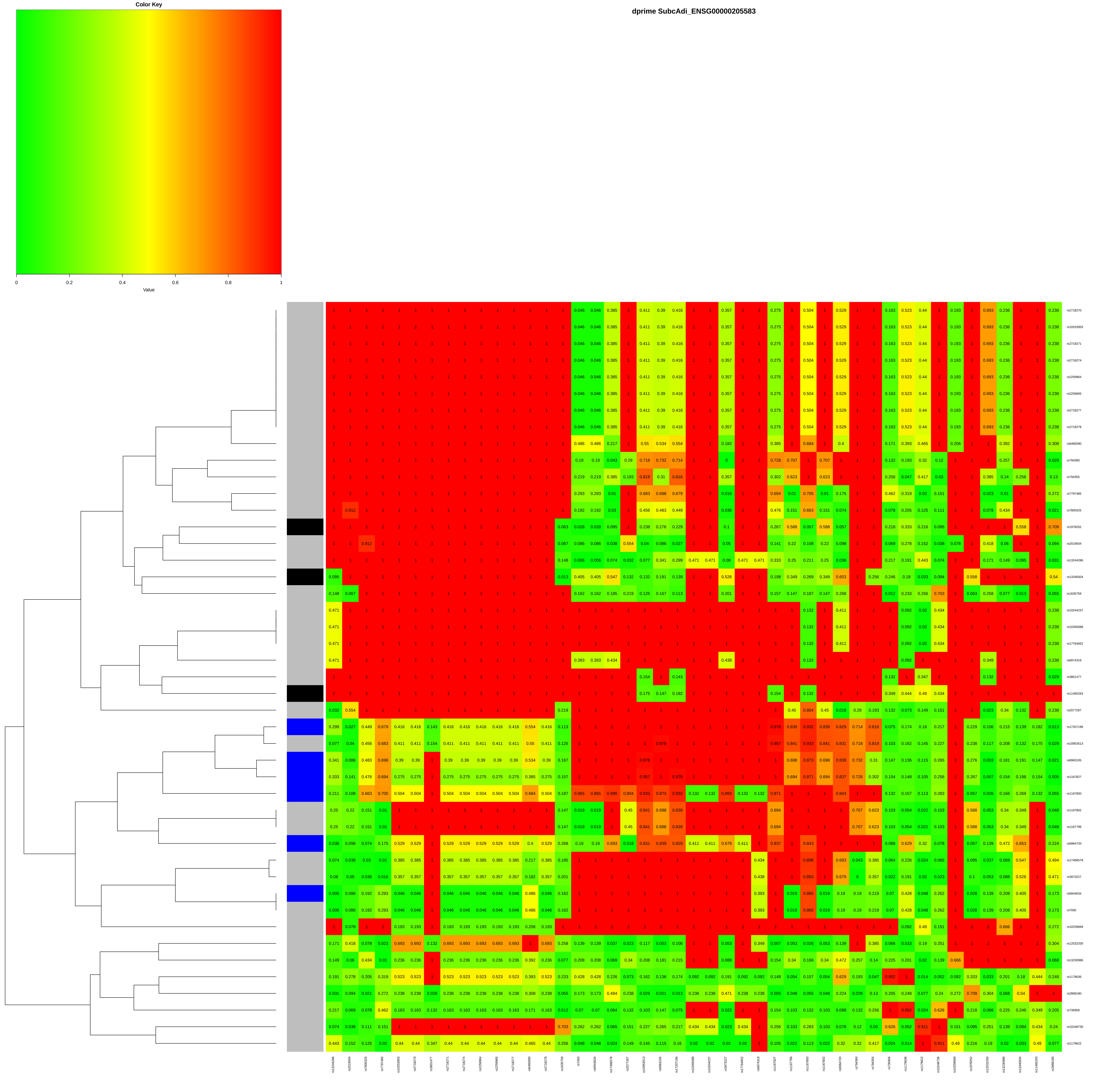
dprime SubcAdi_ENSG00000205583
Color Key
0
0.2
0.4
0.6
0.8
1
Value

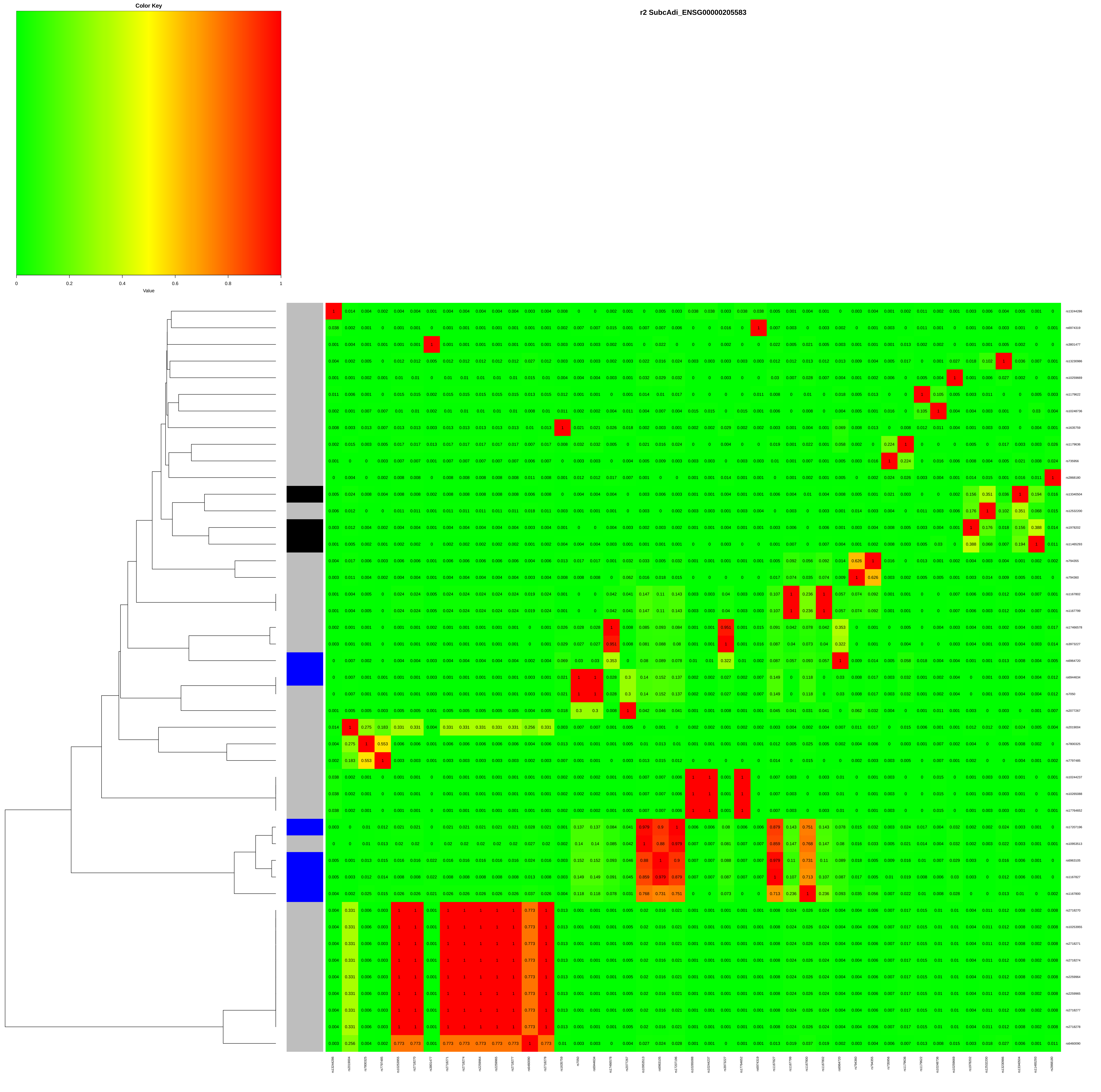
r2 SubcAdi_ENSG00000205583
Color Key
0
0.2
0.4
0.6
0.8
1
Value
