# Supplementary figures and images for: The transcriptome-wide association search for genes and genetic variants which associate with BMI and gestational weight gain in women with type 1 diabetes
Source: Mol Med. 2021 Jan 20;27:6. doi: 10.1186/s10020-020-00266-z (PMC7818927; doi:10.1186/s10020-020-00266-z)

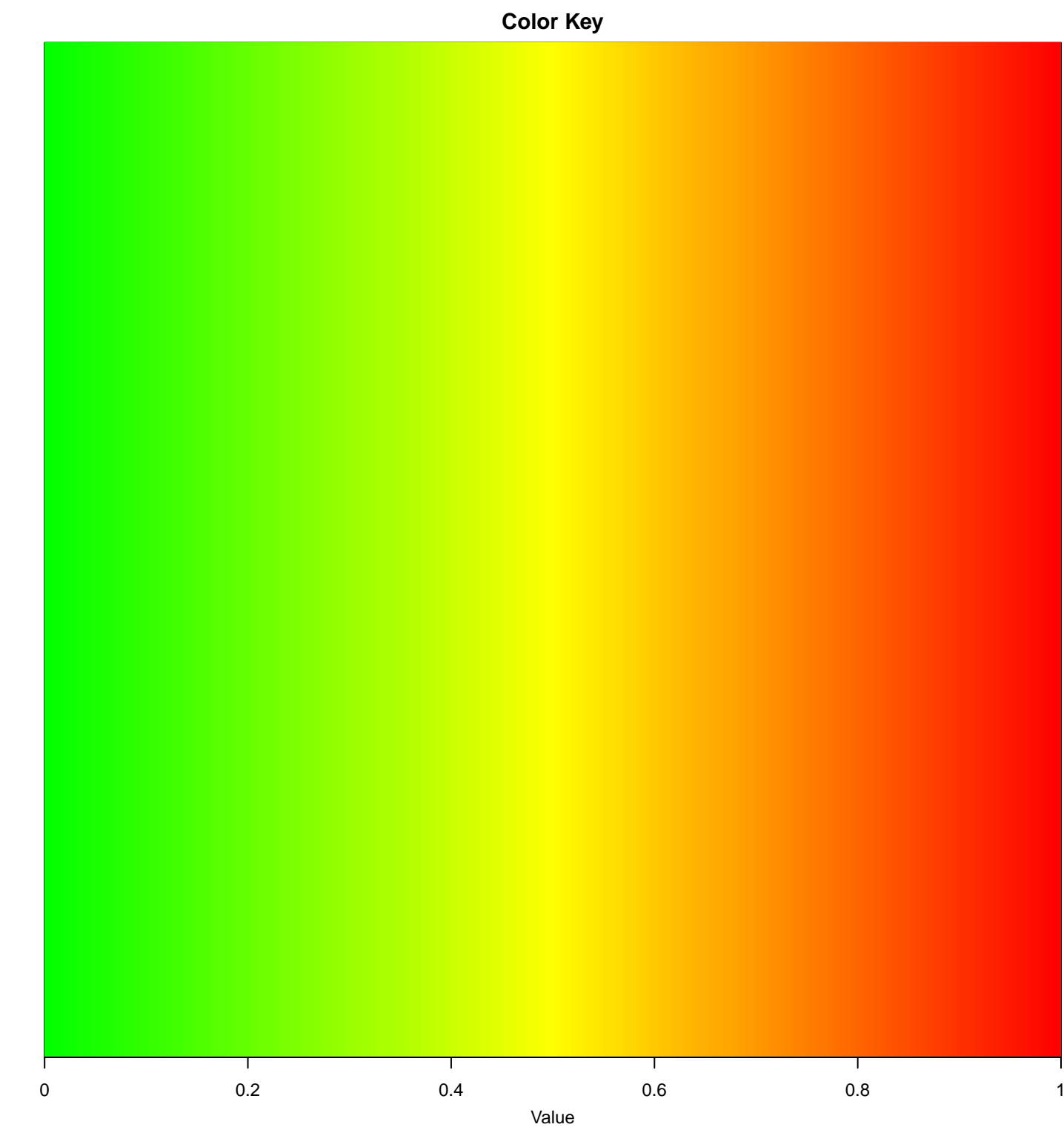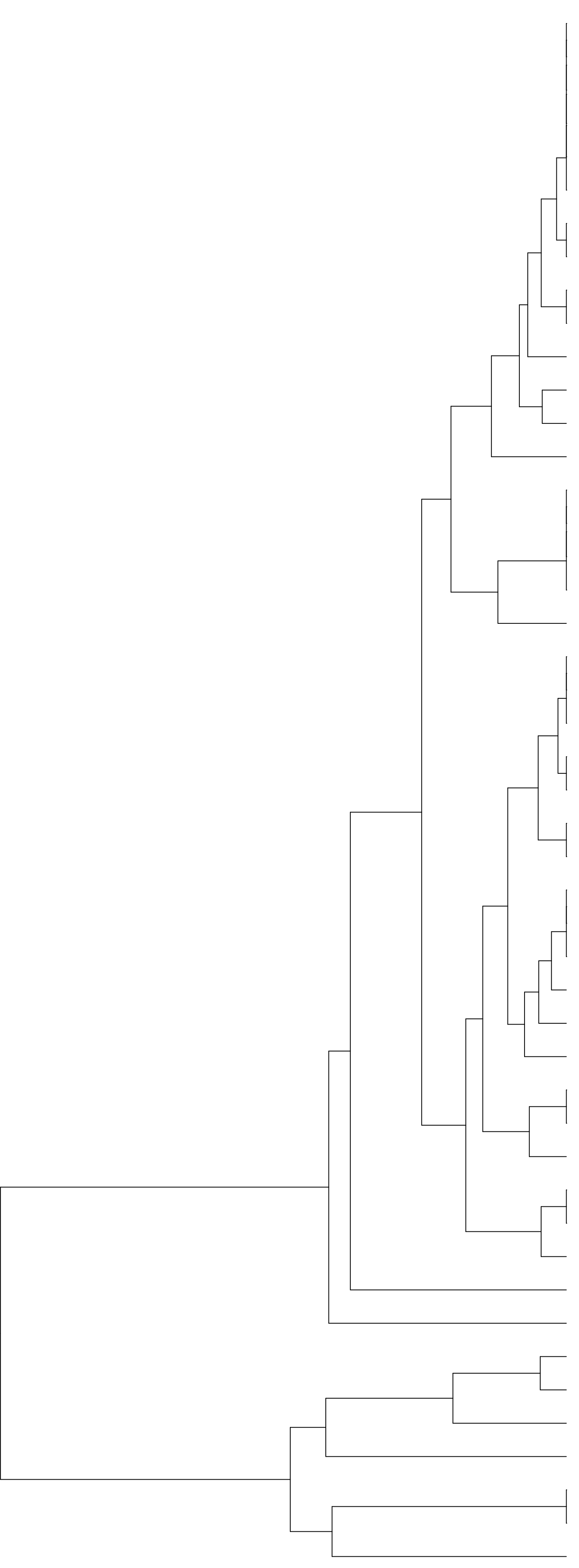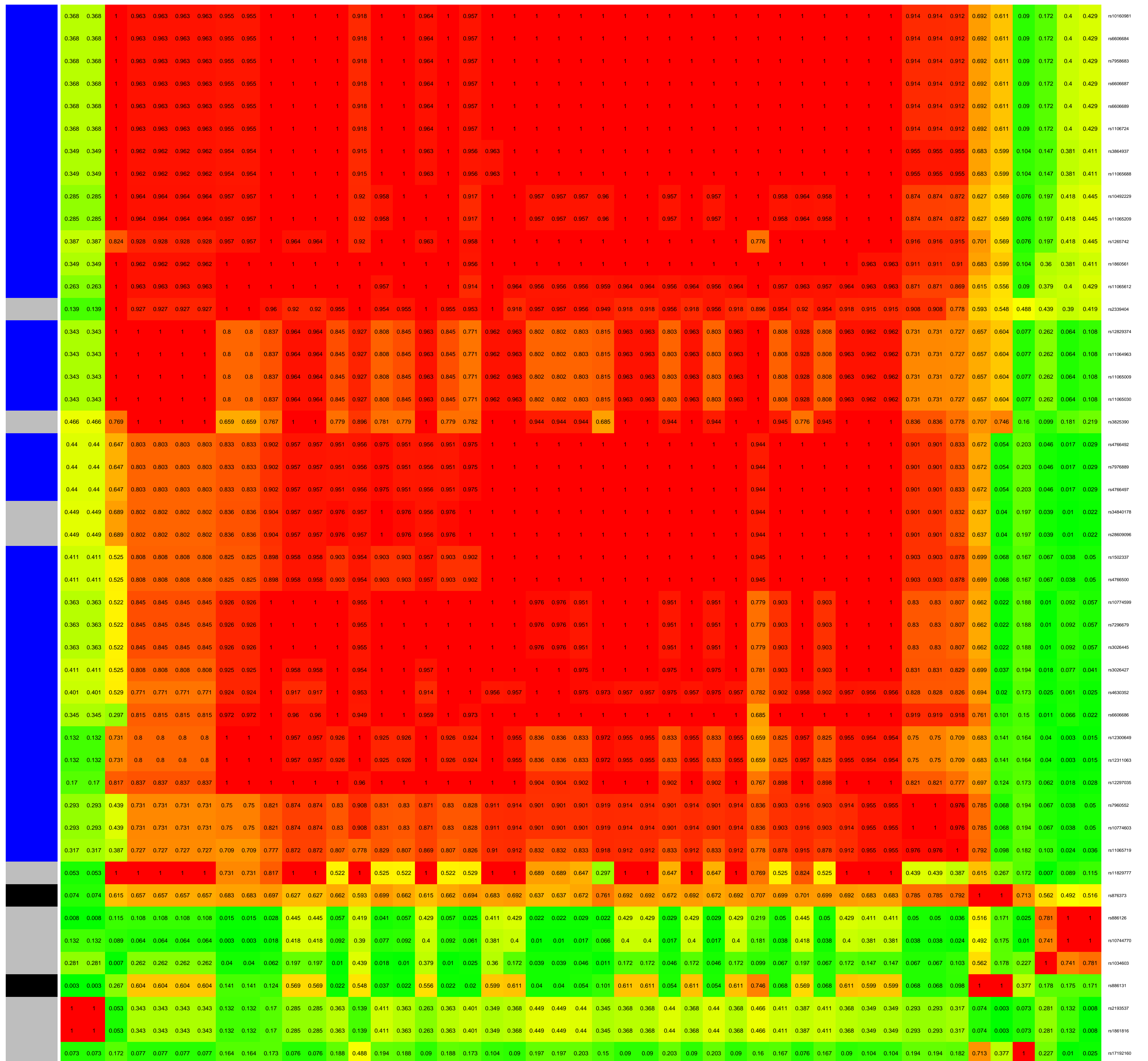

Supplement: Supplementary file 6 — Additional file 6: Figure S1a. The LD analysis for GPN3 gene. b. The LD analysis for PMS2P3 gene. c. The LD analysis for STAG3L1 gene. [file 10020_2020_266_MOESM6_ESM.zip › Fig S1a.pdf]

Color Key

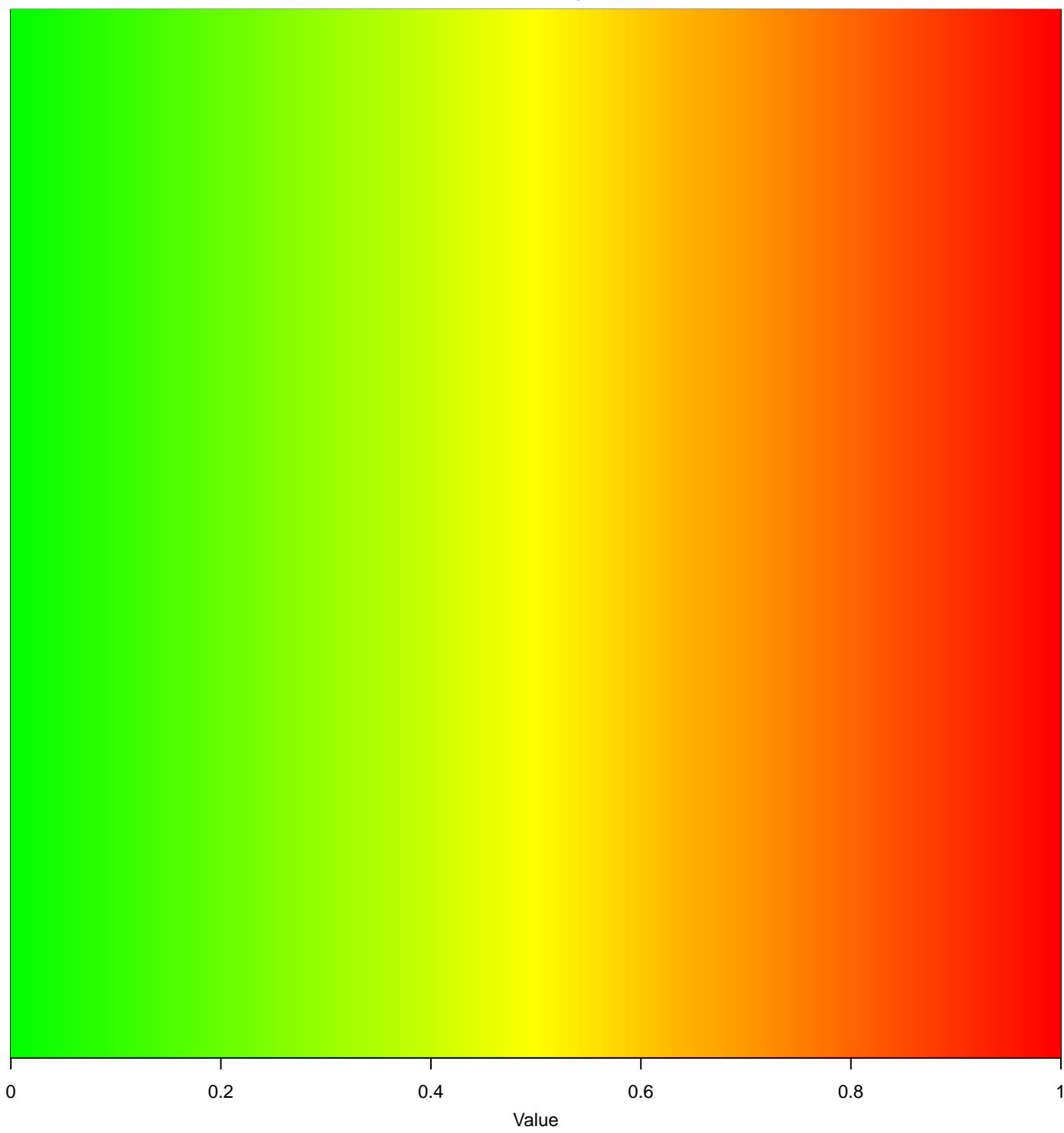

dprime SubcAdi\_ENSG00000127957

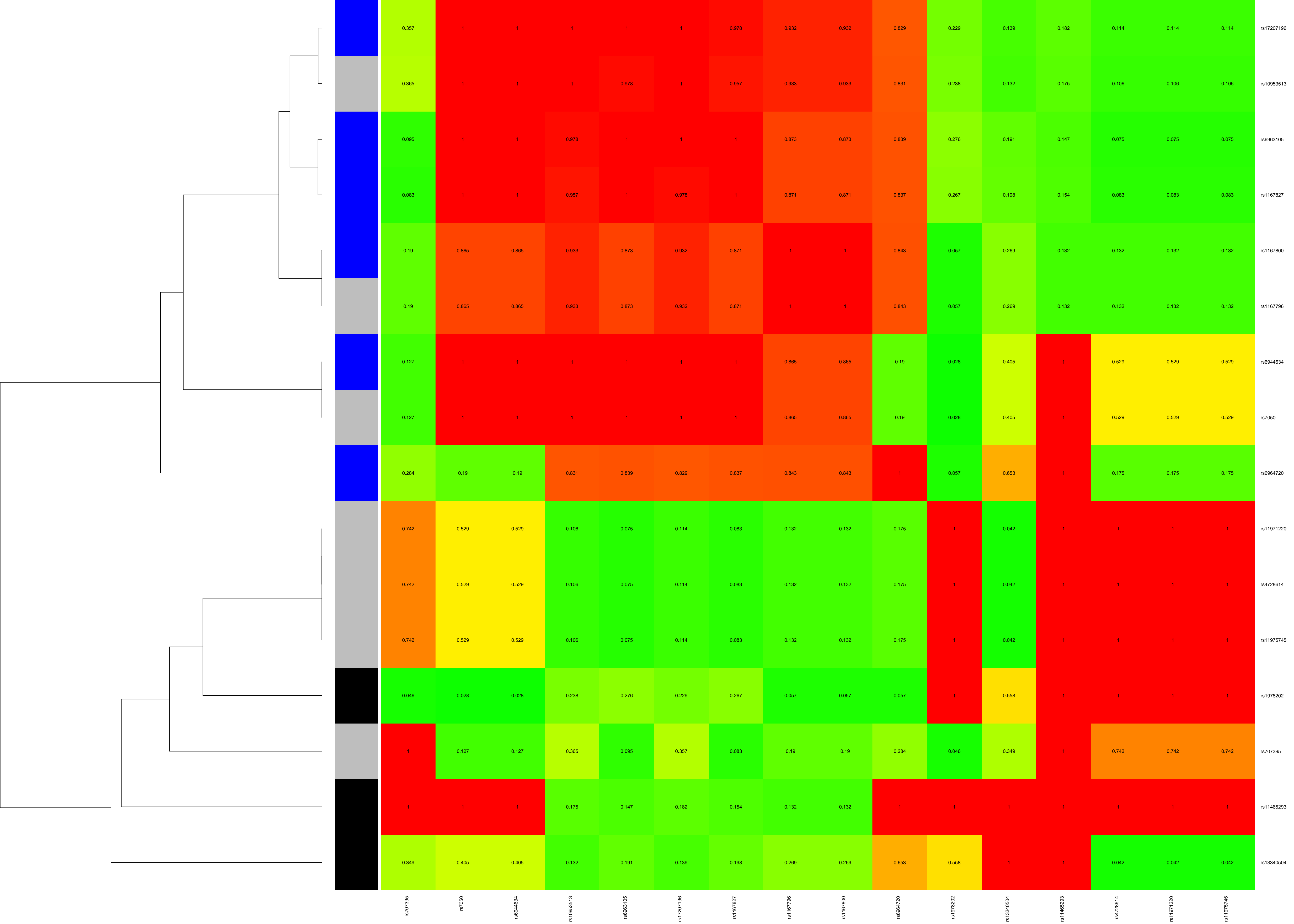

Color Key

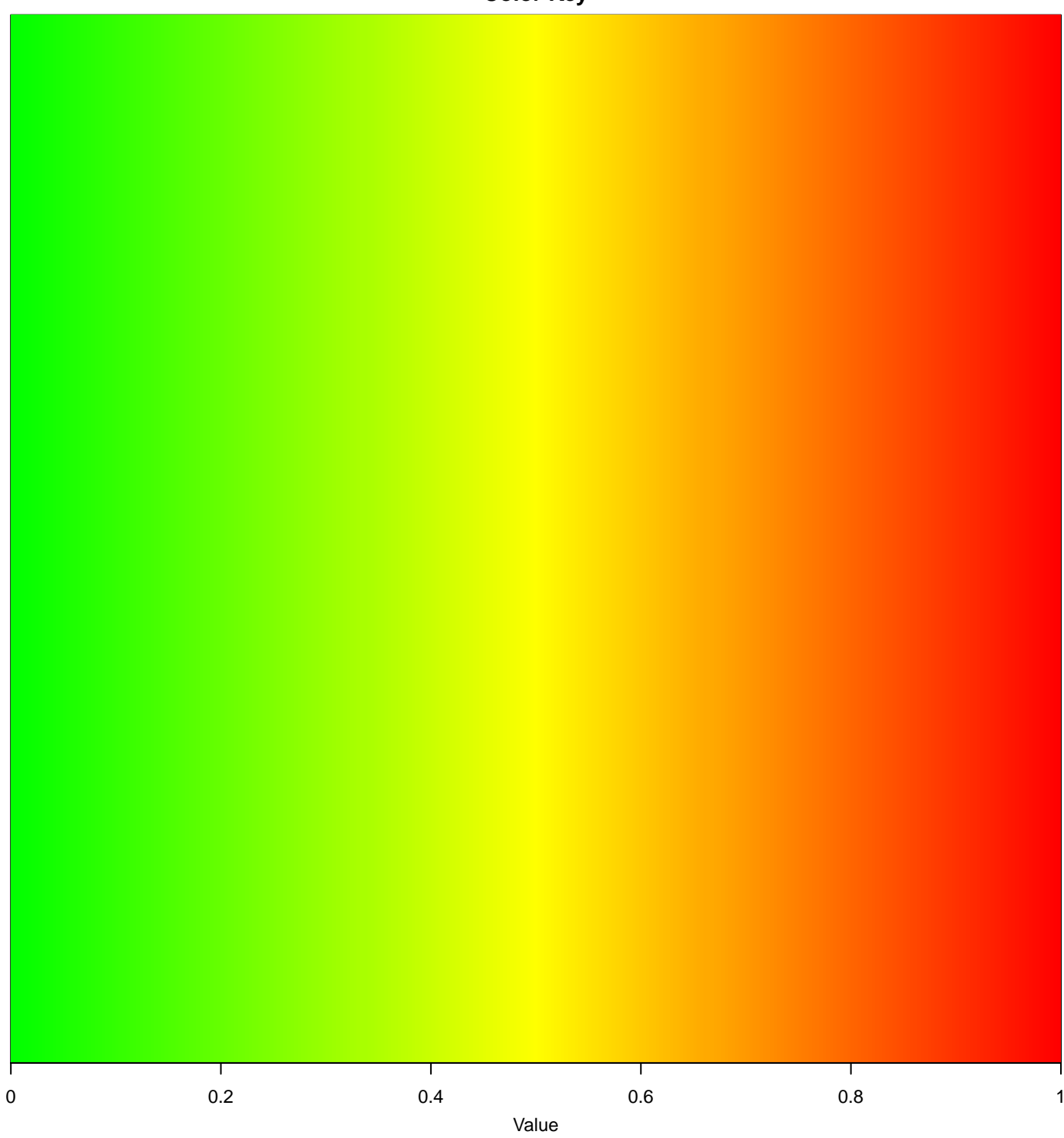

r2 SubcAdi\_ENSG00000127957

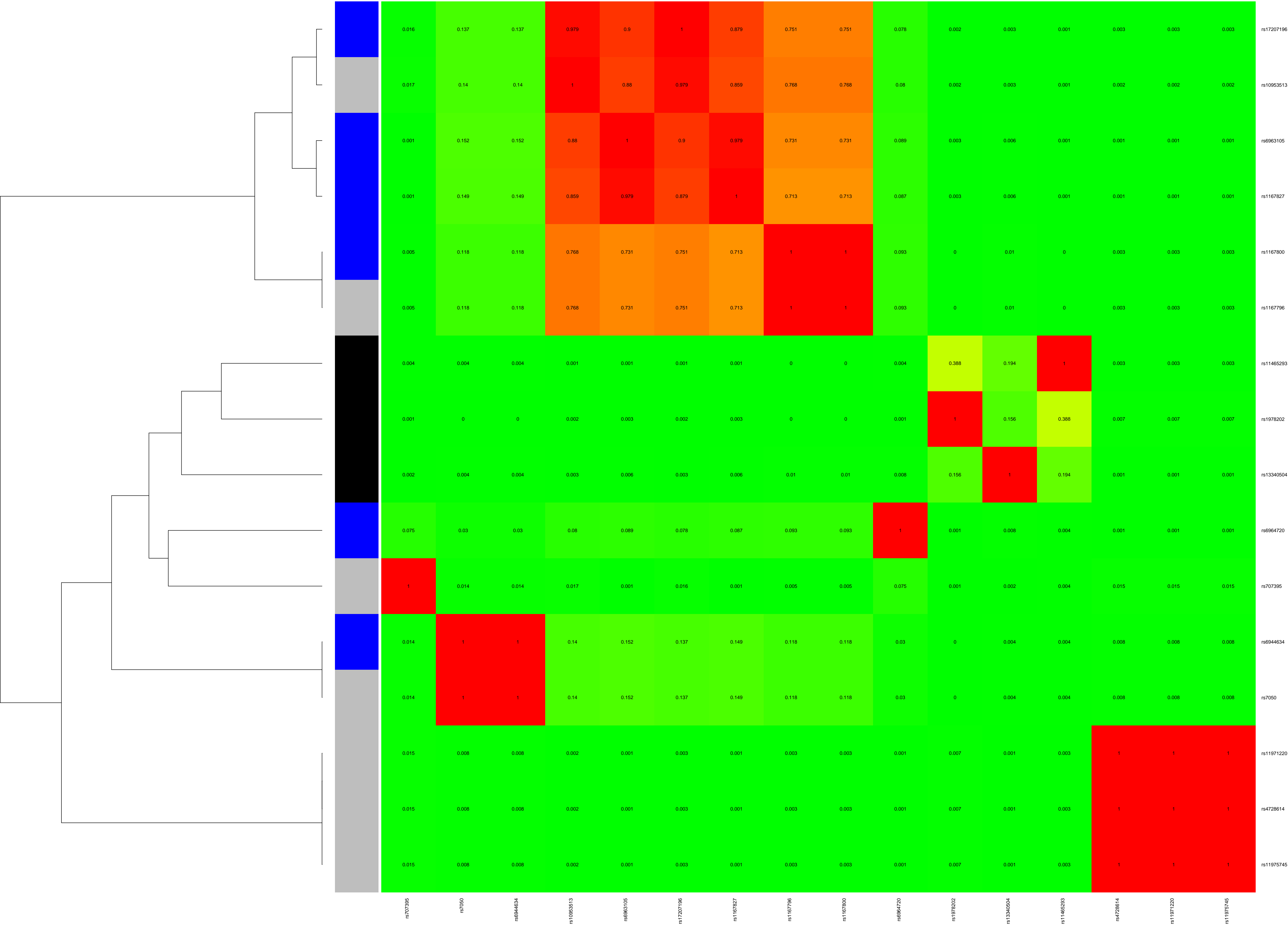

Supplement: Supplementary file 6 — Additional file 6: Figure S1a. The LD analysis for GPN3 gene. b. The LD analysis for PMS2P3 gene. c. The LD analysis for STAG3L1 gene. [file 10020_2020_266_MOESM6_ESM.zip › Fig S1b.pdf]

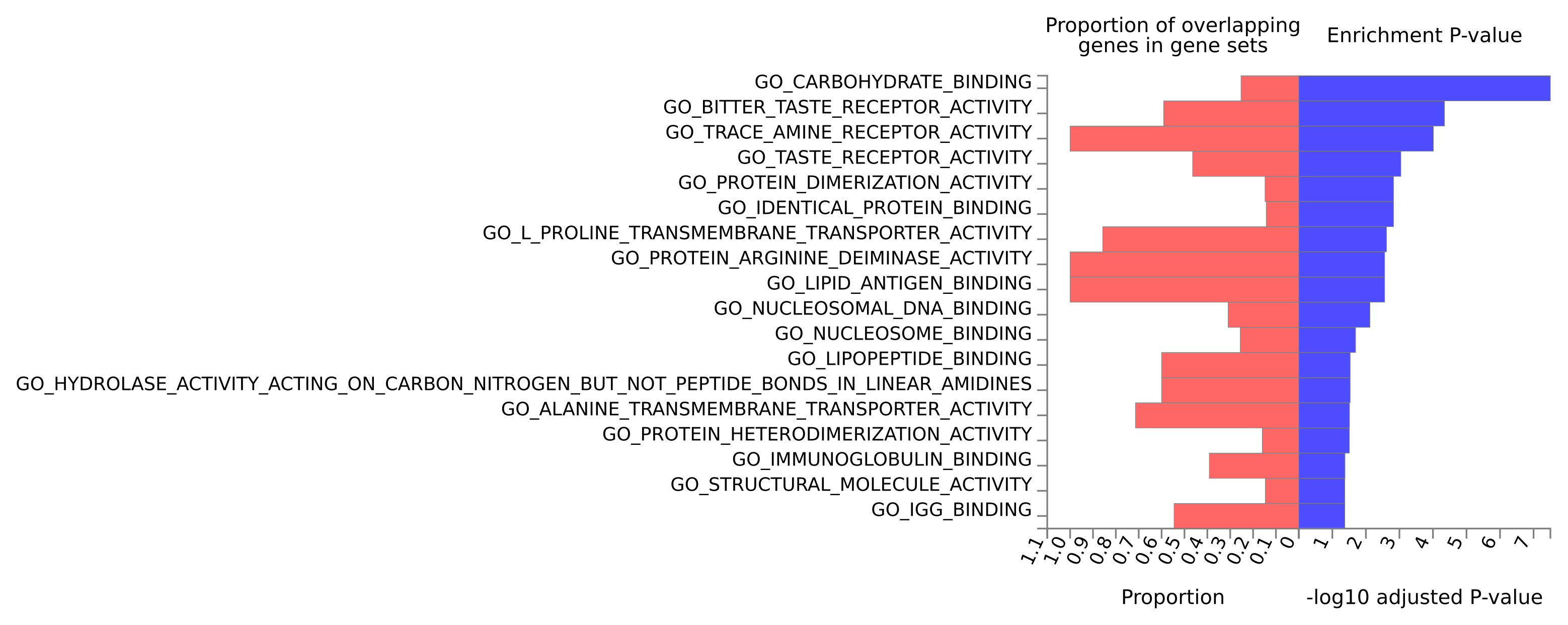

Supplement: Supplementary file 10 — Additional file 10: Figure S2. The KEGG analysis on GWG associated variants in BMI associated genes in T2D&ARIC cohorts. [file 10020_2020_266_MOESM10_ESM.png]

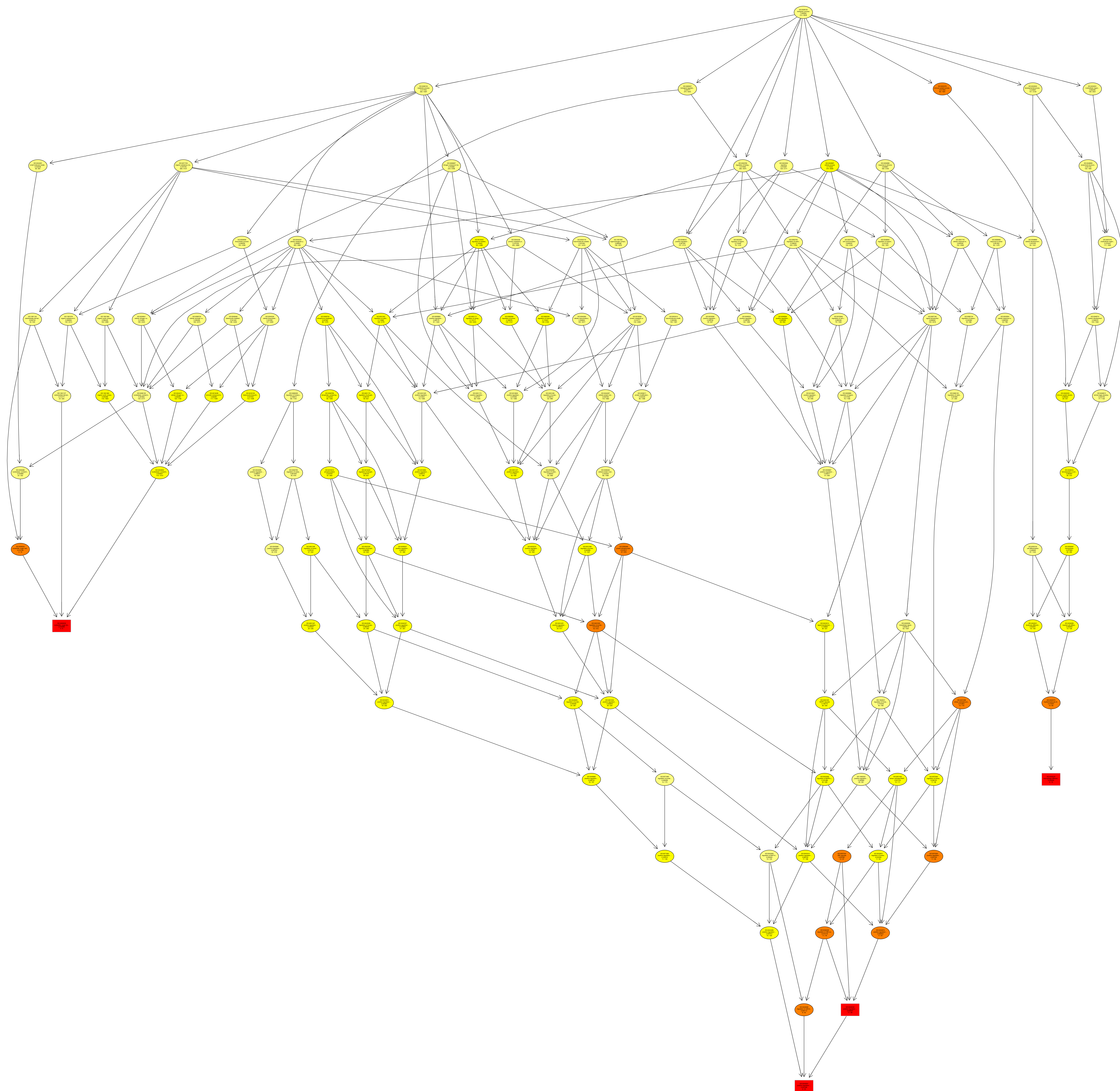

Supplement: Supplementary file 13 — Additional file 13: Figure S3a. Go Enrichment analysis on GWG only associated genes in the Giant cohort. b. Go Enrichment analysis on the overlap between BMI and GWG genes in the Giant cohort. [file 10020_2020_266_MOESM13_ESM.zip › Fig S3a.pdf]

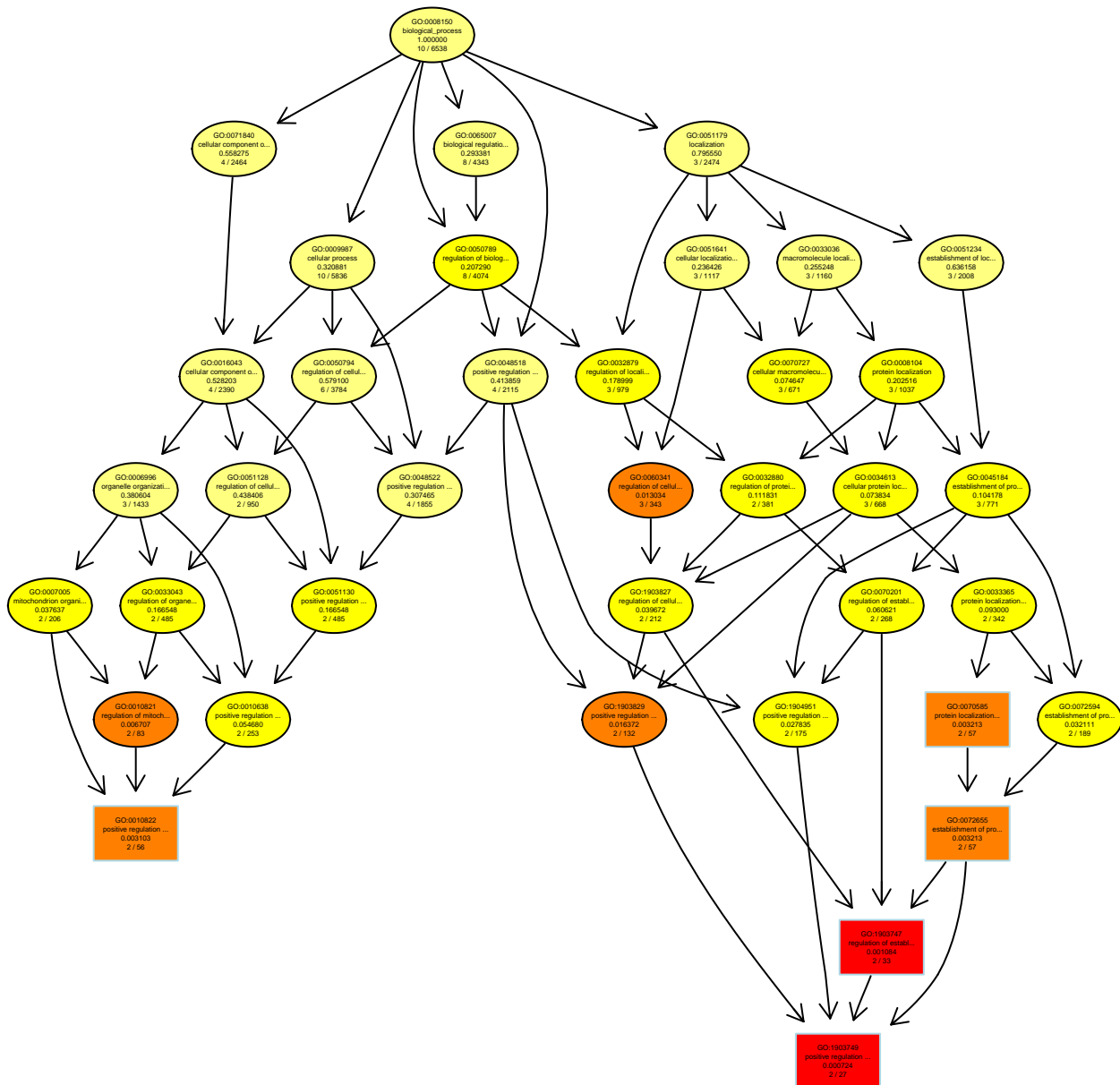

Supplement: Supplementary file 13 — Additional file 13: Figure S3a. Go Enrichment analysis on GWG only associated genes in the Giant cohort. b. Go Enrichment analysis on the overlap between BMI and GWG genes in the Giant cohort. [file 10020_2020_266_MOESM13_ESM.zip › FigS3b.pdf]

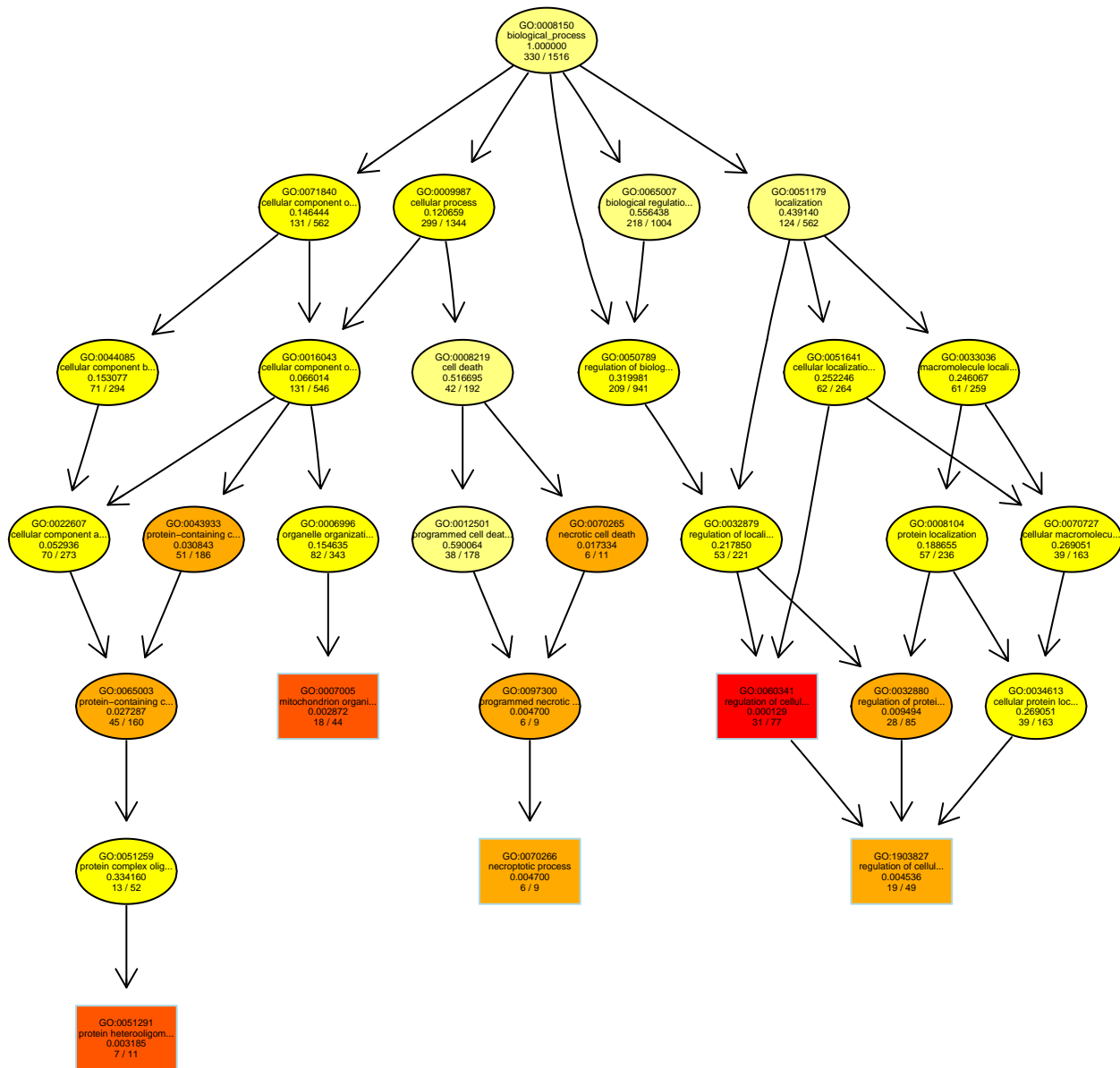

Supplement: Supplementary file 15 — Additional file 15: Figure S4a. Go Enrichment analysis on GWG only associated genes in T2D and ARIC cohorts. b. Go Enrichment analysis on the overlap between BMI and GWG genes in T2D and ARIC cohorts. [file 10020_2020_266_MOESM15_ESM.zip › FigS4a.pdf]

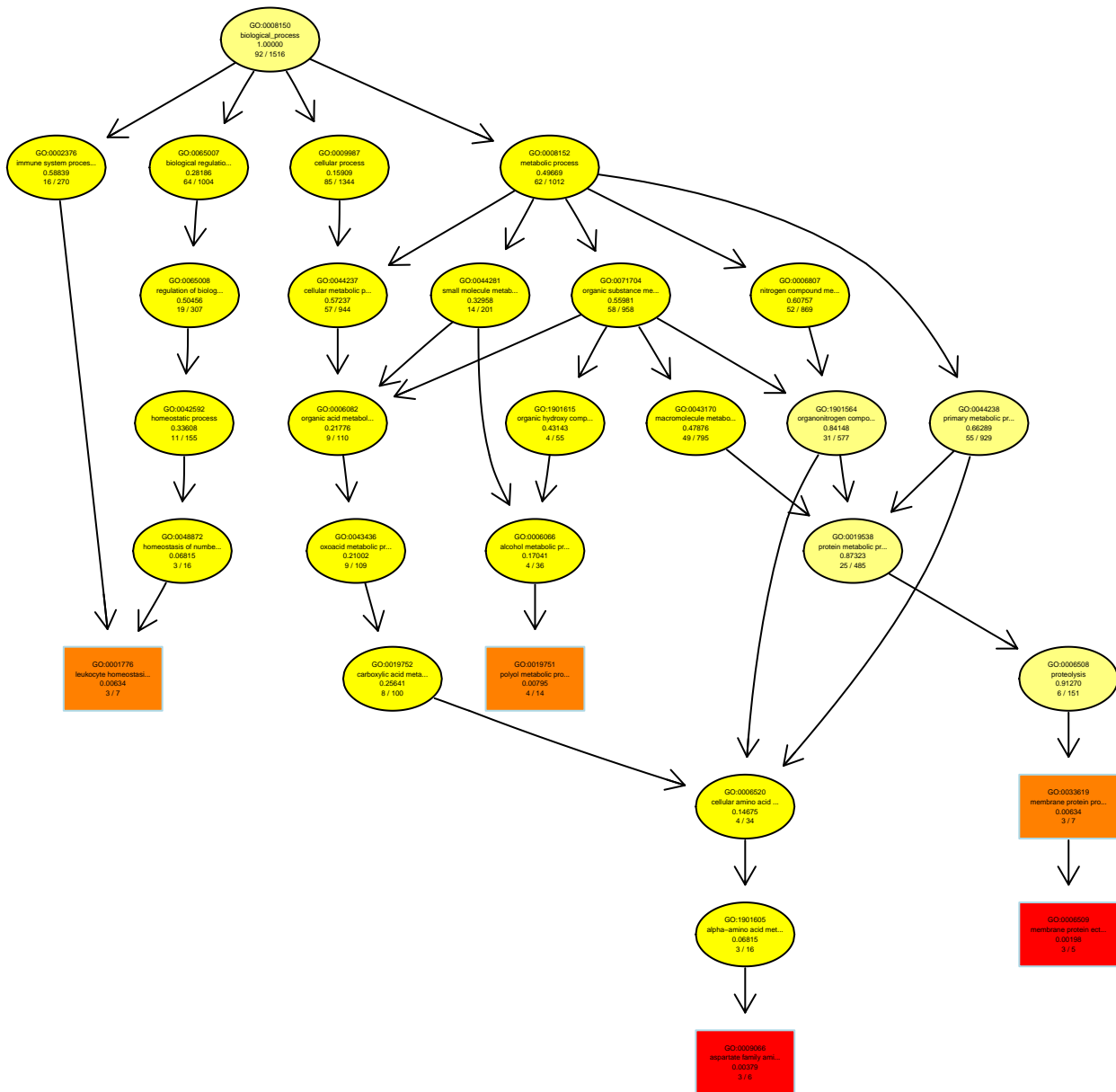

Supplement: Supplementary file 15 — Additional file 15: Figure S4a. Go Enrichment analysis on GWG only associated genes in T2D and ARIC cohorts. b. Go Enrichment analysis on the overlap between BMI and GWG genes in T2D and ARIC cohorts. [file 10020_2020_266_MOESM15_ESM.zip › FigS4b.pdf]
