# Supplementary material for: The transcriptome-wide association search for genes and genetic variants which associate with BMI and gestational weight gain in women with type 1 diabetes
Source: Mol Med. 2021 Jan 20;27:6. doi: 10.1186/s10020-020-00266-z (PMC7818927; doi:10.1186/s10020-020-00266-z)
Supplement: Supplementary file 7 — Additional file 7: Table S4a.The results of PrediXcan on BMI in T2D cohort in Subcutaneous Adipose Tissue. b. The results of PrediXcan on BMI in T2D cohort in Visceral Adipose Tissue. c. The results of PrediXcan on BMI in ARIC cohort in Subcutaneous Adipose Tissue. d. The results of PrediXcan on BMI in ARIC cohort in Visceral Adipose Tissue. [file 10020_2020_266_MOESM7_ESM.zip › Table S4a.pdf]

| geneID | logFC | AveExpr | t | P.Value | adj.P.Val |
|---|---|---|---|---|---|
| ENSG0000 | -0,010367 | -0,389498 | -4,640994 | 4,31E-06 | 0,027757 |
| ENSG0000 | 0,005434 | 0,081972 | 4,544113 | 6,75E-06 | 0,027757 |
| ENSG0000 | 0,008107 | 0,288143 | 4,395054 | 1,32E-05 | 0,036273 |
| ENSG0000 | -0,008047 | 0,089479 | -4,29015 | 2,10E-05 | 0,04319 |
| ENSG0000 | -0,004144 | 0,647951 | -4,131732 | 4,14E-05 | 0,060763 |
| ENSG0000 | -0,00923 | -1,232064 | -4,115876 | 4,43E-05 | 0,060763 |
| ENSG0000 | 0,005407 | 0,391538 | 4,012486 | 6,81E-05 | 0,080113 |
| ENSG0000 | 0,00709 | -0,972179 | 3,95725 | 8,54E-05 | 0,087896 |
| ENSG0000 | -0,005016 | -0,005772 | -3,91849 | 1,00E-04 | 0,091427 |
| ENSG0000 | -0,009799 | -1,315013 | -3,859481 | 0,000127 | 0,104264 |
| ENSG0000 | 0,002807 | 0,243103 | 3,833888 | 0,00014 | 0,104366 |
| ENSG0000 | -0,007079 | 2,143726 | -3,808699 | 0,000155 | 0,104366 |
| ENSG0000 | 0,005554 | -0,176715 | 3,792938 | 0,000165 | 0,104366 |
| ENSG0000 | -0,009229 | -0,119117 | -3,759294 | 0,000188 | 0,11055 |
| ENSG0000 | -0,001454 | -0,027617 | -3,733571 | 0,000208 | 0,114032 |
| ENSG0000 | 0,007035 | 0,748125 | 3,691729 | 0,000244 | 0,121365 |
| ENSG0000 | 0,006679 | 0,738422 | 3,684935 | 0,000251 | 0,121365 |
| ENSG0000 | -0,005079 | 0,293042 | -3,657259 | 0,000279 | 0,121879 |
| ENSG0000 | 0,007779 | -1,346614 | 3,654716 | 0,000281 | 0,121879 |
| ENSG0000 | 0,003514 | -0,182877 | 3,638097 | 0,0003 | 0,123333 |
| ENSG0000 | -0,007686 | 0,213575 | -3,572826 | 0,000383 | 0,140248 |
| ENSG0000 | -0,012675 | -1,913372 | -3,542351 | 0,000429 | 0,140248 |
| ENSG0000 | 0,009541 | 1,4594 | 3,542324 | 0,000429 | 0,140248 |
| ENSG0000 | -0,002707 | -0,4985 | -3,528607 | 0,000452 | 0,140248 |
| ENSG0000 | -0,008663 | -0,215037 | -3,515071 | 0,000475 | 0,140248 |
| ENSG0000 | 0,005133 | 0,385365 | 3,507351 | 0,000488 | 0,140248 |
| ENSG0000 | -0,008445 | -0,463762 | -3,507111 | 0,000489 | 0,140248 |
| ENSG0000 | -0,009152 | 4,187076 | -3,50283 | 0,000497 | 0,140248 |
| ENSG0000 | -0,002623 | 0,134267 | -3,501253 | 0,0005 | 0,140248 |
| ENSG0000 | -0,003624 | -0,864507 | -3,494931 | 0,000511 | 0,140248 |
| ENSG0000 | 0,0037 | -0,347987 | 3,474658 | 0,000551 | 0,141359 |
| ENSG0000 | 0,009224 | -0,108636 | 3,468033 | 0,000564 | 0,141359 |
| ENSG0000 | 0,001853 | 0,236866 | 3,459611 | 0,000582 | 0,141359 |
| ENSG0000 | -0,007927 | 0,604869 | -3,458487 | 0,000584 | 0,141359 |
| ENSG0000 | 0,006436 | 0,396966 | 3,439042 | 0,000627 | 0,147353 |
| ENSG0000 | 0,002502 | -0,1062 | 3,382353 | 0,000768 | 0,171786 |
| ENSG0000 | -0,003438 | -0,4567 | -3,380864 | 0,000772 | 0,171786 |
| ENSG0000 | -0,007686 | 0,187875 | -3,360242 | 0,000831 | 0,1742 |
| ENSG0000 | -0,002463 | 0,859238 | -3,357522 | 0,000839 | 0,1742 |
| ENSG0000 | -0,005797 | -0,456968 | -3,351168 | 0,000858 | 0,1742 |
| ENSG0000 | -0,003634 | -0,627118 | -3,348037 | 0,000868 | 0,1742 |
| ENSG0000 | -0,008816 | -0,990198 | -3,335099 | 0,000908 | 0,178004 |
| ENSG0000 | -0,000471 | 0,100837 | -3,316116 | 0,000971 | 0,185872 |
| ENSG0000 | -0,002456 | -0,02739 | -3,300185 | 0,001027 | 0,190163 |
| ENSG0000 | 0,003519 | 0,190025 | 3,296612 | 0,00104 | 0,190163 |
| ENSG0000 | 0,006742 | -0,075875 | 3,27899 | 0,001106 | 0,191505 |
| ENSG0000 | 0,0045 | -0,365837 | 3,272937 | 0,001129 | 0,191505 |
| ENSG0000 | -0,00541 | -0,88867 | -3,267168 | 0,001152 | 0,191505 |

| | | | | | |
|---|---|---|---|---|---|
| ENSG0000 | 0,015204 | 0,49303 | 3,260699 | 0,001178 | 0,191505 |
| ENSG0000 | -0,006171 | -0,124502 | -3,259522 | 0,001183 | 0,191505 |
| ENSG0000 | 0,001129 | 0,39435 | 3,254834 | 0,001202 | 0,191505 |
| ENSG0000 | 0,010374 | -0,693761 | 3,235042 | 0,001287 | 0,191505 |
| ENSG0000 | -0,00276 | 0,39491 | -3,233997 | 0,001292 | 0,191505 |
| ENSG0000 | 0,009027 | 0,41273 | 3,2243 | 0,001335 | 0,191505 |
| ENSG0000 | -0,006617 | 0,02179 | -3,21049 | 0,0014 | 0,191505 |
| ENSG0000 | 0,001739 | 0,027248 | 3,209024 | 0,001407 | 0,191505 |
| ENSG0000 | -0,008689 | 0,239509 | -3,201747 | 0,001442 | 0,191505 |
| ENSG0000 | 0,005733 | -0,213045 | 3,200676 | 0,001448 | 0,191505 |
| ENSG0000 | -0,005427 | -0,575956 | -3,197425 | 0,001464 | 0,191505 |
| ENSG0000 | -0,002531 | 0,124947 | -3,197163 | 0,001465 | 0,191505 |
| ENSG0000 | 0,002334 | -0,197193 | 3,194932 | 0,001476 | 0,191505 |
| ENSG0000 | -0,003432 | 0,111042 | -3,193525 | 0,001483 | 0,191505 |
| ENSG0000 | -0,004249 | 0,050594 | -3,188997 | 0,001506 | 0,191505 |
| ENSG0000 | -0,002508 | 0,277298 | -3,188956 | 0,001507 | 0,191505 |
| ENSG0000 | -0,005561 | 0,105027 | -3,184865 | 0,001528 | 0,191505 |
| ENSG0000 | -0,014508 | 0,835174 | -3,182732 | 0,001539 | 0,191505 |
| ENSG0000 | -0,014136 | 0,074299 | -3,178882 | 0,001559 | 0,191505 |
| ENSG0000 | 0,007834 | 0,50455 | 3,168683 | 0,001614 | 0,195315 |
| ENSG0000 | 0,006492 | -0,372629 | 3,161851 | 0,001651 | 0,196977 |
| ENSG0000 | 0,003583 | -0,741708 | 3,150175 | 0,001718 | 0,201952 |
| ENSG0000 | 0,004393 | 0,485082 | 3,129439 | 0,001841 | 0,204295 |
| ENSG0000 | -0,007694 | 0,49806 | -3,126293 | 0,001861 | 0,204295 |
| ENSG0000 | -0,008174 | 0,542021 | -3,126001 | 0,001863 | 0,204295 |
| ENSG0000 | 0,002552 | 0,579404 | 3,125777 | 0,001864 | 0,204295 |
| ENSG0000 | -0,005087 | 0,227692 | -3,12343 | 0,001879 | 0,204295 |
| ENSG0000 | 0,003465 | 0,414367 | 3,117624 | 0,001916 | 0,204295 |
| ENSG0000 | 0,005877 | -0,310347 | 3,116813 | 0,001921 | 0,204295 |
| ENSG0000 | 0,004666 | 0,177596 | 3,114405 | 0,001936 | 0,204295 |
| ENSG0000 | -0,00164 | 0,104196 | -3,104077 | 0,002004 | 0,208761 |
| ENSG0000 | 0,004345 | 0,485488 | 3,092254 | 0,002084 | 0,214398 |
| ENSG0000 | 0,004653 | -0,667426 | 3,088028 | 0,002113 | 0,214734 |
| ENSG0000 | -0,00284 | -0,158018 | -3,076981 | 0,002192 | 0,21704 |
| ENSG0000 | -0,007072 | -0,557783 | -3,075979 | 0,002199 | 0,21704 |
| ENSG0000 | 0,002415 | 0,087895 | 3,069439 | 0,002247 | 0,21704 |
| ENSG0000 | 0,002924 | -0,240881 | 3,068707 | 0,002253 | 0,21704 |
| ENSG0000 | 0,006698 | -0,103981 | 3,066636 | 0,002268 | 0,21704 |
| ENSG0000 | 0,002588 | -0,334873 | 3,056363 | 0,002346 | 0,219796 |
| ENSG0000 | 0,001859 | -0,92927 | 3,053361 | 0,002369 | 0,219796 |
| ENSG0000 | -0,007906 | -0,894807 | -3,052351 | 0,002377 | 0,219796 |
| ENSG0000 | 0,004739 | -0,674144 | 3,042621 | 0,002454 | 0,222081 |
| ENSG0000 | 0,001792 | 0,418668 | 3,040948 | 0,002467 | 0,222081 |
| ENSG0000 | 0,002905 | 0,204243 | 3,035954 | 0,002508 | 0,222081 |
| ENSG0000 | -0,003287 | -1,081959 | -3,031942 | 0,002541 | 0,222081 |
| ENSG0000 | 0,002207 | -0,27203 | 3,031426 | 0,002545 | 0,222081 |
| ENSG0000 | -0,005912 | -0,859012 | -3,026178 | 0,002589 | 0,222081 |
| ENSG0000 | 0,002345 | 0,206523 | 3,022586 | 0,002619 | 0,222081 |

| | | | | | |
|---|---|---|---|---|---|
| ENSG0000 | 0,004624 | -0,546685 | 3,019034 | 0,00265 | 0,222081 |
| ENSG0000 | -0,00857 | 1,1582 | -3,016767 | 0,002669 | 0,222081 |
| ENSG0000 | 0,001694 | 0,726632 | 3,016531 | 0,002671 | 0,222081 |
| ENSG0000 | -0,008328 | 0,1387 | -2,998133 | 0,002835 | 0,233354 |
| ENSG0000 | -0,002945 | -1,271563 | -2,99273 | 0,002885 | 0,233405 |
| ENSG0000 | -0,002989 | -0,397295 | -2,991928 | 0,002893 | 0,233405 |
| ENSG0000 | -0,004193 | -0,373996 | -2,98412 | 0,002966 | 0,237025 |
| ENSG0000 | 0,003817 | -0,507903 | 2,978167 | 0,003024 | 0,239281 |
| ENSG0000 | 0,010567 | -1,143267 | 2,972094 | 0,003083 | 0,240969 |
| ENSG0000 | -0,003913 | 0,622606 | -2,970039 | 0,003104 | 0,240969 |
| ENSG0000 | -0,005589 | -0,374939 | -2,959921 | 0,003206 | 0,246573 |
| ENSG0000 | -0,00584 | 2,88052 | -2,948764 | 0,003322 | 0,252544 |
| ENSG0000 | 0,003289 | 0,155052 | 2,946615 | 0,003345 | 0,252544 |
| ENSG0000 | -0,004339 | -0,242747 | -2,943303 | 0,00338 | 0,2529 |
| ENSG0000 | -0,00511 | -0,016957 | -2,930979 | 0,003515 | 0,257748 |
| ENSG0000 | -0,008493 | -1,485549 | -2,929289 | 0,003534 | 0,257748 |
| ENSG0000 | 0,001598 | -0,193565 | 2,928846 | 0,003539 | 0,257748 |
| ENSG0000 | -0,003362 | -0,19897 | -2,923539 | 0,003599 | 0,258139 |
| ENSG0000 | -0,002815 | -0,352912 | -2,922826 | 0,003607 | 0,258139 |
| ENSG0000 | -0,003703 | -0,024656 | -2,904718 | 0,003819 | 0,261581 |
| ENSG0000 | -0,010131 | -3,290338 | -2,902784 | 0,003842 | 0,261581 |
| ENSG0000 | -0,003175 | -0,534824 | -2,900912 | 0,003865 | 0,261581 |
| ENSG0000 | 0,001834 | 0,122823 | 2,895858 | 0,003927 | 0,261581 |
| ENSG0000 | -0,00426 | 0,726413 | -2,894366 | 0,003945 | 0,261581 |
| ENSG0000 | -0,008796 | 0,494253 | -2,890533 | 0,003993 | 0,261581 |
| ENSG0000 | -0,001327 | -0,033016 | -2,886251 | 0,004047 | 0,261581 |
| ENSG0000 | 0,007573 | 0,331966 | 2,885147 | 0,004061 | 0,261581 |
| ENSG0000 | 0,002792 | -0,30218 | 2,885135 | 0,004061 | 0,261581 |
| ENSG0000 | 0,003625 | -0,028319 | 2,882428 | 0,004096 | 0,261581 |
| ENSG0000 | -0,003089 | 0,010898 | -2,882019 | 0,004101 | 0,261581 |
| ENSG0000 | 0,004203 | 0,397342 | 2,8802 | 0,004124 | 0,261581 |
| ENSG0000 | 0,005734 | -0,175443 | 2,877157 | 0,004164 | 0,261581 |
| ENSG0000 | 0,003123 | -0,247969 | 2,872971 | 0,004219 | 0,261581 |
| ENSG0000 | 0,004078 | -0,274157 | 2,870006 | 0,004258 | 0,261581 |
| ENSG0000 | 0,007063 | -0,046065 | 2,868754 | 0,004274 | 0,261581 |
| ENSG0000 | -0,008566 | 0,539095 | -2,867771 | 0,004287 | 0,261581 |
| ENSG0000 | -0,004996 | -0,250896 | -2,867662 | 0,004289 | 0,261581 |
| ENSG0000 | 0,003144 | -1,162638 | 2,864976 | 0,004325 | 0,261581 |
| ENSG0000 | -0,002216 | -0,025151 | -2,863937 | 0,004339 | 0,261581 |
| ENSG0000 | 0,002333 | -0,789481 | 2,862966 | 0,004352 | 0,261581 |
| ENSG0000 | 0,003905 | -0,114089 | 2,862795 | 0,004354 | 0,261581 |
| ENSG0000 | -0,013636 | 1,057846 | -2,853874 | 0,004477 | 0,26391 |
| ENSG0000 | 0,002714 | -0,159763 | 2,850917 | 0,004518 | 0,26391 |
| ENSG0000 | -0,012871 | 1,366264 | -2,849551 | 0,004537 | 0,26391 |
| ENSG0000 | 0,006787 | -0,820561 | 2,845921 | 0,004588 | 0,26391 |
| ENSG0000 | 0,004976 | 0,413722 | 2,844516 | 0,004608 | 0,26391 |
| ENSG0000 | 0,003451 | 0,312778 | 2,843332 | 0,004625 | 0,26391 |
| ENSG0000 | -0,005763 | -0,450836 | -2,842152 | 0,004642 | 0,26391 |

| | | | | | |
|---|---|---|---|---|---|
| ENSG0000 | -0,00428 | 0,263174 | -2,841634 | 0,00465 | 0,26391 |
| ENSG0000 | 0,001812 | -0,428063 | 2,839199 | 0,004685 | 0,264083 |
| ENSG0000 | 0,010088 | 0,202672 | 2,834923 | 0,004747 | 0,264172 |
| ENSG0000 | -0,004184 | 0,518064 | -2,834683 | 0,004751 | 0,264172 |
| ENSG0000 | -0,003448 | -0,327325 | -2,829789 | 0,004823 | 0,266102 |
| ENSG0000 | -0,000655 | 0,161339 | -2,824149 | 0,004907 | 0,266102 |
| ENSG0000 | -0,002361 | 0,280517 | -2,823683 | 0,004914 | 0,266102 |
| ENSG0000 | 0,005509 | -0,0028 | 2,822926 | 0,004926 | 0,266102 |
| ENSG0000 | 0,007847 | 0,529351 | 2,81975 | 0,004974 | 0,266102 |
| ENSG0000 | 0,005401 | 0,469988 | 2,816018 | 0,005031 | 0,266102 |
| ENSG0000 | -0,004269 | 0,412688 | -2,812576 | 0,005085 | 0,266102 |
| ENSG0000 | -0,002131 | 0,033209 | -2,812275 | 0,00509 | 0,266102 |
| ENSG0000 | 0,003867 | 1,946199 | 2,811195 | 0,005106 | 0,266102 |
| ENSG0000 | -0,00957 | 0,458844 | -2,81092 | 0,005111 | 0,266102 |
| ENSG0000 | 0,004968 | 0,184888 | 2,805826 | 0,005191 | 0,266102 |
| ENSG0000 | -0,004839 | -0,80146 | -2,805148 | 0,005202 | 0,266102 |
| ENSG0000 | 0,008872 | 0,728822 | 2,80424 | 0,005216 | 0,266102 |
| ENSG0000 | 0,008403 | 1,400465 | 2,802886 | 0,005238 | 0,266102 |
| ENSG0000 | 0,004831 | -0,838055 | 2,797327 | 0,005328 | 0,268442 |
| ENSG0000 | -0,005717 | -1,342855 | -2,796002 | 0,005349 | 0,268442 |
| ENSG0000 | 0,009502 | 1,046169 | 2,783378 | 0,005559 | 0,277274 |
| ENSG0000 | 0,008342 | 0,503339 | 2,781079 | 0,005598 | 0,277523 |
| ENSG0000 | -0,000741 | 0,127798 | -2,778219 | 0,005647 | 0,277523 |
| ENSG0000 | 0,003604 | -0,252919 | 2,777151 | 0,005665 | 0,277523 |
| ENSG0000 | 0,006534 | 0,884605 | 2,77308 | 0,005735 | 0,279308 |
| ENSG0000 | 0,002576 | -0,086739 | 2,770665 | 0,005778 | 0,279623 |
| ENSG0000 | -0,001957 | -0,258427 | -2,76827 | 0,00582 | 0,279623 |
| ENSG0000 | 0,003659 | -1,431576 | 2,766517 | 0,005851 | 0,279623 |
| ENSG0000 | 0,001944 | -0,346342 | 2,761007 | 0,005949 | 0,279623 |
| ENSG0000 | -0,004705 | -0,457902 | -2,760676 | 0,005955 | 0,279623 |
| ENSG0000 | 0,004192 | 1,073323 | 2,75988 | 0,005969 | 0,279623 |
| ENSG0000 | 0,010931 | 0,604369 | 2,758113 | 0,006001 | 0,279623 |
| ENSG0000 | 0,007506 | -0,483109 | 2,754855 | 0,00606 | 0,279623 |
| ENSG0000 | 0,001621 | -0,124567 | 2,753169 | 0,006091 | 0,279623 |
| ENSG0000 | 0,001323 | 0,352904 | 2,752344 | 0,006106 | 0,279623 |
| ENSG0000 | 0,001871 | 0,57526 | 2,75184 | 0,006116 | 0,279623 |
| ENSG0000 | 0,005335 | 0,666307 | 2,738721 | 0,006362 | 0,288551 |
| ENSG0000 | 0,004036 | -0,373657 | 2,73771 | 0,006381 | 0,288551 |
| ENSG0000 | -0,003427 | 0,197309 | -2,731469 | 0,006502 | 0,292393 |
| ENSG0000 | -0,003396 | 1,213297 | -2,725666 | 0,006615 | 0,295467 |
| ENSG0000 | -0,003181 | 1,154286 | -2,72434 | 0,006642 | 0,295467 |
| ENSG0000 | 0,007784 | -0,72642 | 2,720483 | 0,006719 | 0,297283 |
| ENSG0000 | -0,005717 | -0,313794 | -2,718644 | 0,006756 | 0,29732 |
| ENSG0000 | 0,003812 | -0,572774 | 2,714451 | 0,006841 | 0,299459 |
| ENSG0000 | -0,005329 | 0,037376 | -2,712149 | 0,006888 | 0,299924 |
| ENSG0000 | 0,006238 | -0,757109 | 2,709275 | 0,006947 | 0,300908 |
| ENSG0000 | -0,003692 | 0,115659 | -2,705688 | 0,007021 | 0,302542 |
| ENSG0000 | -0,003008 | -0,666637 | -2,700831 | 0,007123 | 0,305339 |

| | | | | | |
|---|---|---|---|---|---|
| ENSG0000 | 0,003209 | -0,221796 | 2,69504 | 0,007247 | 0,309018 |
| ENSG0000 | -0,003311 | 0,295505 | -2,690636 | 0,007342 | 0,309476 |
| ENSG0000 | -0,008256 | 1,348579 | -2,687029 | 0,007421 | 0,309476 |
| ENSG0000 | 0,002816 | 0,206533 | 2,68575 | 0,007449 | 0,309476 |
| ENSG0000 | 0,000876 | 0,126948 | 2,684855 | 0,007468 | 0,309476 |
| ENSG0000 | 0,006911 | -0,056474 | 2,684711 | 0,007472 | 0,309476 |
| ENSG0000 | -0,009986 | 0,548245 | -2,684189 | 0,007483 | 0,309476 |
| ENSG0000 | -0,005838 | -0,782549 | -2,682003 | 0,007532 | 0,309791 |
| ENSG0000 | 0,007119 | 0,501064 | 2,680456 | 0,007566 | 0,309791 |
| ENSG0000 | 0,009764 | 0,628941 | 2,675681 | 0,007673 | 0,312262 |
| ENSG0000 | 0,004362 | -0,748139 | 2,673813 | 0,007716 | 0,312262 |
| ENSG0000 | -0,006068 | -0,619539 | -2,67199 | 0,007757 | 0,312262 |
| ENSG0000 | -0,006217 | 0,312587 | -2,67107 | 0,007778 | 0,312262 |
| ENSG0000 | -0,009241 | 1,063578 | -2,664848 | 0,007922 | 0,315859 |
| ENSG0000 | -0,004368 | 0,542717 | -2,663867 | 0,007944 | 0,315859 |
| ENSG0000 | -0,003605 | -0,614908 | -2,661602 | 0,007997 | 0,316436 |
| ENSG0000 | 0,003808 | -0,559963 | 2,658343 | 0,008074 | 0,316539 |
| ENSG0000 | -0,003068 | 0,403019 | -2,657631 | 0,008091 | 0,316539 |
| ENSG0000 | -0,001365 | -0,142578 | -2,656605 | 0,008115 | 0,316539 |
| ENSG0000 | -0,005792 | 0,875752 | -2,652109 | 0,008223 | 0,319219 |
| ENSG0000 | 0,00266 | -0,488538 | 2,649437 | 0,008287 | 0,320166 |
| ENSG0000 | -0,003663 | 0,037009 | -2,645069 | 0,008394 | 0,320166 |
| ENSG0000 | 0,00211 | 0,56171 | 2,644398 | 0,00841 | 0,320166 |
| ENSG0000 | -0,002299 | 0,685222 | -2,643381 | 0,008435 | 0,320166 |
| ENSG0000 | -0,005115 | 0,434881 | -2,643115 | 0,008442 | 0,320166 |
| ENSG0000 | 0,002791 | 0,361719 | 2,641374 | 0,008485 | 0,32022 |
| ENSG0000 | -0,002474 | -0,176718 | -2,638562 | 0,008555 | 0,32022 |
| ENSG0000 | -0,00337 | -0,556742 | -2,638346 | 0,00856 | 0,32022 |
| ENSG0000 | -0,007464 | 0,847969 | -2,633517 | 0,008681 | 0,323283 |
| ENSG0000 | -0,003174 | 0,318987 | -2,631071 | 0,008743 | 0,324123 |
| ENSG0000 | 0,009424 | -2,638969 | 2,62569 | 0,008881 | 0,327752 |
| ENSG0000 | -0,011426 | -0,697622 | -2,622027 | 0,008976 | 0,329773 |
| ENSG0000 | 0,00436 | 0,531841 | 2,620488 | 0,009016 | 0,329775 |
| ENSG0000 | -0,002171 | -0,27836 | -2,615503 | 0,009147 | 0,333088 |
| ENSG0000 | -0,003611 | -1,142366 | -2,613096 | 0,009211 | 0,333937 |
| ENSG0000 | 0,003925 | -0,528598 | 2,611074 | 0,009265 | 0,33442 |
| ENSG0000 | -0,005557 | 1,203337 | -2,609428 | 0,009309 | 0,334547 |
| ENSG0000 | 0,006603 | -0,294748 | 2,605475 | 0,009416 | 0,336913 |
| ENSG0000 | 0,006198 | 0,510407 | 2,602984 | 0,009483 | 0,337872 |
| ENSG0000 | -0,003592 | -0,17186 | -2,596876 | 0,009652 | 0,338241 |
| ENSG0000 | -0,004674 | -0,143605 | -2,595074 | 0,009702 | 0,338241 |
| ENSG0000 | 0,002672 | 0,049533 | 2,593021 | 0,009759 | 0,338241 |
| ENSG0000 | 0,006337 | -0,598564 | 2,59281 | 0,009765 | 0,338241 |
| ENSG0000 | 0,002826 | 0,220841 | 2,592417 | 0,009776 | 0,338241 |
| ENSG0000 | 0,014214 | 1,400814 | 2,59237 | 0,009777 | 0,338241 |
| ENSG0000 | 0,004795 | -0,101778 | 2,590746 | 0,009823 | 0,338241 |
| ENSG0000 | 0,001417 | -0,140864 | 2,588528 | 0,009886 | 0,338241 |
| ENSG0000 | -0,001718 | -0,767557 | -2,58809 | 0,009898 | 0,338241 |

| | | | | | |
|---|---|---|---|---|---|
| ENSG0000 | 0,003357 | -0,238824 | 2,587864 | 0,009905 | 0,338241 |
| ENSG0000 | -0,006126 | 1,292249 | -2,581313 | 0,010092 | 0,343226 |
| ENSG0000 | -0,003329 | 0,301454 | -2,577866 | 0,010192 | 0,344444 |
| ENSG0000 | -0,004455 | 0,896439 | -2,577198 | 0,010212 | 0,344444 |
| ENSG0000 | 0,003469 | -0,060623 | 2,574206 | 0,0103 | 0,345983 |
| ENSG0000 | -0,001636 | 0,237462 | -2,566848 | 0,010518 | 0,351395 |
| ENSG0000 | 0,003832 | 0,910168 | 2,565915 | 0,010546 | 0,351395 |
| ENSG0000 | -0,001102 | -0,1349 | -2,562141 | 0,01066 | 0,352471 |
| ENSG0000 | -0,002572 | 0,083694 | -2,561121 | 0,010691 | 0,352471 |
| ENSG0000 | 0,002256 | -0,118821 | 2,5606 | 0,010707 | 0,352471 |
| ENSG0000 | -0,008675 | -0,676282 | -2,558227 | 0,010779 | 0,352523 |
| ENSG0000 | -0,006633 | 0,538415 | -2,557746 | 0,010794 | 0,352523 |
| ENSG0000 | 0,008326 | 1,884331 | 2,554269 | 0,010901 | 0,354107 |
| ENSG0000 | 0,008869 | 0,722244 | 2,553384 | 0,010929 | 0,354107 |
| ENSG0000 | -0,004096 | 0,06193 | -2,550982 | 0,011003 | 0,35416 |
| ENSG0000 | -0,002656 | 0,039323 | -2,547954 | 0,011098 | 0,35416 |
| ENSG0000 | -0,005178 | -0,82762 | -2,547915 | 0,0111 | 0,35416 |
| ENSG0000 | 0,002113 | -0,10288 | 2,547821 | 0,011102 | 0,35416 |
| ENSG0000 | -0,006455 | 0,182546 | -2,543542 | 0,011238 | 0,355937 |
| ENSG0000 | -0,004561 | -0,421621 | -2,542678 | 0,011265 | 0,355937 |
| ENSG0000 | -0,003127 | 0,45615 | -2,540683 | 0,011329 | 0,355937 |
| ENSG0000 | 0,002221 | 0,238738 | 2,540528 | 0,011334 | 0,355937 |
| ENSG0000 | -0,005852 | 0,136579 | -2,538746 | 0,011391 | 0,355937 |
| ENSG0000 | -0,002726 | -0,461271 | -2,537925 | 0,011418 | 0,355937 |
| ENSG0000 | 0,006559 | -0,145859 | 2,535587 | 0,011493 | 0,356014 |
| ENSG0000 | 0,002337 | -0,347259 | 2,535175 | 0,011507 | 0,356014 |
| ENSG0000 | 0,001556 | 0,235715 | 2,527565 | 0,011756 | 0,360356 |
| ENSG0000 | -0,005044 | 0,705767 | -2,526667 | 0,011786 | 0,360356 |
| ENSG0000 | -0,002379 | 0,597788 | -2,525547 | 0,011823 | 0,360356 |
| ENSG0000 | -0,003686 | 0,870206 | -2,524687 | 0,011852 | 0,360356 |
| ENSG0000 | -0,002443 | 0,320981 | -2,524263 | 0,011866 | 0,360356 |
| ENSG0000 | -0,004719 | 0,527475 | -2,519423 | 0,012028 | 0,363947 |
| ENSG0000 | -0,004712 | -0,031959 | -2,517444 | 0,012095 | 0,364635 |
| ENSG0000 | -0,002081 | 0,331725 | -2,508797 | 0,012392 | 0,372216 |
| ENSG0000 | 0,002342 | 0,468771 | 2,504001 | 0,012559 | 0,373876 |
| ENSG0000 | 0,002774 | -0,092191 | 2,50174 | 0,012639 | 0,373876 |
| ENSG0000 | 0,002747 | 0,198284 | 2,501703 | 0,01264 | 0,373876 |
| ENSG0000 | -0,003484 | -0,019056 | -2,501332 | 0,012653 | 0,373876 |
| ENSG0000 | -0,004009 | -0,141576 | -2,500736 | 0,012675 | 0,373876 |
| ENSG0000 | -0,004983 | -0,566976 | -2,496646 | 0,01282 | 0,375893 |
| ENSG0000 | -0,004952 | 0,711652 | -2,496247 | 0,012834 | 0,375893 |
| ENSG0000 | -0,006226 | -0,7169 | -2,490967 | 0,013024 | 0,38011 |
| ENSG0000 | -0,002168 | -0,017376 | -2,484532 | 0,01326 | 0,385367 |
| ENSG0000 | 0,00418 | 0,504482 | 2,481531 | 0,013371 | 0,385367 |
| ENSG0000 | 0,008033 | -0,289048 | 2,4806 | 0,013405 | 0,385367 |
| ENSG0000 | 0,006476 | 0,937589 | 2,477113 | 0,013535 | 0,385367 |
| ENSG0000 | -0,004941 | -0,883876 | -2,477061 | 0,013537 | 0,385367 |
| ENSG0000 | -0,002202 | -0,307106 | -2,477061 | 0,013537 | 0,385367 |

| | | | | | |
|---|---|---|---|---|---|
| ENSG0000 | 0,001517 | -0,006382 | 2,47659 | 0,013555 | 0,385367 |
| ENSG0000 | -0,004315 | -0,622391 | -2,473544 | 0,01367 | 0,385367 |
| ENSG0000 | -0,00082 | -0,087552 | -2,473141 | 0,013685 | 0,385367 |
| ENSG0000 | 0,00373 | 0,242365 | 2,47253 | 0,013708 | 0,385367 |
| ENSG0000 | 0,002385 | 1,014447 | 2,472229 | 0,01372 | 0,385367 |
| ENSG0000 | -0,003162 | -0,21798 | -2,468124 | 0,013876 | 0,387411 |
| ENSG0000 | 0,007209 | 0,680778 | 2,467856 | 0,013887 | 0,387411 |
| ENSG0000 | 0,003009 | -0,399065 | 2,465532 | 0,013976 | 0,388587 |
| ENSG0000 | -0,006395 | 0,243865 | -2,463741 | 0,014045 | 0,389198 |
| ENSG0000 | -0,001932 | 0,092111 | -2,461911 | 0,014116 | 0,389856 |
| ENSG0000 | 0,001032 | -0,11755 | 2,455163 | 0,014381 | 0,395843 |
| ENSG0000 | 0,000536 | -0,081555 | 2,452184 | 0,014499 | 0,397769 |
| ENSG0000 | 0,003458 | 1,906077 | 2,450241 | 0,014577 | 0,398137 |
| ENSG0000 | -0,003457 | 0,264481 | -2,448693 | 0,014639 | 0,398137 |
| ENSG0000 | -0,002871 | -0,485025 | -2,447979 | 0,014668 | 0,398137 |
| ENSG0000 | 0,00381 | -0,917507 | 2,446171 | 0,014741 | 0,398137 |
| ENSG0000 | -0,005678 | 1,184943 | -2,445829 | 0,014755 | 0,398137 |
| ENSG0000 | -0,002758 | 0,806017 | -2,441333 | 0,014938 | 0,400308 |
| ENSG0000 | -0,005304 | 0,828718 | -2,440465 | 0,014973 | 0,400308 |
| ENSG0000 | 0,010946 | -1,336154 | 2,438196 | 0,015067 | 0,400308 |
| ENSG0000 | -0,002695 | -0,199786 | -2,437306 | 0,015103 | 0,400308 |
| ENSG0000 | -0,002885 | -0,713533 | -2,436836 | 0,015123 | 0,400308 |
| ENSG0000 | 0,008842 | 1,120561 | 2,436091 | 0,015154 | 0,400308 |
| ENSG0000 | 0,008131 | -0,014193 | 2,434844 | 0,015205 | 0,400308 |
| ENSG0000 | 0,004124 | -1,516915 | 2,43439 | 0,015224 | 0,400308 |
| ENSG0000 | -0,005477 | 0,612503 | -2,431083 | 0,015363 | 0,402353 |
| ENSG0000 | -0,004487 | -0,085094 | -2,430194 | 0,0154 | 0,402353 |
| ENSG0000 | 0,006294 | 0,650731 | 2,428748 | 0,015461 | 0,402514 |
| ENSG0000 | -0,002222 | -0,589252 | -2,427728 | 0,015504 | 0,402514 |
| ENSG0000 | 0,009338 | -1,058269 | 2,423856 | 0,015668 | 0,405048 |
| ENSG0000 | -0,00491 | -0,225885 | -2,423118 | 0,0157 | 0,405048 |
| ENSG0000 | -0,003313 | -0,238852 | -2,417647 | 0,015935 | 0,409498 |
| ENSG0000 | 0,003415 | 0,999651 | 2,415743 | 0,016018 | 0,409498 |
| ENSG0000 | 0,004309 | 0,817477 | 2,414168 | 0,016087 | 0,409498 |
| ENSG0000 | -0,004581 | -0,971974 | -2,412407 | 0,016164 | 0,409498 |
| ENSG0000 | -0,004206 | 0,191283 | -2,411912 | 0,016186 | 0,409498 |
| ENSG0000 | 0,003064 | 0,423656 | 2,411163 | 0,016219 | 0,409498 |
| ENSG0000 | -0,002359 | 0,307377 | -2,411115 | 0,016221 | 0,409498 |
| ENSG0000 | 0,002002 | 0,122371 | 2,408448 | 0,016338 | 0,41054 |
| ENSG0000 | -0,002411 | 0,039495 | -2,406949 | 0,016405 | 0,41054 |
| ENSG0000 | 0,00545 | -0,630297 | 2,405994 | 0,016447 | 0,41054 |
| ENSG0000 | 0,00122 | 0,097566 | 2,405679 | 0,016461 | 0,41054 |
| ENSG0000 | 0,004839 | 1,033241 | 2,389289 | 0,017207 | 0,427829 |
| ENSG0000 | -0,002071 | -0,183541 | -2,386046 | 0,017358 | 0,43014 |
| ENSG0000 | 0,002411 | -0,438118 | 2,384989 | 0,017407 | 0,43014 |
| ENSG0000 | 0,001812 | -0,063614 | 2,382727 | 0,017513 | 0,43014 |
| ENSG0000 | 0,008459 | -1,99588 | 2,382617 | 0,017519 | 0,43014 |
| ENSG0000 | -0,002849 | -0,062181 | -2,381642 | 0,017564 | 0,43014 |

| | | | | | |
|---|---|---|---|---|---|
| ENSG0000 | -0,004218 | 0,800224 | -2,38061 | 0,017613 | 0,43014 |
| ENSG0000 | -0,001234 | 0,549326 | -2,37658 | 0,017805 | 0,430533 |
| ENSG0000 | 0,005086 | -2,475461 | 2,376171 | 0,017824 | 0,430533 |
| ENSG0000 | 0,0033 | 0,559058 | 2,374677 | 0,017896 | 0,430533 |
| ENSG0000 | -0,002829 | 0,573321 | -2,373539 | 0,017951 | 0,430533 |
| ENSG0000 | -0,002016 | 0,266156 | -2,369886 | 0,018127 | 0,430533 |
| ENSG0000 | 0,006765 | -2,089829 | 2,368862 | 0,018177 | 0,430533 |
| ENSG0000 | -0,001739 | -0,049996 | -2,368826 | 0,018179 | 0,430533 |
| ENSG0000 | 0,007665 | 0,046273 | 2,368631 | 0,018188 | 0,430533 |
| ENSG0000 | 0,003272 | 0,556181 | 2,367389 | 0,018249 | 0,430533 |
| ENSG0000 | -0,002071 | -0,27664 | -2,367159 | 0,01826 | 0,430533 |
| ENSG0000 | 0,00324 | -0,318501 | 2,367055 | 0,018265 | 0,430533 |
| ENSG0000 | 0,005523 | -0,459803 | 2,366309 | 0,018302 | 0,430533 |
| ENSG0000 | -0,003024 | -0,640664 | -2,364966 | 0,018367 | 0,430533 |
| ENSG0000 | -0,004667 | 1,13176 | -2,364519 | 0,018389 | 0,430533 |
| ENSG0000 | 0,005904 | -0,00995 | 2,363261 | 0,018451 | 0,430533 |
| ENSG0000 | 0,001241 | -0,222272 | 2,362955 | 0,018466 | 0,430533 |
| ENSG0000 | 0,003597 | 0,573963 | 2,358892 | 0,018668 | 0,433464 |
| ENSG0000 | 0,002008 | -0,088609 | 2,357516 | 0,018736 | 0,433464 |
| ENSG0000 | 0,001234 | -0,218249 | 2,356462 | 0,018789 | 0,433464 |
| ENSG0000 | -0,00736 | 0,204904 | -2,356047 | 0,01881 | 0,433464 |
| ENSG0000 | -0,003185 | -0,385715 | -2,355139 | 0,018855 | 0,433464 |
| ENSG0000 | -0,006706 | -0,606125 | -2,353037 | 0,018961 | 0,434684 |
| ENSG0000 | -0,004463 | 1,182292 | -2,345492 | 0,019346 | 0,441411 |
| ENSG0000 | 0,00264 | 0,210186 | 2,343601 | 0,019443 | 0,441411 |
| ENSG0000 | 0,00419 | 0,865272 | 2,341521 | 0,019551 | 0,441411 |
| ENSG0000 | -0,003763 | -0,343744 | -2,340105 | 0,019624 | 0,441411 |
| ENSG0000 | -0,003043 | -0,342107 | -2,338785 | 0,019693 | 0,441411 |
| ENSG0000 | -0,005682 | 0,909664 | -2,337893 | 0,01974 | 0,441411 |
| ENSG0000 | 0,002019 | 0,043884 | 2,335417 | 0,019869 | 0,441411 |
| ENSG0000 | -0,005283 | 0,541234 | -2,33343 | 0,019974 | 0,441411 |
| ENSG0000 | -0,003733 | -0,457491 | -2,333111 | 0,019991 | 0,441411 |
| ENSG0000 | -0,003253 | 0,360044 | -2,333005 | 0,019997 | 0,441411 |
| ENSG0000 | -0,001247 | 0,190503 | -2,332407 | 0,020028 | 0,441411 |
| ENSG0000 | 0,002774 | 0,196527 | 2,330771 | 0,020115 | 0,441411 |
| ENSG0000 | 0,003618 | -0,30376 | 2,329994 | 0,020156 | 0,441411 |
| ENSG0000 | 0,008081 | 0,746216 | 2,329046 | 0,020207 | 0,441411 |
| ENSG0000 | -0,001796 | 0,073269 | -2,327799 | 0,020274 | 0,441411 |
| ENSG0000 | -0,002296 | 0,262671 | -2,327435 | 0,020293 | 0,441411 |
| ENSG0000 | 0,003538 | -0,315195 | 2,327395 | 0,020295 | 0,441411 |
| ENSG0000 | 0,00411 | 0,319183 | 2,327309 | 0,0203 | 0,441411 |
| ENSG0000 | -0,002764 | -0,085487 | -2,326603 | 0,020338 | 0,441411 |
| ENSG0000 | -0,007303 | 0,329043 | -2,326585 | 0,020339 | 0,441411 |
| ENSG0000 | -0,002735 | 0,064978 | -2,325796 | 0,020381 | 0,441411 |
| ENSG0000 | 0,007446 | -1,149896 | 2,323451 | 0,020508 | 0,442985 |
| ENSG0000 | 0,000784 | -0,118759 | 2,318364 | 0,020784 | 0,443103 |
| ENSG0000 | 0,003962 | -0,04471 | 2,318344 | 0,020785 | 0,443103 |
| ENSG0000 | 0,003217 | -1,650038 | 2,318261 | 0,02079 | 0,443103 |

| | | | | | |
|---|---|---|---|---|---|
| ENSG0000 | 0,00332 | -0,33943 | 2,318039 | 0,020802 | 0,443103 |
| ENSG0000 | -0,00251 | -0,434196 | -2,316475 | 0,020888 | 0,443103 |
| ENSG0000 | 0,002533 | -0,11671 | 2,315107 | 0,020963 | 0,443103 |
| ENSG0000 | -0,001633 | -0,206076 | -2,313612 | 0,021046 | 0,443103 |
| ENSG0000 | 0,004987 | -0,552996 | 2,313162 | 0,021071 | 0,443103 |
| ENSG0000 | -0,004284 | -0,519832 | -2,312755 | 0,021093 | 0,443103 |
| ENSG0000 | 0,002646 | -0,664022 | 2,312588 | 0,021102 | 0,443103 |
| ENSG0000 | -0,002763 | 0,95376 | -2,311577 | 0,021158 | 0,443103 |
| ENSG0000 | 0,002511 | -0,016288 | 2,311565 | 0,021159 | 0,443103 |
| ENSG0000 | 0,003713 | -0,625999 | 2,307845 | 0,021367 | 0,445364 |
| ENSG0000 | -0,002421 | -0,569611 | -2,306938 | 0,021418 | 0,445364 |
| ENSG0000 | 0,004147 | -0,433608 | 2,306324 | 0,021452 | 0,445364 |
| ENSG0000 | -0,002066 | -0,137525 | -2,301528 | 0,021723 | 0,445364 |
| ENSG0000 | 0,001987 | -0,141226 | 2,301392 | 0,021731 | 0,445364 |
| ENSG0000 | -0,007016 | 0,512005 | -2,300801 | 0,021765 | 0,445364 |
| ENSG0000 | -0,00279 | -0,258273 | -2,299821 | 0,021821 | 0,445364 |
| ENSG0000 | -0,006401 | -0,55457 | -2,299422 | 0,021843 | 0,445364 |
| ENSG0000 | 0,004458 | -0,383617 | 2,298789 | 0,02188 | 0,445364 |
| ENSG0000 | 0,006004 | -2,961741 | 2,298363 | 0,021904 | 0,445364 |
| ENSG0000 | -0,0066 | 1,133759 | -2,296105 | 0,022034 | 0,445364 |
| ENSG0000 | -0,002023 | 0,802868 | -2,295774 | 0,022053 | 0,445364 |
| ENSG0000 | 0,002903 | 0,45506 | 2,295292 | 0,022081 | 0,445364 |
| ENSG0000 | 0,00851 | 0,389971 | 2,293841 | 0,022164 | 0,445364 |
| ENSG0000 | 0,007 | 0,895707 | 2,292699 | 0,022231 | 0,445364 |
| ENSG0000 | 0,006591 | -0,782412 | 2,292565 | 0,022238 | 0,445364 |
| ENSG0000 | -0,005071 | -0,762097 | -2,290664 | 0,022349 | 0,445364 |
| ENSG0000 | -0,00235 | 0,292152 | -2,290512 | 0,022358 | 0,445364 |
| ENSG0000 | 0,008258 | -1,196642 | 2,290155 | 0,022379 | 0,445364 |
| ENSG0000 | -0,003447 | -0,903103 | -2,287966 | 0,022507 | 0,445364 |
| ENSG0000 | -0,003792 | 0,413287 | -2,287168 | 0,022553 | 0,445364 |
| ENSG0000 | -0,007521 | 2,244475 | -2,286654 | 0,022584 | 0,445364 |
| ENSG0000 | -0,002571 | -0,113925 | -2,286309 | 0,022604 | 0,445364 |
| ENSG0000 | 0,00577 | 0,005887 | 2,285516 | 0,022651 | 0,445364 |
| ENSG0000 | 0,002312 | 0,611183 | 2,283098 | 0,022794 | 0,445364 |
| ENSG0000 | 0,001296 | -0,293707 | 2,282314 | 0,02284 | 0,445364 |
| ENSG0000 | -0,004468 | 0,473686 | -2,282215 | 0,022846 | 0,445364 |
| ENSG0000 | -0,003601 | -0,452741 | -2,279555 | 0,023005 | 0,445364 |
| ENSG0000 | 0,006067 | -1,056707 | 2,279289 | 0,023021 | 0,445364 |
| ENSG0000 | -0,003386 | -0,514614 | -2,278609 | 0,023061 | 0,445364 |
| ENSG0000 | 0,001122 | -0,069553 | 2,278143 | 0,023089 | 0,445364 |
| ENSG0000 | 0,002214 | -0,5054 | 2,277779 | 0,023111 | 0,445364 |
| ENSG0000 | -0,00286 | -0,028423 | -2,277713 | 0,023115 | 0,445364 |
| ENSG0000 | 0,004431 | 0,405895 | 2,277491 | 0,023128 | 0,445364 |
| ENSG0000 | 0,002095 | -0,396783 | 2,276716 | 0,023175 | 0,445364 |
| ENSG0000 | -0,005642 | -0,68008 | -2,274626 | 0,023301 | 0,445364 |
| ENSG0000 | -0,003172 | 0,902919 | -2,2744 | 0,023315 | 0,445364 |
| ENSG0000 | -0,003266 | -0,212805 | -2,27376 | 0,023353 | 0,445364 |
| ENSG0000 | -0,003708 | 0,575163 | -2,272981 | 0,023401 | 0,445364 |

| | | | | | |
|---|---|---|---|---|---|
| ENSG0000 | 0,004551 | 0,891874 | 2,271839 | 0,02347 | 0,445364 |
| ENSG0000 | 0,002646 | 0,537753 | 2,271166 | 0,023511 | 0,445364 |
| ENSG0000 | -0,001405 | -0,118958 | -2,269639 | 0,023604 | 0,445364 |
| ENSG0000 | -0,010225 | -0,042049 | -2,269282 | 0,023626 | 0,445364 |
| ENSG0000 | -0,006205 | 0,052135 | -2,267666 | 0,023725 | 0,445364 |
| ENSG0000 | 0,002115 | -0,182649 | 2,267215 | 0,023753 | 0,445364 |
| ENSG0000 | -0,008624 | 0,627017 | -2,267154 | 0,023756 | 0,445364 |
| ENSG0000 | 0,006657 | 0,403801 | 2,265431 | 0,023863 | 0,446338 |
| ENSG0000 | 0,006243 | -1,171944 | 2,261511 | 0,024105 | 0,446666 |
| ENSG0000 | -0,004052 | -0,187815 | -2,261203 | 0,024125 | 0,446666 |
| ENSG0000 | -0,003049 | 0,178897 | -2,260489 | 0,024169 | 0,446666 |
| ENSG0000 | -0,009605 | 1,657661 | -2,260421 | 0,024173 | 0,446666 |
| ENSG0000 | 0,001514 | -0,327018 | 2,260383 | 0,024176 | 0,446666 |
| ENSG0000 | 0,00322 | -0,569486 | 2,259904 | 0,024206 | 0,446666 |
| ENSG0000 | 0,005859 | -0,955308 | 2,258536 | 0,024291 | 0,447165 |
| ENSG0000 | -0,004685 | 0,299623 | -2,257737 | 0,024341 | 0,447165 |
| ENSG0000 | -0,007178 | -0,321482 | -2,255829 | 0,024462 | 0,44837 |
| ENSG0000 | 0,001418 | -0,223002 | 2,25372 | 0,024595 | 0,449216 |
| ENSG0000 | 0,003327 | -0,240318 | 2,253373 | 0,024617 | 0,449216 |
| ENSG0000 | -0,010939 | -0,17478 | -2,25007 | 0,024827 | 0,449432 |
| ENSG0000 | -0,003334 | -1,203226 | -2,250018 | 0,02483 | 0,449432 |
| ENSG0000 | -0,004047 | -0,75102 | -2,249793 | 0,024845 | 0,449432 |
| ENSG0000 | 0,003551 | 0,772098 | 2,249755 | 0,024847 | 0,449432 |
| ENSG0000 | -0,002984 | -0,422641 | -2,24881 | 0,024908 | 0,449538 |
| ENSG0000 | -0,002054 | -0,100876 | -2,247576 | 0,024987 | 0,44998 |
| ENSG0000 | 0,002144 | -0,262402 | 2,246539 | 0,025053 | 0,450196 |
| ENSG0000 | -0,004079 | -0,835578 | -2,244139 | 0,025208 | 0,451912 |
| ENSG0000 | 0,002235 | 0,328391 | 2,243361 | 0,025259 | 0,451912 |
| ENSG0000 | -0,005591 | 0,795065 | -2,236383 | 0,025715 | 0,456812 |
| ENSG0000 | 0,002977 | 0,641386 | 2,236183 | 0,025728 | 0,456812 |
| ENSG0000 | -0,002521 | -0,110974 | -2,235863 | 0,025749 | 0,456812 |
| ENSG0000 | 0,002442 | -0,550178 | 2,234525 | 0,025837 | 0,456812 |
| ENSG0000 | -0,003271 | 0,613722 | -2,233684 | 0,025893 | 0,456812 |
| ENSG0000 | -0,006903 | 0,104451 | -2,233571 | 0,0259 | 0,456812 |
| ENSG0000 | -0,004449 | -0,261197 | -2,233259 | 0,025921 | 0,456812 |
| ENSG0000 | 0,00269 | 0,445927 | 2,231178 | 0,026059 | 0,458188 |
| ENSG0000 | -0,005259 | -1,662925 | -2,230411 | 0,026111 | 0,458188 |
| ENSG0000 | -0,004983 | 0,92059 | -2,227291 | 0,026319 | 0,459916 |
| ENSG0000 | -0,003214 | 0,633247 | -2,225943 | 0,02641 | 0,459916 |
| ENSG0000 | -0,007229 | 0,045492 | -2,225315 | 0,026453 | 0,459916 |
| ENSG0000 | -0,005289 | 0,720858 | -2,225157 | 0,026463 | 0,459916 |
| ENSG0000 | -0,002401 | -0,118868 | -2,224783 | 0,026488 | 0,459916 |
| ENSG0000 | -0,001466 | -0,33786 | -2,22382 | 0,026554 | 0,460076 |
| ENSG0000 | -0,009465 | -4,258899 | -2,222415 | 0,026649 | 0,460373 |
| ENSG0000 | 0,003975 | -0,013456 | 2,221919 | 0,026683 | 0,460373 |
| ENSG0000 | -0,002914 | -0,702805 | -2,220953 | 0,026748 | 0,460541 |
| ENSG0000 | -0,001806 | 0,178531 | -2,217357 | 0,026994 | 0,461521 |
| ENSG0000 | -0,002737 | -0,297907 | -2,216925 | 0,027024 | 0,461521 |

| | | | | | |
|---|---|---|---|---|---|
| ENSG0000 | -0,001939 | 0,459578 | -2,216313 | 0,027066 | 0,461521 |
| ENSG0000 | 0,002166 | -0,310834 | 2,2162 | 0,027074 | 0,461521 |
| ENSG0000 | 0,004028 | -0,35939 | 2,21603 | 0,027086 | 0,461521 |
| ENSG0000 | -0,00237 | 0,43324 | -2,214552 | 0,027188 | 0,462262 |
| ENSG0000 | -0,006319 | -0,889277 | -2,212938 | 0,027299 | 0,462262 |
| ENSG0000 | -0,005197 | -1,702851 | -2,211533 | 0,027397 | 0,462262 |
| ENSG0000 | 0,001648 | 0,005719 | 2,211517 | 0,027398 | 0,462262 |
| ENSG0000 | -0,004285 | -0,081719 | -2,211275 | 0,027415 | 0,462262 |
| ENSG0000 | -0,004055 | 0,514852 | -2,210539 | 0,027466 | 0,462262 |
| ENSG0000 | 0,002779 | -0,231167 | 2,205514 | 0,027818 | 0,466994 |
| ENSG0000 | 0,002311 | -0,118048 | 2,204879 | 0,027863 | 0,466994 |
| ENSG0000 | -0,004289 | -1,417939 | -2,20411 | 0,027917 | 0,466994 |
| ENSG0000 | -0,002347 | -0,062638 | -2,202511 | 0,028031 | 0,467412 |
| ENSG0000 | -0,002404 | 0,130924 | -2,202155 | 0,028056 | 0,467412 |
| ENSG0000 | 0,003225 | 0,853703 | 2,198154 | 0,028341 | 0,469174 |
| ENSG0000 | 0,005539 | -0,413618 | 2,198127 | 0,028343 | 0,469174 |
| ENSG0000 | 0,004796 | -0,586953 | 2,197806 | 0,028366 | 0,469174 |
| ENSG0000 | 0,003133 | 1,819688 | 2,197478 | 0,02839 | 0,469174 |
| ENSG0000 | -0,007106 | -0,569767 | -2,196128 | 0,028487 | 0,469833 |
| ENSG0000 | 0,003964 | 0,426675 | 2,194275 | 0,02862 | 0,470553 |
| ENSG0000 | -0,004128 | -2,047135 | -2,193937 | 0,028645 | 0,470553 |
| ENSG0000 | 0,003602 | 0,069813 | 2,192884 | 0,028721 | 0,470866 |
| ENSG0000 | 0,00222 | -0,132653 | 2,1911 | 0,028851 | 0,471779 |
| ENSG0000 | 0,001302 | -0,364746 | 2,190199 | 0,028916 | 0,471779 |
| ENSG0000 | 0,002634 | 0,433922 | 2,189069 | 0,028999 | 0,471779 |
| ENSG0000 | 0,002813 | -0,7481 | 2,188968 | 0,029006 | 0,471779 |
| ENSG0000 | -0,001437 | 0,04342 | -2,186381 | 0,029196 | 0,473686 |
| ENSG0000 | -0,003269 | 1,04484 | -2,185493 | 0,029261 | 0,473686 |
| ENSG0000 | 0,00475 | 0,268202 | 2,184399 | 0,029342 | 0,473686 |
| ENSG0000 | 0,009893 | 0,32405 | 2,184237 | 0,029354 | 0,473686 |
| ENSG0000 | 0,003334 | 1,676398 | 2,183039 | 0,029442 | 0,474186 |
| ENSG0000 | -0,002818 | -0,410653 | -2,182187 | 0,029505 | 0,474275 |
| ENSG0000 | -0,007098 | -0,677061 | -2,180423 | 0,029636 | 0,474728 |
| ENSG0000 | 0,004603 | 0,548676 | 2,180256 | 0,029649 | 0,474728 |
| ENSG0000 | 0,001467 | -0,12821 | 2,179354 | 0,029716 | 0,474881 |
| ENSG0000 | -0,00578 | 0,067958 | -2,17855 | 0,029776 | 0,474918 |
| ENSG0000 | 0,003264 | -0,081881 | 2,176357 | 0,02994 | 0,476615 |
| ENSG0000 | 0,007199 | -0,533976 | 2,175078 | 0,030037 | 0,477223 |
| ENSG0000 | 0,007042 | 0,761958 | 2,17408 | 0,030112 | 0,477497 |
| ENSG0000 | -0,006538 | 1,373365 | -2,172042 | 0,030266 | 0,478178 |
| ENSG0000 | 0,001552 | 0,302089 | 2,170988 | 0,030346 | 0,478178 |
| ENSG0000 | 0,002904 | 0,048138 | 2,170243 | 0,030403 | 0,478178 |
| ENSG0000 | -0,007318 | -0,615579 | -2,168942 | 0,030502 | 0,478178 |
| ENSG0000 | -0,002093 | 0,244155 | -2,168622 | 0,030526 | 0,478178 |
| ENSG0000 | 0,005042 | 0,862271 | 2,168154 | 0,030562 | 0,478178 |
| ENSG0000 | -0,003939 | 0,079443 | -2,16747 | 0,030614 | 0,478178 |
| ENSG0000 | -0,003868 | 1,322034 | -2,167401 | 0,03062 | 0,478178 |
| ENSG0000 | 0,006238 | 1,091435 | 2,164772 | 0,030822 | 0,479075 |

| | | | | | |
|---|---|---|---|---|---|
| ENSG0000 | 0,00288 | 0,200758 | 2,164495 | 0,030843 | 0,479075 |
| ENSG0000 | 0,002224 | 0,047498 | 2,163741 | 0,030901 | 0,479075 |
| ENSG0000 | -0,004673 | -1,309042 | -2,163179 | 0,030944 | 0,479075 |
| ENSG0000 | 0,000899 | 0,659437 | 2,162872 | 0,030968 | 0,479075 |
| ENSG0000 | 0,001897 | -0,120833 | 2,158867 | 0,031279 | 0,482979 |
| ENSG0000 | 0,005465 | 0,552633 | 2,156796 | 0,031441 | 0,484569 |
| ENSG0000 | -0,00893 | 0,868453 | -2,154415 | 0,031628 | 0,486541 |
| ENSG0000 | -0,004232 | 0,12198 | -2,149979 | 0,031979 | 0,490014 |
| ENSG0000 | 0,002707 | -0,35265 | 2,149294 | 0,032033 | 0,490014 |
| ENSG0000 | -0,002494 | -0,199441 | -2,149293 | 0,032034 | 0,490014 |
| ENSG0000 | 0,002165 | 0,614418 | 2,147169 | 0,032203 | 0,490014 |
| ENSG0000 | 0,005223 | -0,295416 | 2,145148 | 0,032365 | 0,490014 |
| ENSG0000 | 0,002424 | -0,64103 | 2,144182 | 0,032443 | 0,490014 |
| ENSG0000 | -0,003714 | 1,631888 | -2,142925 | 0,032544 | 0,490014 |
| ENSG0000 | 0,001268 | -0,049006 | 2,142885 | 0,032547 | 0,490014 |
| ENSG0000 | -0,002578 | -0,369058 | -2,14219 | 0,032603 | 0,490014 |
| ENSG0000 | -0,000572 | -0,085758 | -2,141614 | 0,03265 | 0,490014 |
| ENSG0000 | 0,00296 | 2,581049 | 2,141203 | 0,032683 | 0,490014 |
| ENSG0000 | -0,001873 | 0,063294 | -2,140857 | 0,032711 | 0,490014 |
| ENSG0000 | -0,00317 | -0,398316 | -2,140682 | 0,032725 | 0,490014 |
| ENSG0000 | -0,00542 | 0,35087 | -2,139979 | 0,032782 | 0,490014 |
| ENSG0000 | 0,008225 | -2,267258 | 2,139184 | 0,032847 | 0,490014 |
| ENSG0000 | -0,003421 | 0,478176 | -2,138827 | 0,032876 | 0,490014 |
| ENSG0000 | 0,002927 | -0,226946 | 2,13797 | 0,032946 | 0,490014 |
| ENSG0000 | 0,001949 | -0,236334 | 2,137902 | 0,032951 | 0,490014 |
| ENSG0000 | -0,001786 | -0,162328 | -2,137156 | 0,033012 | 0,490014 |
| ENSG0000 | 0,002345 | 0,079515 | 2,13676 | 0,033045 | 0,490014 |
| ENSG0000 | 0,006314 | 0,908224 | 2,132438 | 0,0334 | 0,494388 |
| ENSG0000 | 0,003048 | 0,6047 | 2,130095 | 0,033594 | 0,496364 |
| ENSG0000 | -0,004511 | 0,861092 | -2,12844 | 0,033731 | 0,496668 |
| ENSG0000 | 0,001367 | 0,069215 | 2,128058 | 0,033763 | 0,496668 |
| ENSG0000 | 0,002904 | 0,446132 | 2,127362 | 0,033821 | 0,496668 |
| ENSG0000 | -0,002739 | 0,567077 | -2,126428 | 0,033899 | 0,496668 |
| ENSG0000 | 0,002709 | 0,291318 | 2,126224 | 0,033916 | 0,496668 |
| ENSG0000 | 0,003724 | 0,390275 | 2,125156 | 0,034005 | 0,497092 |
| ENSG0000 | 0,002172 | -0,114875 | 2,124309 | 0,034076 | 0,497248 |
| ENSG0000 | -0,00285 | -0,794406 | -2,122734 | 0,034209 | 0,498296 |
| ENSG0000 | 0,006675 | 1,358989 | 2,121704 | 0,034296 | 0,498679 |
| ENSG0000 | 0,003577 | 0,063476 | 2,119127 | 0,034514 | 0,500966 |
| ENSG0000 | -0,002438 | 0,376106 | -2,117113 | 0,034685 | 0,502038 |
| ENSG0000 | 0,002735 | 0,378439 | 2,114915 | 0,034873 | 0,502038 |
| ENSG0000 | 0,002627 | 0,009494 | 2,113773 | 0,034971 | 0,502038 |
| ENSG0000 | 0,001554 | 0,172969 | 2,113635 | 0,034982 | 0,502038 |
| ENSG0000 | 0,002322 | -0,331415 | 2,113417 | 0,035001 | 0,502038 |
| ENSG0000 | 0,003377 | -0,549302 | 2,112763 | 0,035057 | 0,502038 |
| ENSG0000 | -0,001382 | -0,6991 | -2,112405 | 0,035088 | 0,502038 |
| ENSG0000 | 0,00637 | 0,765732 | 2,111738 | 0,035146 | 0,502038 |
| ENSG0000 | 0,003255 | 0,10131 | 2,110602 | 0,035244 | 0,502038 |

| | | | | | |
|---|---|---|---|---|---|
| ENSG0000 | -0,00239 | 0,367974 | -2,110483 | 0,035254 | 0,502038 |
| ENSG0000 | 0,00385 | 1,264293 | 2,11036 | 0,035265 | 0,502038 |
| ENSG0000 | -0,0055 | 1,274134 | -2,109725 | 0,03532 | 0,502038 |
| ENSG0000 | 0,005108 | -0,655049 | 2,107879 | 0,03548 | 0,502857 |
| ENSG0000 | 0,002139 | 0,450323 | 2,107617 | 0,035502 | 0,502857 |
| ENSG0000 | -0,004604 | 0,574171 | -2,106949 | 0,03556 | 0,502857 |
| ENSG0000 | 0,002252 | 0,501136 | 2,104644 | 0,035762 | 0,504834 |
| ENSG0000 | -0,004283 | -0,383325 | -2,101818 | 0,03601 | 0,507465 |
| ENSG0000 | 0,004774 | 0,654781 | 2,100553 | 0,036121 | 0,508166 |
| ENSG0000 | 0,001933 | 0,485757 | 2,099169 | 0,036243 | 0,509015 |
| ENSG0000 | -0,005371 | -0,396537 | -2,096391 | 0,03649 | 0,511605 |
| ENSG0000 | -0,003743 | 0,081998 | -2,095363 | 0,036582 | 0,512017 |
| ENSG0000 | 0,004901 | 0,749507 | 2,093897 | 0,036712 | 0,512196 |
| ENSG0000 | -0,00319 | 0,962945 | -2,093827 | 0,036719 | 0,512196 |
| ENSG0000 | 0,005044 | -1,459673 | 2,092459 | 0,036841 | 0,512735 |
| ENSG0000 | -0,001879 | 0,147266 | -2,090994 | 0,036973 | 0,512735 |
| ENSG0000 | -0,001542 | 0,255303 | -2,090611 | 0,037007 | 0,512735 |
| ENSG0000 | -0,005518 | -1,405946 | -2,089613 | 0,037097 | 0,512735 |
| ENSG0000 | -0,002506 | -0,208244 | -2,089449 | 0,037112 | 0,512735 |
| ENSG0000 | 0,007199 | 0,56683 | 2,089239 | 0,037131 | 0,512735 |
| ENSG0000 | 0,003238 | -0,077635 | 2,087014 | 0,037333 | 0,514559 |
| ENSG0000 | -0,004168 | -0,089484 | -2,085805 | 0,037443 | 0,514559 |
| ENSG0000 | -0,003627 | 0,063025 | -2,08492 | 0,037523 | 0,514559 |
| ENSG0000 | 0,00344 | -0,217804 | 2,084698 | 0,037543 | 0,514559 |
| ENSG0000 | 0,002195 | 0,531664 | 2,083736 | 0,037631 | 0,514559 |
| ENSG0000 | -0,004215 | -0,307895 | -2,083419 | 0,03766 | 0,514559 |
| ENSG0000 | 0,00505 | -0,857235 | 2,081437 | 0,037842 | 0,514559 |
| ENSG0000 | -0,000386 | 0,022952 | -2,081304 | 0,037854 | 0,514559 |
| ENSG0000 | 0,005283 | -0,380893 | 2,080951 | 0,037887 | 0,514559 |
| ENSG0000 | 0,00287 | -0,436395 | 2,080404 | 0,037937 | 0,514559 |
| ENSG0000 | 0,005371 | 0,431553 | 2,078921 | 0,038073 | 0,514559 |
| ENSG0000 | 0,0066 | -0,937468 | 2,078909 | 0,038075 | 0,514559 |
| ENSG0000 | 0,004866 | -1,151355 | 2,078775 | 0,038087 | 0,514559 |
| ENSG0000 | -0,003258 | 1,105269 | -2,078209 | 0,038139 | 0,514559 |
| ENSG0000 | -0,004796 | -0,862417 | -2,077164 | 0,038236 | 0,514559 |
| ENSG0000 | -0,001425 | 0,657971 | -2,075947 | 0,038349 | 0,514559 |
| ENSG0000 | 0,002308 | -0,79682 | 2,075078 | 0,03843 | 0,514559 |
| ENSG0000 | 0,005071 | 0,761329 | 2,074762 | 0,038459 | 0,514559 |
| ENSG0000 | -0,001952 | -0,340607 | -2,073873 | 0,038542 | 0,514559 |
| ENSG0000 | -0,002078 | 0,908823 | -2,073868 | 0,038542 | 0,514559 |
| ENSG0000 | -0,003535 | 0,642457 | -2,073159 | 0,038608 | 0,514559 |
| ENSG0000 | -0,003726 | -0,561213 | -2,069691 | 0,038933 | 0,514559 |
| ENSG0000 | -0,002225 | 0,193118 | -2,069537 | 0,038948 | 0,514559 |
| ENSG0000 | -0,004488 | -0,091305 | -2,068452 | 0,03905 | 0,514559 |
| ENSG0000 | -0,004157 | 0,979688 | -2,068049 | 0,039088 | 0,514559 |
| ENSG0000 | 0,007139 | 1,518012 | 2,067938 | 0,039099 | 0,514559 |
| ENSG0000 | -0,008229 | -1,485992 | -2,06791 | 0,039101 | 0,514559 |
| ENSG0000 | -0,004415 | 0,313777 | -2,067843 | 0,039108 | 0,514559 |

| | | | | | |
|---|---|---|---|---|---|
| ENSG0000 | 0,002226 | 0,158398 | 2,067467 | 0,039143 | 0,514559 |
| ENSG0000 | -0,002572 | 0,353458 | -2,066399 | 0,039244 | 0,514559 |
| ENSG0000 | -0,002797 | 1,74265 | -2,066333 | 0,03925 | 0,514559 |
| ENSG0000 | -0,000535 | -0,088314 | -2,065725 | 0,039308 | 0,514559 |
| ENSG0000 | 0,002997 | 0,326796 | 2,06553 | 0,039327 | 0,514559 |
| ENSG0000 | 0,002956 | 1,353878 | 2,063927 | 0,039479 | 0,514855 |
| ENSG0000 | 0,001679 | 0,109086 | 2,063173 | 0,039551 | 0,514855 |
| ENSG0000 | 0,000707 | -0,06 | 2,062731 | 0,039593 | 0,514855 |
| ENSG0000 | 0,001298 | 0,126824 | 2,062662 | 0,039599 | 0,514855 |
| ENSG0000 | 0,002297 | 0,095808 | 2,061322 | 0,039728 | 0,515706 |
| ENSG0000 | 0,001874 | 0,32371 | 2,059523 | 0,0399 | 0,516843 |
| ENSG0000 | 0,005807 | -0,617142 | 2,057693 | 0,040076 | 0,516843 |
| ENSG0000 | -0,002131 | 0,29988 | -2,055782 | 0,040261 | 0,516843 |
| ENSG0000 | 0,003304 | 0,178683 | 2,055253 | 0,040312 | 0,516843 |
| ENSG0000 | -0,004196 | 0,821765 | -2,054451 | 0,04039 | 0,516843 |
| ENSG0000 | -0,003971 | 0,299867 | -2,053932 | 0,04044 | 0,516843 |
| ENSG0000 | 0,002177 | 0,454162 | 2,053068 | 0,040524 | 0,516843 |
| ENSG0000 | 0,002244 | -0,402741 | 2,052884 | 0,040542 | 0,516843 |
| ENSG0000 | 0,007734 | -0,805996 | 2,052823 | 0,040548 | 0,516843 |
| ENSG0000 | -0,001977 | 0,229421 | -2,052606 | 0,040569 | 0,516843 |
| ENSG0000 | 0,000342 | -0,147542 | 2,051114 | 0,040715 | 0,516843 |
| ENSG0000 | -0,001752 | 0,195303 | -2,051061 | 0,04072 | 0,516843 |
| ENSG0000 | 0,00543 | 1,049015 | 2,05097 | 0,040729 | 0,516843 |
| ENSG0000 | -0,00407 | -2,814412 | -2,050705 | 0,040755 | 0,516843 |
| ENSG0000 | 0,003619 | 0,249359 | 2,05068 | 0,040757 | 0,516843 |
| ENSG0000 | -0,003374 | -0,455292 | -2,049191 | 0,040903 | 0,517478 |
| ENSG0000 | -0,003867 | -0,45673 | -2,048885 | 0,040933 | 0,517478 |
| ENSG0000 | -0,00551 | 0,165294 | -2,04724 | 0,041095 | 0,518726 |
| ENSG0000 | -0,006207 | 1,223268 | -2,044535 | 0,041362 | 0,520967 |
| ENSG0000 | 0,006851 | -0,39444 | 2,044162 | 0,041399 | 0,520967 |
| ENSG0000 | -0,004198 | -0,43249 | -2,043471 | 0,041467 | 0,521032 |
| ENSG0000 | -0,001714 | 0,072116 | -2,041248 | 0,041688 | 0,522565 |
| ENSG0000 | -0,003818 | -0,32962 | -2,040702 | 0,041743 | 0,522565 |
| ENSG0000 | -0,001077 | 0,156538 | -2,039196 | 0,041894 | 0,522565 |
| ENSG0000 | -0,004258 | -2,77E-05 | -2,038499 | 0,041963 | 0,522565 |
| ENSG0000 | 0,004171 | -0,643092 | 2,037992 | 0,042014 | 0,522565 |
| ENSG0000 | 0,004612 | 1,738808 | 2,037986 | 0,042015 | 0,522565 |
| ENSG0000 | 0,001566 | 0,515681 | 2,03694 | 0,04212 | 0,522565 |
| ENSG0000 | -0,002717 | -0,121522 | -2,036153 | 0,042199 | 0,522565 |
| ENSG0000 | 0,002972 | -0,292593 | 2,035478 | 0,042267 | 0,522565 |
| ENSG0000 | 0,005836 | -1,687372 | 2,035295 | 0,042286 | 0,522565 |
| ENSG0000 | 0,001127 | 0,135081 | 2,035273 | 0,042288 | 0,522565 |
| ENSG0000 | 0,002622 | 0,352952 | 2,032274 | 0,042591 | 0,525526 |
| ENSG0000 | 0,004142 | 0,416308 | 2,031646 | 0,042655 | 0,525526 |
| ENSG0000 | 0,003082 | 0,972031 | 2,02894 | 0,042931 | 0,527882 |
| ENSG0000 | 0,003447 | 0,523961 | 2,027721 | 0,043056 | 0,527882 |
| ENSG0000 | -0,001643 | -0,215897 | -2,027513 | 0,043077 | 0,527882 |
| ENSG0000 | 0,002161 | 0,874815 | 2,027261 | 0,043103 | 0,527882 |

| | | | | | |
|---|---|---|---|---|---|
| ENSG0000 | 0,000834 | 0,191924 | 2,025657 | 0,043268 | 0,528524 |
| ENSG0000 | 0,003332 | -1,022598 | 2,025129 | 0,043322 | 0,528524 |
| ENSG0000 | 0,001911 | 0,072306 | 2,024611 | 0,043375 | 0,528524 |
| ENSG0000 | -0,004077 | 0,653362 | -2,02255 | 0,043588 | 0,528524 |
| ENSG0000 | -0,001793 | 0,259315 | -2,022305 | 0,043614 | 0,528524 |
| ENSG0000 | -0,003566 | 0,218732 | -2,022244 | 0,04362 | 0,528524 |
| ENSG0000 | 0,00116 | -0,085775 | 2,021942 | 0,043651 | 0,528524 |
| ENSG0000 | 0,001263 | 0,130517 | 2,021771 | 0,043669 | 0,528524 |
| ENSG0000 | 0,002157 | 0,384732 | 2,019265 | 0,04393 | 0,528732 |
| ENSG0000 | 0,004013 | 0,500724 | 2,019012 | 0,043956 | 0,528732 |
| ENSG0000 | -0,001545 | 0,274641 | -2,01898 | 0,043959 | 0,528732 |
| ENSG0000 | -0,005778 | 1,284043 | -2,018183 | 0,044042 | 0,528732 |
| ENSG0000 | 0,002556 | -0,240606 | 2,017912 | 0,044071 | 0,528732 |
| ENSG0000 | -0,003021 | -0,032611 | -2,017728 | 0,04409 | 0,528732 |
| ENSG0000 | 0,003031 | -0,427597 | 2,017088 | 0,044157 | 0,528732 |
| ENSG0000 | 0,003499 | 0,411872 | 2,016547 | 0,044214 | 0,528732 |
| ENSG0000 | 0,004249 | -0,0968 | 2,016062 | 0,044264 | 0,528732 |
| ENSG0000 | 0,004179 | -1,973428 | 2,013684 | 0,044515 | 0,530948 |
| ENSG0000 | -0,006999 | -1,808709 | -2,010881 | 0,044811 | 0,53322 |
| ENSG0000 | 0,002334 | -0,116881 | 2,010656 | 0,044835 | 0,53322 |
| ENSG0000 | 0,000954 | 0,025633 | 2,009196 | 0,04499 | 0,534293 |
| ENSG0000 | 0,002433 | 0,777052 | 2,00806 | 0,045111 | 0,534957 |
| ENSG0000 | -0,002363 | -0,217485 | -2,006419 | 0,045286 | 0,535171 |
| ENSG0000 | 0,001652 | 0,142329 | 2,004372 | 0,045505 | 0,535171 |
| ENSG0000 | 0,001702 | 0,098041 | 2,003956 | 0,04555 | 0,535171 |
| ENSG0000 | 0,004465 | 2,590318 | 2,003738 | 0,045573 | 0,535171 |
| ENSG0000 | 0,003303 | 0,045566 | 2,00351 | 0,045598 | 0,535171 |
| ENSG0000 | 0,001618 | 1,097962 | 2,003254 | 0,045625 | 0,535171 |
| ENSG0000 | -0,003993 | 0,604574 | -2,002908 | 0,045663 | 0,535171 |
| ENSG0000 | -0,002946 | -0,105225 | -2,002725 | 0,045682 | 0,535171 |
| ENSG0000 | 0,002231 | -0,60724 | 2,002431 | 0,045714 | 0,535171 |
| ENSG0000 | -0,002965 | -1,137551 | -2,001241 | 0,045842 | 0,535461 |
| ENSG0000 | -0,001303 | -0,935139 | -2,000405 | 0,045933 | 0,535461 |
| ENSG0000 | 0,006552 | 1,114841 | 2,000395 | 0,045934 | 0,535461 |
| ENSG0000 | -0,006431 | 0,273678 | -1,998182 | 0,046174 | 0,536743 |
| ENSG0000 | 0,008785 | -2,103464 | 1,9981 | 0,046183 | 0,536743 |
| ENSG0000 | -0,002637 | -0,183984 | -1,997171 | 0,046284 | 0,536743 |
| ENSG0000 | -0,001351 | 0,436479 | -1,996398 | 0,046368 | 0,536743 |
| ENSG0000 | 0,0018 | -0,199972 | 1,996223 | 0,046387 | 0,536743 |
| ENSG0000 | -0,001092 | 0,067952 | -1,995785 | 0,046435 | 0,536743 |
| ENSG0000 | 0,004245 | 0,463268 | 1,993474 | 0,046688 | 0,538196 |
| ENSG0000 | -0,003593 | 0,956988 | -1,992976 | 0,046743 | 0,538196 |
| ENSG0000 | 0,008081 | 1,218072 | 1,992847 | 0,046757 | 0,538196 |
| ENSG0000 | 0,000894 | 0,00612 | 1,991561 | 0,046898 | 0,538268 |
| ENSG0000 | -0,002622 | 0,231594 | -1,991037 | 0,046956 | 0,538268 |
| ENSG0000 | 0,003898 | -1,179993 | 1,990471 | 0,047019 | 0,538268 |
| ENSG0000 | -0,001655 | -0,151066 | -1,990054 | 0,047065 | 0,538268 |
| ENSG0000 | 0,007459 | 0,511967 | 1,989729 | 0,047101 | 0,538268 |

| | | | | | |
|---|---|---|---|---|---|
| ENSG0000 | 0,003142 | -0,240409 | 1,98923 | 0,047156 | 0,538268 |
| ENSG0000 | 0,002799 | 0,069693 | 1,987848 | 0,047309 | 0,538853 |
| ENSG0000 | 0,004008 | -1,343007 | 1,987586 | 0,047338 | 0,538853 |
| ENSG0000 | -0,009327 | -2,520039 | -1,986463 | 0,047463 | 0,539373 |
| ENSG0000 | -0,002526 | -1,342545 | -1,984682 | 0,047661 | 0,539373 |
| ENSG0000 | -0,00271 | 0,008391 | -1,984609 | 0,047669 | 0,539373 |
| ENSG0000 | 0,00469 | 1,58112 | 1,984509 | 0,047681 | 0,539373 |
| ENSG0000 | -0,003665 | -0,343966 | -1,983994 | 0,047738 | 0,539373 |
| ENSG0000 | -0,003675 | -0,816663 | -1,983166 | 0,047831 | 0,539373 |
| ENSG0000 | 0,003927 | 0,0985 | 1,982056 | 0,047955 | 0,539373 |
| ENSG0000 | -0,001692 | -0,368407 | -1,981605 | 0,048006 | 0,539373 |
| ENSG0000 | -0,004095 | -0,200568 | -1,980794 | 0,048097 | 0,539373 |
| ENSG0000 | -0,004061 | 0,62013 | -1,980153 | 0,048169 | 0,539373 |
| ENSG0000 | -0,00382 | 0,806123 | -1,980101 | 0,048175 | 0,539373 |
| ENSG0000 | -0,003862 | 0,671696 | -1,980044 | 0,048181 | 0,539373 |
| ENSG0000 | -0,002204 | 0,121866 | -1,979365 | 0,048258 | 0,539373 |
| ENSG0000 | -0,001936 | -0,098412 | -1,978855 | 0,048316 | 0,539373 |
| ENSG0000 | -0,002611 | -0,42552 | -1,978403 | 0,048367 | 0,539373 |
| ENSG0000 | -0,002616 | -0,324152 | -1,976413 | 0,048592 | 0,540511 |
| ENSG0000 | -0,002395 | -0,112259 | -1,975518 | 0,048694 | 0,540511 |
| ENSG0000 | -0,005956 | -1,405255 | -1,975258 | 0,048723 | 0,540511 |
| ENSG0000 | 0,004644 | -0,848019 | 1,975187 | 0,048731 | 0,540511 |
| ENSG0000 | -0,003347 | -0,486116 | -1,973889 | 0,048879 | 0,541399 |
| ENSG0000 | 0,001164 | 0,241588 | 1,972566 | 0,04903 | 0,541399 |
| ENSG0000 | 0,002531 | 0,844399 | 1,972532 | 0,049034 | 0,541399 |
| ENSG0000 | -0,005385 | 0,186898 | -1,971806 | 0,049117 | 0,541399 |
| ENSG0000 | -0,003601 | -0,143378 | -1,971605 | 0,04914 | 0,541399 |
| ENSG0000 | 0,001706 | -0,225702 | 1,970986 | 0,049211 | 0,541455 |
| ENSG0000 | 0,001639 | 0,302592 | 1,970032 | 0,049321 | 0,541936 |
| ENSG0000 | 0,005229 | 0,242696 | 1,967904 | 0,049566 | 0,543253 |
| ENSG0000 | -0,001107 | -0,577544 | -1,967845 | 0,049573 | 0,543253 |
| ENSG0000 | 0,005 | 1,310446 | 1,966153 | 0,049768 | 0,544671 |
| ENSG0000 | -0,006324 | -0,866322 | -1,965533 | 0,04984 | 0,544733 |

| geneID | logFC | AveExpr | t | P.Value | adj.P.Val |
|---|---|---|---|---|---|
| ENSG0000 | 0,004342 | -0,619127 | 5,072113 | 5,33E-07 | 0,003495 |
| ENSG0000 | -0,0089 | -0,360946 | -4,639783 | 4,33E-06 | 0,014199 |
| ENSG0000 | -0,00313 | 0,08766 | -4,332015 | 1,75E-05 | 0,038162 |
| ENSG0000 | -0,010361 | -0,774147 | -4,204822 | 3,03E-05 | 0,049725 |
| ENSG0000 | 0,002935 | 0,156175 | 4,105329 | 4,63E-05 | 0,05594 |
| ENSG0000 | 0,005579 | 0,393023 | 4,081233 | 5,12E-05 | 0,05594 |
| ENSG0000 | -0,012044 | 3,780198 | -4,023093 | 6,52E-05 | 0,06105 |
| ENSG0000 | -0,00616 | -0,284431 | -3,943702 | 9,02E-05 | 0,073941 |
| ENSG0000 | 0,010074 | -0,03363 | 3,907999 | 0,000104 | 0,075937 |
| ENSG0000 | 0,002895 | 0,033496 | 3,813926 | 0,000152 | 0,086064 |
| ENSG0000 | -0,003828 | 0,303037 | -3,80209 | 0,000159 | 0,086064 |
| ENSG0000 | -0,008856 | 0,59717 | -3,798814 | 0,000161 | 0,086064 |
| ENSG0000 | 0,006 | -0,296173 | 3,770861 | 0,00018 | 0,086064 |
| ENSG0000 | 0,009087 | -0,623329 | 3,765054 | 0,000184 | 0,086064 |
| ENSG0000 | -0,001832 | -0,01297 | -3,726589 | 0,000213 | 0,093283 |
| ENSG0000 | -0,008507 | 0,069019 | -3,707302 | 0,00023 | 0,094215 |
| ENSG0000 | 0,0081 | 0,949492 | 3,67364 | 0,000262 | 0,097761 |
| ENSG0000 | 0,002279 | 0,662493 | 3,658321 | 0,000277 | 0,097761 |
| ENSG0000 | 0,00415 | -1,549446 | 3,652102 | 0,000284 | 0,097761 |
| ENSG0000 | -0,004756 | -0,942853 | -3,627724 | 0,000312 | 0,097761 |
| ENSG0000 | 0,002469 | 0,280148 | 3,61493 | 0,000327 | 0,097761 |
| ENSG0000 | 0,008244 | 1,429056 | 3,61409 | 0,000328 | 0,097761 |
| ENSG0000 | 0,006001 | -1,70614 | 3,588726 | 0,000361 | 0,10287 |
| ENSG0000 | 0,007619 | 0,007741 | 3,539328 | 0,000434 | 0,118517 |
| ENSG0000 | -0,007415 | -0,208425 | -3,507405 | 0,000488 | 0,124063 |
| ENSG0000 | -0,004199 | 0,26474 | -3,493466 | 0,000514 | 0,124063 |
| ENSG0000 | -0,00531 | -0,871922 | -3,491652 | 0,000517 | 0,124063 |
| ENSG0000 | 0,002565 | 0,160928 | 3,480235 | 0,000539 | 0,124063 |
| ENSG0000 | -0,013626 | 1,758868 | -3,475444 | 0,000549 | 0,124063 |
| ENSG0000 | 0,016597 | -0,382554 | 3,44576 | 0,000611 | 0,133601 |
| ENSG0000 | 0,004698 | -0,164184 | 3,418224 | 0,000675 | 0,142811 |
| ENSG0000 | 0,006272 | -0,276785 | 3,386246 | 0,000757 | 0,151526 |
| ENSG0000 | -0,007768 | 0,196527 | -3,381029 | 0,000772 | 0,151526 |
| ENSG0000 | -0,004107 | 0,0995 | -3,357953 | 0,000838 | 0,151526 |
| ENSG0000 | -0,004028 | -0,282039 | -3,346528 | 0,000872 | 0,151526 |
| ENSG0000 | -0,002564 | 0,321932 | -3,341217 | 0,000889 | 0,151526 |
| ENSG0000 | 0,006743 | 1,314 | 3,33314 | 0,000914 | 0,151526 |
| ENSG0000 | -0,004361 | -0,411546 | -3,332336 | 0,000917 | 0,151526 |
| ENSG0000 | -0,009714 | -0,96392 | -3,331776 | 0,000919 | 0,151526 |
| ENSG0000 | -0,001853 | 0,135801 | -3,33001 | 0,000925 | 0,151526 |
| ENSG0000 | 0,007066 | -0,116262 | 3,316115 | 0,000971 | 0,153066 |
| ENSG0000 | -0,006535 | -0,668499 | -3,31084 | 0,000989 | 0,153066 |
| ENSG0000 | 0,011926 | -0,489427 | 3,300633 | 0,001025 | 0,153066 |
| ENSG0000 | 0,006949 | -0,535859 | 3,292729 | 0,001054 | 0,153066 |
| ENSG0000 | 0,001859 | 0,124879 | 3,291534 | 0,001058 | 0,153066 |
| ENSG0000 | 0,002758 | -0,117323 | 3,280567 | 0,001099 | 0,153066 |
| ENSG0000 | 0,002263 | 0,170621 | 3,280068 | 0,001101 | 0,153066 |
| ENSG0000 | -0,004379 | -0,329511 | -3,269492 | 0,001142 | 0,153066 |

| | | | | | |
|---|---|---|---|---|---|
| ENSG0000 | -0,003447 | -0,129173 | -3,269054 | 0,001144 | 0,153066 |
| ENSG0000 | -0,003156 | -0,013846 | -3,230222 | 0,001308 | 0,168993 |
| ENSG0000 | -0,008578 | 0,304021 | -3,228776 | 0,001315 | 0,168993 |
| ENSG0000 | 0,007721 | -0,325398 | 3,213785 | 0,001384 | 0,171699 |
| ENSG0000 | -0,004954 | 0,973415 | -3,212909 | 0,001388 | 0,171699 |
| ENSG0000 | 0,008031 | -0,817614 | 3,198674 | 0,001457 | 0,176909 |
| ENSG0000 | -0,006229 | 0,40371 | -3,185223 | 0,001526 | 0,180566 |
| ENSG0000 | -0,009528 | 0,565706 | -3,178513 | 0,001561 | 0,180566 |
| ENSG0000 | 0,002124 | 0,159968 | 3,176733 | 0,00157 | 0,180566 |
| ENSG0000 | 0,009335 | -0,246368 | 3,171494 | 0,001598 | 0,180627 |
| ENSG0000 | -0,002017 | -0,254984 | -3,161411 | 0,001654 | 0,181854 |
| ENSG0000 | 0,00221 | 0,329778 | 3,159452 | 0,001665 | 0,181854 |
| ENSG0000 | 0,004856 | -0,354204 | 3,143023 | 0,001759 | 0,189038 |
| ENSG0000 | -0,005835 | 0,328648 | -3,129889 | 0,001838 | 0,192033 |
| ENSG0000 | 0,002719 | 0,221083 | 3,127864 | 0,001851 | 0,192033 |
| ENSG0000 | -0,005805 | 1,244688 | -3,123995 | 0,001875 | 0,192033 |
| ENSG0000 | -0,004264 | 0,027727 | -3,100798 | 0,002026 | 0,19297 |
| ENSG0000 | -0,00387 | 0,509039 | -3,100777 | 0,002026 | 0,19297 |
| ENSG0000 | -0,002217 | 0,135775 | -3,098326 | 0,002042 | 0,19297 |
| ENSG0000 | -0,004603 | 0,308074 | -3,098199 | 0,002043 | 0,19297 |
| ENSG0000 | -0,007006 | 0,382139 | -3,098072 | 0,002044 | 0,19297 |
| ENSG0000 | -0,010752 | -0,175079 | -3,093106 | 0,002078 | 0,19297 |
| ENSG0000 | -0,002788 | 0,242328 | -3,091326 | 0,00209 | 0,19297 |
| ENSG0000 | 0,001919 | -0,085386 | 3,07828 | 0,002182 | 0,198676 |
| ENSG0000 | 0,005104 | 0,832465 | 3,065069 | 0,002279 | 0,201718 |
| ENSG0000 | -0,003009 | -0,409106 | -3,061052 | 0,00231 | 0,201718 |
| ENSG0000 | -0,004947 | -0,43876 | -3,06046 | 0,002314 | 0,201718 |
| ENSG0000 | -0,005415 | 3,703992 | -3,055305 | 0,002354 | 0,201718 |
| ENSG0000 | -0,005817 | -0,541498 | -3,053249 | 0,00237 | 0,201718 |
| ENSG0000 | 0,002443 | 0,042343 | 3,04598 | 0,002427 | 0,203929 |
| ENSG0000 | -0,005357 | -0,004432 | -3,034983 | 0,002515 | 0,208713 |
| ENSG0000 | 0,004434 | -0,6775 | 3,028621 | 0,002568 | 0,210423 |
| ENSG0000 | -0,006848 | -0,926015 | -3,020695 | 0,002635 | 0,213253 |
| ENSG0000 | -0,015735 | 0,586731 | -3,015914 | 0,002676 | 0,213453 |
| ENSG0000 | 0,01015 | -0,431056 | 3,012892 | 0,002703 | 0,213453 |
| ENSG0000 | 0,003316 | -0,27036 | 3,005343 | 0,00277 | 0,216133 |
| ENSG0000 | -0,003565 | 0,594139 | -2,99337 | 0,002879 | 0,218567 |
| ENSG0000 | 0,002797 | 0,235764 | 2,992227 | 0,00289 | 0,218567 |
| ENSG0000 | -0,005051 | 1,140049 | -2,986496 | 0,002943 | 0,218567 |
| ENSG0000 | -0,003158 | -0,986804 | -2,983999 | 0,002967 | 0,218567 |
| ENSG0000 | -0,00501 | -1,467504 | -2,983953 | 0,002968 | 0,218567 |
| ENSG0000 | 0,006662 | 0,371734 | 2,9784 | 0,003021 | 0,220031 |
| ENSG0000 | -0,004003 | -0,371452 | -2,961658 | 0,003188 | 0,227936 |
| ENSG0000 | -0,009578 | 0,544205 | -2,960526 | 0,003199 | 0,227936 |
| ENSG0000 | -0,006636 | -1,020748 | -2,942317 | 0,00339 | 0,238965 |
| ENSG0000 | -0,004226 | 0,007877 | -2,934717 | 0,003473 | 0,239995 |
| ENSG0000 | -0,004807 | 0,491974 | -2,928775 | 0,003539 | 0,239995 |
| ENSG0000 | 0,002006 | -0,543857 | 2,927718 | 0,003551 | 0,239995 |

| | | | | | |
|---|---|---|---|---|---|
| ENSG0000 | -0,006559 | 0,594532 | -2,927694 | 0,003551 | 0,239995 |
| ENSG0000 | -0,013571 | 0,677681 | -2,912754 | 0,003723 | 0,249033 |
| ENSG0000 | 0,002237 | -0,563278 | 2,908116 | 0,003778 | 0,250148 |
| ENSG0000 | -0,004754 | 0,605235 | -2,899813 | 0,003878 | 0,25281 |
| ENSG0000 | -0,013026 | 1,033472 | -2,898395 | 0,003895 | 0,25281 |
| ENSG0000 | 0,000849 | 0,7327 | 2,884369 | 0,00407 | 0,261586 |
| ENSG0000 | 0,002854 | 0,28019 | 2,880731 | 0,004117 | 0,26201 |
| ENSG0000 | 0,003796 | 0,473776 | 2,87615 | 0,004176 | 0,262895 |
| ENSG0000 | 0,005543 | -0,737714 | 2,873493 | 0,004211 | 0,262895 |
| ENSG0000 | -0,000808 | 0,136252 | -2,854033 | 0,004474 | 0,276669 |
| ENSG0000 | 0,00718 | 0,748179 | 2,849125 | 0,004543 | 0,27687 |
| ENSG0000 | -0,003202 | 0,026621 | -2,84777 | 0,004562 | 0,27687 |
| ENSG0000 | 0,00567 | -0,526978 | 2,84233 | 0,004639 | 0,278987 |
| ENSG0000 | -0,009514 | 0,049278 | -2,833176 | 0,004772 | 0,284377 |
| ENSG0000 | 0,003941 | 0,554251 | 2,821651 | 0,004945 | 0,284604 |
| ENSG0000 | -0,001809 | -0,412035 | -2,818795 | 0,004988 | 0,284604 |
| ENSG0000 | -0,00716 | 0,505778 | -2,816373 | 0,005025 | 0,284604 |
| ENSG0000 | 0,010734 | 0,567156 | 2,813489 | 0,00507 | 0,284604 |
| ENSG0000 | 0,001291 | 0,146082 | 2,809546 | 0,005132 | 0,284604 |
| ENSG0000 | -0,009389 | 1,42455 | -2,808136 | 0,005154 | 0,284604 |
| ENSG0000 | 0,006776 | 0,568866 | 2,805915 | 0,005189 | 0,284604 |
| ENSG0000 | 0,004998 | 0,128004 | 2,801398 | 0,005261 | 0,284604 |
| ENSG0000 | 0,002578 | 0,583502 | 2,800062 | 0,005283 | 0,284604 |
| ENSG0000 | -0,003668 | -0,79799 | -2,798565 | 0,005307 | 0,284604 |
| ENSG0000 | 0,010091 | 0,99389 | 2,797504 | 0,005324 | 0,284604 |
| ENSG0000 | -0,010955 | 0,695313 | -2,796831 | 0,005335 | 0,284604 |
| ENSG0000 | 0,00246 | -0,283698 | 2,796509 | 0,00534 | 0,284604 |
| ENSG0000 | -0,004677 | 1,272623 | -2,791131 | 0,005429 | 0,285765 |
| ENSG0000 | 0,007197 | -1,026391 | 2,788151 | 0,005478 | 0,285765 |
| ENSG0000 | 0,004522 | 1,702555 | 2,787266 | 0,005493 | 0,285765 |
| ENSG0000 | -0,003937 | 0,057102 | -2,784203 | 0,005544 | 0,28617 |
| ENSG0000 | 0,003389 | 0,854787 | 2,77724 | 0,005663 | 0,289904 |
| ENSG0000 | 0,005665 | 0,100468 | 2,774056 | 0,005718 | 0,289904 |
| ENSG0000 | 0,003677 | 0,346479 | 2,768105 | 0,005822 | 0,289904 |
| ENSG0000 | 0,003423 | 0,455649 | 2,762903 | 0,005914 | 0,289904 |
| ENSG0000 | -0,009691 | 1,119758 | -2,758854 | 0,005987 | 0,289904 |
| ENSG0000 | -0,00113 | 0,200223 | -2,757301 | 0,006015 | 0,289904 |
| ENSG0000 | 0,005881 | 0,631345 | 2,752045 | 0,006111 | 0,289904 |
| ENSG0000 | 0,006647 | -0,677998 | 2,750945 | 0,006132 | 0,289904 |
| ENSG0000 | 0,001949 | -0,076456 | 2,750885 | 0,006133 | 0,289904 |
| ENSG0000 | 0,004992 | 1,182078 | 2,747162 | 0,006202 | 0,289904 |
| ENSG0000 | -0,006011 | 0,447273 | -2,741264 | 0,006313 | 0,289904 |
| ENSG0000 | 0,011721 | -0,227135 | 2,740652 | 0,006324 | 0,289904 |
| ENSG0000 | 0,006845 | 0,473411 | 2,739689 | 0,006343 | 0,289904 |
| ENSG0000 | -0,006441 | -0,449837 | -2,73961 | 0,006344 | 0,289904 |
| ENSG0000 | -0,002163 | -0,025407 | -2,737855 | 0,006378 | 0,289904 |
| ENSG0000 | -0,000907 | -0,309717 | -2,73743 | 0,006386 | 0,289904 |
| ENSG0000 | -0,00204 | -0,394513 | -2,735747 | 0,006418 | 0,289904 |

| | | | | | |
|---|---|---|---|---|---|
| ENSG0000 | -0,001956 | -0,120155 | -2,731642 | 0,006498 | 0,289904 |
| ENSG0000 | -0,003971 | -0,058592 | -2,729401 | 0,006541 | 0,289904 |
| ENSG0000 | 0,002986 | 0,708603 | 2,72456 | 0,006637 | 0,289904 |
| ENSG0000 | 0,00454 | 0,565658 | 2,724099 | 0,006646 | 0,289904 |
| ENSG0000 | 0,002539 | 0,005462 | 2,71978 | 0,006732 | 0,289904 |
| ENSG0000 | 0,002933 | 0,325843 | 2,719434 | 0,006739 | 0,289904 |
| ENSG0000 | -0,018609 | -4,02181 | -2,717732 | 0,006773 | 0,289904 |
| ENSG0000 | -0,003852 | 0,478318 | -2,715839 | 0,006812 | 0,289904 |
| ENSG0000 | 0,002075 | 0,236563 | 2,714335 | 0,006842 | 0,289904 |
| ENSG0000 | 0,002109 | -0,432218 | 2,713952 | 0,00685 | 0,289904 |
| ENSG0000 | 0,003683 | 0,393231 | 2,713707 | 0,006855 | 0,289904 |
| ENSG0000 | -0,005324 | -0,902015 | -2,704067 | 0,007055 | 0,294868 |
| ENSG0000 | 0,006406 | -1,450848 | 2,70369 | 0,007062 | 0,294868 |
| ENSG0000 | 0,003248 | 0,173025 | 2,699772 | 0,007145 | 0,295531 |
| ENSG0000 | -0,002256 | 0,149433 | -2,698172 | 0,007179 | 0,295531 |
| ENSG0000 | -0,010648 | 1,179806 | -2,695409 | 0,007238 | 0,295531 |
| ENSG0000 | 0,00209 | 0,39178 | 2,693737 | 0,007274 | 0,295531 |
| ENSG0000 | 0,006839 | -0,022547 | 2,69236 | 0,007304 | 0,295531 |
| ENSG0000 | 0,001932 | 0,076503 | 2,69015 | 0,007352 | 0,295644 |
| ENSG0000 | 0,002732 | 1,457992 | 2,687624 | 0,007407 | 0,296045 |
| ENSG0000 | 0,008442 | -0,603375 | 2,681353 | 0,007545 | 0,29768 |
| ENSG0000 | -0,004492 | 0,601406 | -2,681107 | 0,007551 | 0,29768 |
| ENSG0000 | 0,004304 | -0,272646 | 2,679419 | 0,007588 | 0,29768 |
| ENSG0000 | -0,006882 | -0,650071 | -2,677595 | 0,007629 | 0,29768 |
| ENSG0000 | -0,006055 | 0,78012 | -2,673727 | 0,007717 | 0,299309 |
| ENSG0000 | -0,002433 | 0,13257 | -2,670329 | 0,007794 | 0,300539 |
| ENSG0000 | -0,004511 | 1,269634 | -2,666115 | 0,007891 | 0,302504 |
| ENSG0000 | -0,004086 | 0,456336 | -2,660352 | 0,008026 | 0,305243 |
| ENSG0000 | -0,003115 | 0,383016 | -2,659081 | 0,008056 | 0,305243 |
| ENSG0000 | -0,004292 | -0,376235 | -2,652957 | 0,008202 | 0,307271 |
| ENSG0000 | -0,003692 | -0,675899 | -2,652894 | 0,008203 | 0,307271 |
| ENSG0000 | -0,004067 | -1,325858 | -2,648461 | 0,00831 | 0,307428 |
| ENSG0000 | -0,002662 | -0,549373 | -2,647822 | 0,008326 | 0,307428 |
| ENSG0000 | -0,004193 | -0,886348 | -2,645085 | 0,008393 | 0,307428 |
| ENSG0000 | -0,002211 | -0,067555 | -2,643724 | 0,008426 | 0,307428 |
| ENSG0000 | 0,007011 | 0,615145 | 2,640918 | 0,008495 | 0,307428 |
| ENSG0000 | -0,003287 | 0,338645 | -2,63825 | 0,008562 | 0,307428 |
| ENSG0000 | -0,008482 | -0,062682 | -2,636321 | 0,00861 | 0,307428 |
| ENSG0000 | 0,004231 | 1,744317 | 2,634025 | 0,008667 | 0,307428 |
| ENSG0000 | 0,00405 | -0,41675 | 2,631199 | 0,008739 | 0,307428 |
| ENSG0000 | -0,007577 | 0,381337 | -2,629099 | 0,008792 | 0,307428 |
| ENSG0000 | 0,0027 | -0,219898 | 2,628008 | 0,00882 | 0,307428 |
| ENSG0000 | 0,005347 | -0,750308 | 2,625246 | 0,008891 | 0,307428 |
| ENSG0000 | 0,003976 | 0,478466 | 2,623554 | 0,008935 | 0,307428 |
| ENSG0000 | 0,002069 | 0,539265 | 2,623136 | 0,008946 | 0,307428 |
| ENSG0000 | -0,005703 | 0,042404 | -2,621284 | 0,008994 | 0,307428 |
| ENSG0000 | 0,006716 | 0,767119 | 2,620165 | 0,009023 | 0,307428 |
| ENSG0000 | 0,001821 | -0,039671 | 2,619605 | 0,009038 | 0,307428 |

| | | | | | |
|---|---|---|---|---|---|
| ENSG0000 | 0,002863 | -0,087308 | 2,61608 | 0,009131 | 0,307428 |
| ENSG0000 | -0,001905 | -0,202558 | -2,613458 | 0,0092 | 0,307428 |
| ENSG0000 | -0,002465 | -0,163922 | -2,612392 | 0,009229 | 0,307428 |
| ENSG0000 | -0,010725 | -1,162805 | -2,612275 | 0,009232 | 0,307428 |
| ENSG0000 | 0,001145 | -0,100588 | 2,610645 | 0,009275 | 0,307428 |
| ENSG0000 | -0,000681 | -0,095617 | -2,610239 | 0,009286 | 0,307428 |
| ENSG0000 | -0,004552 | -0,078158 | -2,607403 | 0,009362 | 0,308397 |
| ENSG0000 | -0,004193 | 0,329876 | -2,603676 | 0,009464 | 0,308968 |
| ENSG0000 | 0,004017 | -0,189192 | 2,602042 | 0,009508 | 0,308968 |
| ENSG0000 | 0,002956 | 0,071664 | 2,601573 | 0,009521 | 0,308968 |
| ENSG0000 | -0,000932 | 0,063698 | -2,597141 | 0,009643 | 0,311393 |
| ENSG0000 | -0,003795 | -0,318593 | -2,594194 | 0,009725 | 0,311635 |
| ENSG0000 | -0,001321 | -0,145899 | -2,592395 | 0,009776 | 0,311635 |
| ENSG0000 | -0,00411 | -0,380595 | -2,589987 | 0,009844 | 0,311635 |
| ENSG0000 | -0,0069 | 0,659788 | -2,589193 | 0,009866 | 0,311635 |
| ENSG0000 | -0,001775 | 0,115285 | -2,587346 | 0,009919 | 0,311635 |
| ENSG0000 | -0,003885 | 0,15646 | -2,586729 | 0,009936 | 0,311635 |
| ENSG0000 | 0,004145 | 0,002153 | 2,581845 | 0,010076 | 0,313049 |
| ENSG0000 | -0,002365 | 0,366445 | -2,581824 | 0,010077 | 0,313049 |
| ENSG0000 | -0,007005 | -0,426771 | -2,571696 | 0,010373 | 0,320139 |
| ENSG0000 | -0,005275 | 0,311112 | -2,570018 | 0,010423 | 0,320139 |
| ENSG0000 | -0,002536 | -0,318015 | -2,569046 | 0,010452 | 0,320139 |
| ENSG0000 | -0,00878 | 0,390187 | -2,564641 | 0,010584 | 0,320712 |
| ENSG0000 | 0,003448 | -0,123324 | 2,564525 | 0,010587 | 0,320712 |
| ENSG0000 | -0,007003 | -1,105629 | -2,561511 | 0,010678 | 0,320712 |
| ENSG0000 | -0,005201 | -0,0974 | -2,561187 | 0,010688 | 0,320712 |
| ENSG0000 | 0,000671 | 0,063561 | 2,558792 | 0,010761 | 0,320712 |
| ENSG0000 | 0,004309 | 0,131357 | 2,558707 | 0,010764 | 0,320712 |
| ENSG0000 | -0,001765 | 0,400631 | -2,555492 | 0,010863 | 0,322191 |
| ENSG0000 | 0,007433 | 0,309893 | 2,549756 | 0,011041 | 0,325457 |
| ENSG0000 | -0,005218 | -1,795105 | -2,547476 | 0,011112 | 0,325457 |
| ENSG0000 | 0,004639 | 0,807732 | 2,547085 | 0,011125 | 0,325457 |
| ENSG0000 | -0,006722 | 0,349966 | -2,545373 | 0,011179 | 0,325457 |
| ENSG0000 | -0,004448 | 1,871426 | -2,542241 | 0,011278 | 0,325457 |
| ENSG0000 | 0,003202 | -0,34748 | 2,541506 | 0,011302 | 0,325457 |
| ENSG0000 | 0,004622 | -1,947147 | 2,540928 | 0,01132 | 0,325457 |
| ENSG0000 | 0,002887 | -0,429526 | 2,53651 | 0,011462 | 0,328106 |
| ENSG0000 | 0,001279 | -0,044066 | 2,533008 | 0,011576 | 0,329924 |
| ENSG0000 | -0,003723 | -0,338266 | -2,529129 | 0,011704 | 0,332107 |
| ENSG0000 | 0,004985 | -0,290145 | 2,521402 | 0,011961 | 0,333771 |
| ENSG0000 | 0,003681 | -0,431919 | 2,520129 | 0,012004 | 0,333771 |
| ENSG0000 | 0,001863 | 0,494663 | 2,51936 | 0,01203 | 0,333771 |
| ENSG0000 | -0,003684 | -0,993636 | -2,518696 | 0,012052 | 0,333771 |
| ENSG0000 | -0,004429 | 0,675285 | -2,518581 | 0,012056 | 0,333771 |
| ENSG0000 | -0,004106 | -0,256418 | -2,51761 | 0,012089 | 0,333771 |
| ENSG0000 | 0,007046 | -1,027076 | 2,516733 | 0,012119 | 0,333771 |
| ENSG0000 | -0,00477 | 0,801332 | -2,513873 | 0,012216 | 0,335051 |
| ENSG0000 | -0,001983 | -0,431949 | -2,510653 | 0,012327 | 0,336679 |

| | | | | | |
|---|---|---|---|---|---|
| ENSG0000 | 0,008307 | 0,414375 | 2,507607 | 0,012432 | 0,338154 |
| ENSG0000 | 0,00374 | -0,087825 | 2,506107 | 0,012485 | 0,338172 |
| ENSG0000 | -0,004933 | -0,856883 | -2,504246 | 0,01255 | 0,338537 |
| ENSG0000 | -0,007272 | 1,862805 | -2,494916 | 0,012881 | 0,344048 |
| ENSG0000 | -0,004277 | 0,048338 | -2,494191 | 0,012907 | 0,344048 |
| ENSG0000 | 0,002417 | 0,099687 | 2,492902 | 0,012953 | 0,344048 |
| ENSG0000 | -0,002459 | -0,180051 | -2,489232 | 0,013086 | 0,344048 |
| ENSG0000 | -0,00261 | -0,117738 | -2,487338 | 0,013156 | 0,344048 |
| ENSG0000 | -0,004272 | 0,340374 | -2,486484 | 0,013187 | 0,344048 |
| ENSG0000 | -0,004787 | 0,364701 | -2,486004 | 0,013204 | 0,344048 |
| ENSG0000 | 0,002846 | -1,343907 | 2,481509 | 0,01337 | 0,344048 |
| ENSG0000 | 0,004898 | -0,194728 | 2,481304 | 0,013378 | 0,344048 |
| ENSG0000 | 0,002415 | 0,420679 | 2,480641 | 0,013403 | 0,344048 |
| ENSG0000 | -0,003643 | 1,115786 | -2,479683 | 0,013438 | 0,344048 |
| ENSG0000 | 0,0034 | 1,254717 | 2,478678 | 0,013476 | 0,344048 |
| ENSG0000 | 0,003372 | 1,483233 | 2,477973 | 0,013502 | 0,344048 |
| ENSG0000 | 0,005412 | -1,5017 | 2,475624 | 0,01359 | 0,344048 |
| ENSG0000 | -0,006878 | -1,742444 | -2,47533 | 0,013601 | 0,344048 |
| ENSG0000 | 0,0066 | -0,448777 | 2,473882 | 0,013656 | 0,344048 |
| ENSG0000 | -0,007627 | 1,211025 | -2,468156 | 0,013874 | 0,344048 |
| ENSG0000 | 0,002433 | -0,09812 | 2,465946 | 0,013959 | 0,344048 |
| ENSG0000 | -0,00852 | 0,737476 | -2,463373 | 0,014058 | 0,344048 |
| ENSG0000 | 0,003297 | -0,940512 | 2,463278 | 0,014062 | 0,344048 |
| ENSG0000 | 0,003556 | -1,19834 | 2,461125 | 0,014146 | 0,344048 |
| ENSG0000 | 0,002931 | -0,812166 | 2,460768 | 0,01416 | 0,344048 |
| ENSG0000 | 0,005865 | 0,283351 | 2,45982 | 0,014197 | 0,344048 |
| ENSG0000 | 0,004012 | -0,36836 | 2,459518 | 0,014209 | 0,344048 |
| ENSG0000 | 0,00257 | 0,662669 | 2,459498 | 0,014209 | 0,344048 |
| ENSG0000 | 0,003833 | 0,758932 | 2,458443 | 0,014251 | 0,344048 |
| ENSG0000 | -0,00127 | 0,347356 | -2,457017 | 0,014307 | 0,344048 |
| ENSG0000 | -0,004097 | -0,06806 | -2,456486 | 0,014328 | 0,344048 |
| ENSG0000 | 0,00329 | -0,317667 | 2,456127 | 0,014342 | 0,344048 |
| ENSG0000 | 0,006162 | -0,660059 | 2,454974 | 0,014388 | 0,344048 |
| ENSG0000 | 0,001769 | 0,009745 | 2,454675 | 0,014399 | 0,344048 |
| ENSG0000 | 0,002685 | 0,146229 | 2,453413 | 0,01445 | 0,344048 |
| ENSG0000 | -0,003856 | -0,931298 | -2,45249 | 0,014486 | 0,344048 |
| ENSG0000 | -0,006811 | -2,40548 | -2,448391 | 0,01465 | 0,345911 |
| ENSG0000 | 0,001937 | 0,477612 | 2,447897 | 0,01467 | 0,345911 |
| ENSG0000 | -0,004215 | -0,809921 | -2,445953 | 0,014749 | 0,346115 |
| ENSG0000 | -0,00366 | 0,299051 | -2,44507 | 0,014784 | 0,346115 |
| ENSG0000 | -0,004212 | 0,148131 | -2,442248 | 0,014899 | 0,346776 |
| ENSG0000 | 0,001401 | 0,261471 | 2,441779 | 0,014918 | 0,346776 |
| ENSG0000 | 0,002644 | -0,070216 | 2,438915 | 0,015036 | 0,348272 |
| ENSG0000 | -0,004635 | 0,542307 | -2,433005 | 0,015281 | 0,350874 |
| ENSG0000 | 0,004151 | 0,836887 | 2,431591 | 0,01534 | 0,350874 |
| ENSG0000 | 0,002264 | 0,14902 | 2,43102 | 0,015364 | 0,350874 |
| ENSG0000 | 0,002211 | -0,532052 | 2,430972 | 0,015366 | 0,350874 |
| ENSG0000 | 0,005272 | -0,093719 | 2,429785 | 0,015416 | 0,350874 |

| | | | | | |
|---|---|---|---|---|---|
| ENSG0000 | 0,001833 | -0,302803 | 2,422301 | 0,015734 | 0,356868 |
| ENSG0000 | -0,007786 | 0,493352 | -2,420335 | 0,015818 | 0,357547 |
| ENSG0000 | 0,004269 | 0,219647 | 2,417184 | 0,015954 | 0,359386 |
| ENSG0000 | 0,003668 | 0,27866 | 2,414971 | 0,016051 | 0,360315 |
| ENSG0000 | -0,003757 | 0,437026 | -2,412283 | 0,016168 | 0,361716 |
| ENSG0000 | -0,00527 | -0,368853 | -2,409629 | 0,016285 | 0,363091 |
| ENSG0000 | 0,005698 | 0,940892 | 2,403998 | 0,016535 | 0,366283 |
| ENSG0000 | 0,003622 | 0,071048 | 2,403897 | 0,01654 | 0,366283 |
| ENSG0000 | -0,001896 | 0,190877 | -2,402016 | 0,016624 | 0,366913 |
| ENSG0000 | -0,004346 | -0,672349 | -2,396653 | 0,016867 | 0,371021 |
| ENSG0000 | -0,003564 | 0,059338 | -2,394063 | 0,016986 | 0,372376 |
| ENSG0000 | -0,005485 | 0,491768 | -2,392118 | 0,017075 | 0,373086 |
| ENSG0000 | -0,006089 | 0,773069 | -2,390384 | 0,017155 | 0,373327 |
| ENSG0000 | 0,006012 | 0,824168 | 2,389115 | 0,017214 | 0,373327 |
| ENSG0000 | 0,00199 | 0,206528 | 2,387365 | 0,017295 | 0,373327 |
| ENSG0000 | 0,013906 | 1,133838 | 2,386963 | 0,017314 | 0,373327 |
| ENSG0000 | -0,001761 | 0,244309 | -2,382138 | 0,01754 | 0,376965 |
| ENSG0000 | 0,002985 | 0,109348 | 2,380019 | 0,01764 | 0,377224 |
| ENSG0000 | -0,002221 | 0,20953 | -2,376434 | 0,017811 | 0,377224 |
| ENSG0000 | 0,002929 | -0,218122 | 2,376092 | 0,017827 | 0,377224 |
| ENSG0000 | -0,00586 | 0,408599 | -2,376044 | 0,017829 | 0,377224 |
| ENSG0000 | -0,005951 | -0,065047 | -2,374194 | 0,017918 | 0,377224 |
| ENSG0000 | 0,003316 | 0,692688 | 2,374141 | 0,017921 | 0,377224 |
| ENSG0000 | -0,002941 | -0,154291 | -2,37071 | 0,018086 | 0,377224 |
| ENSG0000 | -0,002642 | -0,009312 | -2,369164 | 0,018161 | 0,377224 |
| ENSG0000 | 0,003597 | -0,935195 | 2,366997 | 0,018267 | 0,377224 |
| ENSG0000 | -0,004381 | -0,998873 | -2,36669 | 0,018282 | 0,377224 |
| ENSG0000 | 0,002356 | 0,230147 | 2,366226 | 0,018304 | 0,377224 |
| ENSG0000 | 0,009013 | 0,058167 | 2,366161 | 0,018308 | 0,377224 |
| ENSG0000 | -0,008243 | 0,209943 | -2,365894 | 0,018321 | 0,377224 |
| ENSG0000 | -0,002342 | 0,141604 | -2,36514 | 0,018358 | 0,377224 |
| ENSG0000 | 0,003801 | -0,279356 | 2,359123 | 0,018655 | 0,382137 |
| ENSG0000 | 0,002681 | -0,772769 | 2,356718 | 0,018775 | 0,383398 |
| ENSG0000 | -0,001818 | -0,240017 | -2,352677 | 0,018978 | 0,384692 |
| ENSG0000 | -0,002185 | -0,19958 | -2,351757 | 0,019025 | 0,384692 |
| ENSG0000 | 0,004031 | 0,303498 | 2,349942 | 0,019117 | 0,384692 |
| ENSG0000 | 0,009193 | -0,369233 | 2,349791 | 0,019125 | 0,384692 |
| ENSG0000 | 0,004267 | -1,088443 | 2,349648 | 0,019132 | 0,384692 |
| ENSG0000 | 0,009433 | 1,422408 | 2,348139 | 0,019209 | 0,384797 |
| ENSG0000 | 0,002199 | -0,39394 | 2,347244 | 0,019255 | 0,384797 |
| ENSG0000 | -0,002635 | 0,643091 | -2,344778 | 0,019381 | 0,385021 |
| ENSG0000 | 0,004123 | 0,398394 | 2,344737 | 0,019383 | 0,385021 |
| ENSG0000 | 0,006254 | 0,75537 | 2,342602 | 0,019493 | 0,385879 |
| ENSG0000 | 0,006862 | 1,458167 | 2,341158 | 0,019568 | 0,385879 |
| ENSG0000 | 0,003286 | -0,335599 | 2,340488 | 0,019603 | 0,385879 |
| ENSG0000 | -0,00773 | 0,240013 | -2,335233 | 0,019878 | 0,389602 |
| ENSG0000 | -0,009503 | -0,249172 | -2,334603 | 0,019911 | 0,389602 |
| ENSG0000 | 0,00293 | -0,400368 | 2,330862 | 0,020109 | 0,391161 |

| | | | | | |
|---|---|---|---|---|---|
| ENSG0000 | 0,004174 | 0,42973 | 2,330843 | 0,02011 | 0,391161 |
| ENSG0000 | -0,002787 | -0,5341 | -2,327879 | 0,020268 | 0,393069 |
| ENSG0000 | -0,002652 | 0,351588 | -2,324039 | 0,020475 | 0,393928 |
| ENSG0000 | 0,004728 | -0,086301 | 2,324024 | 0,020475 | 0,393928 |
| ENSG0000 | 0,009492 | 0,162695 | 2,32153 | 0,02061 | 0,393928 |
| ENSG0000 | 0,002938 | 0,41212 | 2,320365 | 0,020674 | 0,393928 |
| ENSG0000 | 0,006115 | -1,761277 | 2,320274 | 0,020679 | 0,393928 |
| ENSG0000 | -0,005643 | 1,176441 | -2,317844 | 0,020811 | 0,393928 |
| ENSG0000 | 0,010607 | 1,752684 | 2,317761 | 0,020816 | 0,393928 |
| ENSG0000 | 0,002733 | 0,045914 | 2,317402 | 0,020836 | 0,393928 |
| ENSG0000 | -0,00635 | -0,621609 | -2,317082 | 0,020853 | 0,393928 |
| ENSG0000 | -0,00619 | 0,289305 | -2,309325 | 0,021283 | 0,400886 |
| ENSG0000 | 0,002909 | 0,553694 | 2,306964 | 0,021415 | 0,40115 |
| ENSG0000 | -0,002429 | 1,500439 | -2,306889 | 0,021419 | 0,40115 |
| ENSG0000 | -0,008129 | -0,290662 | -2,298978 | 0,021868 | 0,40672 |
| ENSG0000 | 0,002323 | 0,013726 | 2,297615 | 0,021946 | 0,40672 |
| ENSG0000 | -0,004872 | 0,748766 | -2,296658 | 0,022001 | 0,40672 |
| ENSG0000 | -0,004623 | -0,898661 | -2,296567 | 0,022006 | 0,40672 |
| ENSG0000 | 0,005309 | -0,912474 | 2,295573 | 0,022063 | 0,40672 |
| ENSG0000 | -0,003335 | 0,714443 | -2,295125 | 0,022089 | 0,40672 |
| ENSG0000 | -0,001446 | -0,370217 | -2,292172 | 0,02226 | 0,406967 |
| ENSG0000 | -0,004941 | -1,6277 | -2,290426 | 0,022361 | 0,406967 |
| ENSG0000 | -0,003569 | 0,762207 | -2,289456 | 0,022418 | 0,406967 |
| ENSG0000 | -0,008055 | -0,234463 | -2,28849 | 0,022475 | 0,406967 |
| ENSG0000 | 0,005804 | -1,072713 | 2,287483 | 0,022534 | 0,406967 |
| ENSG0000 | 0,001177 | 0,110391 | 2,286738 | 0,022577 | 0,406967 |
| ENSG0000 | -0,004749 | 1,185957 | -2,284132 | 0,022731 | 0,406967 |
| ENSG0000 | -0,003398 | -1,234389 | -2,284066 | 0,022735 | 0,406967 |
| ENSG0000 | -0,005569 | -0,23769 | -2,283363 | 0,022777 | 0,406967 |
| ENSG0000 | 0,002074 | 0,108088 | 2,282668 | 0,022818 | 0,406967 |
| ENSG0000 | -0,00503 | 0,763636 | -2,281968 | 0,022859 | 0,406967 |
| ENSG0000 | 0,007228 | -1,43057 | 2,281369 | 0,022895 | 0,406967 |
| ENSG0000 | 0,008615 | 1,232937 | 2,278958 | 0,023039 | 0,406967 |
| ENSG0000 | 0,003793 | -0,64586 | 2,277636 | 0,023118 | 0,406967 |
| ENSG0000 | 0,004432 | 0,21856 | 2,277601 | 0,02312 | 0,406967 |
| ENSG0000 | 0,001709 | -0,209811 | 2,277565 | 0,023123 | 0,406967 |
| ENSG0000 | 0,000988 | 0,123122 | 2,276979 | 0,023158 | 0,406967 |
| ENSG0000 | -0,00734 | -0,405471 | -2,273554 | 0,023365 | 0,409504 |
| ENSG0000 | -0,007854 | -0,840683 | -2,269071 | 0,023637 | 0,412155 |
| ENSG0000 | -0,002829 | -0,176413 | -2,267889 | 0,02371 | 0,412155 |
| ENSG0000 | -0,00697 | -1,090666 | -2,267327 | 0,023744 | 0,412155 |
| ENSG0000 | 0,003436 | 0,012104 | 2,265463 | 0,023859 | 0,412155 |
| ENSG0000 | -0,001275 | -0,364543 | -2,265442 | 0,023861 | 0,412155 |
| ENSG0000 | 0,005673 | 0,33491 | 2,263969 | 0,023952 | 0,412155 |
| ENSG0000 | 0,00221 | -0,750046 | 2,263527 | 0,023979 | 0,412155 |
| ENSG0000 | -0,001284 | 0,35255 | -2,262339 | 0,024053 | 0,412155 |
| ENSG0000 | 0,005487 | 0,519578 | 2,261872 | 0,024082 | 0,412155 |
| ENSG0000 | -0,003546 | 0,91548 | -2,260344 | 0,024177 | 0,412707 |

| | | | | | |
|---|---|---|---|---|---|
| ENSG0000 | -0,004619 | 1,278485 | -2,256799 | 0,024399 | 0,41366 |
| ENSG0000 | -0,005783 | 1,347006 | -2,254631 | 0,024536 | 0,41366 |
| ENSG0000 | 0,004653 | -0,789655 | 2,253545 | 0,024605 | 0,41366 |
| ENSG0000 | 0,001644 | -0,10219 | 2,253127 | 0,024631 | 0,41366 |
| ENSG0000 | 0,003275 | -0,229265 | 2,25199 | 0,024703 | 0,41366 |
| ENSG0000 | -0,007386 | -0,093094 | -2,250663 | 0,024788 | 0,41366 |
| ENSG0000 | -0,001053 | -0,082322 | -2,250112 | 0,024823 | 0,41366 |
| ENSG0000 | 0,008055 | -2,527901 | 2,248561 | 0,024922 | 0,41366 |
| ENSG0000 | -0,003343 | -0,271499 | -2,247521 | 0,024989 | 0,41366 |
| ENSG0000 | -0,006345 | -0,695651 | -2,246861 | 0,025031 | 0,41366 |
| ENSG0000 | -0,002885 | 0,280057 | -2,243911 | 0,025222 | 0,41366 |
| ENSG0000 | 0,003711 | -0,26588 | 2,24336 | 0,025257 | 0,41366 |
| ENSG0000 | -0,002643 | -0,650833 | -2,243212 | 0,025267 | 0,41366 |
| ENSG0000 | 0,00232 | 0,063734 | 2,242935 | 0,025285 | 0,41366 |
| ENSG0000 | -0,00533 | 0,657123 | -2,24203 | 0,025344 | 0,41366 |
| ENSG0000 | 0,003529 | -0,250046 | 2,241986 | 0,025347 | 0,41366 |
| ENSG0000 | -0,001648 | 0,224321 | -2,241124 | 0,025403 | 0,41366 |
| ENSG0000 | 0,002605 | 1,371145 | 2,240862 | 0,02542 | 0,41366 |
| ENSG0000 | -0,005961 | 0,446805 | -2,24068 | 0,025432 | 0,41366 |
| ENSG0000 | 0,004346 | -0,451738 | 2,23739 | 0,025647 | 0,41481 |
| ENSG0000 | -0,008616 | 1,000361 | -2,236256 | 0,025722 | 0,41481 |
| ENSG0000 | -0,006872 | 0,508421 | -2,236032 | 0,025736 | 0,41481 |
| ENSG0000 | -0,003274 | -0,414249 | -2,235742 | 0,025756 | 0,41481 |
| ENSG0000 | 0,004174 | 0,300284 | 2,22961 | 0,026163 | 0,419643 |
| ENSG0000 | 0,00261 | 0,930214 | 2,228037 | 0,026268 | 0,419643 |
| ENSG0000 | -0,003228 | 0,879026 | -2,22751 | 0,026303 | 0,419643 |
| ENSG0000 | -0,004815 | -0,488796 | -2,227386 | 0,026312 | 0,419643 |
| ENSG0000 | -0,001868 | -0,278684 | -2,225907 | 0,026411 | 0,420208 |
| ENSG0000 | -0,005526 | 0,125958 | -2,222327 | 0,026653 | 0,422629 |
| ENSG0000 | -0,002705 | -0,105172 | -2,220671 | 0,026766 | 0,422629 |
| ENSG0000 | 0,002469 | -0,368711 | 2,220128 | 0,026803 | 0,422629 |
| ENSG0000 | 0,001756 | -0,72379 | 2,219863 | 0,026821 | 0,422629 |
| ENSG0000 | 0,007448 | 0,063025 | 2,217718 | 0,026968 | 0,423023 |
| ENSG0000 | 0,003344 | -0,300944 | 2,216381 | 0,02706 | 0,423023 |
| ENSG0000 | 0,003333 | 0,413486 | 2,216046 | 0,027083 | 0,423023 |
| ENSG0000 | 0,001944 | -0,705204 | 2,215348 | 0,027131 | 0,423023 |
| ENSG0000 | 0,005561 | -0,046146 | 2,214801 | 0,027169 | 0,423023 |
| ENSG0000 | 0,004365 | -0,381546 | 2,211809 | 0,027376 | 0,425241 |
| ENSG0000 | 0,001216 | -0,079308 | 2,208062 | 0,027638 | 0,428289 |
| ENSG0000 | 0,003879 | -0,898126 | 2,20701 | 0,027712 | 0,42842 |
| ENSG0000 | -0,002951 | -0,086916 | -2,205814 | 0,027796 | 0,428512 |
| ENSG0000 | 0,00246 | 0,441882 | 2,205069 | 0,027848 | 0,428512 |
| ENSG0000 | 0,011584 | -1,612981 | 2,203923 | 0,027929 | 0,428752 |
| ENSG0000 | -0,004619 | -1,195141 | -2,202226 | 0,02805 | 0,429226 |
| ENSG0000 | -0,002105 | 0,156286 | -2,20164 | 0,028091 | 0,429226 |
| ENSG0000 | -0,001238 | -0,048286 | -2,199752 | 0,028226 | 0,430141 |
| ENSG0000 | 0,004596 | 1,105012 | 2,19896 | 0,028282 | 0,430141 |
| ENSG0000 | -0,002039 | 0,023269 | -2,197188 | 0,028409 | 0,430691 |

| | | | | | |
|---|---|---|---|---|---|
| ENSG0000 | -0,006418 | -1,078906 | -2,196517 | 0,028457 | 0,430691 |
| ENSG0000 | 0,003395 | -0,663648 | 2,195709 | 0,028516 | 0,430691 |
| ENSG0000 | -0,003751 | -0,798957 | -2,194552 | 0,028599 | 0,430958 |
| ENSG0000 | -0,005465 | 0,818835 | -2,192545 | 0,028744 | 0,432153 |
| ENSG0000 | 0,008885 | 1,191826 | 2,189097 | 0,028995 | 0,433454 |
| ENSG0000 | -0,004168 | 0,154803 | -2,188706 | 0,029024 | 0,433454 |
| ENSG0000 | 0,00368 | 0,517995 | 2,186831 | 0,029161 | 0,433454 |
| ENSG0000 | 0,003147 | -0,217607 | 2,186634 | 0,029176 | 0,433454 |
| ENSG0000 | 0,001921 | -0,218684 | 2,186056 | 0,029218 | 0,433454 |
| ENSG0000 | 0,003807 | -0,774994 | 2,185928 | 0,029228 | 0,433454 |
| ENSG0000 | 0,001571 | 0,263699 | 2,180995 | 0,029592 | 0,437722 |
| ENSG0000 | 0,002761 | 0,380151 | 2,180236 | 0,029649 | 0,437722 |
| ENSG0000 | 0,003154 | -1,114112 | 2,174768 | 0,030059 | 0,440278 |
| ENSG0000 | 0,008287 | -0,213653 | 2,174332 | 0,030091 | 0,440278 |
| ENSG0000 | 0,003293 | 0,186514 | 2,173893 | 0,030125 | 0,440278 |
| ENSG0000 | -0,003301 | 0,342935 | -2,173708 | 0,030139 | 0,440278 |
| ENSG0000 | 0,001491 | 0,482583 | 2,17258 | 0,030224 | 0,440278 |
| ENSG0000 | 0,002341 | -0,381595 | 2,172564 | 0,030225 | 0,440278 |
| ENSG0000 | 0,003355 | -0,114715 | 2,171467 | 0,030308 | 0,440511 |
| ENSG0000 | 0,001957 | 0,929468 | 2,169055 | 0,030492 | 0,442199 |
| ENSG0000 | -0,002522 | 0,616539 | -2,167292 | 0,030627 | 0,443049 |
| ENSG0000 | 0,002566 | 0,174164 | 2,165479 | 0,030766 | 0,443049 |
| ENSG0000 | 0,003286 | 0,336997 | 2,164533 | 0,030839 | 0,443049 |
| ENSG0000 | 0,001024 | -0,330758 | 2,16445 | 0,030845 | 0,443049 |
| ENSG0000 | -0,007423 | -0,446593 | -2,163886 | 0,030888 | 0,443049 |
| ENSG0000 | 0,004234 | 0,474528 | 2,162419 | 0,031002 | 0,443704 |
| ENSG0000 | 0,00562 | 0,081783 | 2,160992 | 0,031112 | 0,444317 |
| ENSG0000 | -0,001567 | 0,097117 | -2,160031 | 0,031187 | 0,444415 |
| ENSG0000 | 0,001839 | -0,658676 | 2,157461 | 0,031388 | 0,445748 |
| ENSG0000 | -0,002968 | 0,813359 | -2,1565 | 0,031463 | 0,445748 |
| ENSG0000 | 0,002617 | -0,787406 | 2,156223 | 0,031485 | 0,445748 |
| ENSG0000 | -0,005002 | 0,250962 | -2,155178 | 0,031567 | 0,445946 |
| ENSG0000 | 0,007562 | 0,282355 | 2,152257 | 0,031797 | 0,447407 |
| ENSG0000 | -0,001293 | 0,278387 | -2,151806 | 0,031833 | 0,447407 |
| ENSG0000 | -0,000632 | -0,084551 | -2,150762 | 0,031915 | 0,447407 |
| ENSG0000 | -0,004346 | 0,097789 | -2,150328 | 0,03195 | 0,447407 |
| ENSG0000 | -0,005611 | 0,162152 | -2,149555 | 0,032011 | 0,447407 |
| ENSG0000 | 0,002147 | 0,242651 | 2,144912 | 0,032382 | 0,451214 |
| ENSG0000 | -0,002964 | 0,239458 | -2,144429 | 0,032421 | 0,451214 |
| ENSG0000 | -0,001632 | 0,195072 | -2,143457 | 0,0325 | 0,451345 |
| ENSG0000 | 0,003013 | 0,512439 | 2,141746 | 0,032638 | 0,451583 |
| ENSG0000 | -0,002163 | 0,714955 | -2,140769 | 0,032717 | 0,451583 |
| ENSG0000 | -0,007686 | 0,799323 | -2,140689 | 0,032723 | 0,451583 |
| ENSG0000 | 0,007652 | 0,14576 | 2,138898 | 0,032869 | 0,452637 |
| ENSG0000 | 0,00517 | 0,46942 | 2,135913 | 0,033113 | 0,454171 |
| ENSG0000 | 0,002651 | -1,014094 | 2,135837 | 0,033119 | 0,454171 |
| ENSG0000 | -0,002484 | -1,147938 | -2,130349 | 0,033571 | 0,458558 |
| ENSG0000 | -0,000912 | -0,102575 | -2,129182 | 0,033668 | 0,458558 |

| | | | | | |
|---|---|---|---|---|---|
| ENSG0000 | 0,003384 | -0,232556 | 2,127795 | 0,033783 | 0,458558 |
| ENSG0000 | 0,002637 | 0,277442 | 2,127268 | 0,033827 | 0,458558 |
| ENSG0000 | -0,004173 | -0,761269 | -2,127029 | 0,033847 | 0,458558 |
| ENSG0000 | 0,002501 | 0,308129 | 2,126893 | 0,033858 | 0,458558 |
| ENSG0000 | 0,004224 | 1,815846 | 2,125121 | 0,034007 | 0,459615 |
| ENSG0000 | -0,007882 | 2,33791 | -2,123523 | 0,034141 | 0,460479 |
| ENSG0000 | 0,004409 | -2,186426 | 2,120869 | 0,034365 | 0,462516 |
| ENSG0000 | -0,005506 | -1,017507 | -2,120061 | 0,034433 | 0,462516 |
| ENSG0000 | 0,006875 | 0,518548 | 2,118422 | 0,034572 | 0,462622 |
| ENSG0000 | 0,004383 | -0,570622 | 2,117059 | 0,034688 | 0,462622 |
| ENSG0000 | 0,002829 | -0,010437 | 2,116813 | 0,034709 | 0,462622 |
| ENSG0000 | 0,001598 | 0,37602 | 2,115246 | 0,034843 | 0,462622 |
| ENSG0000 | 0,000618 | -0,001581 | 2,11519 | 0,034848 | 0,462622 |
| ENSG0000 | 0,006386 | 0,092845 | 2,114996 | 0,034864 | 0,462622 |
| ENSG0000 | -0,001075 | 0,072565 | -2,109141 | 0,035369 | 0,467405 |
| ENSG0000 | -0,005191 | 0,556937 | -2,108509 | 0,035423 | 0,467405 |
| ENSG0000 | -0,001672 | -0,086505 | -2,108073 | 0,035461 | 0,467405 |
| ENSG0000 | -0,003751 | -0,063358 | -2,107513 | 0,03551 | 0,467405 |
| ENSG0000 | 0,003587 | 0,886029 | 2,106113 | 0,035632 | 0,468069 |
| ENSG0000 | -0,003482 | -0,430724 | -2,104069 | 0,03581 | 0,468879 |
| ENSG0000 | 0,002784 | 1,047518 | 2,10347 | 0,035863 | 0,468879 |
| ENSG0000 | -0,002845 | -0,422041 | -2,101613 | 0,036026 | 0,468879 |
| ENSG0000 | 0,005969 | 0,79854 | 2,100891 | 0,03609 | 0,468879 |
| ENSG0000 | -0,004862 | -2,277135 | -2,099548 | 0,036208 | 0,468879 |
| ENSG0000 | 0,00245 | 0,07639 | 2,099511 | 0,036212 | 0,468879 |
| ENSG0000 | -0,005793 | -0,942796 | -2,099125 | 0,036246 | 0,468879 |
| ENSG0000 | 0,002688 | -0,547391 | 2,0989 | 0,036266 | 0,468879 |
| ENSG0000 | -0,002935 | -1,582421 | -2,098058 | 0,03634 | 0,468919 |
| ENSG0000 | -0,005226 | 1,063578 | -2,096768 | 0,036455 | 0,469263 |
| ENSG0000 | -0,002518 | -1,269738 | -2,096147 | 0,03651 | 0,469263 |
| ENSG0000 | 0,007103 | -0,707215 | 2,095015 | 0,036611 | 0,46957 |
| ENSG0000 | -0,001374 | -0,010965 | -2,093867 | 0,036714 | 0,46957 |
| ENSG0000 | 0,003523 | -0,779389 | 2,092356 | 0,036849 | 0,46957 |
| ENSG0000 | 0,003098 | 0,018245 | 2,091905 | 0,03689 | 0,46957 |
| ENSG0000 | -0,003383 | -0,229528 | -2,091875 | 0,036892 | 0,46957 |
| ENSG0000 | 0,00166 | 0,810205 | 2,085332 | 0,037484 | 0,476179 |
| ENSG0000 | -0,006013 | 0,17983 | -2,084388 | 0,03757 | 0,47635 |
| ENSG0000 | -0,004345 | 0,006853 | -2,0832 | 0,037679 | 0,476758 |
| ENSG0000 | -0,00175 | -0,479531 | -2,082445 | 0,037748 | 0,476758 |
| ENSG0000 | -0,004468 | -0,138614 | -2,078868 | 0,038077 | 0,476837 |
| ENSG0000 | -0,001673 | 0,748979 | -2,078105 | 0,038147 | 0,476837 |
| ENSG0000 | 0,005171 | 0,924198 | 2,078099 | 0,038148 | 0,476837 |
| ENSG0000 | -0,001865 | -0,175991 | -2,077855 | 0,03817 | 0,476837 |
| ENSG0000 | -0,001705 | -0,319718 | -2,076464 | 0,038299 | 0,476837 |
| ENSG0000 | 0,004432 | -0,724878 | 2,076094 | 0,038334 | 0,476837 |
| ENSG0000 | 0,003126 | -1,068736 | 2,07542 | 0,038396 | 0,476837 |
| ENSG0000 | -0,003608 | 0,349554 | -2,073638 | 0,038562 | 0,476837 |
| ENSG0000 | 0,002837 | -0,161775 | 2,073612 | 0,038565 | 0,476837 |

| | | | | | |
|---|---|---|---|---|---|
| ENSG0000 | -0,004921 | 0,602176 | -2,07223 | 0,038694 | 0,476837 |
| ENSG0000 | 0,00817 | 0,854636 | 2,071648 | 0,038748 | 0,476837 |
| ENSG0000 | -0,004494 | 0,676659 | -2,071115 | 0,038798 | 0,476837 |
| ENSG0000 | -0,002372 | 0,511234 | -2,071026 | 0,038806 | 0,476837 |
| ENSG0000 | -0,001942 | -0,212847 | -2,070624 | 0,038844 | 0,476837 |
| ENSG0000 | 0,002044 | -0,233211 | 2,070612 | 0,038845 | 0,476837 |
| ENSG0000 | 0,003834 | 0,620969 | 2,068357 | 0,039058 | 0,477793 |
| ENSG0000 | 0,003992 | 1,629701 | 2,068236 | 0,039069 | 0,477793 |
| ENSG0000 | -0,000698 | -0,12607 | -2,067324 | 0,039155 | 0,477954 |
| ENSG0000 | -0,004434 | 0,935602 | -2,066085 | 0,039272 | 0,478496 |
| ENSG0000 | 0,004325 | -0,606142 | 2,064923 | 0,039383 | 0,478948 |
| ENSG0000 | 0,005199 | 0,583557 | 2,064035 | 0,039467 | 0,478979 |
| ENSG0000 | 0,002422 | 0,427732 | 2,061951 | 0,039666 | 0,478979 |
| ENSG0000 | 0,004141 | 0,554283 | 2,061637 | 0,039696 | 0,478979 |
| ENSG0000 | -0,001311 | -0,861199 | -2,06149 | 0,03971 | 0,478979 |
| ENSG0000 | -0,006686 | -0,978315 | -2,05937 | 0,039913 | 0,478979 |
| ENSG0000 | 0,001434 | 0,252376 | 2,059293 | 0,03992 | 0,478979 |
| ENSG0000 | 0,002394 | 0,05789 | 2,058601 | 0,039987 | 0,478979 |
| ENSG0000 | -0,003862 | -0,061114 | -2,057921 | 0,040053 | 0,478979 |
| ENSG0000 | 0,00141 | -0,362 | 2,057463 | 0,040097 | 0,478979 |
| ENSG0000 | 0,006089 | 0,625679 | 2,057264 | 0,040116 | 0,478979 |
| ENSG0000 | -0,003812 | 1,476854 | -2,056275 | 0,040211 | 0,479247 |
| ENSG0000 | 0,003656 | -0,388435 | 2,054074 | 0,040425 | 0,480915 |
| ENSG0000 | -0,004162 | -0,23272 | -2,052168 | 0,04061 | 0,482114 |
| ENSG0000 | 0,007859 | 1,861563 | 2,051166 | 0,040708 | 0,482114 |
| ENSG0000 | -0,004226 | 0,372517 | -2,050173 | 0,040805 | 0,482114 |
| ENSG0000 | -0,003158 | -1,416141 | -2,049534 | 0,040868 | 0,482114 |
| ENSG0000 | 0,00392 | 0,706699 | 2,049274 | 0,040893 | 0,482114 |
| ENSG0000 | -0,008147 | 0,318679 | -2,047963 | 0,041022 | 0,482763 |
| ENSG0000 | 0,00399 | 0,537092 | 2,045259 | 0,041289 | 0,484813 |
| ENSG0000 | -0,004743 | -0,720741 | -2,044698 | 0,041344 | 0,484813 |
| ENSG0000 | 0,002903 | -0,111047 | 2,040785 | 0,041733 | 0,488122 |
| ENSG0000 | 0,004748 | 1,716786 | 2,040224 | 0,041789 | 0,488122 |
| ENSG0000 | -0,002157 | -0,435816 | -2,039618 | 0,04185 | 0,488122 |
| ENSG0000 | -0,00261 | -0,953932 | -2,036921 | 0,04212 | 0,490404 |
| ENSG0000 | 0,002754 | -0,615083 | 2,033668 | 0,042448 | 0,493349 |
| ENSG0000 | 0,00485 | 0,495442 | 2,031586 | 0,04266 | 0,494176 |
| ENSG0000 | 0,002189 | -0,20339 | 2,031482 | 0,04267 | 0,494176 |
| ENSG0000 | 0,002796 | -0,316578 | 2,029702 | 0,042852 | 0,4954 |
| ENSG0000 | -0,003638 | 0,494733 | -2,02667 | 0,043162 | 0,49811 |
| ENSG0000 | 0,002927 | -0,042503 | 2,019634 | 0,04389 | 0,504798 |
| ENSG0000 | 0,003262 | 0,03953 | 2,019577 | 0,043895 | 0,504798 |
| ENSG0000 | -0,00348 | -0,976781 | -2,018347 | 0,044024 | 0,505386 |
| ENSG0000 | 0,001775 | -0,51728 | 2,016275 | 0,044241 | 0,506262 |
| ENSG0000 | -0,000623 | -0,050643 | -2,016141 | 0,044255 | 0,506262 |
| ENSG0000 | 0,002411 | -0,014811 | 2,013733 | 0,044508 | 0,506578 |
| ENSG0000 | -0,005902 | 1,35365 | -2,012267 | 0,044662 | 0,506578 |
| ENSG0000 | 0,003443 | -0,822778 | 2,012222 | 0,044667 | 0,506578 |

| | | | | | |
|---|---|---|---|---|---|
| ENSG0000 | -0,001625 | -0,217656 | -2,01145 | 0,044749 | 0,506578 |
| ENSG0000 | 0,004605 | -0,903711 | 2,01045 | 0,044855 | 0,506578 |
| ENSG0000 | -0,001547 | 0,03506 | -2,009501 | 0,044956 | 0,506578 |
| ENSG0000 | -0,002664 | -0,983971 | -2,009479 | 0,044958 | 0,506578 |
| ENSG0000 | 0,002722 | -0,146924 | 2,008536 | 0,045058 | 0,506578 |
| ENSG0000 | -0,003257 | -0,471792 | -2,007865 | 0,04513 | 0,506578 |
| ENSG0000 | 0,003601 | 0,305449 | 2,007342 | 0,045186 | 0,506578 |
| ENSG0000 | 0,001066 | -0,184848 | 2,006868 | 0,045236 | 0,506578 |
| ENSG0000 | 0,005028 | 0,000816 | 2,006075 | 0,045321 | 0,506578 |
| ENSG0000 | 0,003332 | 0,179132 | 2,005568 | 0,045375 | 0,506578 |
| ENSG0000 | -0,003054 | -0,211774 | -2,005051 | 0,045431 | 0,506578 |
| ENSG0000 | 0,001976 | 0,347823 | 2,004952 | 0,045441 | 0,506578 |
| ENSG0000 | 0,003803 | -1,000919 | 2,003857 | 0,045559 | 0,507025 |
| ENSG0000 | 0,00264 | -0,391552 | 2,002488 | 0,045706 | 0,507052 |
| ENSG0000 | -0,001573 | 0,240191 | -2,001852 | 0,045775 | 0,507052 |
| ENSG0000 | -0,005205 | 0,859798 | -2,001176 | 0,045848 | 0,507052 |
| ENSG0000 | -0,002319 | -0,547066 | -2,000965 | 0,045871 | 0,507052 |
| ENSG0000 | -0,005122 | -0,161412 | -1,999946 | 0,045981 | 0,507414 |
| ENSG0000 | -0,002081 | -0,217143 | -1,998906 | 0,046094 | 0,507433 |
| ENSG0000 | 0,001534 | 0,259294 | 1,998504 | 0,046137 | 0,507433 |
| ENSG0000 | -0,002234 | 0,366919 | -1,994796 | 0,046542 | 0,509833 |
| ENSG0000 | 0,002368 | 0,522145 | 1,993424 | 0,046692 | 0,509833 |
| ENSG0000 | 0,003153 | 0,249932 | 1,993105 | 0,046727 | 0,509833 |
| ENSG0000 | 0,001945 | -0,112608 | 1,99291 | 0,046748 | 0,509833 |
| ENSG0000 | -0,004002 | 0,595821 | -1,992688 | 0,046773 | 0,509833 |
| ENSG0000 | -0,005294 | 0,059313 | -1,991519 | 0,046901 | 0,509833 |
| ENSG0000 | 0,003757 | -1,203148 | 1,990979 | 0,046961 | 0,509833 |
| ENSG0000 | 0,00444 | 0,01227 | 1,990826 | 0,046978 | 0,509833 |
| ENSG0000 | -0,00638 | -0,285882 | -1,989432 | 0,047132 | 0,510181 |
| ENSG0000 | 0,003839 | 1,894588 | 1,989127 | 0,047165 | 0,510181 |
| ENSG0000 | 0,006845 | 0,927364 | 1,988239 | 0,047264 | 0,510283 |
| ENSG0000 | -0,002864 | -0,146537 | -1,987099 | 0,04739 | 0,510283 |
| ENSG0000 | 0,002461 | -0,350198 | 1,986937 | 0,047408 | 0,510283 |
| ENSG0000 | -0,003035 | 0,321298 | -1,98606 | 0,047506 | 0,510494 |
| ENSG0000 | -0,003827 | -0,152934 | -1,983179 | 0,047828 | 0,512516 |
| ENSG0000 | 0,002036 | 0,663918 | 1,982749 | 0,047876 | 0,512516 |
| ENSG0000 | -0,003929 | 0,396488 | -1,982015 | 0,047958 | 0,512516 |
| ENSG0000 | -0,002849 | 0,096841 | -1,981319 | 0,048036 | 0,512516 |
| ENSG0000 | 0,004107 | -0,416443 | 1,980886 | 0,048085 | 0,512516 |
| ENSG0000 | 0,002672 | 0,243159 | 1,979401 | 0,048252 | 0,512851 |
| ENSG0000 | -0,00339 | 1,307442 | -1,979035 | 0,048294 | 0,512851 |
| ENSG0000 | -0,002831 | -1,07399 | -1,978525 | 0,048351 | 0,512851 |
| ENSG0000 | -0,00202 | 0,135691 | -1,976802 | 0,048546 | 0,514088 |
| ENSG0000 | -0,001781 | 0,529834 | -1,975999 | 0,048637 | 0,514223 |
| ENSG0000 | -0,002054 | 0,32046 | -1,975265 | 0,048721 | 0,514275 |
| ENSG0000 | 0,002942 | -0,107976 | 1,973544 | 0,048917 | 0,514374 |
| ENSG0000 | 0,003607 | 0,902447 | 1,972894 | 0,048991 | 0,514374 |
| ENSG0000 | -0,00483 | -1,442761 | -1,972764 | 0,049006 | 0,514374 |

| | | | | | |
|---|---|---|---|---|---|
| ENSG0000 | 0,003951 | 0,355555 | 1,972431 | 0,049044 | 0,514374 |
| ENSG0000 | 0,002861 | 0,693097 | 1,970126 | 0,049308 | 0,515312 |
| ENSG0000 | 0,001517 | -0,047766 | 1,969609 | 0,049368 | 0,515312 |
| ENSG0000 | -0,003636 | 0,022882 | -1,968802 | 0,049461 | 0,515312 |
| ENSG0000 | 0,002767 | -1,092778 | 1,968435 | 0,049503 | 0,515312 |
| ENSG0000 | -0,003239 | -0,496964 | -1,96823 | 0,049527 | 0,515312 |
| ENSG0000 | -0,001911 | 0,52514 | -1,967185 | 0,049647 | 0,515528 |
| ENSG0000 | 0,00484 | 0,555709 | 1,966036 | 0,04978 | 0,515528 |
| ENSG0000 | -0,005292 | 3,01552 | -1,965629 | 0,049827 | 0,515528 |
| ENSG0000 | -0,003524 | -1,142614 | -1,964798 | 0,049924 | 0,515528 |
| ENSG0000 | -0,002433 | 0,783909 | -1,964653 | 0,049941 | 0,515528 |

| geneID | logFC | AveExpr | t | P.Value | adj.P.Val |
|---|---|---|---|---|---|
| ENSG0000 | -0,005039 | -0,276357 | -4,816411 | 1,49E-06 | 0,012274 |
| ENSG0000 | 0,008454 | 2,188724 | 4,564881 | 5,07E-06 | 0,020919 |
| ENSG0000 | 0,003054 | 0,987098 | 4,393908 | 1,13E-05 | 0,023363 |
| ENSG0000 | 0,001621 | 0,175256 | 4,392917 | 1,13E-05 | 0,023363 |
| ENSG0000 | -0,00365 | -0,510186 | -4,148825 | 3,37E-05 | 0,05572 |
| ENSG0000 | -0,002641 | 0,251469 | -3,797629 | 0,000147 | 0,202434 |
| ENSG0000 | 0,006298 | 1,226515 | 3,693943 | 0,000222 | 0,252525 |
| ENSG0000 | 0,000479 | -0,054264 | 3,614865 | 0,000302 | 0,252525 |
| ENSG0000 | -0,002314 | 0,120207 | -3,611279 | 0,000306 | 0,252525 |
| ENSG0000 | -0,001377 | -0,241219 | -3,592635 | 0,000329 | 0,252525 |
| ENSG0000 | -0,002667 | -0,018541 | -3,586975 | 0,000336 | 0,252525 |
| ENSG0000 | -0,002931 | 0,698749 | -3,518735 | 0,000436 | 0,299921 |
| ENSG0000 | 0,002428 | 0,05194 | 3,464492 | 0,000534 | 0,339084 |
| ENSG0000 | 0,002659 | 0,837464 | 3,422012 | 0,000624 | 0,364342 |
| ENSG0000 | -0,002782 | -0,593123 | -3,399612 | 0,000678 | 0,364342 |
| ENSG0000 | 0,00175 | -0,076156 | 3,379601 | 0,000729 | 0,364342 |
| ENSG0000 | -0,001764 | 0,003448 | -3,371802 | 0,00075 | 0,364342 |
| ENSG0000 | -0,003668 | 1,407669 | -3,238855 | 0,001205 | 0,528757 |
| ENSG0000 | 0,00176 | -0,234287 | 3,229763 | 0,001244 | 0,528757 |
| ENSG0000 | 0,002233 | -1,448905 | 3,221347 | 0,001281 | 0,528757 |
| ENSG0000 | -0,003273 | 0,667881 | -3,206821 | 0,001347 | 0,529678 |
| ENSG0000 | -0,001931 | 0,464784 | -3,170408 | 0,001528 | 0,546817 |
| ENSG0000 | -0,002203 | 0,556427 | -3,170381 | 0,001528 | 0,546817 |
| ENSG0000 | -0,003438 | 0,308399 | -3,158879 | 0,001589 | 0,546817 |
| ENSG0000 | 0,004881 | -0,703453 | 3,111416 | 0,001868 | 0,617024 |
| ENSG0000 | 0,002317 | -0,556569 | 3,071183 | 0,002139 | 0,679287 |
| ENSG0000 | 0,002656 | -0,390252 | 3,020206 | 0,002533 | 0,738185 |
| ENSG0000 | 0,00281 | 0,257897 | 3,016206 | 0,002567 | 0,738185 |
| ENSG0000 | -0,000721 | -0,449708 | -3,003533 | 0,002676 | 0,738185 |
| ENSG0000 | 0,001771 | -0,908335 | 2,959523 | 0,003089 | 0,738185 |
| ENSG0000 | -0,001599 | -0,616311 | -2,953965 | 0,003146 | 0,738185 |
| ENSG0000 | -0,002065 | 0,599712 | -2,953774 | 0,003148 | 0,738185 |
| ENSG0000 | -0,001248 | -0,486178 | -2,944906 | 0,003239 | 0,738185 |
| ENSG0000 | 0,004648 | -2,816489 | 2,930684 | 0,003391 | 0,738185 |
| ENSG0000 | -0,001456 | -0,041566 | -2,922665 | 0,003479 | 0,738185 |
| ENSG0000 | -0,003276 | -0,00444 | -2,922178 | 0,003485 | 0,738185 |
| ENSG0000 | 0,001423 | -0,425375 | 2,920404 | 0,003505 | 0,738185 |
| ENSG0000 | -0,002043 | -0,252356 | -2,919583 | 0,003514 | 0,738185 |
| ENSG0000 | 0,002575 | -0,593094 | 2,892009 | 0,003837 | 0,738185 |
| ENSG0000 | 0,002028 | -1,723584 | 2,885363 | 0,003919 | 0,738185 |
| ENSG0000 | 0,003473 | -1,911798 | 2,870737 | 0,004105 | 0,738185 |
| ENSG0000 | -0,000871 | -0,418561 | -2,857337 | 0,004282 | 0,738185 |
| ENSG0000 | 0,001621 | -1,915557 | 2,834684 | 0,004598 | 0,738185 |
| ENSG0000 | 0,00266 | 0,519732 | 2,823886 | 0,004755 | 0,738185 |
| ENSG0000 | -0,001492 | -0,689172 | -2,823156 | 0,004766 | 0,738185 |
| ENSG0000 | 0,000264 | -0,494717 | 2,80007 | 0,00512 | 0,738185 |
| ENSG0000 | -0,001591 | -1,455402 | -2,785989 | 0,005348 | 0,738185 |
| ENSG0000 | 0,00345 | 2,123006 | 2,780363 | 0,005441 | 0,738185 |

| | | | | | |
|---|---|---|---|---|---|
| ENSG0000 | 0,001642 | -1,186027 | 2,778785 | 0,005468 | 0,738185 |
| ENSG0000 | 0,002074 | 0,859409 | 2,777741 | 0,005486 | 0,738185 |
| ENSG0000 | 0,00179 | 0,844617 | 2,775724 | 0,00552 | 0,738185 |
| ENSG0000 | 0,001993 | 1,135641 | 2,774269 | 0,005544 | 0,738185 |
| ENSG0000 | 0,001315 | 0,020873 | 2,768977 | 0,005635 | 0,738185 |
| ENSG0000 | 0,002664 | 1,367883 | 2,76605 | 0,005686 | 0,738185 |
| ENSG0000 | -0,000994 | 0,191161 | -2,762104 | 0,005755 | 0,738185 |
| ENSG0000 | -0,001673 | -0,070643 | -2,745527 | 0,006054 | 0,738185 |
| ENSG0000 | 0,001263 | 0,226904 | 2,73643 | 0,006224 | 0,738185 |
| ENSG0000 | 0,001666 | 0,211027 | 2,735955 | 0,006233 | 0,738185 |
| ENSG0000 | 0,001015 | 0,216574 | 2,733323 | 0,006283 | 0,738185 |
| ENSG0000 | -0,000664 | 0,01527 | -2,73138 | 0,00632 | 0,738185 |
| ENSG0000 | -0,000855 | -0,7433 | -2,72334 | 0,006475 | 0,738185 |
| ENSG0000 | 0,002028 | -1,197159 | 2,721171 | 0,006518 | 0,738185 |
| ENSG0000 | -0,000859 | 0,386881 | -2,719777 | 0,006546 | 0,738185 |
| ENSG0000 | 0,001551 | 0,305529 | 2,713713 | 0,006667 | 0,738185 |
| ENSG0000 | 0,001919 | 0,770355 | 2,710058 | 0,00674 | 0,738185 |
| ENSG0000 | -0,001043 | 0,251891 | -2,708347 | 0,006775 | 0,738185 |
| ENSG0000 | -0,000383 | 0,115335 | -2,707958 | 0,006783 | 0,738185 |
| ENSG0000 | -0,00173 | 0,154775 | -2,696568 | 0,007019 | 0,738185 |
| ENSG0000 | 0,003507 | -1,314767 | 2,695069 | 0,007051 | 0,738185 |
| ENSG0000 | -0,001587 | 1,363728 | -2,683464 | 0,0073 | 0,738185 |
| ENSG0000 | 0,003628 | 0,85808 | 2,682804 | 0,007315 | 0,738185 |
| ENSG0000 | -0,00137 | -1,472128 | -2,680705 | 0,007361 | 0,738185 |
| ENSG0000 | -0,001031 | -0,305243 | -2,678262 | 0,007414 | 0,738185 |
| ENSG0000 | 0,000213 | 0,026368 | 2,672811 | 0,007536 | 0,738185 |
| ENSG0000 | 0,003485 | 0,884812 | 2,670409 | 0,00759 | 0,738185 |
| ENSG0000 | -0,00053 | 0,248644 | -2,670134 | 0,007596 | 0,738185 |
| ENSG0000 | -0,001278 | -0,104702 | -2,666807 | 0,007672 | 0,738185 |
| ENSG0000 | -0,001284 | 0,357207 | -2,664898 | 0,007715 | 0,738185 |
| ENSG0000 | -0,002004 | -0,366862 | -2,662461 | 0,007771 | 0,738185 |
| ENSG0000 | -0,003278 | -0,014854 | -2,660187 | 0,007824 | 0,738185 |
| ENSG0000 | -0,001691 | 0,48306 | -2,656472 | 0,007911 | 0,738185 |
| ENSG0000 | -0,002049 | -0,714638 | -2,653228 | 0,007987 | 0,738185 |
| ENSG0000 | -0,003936 | -0,826128 | -2,65243 | 0,008006 | 0,738185 |
| ENSG0000 | 0,00076 | -0,289426 | 2,651153 | 0,008036 | 0,738185 |
| ENSG0000 | 0,001898 | 2,566335 | 2,649008 | 0,008087 | 0,738185 |
| ENSG0000 | -0,001298 | 0,203478 | -2,644485 | 0,008196 | 0,738185 |
| ENSG0000 | 0,001441 | -0,337213 | 2,644126 | 0,008205 | 0,738185 |
| ENSG0000 | -0,001709 | 0,960026 | -2,642242 | 0,008251 | 0,738185 |
| ENSG0000 | -0,001567 | -0,779504 | -2,64092 | 0,008283 | 0,738185 |
| ENSG0000 | -0,001105 | -0,266433 | -2,632938 | 0,00848 | 0,738185 |
| ENSG0000 | -0,001317 | -0,140673 | -2,627241 | 0,008623 | 0,738185 |
| ENSG0000 | 0,00075 | 0,144024 | 2,626198 | 0,00865 | 0,738185 |
| ENSG0000 | -0,000902 | -0,446227 | -2,626034 | 0,008654 | 0,738185 |
| ENSG0000 | 0,001746 | -0,446545 | 2,624313 | 0,008698 | 0,738185 |
| ENSG0000 | -0,001277 | 0,591514 | -2,61838 | 0,00885 | 0,738185 |
| ENSG0000 | -0,000833 | 0,422883 | -2,617317 | 0,008878 | 0,738185 |

| | | | | | |
|---|---|---|---|---|---|
| ENSG0000 | -0,000991 | 0,118126 | -2,616661 | 0,008895 | 0,738185 |
| ENSG0000 | -0,002177 | 0,715011 | -2,604713 | 0,009211 | 0,738185 |
| ENSG0000 | -0,00083 | -0,022928 | -2,598602 | 0,009376 | 0,738185 |
| ENSG0000 | -0,000786 | -0,241131 | -2,597395 | 0,009409 | 0,738185 |
| ENSG0000 | -0,000906 | -0,179634 | -2,595241 | 0,009468 | 0,738185 |
| ENSG0000 | 0,00161 | -1,272896 | 2,594297 | 0,009494 | 0,738185 |
| ENSG0000 | 0,004778 | -0,169773 | 2,592479 | 0,009545 | 0,738185 |
| ENSG0000 | -0,000986 | -0,810736 | -2,589681 | 0,009623 | 0,738185 |
| ENSG0000 | -0,000698 | 0,033673 | -2,584047 | 0,009781 | 0,738185 |
| ENSG0000 | -0,001121 | 0,860082 | -2,58206 | 0,009837 | 0,738185 |
| ENSG0000 | 0,002822 | 1,840854 | 2,580579 | 0,00988 | 0,738185 |
| ENSG0000 | 0,000523 | 0,231683 | 2,579207 | 0,009919 | 0,738185 |
| ENSG0000 | 0,002169 | 0,492706 | 2,578812 | 0,00993 | 0,738185 |
| ENSG0000 | -0,001714 | -0,518834 | -2,576927 | 0,009985 | 0,738185 |
| ENSG0000 | 0,000438 | -0,011748 | 2,57345 | 0,010085 | 0,738185 |
| ENSG0000 | -0,002571 | 1,220999 | -2,573021 | 0,010098 | 0,738185 |
| ENSG0000 | 0,001712 | -0,969269 | 2,57106 | 0,010155 | 0,738185 |
| ENSG0000 | 0,002021 | -0,191721 | 2,563771 | 0,010371 | 0,738185 |
| ENSG0000 | 0,001713 | -0,605753 | 2,555291 | 0,010627 | 0,738185 |
| ENSG0000 | 0,001933 | -0,402154 | 2,55043 | 0,010776 | 0,738185 |
| ENSG0000 | 0,001136 | 0,036438 | 2,544132 | 0,010972 | 0,738185 |
| ENSG0000 | -0,002602 | 0,562048 | -2,543417 | 0,010995 | 0,738185 |
| ENSG0000 | 0,001601 | -0,725847 | 2,543148 | 0,011003 | 0,738185 |
| ENSG0000 | 0,00166 | 0,214404 | 2,538404 | 0,011153 | 0,738185 |
| ENSG0000 | 0,000886 | -0,047379 | 2,537307 | 0,011188 | 0,738185 |
| ENSG0000 | -0,001216 | 0,573906 | -2,533926 | 0,011297 | 0,738185 |
| ENSG0000 | 0,001505 | 1,874477 | 2,529597 | 0,011437 | 0,738185 |
| ENSG0000 | -0,001714 | 0,443739 | -2,529524 | 0,011439 | 0,738185 |
| ENSG0000 | -0,000406 | 0,253591 | -2,52921 | 0,01145 | 0,738185 |
| ENSG0000 | 0,00084 | -1,226127 | 2,528731 | 0,011465 | 0,738185 |
| ENSG0000 | -0,000999 | -0,575222 | -2,525342 | 0,011576 | 0,738185 |
| ENSG0000 | -0,000798 | -0,297714 | -2,521423 | 0,011706 | 0,738185 |
| ENSG0000 | 0,001447 | -0,37972 | 2,514874 | 0,011925 | 0,738185 |
| ENSG0000 | -0,003464 | -0,986472 | -2,513822 | 0,011961 | 0,738185 |
| ENSG0000 | -0,001181 | 0,535167 | -2,510056 | 0,012089 | 0,738185 |
| ENSG0000 | 0,000496 | 0,045311 | 2,509045 | 0,012124 | 0,738185 |
| ENSG0000 | -0,000844 | 0,075129 | -2,506492 | 0,012212 | 0,738185 |
| ENSG0000 | 0,001196 | -0,080318 | 2,499864 | 0,012442 | 0,738185 |
| ENSG0000 | 0,001165 | -1,739445 | 2,496066 | 0,012576 | 0,738185 |
| ENSG0000 | 0,001447 | -0,731671 | 2,495184 | 0,012608 | 0,738185 |
| ENSG0000 | -0,002503 | 1,92892 | -2,49057 | 0,012772 | 0,738185 |
| ENSG0000 | 0,00209 | -0,744204 | 2,490458 | 0,012776 | 0,738185 |
| ENSG0000 | -0,001635 | 1,037342 | -2,490414 | 0,012778 | 0,738185 |
| ENSG0000 | -0,002155 | 1,67321 | -2,482785 | 0,013055 | 0,738185 |
| ENSG0000 | -0,001501 | -1,028441 | -2,480523 | 0,013138 | 0,738185 |
| ENSG0000 | 0,001424 | 1,12101 | 2,473628 | 0,013394 | 0,738185 |
| ENSG0000 | 0,000741 | -0,039795 | 2,469632 | 0,013544 | 0,738185 |
| ENSG0000 | -0,001801 | 0,457632 | -2,469228 | 0,01356 | 0,738185 |

| | | | | | |
|---|---|---|---|---|---|
| ENSG0000 | -0,001927 | -1,315805 | -2,465785 | 0,013691 | 0,738185 |
| ENSG0000 | 0,001738 | 0,366453 | 2,464529 | 0,013739 | 0,738185 |
| ENSG0000 | 0,000846 | -0,387088 | 2,462968 | 0,013798 | 0,738185 |
| ENSG0000 | 0,002412 | 1,350611 | 2,46172 | 0,013847 | 0,738185 |
| ENSG0000 | -0,002267 | 0,325824 | -2,460483 | 0,013894 | 0,738185 |
| ENSG0000 | -0,002106 | 0,738943 | -2,457748 | 0,014001 | 0,738185 |
| ENSG0000 | 0,001269 | -0,208947 | 2,456166 | 0,014062 | 0,738185 |
| ENSG0000 | -0,001661 | 0,985063 | -2,453554 | 0,014165 | 0,738185 |
| ENSG0000 | 0,001527 | -0,066907 | 2,448542 | 0,014363 | 0,738185 |
| ENSG0000 | 0,000505 | 0,019387 | 2,448491 | 0,014365 | 0,738185 |
| ENSG0000 | -0,001558 | -0,496757 | -2,442741 | 0,014596 | 0,738185 |
| ENSG0000 | 0,002421 | 0,379438 | 2,440797 | 0,014675 | 0,738185 |
| ENSG0000 | 0,001183 | -1,171687 | 2,437874 | 0,014794 | 0,738185 |
| ENSG0000 | -0,001669 | 0,049522 | -2,436772 | 0,014839 | 0,738185 |
| ENSG0000 | 0,00033 | -0,220725 | 2,436025 | 0,01487 | 0,738185 |
| ENSG0000 | -0,003619 | 0,485638 | -2,432616 | 0,01501 | 0,738185 |
| ENSG0000 | -0,001224 | -0,627342 | -2,432363 | 0,015021 | 0,738185 |
| ENSG0000 | 0,000743 | -0,745644 | 2,432203 | 0,015027 | 0,738185 |
| ENSG0000 | -0,00111 | -0,147844 | -2,424208 | 0,015362 | 0,738185 |
| ENSG0000 | -0,003628 | 0,678838 | -2,422153 | 0,015449 | 0,738185 |
| ENSG0000 | 0,001313 | 0,316809 | 2,420527 | 0,015518 | 0,738185 |
| ENSG0000 | 0,001533 | -0,251459 | 2,418896 | 0,015588 | 0,738185 |
| ENSG0000 | 0,000919 | 0,579884 | 2,418647 | 0,015599 | 0,738185 |
| ENSG0000 | -0,001086 | -0,000312 | -2,417917 | 0,01563 | 0,738185 |
| ENSG0000 | -0,002255 | 0,298504 | -2,417636 | 0,015642 | 0,738185 |
| ENSG0000 | 0,000726 | 0,0496 | 2,416769 | 0,015679 | 0,738185 |
| ENSG0000 | -0,001883 | 0,405702 | -2,416133 | 0,015707 | 0,738185 |
| ENSG0000 | -0,00071 | 0,30455 | -2,41262 | 0,015859 | 0,738185 |
| ENSG0000 | 0,002489 | 1,063117 | 2,4121 | 0,015881 | 0,738185 |
| ENSG0000 | -0,002198 | 2,230042 | -2,411154 | 0,015923 | 0,738185 |
| ENSG0000 | -0,004087 | 2,267851 | -2,409413 | 0,015999 | 0,738185 |
| ENSG0000 | -0,000742 | -0,456656 | -2,408751 | 0,016028 | 0,738185 |
| ENSG0000 | -0,000541 | 0,379029 | -2,407248 | 0,016094 | 0,738185 |
| ENSG0000 | -0,001862 | 0,67543 | -2,406476 | 0,016128 | 0,738185 |
| ENSG0000 | -0,002365 | 0,579576 | -2,405534 | 0,01617 | 0,738185 |
| ENSG0000 | 0,00105 | 0,217407 | 2,404766 | 0,016204 | 0,738185 |
| ENSG0000 | -0,000688 | -0,048058 | -2,403879 | 0,016243 | 0,738185 |
| ENSG0000 | -0,000917 | -0,38497 | -2,401914 | 0,01633 | 0,738185 |
| ENSG0000 | -0,00365 | -1,046557 | -2,401286 | 0,016358 | 0,738185 |
| ENSG0000 | -0,000388 | -0,115348 | -2,393349 | 0,016716 | 0,75024 |
| ENSG0000 | -0,001497 | -0,428362 | -2,389876 | 0,016875 | 0,752793 |
| ENSG0000 | -0,000834 | -0,24928 | -2,387148 | 0,017001 | 0,752793 |
| ENSG0000 | 0,001332 | -1,184408 | 2,383575 | 0,017167 | 0,752793 |
| ENSG0000 | -0,000648 | 0,330443 | -2,380909 | 0,017291 | 0,752793 |
| ENSG0000 | -0,000962 | 0,516727 | -2,378756 | 0,017393 | 0,752793 |
| ENSG0000 | -0,001468 | -0,536569 | -2,374482 | 0,017595 | 0,752793 |
| ENSG0000 | 0,001358 | 0,801703 | 2,371693 | 0,017728 | 0,752793 |
| ENSG0000 | -0,001391 | 0,326359 | -2,370453 | 0,017788 | 0,752793 |

| | | | | | |
|---|---|---|---|---|---|
| ENSG0000 | 0,003181 | 0,321117 | 2,365613 | 0,018022 | 0,752793 |
| ENSG0000 | -0,0027 | 0,076709 | -2,362773 | 0,018161 | 0,752793 |
| ENSG0000 | 0,001552 | -1,092681 | 2,361898 | 0,018204 | 0,752793 |
| ENSG0000 | 0,000548 | -0,43369 | 2,360078 | 0,018293 | 0,752793 |
| ENSG0000 | 0,004139 | 1,079064 | 2,356883 | 0,018451 | 0,752793 |
| ENSG0000 | -0,000749 | 0,340335 | -2,356563 | 0,018467 | 0,752793 |
| ENSG0000 | 0,000779 | -1,216962 | 2,356134 | 0,018488 | 0,752793 |
| ENSG0000 | -0,000678 | -0,036721 | -2,35563 | 0,018513 | 0,752793 |
| ENSG0000 | -0,000874 | -0,290758 | -2,355414 | 0,018524 | 0,752793 |
| ENSG0000 | -0,000979 | 1,900479 | -2,354446 | 0,018572 | 0,752793 |
| ENSG0000 | 0,001777 | -1,129885 | 2,35391 | 0,018599 | 0,752793 |
| ENSG0000 | 0,001192 | -0,108817 | 2,349125 | 0,01884 | 0,752793 |
| ENSG0000 | 0,001968 | 0,757879 | 2,347217 | 0,018937 | 0,752793 |
| ENSG0000 | 0,000507 | 0,001162 | 2,347036 | 0,018946 | 0,752793 |
| ENSG0000 | -0,001487 | 0,037563 | -2,345377 | 0,01903 | 0,752793 |
| ENSG0000 | -0,000486 | 0,025889 | -2,343392 | 0,019132 | 0,752793 |
| ENSG0000 | 0,000682 | 0,203362 | 2,341363 | 0,019236 | 0,752793 |
| ENSG0000 | 0,003066 | 1,200214 | 2,340271 | 0,019292 | 0,752793 |
| ENSG0000 | 0,000418 | -0,211201 | 2,340155 | 0,019298 | 0,752793 |
| ENSG0000 | -0,001028 | 0,63767 | -2,336464 | 0,01949 | 0,752793 |
| ENSG0000 | -0,003355 | -0,88698 | -2,334171 | 0,019609 | 0,752793 |
| ENSG0000 | 0,001107 | 0,572802 | 2,333708 | 0,019634 | 0,752793 |
| ENSG0000 | -0,003103 | 1,336297 | -2,331358 | 0,019757 | 0,752793 |
| ENSG0000 | 0,000996 | -0,208492 | 2,330299 | 0,019813 | 0,752793 |
| ENSG0000 | -0,001336 | -0,520862 | -2,326735 | 0,020002 | 0,752793 |
| ENSG0000 | 0,001202 | 0,317546 | 2,325028 | 0,020093 | 0,752793 |
| ENSG0000 | -0,001408 | 0,51634 | -2,324432 | 0,020125 | 0,752793 |
| ENSG0000 | 0,000698 | 0,062077 | 2,32378 | 0,02016 | 0,752793 |
| ENSG0000 | 0,001155 | -1,523709 | 2,323246 | 0,020189 | 0,752793 |
| ENSG0000 | -0,001143 | 0,058785 | -2,320751 | 0,020323 | 0,752793 |
| ENSG0000 | 0,000752 | 0,097387 | 2,314582 | 0,020659 | 0,752793 |
| ENSG0000 | 0,001298 | -0,356806 | 2,31337 | 0,020726 | 0,752793 |
| ENSG0000 | -0,000915 | 0,615806 | -2,313116 | 0,020739 | 0,752793 |
| ENSG0000 | -0,000916 | -0,548105 | -2,312327 | 0,020783 | 0,752793 |
| ENSG0000 | -0,001136 | 0,641068 | -2,312243 | 0,020788 | 0,752793 |
| ENSG0000 | -0,001049 | -0,956451 | -2,311043 | 0,020854 | 0,752793 |
| ENSG0000 | -0,000957 | -0,320264 | -2,309345 | 0,020948 | 0,752793 |
| ENSG0000 | 0,003505 | -0,479112 | 2,306792 | 0,02109 | 0,752793 |
| ENSG0000 | 0,002533 | 2,177248 | 2,304473 | 0,02122 | 0,752793 |
| ENSG0000 | 0,00088 | 0,492843 | 2,304349 | 0,021227 | 0,752793 |
| ENSG0000 | 0,001432 | 0,77379 | 2,302341 | 0,021339 | 0,752793 |
| ENSG0000 | -0,000974 | -0,507634 | -2,301785 | 0,021371 | 0,752793 |
| ENSG0000 | 0,002173 | -0,341284 | 2,297624 | 0,021607 | 0,752793 |
| ENSG0000 | -0,00061 | 0,712536 | -2,297245 | 0,021628 | 0,752793 |
| ENSG0000 | 0,001412 | -0,747163 | 2,294482 | 0,021787 | 0,752793 |
| ENSG0000 | -0,000475 | 0,113617 | -2,292648 | 0,021892 | 0,752793 |
| ENSG0000 | -0,001125 | 0,445507 | -2,29032 | 0,022027 | 0,752793 |
| ENSG0000 | 0,000744 | -0,992205 | 2,29032 | 0,022027 | 0,752793 |

| | | | | | |
|---|---|---|---|---|---|
| ENSG0000 | 0,002387 | 0,78768 | 2,29031 | 0,022027 | 0,752793 |
| ENSG0000 | -0,001548 | 0,589404 | -2,288881 | 0,02211 | 0,752793 |
| ENSG0000 | 0,00051 | 0,060113 | 2,288167 | 0,022152 | 0,752793 |
| ENSG0000 | 0,001845 | -0,055972 | 2,273482 | 0,023021 | 0,763048 |
| ENSG0000 | 0,000587 | 0,027353 | 2,273211 | 0,023038 | 0,763048 |
| ENSG0000 | -0,001454 | -0,066575 | -2,270241 | 0,023217 | 0,763048 |
| ENSG0000 | -0,001277 | 0,930389 | -2,268918 | 0,023298 | 0,763048 |
| ENSG0000 | 0,00203 | -1,319866 | 2,268504 | 0,023323 | 0,763048 |
| ENSG0000 | -0,002312 | 1,798401 | -2,266128 | 0,023468 | 0,763048 |
| ENSG0000 | -0,00188 | 0,104995 | -2,265975 | 0,023477 | 0,763048 |
| ENSG0000 | -0,001151 | 0,575716 | -2,265362 | 0,023515 | 0,763048 |
| ENSG0000 | -0,00227 | -0,352106 | -2,263924 | 0,023603 | 0,763048 |
| ENSG0000 | 0,000919 | 0,826839 | 2,263771 | 0,023613 | 0,763048 |
| ENSG0000 | 0,001563 | -0,52136 | 2,263254 | 0,023645 | 0,763048 |
| ENSG0000 | 0,001436 | -0,257435 | 2,262762 | 0,023675 | 0,763048 |
| ENSG0000 | -0,002257 | -1,495407 | -2,261795 | 0,023735 | 0,763048 |
| ENSG0000 | 0,001094 | -0,261288 | 2,26031 | 0,023827 | 0,763048 |
| ENSG0000 | -0,001229 | 0,900408 | -2,260103 | 0,023839 | 0,763048 |
| ENSG0000 | 0,002516 | 0,888586 | 2,247918 | 0,024606 | 0,784554 |
| ENSG0000 | 0,000572 | 0,161123 | 2,241947 | 0,02499 | 0,789641 |
| ENSG0000 | -0,001384 | -0,240209 | -2,24152 | 0,025018 | 0,789641 |
| ENSG0000 | -0,001494 | 0,730923 | -2,238978 | 0,025183 | 0,789641 |
| ENSG0000 | -0,001064 | -0,526794 | -2,23847 | 0,025216 | 0,789641 |
| ENSG0000 | -0,000232 | 0,116996 | -2,235159 | 0,025432 | 0,789641 |
| ENSG0000 | 0,001511 | -1,458218 | 2,234749 | 0,025459 | 0,789641 |
| ENSG0000 | -0,001819 | 0,685816 | -2,231296 | 0,025687 | 0,789641 |
| ENSG0000 | -0,000596 | 0,583786 | -2,226389 | 0,026014 | 0,789641 |
| ENSG0000 | -0,003695 | -1,771735 | -2,225422 | 0,026078 | 0,789641 |
| ENSG0000 | 0,00139 | 0,917419 | 2,224626 | 0,026132 | 0,789641 |
| ENSG0000 | -0,001403 | 0,049915 | -2,223901 | 0,026181 | 0,789641 |
| ENSG0000 | 0,001812 | -2,251471 | 2,221555 | 0,026339 | 0,789641 |
| ENSG0000 | 0,001462 | 0,009516 | 2,219787 | 0,026459 | 0,789641 |
| ENSG0000 | 0,001494 | 0,660702 | 2,219557 | 0,026475 | 0,789641 |
| ENSG0000 | -0,0011 | -0,249562 | -2,218885 | 0,02652 | 0,789641 |
| ENSG0000 | -0,001498 | 0,617129 | -2,21818 | 0,026568 | 0,789641 |
| ENSG0000 | 0,000673 | -0,214911 | 2,216067 | 0,026713 | 0,789641 |
| ENSG0000 | -0,001136 | 0,036498 | -2,215841 | 0,026728 | 0,789641 |
| ENSG0000 | -0,001338 | 0,644357 | -2,213976 | 0,026856 | 0,789641 |
| ENSG0000 | 0,000509 | -0,184844 | 2,21326 | 0,026906 | 0,789641 |
| ENSG0000 | 0,001701 | -0,768357 | 2,211632 | 0,027018 | 0,789641 |
| ENSG0000 | 0,00103 | -0,367833 | 2,209992 | 0,027132 | 0,789641 |
| ENSG0000 | 0,001421 | 1,426627 | 2,209895 | 0,027138 | 0,789641 |
| ENSG0000 | 0,001386 | -0,812498 | 2,209204 | 0,027186 | 0,789641 |
| ENSG0000 | -0,001758 | 0,922768 | -2,208473 | 0,027237 | 0,789641 |
| ENSG0000 | 0,001136 | -1,368065 | 2,208261 | 0,027252 | 0,789641 |
| ENSG0000 | 0,00111 | -0,641763 | 2,206693 | 0,027362 | 0,79004 |
| ENSG0000 | -0,001945 | 0,60792 | -2,200938 | 0,027767 | 0,792549 |
| ENSG0000 | 0,002428 | 2,018457 | 2,200186 | 0,02782 | 0,792549 |

| | | | | | |
|---|---|---|---|---|---|
| ENSG0000 | 0,000811 | -0,178347 | 2,200136 | 0,027823 | 0,792549 |
| ENSG0000 | -0,000966 | 1,00897 | -2,200011 | 0,027832 | 0,792549 |
| ENSG0000 | 0,000806 | 0,252841 | 2,193404 | 0,028305 | 0,79708 |
| ENSG0000 | 0,00397 | 0,179775 | 2,189873 | 0,02856 | 0,79708 |
| ENSG0000 | 0,001524 | -0,715689 | 2,189832 | 0,028563 | 0,79708 |
| ENSG0000 | -0,001087 | 0,514889 | -2,189488 | 0,028588 | 0,79708 |
| ENSG0000 | 0,00205 | 0,594712 | 2,186975 | 0,028771 | 0,79708 |
| ENSG0000 | 0,000305 | 0,036783 | 2,186052 | 0,028838 | 0,79708 |
| ENSG0000 | 0,000998 | -0,647128 | 2,183571 | 0,02902 | 0,79708 |
| ENSG0000 | -0,001105 | 0,276268 | -2,183207 | 0,029047 | 0,79708 |
| ENSG0000 | 0,000491 | -0,672608 | 2,182035 | 0,029134 | 0,79708 |
| ENSG0000 | 0,000918 | -0,626298 | 2,179332 | 0,029334 | 0,79708 |
| ENSG0000 | -0,000866 | 0,172304 | -2,178956 | 0,029362 | 0,79708 |
| ENSG0000 | 0,001136 | -0,599724 | 2,176102 | 0,029574 | 0,79708 |
| ENSG0000 | 0,001394 | -0,47116 | 2,175601 | 0,029612 | 0,79708 |
| ENSG0000 | 0,001286 | 1,561587 | 2,174745 | 0,029676 | 0,79708 |
| ENSG0000 | -0,001028 | 1,03521 | -2,173687 | 0,029756 | 0,79708 |
| ENSG0000 | 0,002268 | 0,487754 | 2,17205 | 0,029879 | 0,79708 |
| ENSG0000 | 0,000881 | 0,68226 | 2,169476 | 0,030074 | 0,79708 |
| ENSG0000 | -0,001512 | 0,378121 | -2,166178 | 0,030325 | 0,79708 |
| ENSG0000 | -0,000735 | -0,424461 | -2,165798 | 0,030354 | 0,79708 |
| ENSG0000 | -0,000719 | -0,836029 | -2,165713 | 0,03036 | 0,79708 |
| ENSG0000 | 0,002025 | 0,875457 | 2,164881 | 0,030424 | 0,79708 |
| ENSG0000 | 0,000521 | 0,064165 | 2,164685 | 0,030439 | 0,79708 |
| ENSG0000 | -0,001303 | 1,603546 | -2,164135 | 0,030481 | 0,79708 |
| ENSG0000 | -0,000951 | 0,555913 | -2,164039 | 0,030489 | 0,79708 |
| ENSG0000 | -0,000901 | -0,457227 | -2,160843 | 0,030735 | 0,79708 |
| ENSG0000 | -0,002117 | 1,053456 | -2,160738 | 0,030743 | 0,79708 |
| ENSG0000 | -0,000632 | 0,08939 | -2,159148 | 0,030866 | 0,79708 |
| ENSG0000 | -0,001175 | -0,242116 | -2,158935 | 0,030882 | 0,79708 |
| ENSG0000 | -0,001887 | 1,881836 | -2,158681 | 0,030902 | 0,79708 |
| ENSG0000 | -0,001501 | -0,21648 | -2,158527 | 0,030914 | 0,79708 |
| ENSG0000 | -0,000392 | -0,323824 | -2,157634 | 0,030984 | 0,79708 |
| ENSG0000 | 0,000505 | -0,565757 | 2,15631 | 0,031087 | 0,797251 |
| ENSG0000 | -0,001771 | -0,325559 | -2,147945 | 0,031746 | 0,809696 |
| ENSG0000 | -0,001225 | -0,837434 | -2,147662 | 0,031768 | 0,809696 |
| ENSG0000 | 0,00289 | 4,483369 | 2,145797 | 0,031917 | 0,810271 |
| ENSG0000 | -0,000563 | 0,468037 | -2,14417 | 0,032047 | 0,810271 |
| ENSG0000 | 0,001256 | 0,231145 | 2,143694 | 0,032085 | 0,810271 |
| ENSG0000 | -0,000768 | 0,090759 | -2,142402 | 0,032189 | 0,810412 |
| ENSG0000 | -0,000589 | -0,282565 | -2,139608 | 0,032414 | 0,811149 |
| ENSG0000 | 0,000747 | -0,062294 | 2,138325 | 0,032518 | 0,811149 |
| ENSG0000 | 0,000832 | -0,255941 | 2,137401 | 0,032593 | 0,811149 |
| ENSG0000 | 0,000965 | -0,345839 | 2,137183 | 0,032611 | 0,811149 |
| ENSG0000 | 0,001018 | 0,400302 | 2,129009 | 0,033282 | 0,822087 |
| ENSG0000 | 0,000838 | -0,313894 | 2,128806 | 0,033298 | 0,822087 |
| ENSG0000 | 0,002269 | 1,330551 | 2,127658 | 0,033394 | 0,822087 |
| ENSG0000 | -0,001272 | -0,208203 | -2,126791 | 0,033465 | 0,822087 |

| | | | | | |
|---|---|---|---|---|---|
| ENSG0000 | 0,000707 | 0,609633 | 2,125795 | 0,033548 | 0,822087 |
| ENSG0000 | 0,002583 | 1,001896 | 2,122999 | 0,033782 | 0,824815 |
| ENSG0000 | -0,001199 | -0,469432 | -2,121073 | 0,033944 | 0,824815 |
| ENSG0000 | 0,000902 | 1,076061 | 2,119928 | 0,03404 | 0,824815 |
| ENSG0000 | -0,00151 | -0,552538 | -2,119704 | 0,034059 | 0,824815 |
| ENSG0000 | 0,001074 | 0,398577 | 2,117949 | 0,034208 | 0,825986 |
| ENSG0000 | 0,001719 | -0,363481 | 2,114499 | 0,034501 | 0,829088 |
| ENSG0000 | 0,001408 | 0,892074 | 2,113734 | 0,034566 | 0,829088 |
| ENSG0000 | -0,000961 | -0,527789 | -2,112023 | 0,034713 | 0,829088 |
| ENSG0000 | 0,001182 | -0,349245 | 2,11068 | 0,034828 | 0,829088 |
| ENSG0000 | -0,000886 | -0,388035 | -2,106825 | 0,035161 | 0,829088 |
| ENSG0000 | -0,001171 | -1,311275 | -2,099237 | 0,035825 | 0,829088 |
| ENSG0000 | -0,002247 | 1,042525 | -2,095247 | 0,036178 | 0,829088 |
| ENSG0000 | -0,001426 | -0,313842 | -2,094038 | 0,036286 | 0,829088 |
| ENSG0000 | 0,002661 | -0,256256 | 2,093754 | 0,036311 | 0,829088 |
| ENSG0000 | -0,002624 | -0,302683 | -2,093258 | 0,036355 | 0,829088 |
| ENSG0000 | -0,002567 | 0,941933 | -2,091272 | 0,036533 | 0,829088 |
| ENSG0000 | -0,001207 | 0,473791 | -2,090171 | 0,036631 | 0,829088 |
| ENSG0000 | -0,000863 | 0,56636 | -2,089037 | 0,036733 | 0,829088 |
| ENSG0000 | 0,000856 | -0,984899 | 2,088116 | 0,036816 | 0,829088 |
| ENSG0000 | 0,00045 | 0,141575 | 2,086965 | 0,03692 | 0,829088 |
| ENSG0000 | 0,001249 | -0,161624 | 2,08605 | 0,037003 | 0,829088 |
| ENSG0000 | -0,000583 | -0,3851 | -2,08576 | 0,037029 | 0,829088 |
| ENSG0000 | 0,000767 | 0,194129 | 2,085619 | 0,037042 | 0,829088 |
| ENSG0000 | -0,001245 | -0,676221 | -2,084573 | 0,037137 | 0,829088 |
| ENSG0000 | 0,000492 | 0,052151 | 2,083834 | 0,037204 | 0,829088 |
| ENSG0000 | -0,000485 | -0,071784 | -2,083552 | 0,03723 | 0,829088 |
| ENSG0000 | 0,000886 | -1,11134 | 2,08323 | 0,037259 | 0,829088 |
| ENSG0000 | 0,001277 | -0,638978 | 2,083062 | 0,037275 | 0,829088 |
| ENSG0000 | 0,0012 | 0,479529 | 2,080758 | 0,037485 | 0,829088 |
| ENSG0000 | -0,002117 | 1,483355 | -2,080749 | 0,037486 | 0,829088 |
| ENSG0000 | -0,000717 | -0,15258 | -2,080423 | 0,037516 | 0,829088 |
| ENSG0000 | 0,000939 | -0,035804 | 2,080312 | 0,037526 | 0,829088 |
| ENSG0000 | -0,002321 | -0,042363 | -2,079877 | 0,037566 | 0,829088 |
| ENSG0000 | -0,001083 | -0,458124 | -2,079455 | 0,037605 | 0,829088 |
| ENSG0000 | -0,001358 | 1,031395 | -2,078437 | 0,037698 | 0,829088 |
| ENSG0000 | 0,001171 | -0,427321 | 2,077349 | 0,037799 | 0,829088 |
| ENSG0000 | 0,002627 | 1,628401 | 2,077193 | 0,037813 | 0,829088 |
| ENSG0000 | 0,000535 | 0,088951 | 2,076024 | 0,037921 | 0,829088 |
| ENSG0000 | -0,001465 | 0,257763 | -2,075629 | 0,037958 | 0,829088 |
| ENSG0000 | -0,001148 | 0,270944 | -2,074633 | 0,03805 | 0,829088 |
| ENSG0000 | 0,001303 | 1,125719 | 2,07288 | 0,038213 | 0,829088 |
| ENSG0000 | -0,00045 | -0,119661 | -2,07011 | 0,038471 | 0,829088 |
| ENSG0000 | 0,00113 | -0,702189 | 2,069131 | 0,038563 | 0,829088 |
| ENSG0000 | 0,001116 | -0,635241 | 2,068689 | 0,038605 | 0,829088 |
| ENSG0000 | 0,000734 | -0,427542 | 2,068162 | 0,038654 | 0,829088 |
| ENSG0000 | -0,000409 | -0,058416 | -2,06594 | 0,038864 | 0,829088 |
| ENSG0000 | -0,002922 | 0,324447 | -2,065372 | 0,038917 | 0,829088 |

| | | | | | |
|---|---|---|---|---|---|
| ENSG0000 | -0,000441 | 0,073426 | -2,061622 | 0,039273 | 0,829088 |
| ENSG0000 | -0,000546 | -0,368801 | -2,060207 | 0,039408 | 0,829088 |
| ENSG0000 | -0,000335 | -0,199865 | -2,059831 | 0,039444 | 0,829088 |
| ENSG0000 | -0,001834 | 0,635918 | -2,059079 | 0,039516 | 0,829088 |
| ENSG0000 | -0,001695 | 1,179638 | -2,055895 | 0,039822 | 0,829088 |
| ENSG0000 | -0,00075 | 0,237357 | -2,05583 | 0,039829 | 0,829088 |
| ENSG0000 | 0,000738 | 0,216813 | 2,053467 | 0,040057 | 0,829088 |
| ENSG0000 | 0,001441 | -0,104474 | 2,053137 | 0,040089 | 0,829088 |
| ENSG0000 | 0,000562 | 0,36548 | 2,051658 | 0,040233 | 0,829088 |
| ENSG0000 | -0,001332 | -0,831361 | -2,051008 | 0,040296 | 0,829088 |
| ENSG0000 | 0,001927 | 0,133995 | 2,050668 | 0,040329 | 0,829088 |
| ENSG0000 | 0,000528 | 0,206177 | 2,049901 | 0,040404 | 0,829088 |
| ENSG0000 | 0,002004 | -0,932024 | 2,048991 | 0,040493 | 0,829088 |
| ENSG0000 | 0,002076 | 0,861495 | 2,047049 | 0,040683 | 0,829088 |
| ENSG0000 | 0,001608 | -1,315502 | 2,045882 | 0,040798 | 0,829088 |
| ENSG0000 | 0,001033 | 0,26253 | 2,044252 | 0,040959 | 0,829088 |
| ENSG0000 | -0,001783 | -0,108269 | -2,041776 | 0,041204 | 0,829088 |
| ENSG0000 | 0,000626 | -0,410157 | 2,041696 | 0,041212 | 0,829088 |
| ENSG0000 | -0,001623 | 1,806437 | -2,041201 | 0,041261 | 0,829088 |
| ENSG0000 | -0,000597 | -0,466096 | -2,04057 | 0,041324 | 0,829088 |
| ENSG0000 | -0,001182 | 0,642914 | -2,039722 | 0,041408 | 0,829088 |
| ENSG0000 | 0,001829 | 0,29238 | 2,036707 | 0,04171 | 0,829088 |
| ENSG0000 | 0,002107 | 0,070513 | 2,03278 | 0,042105 | 0,829088 |
| ENSG0000 | 0,001013 | -0,436971 | 2,03182 | 0,042202 | 0,829088 |
| ENSG0000 | 0,001057 | -0,329335 | 2,031123 | 0,042273 | 0,829088 |
| ENSG0000 | -0,00063 | 0,19374 | -2,029648 | 0,042423 | 0,829088 |
| ENSG0000 | 0,00085 | 0,19016 | 2,02829 | 0,042561 | 0,829088 |
| ENSG0000 | -0,000923 | 0,161005 | -2,028062 | 0,042584 | 0,829088 |
| ENSG0000 | -0,001384 | 0,410685 | -2,026999 | 0,042693 | 0,829088 |
| ENSG0000 | -0,000758 | -0,112617 | -2,026714 | 0,042722 | 0,829088 |
| ENSG0000 | -0,000468 | 1,35644 | -2,026658 | 0,042728 | 0,829088 |
| ENSG0000 | -0,000675 | -0,220472 | -2,026488 | 0,042745 | 0,829088 |
| ENSG0000 | 0,002189 | 0,48377 | 2,026416 | 0,042753 | 0,829088 |
| ENSG0000 | 0,003272 | -0,277818 | 2,025852 | 0,04281 | 0,829088 |
| ENSG0000 | 0,001481 | 1,064337 | 2,025394 | 0,042857 | 0,829088 |
| ENSG0000 | 0,002748 | 0,403752 | 2,024976 | 0,0429 | 0,829088 |
| ENSG0000 | 0,001025 | 0,128276 | 2,024222 | 0,042978 | 0,829088 |
| ENSG0000 | -0,002076 | 0,151887 | -2,023375 | 0,043065 | 0,829088 |
| ENSG0000 | -0,001383 | 0,586427 | -2,022442 | 0,043161 | 0,829088 |
| ENSG0000 | -0,000762 | 0,598473 | -2,022167 | 0,04319 | 0,829088 |
| ENSG0000 | 0,001583 | 0,034502 | 2,021295 | 0,04328 | 0,829088 |
| ENSG0000 | -0,001023 | 1,414166 | -2,019979 | 0,043416 | 0,829088 |
| ENSG0000 | 0,00043 | 0,146873 | 2,019699 | 0,043445 | 0,829088 |
| ENSG0000 | 0,000521 | 0,201539 | 2,017899 | 0,043632 | 0,829088 |
| ENSG0000 | 0,000634 | -0,129238 | 2,017175 | 0,043708 | 0,829088 |
| ENSG0000 | -0,000739 | 0,3113 | -2,016594 | 0,043769 | 0,829088 |
| ENSG0000 | 0,001572 | -0,42205 | 2,016513 | 0,043777 | 0,829088 |
| ENSG0000 | -0,002099 | -0,899232 | -2,013317 | 0,044112 | 0,829088 |

| | | | | | |
|---|---|---|---|---|---|
| ENSG0000 | -0,000941 | 0,819259 | -2,01122 | 0,044333 | 0,829088 |
| ENSG0000 | 0,000757 | -0,094748 | 2,010619 | 0,044397 | 0,829088 |
| ENSG0000 | -0,000287 | -0,11479 | -2,010505 | 0,044409 | 0,829088 |
| ENSG0000 | -0,003131 | 0,209778 | -2,006976 | 0,044783 | 0,829088 |
| ENSG0000 | -0,001292 | -0,014968 | -2,006424 | 0,044842 | 0,829088 |
| ENSG0000 | -0,001935 | -0,671991 | -2,00573 | 0,044916 | 0,829088 |
| ENSG0000 | -0,00086 | 0,249873 | -2,005091 | 0,044984 | 0,829088 |
| ENSG0000 | 0,00145 | -0,991189 | 2,003203 | 0,045186 | 0,829088 |
| ENSG0000 | 0,001396 | 1,135303 | 2,002134 | 0,045301 | 0,829088 |
| ENSG0000 | 0,000902 | -0,323089 | 2,001024 | 0,045421 | 0,829088 |
| ENSG0000 | 0,001067 | 0,499857 | 1,999761 | 0,045557 | 0,829088 |
| ENSG0000 | -0,000953 | -1,424609 | -1,999406 | 0,045596 | 0,829088 |
| ENSG0000 | -0,000779 | -0,172571 | -1,999107 | 0,045628 | 0,829088 |
| ENSG0000 | 0,000474 | 0,465712 | 1,998452 | 0,045699 | 0,829088 |
| ENSG0000 | 0,00266 | -0,535392 | 1,997326 | 0,045821 | 0,829088 |
| ENSG0000 | -0,00113 | 0,962984 | -1,995274 | 0,046044 | 0,829088 |
| ENSG0000 | 0,00246 | -1,190134 | 1,995033 | 0,04607 | 0,829088 |
| ENSG0000 | 0,000908 | -0,344674 | 1,993431 | 0,046245 | 0,829088 |
| ENSG0000 | -0,000889 | -0,317489 | -1,993277 | 0,046262 | 0,829088 |
| ENSG0000 | -0,000426 | 0,269336 | -1,992145 | 0,046386 | 0,829088 |
| ENSG0000 | 0,001116 | -1,511084 | 1,991763 | 0,046428 | 0,829088 |
| ENSG0000 | 0,001045 | -0,024353 | 1,987594 | 0,046888 | 0,829088 |
| ENSG0000 | 0,000972 | 1,182848 | 1,98639 | 0,047021 | 0,829088 |
| ENSG0000 | -0,000895 | -0,46748 | -1,985701 | 0,047098 | 0,829088 |
| ENSG0000 | 0,001677 | -0,590403 | 1,985691 | 0,047099 | 0,829088 |
| ENSG0000 | -0,001611 | 0,372549 | -1,98508 | 0,047167 | 0,829088 |
| ENSG0000 | -0,000909 | 0,253825 | -1,983035 | 0,047395 | 0,829088 |
| ENSG0000 | -0,001199 | 1,380566 | -1,980531 | 0,047675 | 0,829088 |
| ENSG0000 | -0,001326 | 1,178415 | -1,980089 | 0,047725 | 0,829088 |
| ENSG0000 | -0,001888 | 0,470945 | -1,979996 | 0,047735 | 0,829088 |
| ENSG0000 | -0,000873 | 0,295335 | -1,979475 | 0,047794 | 0,829088 |
| ENSG0000 | -0,000426 | -0,194059 | -1,977926 | 0,047969 | 0,829088 |
| ENSG0000 | 0,000515 | -0,187652 | 1,977924 | 0,047969 | 0,829088 |
| ENSG0000 | -0,00072 | -0,099047 | -1,977113 | 0,04806 | 0,829088 |
| ENSG0000 | 0,003437 | -1,942596 | 1,976 | 0,048186 | 0,829088 |
| ENSG0000 | 0,00144 | -0,347511 | 1,974712 | 0,048332 | 0,829088 |
| ENSG0000 | 0,001081 | 0,441725 | 1,972888 | 0,04854 | 0,829088 |
| ENSG0000 | 0,00154 | -1,491247 | 1,97219 | 0,04862 | 0,829088 |
| ENSG0000 | -0,002998 | 1,341638 | -1,971595 | 0,048688 | 0,829088 |
| ENSG0000 | 0,001145 | 0,273905 | 1,970242 | 0,048842 | 0,829088 |
| ENSG0000 | 0,000735 | -0,686522 | 1,969186 | 0,048964 | 0,829088 |
| ENSG0000 | 0,000779 | 0,972937 | 1,968079 | 0,049091 | 0,829088 |
| ENSG0000 | -0,000608 | -0,556599 | -1,966846 | 0,049233 | 0,829088 |
| ENSG0000 | -0,002563 | -0,021294 | -1,966811 | 0,049237 | 0,829088 |
| ENSG0000 | -0,002891 | -0,985523 | -1,966305 | 0,049295 | 0,829088 |
| ENSG0000 | -0,00123 | -0,372599 | -1,965552 | 0,049382 | 0,829088 |
| ENSG0000 | 0,000509 | -0,305358 | 1,965244 | 0,049418 | 0,829088 |
| ENSG0000 | -0,001525 | -2,629678 | -1,963908 | 0,049573 | 0,829088 |

| | | | | | |
|---|---|---|---|---|---|
| ENSG0000 | 0,00132 | -2,548264 | 1,961666 | 0,049833 | 0,829088 |

| geneID | logFC | AveExpr | t | P.Value | adj.P.Val |
|---|---|---|---|---|---|
| ENSG0000 | 0,007399 | 1,998693 | 4,721561 | 2,38E-06 | 0,015643 |
| ENSG0000 | 0,001759 | 0,636432 | 4,110613 | 3,98E-05 | 0,094535 |
| ENSG0000 | -0,004039 | -0,536411 | -4,092317 | 4,31E-05 | 0,094535 |
| ENSG0000 | -0,003961 | -0,972503 | -3,881558 | 0,000105 | 0,172023 |
| ENSG0000 | 0,002408 | -0,385819 | 3,72889 | 0,000194 | 0,254747 |
| ENSG0000 | -0,003687 | 0,843592 | -3,643841 | 0,00027 | 0,296325 |
| ENSG0000 | -0,002419 | -0,084561 | -3,598075 | 0,000322 | 0,303068 |
| ENSG0000 | 0,003397 | 0,55219 | 3,533904 | 0,000412 | 0,338591 |
| ENSG0000 | -0,002366 | -0,248278 | -3,499308 | 0,000469 | 0,342799 |
| ENSG0000 | -0,000172 | -0,071839 | -3,457434 | 0,000548 | 0,360611 |
| ENSG0000 | 0,001099 | -0,431026 | 3,355586 | 0,000795 | 0,475844 |
| ENSG0000 | -0,001359 | 0,821647 | -3,253256 | 0,001145 | 0,545616 |
| ENSG0000 | 0,001908 | -0,584146 | 3,223766 | 0,00127 | 0,545616 |
| ENSG0000 | 0,001035 | -0,376627 | 3,222015 | 0,001278 | 0,545616 |
| ENSG0000 | 0,002981 | -0,0751 | 3,177571 | 0,00149 | 0,545616 |
| ENSG0000 | 0,00149 | -0,405268 | 3,172883 | 0,001515 | 0,545616 |
| ENSG0000 | -0,001404 | 0,625279 | -3,169204 | 0,001534 | 0,545616 |
| ENSG0000 | -0,003892 | -0,206468 | -3,135382 | 0,001722 | 0,545616 |
| ENSG0000 | 0,003373 | -0,51391 | 3,132966 | 0,001736 | 0,545616 |
| ENSG0000 | 0,000544 | -0,097702 | 3,124588 | 0,001786 | 0,545616 |
| ENSG0000 | -0,002133 | -0,073375 | -3,112993 | 0,001858 | 0,545616 |
| ENSG0000 | -0,001571 | -0,278818 | -3,10589 | 0,001903 | 0,545616 |
| ENSG0000 | -0,00158 | -0,60233 | -3,105315 | 0,001907 | 0,545616 |
| ENSG0000 | -0,001586 | -0,911879 | -3,089235 | 0,002013 | 0,551998 |
| ENSG0000 | 0,001322 | -0,562469 | 3,070208 | 0,002146 | 0,561107 |
| ENSG0000 | -0,003626 | 0,180432 | -3,060448 | 0,002217 | 0,561107 |
| ENSG0000 | -0,001903 | -0,34929 | -3,038326 | 0,002386 | 0,581573 |
| ENSG0000 | 0,001579 | 0,381345 | 3,00344 | 0,002677 | 0,618057 |
| ENSG0000 | 0,002984 | -1,03418 | 2,993015 | 0,00277 | 0,618057 |
| ENSG0000 | -0,002064 | 0,121432 | -2,987835 | 0,002817 | 0,618057 |
| ENSG0000 | -0,001769 | -0,5673 | -2,959799 | 0,003087 | 0,630502 |
| ENSG0000 | -0,00058 | 0,09259 | -2,950808 | 0,003178 | 0,630502 |
| ENSG0000 | 0,002274 | 0,101628 | 2,942147 | 0,003268 | 0,630502 |
| ENSG0000 | -0,001607 | 0,399764 | -2,923616 | 0,003469 | 0,630502 |
| ENSG0000 | 0,001921 | -0,094325 | 2,919929 | 0,00351 | 0,630502 |
| ENSG0000 | 0,001089 | -0,378838 | 2,917105 | 0,003542 | 0,630502 |
| ENSG0000 | -0,001841 | -1,533867 | -2,916856 | 0,003545 | 0,630502 |
| ENSG0000 | -0,002928 | -1,59147 | -2,885387 | 0,003919 | 0,636986 |
| ENSG0000 | -0,000829 | -0,178374 | -2,885349 | 0,003919 | 0,636986 |
| ENSG0000 | -0,003127 | -1,411874 | -2,885218 | 0,003921 | 0,636986 |
| ENSG0000 | -0,003204 | 0,679742 | -2,881429 | 0,003968 | 0,636986 |
| ENSG0000 | 0,001581 | -1,064756 | 2,859144 | 0,004258 | 0,667182 |
| ENSG0000 | 0,001266 | -1,094443 | 2,819187 | 0,004825 | 0,721921 |
| ENSG0000 | 0,001404 | 0,189128 | 2,819103 | 0,004827 | 0,721921 |
| ENSG0000 | -0,000765 | 0,101966 | -2,787633 | 0,005321 | 0,734212 |
| ENSG0000 | -0,000696 | -0,130029 | -2,778088 | 0,00548 | 0,734212 |
| ENSG0000 | 0,004071 | 2,582882 | 2,769255 | 0,00563 | 0,734212 |
| ENSG0000 | -0,002344 | -0,923037 | -2,768824 | 0,005638 | 0,734212 |

| | | | | | |
|---|---|---|---|---|---|
| ENSG0000 | 0,001535 | 0,432858 | 2,760772 | 0,005779 | 0,734212 |
| ENSG0000 | -0,000781 | 1,292793 | -2,759375 | 0,005803 | 0,734212 |
| ENSG0000 | 0,003884 | 1,006168 | 2,759293 | 0,005805 | 0,734212 |
| ENSG0000 | 0,004865 | -0,013518 | 2,745591 | 0,006053 | 0,734212 |
| ENSG0000 | 0,001375 | -0,634597 | 2,743461 | 0,006092 | 0,734212 |
| ENSG0000 | 0,001341 | 0,301849 | 2,743167 | 0,006097 | 0,734212 |
| ENSG0000 | -0,001864 | 0,943242 | -2,741089 | 0,006136 | 0,734212 |
| ENSG0000 | 0,00201 | -1,259642 | 2,732874 | 0,006291 | 0,73932 |
| ENSG0000 | -0,001249 | 0,419921 | -2,718862 | 0,006564 | 0,75557 |
| ENSG0000 | -0,000597 | -0,06866 | -2,710343 | 0,006735 | 0,75557 |
| ENSG0000 | 0,001532 | -0,426875 | 2,702376 | 0,006898 | 0,75557 |
| ENSG0000 | 0,003751 | -1,633965 | 2,695222 | 0,007048 | 0,75557 |
| ENSG0000 | -0,001029 | 0,10852 | -2,692825 | 0,007099 | 0,75557 |
| ENSG0000 | -0,002492 | 0,089048 | -2,690746 | 0,007143 | 0,75557 |
| ENSG0000 | 0,004871 | -0,356416 | 2,663934 | 0,007738 | 0,75557 |
| ENSG0000 | 0,000946 | 0,96348 | 2,656846 | 0,007902 | 0,75557 |
| ENSG0000 | -0,000668 | 0,155336 | -2,656571 | 0,007908 | 0,75557 |
| ENSG0000 | 0,000805 | 0,343841 | 2,656241 | 0,007916 | 0,75557 |
| ENSG0000 | 0,002627 | 1,587171 | 2,655828 | 0,007926 | 0,75557 |
| ENSG0000 | -0,001401 | -0,132186 | -2,646946 | 0,008137 | 0,75557 |
| ENSG0000 | 0,001094 | -0,175903 | 2,639546 | 0,008317 | 0,75557 |
| ENSG0000 | 0,001453 | -0,244921 | 2,636087 | 0,008402 | 0,75557 |
| ENSG0000 | -0,001347 | 0,203372 | -2,632903 | 0,008481 | 0,75557 |
| ENSG0000 | -0,000761 | -0,16877 | -2,630442 | 0,008542 | 0,75557 |
| ENSG0000 | -0,001237 | 0,218484 | -2,630198 | 0,008549 | 0,75557 |
| ENSG0000 | -0,001701 | -0,26736 | -2,629436 | 0,008568 | 0,75557 |
| ENSG0000 | -0,002796 | 1,78062 | -2,627728 | 0,008611 | 0,75557 |
| ENSG0000 | 0,002121 | -0,378825 | 2,622657 | 0,00874 | 0,756809 |
| ENSG0000 | -0,000295 | -0,098355 | -2,610401 | 0,009059 | 0,765152 |
| ENSG0000 | -0,001879 | 0,381842 | -2,609104 | 0,009094 | 0,765152 |
| ENSG0000 | -0,00166 | 0,293904 | -2,601692 | 0,009292 | 0,765152 |
| ENSG0000 | -0,001351 | 0,307832 | -2,601356 | 0,009301 | 0,765152 |
| ENSG0000 | 0,001004 | -0,277881 | 2,575077 | 0,010038 | 0,776218 |
| ENSG0000 | -0,001144 | -0,502061 | -2,573125 | 0,010095 | 0,776218 |
| ENSG0000 | 0,00167 | -0,533163 | 2,573071 | 0,010096 | 0,776218 |
| ENSG0000 | -0,000891 | 0,053657 | -2,572539 | 0,010112 | 0,776218 |
| ENSG0000 | 0,001009 | 0,246626 | 2,571908 | 0,01013 | 0,776218 |
| ENSG0000 | -0,002163 | 0,660759 | -2,567767 | 0,010252 | 0,776218 |
| ENSG0000 | -0,001178 | -0,678673 | -2,565321 | 0,010325 | 0,776218 |
| ENSG0000 | 0,001593 | -0,21274 | 2,563483 | 0,010379 | 0,776218 |
| ENSG0000 | -0,003623 | -0,831122 | -2,558203 | 0,010538 | 0,777509 |
| ENSG0000 | 0,001511 | 0,640543 | 2,554658 | 0,010646 | 0,777509 |
| ENSG0000 | -0,00157 | 0,164589 | -2,549114 | 0,010817 | 0,777509 |
| ENSG0000 | -0,002637 | 1,084309 | -2,537566 | 0,01118 | 0,777509 |
| ENSG0000 | 0,002095 | -0,667552 | 2,536596 | 0,011211 | 0,777509 |
| ENSG0000 | -0,000985 | -1,08218 | -2,53596 | 0,011231 | 0,777509 |
| ENSG0000 | -0,001169 | 0,607256 | -2,53576 | 0,011238 | 0,777509 |
| ENSG0000 | -0,001131 | 0,24714 | -2,529411 | 0,011443 | 0,777509 |

| | | | | | |
|---|---|---|---|---|---|
| ENSG0000 | -0,001849 | 1,212543 | -2,525014 | 0,011587 | 0,777509 |
| ENSG0000 | -0,000686 | -0,109784 | -2,522319 | 0,011676 | 0,777509 |
| ENSG0000 | 0,000911 | -0,197169 | 2,519786 | 0,01176 | 0,777509 |
| ENSG0000 | -0,00124 | 0,197678 | -2,512522 | 0,012005 | 0,777509 |
| ENSG0000 | 0,003502 | -1,542261 | 2,510103 | 0,012088 | 0,777509 |
| ENSG0000 | 0,001944 | -0,726741 | 2,508794 | 0,012132 | 0,777509 |
| ENSG0000 | 0,001335 | 0,441156 | 2,504199 | 0,012291 | 0,777509 |
| ENSG0000 | -0,000655 | -0,1289 | -2,503179 | 0,012327 | 0,777509 |
| ENSG0000 | 0,003341 | 1,552878 | 2,498883 | 0,012477 | 0,777509 |
| ENSG0000 | -0,002457 | -1,401414 | -2,493883 | 0,012654 | 0,777509 |
| ENSG0000 | 0,001966 | 1,814375 | 2,488562 | 0,012845 | 0,777509 |
| ENSG0000 | 0,001119 | 0,215669 | 2,485463 | 0,012957 | 0,777509 |
| ENSG0000 | 0,000789 | 0,825511 | 2,48374 | 0,01302 | 0,777509 |
| ENSG0000 | 0,003238 | 1,655712 | 2,475172 | 0,013336 | 0,777509 |
| ENSG0000 | 0,00141 | 0,259519 | 2,468583 | 0,013584 | 0,777509 |
| ENSG0000 | -0,002016 | 1,293652 | -2,463537 | 0,013777 | 0,777509 |
| ENSG0000 | -0,002669 | -0,186759 | -2,458454 | 0,013973 | 0,777509 |
| ENSG0000 | 0,001681 | 0,534009 | 2,456767 | 0,014039 | 0,777509 |
| ENSG0000 | -0,001397 | 0,448828 | -2,454618 | 0,014123 | 0,777509 |
| ENSG0000 | 0,000868 | 0,352969 | 2,454258 | 0,014137 | 0,777509 |
| ENSG0000 | -0,000371 | 0,136605 | -2,449333 | 0,014332 | 0,777509 |
| ENSG0000 | -0,001727 | 0,286816 | -2,44553 | 0,014484 | 0,777509 |
| ENSG0000 | -0,001247 | 1,416073 | -2,445512 | 0,014484 | 0,777509 |
| ENSG0000 | -0,001525 | 0,550872 | -2,444826 | 0,014512 | 0,777509 |
| ENSG0000 | 0,002137 | -0,282368 | 2,440589 | 0,014683 | 0,777509 |
| ENSG0000 | -0,002011 | -0,496078 | -2,440236 | 0,014698 | 0,777509 |
| ENSG0000 | -0,002018 | 0,760627 | -2,435909 | 0,014874 | 0,777509 |
| ENSG0000 | -0,002756 | 0,127004 | -2,431565 | 0,015054 | 0,777509 |
| ENSG0000 | -0,001762 | -1,132124 | -2,429176 | 0,015153 | 0,777509 |
| ENSG0000 | -0,003189 | -1,063749 | -2,424841 | 0,015335 | 0,777509 |
| ENSG0000 | -0,001179 | -0,30332 | -2,42339 | 0,015397 | 0,777509 |
| ENSG0000 | -0,002291 | 1,718667 | -2,420765 | 0,015508 | 0,777509 |
| ENSG0000 | -0,00122 | 0,943438 | -2,412889 | 0,015847 | 0,777509 |
| ENSG0000 | 0,000716 | 1,049404 | 2,406756 | 0,016116 | 0,777509 |
| ENSG0000 | 0,001724 | 0,316564 | 2,405639 | 0,016165 | 0,777509 |
| ENSG0000 | 0,002865 | -0,705709 | 2,402276 | 0,016314 | 0,777509 |
| ENSG0000 | 0,000446 | 0,026676 | 2,394775 | 0,016652 | 0,777509 |
| ENSG0000 | 0,000406 | -0,058989 | 2,393778 | 0,016697 | 0,777509 |
| ENSG0000 | -0,002933 | 1,283529 | -2,389836 | 0,016877 | 0,777509 |
| ENSG0000 | -0,001176 | -0,667036 | -2,389621 | 0,016887 | 0,777509 |
| ENSG0000 | -0,003294 | 2,093883 | -2,38491 | 0,017105 | 0,777509 |
| ENSG0000 | 0,002111 | 0,610882 | 2,384298 | 0,017133 | 0,777509 |
| ENSG0000 | 0,000577 | 0,207028 | 2,381524 | 0,017263 | 0,777509 |
| ENSG0000 | -0,001518 | 0,259342 | -2,381243 | 0,017276 | 0,777509 |
| ENSG0000 | 0,001405 | 0,231611 | 2,377145 | 0,017469 | 0,777509 |
| ENSG0000 | 0,001002 | -0,216064 | 2,374249 | 0,017606 | 0,777509 |
| ENSG0000 | 0,00114 | -0,836818 | 2,372861 | 0,017673 | 0,777509 |
| ENSG0000 | -0,001283 | 0,162032 | -2,371648 | 0,017731 | 0,777509 |

| | | | | | |
|---|---|---|---|---|---|
| ENSG0000 | 0,001636 | -0,36087 | 2,369779 | 0,01782 | 0,777509 |
| ENSG0000 | 0,000812 | 0,530493 | 2,368771 | 0,017869 | 0,777509 |
| ENSG0000 | 0,000217 | -0,137053 | 2,365688 | 0,018018 | 0,777509 |
| ENSG0000 | -0,000408 | -0,195213 | -2,365517 | 0,018027 | 0,777509 |
| ENSG0000 | 0,000546 | 0,837687 | 2,364299 | 0,018086 | 0,777509 |
| ENSG0000 | -0,001736 | 0,300692 | -2,362601 | 0,018169 | 0,777509 |
| ENSG0000 | -0,002204 | -0,425267 | -2,360113 | 0,018291 | 0,777509 |
| ENSG0000 | 0,001335 | -0,357582 | 2,359659 | 0,018314 | 0,777509 |
| ENSG0000 | -0,001041 | 0,357897 | -2,355298 | 0,01853 | 0,777509 |
| ENSG0000 | 0,004601 | 2,382356 | 2,355036 | 0,018543 | 0,777509 |
| ENSG0000 | -0,003353 | -0,57075 | -2,353653 | 0,018612 | 0,777509 |
| ENSG0000 | -0,002707 | 4,831258 | -2,353134 | 0,018638 | 0,777509 |
| ENSG0000 | -0,003671 | 1,154995 | -2,352483 | 0,018671 | 0,777509 |
| ENSG0000 | 0,002214 | 0,721578 | 2,351046 | 0,018743 | 0,777509 |
| ENSG0000 | 0,002662 | -0,761154 | 2,34713 | 0,018941 | 0,777509 |
| ENSG0000 | 0,000572 | -0,409239 | 2,343209 | 0,019141 | 0,777509 |
| ENSG0000 | -0,001049 | 1,820777 | -2,343109 | 0,019146 | 0,777509 |
| ENSG0000 | -0,002268 | 0,887156 | -2,340247 | 0,019293 | 0,777509 |
| ENSG0000 | 0,000699 | 0,343228 | 2,339609 | 0,019326 | 0,777509 |
| ENSG0000 | -0,001577 | 0,934756 | -2,338658 | 0,019376 | 0,777509 |
| ENSG0000 | -0,000883 | 0,591515 | -2,335283 | 0,019551 | 0,778577 |
| ENSG0000 | 0,00149 | 0,699552 | 2,332881 | 0,019677 | 0,778577 |
| ENSG0000 | 0,000963 | 0,87359 | 2,328941 | 0,019885 | 0,778577 |
| ENSG0000 | -0,001854 | -1,376322 | -2,326481 | 0,020016 | 0,778577 |
| ENSG0000 | 0,001013 | 0,366438 | 2,326107 | 0,020036 | 0,778577 |
| ENSG0000 | -0,000743 | -0,216103 | -2,324677 | 0,020112 | 0,778577 |
| ENSG0000 | 0,001661 | 0,923143 | 2,321945 | 0,020259 | 0,77967 |
| ENSG0000 | 0,001445 | 0,469846 | 2,319195 | 0,020408 | 0,780825 |
| ENSG0000 | 0,001114 | 0,041488 | 2,312895 | 0,020752 | 0,788711 |
| ENSG0000 | 0,001258 | 0,966848 | 2,309829 | 0,020921 | 0,788711 |
| ENSG0000 | -0,003382 | 0,401208 | -2,307576 | 0,021046 | 0,788711 |
| ENSG0000 | -0,000934 | 0,009545 | -2,300993 | 0,021416 | 0,788711 |
| ENSG0000 | -0,001126 | -0,052215 | -2,297248 | 0,021628 | 0,788711 |
| ENSG0000 | 0,002484 | -0,48594 | 2,297159 | 0,021633 | 0,788711 |
| ENSG0000 | 0,000404 | 0,374648 | 2,29663 | 0,021664 | 0,788711 |
| ENSG0000 | -0,001247 | 0,370742 | -2,294885 | 0,021763 | 0,788711 |
| ENSG0000 | 0,001913 | 0,008044 | 2,294613 | 0,021779 | 0,788711 |
| ENSG0000 | 0,002067 | 0,713003 | 2,290154 | 0,022036 | 0,788711 |
| ENSG0000 | 0,001253 | 0,899003 | 2,288618 | 0,022125 | 0,788711 |
| ENSG0000 | -0,001064 | 0,565347 | -2,286479 | 0,02225 | 0,788711 |
| ENSG0000 | -0,001083 | -0,165089 | -2,28539 | 0,022314 | 0,788711 |
| ENSG0000 | 0,001198 | -0,31572 | 2,281402 | 0,022549 | 0,788711 |
| ENSG0000 | -0,00151 | 0,453665 | -2,280581 | 0,022597 | 0,788711 |
| ENSG0000 | 0,001513 | -0,193901 | 2,280364 | 0,02261 | 0,788711 |
| ENSG0000 | 0,001125 | 0,104821 | 2,27368 | 0,02301 | 0,788711 |
| ENSG0000 | -0,000988 | -0,27222 | -2,272476 | 0,023082 | 0,788711 |
| ENSG0000 | 0,001443 | -0,500984 | 2,270846 | 0,023181 | 0,788711 |
| ENSG0000 | 0,000816 | -1,179852 | 2,266199 | 0,023464 | 0,788711 |

| | | | | | |
|---|---|---|---|---|---|
| ENSG0000 | 0,000313 | 0,067217 | 2,262321 | 0,023702 | 0,788711 |
| ENSG0000 | 0,001493 | 0,231983 | 2,262128 | 0,023714 | 0,788711 |
| ENSG0000 | 0,001863 | 0,29837 | 2,260829 | 0,023794 | 0,788711 |
| ENSG0000 | -0,001948 | -0,0405 | -2,258331 | 0,02395 | 0,788711 |
| ENSG0000 | 0,001122 | 2,139595 | 2,256551 | 0,024061 | 0,788711 |
| ENSG0000 | -0,001448 | 0,470195 | -2,256292 | 0,024077 | 0,788711 |
| ENSG0000 | 0,000746 | -0,086432 | 2,256047 | 0,024092 | 0,788711 |
| ENSG0000 | -0,001638 | 0,26235 | -2,255899 | 0,024102 | 0,788711 |
| ENSG0000 | 0,001581 | 0,593586 | 2,24987 | 0,024482 | 0,788711 |
| ENSG0000 | 0,003217 | 0,372119 | 2,248889 | 0,024545 | 0,788711 |
| ENSG0000 | 0,001461 | 0,526687 | 2,246081 | 0,024724 | 0,788711 |
| ENSG0000 | -0,000654 | 0,496982 | -2,245448 | 0,024764 | 0,788711 |
| ENSG0000 | -0,002023 | -0,421892 | -2,241465 | 0,025021 | 0,788711 |
| ENSG0000 | 0,001589 | 0,79154 | 2,241146 | 0,025042 | 0,788711 |
| ENSG0000 | -0,00119 | 1,735689 | -2,23997 | 0,025118 | 0,788711 |
| ENSG0000 | -0,001347 | 0,130238 | -2,239037 | 0,025179 | 0,788711 |
| ENSG0000 | -0,000797 | 1,215422 | -2,238083 | 0,025241 | 0,788711 |
| ENSG0000 | 0,002253 | 0,81518 | 2,236966 | 0,025314 | 0,788711 |
| ENSG0000 | 0,00137 | -1,183267 | 2,236371 | 0,025353 | 0,788711 |
| ENSG0000 | 0,000537 | -1,723631 | 2,235535 | 0,025408 | 0,788711 |
| ENSG0000 | -0,002931 | -0,611339 | -2,225249 | 0,02609 | 0,797807 |
| ENSG0000 | 0,001358 | 0,159338 | 2,224409 | 0,026147 | 0,797807 |
| ENSG0000 | -0,002246 | -0,009225 | -2,223623 | 0,026199 | 0,797807 |
| ENSG0000 | 0,001548 | -0,822621 | 2,217366 | 0,026624 | 0,797807 |
| ENSG0000 | -0,000718 | -0,240635 | -2,217061 | 0,026645 | 0,797807 |
| ENSG0000 | 0,001185 | -0,511714 | 2,214787 | 0,026801 | 0,797807 |
| ENSG0000 | 0,000998 | 0,283776 | 2,213206 | 0,026909 | 0,797807 |
| ENSG0000 | 0,001865 | 2,660645 | 2,212656 | 0,026947 | 0,797807 |
| ENSG0000 | 0,001578 | 2,557741 | 2,212505 | 0,026958 | 0,797807 |
| ENSG0000 | -0,002505 | 0,899291 | -2,21227 | 0,026974 | 0,797807 |
| ENSG0000 | -0,00216 | -0,24441 | -2,2114 | 0,027034 | 0,797807 |
| ENSG0000 | 0,002002 | -0,382718 | 2,208824 | 0,027213 | 0,798575 |
| ENSG0000 | 0,001171 | 0,154935 | 2,207535 | 0,027303 | 0,798575 |
| ENSG0000 | 0,002455 | 1,312059 | 2,201833 | 0,027703 | 0,806705 |
| ENSG0000 | -0,000525 | -0,208914 | -2,197906 | 0,027982 | 0,809654 |
| ENSG0000 | -0,001125 | 0,135726 | -2,196946 | 0,028051 | 0,809654 |
| ENSG0000 | 0,002806 | 2,05593 | 2,193262 | 0,028315 | 0,810343 |
| ENSG0000 | 0,001617 | -0,034865 | 2,192897 | 0,028341 | 0,810343 |
| ENSG0000 | -0,001988 | 1,314349 | -2,189947 | 0,028555 | 0,810343 |
| ENSG0000 | -0,000698 | -1,744498 | -2,189776 | 0,028567 | 0,810343 |
| ENSG0000 | -0,001497 | 0,035973 | -2,18594 | 0,028847 | 0,814088 |
| ENSG0000 | 0,000708 | -0,438156 | 2,184578 | 0,028946 | 0,814088 |
| ENSG0000 | 0,001367 | -0,3821 | 2,178087 | 0,029426 | 0,82207 |
| ENSG0000 | -0,001119 | -0,299754 | -2,176969 | 0,02951 | 0,82207 |
| ENSG0000 | -0,002059 | 1,349212 | -2,174615 | 0,029686 | 0,82207 |
| ENSG0000 | -0,000475 | -0,839574 | -2,166508 | 0,0303 | 0,82207 |
| ENSG0000 | -0,001099 | -0,034756 | -2,166322 | 0,030314 | 0,82207 |
| ENSG0000 | -0,003279 | -0,812291 | -2,165475 | 0,030378 | 0,82207 |

| | | | | | |
|---|---|---|---|---|---|
| ENSG0000 | 0,00143 | 1,442917 | 2,165335 | 0,030389 | 0,82207 |
| ENSG0000 | 0,003697 | 3,660671 | 2,163741 | 0,030511 | 0,82207 |
| ENSG0000 | 0,001094 | 1,183531 | 2,157386 | 0,031003 | 0,82207 |
| ENSG0000 | 0,001819 | -1,027795 | 2,15521 | 0,031173 | 0,82207 |
| ENSG0000 | -0,000963 | 1,555155 | -2,154877 | 0,031199 | 0,82207 |
| ENSG0000 | 0,002921 | -0,949647 | 2,153881 | 0,031277 | 0,82207 |
| ENSG0000 | -0,001135 | 0,519881 | -2,153868 | 0,031278 | 0,82207 |
| ENSG0000 | 0,003327 | 0,098235 | 2,153559 | 0,031302 | 0,82207 |
| ENSG0000 | 0,001713 | -0,660034 | 2,151733 | 0,031446 | 0,82207 |
| ENSG0000 | 0,00251 | 0,633983 | 2,150919 | 0,03151 | 0,82207 |
| ENSG0000 | 0,001526 | -0,272347 | 2,14962 | 0,031613 | 0,82207 |
| ENSG0000 | 0,002171 | 0,466739 | 2,149498 | 0,031622 | 0,82207 |
| ENSG0000 | 0,001229 | 0,699914 | 2,147973 | 0,031743 | 0,82207 |
| ENSG0000 | -0,000899 | 0,598336 | -2,144723 | 0,032003 | 0,82207 |
| ENSG0000 | -0,000597 | 0,14623 | -2,13992 | 0,032389 | 0,82207 |
| ENSG0000 | -0,001604 | 1,394785 | -2,138153 | 0,032532 | 0,82207 |
| ENSG0000 | -0,000265 | -0,539335 | -2,137868 | 0,032555 | 0,82207 |
| ENSG0000 | 0,000658 | -0,105061 | 2,134952 | 0,032793 | 0,82207 |
| ENSG0000 | -0,001538 | -0,343282 | -2,134577 | 0,032823 | 0,82207 |
| ENSG0000 | -0,001204 | -1,096816 | -2,132454 | 0,032997 | 0,82207 |
| ENSG0000 | 0,001173 | -0,042161 | 2,131704 | 0,033059 | 0,82207 |
| ENSG0000 | -0,000784 | -0,356954 | -2,129484 | 0,033242 | 0,82207 |
| ENSG0000 | -0,001317 | -1,956627 | -2,128374 | 0,033334 | 0,82207 |
| ENSG0000 | 0,001259 | 1,991836 | 2,127702 | 0,03339 | 0,82207 |
| ENSG0000 | 0,00241 | 0,440951 | 2,124486 | 0,033658 | 0,82207 |
| ENSG0000 | 0,000563 | 0,055796 | 2,123664 | 0,033726 | 0,82207 |
| ENSG0000 | 0,000607 | -0,533168 | 2,122974 | 0,033784 | 0,82207 |
| ENSG0000 | 0,000768 | -0,089478 | 2,121861 | 0,033878 | 0,82207 |
| ENSG0000 | 0,002382 | 0,968953 | 2,120547 | 0,033988 | 0,82207 |
| ENSG0000 | -0,001052 | -0,304821 | -2,119982 | 0,034036 | 0,82207 |
| ENSG0000 | -0,001266 | 0,782532 | -2,11918 | 0,034104 | 0,82207 |
| ENSG0000 | 0,00103 | -0,812975 | 2,11907 | 0,034113 | 0,82207 |
| ENSG0000 | 0,001845 | -0,516934 | 2,118507 | 0,03416 | 0,82207 |
| ENSG0000 | 0,000971 | -0,345124 | 2,117722 | 0,034227 | 0,82207 |
| ENSG0000 | -0,001659 | 0,433852 | -2,114872 | 0,034469 | 0,82488 |
| ENSG0000 | -0,001467 | -1,488305 | -2,113014 | 0,034628 | 0,825676 |
| ENSG0000 | -0,000604 | -0,258198 | -2,10988 | 0,034897 | 0,829092 |
| ENSG0000 | 0,000344 | 0,179644 | 2,105829 | 0,035248 | 0,829753 |
| ENSG0000 | 0,000474 | 0,258798 | 2,104281 | 0,035383 | 0,829753 |
| ENSG0000 | -0,001416 | 1,485693 | -2,103378 | 0,035461 | 0,829753 |
| ENSG0000 | -0,000968 | -0,531469 | -2,103251 | 0,035472 | 0,829753 |
| ENSG0000 | 0,001261 | -0,071355 | 2,096781 | 0,036042 | 0,829753 |
| ENSG0000 | 0,001255 | -0,479367 | 2,093825 | 0,036304 | 0,829753 |
| ENSG0000 | -0,000775 | 0,294231 | -2,091979 | 0,036469 | 0,829753 |
| ENSG0000 | 0,00181 | 0,435943 | 2,09117 | 0,036542 | 0,829753 |
| ENSG0000 | -0,001016 | 0,007234 | -2,090898 | 0,036566 | 0,829753 |
| ENSG0000 | -0,00112 | -0,056686 | -2,09061 | 0,036592 | 0,829753 |
| ENSG0000 | 0,000538 | -0,186938 | 2,08885 | 0,03675 | 0,829753 |

| | | | | | |
|---|---|---|---|---|---|
| ENSG0000 | 0,001303 | -0,239682 | 2,086652 | 0,036949 | 0,829753 |
| ENSG0000 | -0,001819 | -0,681048 | -2,083499 | 0,037235 | 0,829753 |
| ENSG0000 | 0,000615 | -0,343187 | 2,083343 | 0,037249 | 0,829753 |
| ENSG0000 | -0,00298 | -0,467958 | -2,083139 | 0,037268 | 0,829753 |
| ENSG0000 | 0,001572 | -0,09494 | 2,082231 | 0,037351 | 0,829753 |
| ENSG0000 | 0,000835 | -0,30009 | 2,082019 | 0,03737 | 0,829753 |
| ENSG0000 | -0,000803 | 0,247431 | -2,081164 | 0,037448 | 0,829753 |
| ENSG0000 | -0,00075 | -0,193447 | -2,080034 | 0,037552 | 0,829753 |
| ENSG0000 | -0,002223 | 0,383548 | -2,078375 | 0,037704 | 0,829753 |
| ENSG0000 | -0,003034 | -1,699157 | -2,078031 | 0,037736 | 0,829753 |
| ENSG0000 | 0,00072 | -0,069178 | 2,076335 | 0,037892 | 0,829753 |
| ENSG0000 | -0,001209 | -0,627073 | -2,075172 | 0,038 | 0,829753 |
| ENSG0000 | -0,000928 | -0,37627 | -2,074473 | 0,038065 | 0,829753 |
| ENSG0000 | 0,001334 | 0,067946 | 2,074252 | 0,038085 | 0,829753 |
| ENSG0000 | -0,001149 | 0,528426 | -2,072626 | 0,038236 | 0,829753 |
| ENSG0000 | 0,001881 | -0,018585 | 2,070213 | 0,038462 | 0,829753 |
| ENSG0000 | 0,000478 | 0,170018 | 2,068178 | 0,038653 | 0,829753 |
| ENSG0000 | -0,000586 | -0,057337 | -2,067726 | 0,038695 | 0,829753 |
| ENSG0000 | 0,001293 | 0,217395 | 2,067596 | 0,038708 | 0,829753 |
| ENSG0000 | 0,002485 | -0,540872 | 2,066186 | 0,03884 | 0,829899 |
| ENSG0000 | -0,003013 | -2,245519 | -2,061547 | 0,039281 | 0,833062 |
| ENSG0000 | 0,00152 | 0,495274 | 2,059981 | 0,03943 | 0,833062 |
| ENSG0000 | -0,000369 | 0,074446 | -2,058019 | 0,039618 | 0,833062 |
| ENSG0000 | 0,001111 | 0,166379 | 2,057998 | 0,03962 | 0,833062 |
| ENSG0000 | -0,000536 | 0,809175 | -2,057536 | 0,039664 | 0,833062 |
| ENSG0000 | 0,000478 | -0,115013 | 2,056668 | 0,039748 | 0,833062 |
| ENSG0000 | 0,000635 | 0,198083 | 2,053853 | 0,04002 | 0,836096 |
| ENSG0000 | -0,001781 | -0,247394 | -2,052345 | 0,040166 | 0,836495 |
| ENSG0000 | 0,001762 | 2,215134 | 2,047817 | 0,040608 | 0,839122 |
| ENSG0000 | -0,001162 | -0,318594 | -2,047234 | 0,040665 | 0,839122 |
| ENSG0000 | -0,000872 | -0,783273 | -2,047137 | 0,040675 | 0,839122 |
| ENSG0000 | 0,001014 | 0,677152 | 2,044163 | 0,040968 | 0,842504 |
| ENSG0000 | 0,00144 | -1,341837 | 2,042333 | 0,041149 | 0,842504 |
| ENSG0000 | 0,001813 | 1,484864 | 2,041313 | 0,04125 | 0,842504 |
| ENSG0000 | 0,000998 | 0,32022 | 2,04029 | 0,041352 | 0,842504 |
| ENSG0000 | 0,000274 | 0,040329 | 2,038622 | 0,041518 | 0,842504 |
| ENSG0000 | -0,000399 | -0,21687 | -2,036615 | 0,041719 | 0,842504 |
| ENSG0000 | 0,000962 | -0,627895 | 2,035193 | 0,041862 | 0,842504 |
| ENSG0000 | 0,001142 | -0,868563 | 2,035183 | 0,041863 | 0,842504 |
| ENSG0000 | 0,001646 | -1,05491 | 2,031482 | 0,042236 | 0,844963 |
| ENSG0000 | -0,001306 | -0,678827 | -2,031308 | 0,042254 | 0,844963 |
| ENSG0000 | 0,000992 | 0,020458 | 2,030166 | 0,04237 | 0,844963 |
| ENSG0000 | -0,001213 | -0,441628 | -2,027134 | 0,042679 | 0,848554 |
| ENSG0000 | 0,001675 | -0,545087 | 2,02521 | 0,042876 | 0,849907 |
| ENSG0000 | 0,000654 | 0,454077 | 2,023552 | 0,043047 | 0,850724 |
| ENSG0000 | 0,003118 | -2,121647 | 2,020966 | 0,043314 | 0,853439 |
| ENSG0000 | -0,001121 | 0,375645 | -2,018767 | 0,043542 | 0,855374 |
| ENSG0000 | -0,001247 | -0,450121 | -2,015283 | 0,043906 | 0,856556 |

| | | | | | |
|---|---|---|---|---|---|
| ENSG0000 | -0,000818 | 0,18308 | -2,014068 | 0,044033 | 0,856556 |
| ENSG0000 | 0,001475 | -1,014915 | 2,012679 | 0,044179 | 0,856556 |
| ENSG0000 | -0,001131 | 0,444278 | -2,011214 | 0,044334 | 0,856556 |
| ENSG0000 | 0,000661 | 0,272066 | 2,008921 | 0,044576 | 0,856556 |
| ENSG0000 | -0,000741 | -0,209304 | -2,005116 | 0,044982 | 0,856556 |
| ENSG0000 | 0,001336 | -1,267005 | 2,003186 | 0,045188 | 0,856556 |
| ENSG0000 | -0,001836 | 0,298067 | -2,00131 | 0,04539 | 0,856556 |
| ENSG0000 | -0,000222 | 0,362559 | -2,001297 | 0,045391 | 0,856556 |
| ENSG0000 | -0,00086 | 0,400146 | -2,000849 | 0,04544 | 0,856556 |
| ENSG0000 | -0,000648 | 0,343029 | -2,000803 | 0,045445 | 0,856556 |
| ENSG0000 | 0,002565 | 1,309549 | 1,997635 | 0,045787 | 0,856556 |
| ENSG0000 | -0,000275 | -0,079433 | -1,997452 | 0,045807 | 0,856556 |
| ENSG0000 | 0,000591 | -0,721537 | 1,996562 | 0,045904 | 0,856556 |
| ENSG0000 | 0,001522 | -0,617115 | 1,994653 | 0,046112 | 0,856556 |
| ENSG0000 | 0,001695 | -0,421693 | 1,994264 | 0,046154 | 0,856556 |
| ENSG0000 | 0,000884 | 0,165715 | 1,993339 | 0,046256 | 0,856556 |
| ENSG0000 | -0,002092 | 1,264981 | -1,992848 | 0,046309 | 0,856556 |
| ENSG0000 | 0,00205 | 0,959621 | 1,992803 | 0,046314 | 0,856556 |
| ENSG0000 | 0,003105 | 1,522752 | 1,99243 | 0,046355 | 0,856556 |
| ENSG0000 | 0,000796 | 0,291179 | 1,990682 | 0,046547 | 0,856556 |
| ENSG0000 | -0,001918 | 1,290209 | -1,990156 | 0,046605 | 0,856556 |
| ENSG0000 | -0,001393 | 0,42063 | -1,99004 | 0,046618 | 0,856556 |
| ENSG0000 | 0,000805 | -0,067878 | 1,989059 | 0,046726 | 0,856556 |
| ENSG0000 | -0,001638 | 0,630417 | -1,986024 | 0,047062 | 0,857092 |
| ENSG0000 | 0,00125 | 1,128658 | 1,983526 | 0,04734 | 0,857092 |
| ENSG0000 | 0,000967 | -0,135861 | 1,98285 | 0,047416 | 0,857092 |
| ENSG0000 | 0,000429 | 0,347966 | 1,982325 | 0,047474 | 0,857092 |
| ENSG0000 | 0,000945 | -0,058622 | 1,981669 | 0,047548 | 0,857092 |
| ENSG0000 | -0,001977 | 1,423677 | -1,981405 | 0,047577 | 0,857092 |
| ENSG0000 | -0,000725 | 0,27261 | -1,979885 | 0,047748 | 0,857092 |
| ENSG0000 | -0,001433 | 0,234855 | -1,979448 | 0,047797 | 0,857092 |
| ENSG0000 | -0,000956 | 0,038079 | -1,974645 | 0,04834 | 0,858526 |
| ENSG0000 | -0,000732 | -0,100706 | -1,973778 | 0,048439 | 0,858526 |
| ENSG0000 | 0,002839 | 0,233437 | 1,970008 | 0,048869 | 0,858526 |
| ENSG0000 | 0,000965 | 1,06852 | 1,969826 | 0,04889 | 0,858526 |
| ENSG0000 | -0,000997 | 0,66553 | -1,966337 | 0,049291 | 0,858526 |
| ENSG0000 | -0,001741 | 0,266998 | -1,965529 | 0,049385 | 0,858526 |
| ENSG0000 | -0,003087 | -0,077799 | -1,96434 | 0,049522 | 0,858526 |
| ENSG0000 | -0,001715 | 1,057593 | -1,963657 | 0,049602 | 0,858526 |
| ENSG0000 | 0,001146 | 0,298773 | 1,961583 | 0,049843 | 0,858526 |
| ENSG0000 | -0,001551 | -0,231855 | -1,960639 | 0,049953 | 0,858526 |
| ENSG0000 | 0,000538 | -0,311384 | 1,960558 | 0,049962 | 0,858526 |
| ENSG0000 | 0,00106 | 0,212227 | 1,960338 | 0,049988 | 0,858526 |
