# Supplementary material for: The transcriptome-wide association search for genes and genetic variants which associate with BMI and gestational weight gain in women with type 1 diabetes
Source: Mol Med. 2021 Jan 20;27:6. doi: 10.1186/s10020-020-00266-z (PMC7818927; doi:10.1186/s10020-020-00266-z)
Supplement: Supplementary file 7 — Additional file 7: Table S4a.The results of PrediXcan on BMI in T2D cohort in Subcutaneous Adipose Tissue. b. The results of PrediXcan on BMI in T2D cohort in Visceral Adipose Tissue. c. The results of PrediXcan on BMI in ARIC cohort in Subcutaneous Adipose Tissue. d. The results of PrediXcan on BMI in ARIC cohort in Visceral Adipose Tissue. [file 10020_2020_266_MOESM7_ESM.zip › Table S4b.pdf]

geneID	logFC	AveExpr	t	P.Value	adj.P.Val
ENSG0000	-0,010367	-0,389498	-4,640994	4,31E-06	0,027757
ENSG0000	0,005434	0,081972	4,544113	6,75E-06	0,027757
ENSG0000	0,008107	0,288143	4,395054	1,32E-05	0,036273
ENSG0000	-0,008047	0,089479	-4,29015	2,10E-05	0,04319
ENSG0000	-0,004144	0,647951	-4,131732	4,14E-05	0,060763
ENSG0000	-0,00923	-1,232064	-4,115876	4,43E-05	0,060763
ENSG0000	0,005407	0,391538	4,012486	6,81E-05	0,080113
ENSG0000	0,00709	-0,972179	3,95725	8,54E-05	0,087896
ENSG0000	-0,005016	-0,005772	-3,91849	1,00E-04	0,091427
ENSG0000	-0,009799	-1,315013	-3,859481	0,000127	0,104264
ENSG0000	0,002807	0,243103	3,833888	0,00014	0,104366
ENSG0000	-0,007079	2,143726	-3,808699	0,000155	0,104366
ENSG0000	0,005554	-0,176715	3,792938	0,000165	0,104366
ENSG0000	-0,009229	-0,119117	-3,759294	0,000188	0,11055
ENSG0000	-0,001454	-0,027617	-3,733571	0,000208	0,114032
ENSG0000	0,007035	0,748125	3,691729	0,000244	0,121365
ENSG0000	0,006679	0,738422	3,684935	0,000251	0,121365
ENSG0000	-0,005079	0,293042	-3,657259	0,000279	0,121879
ENSG0000	0,007779	-1,346614	3,654716	0,000281	0,121879
ENSG0000	0,003514	-0,182877	3,638097	0,0003	0,123333
ENSG0000	-0,007686	0,213575	-3,572826	0,000383	0,140248
ENSG0000	-0,012675	-1,913372	-3,542351	0,000429	0,140248
ENSG0000	0,009541	1,4594	3,542324	0,000429	0,140248
ENSG0000	-0,002707	-0,4985	-3,528607	0,000452	0,140248
ENSG0000	-0,008663	-0,215037	-3,515071	0,000475	0,140248
ENSG0000	0,005133	0,385365	3,507351	0,000488	0,140248
ENSG0000	-0,008445	-0,463762	-3,507111	0,000489	0,140248
ENSG0000	-0,009152	4,187076	-3,50283	0,000497	0,140248
ENSG0000	-0,002623	0,134267	-3,501253	0,0005	0,140248
ENSG0000	-0,003624	-0,864507	-3,494931	0,000511	0,140248
ENSG0000	0,0037	-0,347987	3,474658	0,000551	0,141359
ENSG0000	0,009224	-0,108636	3,468033	0,000564	0,141359
ENSG0000	0,001853	0,236866	3,459611	0,000582	0,141359
ENSG0000	-0,007927	0,604869	-3,458487	0,000584	0,141359
ENSG0000	0,006436	0,396966	3,439042	0,000627	0,147353
ENSG0000	0,002502	-0,1062	3,382353	0,000768	0,171786
ENSG0000	-0,003438	-0,4567	-3,380864	0,000772	0,171786
ENSG0000	-0,007686	0,187875	-3,360242	0,000831	0,1742
ENSG0000	-0,002463	0,859238	-3,357522	0,000839	0,1742
ENSG0000	-0,005797	-0,456968	-3,351168	0,000858	0,1742
ENSG0000	-0,003634	-0,627118	-3,348037	0,000868	0,1742
ENSG0000	-0,008816	-0,990198	-3,335099	0,000908	0,178004
ENSG0000	-0,000471	0,100837	-3,316116	0,000971	0,185872
ENSG0000	-0,002456	-0,02739	-3,300185	0,001027	0,190163
ENSG0000	0,003519	0,190025	3,296612	0,00104	0,190163
ENSG0000	0,006742	-0,075875	3,27899	0,001106	0,191505
ENSG0000	0,0045	-0,365837	3,272937	0,001129	0,191505
ENSG0000	-0,00541	-0,88867	-3,267168	0,001152	0,191505

ENSG0000	0,015204	0,49303	3,260699	0,001178	0,191505
ENSG0000	-0,006171	-0,124502	-3,259522	0,001183	0,191505
ENSG0000	0,001129	0,39435	3,254834	0,001202	0,191505
ENSG0000	0,010374	-0,693761	3,235042	0,001287	0,191505
ENSG0000	-0,00276	0,39491	-3,233997	0,001292	0,191505
ENSG0000	0,009027	0,41273	3,2243	0,001335	0,191505
ENSG0000	-0,006617	0,02179	-3,21049	0,0014	0,191505
ENSG0000	0,001739	0,027248	3,209024	0,001407	0,191505
ENSG0000	-0,008689	0,239509	-3,201747	0,001442	0,191505
ENSG0000	0,005733	-0,213045	3,200676	0,001448	0,191505
ENSG0000	-0,005427	-0,575956	-3,197425	0,001464	0,191505
ENSG0000	-0,002531	0,124947	-3,197163	0,001465	0,191505
ENSG0000	0,002334	-0,197193	3,194932	0,001476	0,191505
ENSG0000	-0,003432	0,111042	-3,193525	0,001483	0,191505
ENSG0000	-0,004249	0,050594	-3,188997	0,001506	0,191505
ENSG0000	-0,002508	0,277298	-3,188956	0,001507	0,191505
ENSG0000	-0,005561	0,105027	-3,184865	0,001528	0,191505
ENSG0000	-0,014508	0,835174	-3,182732	0,001539	0,191505
ENSG0000	-0,014136	0,074299	-3,178882	0,001559	0,191505
ENSG0000	0,007834	0,50455	3,168683	0,001614	0,195315
ENSG0000	0,006492	-0,372629	3,161851	0,001651	0,196977
ENSG0000	0,003583	-0,741708	3,150175	0,001718	0,201952
ENSG0000	0,004393	0,485082	3,129439	0,001841	0,204295
ENSG0000	-0,007694	0,49806	-3,126293	0,001861	0,204295
ENSG0000	-0,008174	0,542021	-3,126001	0,001863	0,204295
ENSG0000	0,002552	0,579404	3,125777	0,001864	0,204295
ENSG0000	-0,005087	0,227692	-3,12343	0,001879	0,204295
ENSG0000	0,003465	0,414367	3,117624	0,001916	0,204295
ENSG0000	0,005877	-0,310347	3,116813	0,001921	0,204295
ENSG0000	0,004666	0,177596	3,114405	0,001936	0,204295
ENSG0000	-0,00164	0,104196	-3,104077	0,002004	0,208761
ENSG0000	0,004345	0,485488	3,092254	0,002084	0,214398
ENSG0000	0,004653	-0,667426	3,088028	0,002113	0,214734
ENSG0000	-0,00284	-0,158018	-3,076981	0,002192	0,21704
ENSG0000	-0,007072	-0,557783	-3,075979	0,002199	0,21704
ENSG0000	0,002415	0,087895	3,069439	0,002247	0,21704
ENSG0000	0,002924	-0,240881	3,068707	0,002253	0,21704
ENSG0000	0,006698	-0,103981	3,066636	0,002268	0,21704
ENSG0000	0,002588	-0,334873	3,056363	0,002346	0,219796
ENSG0000	0,001859	-0,92927	3,053361	0,002369	0,219796
ENSG0000	-0,007906	-0,894807	-3,052351	0,002377	0,219796
ENSG0000	0,004739	-0,674144	3,042621	0,002454	0,222081
ENSG0000	0,001792	0,418668	3,040948	0,002467	0,222081
ENSG0000	0,002905	0,204243	3,035954	0,002508	0,222081
ENSG0000	-0,003287	-1,081959	-3,031942	0,002541	0,222081
ENSG0000	0,002207	-0,27203	3,031426	0,002545	0,222081
ENSG0000	-0,005912	-0,859012	-3,026178	0,002589	0,222081
ENSG0000	0,002345	0,206523	3,022586	0,002619	0,222081

ENSG0000	0,004624	-0,546685	3,019034	0,00265	0,222081
ENSG0000	-0,00857	1,1582	-3,016767	0,002669	0,222081
ENSG0000	0,001694	0,726632	3,016531	0,002671	0,222081
ENSG0000	-0,008328	0,1387	-2,998133	0,002835	0,233354
ENSG0000	-0,002945	-1,271563	-2,99273	0,002885	0,233405
ENSG0000	-0,002989	-0,397295	-2,991928	0,002893	0,233405
ENSG0000	-0,004193	-0,373996	-2,98412	0,002966	0,237025
ENSG0000	0,003817	-0,507903	2,978167	0,003024	0,239281
ENSG0000	0,010567	-1,143267	2,972094	0,003083	0,240969
ENSG0000	-0,003913	0,622606	-2,970039	0,003104	0,240969
ENSG0000	-0,005589	-0,374939	-2,959921	0,003206	0,246573
ENSG0000	-0,00584	2,88052	-2,948764	0,003322	0,252544
ENSG0000	0,003289	0,155052	2,946615	0,003345	0,252544
ENSG0000	-0,004339	-0,242747	-2,943303	0,00338	0,2529
ENSG0000	-0,00511	-0,016957	-2,930979	0,003515	0,257748
ENSG0000	-0,008493	-1,485549	-2,929289	0,003534	0,257748
ENSG0000	0,001598	-0,193565	2,928846	0,003539	0,257748
ENSG0000	-0,003362	-0,19897	-2,923539	0,003599	0,258139
ENSG0000	-0,002815	-0,352912	-2,922826	0,003607	0,258139
ENSG0000	-0,003703	-0,024656	-2,904718	0,003819	0,261581
ENSG0000	-0,010131	-3,290338	-2,902784	0,003842	0,261581
ENSG0000	-0,003175	-0,534824	-2,900912	0,003865	0,261581
ENSG0000	0,001834	0,122823	2,895858	0,003927	0,261581
ENSG0000	-0,00426	0,726413	-2,894366	0,003945	0,261581
ENSG0000	-0,008796	0,494253	-2,890533	0,003993	0,261581
ENSG0000	-0,001327	-0,033016	-2,886251	0,004047	0,261581
ENSG0000	0,007573	0,331966	2,885147	0,004061	0,261581
ENSG0000	0,002792	-0,30218	2,885135	0,004061	0,261581
ENSG0000	0,003625	-0,028319	2,882428	0,004096	0,261581
ENSG0000	-0,003089	0,010898	-2,882019	0,004101	0,261581
ENSG0000	0,004203	0,397342	2,8802	0,004124	0,261581
ENSG0000	0,005734	-0,175443	2,877157	0,004164	0,261581
ENSG0000	0,003123	-0,247969	2,872971	0,004219	0,261581
ENSG0000	0,004078	-0,274157	2,870006	0,004258	0,261581
ENSG0000	0,007063	-0,046065	2,868754	0,004274	0,261581
ENSG0000	-0,008566	0,539095	-2,867771	0,004287	0,261581
ENSG0000	-0,004996	-0,250896	-2,867662	0,004289	0,261581
ENSG0000	0,003144	-1,162638	2,864976	0,004325	0,261581
ENSG0000	-0,002216	-0,025151	-2,863937	0,004339	0,261581
ENSG0000	0,002333	-0,789481	2,862966	0,004352	0,261581
ENSG0000	0,003905	-0,114089	2,862795	0,004354	0,261581
ENSG0000	-0,013636	1,057846	-2,853874	0,004477	0,26391
ENSG0000	0,002714	-0,159763	2,850917	0,004518	0,26391
ENSG0000	-0,012871	1,366264	-2,849551	0,004537	0,26391
ENSG0000	0,006787	-0,820561	2,845921	0,004588	0,26391
ENSG0000	0,004976	0,413722	2,844516	0,004608	0,26391
ENSG0000	0,003451	0,312778	2,843332	0,004625	0,26391
ENSG0000	-0,005763	-0,450836	-2,842152	0,004642	0,26391

ENSG0000	-0,00428	0,263174	-2,841634	0,00465	0,26391
ENSG0000	0,001812	-0,428063	2,839199	0,004685	0,264083
ENSG0000	0,010088	0,202672	2,834923	0,004747	0,264172
ENSG0000	-0,004184	0,518064	-2,834683	0,004751	0,264172
ENSG0000	-0,003448	-0,327325	-2,829789	0,004823	0,266102
ENSG0000	-0,000655	0,161339	-2,824149	0,004907	0,266102
ENSG0000	-0,002361	0,280517	-2,823683	0,004914	0,266102
ENSG0000	0,005509	-0,0028	2,822926	0,004926	0,266102
ENSG0000	0,007847	0,529351	2,81975	0,004974	0,266102
ENSG0000	0,005401	0,469988	2,816018	0,005031	0,266102
ENSG0000	-0,004269	0,412688	-2,812576	0,005085	0,266102
ENSG0000	-0,002131	0,033209	-2,812275	0,00509	0,266102
ENSG0000	0,003867	1,946199	2,811195	0,005106	0,266102
ENSG0000	-0,00957	0,458844	-2,81092	0,005111	0,266102
ENSG0000	0,004968	0,184888	2,805826	0,005191	0,266102
ENSG0000	-0,004839	-0,80146	-2,805148	0,005202	0,266102
ENSG0000	0,008872	0,728822	2,80424	0,005216	0,266102
ENSG0000	0,008403	1,400465	2,802886	0,005238	0,266102
ENSG0000	0,004831	-0,838055	2,797327	0,005328	0,268442
ENSG0000	-0,005717	-1,342855	-2,796002	0,005349	0,268442
ENSG0000	0,009502	1,046169	2,783378	0,005559	0,277274
ENSG0000	0,008342	0,503339	2,781079	0,005598	0,277523
ENSG0000	-0,000741	0,127798	-2,778219	0,005647	0,277523
ENSG0000	0,003604	-0,252919	2,777151	0,005665	0,277523
ENSG0000	0,006534	0,884605	2,77308	0,005735	0,279308
ENSG0000	0,002576	-0,086739	2,770665	0,005778	0,279623
ENSG0000	-0,001957	-0,258427	-2,76827	0,00582	0,279623
ENSG0000	0,003659	-1,431576	2,766517	0,005851	0,279623
ENSG0000	0,001944	-0,346342	2,761007	0,005949	0,279623
ENSG0000	-0,004705	-0,457902	-2,760676	0,005955	0,279623
ENSG0000	0,004192	1,073323	2,75988	0,005969	0,279623
ENSG0000	0,010931	0,604369	2,758113	0,006001	0,279623
ENSG0000	0,007506	-0,483109	2,754855	0,00606	0,279623
ENSG0000	0,001621	-0,124567	2,753169	0,006091	0,279623
ENSG0000	0,001323	0,352904	2,752344	0,006106	0,279623
ENSG0000	0,001871	0,57526	2,75184	0,006116	0,279623
ENSG0000	0,005335	0,666307	2,738721	0,006362	0,288551
ENSG0000	0,004036	-0,373657	2,73771	0,006381	0,288551
ENSG0000	-0,003427	0,197309	-2,731469	0,006502	0,292393
ENSG0000	-0,003396	1,213297	-2,725666	0,006615	0,295467
ENSG0000	-0,003181	1,154286	-2,72434	0,006642	0,295467
ENSG0000	0,007784	-0,72642	2,720483	0,006719	0,297283
ENSG0000	-0,005717	-0,313794	-2,718644	0,006756	0,29732
ENSG0000	0,003812	-0,572774	2,714451	0,006841	0,299459
ENSG0000	-0,005329	0,037376	-2,712149	0,006888	0,299924
ENSG0000	0,006238	-0,757109	2,709275	0,006947	0,300908
ENSG0000	-0,003692	0,115659	-2,705688	0,007021	0,302542
ENSG0000	-0,003008	-0,666637	-2,700831	0,007123	0,305339

ENSG0000	0,003209	-0,221796	2,69504	0,007247	0,309018
ENSG0000	-0,003311	0,295505	-2,690636	0,007342	0,309476
ENSG0000	-0,008256	1,348579	-2,687029	0,007421	0,309476
ENSG0000	0,002816	0,206533	2,68575	0,007449	0,309476
ENSG0000	0,000876	0,126948	2,684855	0,007468	0,309476
ENSG0000	0,006911	-0,056474	2,684711	0,007472	0,309476
ENSG0000	-0,009986	0,548245	-2,684189	0,007483	0,309476
ENSG0000	-0,005838	-0,782549	-2,682003	0,007532	0,309791
ENSG0000	0,007119	0,501064	2,680456	0,007566	0,309791
ENSG0000	0,009764	0,628941	2,675681	0,007673	0,312262
ENSG0000	0,004362	-0,748139	2,673813	0,007716	0,312262
ENSG0000	-0,006068	-0,619539	-2,67199	0,007757	0,312262
ENSG0000	-0,006217	0,312587	-2,67107	0,007778	0,312262
ENSG0000	-0,009241	1,063578	-2,664848	0,007922	0,315859
ENSG0000	-0,004368	0,542717	-2,663867	0,007944	0,315859
ENSG0000	-0,003605	-0,614908	-2,661602	0,007997	0,316436
ENSG0000	0,003808	-0,559963	2,658343	0,008074	0,316539
ENSG0000	-0,003068	0,403019	-2,657631	0,008091	0,316539
ENSG0000	-0,001365	-0,142578	-2,656605	0,008115	0,316539
ENSG0000	-0,005792	0,875752	-2,652109	0,008223	0,319219
ENSG0000	0,00266	-0,488538	2,649437	0,008287	0,320166
ENSG0000	-0,003663	0,037009	-2,645069	0,008394	0,320166
ENSG0000	0,00211	0,56171	2,644398	0,00841	0,320166
ENSG0000	-0,002299	0,685222	-2,643381	0,008435	0,320166
ENSG0000	-0,005115	0,434881	-2,643115	0,008442	0,320166
ENSG0000	0,002791	0,361719	2,641374	0,008485	0,32022
ENSG0000	-0,002474	-0,176718	-2,638562	0,008555	0,32022
ENSG0000	-0,00337	-0,556742	-2,638346	0,00856	0,32022
ENSG0000	-0,007464	0,847969	-2,633517	0,008681	0,323283
ENSG0000	-0,003174	0,318987	-2,631071	0,008743	0,324123
ENSG0000	0,009424	-2,638969	2,62569	0,008881	0,327752
ENSG0000	-0,011426	-0,697622	-2,622027	0,008976	0,329773
ENSG0000	0,00436	0,531841	2,620488	0,009016	0,329775
ENSG0000	-0,002171	-0,27836	-2,615503	0,009147	0,333088
ENSG0000	-0,003611	-1,142366	-2,613096	0,009211	0,333937
ENSG0000	0,003925	-0,528598	2,611074	0,009265	0,33442
ENSG0000	-0,005557	1,203337	-2,609428	0,009309	0,334547
ENSG0000	0,006603	-0,294748	2,605475	0,009416	0,336913
ENSG0000	0,006198	0,510407	2,602984	0,009483	0,337872
ENSG0000	-0,003592	-0,17186	-2,596876	0,009652	0,338241
ENSG0000	-0,004674	-0,143605	-2,595074	0,009702	0,338241
ENSG0000	0,002672	0,049533	2,593021	0,009759	0,338241
ENSG0000	0,006337	-0,598564	2,59281	0,009765	0,338241
ENSG0000	0,002826	0,220841	2,592417	0,009776	0,338241
ENSG0000	0,014214	1,400814	2,59237	0,009777	0,338241
ENSG0000	0,004795	-0,101778	2,590746	0,009823	0,338241
ENSG0000	0,001417	-0,140864	2,588528	0,009886	0,338241
ENSG0000	-0,001718	-0,767557	-2,58809	0,009898	0,338241

ENSG0000	0,003357	-0,238824	2,587864	0,009905	0,338241
ENSG0000	-0,006126	1,292249	-2,581313	0,010092	0,343226
ENSG0000	-0,003329	0,301454	-2,577866	0,010192	0,344444
ENSG0000	-0,004455	0,896439	-2,577198	0,010212	0,344444
ENSG0000	0,003469	-0,060623	2,574206	0,0103	0,345983
ENSG0000	-0,001636	0,237462	-2,566848	0,010518	0,351395
ENSG0000	0,003832	0,910168	2,565915	0,010546	0,351395
ENSG0000	-0,001102	-0,1349	-2,562141	0,01066	0,352471
ENSG0000	-0,002572	0,083694	-2,561121	0,010691	0,352471
ENSG0000	0,002256	-0,118821	2,5606	0,010707	0,352471
ENSG0000	-0,008675	-0,676282	-2,558227	0,010779	0,352523
ENSG0000	-0,006633	0,538415	-2,557746	0,010794	0,352523
ENSG0000	0,008326	1,884331	2,554269	0,010901	0,354107
ENSG0000	0,008869	0,722244	2,553384	0,010929	0,354107
ENSG0000	-0,004096	0,06193	-2,550982	0,011003	0,35416
ENSG0000	-0,002656	0,039323	-2,547954	0,011098	0,35416
ENSG0000	-0,005178	-0,82762	-2,547915	0,0111	0,35416
ENSG0000	0,002113	-0,10288	2,547821	0,011102	0,35416
ENSG0000	-0,006455	0,182546	-2,543542	0,011238	0,355937
ENSG0000	-0,004561	-0,421621	-2,542678	0,011265	0,355937
ENSG0000	-0,003127	0,45615	-2,540683	0,011329	0,355937
ENSG0000	0,002221	0,238738	2,540528	0,011334	0,355937
ENSG0000	-0,005852	0,136579	-2,538746	0,011391	0,355937
ENSG0000	-0,002726	-0,461271	-2,537925	0,011418	0,355937
ENSG0000	0,006559	-0,145859	2,535587	0,011493	0,356014
ENSG0000	0,002337	-0,347259	2,535175	0,011507	0,356014
ENSG0000	0,001556	0,235715	2,527565	0,011756	0,360356
ENSG0000	-0,005044	0,705767	-2,526667	0,011786	0,360356
ENSG0000	-0,002379	0,597788	-2,525547	0,011823	0,360356
ENSG0000	-0,003686	0,870206	-2,524687	0,011852	0,360356
ENSG0000	-0,002443	0,320981	-2,524263	0,011866	0,360356
ENSG0000	-0,004719	0,527475	-2,519423	0,012028	0,363947
ENSG0000	-0,004712	-0,031959	-2,517444	0,012095	0,364635
ENSG0000	-0,002081	0,331725	-2,508797	0,012392	0,372216
ENSG0000	0,002342	0,468771	2,504001	0,012559	0,373876
ENSG0000	0,002774	-0,092191	2,50174	0,012639	0,373876
ENSG0000	0,002747	0,198284	2,501703	0,01264	0,373876
ENSG0000	-0,003484	-0,019056	-2,501332	0,012653	0,373876
ENSG0000	-0,004009	-0,141576	-2,500736	0,012675	0,373876
ENSG0000	-0,004983	-0,566976	-2,496646	0,01282	0,375893
ENSG0000	-0,004952	0,711652	-2,496247	0,012834	0,375893
ENSG0000	-0,006226	-0,7169	-2,490967	0,013024	0,38011
ENSG0000	-0,002168	-0,017376	-2,484532	0,01326	0,385367
ENSG0000	0,00418	0,504482	2,481531	0,013371	0,385367
ENSG0000	0,008033	-0,289048	2,4806	0,013405	0,385367
ENSG0000	0,006476	0,937589	2,477113	0,013535	0,385367
ENSG0000	-0,004941	-0,883876	-2,477061	0,013537	0,385367
ENSG0000	-0,002202	-0,307106	-2,477061	0,013537	0,385367

ENSG0000	0,001517	-0,006382	2,47659	0,013555	0,385367
ENSG0000	-0,004315	-0,622391	-2,473544	0,01367	0,385367
ENSG0000	-0,00082	-0,087552	-2,473141	0,013685	0,385367
ENSG0000	0,00373	0,242365	2,47253	0,013708	0,385367
ENSG0000	0,002385	1,014447	2,472229	0,01372	0,385367
ENSG0000	-0,003162	-0,21798	-2,468124	0,013876	0,387411
ENSG0000	0,007209	0,680778	2,467856	0,013887	0,387411
ENSG0000	0,003009	-0,399065	2,465532	0,013976	0,388587
ENSG0000	-0,006395	0,243865	-2,463741	0,014045	0,389198
ENSG0000	-0,001932	0,092111	-2,461911	0,014116	0,389856
ENSG0000	0,001032	-0,11755	2,455163	0,014381	0,395843
ENSG0000	0,000536	-0,081555	2,452184	0,014499	0,397769
ENSG0000	0,003458	1,906077	2,450241	0,014577	0,398137
ENSG0000	-0,003457	0,264481	-2,448693	0,014639	0,398137
ENSG0000	-0,002871	-0,485025	-2,447979	0,014668	0,398137
ENSG0000	0,00381	-0,917507	2,446171	0,014741	0,398137
ENSG0000	-0,005678	1,184943	-2,445829	0,014755	0,398137
ENSG0000	-0,002758	0,806017	-2,441333	0,014938	0,400308
ENSG0000	-0,005304	0,828718	-2,440465	0,014973	0,400308
ENSG0000	0,010946	-1,336154	2,438196	0,015067	0,400308
ENSG0000	-0,002695	-0,199786	-2,437306	0,015103	0,400308
ENSG0000	-0,002885	-0,713533	-2,436836	0,015123	0,400308
ENSG0000	0,008842	1,120561	2,436091	0,015154	0,400308
ENSG0000	0,008131	-0,014193	2,434844	0,015205	0,400308
ENSG0000	0,004124	-1,516915	2,43439	0,015224	0,400308
ENSG0000	-0,005477	0,612503	-2,431083	0,015363	0,402353
ENSG0000	-0,004487	-0,085094	-2,430194	0,0154	0,402353
ENSG0000	0,006294	0,650731	2,428748	0,015461	0,402514
ENSG0000	-0,002222	-0,589252	-2,427728	0,015504	0,402514
ENSG0000	0,009338	-1,058269	2,423856	0,015668	0,405048
ENSG0000	-0,00491	-0,225885	-2,423118	0,0157	0,405048
ENSG0000	-0,003313	-0,238852	-2,417647	0,015935	0,409498
ENSG0000	0,003415	0,999651	2,415743	0,016018	0,409498
ENSG0000	0,004309	0,817477	2,414168	0,016087	0,409498
ENSG0000	-0,004581	-0,971974	-2,412407	0,016164	0,409498
ENSG0000	-0,004206	0,191283	-2,411912	0,016186	0,409498
ENSG0000	0,003064	0,423656	2,411163	0,016219	0,409498
ENSG0000	-0,002359	0,307377	-2,411115	0,016221	0,409498
ENSG0000	0,002002	0,122371	2,408448	0,016338	0,41054
ENSG0000	-0,002411	0,039495	-2,406949	0,016405	0,41054
ENSG0000	0,00545	-0,630297	2,405994	0,016447	0,41054
ENSG0000	0,00122	0,097566	2,405679	0,016461	0,41054
ENSG0000	0,004839	1,033241	2,389289	0,017207	0,427829
ENSG0000	-0,002071	-0,183541	-2,386046	0,017358	0,43014
ENSG0000	0,002411	-0,438118	2,384989	0,017407	0,43014
ENSG0000	0,001812	-0,063614	2,382727	0,017513	0,43014
ENSG0000	0,008459	-1,99588	2,382617	0,017519	0,43014
ENSG0000	-0,002849	-0,062181	-2,381642	0,017564	0,43014

ENSG0000	-0,004218	0,800224	-2,38061	0,017613	0,43014
ENSG0000	-0,001234	0,549326	-2,37658	0,017805	0,430533
ENSG0000	0,005086	-2,475461	2,376171	0,017824	0,430533
ENSG0000	0,0033	0,559058	2,374677	0,017896	0,430533
ENSG0000	-0,002829	0,573321	-2,373539	0,017951	0,430533
ENSG0000	-0,002016	0,266156	-2,369886	0,018127	0,430533
ENSG0000	0,006765	-2,089829	2,368862	0,018177	0,430533
ENSG0000	-0,001739	-0,049996	-2,368826	0,018179	0,430533
ENSG0000	0,007665	0,046273	2,368631	0,018188	0,430533
ENSG0000	0,003272	0,556181	2,367389	0,018249	0,430533
ENSG0000	-0,002071	-0,27664	-2,367159	0,01826	0,430533
ENSG0000	0,00324	-0,318501	2,367055	0,018265	0,430533
ENSG0000	0,005523	-0,459803	2,366309	0,018302	0,430533
ENSG0000	-0,003024	-0,640664	-2,364966	0,018367	0,430533
ENSG0000	-0,004667	1,13176	-2,364519	0,018389	0,430533
ENSG0000	0,005904	-0,00995	2,363261	0,018451	0,430533
ENSG0000	0,001241	-0,222272	2,362955	0,018466	0,430533
ENSG0000	0,003597	0,573963	2,358892	0,018668	0,433464
ENSG0000	0,002008	-0,088609	2,357516	0,018736	0,433464
ENSG0000	0,001234	-0,218249	2,356462	0,018789	0,433464
ENSG0000	-0,00736	0,204904	-2,356047	0,01881	0,433464
ENSG0000	-0,003185	-0,385715	-2,355139	0,018855	0,433464
ENSG0000	-0,006706	-0,606125	-2,353037	0,018961	0,434684
ENSG0000	-0,004463	1,182292	-2,345492	0,019346	0,441411
ENSG0000	0,00264	0,210186	2,343601	0,019443	0,441411
ENSG0000	0,00419	0,865272	2,341521	0,019551	0,441411
ENSG0000	-0,003763	-0,343744	-2,340105	0,019624	0,441411
ENSG0000	-0,003043	-0,342107	-2,338785	0,019693	0,441411
ENSG0000	-0,005682	0,909664	-2,337893	0,01974	0,441411
ENSG0000	0,002019	0,043884	2,335417	0,019869	0,441411
ENSG0000	-0,005283	0,541234	-2,33343	0,019974	0,441411
ENSG0000	-0,003733	-0,457491	-2,333111	0,019991	0,441411
ENSG0000	-0,003253	0,360044	-2,333005	0,019997	0,441411
ENSG0000	-0,001247	0,190503	-2,332407	0,020028	0,441411
ENSG0000	0,002774	0,196527	2,330771	0,020115	0,441411
ENSG0000	0,003618	-0,30376	2,329994	0,020156	0,441411
ENSG0000	0,008081	0,746216	2,329046	0,020207	0,441411
ENSG0000	-0,001796	0,073269	-2,327799	0,020274	0,441411
ENSG0000	-0,002296	0,262671	-2,327435	0,020293	0,441411
ENSG0000	0,003538	-0,315195	2,327395	0,020295	0,441411
ENSG0000	0,00411	0,319183	2,327309	0,0203	0,441411
ENSG0000	-0,002764	-0,085487	-2,326603	0,020338	0,441411
ENSG0000	-0,007303	0,329043	-2,326585	0,020339	0,441411
ENSG0000	-0,002735	0,064978	-2,325796	0,020381	0,441411
ENSG0000	0,007446	-1,149896	2,323451	0,020508	0,442985
ENSG0000	0,000784	-0,118759	2,318364	0,020784	0,443103
ENSG0000	0,003962	-0,04471	2,318344	0,020785	0,443103
ENSG0000	0,003217	-1,650038	2,318261	0,02079	0,443103

ENSG0000	0,00332	-0,33943	2,318039	0,020802	0,443103
ENSG0000	-0,00251	-0,434196	-2,316475	0,020888	0,443103
ENSG0000	0,002533	-0,11671	2,315107	0,020963	0,443103
ENSG0000	-0,001633	-0,206076	-2,313612	0,021046	0,443103
ENSG0000	0,004987	-0,552996	2,313162	0,021071	0,443103
ENSG0000	-0,004284	-0,519832	-2,312755	0,021093	0,443103
ENSG0000	0,002646	-0,664022	2,312588	0,021102	0,443103
ENSG0000	-0,002763	0,95376	-2,311577	0,021158	0,443103
ENSG0000	0,002511	-0,016288	2,311565	0,021159	0,443103
ENSG0000	0,003713	-0,625999	2,307845	0,021367	0,445364
ENSG0000	-0,002421	-0,569611	-2,306938	0,021418	0,445364
ENSG0000	0,004147	-0,433608	2,306324	0,021452	0,445364
ENSG0000	-0,002066	-0,137525	-2,301528	0,021723	0,445364
ENSG0000	0,001987	-0,141226	2,301392	0,021731	0,445364
ENSG0000	-0,007016	0,512005	-2,300801	0,021765	0,445364
ENSG0000	-0,00279	-0,258273	-2,299821	0,021821	0,445364
ENSG0000	-0,006401	-0,55457	-2,299422	0,021843	0,445364
ENSG0000	0,004458	-0,383617	2,298789	0,02188	0,445364
ENSG0000	0,006004	-2,961741	2,298363	0,021904	0,445364
ENSG0000	-0,0066	1,133759	-2,296105	0,022034	0,445364
ENSG0000	-0,002023	0,802868	-2,295774	0,022053	0,445364
ENSG0000	0,002903	0,45506	2,295292	0,022081	0,445364
ENSG0000	0,00851	0,389971	2,293841	0,022164	0,445364
ENSG0000	0,007	0,895707	2,292699	0,022231	0,445364
ENSG0000	0,006591	-0,782412	2,292565	0,022238	0,445364
ENSG0000	-0,005071	-0,762097	-2,290664	0,022349	0,445364
ENSG0000	-0,00235	0,292152	-2,290512	0,022358	0,445364
ENSG0000	0,008258	-1,196642	2,290155	0,022379	0,445364
ENSG0000	-0,003447	-0,903103	-2,287966	0,022507	0,445364
ENSG0000	-0,003792	0,413287	-2,287168	0,022553	0,445364
ENSG0000	-0,007521	2,244475	-2,286654	0,022584	0,445364
ENSG0000	-0,002571	-0,113925	-2,286309	0,022604	0,445364
ENSG0000	0,00577	0,005887	2,285516	0,022651	0,445364
ENSG0000	0,002312	0,611183	2,283098	0,022794	0,445364
ENSG0000	0,001296	-0,293707	2,282314	0,02284	0,445364
ENSG0000	-0,004468	0,473686	-2,282215	0,022846	0,445364
ENSG0000	-0,003601	-0,452741	-2,279555	0,023005	0,445364
ENSG0000	0,006067	-1,056707	2,279289	0,023021	0,445364
ENSG0000	-0,003386	-0,514614	-2,278609	0,023061	0,445364
ENSG0000	0,001122	-0,069553	2,278143	0,023089	0,445364
ENSG0000	0,002214	-0,5054	2,277779	0,023111	0,445364
ENSG0000	-0,00286	-0,028423	-2,277713	0,023115	0,445364
ENSG0000	0,004431	0,405895	2,277491	0,023128	0,445364
ENSG0000	0,002095	-0,396783	2,276716	0,023175	0,445364
ENSG0000	-0,005642	-0,68008	-2,274626	0,023301	0,445364
ENSG0000	-0,003172	0,902919	-2,2744	0,023315	0,445364
ENSG0000	-0,003266	-0,212805	-2,27376	0,023353	0,445364
ENSG0000	-0,003708	0,575163	-2,272981	0,023401	0,445364

ENSG0000	0,004551	0,891874	2,271839	0,02347	0,445364
ENSG0000	0,002646	0,537753	2,271166	0,023511	0,445364
ENSG0000	-0,001405	-0,118958	-2,269639	0,023604	0,445364
ENSG0000	-0,010225	-0,042049	-2,269282	0,023626	0,445364
ENSG0000	-0,006205	0,052135	-2,267666	0,023725	0,445364
ENSG0000	0,002115	-0,182649	2,267215	0,023753	0,445364
ENSG0000	-0,008624	0,627017	-2,267154	0,023756	0,445364
ENSG0000	0,006657	0,403801	2,265431	0,023863	0,446338
ENSG0000	0,006243	-1,171944	2,261511	0,024105	0,446666
ENSG0000	-0,004052	-0,187815	-2,261203	0,024125	0,446666
ENSG0000	-0,003049	0,178897	-2,260489	0,024169	0,446666
ENSG0000	-0,009605	1,657661	-2,260421	0,024173	0,446666
ENSG0000	0,001514	-0,327018	2,260383	0,024176	0,446666
ENSG0000	0,00322	-0,569486	2,259904	0,024206	0,446666
ENSG0000	0,005859	-0,955308	2,258536	0,024291	0,447165
ENSG0000	-0,004685	0,299623	-2,257737	0,024341	0,447165
ENSG0000	-0,007178	-0,321482	-2,255829	0,024462	0,44837
ENSG0000	0,001418	-0,223002	2,25372	0,024595	0,449216
ENSG0000	0,003327	-0,240318	2,253373	0,024617	0,449216
ENSG0000	-0,010939	-0,17478	-2,25007	0,024827	0,449432
ENSG0000	-0,003334	-1,203226	-2,250018	0,02483	0,449432
ENSG0000	-0,004047	-0,75102	-2,249793	0,024845	0,449432
ENSG0000	0,003551	0,772098	2,249755	0,024847	0,449432
ENSG0000	-0,002984	-0,422641	-2,24881	0,024908	0,449538
ENSG0000	-0,002054	-0,100876	-2,247576	0,024987	0,44998
ENSG0000	0,002144	-0,262402	2,246539	0,025053	0,450196
ENSG0000	-0,004079	-0,835578	-2,244139	0,025208	0,451912
ENSG0000	0,002235	0,328391	2,243361	0,025259	0,451912
ENSG0000	-0,005591	0,795065	-2,236383	0,025715	0,456812
ENSG0000	0,002977	0,641386	2,236183	0,025728	0,456812
ENSG0000	-0,002521	-0,110974	-2,235863	0,025749	0,456812
ENSG0000	0,002442	-0,550178	2,234525	0,025837	0,456812
ENSG0000	-0,003271	0,613722	-2,233684	0,025893	0,456812
ENSG0000	-0,006903	0,104451	-2,233571	0,0259	0,456812
ENSG0000	-0,004449	-0,261197	-2,233259	0,025921	0,456812
ENSG0000	0,00269	0,445927	2,231178	0,026059	0,458188
ENSG0000	-0,005259	-1,662925	-2,230411	0,026111	0,458188
ENSG0000	-0,004983	0,92059	-2,227291	0,026319	0,459916
ENSG0000	-0,003214	0,633247	-2,225943	0,02641	0,459916
ENSG0000	-0,007229	0,045492	-2,225315	0,026453	0,459916
ENSG0000	-0,005289	0,720858	-2,225157	0,026463	0,459916
ENSG0000	-0,002401	-0,118868	-2,224783	0,026488	0,459916
ENSG0000	-0,001466	-0,33786	-2,22382	0,026554	0,460076
ENSG0000	-0,009465	-4,258899	-2,222415	0,026649	0,460373
ENSG0000	0,003975	-0,013456	2,221919	0,026683	0,460373
ENSG0000	-0,002914	-0,702805	-2,220953	0,026748	0,460541
ENSG0000	-0,001806	0,178531	-2,217357	0,026994	0,461521
ENSG0000	-0,002737	-0,297907	-2,216925	0,027024	0,461521

ENSG0000	-0,001939	0,459578	-2,216313	0,027066	0,461521
ENSG0000	0,002166	-0,310834	2,2162	0,027074	0,461521
ENSG0000	0,004028	-0,35939	2,21603	0,027086	0,461521
ENSG0000	-0,00237	0,43324	-2,214552	0,027188	0,462262
ENSG0000	-0,006319	-0,889277	-2,212938	0,027299	0,462262
ENSG0000	-0,005197	-1,702851	-2,211533	0,027397	0,462262
ENSG0000	0,001648	0,005719	2,211517	0,027398	0,462262
ENSG0000	-0,004285	-0,081719	-2,211275	0,027415	0,462262
ENSG0000	-0,004055	0,514852	-2,210539	0,027466	0,462262
ENSG0000	0,002779	-0,231167	2,205514	0,027818	0,466994
ENSG0000	0,002311	-0,118048	2,204879	0,027863	0,466994
ENSG0000	-0,004289	-1,417939	-2,20411	0,027917	0,466994
ENSG0000	-0,002347	-0,062638	-2,202511	0,028031	0,467412
ENSG0000	-0,002404	0,130924	-2,202155	0,028056	0,467412
ENSG0000	0,003225	0,853703	2,198154	0,028341	0,469174
ENSG0000	0,005539	-0,413618	2,198127	0,028343	0,469174
ENSG0000	0,004796	-0,586953	2,197806	0,028366	0,469174
ENSG0000	0,003133	1,819688	2,197478	0,02839	0,469174
ENSG0000	-0,007106	-0,569767	-2,196128	0,028487	0,469833
ENSG0000	0,003964	0,426675	2,194275	0,02862	0,470553
ENSG0000	-0,004128	-2,047135	-2,193937	0,028645	0,470553
ENSG0000	0,003602	0,069813	2,192884	0,028721	0,470866
ENSG0000	0,00222	-0,132653	2,1911	0,028851	0,471779
ENSG0000	0,001302	-0,364746	2,190199	0,028916	0,471779
ENSG0000	0,002634	0,433922	2,189069	0,028999	0,471779
ENSG0000	0,002813	-0,7481	2,188968	0,029006	0,471779
ENSG0000	-0,001437	0,04342	-2,186381	0,029196	0,473686
ENSG0000	-0,003269	1,04484	-2,185493	0,029261	0,473686
ENSG0000	0,00475	0,268202	2,184399	0,029342	0,473686
ENSG0000	0,009893	0,32405	2,184237	0,029354	0,473686
ENSG0000	0,003334	1,676398	2,183039	0,029442	0,474186
ENSG0000	-0,002818	-0,410653	-2,182187	0,029505	0,474275
ENSG0000	-0,007098	-0,677061	-2,180423	0,029636	0,474728
ENSG0000	0,004603	0,548676	2,180256	0,029649	0,474728
ENSG0000	0,001467	-0,12821	2,179354	0,029716	0,474881
ENSG0000	-0,00578	0,067958	-2,17855	0,029776	0,474918
ENSG0000	0,003264	-0,081881	2,176357	0,02994	0,476615
ENSG0000	0,007199	-0,533976	2,175078	0,030037	0,477223
ENSG0000	0,007042	0,761958	2,17408	0,030112	0,477497
ENSG0000	-0,006538	1,373365	-2,172042	0,030266	0,478178
ENSG0000	0,001552	0,302089	2,170988	0,030346	0,478178
ENSG0000	0,002904	0,048138	2,170243	0,030403	0,478178
ENSG0000	-0,007318	-0,615579	-2,168942	0,030502	0,478178
ENSG0000	-0,002093	0,244155	-2,168622	0,030526	0,478178
ENSG0000	0,005042	0,862271	2,168154	0,030562	0,478178
ENSG0000	-0,003939	0,079443	-2,16747	0,030614	0,478178
ENSG0000	-0,003868	1,322034	-2,167401	0,03062	0,478178
ENSG0000	0,006238	1,091435	2,164772	0,030822	0,479075

ENSG0000	0,00288	0,200758	2,164495	0,030843	0,479075
ENSG0000	0,002224	0,047498	2,163741	0,030901	0,479075
ENSG0000	-0,004673	-1,309042	-2,163179	0,030944	0,479075
ENSG0000	0,000899	0,659437	2,162872	0,030968	0,479075
ENSG0000	0,001897	-0,120833	2,158867	0,031279	0,482979
ENSG0000	0,005465	0,552633	2,156796	0,031441	0,484569
ENSG0000	-0,00893	0,868453	-2,154415	0,031628	0,486541
ENSG0000	-0,004232	0,12198	-2,149979	0,031979	0,490014
ENSG0000	0,002707	-0,35265	2,149294	0,032033	0,490014
ENSG0000	-0,002494	-0,199441	-2,149293	0,032034	0,490014
ENSG0000	0,002165	0,614418	2,147169	0,032203	0,490014
ENSG0000	0,005223	-0,295416	2,145148	0,032365	0,490014
ENSG0000	0,002424	-0,64103	2,144182	0,032443	0,490014
ENSG0000	-0,003714	1,631888	-2,142925	0,032544	0,490014
ENSG0000	0,001268	-0,049006	2,142885	0,032547	0,490014
ENSG0000	-0,002578	-0,369058	-2,14219	0,032603	0,490014
ENSG0000	-0,000572	-0,085758	-2,141614	0,03265	0,490014
ENSG0000	0,00296	2,581049	2,141203	0,032683	0,490014
ENSG0000	-0,001873	0,063294	-2,140857	0,032711	0,490014
ENSG0000	-0,00317	-0,398316	-2,140682	0,032725	0,490014
ENSG0000	-0,00542	0,35087	-2,139979	0,032782	0,490014
ENSG0000	0,008225	-2,267258	2,139184	0,032847	0,490014
ENSG0000	-0,003421	0,478176	-2,138827	0,032876	0,490014
ENSG0000	0,002927	-0,226946	2,13797	0,032946	0,490014
ENSG0000	0,001949	-0,236334	2,137902	0,032951	0,490014
ENSG0000	-0,001786	-0,162328	-2,137156	0,033012	0,490014
ENSG0000	0,002345	0,079515	2,13676	0,033045	0,490014
ENSG0000	0,006314	0,908224	2,132438	0,0334	0,494388
ENSG0000	0,003048	0,6047	2,130095	0,033594	0,496364
ENSG0000	-0,004511	0,861092	-2,12844	0,033731	0,496668
ENSG0000	0,001367	0,069215	2,128058	0,033763	0,496668
ENSG0000	0,002904	0,446132	2,127362	0,033821	0,496668
ENSG0000	-0,002739	0,567077	-2,126428	0,033899	0,496668
ENSG0000	0,002709	0,291318	2,126224	0,033916	0,496668
ENSG0000	0,003724	0,390275	2,125156	0,034005	0,497092
ENSG0000	0,002172	-0,114875	2,124309	0,034076	0,497248
ENSG0000	-0,00285	-0,794406	-2,122734	0,034209	0,498296
ENSG0000	0,006675	1,358989	2,121704	0,034296	0,498679
ENSG0000	0,003577	0,063476	2,119127	0,034514	0,500966
ENSG0000	-0,002438	0,376106	-2,117113	0,034685	0,502038
ENSG0000	0,002735	0,378439	2,114915	0,034873	0,502038
ENSG0000	0,002627	0,009494	2,113773	0,034971	0,502038
ENSG0000	0,001554	0,172969	2,113635	0,034982	0,502038
ENSG0000	0,002322	-0,331415	2,113417	0,035001	0,502038
ENSG0000	0,003377	-0,549302	2,112763	0,035057	0,502038
ENSG0000	-0,001382	-0,6991	-2,112405	0,035088	0,502038
ENSG0000	0,00637	0,765732	2,111738	0,035146	0,502038
ENSG0000	0,003255	0,10131	2,110602	0,035244	0,502038

ENSG0000	-0,00239	0,367974	-2,110483	0,035254	0,502038
ENSG0000	0,00385	1,264293	2,11036	0,035265	0,502038
ENSG0000	-0,0055	1,274134	-2,109725	0,03532	0,502038
ENSG0000	0,005108	-0,655049	2,107879	0,03548	0,502857
ENSG0000	0,002139	0,450323	2,107617	0,035502	0,502857
ENSG0000	-0,004604	0,574171	-2,106949	0,03556	0,502857
ENSG0000	0,002252	0,501136	2,104644	0,035762	0,504834
ENSG0000	-0,004283	-0,383325	-2,101818	0,03601	0,507465
ENSG0000	0,004774	0,654781	2,100553	0,036121	0,508166
ENSG0000	0,001933	0,485757	2,099169	0,036243	0,509015
ENSG0000	-0,005371	-0,396537	-2,096391	0,03649	0,511605
ENSG0000	-0,003743	0,081998	-2,095363	0,036582	0,512017
ENSG0000	0,004901	0,749507	2,093897	0,036712	0,512196
ENSG0000	-0,00319	0,962945	-2,093827	0,036719	0,512196
ENSG0000	0,005044	-1,459673	2,092459	0,036841	0,512735
ENSG0000	-0,001879	0,147266	-2,090994	0,036973	0,512735
ENSG0000	-0,001542	0,255303	-2,090611	0,037007	0,512735
ENSG0000	-0,005518	-1,405946	-2,089613	0,037097	0,512735
ENSG0000	-0,002506	-0,208244	-2,089449	0,037112	0,512735
ENSG0000	0,007199	0,56683	2,089239	0,037131	0,512735
ENSG0000	0,003238	-0,077635	2,087014	0,037333	0,514559
ENSG0000	-0,004168	-0,089484	-2,085805	0,037443	0,514559
ENSG0000	-0,003627	0,063025	-2,08492	0,037523	0,514559
ENSG0000	0,00344	-0,217804	2,084698	0,037543	0,514559
ENSG0000	0,002195	0,531664	2,083736	0,037631	0,514559
ENSG0000	-0,004215	-0,307895	-2,083419	0,03766	0,514559
ENSG0000	0,00505	-0,857235	2,081437	0,037842	0,514559
ENSG0000	-0,000386	0,022952	-2,081304	0,037854	0,514559
ENSG0000	0,005283	-0,380893	2,080951	0,037887	0,514559
ENSG0000	0,00287	-0,436395	2,080404	0,037937	0,514559
ENSG0000	0,005371	0,431553	2,078921	0,038073	0,514559
ENSG0000	0,0066	-0,937468	2,078909	0,038075	0,514559
ENSG0000	0,004866	-1,151355	2,078775	0,038087	0,514559
ENSG0000	-0,003258	1,105269	-2,078209	0,038139	0,514559
ENSG0000	-0,004796	-0,862417	-2,077164	0,038236	0,514559
ENSG0000	-0,001425	0,657971	-2,075947	0,038349	0,514559
ENSG0000	0,002308	-0,79682	2,075078	0,03843	0,514559
ENSG0000	0,005071	0,761329	2,074762	0,038459	0,514559
ENSG0000	-0,001952	-0,340607	-2,073873	0,038542	0,514559
ENSG0000	-0,002078	0,908823	-2,073868	0,038542	0,514559
ENSG0000	-0,003535	0,642457	-2,073159	0,038608	0,514559
ENSG0000	-0,003726	-0,561213	-2,069691	0,038933	0,514559
ENSG0000	-0,002225	0,193118	-2,069537	0,038948	0,514559
ENSG0000	-0,004488	-0,091305	-2,068452	0,03905	0,514559
ENSG0000	-0,004157	0,979688	-2,068049	0,039088	0,514559
ENSG0000	0,007139	1,518012	2,067938	0,039099	0,514559
ENSG0000	-0,008229	-1,485992	-2,06791	0,039101	0,514559
ENSG0000	-0,004415	0,313777	-2,067843	0,039108	0,514559

ENSG0000	0,002226	0,158398	2,067467	0,039143	0,514559
ENSG0000	-0,002572	0,353458	-2,066399	0,039244	0,514559
ENSG0000	-0,002797	1,74265	-2,066333	0,03925	0,514559
ENSG0000	-0,000535	-0,088314	-2,065725	0,039308	0,514559
ENSG0000	0,002997	0,326796	2,06553	0,039327	0,514559
ENSG0000	0,002956	1,353878	2,063927	0,039479	0,514855
ENSG0000	0,001679	0,109086	2,063173	0,039551	0,514855
ENSG0000	0,000707	-0,06	2,062731	0,039593	0,514855
ENSG0000	0,001298	0,126824	2,062662	0,039599	0,514855
ENSG0000	0,002297	0,095808	2,061322	0,039728	0,515706
ENSG0000	0,001874	0,32371	2,059523	0,0399	0,516843
ENSG0000	0,005807	-0,617142	2,057693	0,040076	0,516843
ENSG0000	-0,002131	0,29988	-2,055782	0,040261	0,516843
ENSG0000	0,003304	0,178683	2,055253	0,040312	0,516843
ENSG0000	-0,004196	0,821765	-2,054451	0,04039	0,516843
ENSG0000	-0,003971	0,299867	-2,053932	0,04044	0,516843
ENSG0000	0,002177	0,454162	2,053068	0,040524	0,516843
ENSG0000	0,002244	-0,402741	2,052884	0,040542	0,516843
ENSG0000	0,007734	-0,805996	2,052823	0,040548	0,516843
ENSG0000	-0,001977	0,229421	-2,052606	0,040569	0,516843
ENSG0000	0,000342	-0,147542	2,051114	0,040715	0,516843
ENSG0000	-0,001752	0,195303	-2,051061	0,04072	0,516843
ENSG0000	0,00543	1,049015	2,05097	0,040729	0,516843
ENSG0000	-0,00407	-2,814412	-2,050705	0,040755	0,516843
ENSG0000	0,003619	0,249359	2,05068	0,040757	0,516843
ENSG0000	-0,003374	-0,455292	-2,049191	0,040903	0,517478
ENSG0000	-0,003867	-0,45673	-2,048885	0,040933	0,517478
ENSG0000	-0,00551	0,165294	-2,04724	0,041095	0,518726
ENSG0000	-0,006207	1,223268	-2,044535	0,041362	0,520967
ENSG0000	0,006851	-0,39444	2,044162	0,041399	0,520967
ENSG0000	-0,004198	-0,43249	-2,043471	0,041467	0,521032
ENSG0000	-0,001714	0,072116	-2,041248	0,041688	0,522565
ENSG0000	-0,003818	-0,32962	-2,040702	0,041743	0,522565
ENSG0000	-0,001077	0,156538	-2,039196	0,041894	0,522565
ENSG0000	-0,004258	-2,77E-05	-2,038499	0,041963	0,522565
ENSG0000	0,004171	-0,643092	2,037992	0,042014	0,522565
ENSG0000	0,004612	1,738808	2,037986	0,042015	0,522565
ENSG0000	0,001566	0,515681	2,03694	0,04212	0,522565
ENSG0000	-0,002717	-0,121522	-2,036153	0,042199	0,522565
ENSG0000	0,002972	-0,292593	2,035478	0,042267	0,522565
ENSG0000	0,005836	-1,687372	2,035295	0,042286	0,522565
ENSG0000	0,001127	0,135081	2,035273	0,042288	0,522565
ENSG0000	0,002622	0,352952	2,032274	0,042591	0,525526
ENSG0000	0,004142	0,416308	2,031646	0,042655	0,525526
ENSG0000	0,003082	0,972031	2,02894	0,042931	0,527882
ENSG0000	0,003447	0,523961	2,027721	0,043056	0,527882
ENSG0000	-0,001643	-0,215897	-2,027513	0,043077	0,527882
ENSG0000	0,002161	0,874815	2,027261	0,043103	0,527882

ENSG0000	0,000834	0,191924	2,025657	0,043268	0,528524
ENSG0000	0,003332	-1,022598	2,025129	0,043322	0,528524
ENSG0000	0,001911	0,072306	2,024611	0,043375	0,528524
ENSG0000	-0,004077	0,653362	-2,02255	0,043588	0,528524
ENSG0000	-0,001793	0,259315	-2,022305	0,043614	0,528524
ENSG0000	-0,003566	0,218732	-2,022244	0,04362	0,528524
ENSG0000	0,00116	-0,085775	2,021942	0,043651	0,528524
ENSG0000	0,001263	0,130517	2,021771	0,043669	0,528524
ENSG0000	0,002157	0,384732	2,019265	0,04393	0,528732
ENSG0000	0,004013	0,500724	2,019012	0,043956	0,528732
ENSG0000	-0,001545	0,274641	-2,01898	0,043959	0,528732
ENSG0000	-0,005778	1,284043	-2,018183	0,044042	0,528732
ENSG0000	0,002556	-0,240606	2,017912	0,044071	0,528732
ENSG0000	-0,003021	-0,032611	-2,017728	0,04409	0,528732
ENSG0000	0,003031	-0,427597	2,017088	0,044157	0,528732
ENSG0000	0,003499	0,411872	2,016547	0,044214	0,528732
ENSG0000	0,004249	-0,0968	2,016062	0,044264	0,528732
ENSG0000	0,004179	-1,973428	2,013684	0,044515	0,530948
ENSG0000	-0,006999	-1,808709	-2,010881	0,044811	0,53322
ENSG0000	0,002334	-0,116881	2,010656	0,044835	0,53322
ENSG0000	0,000954	0,025633	2,009196	0,04499	0,534293
ENSG0000	0,002433	0,777052	2,00806	0,045111	0,534957
ENSG0000	-0,002363	-0,217485	-2,006419	0,045286	0,535171
ENSG0000	0,001652	0,142329	2,004372	0,045505	0,535171
ENSG0000	0,001702	0,098041	2,003956	0,04555	0,535171
ENSG0000	0,004465	2,590318	2,003738	0,045573	0,535171
ENSG0000	0,003303	0,045566	2,00351	0,045598	0,535171
ENSG0000	0,001618	1,097962	2,003254	0,045625	0,535171
ENSG0000	-0,003993	0,604574	-2,002908	0,045663	0,535171
ENSG0000	-0,002946	-0,105225	-2,002725	0,045682	0,535171
ENSG0000	0,002231	-0,60724	2,002431	0,045714	0,535171
ENSG0000	-0,002965	-1,137551	-2,001241	0,045842	0,535461
ENSG0000	-0,001303	-0,935139	-2,000405	0,045933	0,535461
ENSG0000	0,006552	1,114841	2,000395	0,045934	0,535461
ENSG0000	-0,006431	0,273678	-1,998182	0,046174	0,536743
ENSG0000	0,008785	-2,103464	1,9981	0,046183	0,536743
ENSG0000	-0,002637	-0,183984	-1,997171	0,046284	0,536743
ENSG0000	-0,001351	0,436479	-1,996398	0,046368	0,536743
ENSG0000	0,0018	-0,199972	1,996223	0,046387	0,536743
ENSG0000	-0,001092	0,067952	-1,995785	0,046435	0,536743
ENSG0000	0,004245	0,463268	1,993474	0,046688	0,538196
ENSG0000	-0,003593	0,956988	-1,992976	0,046743	0,538196
ENSG0000	0,008081	1,218072	1,992847	0,046757	0,538196
ENSG0000	0,000894	0,00612	1,991561	0,046898	0,538268
ENSG0000	-0,002622	0,231594	-1,991037	0,046956	0,538268
ENSG0000	0,003898	-1,179993	1,990471	0,047019	0,538268
ENSG0000	-0,001655	-0,151066	-1,990054	0,047065	0,538268
ENSG0000	0,007459	0,511967	1,989729	0,047101	0,538268

ENSG0000	0,003142	-0,240409	1,98923	0,047156	0,538268
ENSG0000	0,002799	0,069693	1,987848	0,047309	0,538853
ENSG0000	0,004008	-1,343007	1,987586	0,047338	0,538853
ENSG0000	-0,009327	-2,520039	-1,986463	0,047463	0,539373
ENSG0000	-0,002526	-1,342545	-1,984682	0,047661	0,539373
ENSG0000	-0,00271	0,008391	-1,984609	0,047669	0,539373
ENSG0000	0,00469	1,58112	1,984509	0,047681	0,539373
ENSG0000	-0,003665	-0,343966	-1,983994	0,047738	0,539373
ENSG0000	-0,003675	-0,816663	-1,983166	0,047831	0,539373
ENSG0000	0,003927	0,0985	1,982056	0,047955	0,539373
ENSG0000	-0,001692	-0,368407	-1,981605	0,048006	0,539373
ENSG0000	-0,004095	-0,200568	-1,980794	0,048097	0,539373
ENSG0000	-0,004061	0,62013	-1,980153	0,048169	0,539373
ENSG0000	-0,00382	0,806123	-1,980101	0,048175	0,539373
ENSG0000	-0,003862	0,671696	-1,980044	0,048181	0,539373
ENSG0000	-0,002204	0,121866	-1,979365	0,048258	0,539373
ENSG0000	-0,001936	-0,098412	-1,978855	0,048316	0,539373
ENSG0000	-0,002611	-0,42552	-1,978403	0,048367	0,539373
ENSG0000	-0,002616	-0,324152	-1,976413	0,048592	0,540511
ENSG0000	-0,002395	-0,112259	-1,975518	0,048694	0,540511
ENSG0000	-0,005956	-1,405255	-1,975258	0,048723	0,540511
ENSG0000	0,004644	-0,848019	1,975187	0,048731	0,540511
ENSG0000	-0,003347	-0,486116	-1,973889	0,048879	0,541399
ENSG0000	0,001164	0,241588	1,972566	0,04903	0,541399
ENSG0000	0,002531	0,844399	1,972532	0,049034	0,541399
ENSG0000	-0,005385	0,186898	-1,971806	0,049117	0,541399
ENSG0000	-0,003601	-0,143378	-1,971605	0,04914	0,541399
ENSG0000	0,001706	-0,225702	1,970986	0,049211	0,541455
ENSG0000	0,001639	0,302592	1,970032	0,049321	0,541936
ENSG0000	0,005229	0,242696	1,967904	0,049566	0,543253
ENSG0000	-0,001107	-0,577544	-1,967845	0,049573	0,543253
ENSG0000	0,005	1,310446	1,966153	0,049768	0,544671
ENSG0000	-0,006324	-0,866322	-1,965533	0,04984	0,544733

geneID	logFC	AveExpr	t	P.Value	adj.P.Val
ENSG0000	0,004342	-0,619127	5,072113	5,33E-07	0,003495
ENSG0000	-0,0089	-0,360946	-4,639783	4,33E-06	0,014199
ENSG0000	-0,00313	0,08766	-4,332015	1,75E-05	0,038162
ENSG0000	-0,010361	-0,774147	-4,204822	3,03E-05	0,049725
ENSG0000	0,002935	0,156175	4,105329	4,63E-05	0,05594
ENSG0000	0,005579	0,393023	4,081233	5,12E-05	0,05594
ENSG0000	-0,012044	3,780198	-4,023093	6,52E-05	0,06105
ENSG0000	-0,00616	-0,284431	-3,943702	9,02E-05	0,073941
ENSG0000	0,010074	-0,03363	3,907999	0,000104	0,075937
ENSG0000	0,002895	0,033496	3,813926	0,000152	0,086064
ENSG0000	-0,003828	0,303037	-3,80209	0,000159	0,086064
ENSG0000	-0,008856	0,59717	-3,798814	0,000161	0,086064
ENSG0000	0,006	-0,296173	3,770861	0,00018	0,086064
ENSG0000	0,009087	-0,623329	3,765054	0,000184	0,086064
ENSG0000	-0,001832	-0,01297	-3,726589	0,000213	0,093283
ENSG0000	-0,008507	0,069019	-3,707302	0,00023	0,094215
ENSG0000	0,0081	0,949492	3,67364	0,000262	0,097761
ENSG0000	0,002279	0,662493	3,658321	0,000277	0,097761
ENSG0000	0,00415	-1,549446	3,652102	0,000284	0,097761
ENSG0000	-0,004756	-0,942853	-3,627724	0,000312	0,097761
ENSG0000	0,002469	0,280148	3,61493	0,000327	0,097761
ENSG0000	0,008244	1,429056	3,61409	0,000328	0,097761
ENSG0000	0,006001	-1,70614	3,588726	0,000361	0,10287
ENSG0000	0,007619	0,007741	3,539328	0,000434	0,118517
ENSG0000	-0,007415	-0,208425	-3,507405	0,000488	0,124063
ENSG0000	-0,004199	0,26474	-3,493466	0,000514	0,124063
ENSG0000	-0,00531	-0,871922	-3,491652	0,000517	0,124063
ENSG0000	0,002565	0,160928	3,480235	0,000539	0,124063
ENSG0000	-0,013626	1,758868	-3,475444	0,000549	0,124063
ENSG0000	0,016597	-0,382554	3,44576	0,000611	0,133601
ENSG0000	0,004698	-0,164184	3,418224	0,000675	0,142811
ENSG0000	0,006272	-0,276785	3,386246	0,000757	0,151526
ENSG0000	-0,007768	0,196527	-3,381029	0,000772	0,151526
ENSG0000	-0,004107	0,0995	-3,357953	0,000838	0,151526
ENSG0000	-0,004028	-0,282039	-3,346528	0,000872	0,151526
ENSG0000	-0,002564	0,321932	-3,341217	0,000889	0,151526
ENSG0000	0,006743	1,314	3,33314	0,000914	0,151526
ENSG0000	-0,004361	-0,411546	-3,332336	0,000917	0,151526
ENSG0000	-0,009714	-0,96392	-3,331776	0,000919	0,151526
ENSG0000	-0,001853	0,135801	-3,33001	0,000925	0,151526
ENSG0000	0,007066	-0,116262	3,316115	0,000971	0,153066
ENSG0000	-0,006535	-0,668499	-3,31084	0,000989	0,153066
ENSG0000	0,011926	-0,489427	3,300633	0,001025	0,153066
ENSG0000	0,006949	-0,535859	3,292729	0,001054	0,153066
ENSG0000	0,001859	0,124879	3,291534	0,001058	0,153066
ENSG0000	0,002758	-0,117323	3,280567	0,001099	0,153066
ENSG0000	0,002263	0,170621	3,280068	0,001101	0,153066
ENSG0000	-0,004379	-0,329511	-3,269492	0,001142	0,153066

ENSG0000	-0,003447	-0,129173	-3,269054	0,001144	0,153066
ENSG0000	-0,003156	-0,013846	-3,230222	0,001308	0,168993
ENSG0000	-0,008578	0,304021	-3,228776	0,001315	0,168993
ENSG0000	0,007721	-0,325398	3,213785	0,001384	0,171699
ENSG0000	-0,004954	0,973415	-3,212909	0,001388	0,171699
ENSG0000	0,008031	-0,817614	3,198674	0,001457	0,176909
ENSG0000	-0,006229	0,40371	-3,185223	0,001526	0,180566
ENSG0000	-0,009528	0,565706	-3,178513	0,001561	0,180566
ENSG0000	0,002124	0,159968	3,176733	0,00157	0,180566
ENSG0000	0,009335	-0,246368	3,171494	0,001598	0,180627
ENSG0000	-0,002017	-0,254984	-3,161411	0,001654	0,181854
ENSG0000	0,00221	0,329778	3,159452	0,001665	0,181854
ENSG0000	0,004856	-0,354204	3,143023	0,001759	0,189038
ENSG0000	-0,005835	0,328648	-3,129889	0,001838	0,192033
ENSG0000	0,002719	0,221083	3,127864	0,001851	0,192033
ENSG0000	-0,005805	1,244688	-3,123995	0,001875	0,192033
ENSG0000	-0,004264	0,027727	-3,100798	0,002026	0,19297
ENSG0000	-0,00387	0,509039	-3,100777	0,002026	0,19297
ENSG0000	-0,002217	0,135775	-3,098326	0,002042	0,19297
ENSG0000	-0,004603	0,308074	-3,098199	0,002043	0,19297
ENSG0000	-0,007006	0,382139	-3,098072	0,002044	0,19297
ENSG0000	-0,010752	-0,175079	-3,093106	0,002078	0,19297
ENSG0000	-0,002788	0,242328	-3,091326	0,00209	0,19297
ENSG0000	0,001919	-0,085386	3,07828	0,002182	0,198676
ENSG0000	0,005104	0,832465	3,065069	0,002279	0,201718
ENSG0000	-0,003009	-0,409106	-3,061052	0,00231	0,201718
ENSG0000	-0,004947	-0,43876	-3,06046	0,002314	0,201718
ENSG0000	-0,005415	3,703992	-3,055305	0,002354	0,201718
ENSG0000	-0,005817	-0,541498	-3,053249	0,00237	0,201718
ENSG0000	0,002443	0,042343	3,04598	0,002427	0,203929
ENSG0000	-0,005357	-0,004432	-3,034983	0,002515	0,208713
ENSG0000	0,004434	-0,6775	3,028621	0,002568	0,210423
ENSG0000	-0,006848	-0,926015	-3,020695	0,002635	0,213253
ENSG0000	-0,015735	0,586731	-3,015914	0,002676	0,213453
ENSG0000	0,01015	-0,431056	3,012892	0,002703	0,213453
ENSG0000	0,003316	-0,27036	3,005343	0,00277	0,216133
ENSG0000	-0,003565	0,594139	-2,99337	0,002879	0,218567
ENSG0000	0,002797	0,235764	2,992227	0,00289	0,218567
ENSG0000	-0,005051	1,140049	-2,986496	0,002943	0,218567
ENSG0000	-0,003158	-0,986804	-2,983999	0,002967	0,218567
ENSG0000	-0,00501	-1,467504	-2,983953	0,002968	0,218567
ENSG0000	0,006662	0,371734	2,9784	0,003021	0,220031
ENSG0000	-0,004003	-0,371452	-2,961658	0,003188	0,227936
ENSG0000	-0,009578	0,544205	-2,960526	0,003199	0,227936
ENSG0000	-0,006636	-1,020748	-2,942317	0,00339	0,238965
ENSG0000	-0,004226	0,007877	-2,934717	0,003473	0,239995
ENSG0000	-0,004807	0,491974	-2,928775	0,003539	0,239995
ENSG0000	0,002006	-0,543857	2,927718	0,003551	0,239995

ENSG0000	-0,006559	0,594532	-2,927694	0,003551	0,239995
ENSG0000	-0,013571	0,677681	-2,912754	0,003723	0,249033
ENSG0000	0,002237	-0,563278	2,908116	0,003778	0,250148
ENSG0000	-0,004754	0,605235	-2,899813	0,003878	0,25281
ENSG0000	-0,013026	1,033472	-2,898395	0,003895	0,25281
ENSG0000	0,000849	0,7327	2,884369	0,00407	0,261586
ENSG0000	0,002854	0,28019	2,880731	0,004117	0,26201
ENSG0000	0,003796	0,473776	2,87615	0,004176	0,262895
ENSG0000	0,005543	-0,737714	2,873493	0,004211	0,262895
ENSG0000	-0,000808	0,136252	-2,854033	0,004474	0,276669
ENSG0000	0,00718	0,748179	2,849125	0,004543	0,27687
ENSG0000	-0,003202	0,026621	-2,84777	0,004562	0,27687
ENSG0000	0,00567	-0,526978	2,84233	0,004639	0,278987
ENSG0000	-0,009514	0,049278	-2,833176	0,004772	0,284377
ENSG0000	0,003941	0,554251	2,821651	0,004945	0,284604
ENSG0000	-0,001809	-0,412035	-2,818795	0,004988	0,284604
ENSG0000	-0,00716	0,505778	-2,816373	0,005025	0,284604
ENSG0000	0,010734	0,567156	2,813489	0,00507	0,284604
ENSG0000	0,001291	0,146082	2,809546	0,005132	0,284604
ENSG0000	-0,009389	1,42455	-2,808136	0,005154	0,284604
ENSG0000	0,006776	0,568866	2,805915	0,005189	0,284604
ENSG0000	0,004998	0,128004	2,801398	0,005261	0,284604
ENSG0000	0,002578	0,583502	2,800062	0,005283	0,284604
ENSG0000	-0,003668	-0,79799	-2,798565	0,005307	0,284604
ENSG0000	0,010091	0,99389	2,797504	0,005324	0,284604
ENSG0000	-0,010955	0,695313	-2,796831	0,005335	0,284604
ENSG0000	0,00246	-0,283698	2,796509	0,00534	0,284604
ENSG0000	-0,004677	1,272623	-2,791131	0,005429	0,285765
ENSG0000	0,007197	-1,026391	2,788151	0,005478	0,285765
ENSG0000	0,004522	1,702555	2,787266	0,005493	0,285765
ENSG0000	-0,003937	0,057102	-2,784203	0,005544	0,28617
ENSG0000	0,003389	0,854787	2,77724	0,005663	0,289904
ENSG0000	0,005665	0,100468	2,774056	0,005718	0,289904
ENSG0000	0,003677	0,346479	2,768105	0,005822	0,289904
ENSG0000	0,003423	0,455649	2,762903	0,005914	0,289904
ENSG0000	-0,009691	1,119758	-2,758854	0,005987	0,289904
ENSG0000	-0,00113	0,200223	-2,757301	0,006015	0,289904
ENSG0000	0,005881	0,631345	2,752045	0,006111	0,289904
ENSG0000	0,006647	-0,677998	2,750945	0,006132	0,289904
ENSG0000	0,001949	-0,076456	2,750885	0,006133	0,289904
ENSG0000	0,004992	1,182078	2,747162	0,006202	0,289904
ENSG0000	-0,006011	0,447273	-2,741264	0,006313	0,289904
ENSG0000	0,011721	-0,227135	2,740652	0,006324	0,289904
ENSG0000	0,006845	0,473411	2,739689	0,006343	0,289904
ENSG0000	-0,006441	-0,449837	-2,73961	0,006344	0,289904
ENSG0000	-0,002163	-0,025407	-2,737855	0,006378	0,289904
ENSG0000	-0,000907	-0,309717	-2,73743	0,006386	0,289904
ENSG0000	-0,00204	-0,394513	-2,735747	0,006418	0,289904

ENSG0000	-0,001956	-0,120155	-2,731642	0,006498	0,289904
ENSG0000	-0,003971	-0,058592	-2,729401	0,006541	0,289904
ENSG0000	0,002986	0,708603	2,72456	0,006637	0,289904
ENSG0000	0,00454	0,565658	2,724099	0,006646	0,289904
ENSG0000	0,002539	0,005462	2,71978	0,006732	0,289904
ENSG0000	0,002933	0,325843	2,719434	0,006739	0,289904
ENSG0000	-0,018609	-4,02181	-2,717732	0,006773	0,289904
ENSG0000	-0,003852	0,478318	-2,715839	0,006812	0,289904
ENSG0000	0,002075	0,236563	2,714335	0,006842	0,289904
ENSG0000	0,002109	-0,432218	2,713952	0,00685	0,289904
ENSG0000	0,003683	0,393231	2,713707	0,006855	0,289904
ENSG0000	-0,005324	-0,902015	-2,704067	0,007055	0,294868
ENSG0000	0,006406	-1,450848	2,70369	0,007062	0,294868
ENSG0000	0,003248	0,173025	2,699772	0,007145	0,295531
ENSG0000	-0,002256	0,149433	-2,698172	0,007179	0,295531
ENSG0000	-0,010648	1,179806	-2,695409	0,007238	0,295531
ENSG0000	0,00209	0,39178	2,693737	0,007274	0,295531
ENSG0000	0,006839	-0,022547	2,69236	0,007304	0,295531
ENSG0000	0,001932	0,076503	2,69015	0,007352	0,295644
ENSG0000	0,002732	1,457992	2,687624	0,007407	0,296045
ENSG0000	0,008442	-0,603375	2,681353	0,007545	0,29768
ENSG0000	-0,004492	0,601406	-2,681107	0,007551	0,29768
ENSG0000	0,004304	-0,272646	2,679419	0,007588	0,29768
ENSG0000	-0,006882	-0,650071	-2,677595	0,007629	0,29768
ENSG0000	-0,006055	0,78012	-2,673727	0,007717	0,299309
ENSG0000	-0,002433	0,13257	-2,670329	0,007794	0,300539
ENSG0000	-0,004511	1,269634	-2,666115	0,007891	0,302504
ENSG0000	-0,004086	0,456336	-2,660352	0,008026	0,305243
ENSG0000	-0,003115	0,383016	-2,659081	0,008056	0,305243
ENSG0000	-0,004292	-0,376235	-2,652957	0,008202	0,307271
ENSG0000	-0,003692	-0,675899	-2,652894	0,008203	0,307271
ENSG0000	-0,004067	-1,325858	-2,648461	0,00831	0,307428
ENSG0000	-0,002662	-0,549373	-2,647822	0,008326	0,307428
ENSG0000	-0,004193	-0,886348	-2,645085	0,008393	0,307428
ENSG0000	-0,002211	-0,067555	-2,643724	0,008426	0,307428
ENSG0000	0,007011	0,615145	2,640918	0,008495	0,307428
ENSG0000	-0,003287	0,338645	-2,63825	0,008562	0,307428
ENSG0000	-0,008482	-0,062682	-2,636321	0,00861	0,307428
ENSG0000	0,004231	1,744317	2,634025	0,008667	0,307428
ENSG0000	0,00405	-0,41675	2,631199	0,008739	0,307428
ENSG0000	-0,007577	0,381337	-2,629099	0,008792	0,307428
ENSG0000	0,0027	-0,219898	2,628008	0,00882	0,307428
ENSG0000	0,005347	-0,750308	2,625246	0,008891	0,307428
ENSG0000	0,003976	0,478466	2,623554	0,008935	0,307428
ENSG0000	0,002069	0,539265	2,623136	0,008946	0,307428
ENSG0000	-0,005703	0,042404	-2,621284	0,008994	0,307428
ENSG0000	0,006716	0,767119	2,620165	0,009023	0,307428
ENSG0000	0,001821	-0,039671	2,619605	0,009038	0,307428

ENSG0000	0,002863	-0,087308	2,61608	0,009131	0,307428
ENSG0000	-0,001905	-0,202558	-2,613458	0,0092	0,307428
ENSG0000	-0,002465	-0,163922	-2,612392	0,009229	0,307428
ENSG0000	-0,010725	-1,162805	-2,612275	0,009232	0,307428
ENSG0000	0,001145	-0,100588	2,610645	0,009275	0,307428
ENSG0000	-0,000681	-0,095617	-2,610239	0,009286	0,307428
ENSG0000	-0,004552	-0,078158	-2,607403	0,009362	0,308397
ENSG0000	-0,004193	0,329876	-2,603676	0,009464	0,308968
ENSG0000	0,004017	-0,189192	2,602042	0,009508	0,308968
ENSG0000	0,002956	0,071664	2,601573	0,009521	0,308968
ENSG0000	-0,000932	0,063698	-2,597141	0,009643	0,311393
ENSG0000	-0,003795	-0,318593	-2,594194	0,009725	0,311635
ENSG0000	-0,001321	-0,145899	-2,592395	0,009776	0,311635
ENSG0000	-0,00411	-0,380595	-2,589987	0,009844	0,311635
ENSG0000	-0,0069	0,659788	-2,589193	0,009866	0,311635
ENSG0000	-0,001775	0,115285	-2,587346	0,009919	0,311635
ENSG0000	-0,003885	0,15646	-2,586729	0,009936	0,311635
ENSG0000	0,004145	0,002153	2,581845	0,010076	0,313049
ENSG0000	-0,002365	0,366445	-2,581824	0,010077	0,313049
ENSG0000	-0,007005	-0,426771	-2,571696	0,010373	0,320139
ENSG0000	-0,005275	0,311112	-2,570018	0,010423	0,320139
ENSG0000	-0,002536	-0,318015	-2,569046	0,010452	0,320139
ENSG0000	-0,00878	0,390187	-2,564641	0,010584	0,320712
ENSG0000	0,003448	-0,123324	2,564525	0,010587	0,320712
ENSG0000	-0,007003	-1,105629	-2,561511	0,010678	0,320712
ENSG0000	-0,005201	-0,0974	-2,561187	0,010688	0,320712
ENSG0000	0,000671	0,063561	2,558792	0,010761	0,320712
ENSG0000	0,004309	0,131357	2,558707	0,010764	0,320712
ENSG0000	-0,001765	0,400631	-2,555492	0,010863	0,322191
ENSG0000	0,007433	0,309893	2,549756	0,011041	0,325457
ENSG0000	-0,005218	-1,795105	-2,547476	0,011112	0,325457
ENSG0000	0,004639	0,807732	2,547085	0,011125	0,325457
ENSG0000	-0,006722	0,349966	-2,545373	0,011179	0,325457
ENSG0000	-0,004448	1,871426	-2,542241	0,011278	0,325457
ENSG0000	0,003202	-0,34748	2,541506	0,011302	0,325457
ENSG0000	0,004622	-1,947147	2,540928	0,01132	0,325457
ENSG0000	0,002887	-0,429526	2,53651	0,011462	0,328106
ENSG0000	0,001279	-0,044066	2,533008	0,011576	0,329924
ENSG0000	-0,003723	-0,338266	-2,529129	0,011704	0,332107
ENSG0000	0,004985	-0,290145	2,521402	0,011961	0,333771
ENSG0000	0,003681	-0,431919	2,520129	0,012004	0,333771
ENSG0000	0,001863	0,494663	2,51936	0,01203	0,333771
ENSG0000	-0,003684	-0,993636	-2,518696	0,012052	0,333771
ENSG0000	-0,004429	0,675285	-2,518581	0,012056	0,333771
ENSG0000	-0,004106	-0,256418	-2,51761	0,012089	0,333771
ENSG0000	0,007046	-1,027076	2,516733	0,012119	0,333771
ENSG0000	-0,00477	0,801332	-2,513873	0,012216	0,335051
ENSG0000	-0,001983	-0,431949	-2,510653	0,012327	0,336679

ENSG0000	0,008307	0,414375	2,507607	0,012432	0,338154
ENSG0000	0,00374	-0,087825	2,506107	0,012485	0,338172
ENSG0000	-0,004933	-0,856883	-2,504246	0,01255	0,338537
ENSG0000	-0,007272	1,862805	-2,494916	0,012881	0,344048
ENSG0000	-0,004277	0,048338	-2,494191	0,012907	0,344048
ENSG0000	0,002417	0,099687	2,492902	0,012953	0,344048
ENSG0000	-0,002459	-0,180051	-2,489232	0,013086	0,344048
ENSG0000	-0,00261	-0,117738	-2,487338	0,013156	0,344048
ENSG0000	-0,004272	0,340374	-2,486484	0,013187	0,344048
ENSG0000	-0,004787	0,364701	-2,486004	0,013204	0,344048
ENSG0000	0,002846	-1,343907	2,481509	0,01337	0,344048
ENSG0000	0,004898	-0,194728	2,481304	0,013378	0,344048
ENSG0000	0,002415	0,420679	2,480641	0,013403	0,344048
ENSG0000	-0,003643	1,115786	-2,479683	0,013438	0,344048
ENSG0000	0,0034	1,254717	2,478678	0,013476	0,344048
ENSG0000	0,003372	1,483233	2,477973	0,013502	0,344048
ENSG0000	0,005412	-1,5017	2,475624	0,01359	0,344048
ENSG0000	-0,006878	-1,742444	-2,47533	0,013601	0,344048
ENSG0000	0,0066	-0,448777	2,473882	0,013656	0,344048
ENSG0000	-0,007627	1,211025	-2,468156	0,013874	0,344048
ENSG0000	0,002433	-0,09812	2,465946	0,013959	0,344048
ENSG0000	-0,00852	0,737476	-2,463373	0,014058	0,344048
ENSG0000	0,003297	-0,940512	2,463278	0,014062	0,344048
ENSG0000	0,003556	-1,19834	2,461125	0,014146	0,344048
ENSG0000	0,002931	-0,812166	2,460768	0,01416	0,344048
ENSG0000	0,005865	0,283351	2,45982	0,014197	0,344048
ENSG0000	0,004012	-0,36836	2,459518	0,014209	0,344048
ENSG0000	0,00257	0,662669	2,459498	0,014209	0,344048
ENSG0000	0,003833	0,758932	2,458443	0,014251	0,344048
ENSG0000	-0,00127	0,347356	-2,457017	0,014307	0,344048
ENSG0000	-0,004097	-0,06806	-2,456486	0,014328	0,344048
ENSG0000	0,00329	-0,317667	2,456127	0,014342	0,344048
ENSG0000	0,006162	-0,660059	2,454974	0,014388	0,344048
ENSG0000	0,001769	0,009745	2,454675	0,014399	0,344048
ENSG0000	0,002685	0,146229	2,453413	0,01445	0,344048
ENSG0000	-0,003856	-0,931298	-2,45249	0,014486	0,344048
ENSG0000	-0,006811	-2,40548	-2,448391	0,01465	0,345911
ENSG0000	0,001937	0,477612	2,447897	0,01467	0,345911
ENSG0000	-0,004215	-0,809921	-2,445953	0,014749	0,346115
ENSG0000	-0,00366	0,299051	-2,44507	0,014784	0,346115
ENSG0000	-0,004212	0,148131	-2,442248	0,014899	0,346776
ENSG0000	0,001401	0,261471	2,441779	0,014918	0,346776
ENSG0000	0,002644	-0,070216	2,438915	0,015036	0,348272
ENSG0000	-0,004635	0,542307	-2,433005	0,015281	0,350874
ENSG0000	0,004151	0,836887	2,431591	0,01534	0,350874
ENSG0000	0,002264	0,14902	2,43102	0,015364	0,350874
ENSG0000	0,002211	-0,532052	2,430972	0,015366	0,350874
ENSG0000	0,005272	-0,093719	2,429785	0,015416	0,350874

ENSG0000	0,001833	-0,302803	2,422301	0,015734	0,356868
ENSG0000	-0,007786	0,493352	-2,420335	0,015818	0,357547
ENSG0000	0,004269	0,219647	2,417184	0,015954	0,359386
ENSG0000	0,003668	0,27866	2,414971	0,016051	0,360315
ENSG0000	-0,003757	0,437026	-2,412283	0,016168	0,361716
ENSG0000	-0,00527	-0,368853	-2,409629	0,016285	0,363091
ENSG0000	0,005698	0,940892	2,403998	0,016535	0,366283
ENSG0000	0,003622	0,071048	2,403897	0,01654	0,366283
ENSG0000	-0,001896	0,190877	-2,402016	0,016624	0,366913
ENSG0000	-0,004346	-0,672349	-2,396653	0,016867	0,371021
ENSG0000	-0,003564	0,059338	-2,394063	0,016986	0,372376
ENSG0000	-0,005485	0,491768	-2,392118	0,017075	0,373086
ENSG0000	-0,006089	0,773069	-2,390384	0,017155	0,373327
ENSG0000	0,006012	0,824168	2,389115	0,017214	0,373327
ENSG0000	0,00199	0,206528	2,387365	0,017295	0,373327
ENSG0000	0,013906	1,133838	2,386963	0,017314	0,373327
ENSG0000	-0,001761	0,244309	-2,382138	0,01754	0,376965
ENSG0000	0,002985	0,109348	2,380019	0,01764	0,377224
ENSG0000	-0,002221	0,20953	-2,376434	0,017811	0,377224
ENSG0000	0,002929	-0,218122	2,376092	0,017827	0,377224
ENSG0000	-0,00586	0,408599	-2,376044	0,017829	0,377224
ENSG0000	-0,005951	-0,065047	-2,374194	0,017918	0,377224
ENSG0000	0,003316	0,692688	2,374141	0,017921	0,377224
ENSG0000	-0,002941	-0,154291	-2,37071	0,018086	0,377224
ENSG0000	-0,002642	-0,009312	-2,369164	0,018161	0,377224
ENSG0000	0,003597	-0,935195	2,366997	0,018267	0,377224
ENSG0000	-0,004381	-0,998873	-2,36669	0,018282	0,377224
ENSG0000	0,002356	0,230147	2,366226	0,018304	0,377224
ENSG0000	0,009013	0,058167	2,366161	0,018308	0,377224
ENSG0000	-0,008243	0,209943	-2,365894	0,018321	0,377224
ENSG0000	-0,002342	0,141604	-2,36514	0,018358	0,377224
ENSG0000	0,003801	-0,279356	2,359123	0,018655	0,382137
ENSG0000	0,002681	-0,772769	2,356718	0,018775	0,383398
ENSG0000	-0,001818	-0,240017	-2,352677	0,018978	0,384692
ENSG0000	-0,002185	-0,19958	-2,351757	0,019025	0,384692
ENSG0000	0,004031	0,303498	2,349942	0,019117	0,384692
ENSG0000	0,009193	-0,369233	2,349791	0,019125	0,384692
ENSG0000	0,004267	-1,088443	2,349648	0,019132	0,384692
ENSG0000	0,009433	1,422408	2,348139	0,019209	0,384797
ENSG0000	0,002199	-0,39394	2,347244	0,019255	0,384797
ENSG0000	-0,002635	0,643091	-2,344778	0,019381	0,385021
ENSG0000	0,004123	0,398394	2,344737	0,019383	0,385021
ENSG0000	0,006254	0,75537	2,342602	0,019493	0,385879
ENSG0000	0,006862	1,458167	2,341158	0,019568	0,385879
ENSG0000	0,003286	-0,335599	2,340488	0,019603	0,385879
ENSG0000	-0,00773	0,240013	-2,335233	0,019878	0,389602
ENSG0000	-0,009503	-0,249172	-2,334603	0,019911	0,389602
ENSG0000	0,00293	-0,400368	2,330862	0,020109	0,391161

ENSG0000	0,004174	0,42973	2,330843	0,02011	0,391161
ENSG0000	-0,002787	-0,5341	-2,327879	0,020268	0,393069
ENSG0000	-0,002652	0,351588	-2,324039	0,020475	0,393928
ENSG0000	0,004728	-0,086301	2,324024	0,020475	0,393928
ENSG0000	0,009492	0,162695	2,32153	0,02061	0,393928
ENSG0000	0,002938	0,41212	2,320365	0,020674	0,393928
ENSG0000	0,006115	-1,761277	2,320274	0,020679	0,393928
ENSG0000	-0,005643	1,176441	-2,317844	0,020811	0,393928
ENSG0000	0,010607	1,752684	2,317761	0,020816	0,393928
ENSG0000	0,002733	0,045914	2,317402	0,020836	0,393928
ENSG0000	-0,00635	-0,621609	-2,317082	0,020853	0,393928
ENSG0000	-0,00619	0,289305	-2,309325	0,021283	0,400886
ENSG0000	0,002909	0,553694	2,306964	0,021415	0,40115
ENSG0000	-0,002429	1,500439	-2,306889	0,021419	0,40115
ENSG0000	-0,008129	-0,290662	-2,298978	0,021868	0,40672
ENSG0000	0,002323	0,013726	2,297615	0,021946	0,40672
ENSG0000	-0,004872	0,748766	-2,296658	0,022001	0,40672
ENSG0000	-0,004623	-0,898661	-2,296567	0,022006	0,40672
ENSG0000	0,005309	-0,912474	2,295573	0,022063	0,40672
ENSG0000	-0,003335	0,714443	-2,295125	0,022089	0,40672
ENSG0000	-0,001446	-0,370217	-2,292172	0,02226	0,406967
ENSG0000	-0,004941	-1,6277	-2,290426	0,022361	0,406967
ENSG0000	-0,003569	0,762207	-2,289456	0,022418	0,406967
ENSG0000	-0,008055	-0,234463	-2,28849	0,022475	0,406967
ENSG0000	0,005804	-1,072713	2,287483	0,022534	0,406967
ENSG0000	0,001177	0,110391	2,286738	0,022577	0,406967
ENSG0000	-0,004749	1,185957	-2,284132	0,022731	0,406967
ENSG0000	-0,003398	-1,234389	-2,284066	0,022735	0,406967
ENSG0000	-0,005569	-0,23769	-2,283363	0,022777	0,406967
ENSG0000	0,002074	0,108088	2,282668	0,022818	0,406967
ENSG0000	-0,00503	0,763636	-2,281968	0,022859	0,406967
ENSG0000	0,007228	-1,43057	2,281369	0,022895	0,406967
ENSG0000	0,008615	1,232937	2,278958	0,023039	0,406967
ENSG0000	0,003793	-0,64586	2,277636	0,023118	0,406967
ENSG0000	0,004432	0,21856	2,277601	0,02312	0,406967
ENSG0000	0,001709	-0,209811	2,277565	0,023123	0,406967
ENSG0000	0,000988	0,123122	2,276979	0,023158	0,406967
ENSG0000	-0,00734	-0,405471	-2,273554	0,023365	0,409504
ENSG0000	-0,007854	-0,840683	-2,269071	0,023637	0,412155
ENSG0000	-0,002829	-0,176413	-2,267889	0,02371	0,412155
ENSG0000	-0,00697	-1,090666	-2,267327	0,023744	0,412155
ENSG0000	0,003436	0,012104	2,265463	0,023859	0,412155
ENSG0000	-0,001275	-0,364543	-2,265442	0,023861	0,412155
ENSG0000	0,005673	0,33491	2,263969	0,023952	0,412155
ENSG0000	0,00221	-0,750046	2,263527	0,023979	0,412155
ENSG0000	-0,001284	0,35255	-2,262339	0,024053	0,412155
ENSG0000	0,005487	0,519578	2,261872	0,024082	0,412155
ENSG0000	-0,003546	0,91548	-2,260344	0,024177	0,412707

ENSG0000	-0,004619	1,278485	-2,256799	0,024399	0,41366
ENSG0000	-0,005783	1,347006	-2,254631	0,024536	0,41366
ENSG0000	0,004653	-0,789655	2,253545	0,024605	0,41366
ENSG0000	0,001644	-0,10219	2,253127	0,024631	0,41366
ENSG0000	0,003275	-0,229265	2,25199	0,024703	0,41366
ENSG0000	-0,007386	-0,093094	-2,250663	0,024788	0,41366
ENSG0000	-0,001053	-0,082322	-2,250112	0,024823	0,41366
ENSG0000	0,008055	-2,527901	2,248561	0,024922	0,41366
ENSG0000	-0,003343	-0,271499	-2,247521	0,024989	0,41366
ENSG0000	-0,006345	-0,695651	-2,246861	0,025031	0,41366
ENSG0000	-0,002885	0,280057	-2,243911	0,025222	0,41366
ENSG0000	0,003711	-0,26588	2,24336	0,025257	0,41366
ENSG0000	-0,002643	-0,650833	-2,243212	0,025267	0,41366
ENSG0000	0,00232	0,063734	2,242935	0,025285	0,41366
ENSG0000	-0,00533	0,657123	-2,24203	0,025344	0,41366
ENSG0000	0,003529	-0,250046	2,241986	0,025347	0,41366
ENSG0000	-0,001648	0,224321	-2,241124	0,025403	0,41366
ENSG0000	0,002605	1,371145	2,240862	0,02542	0,41366
ENSG0000	-0,005961	0,446805	-2,24068	0,025432	0,41366
ENSG0000	0,004346	-0,451738	2,23739	0,025647	0,41481
ENSG0000	-0,008616	1,000361	-2,236256	0,025722	0,41481
ENSG0000	-0,006872	0,508421	-2,236032	0,025736	0,41481
ENSG0000	-0,003274	-0,414249	-2,235742	0,025756	0,41481
ENSG0000	0,004174	0,300284	2,22961	0,026163	0,419643
ENSG0000	0,00261	0,930214	2,228037	0,026268	0,419643
ENSG0000	-0,003228	0,879026	-2,22751	0,026303	0,419643
ENSG0000	-0,004815	-0,488796	-2,227386	0,026312	0,419643
ENSG0000	-0,001868	-0,278684	-2,225907	0,026411	0,420208
ENSG0000	-0,005526	0,125958	-2,222327	0,026653	0,422629
ENSG0000	-0,002705	-0,105172	-2,220671	0,026766	0,422629
ENSG0000	0,002469	-0,368711	2,220128	0,026803	0,422629
ENSG0000	0,001756	-0,72379	2,219863	0,026821	0,422629
ENSG0000	0,007448	0,063025	2,217718	0,026968	0,423023
ENSG0000	0,003344	-0,300944	2,216381	0,02706	0,423023
ENSG0000	0,003333	0,413486	2,216046	0,027083	0,423023
ENSG0000	0,001944	-0,705204	2,215348	0,027131	0,423023
ENSG0000	0,005561	-0,046146	2,214801	0,027169	0,423023
ENSG0000	0,004365	-0,381546	2,211809	0,027376	0,425241
ENSG0000	0,001216	-0,079308	2,208062	0,027638	0,428289
ENSG0000	0,003879	-0,898126	2,20701	0,027712	0,42842
ENSG0000	-0,002951	-0,086916	-2,205814	0,027796	0,428512
ENSG0000	0,00246	0,441882	2,205069	0,027848	0,428512
ENSG0000	0,011584	-1,612981	2,203923	0,027929	0,428752
ENSG0000	-0,004619	-1,195141	-2,202226	0,02805	0,429226
ENSG0000	-0,002105	0,156286	-2,20164	0,028091	0,429226
ENSG0000	-0,001238	-0,048286	-2,199752	0,028226	0,430141
ENSG0000	0,004596	1,105012	2,19896	0,028282	0,430141
ENSG0000	-0,002039	0,023269	-2,197188	0,028409	0,430691

ENSG0000	-0,006418	-1,078906	-2,196517	0,028457	0,430691
ENSG0000	0,003395	-0,663648	2,195709	0,028516	0,430691
ENSG0000	-0,003751	-0,798957	-2,194552	0,028599	0,430958
ENSG0000	-0,005465	0,818835	-2,192545	0,028744	0,432153
ENSG0000	0,008885	1,191826	2,189097	0,028995	0,433454
ENSG0000	-0,004168	0,154803	-2,188706	0,029024	0,433454
ENSG0000	0,00368	0,517995	2,186831	0,029161	0,433454
ENSG0000	0,003147	-0,217607	2,186634	0,029176	0,433454
ENSG0000	0,001921	-0,218684	2,186056	0,029218	0,433454
ENSG0000	0,003807	-0,774994	2,185928	0,029228	0,433454
ENSG0000	0,001571	0,263699	2,180995	0,029592	0,437722
ENSG0000	0,002761	0,380151	2,180236	0,029649	0,437722
ENSG0000	0,003154	-1,114112	2,174768	0,030059	0,440278
ENSG0000	0,008287	-0,213653	2,174332	0,030091	0,440278
ENSG0000	0,003293	0,186514	2,173893	0,030125	0,440278
ENSG0000	-0,003301	0,342935	-2,173708	0,030139	0,440278
ENSG0000	0,001491	0,482583	2,17258	0,030224	0,440278
ENSG0000	0,002341	-0,381595	2,172564	0,030225	0,440278
ENSG0000	0,003355	-0,114715	2,171467	0,030308	0,440511
ENSG0000	0,001957	0,929468	2,169055	0,030492	0,442199
ENSG0000	-0,002522	0,616539	-2,167292	0,030627	0,443049
ENSG0000	0,002566	0,174164	2,165479	0,030766	0,443049
ENSG0000	0,003286	0,336997	2,164533	0,030839	0,443049
ENSG0000	0,001024	-0,330758	2,16445	0,030845	0,443049
ENSG0000	-0,007423	-0,446593	-2,163886	0,030888	0,443049
ENSG0000	0,004234	0,474528	2,162419	0,031002	0,443704
ENSG0000	0,00562	0,081783	2,160992	0,031112	0,444317
ENSG0000	-0,001567	0,097117	-2,160031	0,031187	0,444415
ENSG0000	0,001839	-0,658676	2,157461	0,031388	0,445748
ENSG0000	-0,002968	0,813359	-2,1565	0,031463	0,445748
ENSG0000	0,002617	-0,787406	2,156223	0,031485	0,445748
ENSG0000	-0,005002	0,250962	-2,155178	0,031567	0,445946
ENSG0000	0,007562	0,282355	2,152257	0,031797	0,447407
ENSG0000	-0,001293	0,278387	-2,151806	0,031833	0,447407
ENSG0000	-0,000632	-0,084551	-2,150762	0,031915	0,447407
ENSG0000	-0,004346	0,097789	-2,150328	0,03195	0,447407
ENSG0000	-0,005611	0,162152	-2,149555	0,032011	0,447407
ENSG0000	0,002147	0,242651	2,144912	0,032382	0,451214
ENSG0000	-0,002964	0,239458	-2,144429	0,032421	0,451214
ENSG0000	-0,001632	0,195072	-2,143457	0,0325	0,451345
ENSG0000	0,003013	0,512439	2,141746	0,032638	0,451583
ENSG0000	-0,002163	0,714955	-2,140769	0,032717	0,451583
ENSG0000	-0,007686	0,799323	-2,140689	0,032723	0,451583
ENSG0000	0,007652	0,14576	2,138898	0,032869	0,452637
ENSG0000	0,00517	0,46942	2,135913	0,033113	0,454171
ENSG0000	0,002651	-1,014094	2,135837	0,033119	0,454171
ENSG0000	-0,002484	-1,147938	-2,130349	0,033571	0,458558
ENSG0000	-0,000912	-0,102575	-2,129182	0,033668	0,458558

ENSG0000	0,003384	-0,232556	2,127795	0,033783	0,458558
ENSG0000	0,002637	0,277442	2,127268	0,033827	0,458558
ENSG0000	-0,004173	-0,761269	-2,127029	0,033847	0,458558
ENSG0000	0,002501	0,308129	2,126893	0,033858	0,458558
ENSG0000	0,004224	1,815846	2,125121	0,034007	0,459615
ENSG0000	-0,007882	2,33791	-2,123523	0,034141	0,460479
ENSG0000	0,004409	-2,186426	2,120869	0,034365	0,462516
ENSG0000	-0,005506	-1,017507	-2,120061	0,034433	0,462516
ENSG0000	0,006875	0,518548	2,118422	0,034572	0,462622
ENSG0000	0,004383	-0,570622	2,117059	0,034688	0,462622
ENSG0000	0,002829	-0,010437	2,116813	0,034709	0,462622
ENSG0000	0,001598	0,37602	2,115246	0,034843	0,462622
ENSG0000	0,000618	-0,001581	2,11519	0,034848	0,462622
ENSG0000	0,006386	0,092845	2,114996	0,034864	0,462622
ENSG0000	-0,001075	0,072565	-2,109141	0,035369	0,467405
ENSG0000	-0,005191	0,556937	-2,108509	0,035423	0,467405
ENSG0000	-0,001672	-0,086505	-2,108073	0,035461	0,467405
ENSG0000	-0,003751	-0,063358	-2,107513	0,03551	0,467405
ENSG0000	0,003587	0,886029	2,106113	0,035632	0,468069
ENSG0000	-0,003482	-0,430724	-2,104069	0,03581	0,468879
ENSG0000	0,002784	1,047518	2,10347	0,035863	0,468879
ENSG0000	-0,002845	-0,422041	-2,101613	0,036026	0,468879
ENSG0000	0,005969	0,79854	2,100891	0,03609	0,468879
ENSG0000	-0,004862	-2,277135	-2,099548	0,036208	0,468879
ENSG0000	0,00245	0,07639	2,099511	0,036212	0,468879
ENSG0000	-0,005793	-0,942796	-2,099125	0,036246	0,468879
ENSG0000	0,002688	-0,547391	2,0989	0,036266	0,468879
ENSG0000	-0,002935	-1,582421	-2,098058	0,03634	0,468919
ENSG0000	-0,005226	1,063578	-2,096768	0,036455	0,469263
ENSG0000	-0,002518	-1,269738	-2,096147	0,03651	0,469263
ENSG0000	0,007103	-0,707215	2,095015	0,036611	0,46957
ENSG0000	-0,001374	-0,010965	-2,093867	0,036714	0,46957
ENSG0000	0,003523	-0,779389	2,092356	0,036849	0,46957
ENSG0000	0,003098	0,018245	2,091905	0,03689	0,46957
ENSG0000	-0,003383	-0,229528	-2,091875	0,036892	0,46957
ENSG0000	0,00166	0,810205	2,085332	0,037484	0,476179
ENSG0000	-0,006013	0,17983	-2,084388	0,03757	0,47635
ENSG0000	-0,004345	0,006853	-2,0832	0,037679	0,476758
ENSG0000	-0,00175	-0,479531	-2,082445	0,037748	0,476758
ENSG0000	-0,004468	-0,138614	-2,078868	0,038077	0,476837
ENSG0000	-0,001673	0,748979	-2,078105	0,038147	0,476837
ENSG0000	0,005171	0,924198	2,078099	0,038148	0,476837
ENSG0000	-0,001865	-0,175991	-2,077855	0,03817	0,476837
ENSG0000	-0,001705	-0,319718	-2,076464	0,038299	0,476837
ENSG0000	0,004432	-0,724878	2,076094	0,038334	0,476837
ENSG0000	0,003126	-1,068736	2,07542	0,038396	0,476837
ENSG0000	-0,003608	0,349554	-2,073638	0,038562	0,476837
ENSG0000	0,002837	-0,161775	2,073612	0,038565	0,476837

ENSG0000	-0,004921	0,602176	-2,07223	0,038694	0,476837
ENSG0000	0,00817	0,854636	2,071648	0,038748	0,476837
ENSG0000	-0,004494	0,676659	-2,071115	0,038798	0,476837
ENSG0000	-0,002372	0,511234	-2,071026	0,038806	0,476837
ENSG0000	-0,001942	-0,212847	-2,070624	0,038844	0,476837
ENSG0000	0,002044	-0,233211	2,070612	0,038845	0,476837
ENSG0000	0,003834	0,620969	2,068357	0,039058	0,477793
ENSG0000	0,003992	1,629701	2,068236	0,039069	0,477793
ENSG0000	-0,000698	-0,12607	-2,067324	0,039155	0,477954
ENSG0000	-0,004434	0,935602	-2,066085	0,039272	0,478496
ENSG0000	0,004325	-0,606142	2,064923	0,039383	0,478948
ENSG0000	0,005199	0,583557	2,064035	0,039467	0,478979
ENSG0000	0,002422	0,427732	2,061951	0,039666	0,478979
ENSG0000	0,004141	0,554283	2,061637	0,039696	0,478979
ENSG0000	-0,001311	-0,861199	-2,06149	0,03971	0,478979
ENSG0000	-0,006686	-0,978315	-2,05937	0,039913	0,478979
ENSG0000	0,001434	0,252376	2,059293	0,03992	0,478979
ENSG0000	0,002394	0,05789	2,058601	0,039987	0,478979
ENSG0000	-0,003862	-0,061114	-2,057921	0,040053	0,478979
ENSG0000	0,00141	-0,362	2,057463	0,040097	0,478979
ENSG0000	0,006089	0,625679	2,057264	0,040116	0,478979
ENSG0000	-0,003812	1,476854	-2,056275	0,040211	0,479247
ENSG0000	0,003656	-0,388435	2,054074	0,040425	0,480915
ENSG0000	-0,004162	-0,23272	-2,052168	0,04061	0,482114
ENSG0000	0,007859	1,861563	2,051166	0,040708	0,482114
ENSG0000	-0,004226	0,372517	-2,050173	0,040805	0,482114
ENSG0000	-0,003158	-1,416141	-2,049534	0,040868	0,482114
ENSG0000	0,00392	0,706699	2,049274	0,040893	0,482114
ENSG0000	-0,008147	0,318679	-2,047963	0,041022	0,482763
ENSG0000	0,00399	0,537092	2,045259	0,041289	0,484813
ENSG0000	-0,004743	-0,720741	-2,044698	0,041344	0,484813
ENSG0000	0,002903	-0,111047	2,040785	0,041733	0,488122
ENSG0000	0,004748	1,716786	2,040224	0,041789	0,488122
ENSG0000	-0,002157	-0,435816	-2,039618	0,04185	0,488122
ENSG0000	-0,00261	-0,953932	-2,036921	0,04212	0,490404
ENSG0000	0,002754	-0,615083	2,033668	0,042448	0,493349
ENSG0000	0,00485	0,495442	2,031586	0,04266	0,494176
ENSG0000	0,002189	-0,20339	2,031482	0,04267	0,494176
ENSG0000	0,002796	-0,316578	2,029702	0,042852	0,4954
ENSG0000	-0,003638	0,494733	-2,02667	0,043162	0,49811
ENSG0000	0,002927	-0,042503	2,019634	0,04389	0,504798
ENSG0000	0,003262	0,03953	2,019577	0,043895	0,504798
ENSG0000	-0,00348	-0,976781	-2,018347	0,044024	0,505386
ENSG0000	0,001775	-0,51728	2,016275	0,044241	0,506262
ENSG0000	-0,000623	-0,050643	-2,016141	0,044255	0,506262
ENSG0000	0,002411	-0,014811	2,013733	0,044508	0,506578
ENSG0000	-0,005902	1,35365	-2,012267	0,044662	0,506578
ENSG0000	0,003443	-0,822778	2,012222	0,044667	0,506578

ENSG0000	-0,001625	-0,217656	-2,01145	0,044749	0,506578
ENSG0000	0,004605	-0,903711	2,01045	0,044855	0,506578
ENSG0000	-0,001547	0,03506	-2,009501	0,044956	0,506578
ENSG0000	-0,002664	-0,983971	-2,009479	0,044958	0,506578
ENSG0000	0,002722	-0,146924	2,008536	0,045058	0,506578
ENSG0000	-0,003257	-0,471792	-2,007865	0,04513	0,506578
ENSG0000	0,003601	0,305449	2,007342	0,045186	0,506578
ENSG0000	0,001066	-0,184848	2,006868	0,045236	0,506578
ENSG0000	0,005028	0,000816	2,006075	0,045321	0,506578
ENSG0000	0,003332	0,179132	2,005568	0,045375	0,506578
ENSG0000	-0,003054	-0,211774	-2,005051	0,045431	0,506578
ENSG0000	0,001976	0,347823	2,004952	0,045441	0,506578
ENSG0000	0,003803	-1,000919	2,003857	0,045559	0,507025
ENSG0000	0,00264	-0,391552	2,002488	0,045706	0,507052
ENSG0000	-0,001573	0,240191	-2,001852	0,045775	0,507052
ENSG0000	-0,005205	0,859798	-2,001176	0,045848	0,507052
ENSG0000	-0,002319	-0,547066	-2,000965	0,045871	0,507052
ENSG0000	-0,005122	-0,161412	-1,999946	0,045981	0,507414
ENSG0000	-0,002081	-0,217143	-1,998906	0,046094	0,507433
ENSG0000	0,001534	0,259294	1,998504	0,046137	0,507433
ENSG0000	-0,002234	0,366919	-1,994796	0,046542	0,509833
ENSG0000	0,002368	0,522145	1,993424	0,046692	0,509833
ENSG0000	0,003153	0,249932	1,993105	0,046727	0,509833
ENSG0000	0,001945	-0,112608	1,99291	0,046748	0,509833
ENSG0000	-0,004002	0,595821	-1,992688	0,046773	0,509833
ENSG0000	-0,005294	0,059313	-1,991519	0,046901	0,509833
ENSG0000	0,003757	-1,203148	1,990979	0,046961	0,509833
ENSG0000	0,00444	0,01227	1,990826	0,046978	0,509833
ENSG0000	-0,00638	-0,285882	-1,989432	0,047132	0,510181
ENSG0000	0,003839	1,894588	1,989127	0,047165	0,510181
ENSG0000	0,006845	0,927364	1,988239	0,047264	0,510283
ENSG0000	-0,002864	-0,146537	-1,987099	0,04739	0,510283
ENSG0000	0,002461	-0,350198	1,986937	0,047408	0,510283
ENSG0000	-0,003035	0,321298	-1,98606	0,047506	0,510494
ENSG0000	-0,003827	-0,152934	-1,983179	0,047828	0,512516
ENSG0000	0,002036	0,663918	1,982749	0,047876	0,512516
ENSG0000	-0,003929	0,396488	-1,982015	0,047958	0,512516
ENSG0000	-0,002849	0,096841	-1,981319	0,048036	0,512516
ENSG0000	0,004107	-0,416443	1,980886	0,048085	0,512516
ENSG0000	0,002672	0,243159	1,979401	0,048252	0,512851
ENSG0000	-0,00339	1,307442	-1,979035	0,048294	0,512851
ENSG0000	-0,002831	-1,07399	-1,978525	0,048351	0,512851
ENSG0000	-0,00202	0,135691	-1,976802	0,048546	0,514088
ENSG0000	-0,001781	0,529834	-1,975999	0,048637	0,514223
ENSG0000	-0,002054	0,32046	-1,975265	0,048721	0,514275
ENSG0000	0,002942	-0,107976	1,973544	0,048917	0,514374
ENSG0000	0,003607	0,902447	1,972894	0,048991	0,514374
ENSG0000	-0,00483	-1,442761	-1,972764	0,049006	0,514374

ENSG0000	0,003951	0,355555	1,972431	0,049044	0,514374
ENSG0000	0,002861	0,693097	1,970126	0,049308	0,515312
ENSG0000	0,001517	-0,047766	1,969609	0,049368	0,515312
ENSG0000	-0,003636	0,022882	-1,968802	0,049461	0,515312
ENSG0000	0,002767	-1,092778	1,968435	0,049503	0,515312
ENSG0000	-0,003239	-0,496964	-1,96823	0,049527	0,515312
ENSG0000	-0,001911	0,52514	-1,967185	0,049647	0,515528
ENSG0000	0,00484	0,555709	1,966036	0,04978	0,515528
ENSG0000	-0,005292	3,01552	-1,965629	0,049827	0,515528
ENSG0000	-0,003524	-1,142614	-1,964798	0,049924	0,515528
ENSG0000	-0,002433	0,783909	-1,964653	0,049941	0,515528

geneID	logFC	AveExpr	t	P.Value	adj.P.Val
ENSG0000	-0,005039	-0,276357	-4,816411	1,49E-06	0,012274
ENSG0000	0,008454	2,188724	4,564881	5,07E-06	0,020919
ENSG0000	0,003054	0,987098	4,393908	1,13E-05	0,023363
ENSG0000	0,001621	0,175256	4,392917	1,13E-05	0,023363
ENSG0000	-0,00365	-0,510186	-4,148825	3,37E-05	0,05572
ENSG0000	-0,002641	0,251469	-3,797629	0,000147	0,202434
ENSG0000	0,006298	1,226515	3,693943	0,000222	0,252525
ENSG0000	0,000479	-0,054264	3,614865	0,000302	0,252525
ENSG0000	-0,002314	0,120207	-3,611279	0,000306	0,252525
ENSG0000	-0,001377	-0,241219	-3,592635	0,000329	0,252525
ENSG0000	-0,002667	-0,018541	-3,586975	0,000336	0,252525
ENSG0000	-0,002931	0,698749	-3,518735	0,000436	0,299921
ENSG0000	0,002428	0,05194	3,464492	0,000534	0,339084
ENSG0000	0,002659	0,837464	3,422012	0,000624	0,364342
ENSG0000	-0,002782	-0,593123	-3,399612	0,000678	0,364342
ENSG0000	0,00175	-0,076156	3,379601	0,000729	0,364342
ENSG0000	-0,001764	0,003448	-3,371802	0,00075	0,364342
ENSG0000	-0,003668	1,407669	-3,238855	0,001205	0,528757
ENSG0000	0,00176	-0,234287	3,229763	0,001244	0,528757
ENSG0000	0,002233	-1,448905	3,221347	0,001281	0,528757
ENSG0000	-0,003273	0,667881	-3,206821	0,001347	0,529678
ENSG0000	-0,001931	0,464784	-3,170408	0,001528	0,546817
ENSG0000	-0,002203	0,556427	-3,170381	0,001528	0,546817
ENSG0000	-0,003438	0,308399	-3,158879	0,001589	0,546817
ENSG0000	0,004881	-0,703453	3,111416	0,001868	0,617024
ENSG0000	0,002317	-0,556569	3,071183	0,002139	0,679287
ENSG0000	0,002656	-0,390252	3,020206	0,002533	0,738185
ENSG0000	0,00281	0,257897	3,016206	0,002567	0,738185
ENSG0000	-0,000721	-0,449708	-3,003533	0,002676	0,738185
ENSG0000	0,001771	-0,908335	2,959523	0,003089	0,738185
ENSG0000	-0,001599	-0,616311	-2,953965	0,003146	0,738185
ENSG0000	-0,002065	0,599712	-2,953774	0,003148	0,738185
ENSG0000	-0,001248	-0,486178	-2,944906	0,003239	0,738185
ENSG0000	0,004648	-2,816489	2,930684	0,003391	0,738185
ENSG0000	-0,001456	-0,041566	-2,922665	0,003479	0,738185
ENSG0000	-0,003276	-0,00444	-2,922178	0,003485	0,738185
ENSG0000	0,001423	-0,425375	2,920404	0,003505	0,738185
ENSG0000	-0,002043	-0,252356	-2,919583	0,003514	0,738185
ENSG0000	0,002575	-0,593094	2,892009	0,003837	0,738185
ENSG0000	0,002028	-1,723584	2,885363	0,003919	0,738185
ENSG0000	0,003473	-1,911798	2,870737	0,004105	0,738185
ENSG0000	-0,000871	-0,418561	-2,857337	0,004282	0,738185
ENSG0000	0,001621	-1,915557	2,834684	0,004598	0,738185
ENSG0000	0,00266	0,519732	2,823886	0,004755	0,738185
ENSG0000	-0,001492	-0,689172	-2,823156	0,004766	0,738185
ENSG0000	0,000264	-0,494717	2,80007	0,00512	0,738185
ENSG0000	-0,001591	-1,455402	-2,785989	0,005348	0,738185
ENSG0000	0,00345	2,123006	2,780363	0,005441	0,738185

ENSG0000	0,001642	-1,186027	2,778785	0,005468	0,738185
ENSG0000	0,002074	0,859409	2,777741	0,005486	0,738185
ENSG0000	0,00179	0,844617	2,775724	0,00552	0,738185
ENSG0000	0,001993	1,135641	2,774269	0,005544	0,738185
ENSG0000	0,001315	0,020873	2,768977	0,005635	0,738185
ENSG0000	0,002664	1,367883	2,76605	0,005686	0,738185
ENSG0000	-0,000994	0,191161	-2,762104	0,005755	0,738185
ENSG0000	-0,001673	-0,070643	-2,745527	0,006054	0,738185
ENSG0000	0,001263	0,226904	2,73643	0,006224	0,738185
ENSG0000	0,001666	0,211027	2,735955	0,006233	0,738185
ENSG0000	0,001015	0,216574	2,733323	0,006283	0,738185
ENSG0000	-0,000664	0,01527	-2,73138	0,00632	0,738185
ENSG0000	-0,000855	-0,7433	-2,72334	0,006475	0,738185
ENSG0000	0,002028	-1,197159	2,721171	0,006518	0,738185
ENSG0000	-0,000859	0,386881	-2,719777	0,006546	0,738185
ENSG0000	0,001551	0,305529	2,713713	0,006667	0,738185
ENSG0000	0,001919	0,770355	2,710058	0,00674	0,738185
ENSG0000	-0,001043	0,251891	-2,708347	0,006775	0,738185
ENSG0000	-0,000383	0,115335	-2,707958	0,006783	0,738185
ENSG0000	-0,00173	0,154775	-2,696568	0,007019	0,738185
ENSG0000	0,003507	-1,314767	2,695069	0,007051	0,738185
ENSG0000	-0,001587	1,363728	-2,683464	0,0073	0,738185
ENSG0000	0,003628	0,85808	2,682804	0,007315	0,738185
ENSG0000	-0,00137	-1,472128	-2,680705	0,007361	0,738185
ENSG0000	-0,001031	-0,305243	-2,678262	0,007414	0,738185
ENSG0000	0,000213	0,026368	2,672811	0,007536	0,738185
ENSG0000	0,003485	0,884812	2,670409	0,00759	0,738185
ENSG0000	-0,00053	0,248644	-2,670134	0,007596	0,738185
ENSG0000	-0,001278	-0,104702	-2,666807	0,007672	0,738185
ENSG0000	-0,001284	0,357207	-2,664898	0,007715	0,738185
ENSG0000	-0,002004	-0,366862	-2,662461	0,007771	0,738185
ENSG0000	-0,003278	-0,014854	-2,660187	0,007824	0,738185
ENSG0000	-0,001691	0,48306	-2,656472	0,007911	0,738185
ENSG0000	-0,002049	-0,714638	-2,653228	0,007987	0,738185
ENSG0000	-0,003936	-0,826128	-2,65243	0,008006	0,738185
ENSG0000	0,00076	-0,289426	2,651153	0,008036	0,738185
ENSG0000	0,001898	2,566335	2,649008	0,008087	0,738185
ENSG0000	-0,001298	0,203478	-2,644485	0,008196	0,738185
ENSG0000	0,001441	-0,337213	2,644126	0,008205	0,738185
ENSG0000	-0,001709	0,960026	-2,642242	0,008251	0,738185
ENSG0000	-0,001567	-0,779504	-2,64092	0,008283	0,738185
ENSG0000	-0,001105	-0,266433	-2,632938	0,00848	0,738185
ENSG0000	-0,001317	-0,140673	-2,627241	0,008623	0,738185
ENSG0000	0,00075	0,144024	2,626198	0,00865	0,738185
ENSG0000	-0,000902	-0,446227	-2,626034	0,008654	0,738185
ENSG0000	0,001746	-0,446545	2,624313	0,008698	0,738185
ENSG0000	-0,001277	0,591514	-2,61838	0,00885	0,738185
ENSG0000	-0,000833	0,422883	-2,617317	0,008878	0,738185

ENSG0000	-0,000991	0,118126	-2,616661	0,008895	0,738185
ENSG0000	-0,002177	0,715011	-2,604713	0,009211	0,738185
ENSG0000	-0,00083	-0,022928	-2,598602	0,009376	0,738185
ENSG0000	-0,000786	-0,241131	-2,597395	0,009409	0,738185
ENSG0000	-0,000906	-0,179634	-2,595241	0,009468	0,738185
ENSG0000	0,00161	-1,272896	2,594297	0,009494	0,738185
ENSG0000	0,004778	-0,169773	2,592479	0,009545	0,738185
ENSG0000	-0,000986	-0,810736	-2,589681	0,009623	0,738185
ENSG0000	-0,000698	0,033673	-2,584047	0,009781	0,738185
ENSG0000	-0,001121	0,860082	-2,58206	0,009837	0,738185
ENSG0000	0,002822	1,840854	2,580579	0,00988	0,738185
ENSG0000	0,000523	0,231683	2,579207	0,009919	0,738185
ENSG0000	0,002169	0,492706	2,578812	0,00993	0,738185
ENSG0000	-0,001714	-0,518834	-2,576927	0,009985	0,738185
ENSG0000	0,000438	-0,011748	2,57345	0,010085	0,738185
ENSG0000	-0,002571	1,220999	-2,573021	0,010098	0,738185
ENSG0000	0,001712	-0,969269	2,57106	0,010155	0,738185
ENSG0000	0,002021	-0,191721	2,563771	0,010371	0,738185
ENSG0000	0,001713	-0,605753	2,555291	0,010627	0,738185
ENSG0000	0,001933	-0,402154	2,55043	0,010776	0,738185
ENSG0000	0,001136	0,036438	2,544132	0,010972	0,738185
ENSG0000	-0,002602	0,562048	-2,543417	0,010995	0,738185
ENSG0000	0,001601	-0,725847	2,543148	0,011003	0,738185
ENSG0000	0,00166	0,214404	2,538404	0,011153	0,738185
ENSG0000	0,000886	-0,047379	2,537307	0,011188	0,738185
ENSG0000	-0,001216	0,573906	-2,533926	0,011297	0,738185
ENSG0000	0,001505	1,874477	2,529597	0,011437	0,738185
ENSG0000	-0,001714	0,443739	-2,529524	0,011439	0,738185
ENSG0000	-0,000406	0,253591	-2,52921	0,01145	0,738185
ENSG0000	0,00084	-1,226127	2,528731	0,011465	0,738185
ENSG0000	-0,000999	-0,575222	-2,525342	0,011576	0,738185
ENSG0000	-0,000798	-0,297714	-2,521423	0,011706	0,738185
ENSG0000	0,001447	-0,37972	2,514874	0,011925	0,738185
ENSG0000	-0,003464	-0,986472	-2,513822	0,011961	0,738185
ENSG0000	-0,001181	0,535167	-2,510056	0,012089	0,738185
ENSG0000	0,000496	0,045311	2,509045	0,012124	0,738185
ENSG0000	-0,000844	0,075129	-2,506492	0,012212	0,738185
ENSG0000	0,001196	-0,080318	2,499864	0,012442	0,738185
ENSG0000	0,001165	-1,739445	2,496066	0,012576	0,738185
ENSG0000	0,001447	-0,731671	2,495184	0,012608	0,738185
ENSG0000	-0,002503	1,92892	-2,49057	0,012772	0,738185
ENSG0000	0,00209	-0,744204	2,490458	0,012776	0,738185
ENSG0000	-0,001635	1,037342	-2,490414	0,012778	0,738185
ENSG0000	-0,002155	1,67321	-2,482785	0,013055	0,738185
ENSG0000	-0,001501	-1,028441	-2,480523	0,013138	0,738185
ENSG0000	0,001424	1,12101	2,473628	0,013394	0,738185
ENSG0000	0,000741	-0,039795	2,469632	0,013544	0,738185
ENSG0000	-0,001801	0,457632	-2,469228	0,01356	0,738185

ENSG0000	-0,001927	-1,315805	-2,465785	0,013691	0,738185
ENSG0000	0,001738	0,366453	2,464529	0,013739	0,738185
ENSG0000	0,000846	-0,387088	2,462968	0,013798	0,738185
ENSG0000	0,002412	1,350611	2,46172	0,013847	0,738185
ENSG0000	-0,002267	0,325824	-2,460483	0,013894	0,738185
ENSG0000	-0,002106	0,738943	-2,457748	0,014001	0,738185
ENSG0000	0,001269	-0,208947	2,456166	0,014062	0,738185
ENSG0000	-0,001661	0,985063	-2,453554	0,014165	0,738185
ENSG0000	0,001527	-0,066907	2,448542	0,014363	0,738185
ENSG0000	0,000505	0,019387	2,448491	0,014365	0,738185
ENSG0000	-0,001558	-0,496757	-2,442741	0,014596	0,738185
ENSG0000	0,002421	0,379438	2,440797	0,014675	0,738185
ENSG0000	0,001183	-1,171687	2,437874	0,014794	0,738185
ENSG0000	-0,001669	0,049522	-2,436772	0,014839	0,738185
ENSG0000	0,00033	-0,220725	2,436025	0,01487	0,738185
ENSG0000	-0,003619	0,485638	-2,432616	0,01501	0,738185
ENSG0000	-0,001224	-0,627342	-2,432363	0,015021	0,738185
ENSG0000	0,000743	-0,745644	2,432203	0,015027	0,738185
ENSG0000	-0,00111	-0,147844	-2,424208	0,015362	0,738185
ENSG0000	-0,003628	0,678838	-2,422153	0,015449	0,738185
ENSG0000	0,001313	0,316809	2,420527	0,015518	0,738185
ENSG0000	0,001533	-0,251459	2,418896	0,015588	0,738185
ENSG0000	0,000919	0,579884	2,418647	0,015599	0,738185
ENSG0000	-0,001086	-0,000312	-2,417917	0,01563	0,738185
ENSG0000	-0,002255	0,298504	-2,417636	0,015642	0,738185
ENSG0000	0,000726	0,0496	2,416769	0,015679	0,738185
ENSG0000	-0,001883	0,405702	-2,416133	0,015707	0,738185
ENSG0000	-0,00071	0,30455	-2,41262	0,015859	0,738185
ENSG0000	0,002489	1,063117	2,4121	0,015881	0,738185
ENSG0000	-0,002198	2,230042	-2,411154	0,015923	0,738185
ENSG0000	-0,004087	2,267851	-2,409413	0,015999	0,738185
ENSG0000	-0,000742	-0,456656	-2,408751	0,016028	0,738185
ENSG0000	-0,000541	0,379029	-2,407248	0,016094	0,738185
ENSG0000	-0,001862	0,67543	-2,406476	0,016128	0,738185
ENSG0000	-0,002365	0,579576	-2,405534	0,01617	0,738185
ENSG0000	0,00105	0,217407	2,404766	0,016204	0,738185
ENSG0000	-0,000688	-0,048058	-2,403879	0,016243	0,738185
ENSG0000	-0,000917	-0,38497	-2,401914	0,01633	0,738185
ENSG0000	-0,00365	-1,046557	-2,401286	0,016358	0,738185
ENSG0000	-0,000388	-0,115348	-2,393349	0,016716	0,75024
ENSG0000	-0,001497	-0,428362	-2,389876	0,016875	0,752793
ENSG0000	-0,000834	-0,24928	-2,387148	0,017001	0,752793
ENSG0000	0,001332	-1,184408	2,383575	0,017167	0,752793
ENSG0000	-0,000648	0,330443	-2,380909	0,017291	0,752793
ENSG0000	-0,000962	0,516727	-2,378756	0,017393	0,752793
ENSG0000	-0,001468	-0,536569	-2,374482	0,017595	0,752793
ENSG0000	0,001358	0,801703	2,371693	0,017728	0,752793
ENSG0000	-0,001391	0,326359	-2,370453	0,017788	0,752793

ENSG0000	0,003181	0,321117	2,365613	0,018022	0,752793
ENSG0000	-0,0027	0,076709	-2,362773	0,018161	0,752793
ENSG0000	0,001552	-1,092681	2,361898	0,018204	0,752793
ENSG0000	0,000548	-0,43369	2,360078	0,018293	0,752793
ENSG0000	0,004139	1,079064	2,356883	0,018451	0,752793
ENSG0000	-0,000749	0,340335	-2,356563	0,018467	0,752793
ENSG0000	0,000779	-1,216962	2,356134	0,018488	0,752793
ENSG0000	-0,000678	-0,036721	-2,35563	0,018513	0,752793
ENSG0000	-0,000874	-0,290758	-2,355414	0,018524	0,752793
ENSG0000	-0,000979	1,900479	-2,354446	0,018572	0,752793
ENSG0000	0,001777	-1,129885	2,35391	0,018599	0,752793
ENSG0000	0,001192	-0,108817	2,349125	0,01884	0,752793
ENSG0000	0,001968	0,757879	2,347217	0,018937	0,752793
ENSG0000	0,000507	0,001162	2,347036	0,018946	0,752793
ENSG0000	-0,001487	0,037563	-2,345377	0,01903	0,752793
ENSG0000	-0,000486	0,025889	-2,343392	0,019132	0,752793
ENSG0000	0,000682	0,203362	2,341363	0,019236	0,752793
ENSG0000	0,003066	1,200214	2,340271	0,019292	0,752793
ENSG0000	0,000418	-0,211201	2,340155	0,019298	0,752793
ENSG0000	-0,001028	0,63767	-2,336464	0,01949	0,752793
ENSG0000	-0,003355	-0,88698	-2,334171	0,019609	0,752793
ENSG0000	0,001107	0,572802	2,333708	0,019634	0,752793
ENSG0000	-0,003103	1,336297	-2,331358	0,019757	0,752793
ENSG0000	0,000996	-0,208492	2,330299	0,019813	0,752793
ENSG0000	-0,001336	-0,520862	-2,326735	0,020002	0,752793
ENSG0000	0,001202	0,317546	2,325028	0,020093	0,752793
ENSG0000	-0,001408	0,51634	-2,324432	0,020125	0,752793
ENSG0000	0,000698	0,062077	2,32378	0,02016	0,752793
ENSG0000	0,001155	-1,523709	2,323246	0,020189	0,752793
ENSG0000	-0,001143	0,058785	-2,320751	0,020323	0,752793
ENSG0000	0,000752	0,097387	2,314582	0,020659	0,752793
ENSG0000	0,001298	-0,356806	2,31337	0,020726	0,752793
ENSG0000	-0,000915	0,615806	-2,313116	0,020739	0,752793
ENSG0000	-0,000916	-0,548105	-2,312327	0,020783	0,752793
ENSG0000	-0,001136	0,641068	-2,312243	0,020788	0,752793
ENSG0000	-0,001049	-0,956451	-2,311043	0,020854	0,752793
ENSG0000	-0,000957	-0,320264	-2,309345	0,020948	0,752793
ENSG0000	0,003505	-0,479112	2,306792	0,02109	0,752793
ENSG0000	0,002533	2,177248	2,304473	0,02122	0,752793
ENSG0000	0,00088	0,492843	2,304349	0,021227	0,752793
ENSG0000	0,001432	0,77379	2,302341	0,021339	0,752793
ENSG0000	-0,000974	-0,507634	-2,301785	0,021371	0,752793
ENSG0000	0,002173	-0,341284	2,297624	0,021607	0,752793
ENSG0000	-0,00061	0,712536	-2,297245	0,021628	0,752793
ENSG0000	0,001412	-0,747163	2,294482	0,021787	0,752793
ENSG0000	-0,000475	0,113617	-2,292648	0,021892	0,752793
ENSG0000	-0,001125	0,445507	-2,29032	0,022027	0,752793
ENSG0000	0,000744	-0,992205	2,29032	0,022027	0,752793

ENSG0000	0,002387	0,78768	2,29031	0,022027	0,752793
ENSG0000	-0,001548	0,589404	-2,288881	0,02211	0,752793
ENSG0000	0,00051	0,060113	2,288167	0,022152	0,752793
ENSG0000	0,001845	-0,055972	2,273482	0,023021	0,763048
ENSG0000	0,000587	0,027353	2,273211	0,023038	0,763048
ENSG0000	-0,001454	-0,066575	-2,270241	0,023217	0,763048
ENSG0000	-0,001277	0,930389	-2,268918	0,023298	0,763048
ENSG0000	0,00203	-1,319866	2,268504	0,023323	0,763048
ENSG0000	-0,002312	1,798401	-2,266128	0,023468	0,763048
ENSG0000	-0,00188	0,104995	-2,265975	0,023477	0,763048
ENSG0000	-0,001151	0,575716	-2,265362	0,023515	0,763048
ENSG0000	-0,00227	-0,352106	-2,263924	0,023603	0,763048
ENSG0000	0,000919	0,826839	2,263771	0,023613	0,763048
ENSG0000	0,001563	-0,52136	2,263254	0,023645	0,763048
ENSG0000	0,001436	-0,257435	2,262762	0,023675	0,763048
ENSG0000	-0,002257	-1,495407	-2,261795	0,023735	0,763048
ENSG0000	0,001094	-0,261288	2,26031	0,023827	0,763048
ENSG0000	-0,001229	0,900408	-2,260103	0,023839	0,763048
ENSG0000	0,002516	0,888586	2,247918	0,024606	0,784554
ENSG0000	0,000572	0,161123	2,241947	0,02499	0,789641
ENSG0000	-0,001384	-0,240209	-2,24152	0,025018	0,789641
ENSG0000	-0,001494	0,730923	-2,238978	0,025183	0,789641
ENSG0000	-0,001064	-0,526794	-2,23847	0,025216	0,789641
ENSG0000	-0,000232	0,116996	-2,235159	0,025432	0,789641
ENSG0000	0,001511	-1,458218	2,234749	0,025459	0,789641
ENSG0000	-0,001819	0,685816	-2,231296	0,025687	0,789641
ENSG0000	-0,000596	0,583786	-2,226389	0,026014	0,789641
ENSG0000	-0,003695	-1,771735	-2,225422	0,026078	0,789641
ENSG0000	0,00139	0,917419	2,224626	0,026132	0,789641
ENSG0000	-0,001403	0,049915	-2,223901	0,026181	0,789641
ENSG0000	0,001812	-2,251471	2,221555	0,026339	0,789641
ENSG0000	0,001462	0,009516	2,219787	0,026459	0,789641
ENSG0000	0,001494	0,660702	2,219557	0,026475	0,789641
ENSG0000	-0,0011	-0,249562	-2,218885	0,02652	0,789641
ENSG0000	-0,001498	0,617129	-2,21818	0,026568	0,789641
ENSG0000	0,000673	-0,214911	2,216067	0,026713	0,789641
ENSG0000	-0,001136	0,036498	-2,215841	0,026728	0,789641
ENSG0000	-0,001338	0,644357	-2,213976	0,026856	0,789641
ENSG0000	0,000509	-0,184844	2,21326	0,026906	0,789641
ENSG0000	0,001701	-0,768357	2,211632	0,027018	0,789641
ENSG0000	0,00103	-0,367833	2,209992	0,027132	0,789641
ENSG0000	0,001421	1,426627	2,209895	0,027138	0,789641
ENSG0000	0,001386	-0,812498	2,209204	0,027186	0,789641
ENSG0000	-0,001758	0,922768	-2,208473	0,027237	0,789641
ENSG0000	0,001136	-1,368065	2,208261	0,027252	0,789641
ENSG0000	0,00111	-0,641763	2,206693	0,027362	0,79004
ENSG0000	-0,001945	0,60792	-2,200938	0,027767	0,792549
ENSG0000	0,002428	2,018457	2,200186	0,02782	0,792549

ENSG0000	0,000811	-0,178347	2,200136	0,027823	0,792549
ENSG0000	-0,000966	1,00897	-2,200011	0,027832	0,792549
ENSG0000	0,000806	0,252841	2,193404	0,028305	0,79708
ENSG0000	0,00397	0,179775	2,189873	0,02856	0,79708
ENSG0000	0,001524	-0,715689	2,189832	0,028563	0,79708
ENSG0000	-0,001087	0,514889	-2,189488	0,028588	0,79708
ENSG0000	0,00205	0,594712	2,186975	0,028771	0,79708
ENSG0000	0,000305	0,036783	2,186052	0,028838	0,79708
ENSG0000	0,000998	-0,647128	2,183571	0,02902	0,79708
ENSG0000	-0,001105	0,276268	-2,183207	0,029047	0,79708
ENSG0000	0,000491	-0,672608	2,182035	0,029134	0,79708
ENSG0000	0,000918	-0,626298	2,179332	0,029334	0,79708
ENSG0000	-0,000866	0,172304	-2,178956	0,029362	0,79708
ENSG0000	0,001136	-0,599724	2,176102	0,029574	0,79708
ENSG0000	0,001394	-0,47116	2,175601	0,029612	0,79708
ENSG0000	0,001286	1,561587	2,174745	0,029676	0,79708
ENSG0000	-0,001028	1,03521	-2,173687	0,029756	0,79708
ENSG0000	0,002268	0,487754	2,17205	0,029879	0,79708
ENSG0000	0,000881	0,68226	2,169476	0,030074	0,79708
ENSG0000	-0,001512	0,378121	-2,166178	0,030325	0,79708
ENSG0000	-0,000735	-0,424461	-2,165798	0,030354	0,79708
ENSG0000	-0,000719	-0,836029	-2,165713	0,03036	0,79708
ENSG0000	0,002025	0,875457	2,164881	0,030424	0,79708
ENSG0000	0,000521	0,064165	2,164685	0,030439	0,79708
ENSG0000	-0,001303	1,603546	-2,164135	0,030481	0,79708
ENSG0000	-0,000951	0,555913	-2,164039	0,030489	0,79708
ENSG0000	-0,000901	-0,457227	-2,160843	0,030735	0,79708
ENSG0000	-0,002117	1,053456	-2,160738	0,030743	0,79708
ENSG0000	-0,000632	0,08939	-2,159148	0,030866	0,79708
ENSG0000	-0,001175	-0,242116	-2,158935	0,030882	0,79708
ENSG0000	-0,001887	1,881836	-2,158681	0,030902	0,79708
ENSG0000	-0,001501	-0,21648	-2,158527	0,030914	0,79708
ENSG0000	-0,000392	-0,323824	-2,157634	0,030984	0,79708
ENSG0000	0,000505	-0,565757	2,15631	0,031087	0,797251
ENSG0000	-0,001771	-0,325559	-2,147945	0,031746	0,809696
ENSG0000	-0,001225	-0,837434	-2,147662	0,031768	0,809696
ENSG0000	0,00289	4,483369	2,145797	0,031917	0,810271
ENSG0000	-0,000563	0,468037	-2,14417	0,032047	0,810271
ENSG0000	0,001256	0,231145	2,143694	0,032085	0,810271
ENSG0000	-0,000768	0,090759	-2,142402	0,032189	0,810412
ENSG0000	-0,000589	-0,282565	-2,139608	0,032414	0,811149
ENSG0000	0,000747	-0,062294	2,138325	0,032518	0,811149
ENSG0000	0,000832	-0,255941	2,137401	0,032593	0,811149
ENSG0000	0,000965	-0,345839	2,137183	0,032611	0,811149
ENSG0000	0,001018	0,400302	2,129009	0,033282	0,822087
ENSG0000	0,000838	-0,313894	2,128806	0,033298	0,822087
ENSG0000	0,002269	1,330551	2,127658	0,033394	0,822087
ENSG0000	-0,001272	-0,208203	-2,126791	0,033465	0,822087

ENSG0000	0,000707	0,609633	2,125795	0,033548	0,822087
ENSG0000	0,002583	1,001896	2,122999	0,033782	0,824815
ENSG0000	-0,001199	-0,469432	-2,121073	0,033944	0,824815
ENSG0000	0,000902	1,076061	2,119928	0,03404	0,824815
ENSG0000	-0,00151	-0,552538	-2,119704	0,034059	0,824815
ENSG0000	0,001074	0,398577	2,117949	0,034208	0,825986
ENSG0000	0,001719	-0,363481	2,114499	0,034501	0,829088
ENSG0000	0,001408	0,892074	2,113734	0,034566	0,829088
ENSG0000	-0,000961	-0,527789	-2,112023	0,034713	0,829088
ENSG0000	0,001182	-0,349245	2,11068	0,034828	0,829088
ENSG0000	-0,000886	-0,388035	-2,106825	0,035161	0,829088
ENSG0000	-0,001171	-1,311275	-2,099237	0,035825	0,829088
ENSG0000	-0,002247	1,042525	-2,095247	0,036178	0,829088
ENSG0000	-0,001426	-0,313842	-2,094038	0,036286	0,829088
ENSG0000	0,002661	-0,256256	2,093754	0,036311	0,829088
ENSG0000	-0,002624	-0,302683	-2,093258	0,036355	0,829088
ENSG0000	-0,002567	0,941933	-2,091272	0,036533	0,829088
ENSG0000	-0,001207	0,473791	-2,090171	0,036631	0,829088
ENSG0000	-0,000863	0,56636	-2,089037	0,036733	0,829088
ENSG0000	0,000856	-0,984899	2,088116	0,036816	0,829088
ENSG0000	0,00045	0,141575	2,086965	0,03692	0,829088
ENSG0000	0,001249	-0,161624	2,08605	0,037003	0,829088
ENSG0000	-0,000583	-0,3851	-2,08576	0,037029	0,829088
ENSG0000	0,000767	0,194129	2,085619	0,037042	0,829088
ENSG0000	-0,001245	-0,676221	-2,084573	0,037137	0,829088
ENSG0000	0,000492	0,052151	2,083834	0,037204	0,829088
ENSG0000	-0,000485	-0,071784	-2,083552	0,03723	0,829088
ENSG0000	0,000886	-1,11134	2,08323	0,037259	0,829088
ENSG0000	0,001277	-0,638978	2,083062	0,037275	0,829088
ENSG0000	0,0012	0,479529	2,080758	0,037485	0,829088
ENSG0000	-0,002117	1,483355	-2,080749	0,037486	0,829088
ENSG0000	-0,000717	-0,15258	-2,080423	0,037516	0,829088
ENSG0000	0,000939	-0,035804	2,080312	0,037526	0,829088
ENSG0000	-0,002321	-0,042363	-2,079877	0,037566	0,829088
ENSG0000	-0,001083	-0,458124	-2,079455	0,037605	0,829088
ENSG0000	-0,001358	1,031395	-2,078437	0,037698	0,829088
ENSG0000	0,001171	-0,427321	2,077349	0,037799	0,829088
ENSG0000	0,002627	1,628401	2,077193	0,037813	0,829088
ENSG0000	0,000535	0,088951	2,076024	0,037921	0,829088
ENSG0000	-0,001465	0,257763	-2,075629	0,037958	0,829088
ENSG0000	-0,001148	0,270944	-2,074633	0,03805	0,829088
ENSG0000	0,001303	1,125719	2,07288	0,038213	0,829088
ENSG0000	-0,00045	-0,119661	-2,07011	0,038471	0,829088
ENSG0000	0,00113	-0,702189	2,069131	0,038563	0,829088
ENSG0000	0,001116	-0,635241	2,068689	0,038605	0,829088
ENSG0000	0,000734	-0,427542	2,068162	0,038654	0,829088
ENSG0000	-0,000409	-0,058416	-2,06594	0,038864	0,829088
ENSG0000	-0,002922	0,324447	-2,065372	0,038917	0,829088

ENSG0000	-0,000441	0,073426	-2,061622	0,039273	0,829088
ENSG0000	-0,000546	-0,368801	-2,060207	0,039408	0,829088
ENSG0000	-0,000335	-0,199865	-2,059831	0,039444	0,829088
ENSG0000	-0,001834	0,635918	-2,059079	0,039516	0,829088
ENSG0000	-0,001695	1,179638	-2,055895	0,039822	0,829088
ENSG0000	-0,00075	0,237357	-2,05583	0,039829	0,829088
ENSG0000	0,000738	0,216813	2,053467	0,040057	0,829088
ENSG0000	0,001441	-0,104474	2,053137	0,040089	0,829088
ENSG0000	0,000562	0,36548	2,051658	0,040233	0,829088
ENSG0000	-0,001332	-0,831361	-2,051008	0,040296	0,829088
ENSG0000	0,001927	0,133995	2,050668	0,040329	0,829088
ENSG0000	0,000528	0,206177	2,049901	0,040404	0,829088
ENSG0000	0,002004	-0,932024	2,048991	0,040493	0,829088
ENSG0000	0,002076	0,861495	2,047049	0,040683	0,829088
ENSG0000	0,001608	-1,315502	2,045882	0,040798	0,829088
ENSG0000	0,001033	0,26253	2,044252	0,040959	0,829088
ENSG0000	-0,001783	-0,108269	-2,041776	0,041204	0,829088
ENSG0000	0,000626	-0,410157	2,041696	0,041212	0,829088
ENSG0000	-0,001623	1,806437	-2,041201	0,041261	0,829088
ENSG0000	-0,000597	-0,466096	-2,04057	0,041324	0,829088
ENSG0000	-0,001182	0,642914	-2,039722	0,041408	0,829088
ENSG0000	0,001829	0,29238	2,036707	0,04171	0,829088
ENSG0000	0,002107	0,070513	2,03278	0,042105	0,829088
ENSG0000	0,001013	-0,436971	2,03182	0,042202	0,829088
ENSG0000	0,001057	-0,329335	2,031123	0,042273	0,829088
ENSG0000	-0,00063	0,19374	-2,029648	0,042423	0,829088
ENSG0000	0,00085	0,19016	2,02829	0,042561	0,829088
ENSG0000	-0,000923	0,161005	-2,028062	0,042584	0,829088
ENSG0000	-0,001384	0,410685	-2,026999	0,042693	0,829088
ENSG0000	-0,000758	-0,112617	-2,026714	0,042722	0,829088
ENSG0000	-0,000468	1,35644	-2,026658	0,042728	0,829088
ENSG0000	-0,000675	-0,220472	-2,026488	0,042745	0,829088
ENSG0000	0,002189	0,48377	2,026416	0,042753	0,829088
ENSG0000	0,003272	-0,277818	2,025852	0,04281	0,829088
ENSG0000	0,001481	1,064337	2,025394	0,042857	0,829088
ENSG0000	0,002748	0,403752	2,024976	0,0429	0,829088
ENSG0000	0,001025	0,128276	2,024222	0,042978	0,829088
ENSG0000	-0,002076	0,151887	-2,023375	0,043065	0,829088
ENSG0000	-0,001383	0,586427	-2,022442	0,043161	0,829088
ENSG0000	-0,000762	0,598473	-2,022167	0,04319	0,829088
ENSG0000	0,001583	0,034502	2,021295	0,04328	0,829088
ENSG0000	-0,001023	1,414166	-2,019979	0,043416	0,829088
ENSG0000	0,00043	0,146873	2,019699	0,043445	0,829088
ENSG0000	0,000521	0,201539	2,017899	0,043632	0,829088
ENSG0000	0,000634	-0,129238	2,017175	0,043708	0,829088
ENSG0000	-0,000739	0,3113	-2,016594	0,043769	0,829088
ENSG0000	0,001572	-0,42205	2,016513	0,043777	0,829088
ENSG0000	-0,002099	-0,899232	-2,013317	0,044112	0,829088

ENSG0000	-0,000941	0,819259	-2,01122	0,044333	0,829088
ENSG0000	0,000757	-0,094748	2,010619	0,044397	0,829088
ENSG0000	-0,000287	-0,11479	-2,010505	0,044409	0,829088
ENSG0000	-0,003131	0,209778	-2,006976	0,044783	0,829088
ENSG0000	-0,001292	-0,014968	-2,006424	0,044842	0,829088
ENSG0000	-0,001935	-0,671991	-2,00573	0,044916	0,829088
ENSG0000	-0,00086	0,249873	-2,005091	0,044984	0,829088
ENSG0000	0,00145	-0,991189	2,003203	0,045186	0,829088
ENSG0000	0,001396	1,135303	2,002134	0,045301	0,829088
ENSG0000	0,000902	-0,323089	2,001024	0,045421	0,829088
ENSG0000	0,001067	0,499857	1,999761	0,045557	0,829088
ENSG0000	-0,000953	-1,424609	-1,999406	0,045596	0,829088
ENSG0000	-0,000779	-0,172571	-1,999107	0,045628	0,829088
ENSG0000	0,000474	0,465712	1,998452	0,045699	0,829088
ENSG0000	0,00266	-0,535392	1,997326	0,045821	0,829088
ENSG0000	-0,00113	0,962984	-1,995274	0,046044	0,829088
ENSG0000	0,00246	-1,190134	1,995033	0,04607	0,829088
ENSG0000	0,000908	-0,344674	1,993431	0,046245	0,829088
ENSG0000	-0,000889	-0,317489	-1,993277	0,046262	0,829088
ENSG0000	-0,000426	0,269336	-1,992145	0,046386	0,829088
ENSG0000	0,001116	-1,511084	1,991763	0,046428	0,829088
ENSG0000	0,001045	-0,024353	1,987594	0,046888	0,829088
ENSG0000	0,000972	1,182848	1,98639	0,047021	0,829088
ENSG0000	-0,000895	-0,46748	-1,985701	0,047098	0,829088
ENSG0000	0,001677	-0,590403	1,985691	0,047099	0,829088
ENSG0000	-0,001611	0,372549	-1,98508	0,047167	0,829088
ENSG0000	-0,000909	0,253825	-1,983035	0,047395	0,829088
ENSG0000	-0,001199	1,380566	-1,980531	0,047675	0,829088
ENSG0000	-0,001326	1,178415	-1,980089	0,047725	0,829088
ENSG0000	-0,001888	0,470945	-1,979996	0,047735	0,829088
ENSG0000	-0,000873	0,295335	-1,979475	0,047794	0,829088
ENSG0000	-0,000426	-0,194059	-1,977926	0,047969	0,829088
ENSG0000	0,000515	-0,187652	1,977924	0,047969	0,829088
ENSG0000	-0,00072	-0,099047	-1,977113	0,04806	0,829088
ENSG0000	0,003437	-1,942596	1,976	0,048186	0,829088
ENSG0000	0,00144	-0,347511	1,974712	0,048332	0,829088
ENSG0000	0,001081	0,441725	1,972888	0,04854	0,829088
ENSG0000	0,00154	-1,491247	1,97219	0,04862	0,829088
ENSG0000	-0,002998	1,341638	-1,971595	0,048688	0,829088
ENSG0000	0,001145	0,273905	1,970242	0,048842	0,829088
ENSG0000	0,000735	-0,686522	1,969186	0,048964	0,829088
ENSG0000	0,000779	0,972937	1,968079	0,049091	0,829088
ENSG0000	-0,000608	-0,556599	-1,966846	0,049233	0,829088
ENSG0000	-0,002563	-0,021294	-1,966811	0,049237	0,829088
ENSG0000	-0,002891	-0,985523	-1,966305	0,049295	0,829088
ENSG0000	-0,00123	-0,372599	-1,965552	0,049382	0,829088
ENSG0000	0,000509	-0,305358	1,965244	0,049418	0,829088
ENSG0000	-0,001525	-2,629678	-1,963908	0,049573	0,829088

ENSG0000	0,00132	-2,548264	1,961666	0,049833	0,829088

geneID	logFC	AveExpr	t	P.Value	adj.P.Val
ENSG0000	0,007399	1,998693	4,721561	2,38E-06	0,015643
ENSG0000	0,001759	0,636432	4,110613	3,98E-05	0,094535
ENSG0000	-0,004039	-0,536411	-4,092317	4,31E-05	0,094535
ENSG0000	-0,003961	-0,972503	-3,881558	0,000105	0,172023
ENSG0000	0,002408	-0,385819	3,72889	0,000194	0,254747
ENSG0000	-0,003687	0,843592	-3,643841	0,00027	0,296325
ENSG0000	-0,002419	-0,084561	-3,598075	0,000322	0,303068
ENSG0000	0,003397	0,55219	3,533904	0,000412	0,338591
ENSG0000	-0,002366	-0,248278	-3,499308	0,000469	0,342799
ENSG0000	-0,000172	-0,071839	-3,457434	0,000548	0,360611
ENSG0000	0,001099	-0,431026	3,355586	0,000795	0,475844
ENSG0000	-0,001359	0,821647	-3,253256	0,001145	0,545616
ENSG0000	0,001908	-0,584146	3,223766	0,00127	0,545616
ENSG0000	0,001035	-0,376627	3,222015	0,001278	0,545616
ENSG0000	0,002981	-0,0751	3,177571	0,00149	0,545616
ENSG0000	0,00149	-0,405268	3,172883	0,001515	0,545616
ENSG0000	-0,001404	0,625279	-3,169204	0,001534	0,545616
ENSG0000	-0,003892	-0,206468	-3,135382	0,001722	0,545616
ENSG0000	0,003373	-0,51391	3,132966	0,001736	0,545616
ENSG0000	0,000544	-0,097702	3,124588	0,001786	0,545616
ENSG0000	-0,002133	-0,073375	-3,112993	0,001858	0,545616
ENSG0000	-0,001571	-0,278818	-3,10589	0,001903	0,545616
ENSG0000	-0,00158	-0,60233	-3,105315	0,001907	0,545616
ENSG0000	-0,001586	-0,911879	-3,089235	0,002013	0,551998
ENSG0000	0,001322	-0,562469	3,070208	0,002146	0,561107
ENSG0000	-0,003626	0,180432	-3,060448	0,002217	0,561107
ENSG0000	-0,001903	-0,34929	-3,038326	0,002386	0,581573
ENSG0000	0,001579	0,381345	3,00344	0,002677	0,618057
ENSG0000	0,002984	-1,03418	2,993015	0,00277	0,618057
ENSG0000	-0,002064	0,121432	-2,987835	0,002817	0,618057
ENSG0000	-0,001769	-0,5673	-2,959799	0,003087	0,630502
ENSG0000	-0,00058	0,09259	-2,950808	0,003178	0,630502
ENSG0000	0,002274	0,101628	2,942147	0,003268	0,630502
ENSG0000	-0,001607	0,399764	-2,923616	0,003469	0,630502
ENSG0000	0,001921	-0,094325	2,919929	0,00351	0,630502
ENSG0000	0,001089	-0,378838	2,917105	0,003542	0,630502
ENSG0000	-0,001841	-1,533867	-2,916856	0,003545	0,630502
ENSG0000	-0,002928	-1,59147	-2,885387	0,003919	0,636986
ENSG0000	-0,000829	-0,178374	-2,885349	0,003919	0,636986
ENSG0000	-0,003127	-1,411874	-2,885218	0,003921	0,636986
ENSG0000	-0,003204	0,679742	-2,881429	0,003968	0,636986
ENSG0000	0,001581	-1,064756	2,859144	0,004258	0,667182
ENSG0000	0,001266	-1,094443	2,819187	0,004825	0,721921
ENSG0000	0,001404	0,189128	2,819103	0,004827	0,721921
ENSG0000	-0,000765	0,101966	-2,787633	0,005321	0,734212
ENSG0000	-0,000696	-0,130029	-2,778088	0,00548	0,734212
ENSG0000	0,004071	2,582882	2,769255	0,00563	0,734212
ENSG0000	-0,002344	-0,923037	-2,768824	0,005638	0,734212

ENSG0000	0,001535	0,432858	2,760772	0,005779	0,734212
ENSG0000	-0,000781	1,292793	-2,759375	0,005803	0,734212
ENSG0000	0,003884	1,006168	2,759293	0,005805	0,734212
ENSG0000	0,004865	-0,013518	2,745591	0,006053	0,734212
ENSG0000	0,001375	-0,634597	2,743461	0,006092	0,734212
ENSG0000	0,001341	0,301849	2,743167	0,006097	0,734212
ENSG0000	-0,001864	0,943242	-2,741089	0,006136	0,734212
ENSG0000	0,00201	-1,259642	2,732874	0,006291	0,73932
ENSG0000	-0,001249	0,419921	-2,718862	0,006564	0,75557
ENSG0000	-0,000597	-0,06866	-2,710343	0,006735	0,75557
ENSG0000	0,001532	-0,426875	2,702376	0,006898	0,75557
ENSG0000	0,003751	-1,633965	2,695222	0,007048	0,75557
ENSG0000	-0,001029	0,10852	-2,692825	0,007099	0,75557
ENSG0000	-0,002492	0,089048	-2,690746	0,007143	0,75557
ENSG0000	0,004871	-0,356416	2,663934	0,007738	0,75557
ENSG0000	0,000946	0,96348	2,656846	0,007902	0,75557
ENSG0000	-0,000668	0,155336	-2,656571	0,007908	0,75557
ENSG0000	0,000805	0,343841	2,656241	0,007916	0,75557
ENSG0000	0,002627	1,587171	2,655828	0,007926	0,75557
ENSG0000	-0,001401	-0,132186	-2,646946	0,008137	0,75557
ENSG0000	0,001094	-0,175903	2,639546	0,008317	0,75557
ENSG0000	0,001453	-0,244921	2,636087	0,008402	0,75557
ENSG0000	-0,001347	0,203372	-2,632903	0,008481	0,75557
ENSG0000	-0,000761	-0,16877	-2,630442	0,008542	0,75557
ENSG0000	-0,001237	0,218484	-2,630198	0,008549	0,75557
ENSG0000	-0,001701	-0,26736	-2,629436	0,008568	0,75557
ENSG0000	-0,002796	1,78062	-2,627728	0,008611	0,75557
ENSG0000	0,002121	-0,378825	2,622657	0,00874	0,756809
ENSG0000	-0,000295	-0,098355	-2,610401	0,009059	0,765152
ENSG0000	-0,001879	0,381842	-2,609104	0,009094	0,765152
ENSG0000	-0,00166	0,293904	-2,601692	0,009292	0,765152
ENSG0000	-0,001351	0,307832	-2,601356	0,009301	0,765152
ENSG0000	0,001004	-0,277881	2,575077	0,010038	0,776218
ENSG0000	-0,001144	-0,502061	-2,573125	0,010095	0,776218
ENSG0000	0,00167	-0,533163	2,573071	0,010096	0,776218
ENSG0000	-0,000891	0,053657	-2,572539	0,010112	0,776218
ENSG0000	0,001009	0,246626	2,571908	0,01013	0,776218
ENSG0000	-0,002163	0,660759	-2,567767	0,010252	0,776218
ENSG0000	-0,001178	-0,678673	-2,565321	0,010325	0,776218
ENSG0000	0,001593	-0,21274	2,563483	0,010379	0,776218
ENSG0000	-0,003623	-0,831122	-2,558203	0,010538	0,777509
ENSG0000	0,001511	0,640543	2,554658	0,010646	0,777509
ENSG0000	-0,00157	0,164589	-2,549114	0,010817	0,777509
ENSG0000	-0,002637	1,084309	-2,537566	0,01118	0,777509
ENSG0000	0,002095	-0,667552	2,536596	0,011211	0,777509
ENSG0000	-0,000985	-1,08218	-2,53596	0,011231	0,777509
ENSG0000	-0,001169	0,607256	-2,53576	0,011238	0,777509
ENSG0000	-0,001131	0,24714	-2,529411	0,011443	0,777509

ENSG0000	-0,001849	1,212543	-2,525014	0,011587	0,777509
ENSG0000	-0,000686	-0,109784	-2,522319	0,011676	0,777509
ENSG0000	0,000911	-0,197169	2,519786	0,01176	0,777509
ENSG0000	-0,00124	0,197678	-2,512522	0,012005	0,777509
ENSG0000	0,003502	-1,542261	2,510103	0,012088	0,777509
ENSG0000	0,001944	-0,726741	2,508794	0,012132	0,777509
ENSG0000	0,001335	0,441156	2,504199	0,012291	0,777509
ENSG0000	-0,000655	-0,1289	-2,503179	0,012327	0,777509
ENSG0000	0,003341	1,552878	2,498883	0,012477	0,777509
ENSG0000	-0,002457	-1,401414	-2,493883	0,012654	0,777509
ENSG0000	0,001966	1,814375	2,488562	0,012845	0,777509
ENSG0000	0,001119	0,215669	2,485463	0,012957	0,777509
ENSG0000	0,000789	0,825511	2,48374	0,01302	0,777509
ENSG0000	0,003238	1,655712	2,475172	0,013336	0,777509
ENSG0000	0,00141	0,259519	2,468583	0,013584	0,777509
ENSG0000	-0,002016	1,293652	-2,463537	0,013777	0,777509
ENSG0000	-0,002669	-0,186759	-2,458454	0,013973	0,777509
ENSG0000	0,001681	0,534009	2,456767	0,014039	0,777509
ENSG0000	-0,001397	0,448828	-2,454618	0,014123	0,777509
ENSG0000	0,000868	0,352969	2,454258	0,014137	0,777509
ENSG0000	-0,000371	0,136605	-2,449333	0,014332	0,777509
ENSG0000	-0,001727	0,286816	-2,44553	0,014484	0,777509
ENSG0000	-0,001247	1,416073	-2,445512	0,014484	0,777509
ENSG0000	-0,001525	0,550872	-2,444826	0,014512	0,777509
ENSG0000	0,002137	-0,282368	2,440589	0,014683	0,777509
ENSG0000	-0,002011	-0,496078	-2,440236	0,014698	0,777509
ENSG0000	-0,002018	0,760627	-2,435909	0,014874	0,777509
ENSG0000	-0,002756	0,127004	-2,431565	0,015054	0,777509
ENSG0000	-0,001762	-1,132124	-2,429176	0,015153	0,777509
ENSG0000	-0,003189	-1,063749	-2,424841	0,015335	0,777509
ENSG0000	-0,001179	-0,30332	-2,42339	0,015397	0,777509
ENSG0000	-0,002291	1,718667	-2,420765	0,015508	0,777509
ENSG0000	-0,00122	0,943438	-2,412889	0,015847	0,777509
ENSG0000	0,000716	1,049404	2,406756	0,016116	0,777509
ENSG0000	0,001724	0,316564	2,405639	0,016165	0,777509
ENSG0000	0,002865	-0,705709	2,402276	0,016314	0,777509
ENSG0000	0,000446	0,026676	2,394775	0,016652	0,777509
ENSG0000	0,000406	-0,058989	2,393778	0,016697	0,777509
ENSG0000	-0,002933	1,283529	-2,389836	0,016877	0,777509
ENSG0000	-0,001176	-0,667036	-2,389621	0,016887	0,777509
ENSG0000	-0,003294	2,093883	-2,38491	0,017105	0,777509
ENSG0000	0,002111	0,610882	2,384298	0,017133	0,777509
ENSG0000	0,000577	0,207028	2,381524	0,017263	0,777509
ENSG0000	-0,001518	0,259342	-2,381243	0,017276	0,777509
ENSG0000	0,001405	0,231611	2,377145	0,017469	0,777509
ENSG0000	0,001002	-0,216064	2,374249	0,017606	0,777509
ENSG0000	0,00114	-0,836818	2,372861	0,017673	0,777509
ENSG0000	-0,001283	0,162032	-2,371648	0,017731	0,777509

ENSG0000	0,001636	-0,36087	2,369779	0,01782	0,777509
ENSG0000	0,000812	0,530493	2,368771	0,017869	0,777509
ENSG0000	0,000217	-0,137053	2,365688	0,018018	0,777509
ENSG0000	-0,000408	-0,195213	-2,365517	0,018027	0,777509
ENSG0000	0,000546	0,837687	2,364299	0,018086	0,777509
ENSG0000	-0,001736	0,300692	-2,362601	0,018169	0,777509
ENSG0000	-0,002204	-0,425267	-2,360113	0,018291	0,777509
ENSG0000	0,001335	-0,357582	2,359659	0,018314	0,777509
ENSG0000	-0,001041	0,357897	-2,355298	0,01853	0,777509
ENSG0000	0,004601	2,382356	2,355036	0,018543	0,777509
ENSG0000	-0,003353	-0,57075	-2,353653	0,018612	0,777509
ENSG0000	-0,002707	4,831258	-2,353134	0,018638	0,777509
ENSG0000	-0,003671	1,154995	-2,352483	0,018671	0,777509
ENSG0000	0,002214	0,721578	2,351046	0,018743	0,777509
ENSG0000	0,002662	-0,761154	2,34713	0,018941	0,777509
ENSG0000	0,000572	-0,409239	2,343209	0,019141	0,777509
ENSG0000	-0,001049	1,820777	-2,343109	0,019146	0,777509
ENSG0000	-0,002268	0,887156	-2,340247	0,019293	0,777509
ENSG0000	0,000699	0,343228	2,339609	0,019326	0,777509
ENSG0000	-0,001577	0,934756	-2,338658	0,019376	0,777509
ENSG0000	-0,000883	0,591515	-2,335283	0,019551	0,778577
ENSG0000	0,00149	0,699552	2,332881	0,019677	0,778577
ENSG0000	0,000963	0,87359	2,328941	0,019885	0,778577
ENSG0000	-0,001854	-1,376322	-2,326481	0,020016	0,778577
ENSG0000	0,001013	0,366438	2,326107	0,020036	0,778577
ENSG0000	-0,000743	-0,216103	-2,324677	0,020112	0,778577
ENSG0000	0,001661	0,923143	2,321945	0,020259	0,77967
ENSG0000	0,001445	0,469846	2,319195	0,020408	0,780825
ENSG0000	0,001114	0,041488	2,312895	0,020752	0,788711
ENSG0000	0,001258	0,966848	2,309829	0,020921	0,788711
ENSG0000	-0,003382	0,401208	-2,307576	0,021046	0,788711
ENSG0000	-0,000934	0,009545	-2,300993	0,021416	0,788711
ENSG0000	-0,001126	-0,052215	-2,297248	0,021628	0,788711
ENSG0000	0,002484	-0,48594	2,297159	0,021633	0,788711
ENSG0000	0,000404	0,374648	2,29663	0,021664	0,788711
ENSG0000	-0,001247	0,370742	-2,294885	0,021763	0,788711
ENSG0000	0,001913	0,008044	2,294613	0,021779	0,788711
ENSG0000	0,002067	0,713003	2,290154	0,022036	0,788711
ENSG0000	0,001253	0,899003	2,288618	0,022125	0,788711
ENSG0000	-0,001064	0,565347	-2,286479	0,02225	0,788711
ENSG0000	-0,001083	-0,165089	-2,28539	0,022314	0,788711
ENSG0000	0,001198	-0,31572	2,281402	0,022549	0,788711
ENSG0000	-0,00151	0,453665	-2,280581	0,022597	0,788711
ENSG0000	0,001513	-0,193901	2,280364	0,02261	0,788711
ENSG0000	0,001125	0,104821	2,27368	0,02301	0,788711
ENSG0000	-0,000988	-0,27222	-2,272476	0,023082	0,788711
ENSG0000	0,001443	-0,500984	2,270846	0,023181	0,788711
ENSG0000	0,000816	-1,179852	2,266199	0,023464	0,788711

ENSG0000	0,000313	0,067217	2,262321	0,023702	0,788711
ENSG0000	0,001493	0,231983	2,262128	0,023714	0,788711
ENSG0000	0,001863	0,29837	2,260829	0,023794	0,788711
ENSG0000	-0,001948	-0,0405	-2,258331	0,02395	0,788711
ENSG0000	0,001122	2,139595	2,256551	0,024061	0,788711
ENSG0000	-0,001448	0,470195	-2,256292	0,024077	0,788711
ENSG0000	0,000746	-0,086432	2,256047	0,024092	0,788711
ENSG0000	-0,001638	0,26235	-2,255899	0,024102	0,788711
ENSG0000	0,001581	0,593586	2,24987	0,024482	0,788711
ENSG0000	0,003217	0,372119	2,248889	0,024545	0,788711
ENSG0000	0,001461	0,526687	2,246081	0,024724	0,788711
ENSG0000	-0,000654	0,496982	-2,245448	0,024764	0,788711
ENSG0000	-0,002023	-0,421892	-2,241465	0,025021	0,788711
ENSG0000	0,001589	0,79154	2,241146	0,025042	0,788711
ENSG0000	-0,00119	1,735689	-2,23997	0,025118	0,788711
ENSG0000	-0,001347	0,130238	-2,239037	0,025179	0,788711
ENSG0000	-0,000797	1,215422	-2,238083	0,025241	0,788711
ENSG0000	0,002253	0,81518	2,236966	0,025314	0,788711
ENSG0000	0,00137	-1,183267	2,236371	0,025353	0,788711
ENSG0000	0,000537	-1,723631	2,235535	0,025408	0,788711
ENSG0000	-0,002931	-0,611339	-2,225249	0,02609	0,797807
ENSG0000	0,001358	0,159338	2,224409	0,026147	0,797807
ENSG0000	-0,002246	-0,009225	-2,223623	0,026199	0,797807
ENSG0000	0,001548	-0,822621	2,217366	0,026624	0,797807
ENSG0000	-0,000718	-0,240635	-2,217061	0,026645	0,797807
ENSG0000	0,001185	-0,511714	2,214787	0,026801	0,797807
ENSG0000	0,000998	0,283776	2,213206	0,026909	0,797807
ENSG0000	0,001865	2,660645	2,212656	0,026947	0,797807
ENSG0000	0,001578	2,557741	2,212505	0,026958	0,797807
ENSG0000	-0,002505	0,899291	-2,21227	0,026974	0,797807
ENSG0000	-0,00216	-0,24441	-2,2114	0,027034	0,797807
ENSG0000	0,002002	-0,382718	2,208824	0,027213	0,798575
ENSG0000	0,001171	0,154935	2,207535	0,027303	0,798575
ENSG0000	0,002455	1,312059	2,201833	0,027703	0,806705
ENSG0000	-0,000525	-0,208914	-2,197906	0,027982	0,809654
ENSG0000	-0,001125	0,135726	-2,196946	0,028051	0,809654
ENSG0000	0,002806	2,05593	2,193262	0,028315	0,810343
ENSG0000	0,001617	-0,034865	2,192897	0,028341	0,810343
ENSG0000	-0,001988	1,314349	-2,189947	0,028555	0,810343
ENSG0000	-0,000698	-1,744498	-2,189776	0,028567	0,810343
ENSG0000	-0,001497	0,035973	-2,18594	0,028847	0,814088
ENSG0000	0,000708	-0,438156	2,184578	0,028946	0,814088
ENSG0000	0,001367	-0,3821	2,178087	0,029426	0,82207
ENSG0000	-0,001119	-0,299754	-2,176969	0,02951	0,82207
ENSG0000	-0,002059	1,349212	-2,174615	0,029686	0,82207
ENSG0000	-0,000475	-0,839574	-2,166508	0,0303	0,82207
ENSG0000	-0,001099	-0,034756	-2,166322	0,030314	0,82207
ENSG0000	-0,003279	-0,812291	-2,165475	0,030378	0,82207

ENSG0000	0,00143	1,442917	2,165335	0,030389	0,82207
ENSG0000	0,003697	3,660671	2,163741	0,030511	0,82207
ENSG0000	0,001094	1,183531	2,157386	0,031003	0,82207
ENSG0000	0,001819	-1,027795	2,15521	0,031173	0,82207
ENSG0000	-0,000963	1,555155	-2,154877	0,031199	0,82207
ENSG0000	0,002921	-0,949647	2,153881	0,031277	0,82207
ENSG0000	-0,001135	0,519881	-2,153868	0,031278	0,82207
ENSG0000	0,003327	0,098235	2,153559	0,031302	0,82207
ENSG0000	0,001713	-0,660034	2,151733	0,031446	0,82207
ENSG0000	0,00251	0,633983	2,150919	0,03151	0,82207
ENSG0000	0,001526	-0,272347	2,14962	0,031613	0,82207
ENSG0000	0,002171	0,466739	2,149498	0,031622	0,82207
ENSG0000	0,001229	0,699914	2,147973	0,031743	0,82207
ENSG0000	-0,000899	0,598336	-2,144723	0,032003	0,82207
ENSG0000	-0,000597	0,14623	-2,13992	0,032389	0,82207
ENSG0000	-0,001604	1,394785	-2,138153	0,032532	0,82207
ENSG0000	-0,000265	-0,539335	-2,137868	0,032555	0,82207
ENSG0000	0,000658	-0,105061	2,134952	0,032793	0,82207
ENSG0000	-0,001538	-0,343282	-2,134577	0,032823	0,82207
ENSG0000	-0,001204	-1,096816	-2,132454	0,032997	0,82207
ENSG0000	0,001173	-0,042161	2,131704	0,033059	0,82207
ENSG0000	-0,000784	-0,356954	-2,129484	0,033242	0,82207
ENSG0000	-0,001317	-1,956627	-2,128374	0,033334	0,82207
ENSG0000	0,001259	1,991836	2,127702	0,03339	0,82207
ENSG0000	0,00241	0,440951	2,124486	0,033658	0,82207
ENSG0000	0,000563	0,055796	2,123664	0,033726	0,82207
ENSG0000	0,000607	-0,533168	2,122974	0,033784	0,82207
ENSG0000	0,000768	-0,089478	2,121861	0,033878	0,82207
ENSG0000	0,002382	0,968953	2,120547	0,033988	0,82207
ENSG0000	-0,001052	-0,304821	-2,119982	0,034036	0,82207
ENSG0000	-0,001266	0,782532	-2,11918	0,034104	0,82207
ENSG0000	0,00103	-0,812975	2,11907	0,034113	0,82207
ENSG0000	0,001845	-0,516934	2,118507	0,03416	0,82207
ENSG0000	0,000971	-0,345124	2,117722	0,034227	0,82207
ENSG0000	-0,001659	0,433852	-2,114872	0,034469	0,82488
ENSG0000	-0,001467	-1,488305	-2,113014	0,034628	0,825676
ENSG0000	-0,000604	-0,258198	-2,10988	0,034897	0,829092
ENSG0000	0,000344	0,179644	2,105829	0,035248	0,829753
ENSG0000	0,000474	0,258798	2,104281	0,035383	0,829753
ENSG0000	-0,001416	1,485693	-2,103378	0,035461	0,829753
ENSG0000	-0,000968	-0,531469	-2,103251	0,035472	0,829753
ENSG0000	0,001261	-0,071355	2,096781	0,036042	0,829753
ENSG0000	0,001255	-0,479367	2,093825	0,036304	0,829753
ENSG0000	-0,000775	0,294231	-2,091979	0,036469	0,829753
ENSG0000	0,00181	0,435943	2,09117	0,036542	0,829753
ENSG0000	-0,001016	0,007234	-2,090898	0,036566	0,829753
ENSG0000	-0,00112	-0,056686	-2,09061	0,036592	0,829753
ENSG0000	0,000538	-0,186938	2,08885	0,03675	0,829753

ENSG0000	0,001303	-0,239682	2,086652	0,036949	0,829753
ENSG0000	-0,001819	-0,681048	-2,083499	0,037235	0,829753
ENSG0000	0,000615	-0,343187	2,083343	0,037249	0,829753
ENSG0000	-0,00298	-0,467958	-2,083139	0,037268	0,829753
ENSG0000	0,001572	-0,09494	2,082231	0,037351	0,829753
ENSG0000	0,000835	-0,30009	2,082019	0,03737	0,829753
ENSG0000	-0,000803	0,247431	-2,081164	0,037448	0,829753
ENSG0000	-0,00075	-0,193447	-2,080034	0,037552	0,829753
ENSG0000	-0,002223	0,383548	-2,078375	0,037704	0,829753
ENSG0000	-0,003034	-1,699157	-2,078031	0,037736	0,829753
ENSG0000	0,00072	-0,069178	2,076335	0,037892	0,829753
ENSG0000	-0,001209	-0,627073	-2,075172	0,038	0,829753
ENSG0000	-0,000928	-0,37627	-2,074473	0,038065	0,829753
ENSG0000	0,001334	0,067946	2,074252	0,038085	0,829753
ENSG0000	-0,001149	0,528426	-2,072626	0,038236	0,829753
ENSG0000	0,001881	-0,018585	2,070213	0,038462	0,829753
ENSG0000	0,000478	0,170018	2,068178	0,038653	0,829753
ENSG0000	-0,000586	-0,057337	-2,067726	0,038695	0,829753
ENSG0000	0,001293	0,217395	2,067596	0,038708	0,829753
ENSG0000	0,002485	-0,540872	2,066186	0,03884	0,829899
ENSG0000	-0,003013	-2,245519	-2,061547	0,039281	0,833062
ENSG0000	0,00152	0,495274	2,059981	0,03943	0,833062
ENSG0000	-0,000369	0,074446	-2,058019	0,039618	0,833062
ENSG0000	0,001111	0,166379	2,057998	0,03962	0,833062
ENSG0000	-0,000536	0,809175	-2,057536	0,039664	0,833062
ENSG0000	0,000478	-0,115013	2,056668	0,039748	0,833062
ENSG0000	0,000635	0,198083	2,053853	0,04002	0,836096
ENSG0000	-0,001781	-0,247394	-2,052345	0,040166	0,836495
ENSG0000	0,001762	2,215134	2,047817	0,040608	0,839122
ENSG0000	-0,001162	-0,318594	-2,047234	0,040665	0,839122
ENSG0000	-0,000872	-0,783273	-2,047137	0,040675	0,839122
ENSG0000	0,001014	0,677152	2,044163	0,040968	0,842504
ENSG0000	0,00144	-1,341837	2,042333	0,041149	0,842504
ENSG0000	0,001813	1,484864	2,041313	0,04125	0,842504
ENSG0000	0,000998	0,32022	2,04029	0,041352	0,842504
ENSG0000	0,000274	0,040329	2,038622	0,041518	0,842504
ENSG0000	-0,000399	-0,21687	-2,036615	0,041719	0,842504
ENSG0000	0,000962	-0,627895	2,035193	0,041862	0,842504
ENSG0000	0,001142	-0,868563	2,035183	0,041863	0,842504
ENSG0000	0,001646	-1,05491	2,031482	0,042236	0,844963
ENSG0000	-0,001306	-0,678827	-2,031308	0,042254	0,844963
ENSG0000	0,000992	0,020458	2,030166	0,04237	0,844963
ENSG0000	-0,001213	-0,441628	-2,027134	0,042679	0,848554
ENSG0000	0,001675	-0,545087	2,02521	0,042876	0,849907
ENSG0000	0,000654	0,454077	2,023552	0,043047	0,850724
ENSG0000	0,003118	-2,121647	2,020966	0,043314	0,853439
ENSG0000	-0,001121	0,375645	-2,018767	0,043542	0,855374
ENSG0000	-0,001247	-0,450121	-2,015283	0,043906	0,856556

ENSG0000	-0,000818	0,18308	-2,014068	0,044033	0,856556
ENSG0000	0,001475	-1,014915	2,012679	0,044179	0,856556
ENSG0000	-0,001131	0,444278	-2,011214	0,044334	0,856556
ENSG0000	0,000661	0,272066	2,008921	0,044576	0,856556
ENSG0000	-0,000741	-0,209304	-2,005116	0,044982	0,856556
ENSG0000	0,001336	-1,267005	2,003186	0,045188	0,856556
ENSG0000	-0,001836	0,298067	-2,00131	0,04539	0,856556
ENSG0000	-0,000222	0,362559	-2,001297	0,045391	0,856556
ENSG0000	-0,00086	0,400146	-2,000849	0,04544	0,856556
ENSG0000	-0,000648	0,343029	-2,000803	0,045445	0,856556
ENSG0000	0,002565	1,309549	1,997635	0,045787	0,856556
ENSG0000	-0,000275	-0,079433	-1,997452	0,045807	0,856556
ENSG0000	0,000591	-0,721537	1,996562	0,045904	0,856556
ENSG0000	0,001522	-0,617115	1,994653	0,046112	0,856556
ENSG0000	0,001695	-0,421693	1,994264	0,046154	0,856556
ENSG0000	0,000884	0,165715	1,993339	0,046256	0,856556
ENSG0000	-0,002092	1,264981	-1,992848	0,046309	0,856556
ENSG0000	0,00205	0,959621	1,992803	0,046314	0,856556
ENSG0000	0,003105	1,522752	1,99243	0,046355	0,856556
ENSG0000	0,000796	0,291179	1,990682	0,046547	0,856556
ENSG0000	-0,001918	1,290209	-1,990156	0,046605	0,856556
ENSG0000	-0,001393	0,42063	-1,99004	0,046618	0,856556
ENSG0000	0,000805	-0,067878	1,989059	0,046726	0,856556
ENSG0000	-0,001638	0,630417	-1,986024	0,047062	0,857092
ENSG0000	0,00125	1,128658	1,983526	0,04734	0,857092
ENSG0000	0,000967	-0,135861	1,98285	0,047416	0,857092
ENSG0000	0,000429	0,347966	1,982325	0,047474	0,857092
ENSG0000	0,000945	-0,058622	1,981669	0,047548	0,857092
ENSG0000	-0,001977	1,423677	-1,981405	0,047577	0,857092
ENSG0000	-0,000725	0,27261	-1,979885	0,047748	0,857092
ENSG0000	-0,001433	0,234855	-1,979448	0,047797	0,857092
ENSG0000	-0,000956	0,038079	-1,974645	0,04834	0,858526
ENSG0000	-0,000732	-0,100706	-1,973778	0,048439	0,858526
ENSG0000	0,002839	0,233437	1,970008	0,048869	0,858526
ENSG0000	0,000965	1,06852	1,969826	0,04889	0,858526
ENSG0000	-0,000997	0,66553	-1,966337	0,049291	0,858526
ENSG0000	-0,001741	0,266998	-1,965529	0,049385	0,858526
ENSG0000	-0,003087	-0,077799	-1,96434	0,049522	0,858526
ENSG0000	-0,001715	1,057593	-1,963657	0,049602	0,858526
ENSG0000	0,001146	0,298773	1,961583	0,049843	0,858526
ENSG0000	-0,001551	-0,231855	-1,960639	0,049953	0,858526
ENSG0000	0,000538	-0,311384	1,960558	0,049962	0,858526
ENSG0000	0,00106	0,212227	1,960338	0,049988	0,858526
