# Supplementary material for: The transcriptome-wide association search for genes and genetic variants which associate with BMI and gestational weight gain in women with type 1 diabetes
Source: Mol Med. 2021 Jan 20;27:6. doi: 10.1186/s10020-020-00266-z (PMC7818927; doi:10.1186/s10020-020-00266-z)
Supplement: Supplementary file 8 — Additional file 8: Table S5a. The results of PrediXcan on T2D&ARIC cohorts on BMI only. b. The results of PrediXcan on T2D&ARIC cohorts on BMI and GWG. c. The results of PrediXcan on T2D&ARIC cohorts on GWG only. [file 10020_2020_266_MOESM8_ESM.zip › Table S5a.pdf]

1,"ENSG00000242435.1"
2,"ENSG00000250312.2"
3,"ENSG00000112530.7"
4,"ENSG00000250892.1"
5,"ENSG00000169592.10"
6,"ENSG00000023041.7"
7,"ENSG00000090238.7"
8,"ENSG00000229117.4"
9,"ENSG00000136492.4"
10,"ENSG00000149927.13"
11,"ENSG00000198917.7"
12,"ENSG00000164610.4"
13,"ENSG00000204556.4"
14,"ENSG00000186704.8"
15,"ENSG00000078142.7"
16,"ENSG00000170873.14"
17,"ENSG00000130177.10"
18,"ENSG00000127314.13"
19,"ENSG00000255874.1"
20,"ENSG00000136111.8"
21,"ENSG00000159063.8"
22,"ENSG00000100997.14"
23,"ENSG00000171763.13"
24,"ENSG00000149084.7"
25,"ENSG00000149922.6"
26,"ENSG00000234722.3"
27,"ENSG00000264247.1"
28,"ENSG00000181045.10"
29,"ENSG00000174775.12"
30,"ENSG00000160293.12"
31,"ENSG00000111581.5"
32,"ENSG00000167136.6"
33,"ENSG00000233121.1"
34,"ENSG00000106992.13"
35,"ENSG00000205763.9"
36,"ENSG00000135424.11"
37,"ENSG00000125871.9"
38,"ENSG00000106305.5"
39,"ENSG00000225726.1"
40,"ENSG00000176715.11"
41,"ENSG00000082213.13"
42,"ENSG00000100379.13"
43,"ENSG00000247373.2"
44,"ENSG00000237296.5"
45,"ENSG00000164440.10"
46,"ENSG00000143486.11"
47,"ENSG00000268734.1"
48,"ENSG00000256552.2"

49,"ENSG00000103549.17"
50,"ENSG00000238290.1"
51,"ENSG00000198589.6"
52,"ENSG00000141905.13"
53,"ENSG00000233820.2"
54,"ENSG00000236204.1"
55,"ENSG00000257542.4"
56,"ENSG00000196639.6"
57,"ENSG00000117569.14"
58,"ENSG00000185986.10"
59,"ENSG00000075415.8"
60,"ENSG00000143452.11"
61,"ENSG00000170909.9"
62,"ENSG00000260082.1"
63,"ENSG00000160796.12"
64,"ENSG00000095485.12"
65,"ENSG00000106367.9"
66,"ENSG00000160886.9"
67,"ENSG00000261056.2"
68,"ENSG00000121864.5"
69,"ENSG00000144182.12"
70,"ENSG00000171747.4"
71,"ENSG00000173113.2"
72,"ENSG00000112031.11"
73,"ENSG00000170439.5"
74,"ENSG00000132139.8"
75,"ENSG00000127616.13"
76,"ENSG00000139323.9"
77,"ENSG00000134115.8"
78,"ENSG00000175166.12"
79,"ENSG00000168228.10"
80,"ENSG00000106484.10"
81,"ENSG00000089053.8"
82,"ENSG00000177225.12"
83,"ENSG00000130226.12"
84,"ENSG00000213977.3"
85,"ENSG00000188807.8"
86,"ENSG00000089280.14"
87,"ENSG00000103671.5"
88,"ENSG00000126432.9"
89,"ENSG00000234645.2"
90,"ENSG00000013523.5"
91,"ENSG00000035403.12"
92,"ENSG00000142794.14"
93,"ENSG00000140527.10"
94,"ENSG00000261308.1"
95,"ENSG00000213753.6"
96,"ENSG00000232901.1"

97,"ENSG00000112619.6"
98,"ENSG00000269928.1"
99,"ENSG00000259344.1"
100,"ENSG00000204529.3"
101,"ENSG00000099977.9"
102,"ENSG00000248712.3"
103,"ENSG00000116151.9"
104,"ENSG00000259433.2"
105,"ENSG00000100364.14"
106,"ENSG00000213519.2"
107,"ENSG00000174749.5"
108,"ENSG00000165280.11"
109,"ENSG00000130779.15"
110,"ENSG00000204913.5"
111,"ENSG00000115486.7"
112,"ENSG00000228126.1"
113,"ENSG00000112249.9"
114,"ENSG00000167850.3"
115,"ENSG00000230310.1"
116,"ENSG00000167397.10"
117,"ENSG00000177294.6"
118,"ENSG00000148735.10"
119,"ENSG00000262468.1"
120,"ENSG00000134463.10"
121,"ENSG00000115368.5"
122,"ENSG00000163406.6"
123,"ENSG00000185664.10"
124,"ENSG00000171792.6"
125,"ENSG00000064225.8"
126,"ENSG00000141161.7"
127,"ENSG00000124743.5"
128,"ENSG00000168701.14"
129,"ENSG00000237887.1"
130,"ENSG00000114744.4"
131,"ENSG00000173530.5"
132,"ENSG00000174373.11"
133,"ENSG00000213222.3"
134,"ENSG00000240602.3"
135,"ENSG00000186281.8"
136,"ENSG00000154277.8"
137,"ENSG00000111262.4"
138,"ENSG00000115556.9"
139,"ENSG00000177156.6"
140,"ENSG00000152404.11"
141,"ENSG00000127993.10"
142,"ENSG00000135913.6"
143,"ENSG00000247077.2"
144,"ENSG00000166928.6"

145,"ENSG00000111962.7"
146,"ENSG00000140950.11"
147,"ENSG00000198515.9"
148,"ENSG00000269746.1"
149,"ENSG00000101004.10"
150,"ENSG00000133107.10"
151,"ENSG00000258100.1"
152,"ENSG00000179673.3"
153,"ENSG00000006740.12"
154,"ENSG00000180383.3"
155,"ENSG00000033030.9"
156,"ENSG00000100033.12"
157,"ENSG00000235109.3"
158,"ENSG00000213290.4"
159,"ENSG00000130332.10"
160,"ENSG00000166927.8"
161,"ENSG00000113597.13"
162,"ENSG00000135697.5"
163,"ENSG00000239883.4"
164,"ENSG00000168653.6"
165,"ENSG00000177370.4"
166,"ENSG00000137574.6"
167,"ENSG00000141485.11"
168,"ENSG00000118855.14"
169,"ENSG00000178199.9"
170,"ENSG00000238151.1"
171,"ENSG00000198796.6"
172,"ENSG00000134215.11"
173,"ENSG00000124570.13"
174,"ENSG00000145020.10"
175,"ENSG00000112357.8"
176,"ENSG00000135437.5"
177,"ENSG00000164978.13"
178,"ENSG00000127948.9"
179,"ENSG00000157870.10"
180,"ENSG00000184985.12"
181,"ENSG00000215325.4"
182,"ENSG00000196872.6"
183,"ENSG00000250411.1"
184,"ENSG00000127990.11"
185,"ENSG00000165282.9"
186,"ENSG00000173915.8"
187,"ENSG00000183888.4"
188,"ENSG00000130528.7"
189,"ENSG00000255513.1"
190,"ENSG00000131795.8"
191,"ENSG00000169738.3"
192,"ENSG00000144559.6"

193,"ENSG00000138760.4"
194,"ENSG00000188878.12"
195,"ENSG00000105852.6"
196,"ENSG00000126231.9"
197,"ENSG00000136141.10"
198,"ENSG00000166148.2"
199,"ENSG00000145022.4"
200,"ENSG00000167780.7"
201,"ENSG00000177600.4"
202,"ENSG00000101353.10"
203,"ENSG00000187474.4"
204,"ENSG00000139651.9"
205,"ENSG00000038274.12"
206,"ENSG00000125863.13"
207,"ENSG00000224549.1"
208,"ENSG00000137709.5"
209,"ENSG00000118596.7"
210,"ENSG00000135617.3"
211,"ENSG00000146830.9"
212,"ENSG00000108602.13"
213,"ENSG00000226549.3"
214,"ENSG00000152700.9"
215,"ENSG00000167815.7"
216,"ENSG00000116857.12"
217,"ENSG00000148362.6"
218,"ENSG00000063046.13"
219,"ENSG00000136161.8"
220,"ENSG00000072364.8"
221,"ENSG00000134028.10"
222,"ENSG00000224848.1"
223,"ENSG00000173258.8"
224,"ENSG00000133818.8"
225,"ENSG00000167904.10"
226,"ENSG00000135747.7"
227,"ENSG00000178297.8"
228,"ENSG00000124920.9"
229,"ENSG00000158411.6"
230,"ENSG00000150076.18"
231,"ENSG00000111199.6"
232,"ENSG00000243961.2"
233,"ENSG00000242612.2"
234,"ENSG00000248979.2"
235,"ENSG00000253816.2"
236,"ENSG00000146425.6"
237,"ENSG00000174915.7"
238,"ENSG00000158517.9"
239,"ENSG00000119397.12"
240,"ENSG00000134014.12"

241,"ENSG00000262223.2"
242,"ENSG00000125124.7"
243,"ENSG00000124140.8"
244,"ENSG00000185774.10"
245,"ENSG00000100100.8"
246,"ENSG00000140988.11"
247,"ENSG00000204713.6"
248,"ENSG00000182853.7"
249,"ENSG00000023191.12"
250,"ENSG00000151834.11"
251,"ENSG00000151366.8"
252,"ENSG00000167543.11"
253,"ENSG00000116062.10"
254,"ENSG00000169715.10"
255,"ENSG00000136944.13"
256,"ENSG00000177380.9"
257,"ENSG00000125743.6"
258,"ENSG00000272947.1"
259,"ENSG00000229246.1"
260,"ENSG00000187778.9"
261,"ENSG00000272216.1"
262,"ENSG00000172123.8"
263,"ENSG00000248489.1"
264,"ENSG00000187942.7"
265,"ENSG00000149451.13"
266,"ENSG00000167766.14"
267,"ENSG00000153044.5"
268,"ENSG00000257923.5"
269,"ENSG00000167377.13"
270,"ENSG00000251161.2"
271,"ENSG00000251595.3"
272,"ENSG00000112812.11"
273,"ENSG00000169398.15"
274,"ENSG00000140254.8"
275,"ENSG00000132749.6"
276,"ENSG00000048540.10"
277,"ENSG00000130669.13"
278,"ENSG00000100055.16"
279,"ENSG00000050130.13"
280,"ENSG00000164845.12"
281,"ENSG00000256249.1"
282,"ENSG00000138660.7"
283,"ENSG00000213542.3"
284,"ENSG00000130775.11"
285,"ENSG00000115275.7"
286,"ENSG00000245060.2"
287,"ENSG00000271208.1"
288,"ENSG00000144366.11"

289,"ENSG00000164919.6"
290,"ENSG00000263063.1"
291,"ENSG00000159352.11"
292,"ENSG00000183569.13"
293,"ENSG00000142798.12"
294,"ENSG00000197417.7"
295,"ENSG00000101365.16"
296,"ENSG00000189298.9"
297,"ENSG00000120647.5"
298,"ENSG00000197782.10"
299,"ENSG00000135018.9"
300,"ENSG00000226051.2"
301,"ENSG00000168944.11"
302,"ENSG00000188554.9"
303,"ENSG00000198646.9"
304,"ENSG00000119414.7"
305,"ENSG00000118873.11"
306,"ENSG00000107317.7"
307,"ENSG00000205629.7"
308,"ENSG00000198771.6"
309,"ENSG00000120328.4"
310,"ENSG00000036257.8"
311,"ENSG00000257949.2"
312,"ENSG00000123609.6"
313,"ENSG00000119711.8"
314,"ENSG00000103202.8"
315,"ENSG00000120688.7"
316,"ENSG00000143631.10"
317,"ENSG00000156463.13"
318,"ENSG00000260054.1"
319,"ENSG00000090686.11"
320,"ENSG00000173253.10"
321,"ENSG00000205853.6"
322,"ENSG00000197915.5"
323,"ENSG00000103187.6"
324,"ENSG00000254352.1"
325,"ENSG00000186715.6"
326,"ENSG00000254761.1"
327,"ENSG00000083544.9"
328,"ENSG00000146005.3"
329,"ENSG00000171840.7"
330,"ENSG00000109182.7"
331,"ENSG00000163453.7"
332,"ENSG00000185272.9"
333,"ENSG00000029534.15"
334,"ENSG00000100593.13"
335,"ENSG00000213261.3"
336,"ENSG00000124789.7"

337,"ENSG00000151575.10"
338,"ENSG00000103512.10"
339,"ENSG00000105726.12"
340,"ENSG00000116032.5"
341,"ENSG00000205456.7"
342,"ENSG00000189227.4"
343,"ENSG00000143801.12"
344,"ENSG00000187699.6"
345,"ENSG00000230812.1"
346,"ENSG00000260947.1"
347,"ENSG00000105926.11"
348,"ENSG00000234630.1"
349,"ENSG00000221995.4"
350,"ENSG00000256323.1"
351,"ENSG00000189068.5"
352,"ENSG00000262728.1"
353,"ENSG00000142961.10"
354,"ENSG00000145386.5"
355,"ENSG00000198598.2"
356,"ENSG00000173209.18"
357,"ENSG00000174482.6"
358,"ENSG00000162804.9"
359,"ENSG00000213178.3"
360,"ENSG00000004809.9"
361,"ENSG00000013583.4"
362,"ENSG00000186431.14"
363,"ENSG00000261600.1"
364,"ENSG00000170486.6"
365,"ENSG00000254004.2"
366,"ENSG00000213246.2"
367,"ENSG00000002919.10"
368,"ENSG00000236471.1"
369,"ENSG00000258572.1"
370,"ENSG00000111615.8"
371,"ENSG00000114405.6"
372,"ENSG00000047056.10"
373,"ENSG00000135372.4"
374,"ENSG00000141569.6"
375,"ENSG00000160867.10"
376,"ENSG00000187049.5"
377,"ENSG00000184428.8"
378,"ENSG00000103051.14"
379,"ENSG00000268225.1"
380,"ENSG00000198040.6"
381,"ENSG00000251521.2"
382,"ENSG00000183549.6"
383,"ENSG00000225373.4"
384,"ENSG00000131473.12"

385,"ENSG00000176809.6"
386,"ENSG00000240583.6"
387,"ENSG00000249685.1"
388,"ENSG00000128891.11"
389,"ENSG00000135314.8"
390,"ENSG00000160172.6"
391,"ENSG00000158717.6"
392,"ENSG00000187535.9"
393,"ENSG00000012223.8"
394,"ENSG00000128872.5"
395,"ENSG00000117151.8"
396,"ENSG00000179886.4"
397,"ENSG00000117115.8"
398,"ENSG00000161395.8"
399,"ENSG00000148690.10"
400,"ENSG00000261467.2"
401,"ENSG00000243697.1"
402,"ENSG00000122033.10"
403,"ENSG00000203778.3"
404,"ENSG00000178971.9"
405,"ENSG00000264727.1"
406,"ENSG00000127328.17"
407,"ENSG00000117245.8"
408,"ENSG00000196154.7"
409,"ENSG00000108349.10"
410,"ENSG00000136235.11"
411,"ENSG00000112679.10"
412,"ENSG00000234840.1"
413,"ENSG00000103942.8"
414,"ENSG00000099917.13"
415,"ENSG00000169136.4"
416,"ENSG00000159658.6"
417,"ENSG00000005194.10"
418,"ENSG00000239697.6"
419,"ENSG00000138722.5"
420,"ENSG00000231389.3"
421,"ENSG00000112877.6"
422,"ENSG00000026297.11"
423,"ENSG00000198482.6"
424,"ENSG00000164941.9"
425,"ENSG00000198189.6"
426,"ENSG00000226383.1"
427,"ENSG00000224843.2"
428,"ENSG00000166869.2"
429,"ENSG00000254272.1"
430,"ENSG00000105443.9"
431,"ENSG00000169629.7"
432,"ENSG00000127419.12"

433,"ENSG00000222040.3"
434,"ENSG00000155115.6"
435,"ENSG00000135778.7"
436,"ENSG00000273456.1"
437,"ENSG00000078246.11"
438,"ENSG00000238105.3"
439,"ENSG00000196358.6"
440,"ENSG00000227001.2"
441,"ENSG00000123179.9"
442,"ENSG00000215114.3"
443,"ENSG00000134363.7"
444,"ENSG00000235477.2"
445,"ENSG00000008710.13"
446,"ENSG00000235173.2"
447,"ENSG00000223861.1"
448,"ENSG00000272068.1"
449,"ENSG00000230710.1"
450,"ENSG00000260192.1"
451,"ENSG00000174943.5"
452,"ENSG00000153395.5"
453,"ENSG00000206549.8"
454,"ENSG00000261672.1"
455,"ENSG00000198939.3"
456,"ENSG00000167962.8"
457,"ENSG00000187848.8"
458,"ENSG00000167191.7"
459,"ENSG00000147614.3"
460,"ENSG00000271605.1"
461,"ENSG00000173156.2"
462,"ENSG00000186184.11"
463,"ENSG00000215241.3"
464,"ENSG00000261340.1"
465,"ENSG00000220305.1"
466,"ENSG00000089154.6"
467,"ENSG00000134899.13"
468,"ENSG00000105131.3"
469,"ENSG00000230224.1"
470,"ENSG00000198252.7"
471,"ENSG00000169169.10"
472,"ENSG00000122025.10"
473,"ENSG00000060558.3"
474,"ENSG00000272430.1"
475,"ENSG00000178950.12"
476,"ENSG00000135387.15"
477,"ENSG00000104332.7"
478,"ENSG00000103502.9"
479,"ENSG00000160856.16"
480,"ENSG00000134030.9"

481,"ENSG00000180917.12"
482,"ENSG00000262877.3"
483,"ENSG00000110944.4"
484,"ENSG00000114503.6"
485,"ENSG00000141971.8"
486,"ENSG00000163877.9"
487,"ENSG00000110274.10"
488,"ENSG00000152104.7"
489,"ENSG00000248124.3"
490,"ENSG00000244165.1"
491,"ENSG00000170469.6"
492,"ENSG00000134324.7"
493,"ENSG00000236829.5"
494,"ENSG00000196793.9"
495,"ENSG00000219392.1"
496,"ENSG00000077463.10"
497,"ENSG00000124159.11"
498,"ENSG00000183117.13"
499,"ENSG00000172992.7"
500,"ENSG00000225399.3"
501,"ENSG00000254634.3"
502,"ENSG00000176024.12"
503,"ENSG00000226469.1"
504,"ENSG00000159904.7"
505,"ENSG00000106290.10"
506,"ENSG00000228221.1"
507,"ENSG00000164418.15"
508,"ENSG00000119440.7"
509,"ENSG00000072657.4"
510,"ENSG00000128709.10"
511,"ENSG00000107521.14"
512,"ENSG00000173421.12"
513,"ENSG00000170854.13"
514,"ENSG00000186532.7"
515,"ENSG00000134253.5"
516,"ENSG00000026652.9"
517,"ENSG00000233381.2"
518,"ENSG00000140948.7"
519,"ENSG00000198265.7"
520,"ENSG00000087085.9"
521,"ENSG00000144229.7"
522,"ENSG00000215790.2"
523,"ENSG00000178772.6"
524,"ENSG00000172273.8"
525,"ENSG00000167615.12"
526,"ENSG00000234882.1"
527,"ENSG00000165912.11"
528,"ENSG00000172379.14"

529,"ENSG00000137700.12"
530,"ENSG00000254427.1"
531,"ENSG00000197815.3"
532,"ENSG00000197181.7"
533,"ENSG00000127578.6"
534,"ENSG00000197935.6"
535,"ENSG00000173890.12"
536,"ENSG00000196071.3"
537,"ENSG00000213028.3"
538,"ENSG00000249931.3"
539,"ENSG00000112367.6"
540,"ENSG00000130227.12"
541,"ENSG00000236501.1"
542,"ENSG00000084092.6"
543,"ENSG00000121318.2"
544,"ENSG00000198538.6"
545,"ENSG00000143622.6"
546,"ENSG00000164695.4"
547,"ENSG00000188186.6"
548,"ENSG00000255147.1"
549,"ENSG00000204187.5"
550,"ENSG00000112706.7"
551,"ENSG00000183826.12"
552,"ENSG00000183506.12"
553,"ENSG00000132207.13"
554,"ENSG00000153291.11"
555,"ENSG00000137364.4"
556,"ENSG00000112308.8"
557,"ENSG00000147535.12"
558,"ENSG00000232615.4"
559,"ENSG00000124562.5"
560,"ENSG00000104970.6"
561,"ENSG00000204291.6"
562,"ENSG00000235770.1"
563,"ENSG00000135951.10"
564,"ENSG00000127249.10"
565,"ENSG00000120868.9"
566,"ENSG00000028203.13"
567,"ENSG00000150627.11"
568,"ENSG00000261064.1"
569,"ENSG00000269175.1"
570,"ENSG00000196236.8"
571,"ENSG00000171735.14"
572,"ENSG00000166924.4"
573,"ENSG00000243319.3"
574,"ENSG00000118640.6"
575,"ENSG00000204677.6"
576,"ENSG00000139641.8"

577,"ENSG00000079246.11"
578,"ENSG00000221838.5"
579,"ENSG00000225684.3"
580,"ENSG00000142544.6"
581,"ENSG00000137265.10"
582,"ENSG00000183748.4"
583,"ENSG00000106261.12"
584,"ENSG00000177954.7"
585,"ENSG00000110665.7"
586,"ENSG00000105364.9"
587,"ENSG00000235330.2"
588,"ENSG00000266258.1"
589,"ENSG00000158815.6"
590,"ENSG00000138964.12"
591,"ENSG00000197146.2"
592,"ENSG00000170893.3"
593,"ENSG00000106178.2"
594,"ENSG00000174891.8"
595,"ENSG00000168826.11"
596,"ENSG00000175879.7"
597,"ENSG00000160161.5"
598,"ENSG00000151631.7"
599,"ENSG00000224239.1"
600,"ENSG00000139433.5"
601,"ENSG00000134470.15"
602,"ENSG00000145555.10"
603,"ENSG00000120586.4"
604,"ENSG00000237840.2"
605,"ENSG00000196693.10"
606,"ENSG00000228049.5"
607,"ENSG00000152475.6"
608,"ENSG00000272002.1"
609,"ENSG00000149346.10"
610,"ENSG00000141580.11"
611,"ENSG00000227098.1"
612,"ENSG00000165138.12"
613,"ENSG00000180113.11"
614,"ENSG00000160284.10"
615,"ENSG00000264433.1"
616,"ENSG00000220744.1"
617,"ENSG00000258230.2"
618,"ENSG00000197093.6"
619,"ENSG00000264801.1"
620,"ENSG00000258546.1"
621,"ENSG00000128652.7"
622,"ENSG00000179528.11"
623,"ENSG00000135679.17"
624,"ENSG00000128710.5"

625,"ENSG00000066336.7"
626,"ENSG00000130396.16"
627,"ENSG00000183044.7"
628,"ENSG00000271943.1"
629,"ENSG00000174501.10"
630,"ENSG00000171806.7"
631,"ENSG00000143442.17"
632,"ENSG00000152583.8"
633,"ENSG00000085788.9"
634,"ENSG00000267681.1"
635,"ENSG00000176422.10"
636,"ENSG00000231445.1"
637,"ENSG00000269963.1"
638,"ENSG00000105877.13"
639,"ENSG00000232677.2"
640,"ENSG00000250327.1"
641,"ENSG00000215914.3"
642,"ENSG00000169299.9"
643,"ENSG00000183605.12"
644,"ENSG00000178467.13"
645,"ENSG00000119878.5"
646,"ENSG00000163328.9"
647,"ENSG00000134759.9"
648,"ENSG00000214595.7"
649,"ENSG00000228043.1"
650,"ENSG00000146373.12"
651,"ENSG00000141127.10"
652,"ENSG00000236319.2"
653,"ENSG00000099984.6"
654,"ENSG00000069966.14"
655,"ENSG00000240859.1"
656,"ENSG00000270000.1"
657,"ENSG00000230069.3"
658,"ENSG00000237575.4"
659,"ENSG00000260257.1"
660,"ENSG00000248971.2"
661,"ENSG00000204305.9"
662,"ENSG00000251288.2"
663,"ENSG00000214243.3"
664,"ENSG00000196616.8"
665,"ENSG00000256629.1"
666,"ENSG00000182841.8"
667,"ENSG00000173566.9"
668,"ENSG00000103381.7"
669,"ENSG00000179299.12"
670,"ENSG00000233558.1"
671,"ENSG00000188848.11"
672,"ENSG00000227999.1"

673,"ENSG00000100711.9"
674,"ENSG00000204356.7"
675,"ENSG00000197548.8"
676,"ENSG00000172954.9"
677,"ENSG00000135596.13"
678,"ENSG00000088876.7"
679,"ENSG00000143924.14"
680,"ENSG00000272894.1"
681,"ENSG00000125851.5"
682,"ENSG00000108352.7"
683,"ENSG00000145354.5"
684,"ENSG00000146001.4"
685,"ENSG00000234585.2"
686,"ENSG00000100181.17"
687,"ENSG00000251593.1"
688,"ENSG00000125551.14"
689,"ENSG00000224207.2"
690,"ENSG00000198039.7"
691,"ENSG00000161405.12"
692,"ENSG00000183779.5"
693,"ENSG00000236849.1"
694,"ENSG00000164669.8"
695,"ENSG00000197191.3"
696,"ENSG00000253869.1"
697,"ENSG00000090020.6"
698,"ENSG00000235117.2"
699,"ENSG00000197375.8"
700,"ENSG00000166562.4"
701,"ENSG00000106113.14"
702,"ENSG00000006744.14"
703,"ENSG00000196591.7"
704,"ENSG00000186468.8"
705,"ENSG00000227725.2"
706,"ENSG00000228106.1"
707,"ENSG00000103356.11"
708,"ENSG00000187266.9"
709,"ENSG00000185127.5"
710,"ENSG00000164850.10"
711,"ENSG00000034533.7"
712,"ENSG00000137098.9"
713,"ENSG00000250486.2"
714,"ENSG00000125775.10"
715,"ENSG00000270948.1"
716,"ENSG00000105976.10"
717,"ENSG00000164989.11"
718,"ENSG00000164308.12"
719,"ENSG00000127952.12"
720,"ENSG00000131435.8"

721,"ENSG00000184574.5"
722,"ENSG00000166788.5"
723,"ENSG00000230701.1"
724,"ENSG00000132952.7"
725,"ENSG00000197150.8"
726,"ENSG00000157191.15"
727,"ENSG00000130402.7"
728,"ENSG00000132972.14"
729,"ENSG00000070785.12"
730,"ENSG00000123607.10"
731,"ENSG00000251411.1"
732,"ENSG00000164880.11"
733,"ENSG00000103472.5"
734,"ENSG00000162365.7"
735,"ENSG00000215237.5"
736,"ENSG00000266497.1"
737,"ENSG00000197444.5"
738,"ENSG00000141994.11"
739,"ENSG00000180610.9"
740,"ENSG00000183615.5"
741,"ENSG00000042317.12"
742,"ENSG00000136146.10"
743,"ENSG00000225544.1"
744,"ENSG00000033178.8"
745,"ENSG00000167916.4"
746,"ENSG00000158089.10"
747,"ENSG00000258701.1"
748,"ENSG00000163872.11"
749,"ENSG00000204314.6"
750,"ENSG00000183454.9"
751,"ENSG00000186377.6"
752,"ENSG00000172927.3"
753,"ENSG00000250656.2"
754,"ENSG00000257093.2"
755,"ENSG00000182389.14"
756,"ENSG00000213015.4"
757,"ENSG00000167186.6"
758,"ENSG00000163607.10"
759,"ENSG00000198805.7"
760,"ENSG00000130559.14"
761,"ENSG00000143363.11"
762,"ENSG00000092020.6"
763,"ENSG00000101367.8"
764,"ENSG00000112584.9"
765,"ENSG00000106236.3"
766,"ENSG00000100485.7"
767,"ENSG00000196743.4"
768,"ENSG00000100731.11"

769,"ENSG00000177700.5"
770,"ENSG00000058063.11"
771,"ENSG00000158987.15"
772,"ENSG00000178053.13"
773,"ENSG00000258908.1"
774,"ENSG00000007372.16"
775,"ENSG00000180992.5"
776,"ENSG00000119865.4"
777,"ENSG00000160305.13"
778,"ENSG00000196542.4"
779,"ENSG00000124459.7"
780,"ENSG00000232372.1"
781,"ENSG00000113211.3"
782,"ENSG00000174912.6"
783,"ENSG00000165502.6"
784,"ENSG00000251396.2"
785,"ENSG00000230658.1"
786,"ENSG00000003393.10"
787,"ENSG00000113141.11"
788,"ENSG00000242866.5"
789,"ENSG00000168827.10"
790,"ENSG00000038219.8"
791,"ENSG00000113269.9"
792,"ENSG00000160307.5"
793,"ENSG00000162511.7"
794,"ENSG00000104969.5"
795,"ENSG00000048471.9"
796,"ENSG00000100979.10"
797,"ENSG00000212125.2"
798,"ENSG00000132792.14"
799,"ENSG00000139053.2"
800,"ENSG00000205464.7"
801,"ENSG00000197016.7"
802,"ENSG00000110888.13"
803,"ENSG00000169507.5"
804,"ENSG00000196653.7"
805,"ENSG00000186160.4"
806,"ENSG00000227113.2"
807,"ENSG00000171115.3"
808,"ENSG00000179715.8"
809,"ENSG00000106638.11"
810,"ENSG00000172965.10"
811,"ENSG00000131966.9"
812,"ENSG00000167775.6"
813,"ENSG00000166402.4"
814,"ENSG00000143367.11"
815,"ENSG00000164039.10"
816,"ENSG00000124257.6"

817,"ENSG00000179277.9"
818,"ENSG00000139648.6"
819,"ENSG00000120314.14"
820,"ENSG00000164603.7"
821,"ENSG00000196329.6"
822,"ENSG00000135956.4"
823,"ENSG00000136243.12"
824,"ENSG00000187871.2"
825,"ENSG00000268357.1"
826,"ENSG00000136930.8"
827,"ENSG00000135972.4"
828,"ENSG00000014824.9"
829,"ENSG00000217455.4"
830,"ENSG00000176842.10"
831,"ENSG00000178295.10"
832,"ENSG00000144140.5"
833,"ENSG00000250476.1"
834,"ENSG00000179913.6"
835,"ENSG00000121064.8"
836,"ENSG00000101282.4"
837,"ENSG00000226002.1"
838,"ENSG00000239257.1"
839,"ENSG00000111224.9"
840,"ENSG00000166743.5"
841,"ENSG00000165501.12"
842,"ENSG00000066629.12"
843,"ENSG00000204161.9"
844,"ENSG00000140511.7"
845,"ENSG00000148943.7"
846,"ENSG00000259070.1"
847,"ENSG00000166471.6"
848,"ENSG00000108950.7"
849,"ENSG00000226491.1"
850,"ENSG00000149930.13"
851,"ENSG00000141646.9"
852,"ENSG00000167637.12"
853,"ENSG00000139679.11"
854,"ENSG00000254038.1"
855,"ENSG00000125637.11"
856,"ENSG00000261455.1"
857,"ENSG00000142188.12"
858,"ENSG00000269558.1"
859,"ENSG00000085382.7"
860,"ENSG00000125629.10"
861,"ENSG00000204397.3"
862,"ENSG00000100106.15"
863,"ENSG00000186446.7"
864,"ENSG00000236411.1"

865,"ENSG00000223496.1"
866,"ENSG00000136271.6"
867,"ENSG00000020922.8"
868,"ENSG00000086289.7"
869,"ENSG00000134824.9"
870,"ENSG00000116984.8"
871,"ENSG00000144891.13"
872,"ENSG00000188242.4"
873,"ENSG00000134343.8"
874,"ENSG00000272341.1"
875,"ENSG00000105137.8"
876,"ENSG00000179772.6"
877,"ENSG00000157103.6"
878,"ENSG00000143156.9"
879,"ENSG00000217027.1"
880,"ENSG00000103599.15"
881,"ENSG00000196465.6"
882,"ENSG00000168661.10"
883,"ENSG00000273100.1"
884,"ENSG00000137767.9"
885,"ENSG00000185651.10"
886,"ENSG00000115290.5"
887,"ENSG00000188921.12"
888,"ENSG00000182264.4"
889,"ENSG00000203276.2"
890,"ENSG00000147649.5"
891,"ENSG00000157837.11"
892,"ENSG00000167178.11"
893,"ENSG00000107643.11"
894,"ENSG00000217330.1"
895,"ENSG00000180279.5"
896,"ENSG00000271889.1"
897,"ENSG00000204967.6"
898,"ENSG00000174595.4"
899,"ENSG00000188112.4"
900,"ENSG00000075618.13"
901,"ENSG00000233231.1"
902,"ENSG00000166173.9"
903,"ENSG00000184371.9"
904,"ENSG00000111832.8"
905,"ENSG00000245651.2"
906,"ENSG00000260877.1"
907,"ENSG00000251600.1"
908,"ENSG00000134109.6"
909,"ENSG00000129048.6"
910,"ENSG00000185499.12"
911,"ENSG00000080608.9"
912,"ENSG00000230733.2"

913,"ENSG00000253485.1"
914,"ENSG00000044115.16"
915,"ENSG00000140043.7"
916,"ENSG00000115380.14"
917,"ENSG00000242142.1"
918,"ENSG00000158473.6"
919,"ENSG00000269097.1"
920,"ENSG00000102683.6"
921,"ENSG00000229390.1"
922,"ENSG00000142185.12"
923,"ENSG00000250378.1"
924,"ENSG00000112599.8"
925,"ENSG00000100296.9"
926,"ENSG00000163814.3"
927,"ENSG00000221821.2"
928,"ENSG00000147813.11"
929,"ENSG00000161265.10"
930,"ENSG00000152582.8"
931,"ENSG00000123992.14"
932,"ENSG00000111341.5"
933,"ENSG00000189306.6"
934,"ENSG00000183230.12"
935,"ENSG00000125388.15"
936,"ENSG00000044459.10"
937,"ENSG00000206120.7"
938,"ENSG00000173960.8"
939,"ENSG00000104915.10"
940,"ENSG00000109265.8"
941,"ENSG00000188004.5"
942,"ENSG00000213639.5"
943,"ENSG00000042429.6"
944,"ENSG00000205863.5"
945,"ENSG00000162384.9"
946,"ENSG00000168612.4"
947,"ENSG00000271948.1"
948,"ENSG00000196917.4"
949,"ENSG00000224172.1"
950,"ENSG00000152689.13"
951,"ENSG00000160360.7"
952,"ENSG00000066455.8"
953,"ENSG00000083097.10"
954,"ENSG00000184792.11"
955,"ENSG00000198130.10"
956,"ENSG00000132932.12"
957,"ENSG00000267056.2"
958,"ENSG00000166839.12"
959,"ENSG00000224397.1"
960,"ENSG00000099960.8"

961,"ENSG00000237491.4"
962,"ENSG00000127586.12"
963,"ENSG00000109171.10"
964,"ENSG00000118939.13"
965,"ENSG00000114853.9"
966,"ENSG00000105928.9"
967,"ENSG00000105708.8"
968,"ENSG00000248540.2"
969,"ENSG00000251369.4"
970,"ENSG00000081019.9"
971,"ENSG00000180884.9"
972,"ENSG00000114805.12"
973,"ENSG00000258568.1"
974,"ENSG00000115145.5"
975,"ENSG00000147457.9"
976,"ENSG00000185596.12"
977,"ENSG00000189280.3"
978,"ENSG00000267289.1"
979,"ENSG00000168672.3"
980,"ENSG00000007312.8"
981,"ENSG00000115257.11"
982,"ENSG00000163864.10"
983,"ENSG00000144035.3"
984,"ENSG00000165202.2"
985,"ENSG00000167528.8"
986,"ENSG00000070061.10"
987,"ENSG00000272512.1"
988,"ENSG00000119778.10"
989,"ENSG00000106400.7"
990,"ENSG00000138193.10"
991,"ENSG00000143595.8"
992,"ENSG00000160352.11"
993,"ENSG00000181826.5"
994,"ENSG00000060491.12"
995,"ENSG00000134001.8"
996,"ENSG00000122548.3"
997,"ENSG00000204438.6"
998,"ENSG00000180096.7"
999,"ENSG00000142973.8"
1000,"ENSG00000214357.4"
1001,"ENSG00000138207.8"
1002,"ENSG00000235821.1"
1003,"ENSG00000241318.3"
1004,"ENSG00000090382.2"
1005,"ENSG00000108465.10"
1006,"ENSG00000157259.6"
1007,"ENSG00000198270.8"
1008,"ENSG00000259479.2"

1009,"ENSG00000132640.10"
1010,"ENSG00000162994.11"
1011,"ENSG00000205309.9"
1012,"ENSG00000138363.10"
1013,"ENSG00000116922.10"
1014,"ENSG00000272221.1"
1015,"ENSG00000172638.8"
1016,"ENSG00000197312.7"
1017,"ENSG00000223891.1"
1018,"ENSG00000142208.11"
1019,"ENSG00000198959.7"
1020,"ENSG00000196391.6"
1021,"ENSG00000087494.11"
1022,"ENSG00000231861.1"
1023,"ENSG00000110104.7"
1024,"ENSG00000240875.1"
1025,"ENSG00000169359.9"
1026,"ENSG00000147996.12"
1027,"ENSG00000087077.7"
1028,"ENSG00000139174.6"
1029,"ENSG00000171860.4"
1030,"ENSG00000137103.12"
1031,"ENSG00000144655.10"
1032,"ENSG00000241679.2"
1033,"ENSG00000204060.4"
1034,"ENSG00000151490.9"
1035,"ENSG00000084444.9"
1036,"ENSG00000160439.11"
1037,"ENSG00000221923.4"
1038,"ENSG00000188991.3"
1039,"ENSG00000128789.16"
1040,"ENSG00000177570.9"
1041,"ENSG00000196730.8"
1042,"ENSG00000169689.10"
1043,"ENSG00000175463.7"
1044,"ENSG00000204525.10"
1045,"ENSG00000151327.8"
1046,"ENSG00000232653.4"
1047,"ENSG00000104964.10"
1048,"ENSG00000127337.2"
1049,"ENSG00000183255.7"
1050,"ENSG00000243414.4"
1051,"ENSG00000205822.6"
1052,"ENSG00000242607.1"
1053,"ENSG00000105520.6"
1054,"ENSG00000230154.1"
1055,"ENSG00000164465.14"
1056,"ENSG00000152784.11"

1057,"ENSG00000213699.4"
1058,"ENSG00000214871.3"
1059,"ENSG00000270228.1"
1060,"ENSG00000214297.3"
1061,"ENSG00000203697.7"
1062,"ENSG00000270777.1"
1063,"ENSG00000172775.12"
1064,"ENSG00000078098.9"
1065,"ENSG00000121410.7"
1066,"ENSG00000117859.14"
1067,"ENSG00000103742.7"
1068,"ENSG00000121691.4"
1069,"ENSG00000196712.12"
1070,"ENSG00000143545.4"
1071,"ENSG00000087191.8"
1072,"ENSG00000166313.14"
1073,"ENSG00000225154.2"
1074,"ENSG00000180190.7"
1075,"ENSG00000270673.1"
1076,"ENSG00000130560.4"
1077,"ENSG00000235231.3"
1078,"ENSG00000134333.9"
1079,"ENSG00000187664.8"
1080,"ENSG00000074047.16"
1081,"ENSG00000146007.6"
1082,"ENSG00000162585.12"
1083,"ENSG00000182463.11"
1084,"ENSG00000198624.8"
1085,"ENSG00000114251.9"
1086,"ENSG00000174007.7"
1087,"ENSG00000216775.2"
1088,"ENSG00000261556.4"
1089,"ENSG00000152348.11"
1090,"ENSG00000260423.1"
1091,"ENSG00000051108.10"
1092,"ENSG00000141738.9"
1093,"ENSG00000266970.1"
1094,"ENSG00000251688.1"
1095,"ENSG00000188626.5"
1096,"ENSG00000100348.5"
1097,"ENSG00000130303.8"
1098,"ENSG00000198633.6"
1099,"ENSG00000088325.11"
1100,"ENSG00000159733.9"
1101,"ENSG00000205571.8"
1102,"ENSG00000151365.2"
1103,"ENSG00000063241.3"
1104,"ENSG00000267079.1"

1105,"ENSG00000068028.13"
1106,"ENSG00000157322.12"
1107,"ENSG00000250620.1"
1108,"ENSG00000152558.10"
1109,"ENSG00000138375.8"
1110,"ENSG00000176998.3"
1111,"ENSG00000232956.4"
1112,"ENSG00000172006.7"
1113,"ENSG00000229108.1"
1114,"ENSG00000257431.1"
1115,"ENSG00000148814.13"
1116,"ENSG00000176463.9"
1117,"ENSG00000136573.8"
1118,"ENSG00000112290.8"
1119,"ENSG00000164038.10"
1120,"ENSG00000166965.8"
1121,"ENSG00000228914.1"
1122,"ENSG00000167842.11"
1123,"ENSG00000091106.14"
1124,"ENSG00000186472.15"
1125,"ENSG00000173372.12"
1126,"ENSG00000172955.13"
1127,"ENSG00000271912.1"
1128,"ENSG00000168992.3"
1129,"ENSG00000170581.9"
1130,"ENSG00000111879.14"
1131,"ENSG00000214087.4"
1132,"ENSG00000167653.4"
1133,"ENSG00000225101.3"
1134,"ENSG00000118526.6"
1135,"ENSG00000119929.8"
1136,"ENSG00000229186.3"
1137,"ENSG00000163016.5"
1138,"ENSG00000105186.10"
1139,"ENSG00000176783.10"
1140,"ENSG00000139780.7"
1141,"ENSG00000112149.5"
1142,"ENSG00000100227.13"
1143,"ENSG00000168763.11"
1144,"ENSG00000254681.2"
1145,"ENSG00000242485.1"
1146,"ENSG00000260807.2"
1147,"ENSG00000197608.7"
1148,"ENSG00000185669.5"
1149,"ENSG00000053372.4"
1150,"ENSG00000114978.13"
1151,"ENSG00000158470.5"
1152,"ENSG00000104998.2"

1153,"ENSG00000090060.13"
1154,"ENSG00000168916.11"
1155,"ENSG00000100053.5"
1156,"ENSG00000239272.1"
1157,"ENSG00000101134.7"
1158,"ENSG00000204965.4"
1159,"ENSG00000248994.1"
1160,"ENSG00000204410.10"
1161,"ENSG00000165475.9"
1162,"ENSG00000113273.11"
1163,"ENSG00000206562.7"
1164,"ENSG00000243795.1"
1165,"ENSG00000132205.6"
1166,"ENSG00000163029.11"
1167,"ENSG00000244560.2"
1168,"ENSG00000269305.1"
1169,"ENSG00000113716.8"
1170,"ENSG00000134216.14"
1171,"ENSG00000197980.7"
1172,"ENSG00000231453.1"
1173,"ENSG00000126870.11"
1174,"ENSG00000243678.7"
1175,"ENSG00000108604.11"
1176,"ENSG00000114626.13"
1177,"ENSG00000102804.10"
1178,"ENSG00000163431.11"
1179,"ENSG00000241057.1"
1180,"ENSG00000119718.6"
1181,"ENSG00000105738.6"
1182,"ENSG00000141933.8"
1183,"ENSG00000131943.13"
1184,"ENSG00000128645.11"
1185,"ENSG00000105669.8"
1186,"ENSG00000159899.10"
1187,"ENSG00000196456.4"
1188,"ENSG00000104660.13"
1189,"ENSG00000197226.8"
1190,"ENSG00000184967.2"
1191,"ENSG00000226792.2"
1192,"ENSG00000204301.5"
1193,"ENSG00000119673.10"
1194,"ENSG00000206113.3"
1195,"ENSG00000173821.15"
1196,"ENSG00000228421.2"
1197,"ENSG00000105953.10"
1198,"ENSG00000255260.1"
1199,"ENSG00000049167.9"
1200,"ENSG00000223922.1"

1201,"ENSG00000164164.11"
1202,"ENSG00000166035.6"
1203,"ENSG00000181781.5"
1204,"ENSG00000163933.5"
1205,"ENSG00000178055.8"
1206,"ENSG00000114378.12"
1207,"ENSG00000146707.10"
1208,"ENSG00000240163.1"
1209,"ENSG00000154252.11"
1210,"ENSG00000115825.5"
1211,"ENSG00000126088.8"
1212,"ENSG00000184897.4"
1213,"ENSG00000248610.1"
1214,"ENSG00000099250.13"
1215,"ENSG00000125485.13"
1216,"ENSG00000172590.14"
1217,"ENSG00000132141.9"
1218,"ENSG00000235358.1"
1219,"ENSG00000247317.3"
1220,"ENSG00000233448.2"
1221,"ENSG00000140265.8"
1222,"ENSG00000095917.9"
1223,"ENSG00000176974.13"
1224,"ENSG00000108107.8"
1225,"ENSG00000056661.9"
1226,"ENSG00000151502.6"
1227,"ENSG00000204653.5"
1228,"ENSG00000146842.12"
1229,"ENSG00000107625.8"
1230,"ENSG00000134802.13"
1231,"ENSG00000146147.10"
1232,"ENSG00000196950.9"
1233,"ENSG00000213025.2"
1234,"ENSG00000123908.7"
1235,"ENSG00000166167.13"
1236,"ENSG00000248538.2"
1237,"ENSG00000179242.11"
1238,"ENSG00000266946.1"
1239,"ENSG00000090372.10"
1240,"ENSG00000124215.12"
1241,"ENSG00000173905.4"
1242,"ENSG00000244921.2"
1243,"ENSG00000205918.5"
1244,"ENSG00000172183.10"
1245,"ENSG00000138101.14"
1246,"ENSG00000166136.11"
1247,"ENSG00000079257.3"
1248,"ENSG00000101574.10"

1249,"ENSG00000271095.1"
1250,"ENSG00000214253.4"
1251,"ENSG00000204531.11"
1252,"ENSG00000236611.1"
1253,"ENSG00000173611.13"
1254,"ENSG00000175711.4"
1255,"ENSG00000171533.7"
1256,"ENSG00000161179.9"
1257,"ENSG00000269165.1"
1258,"ENSG00000267596.1"
1259,"ENSG00000268836.1"
1260,"ENSG00000100767.11"
1261,"ENSG00000240089.2"
1262,"ENSG00000162738.5"
1263,"ENSG00000256092.2"
1264,"ENSG00000105880.4"
1265,"ENSG00000082258.8"
1266,"ENSG00000182397.10"
1267,"ENSG00000183784.5"
1268,"ENSG00000106823.8"
1269,"ENSG00000160932.6"
1270,"ENSG00000137288.5"
1271,"ENSG00000272440.1"
1272,"ENSG00000198576.2"
1273,"ENSG00000116138.8"
1274,"ENSG00000087111.16"
1275,"ENSG00000136274.8"
1276,"ENSG00000186153.12"
1277,"ENSG00000129244.4"
1278,"ENSG00000188732.6"
1279,"ENSG00000175764.10"
1280,"ENSG00000235602.4"
1281,"ENSG00000112053.9"
1282,"ENSG00000136104.14"
1283,"ENSG00000169994.14"
1284,"ENSG00000111087.5"
1285,"ENSG00000100024.10"
1286,"ENSG00000261011.1"
1287,"ENSG00000126464.9"
1288,"ENSG00000246596.2"
1289,"ENSG00000242267.2"
1290,"ENSG00000140694.12"
1291,"ENSG00000187764.7"
1292,"ENSG00000100147.9"
1293,"ENSG00000213462.4"
1294,"ENSG00000184990.8"
1295,"ENSG00000213066.7"
1296,"ENSG00000027001.7"

1297,"ENSG00000237310.1"
1298,"ENSG00000260784.1"
1299,"ENSG00000070614.10"
1300,"ENSG00000184840.7"
1301,"ENSG00000060642.6"
1302,"ENSG00000108384.10"
1303,"ENSG00000268996.1"
1304,"ENSG00000272180.1"
1305,"ENSG00000273172.1"
1306,"ENSG00000149474.9"
1307,"ENSG00000154589.2"
1308,"ENSG00000171446.6"
1309,"ENSG00000262165.1"
1310,"ENSG00000135773.8"
1311,"ENSG00000256427.1"
1312,"ENSG00000165061.10"
1313,"ENSG00000109332.15"
1314,"ENSG00000255465.3"
1315,"ENSG00000273258.1"
1316,"ENSG00000185885.11"
1317,"ENSG00000260953.1"
1318,"ENSG00000143994.9"
1319,"ENSG00000124226.7"
1320,"ENSG00000197681.8"
1321,"ENSG00000258713.2"
1322,"ENSG00000174353.13"
1323,"ENSG00000212807.1"
1324,"ENSG00000225418.1"
1325,"ENSG00000120805.9"
1326,"ENSG00000130511.11"
1327,"ENSG00000205339.5"
1328,"ENSG00000250971.1"
1329,"ENSG00000198010.7"
1330,"ENSG00000130023.11"
1331,"ENSG00000230239.1"
1332,"ENSG00000114395.6"
1333,"ENSG00000264514.1"
1334,"ENSG00000153993.9"
1335,"ENSG00000154328.11"
1336,"ENSG00000178150.4"
1337,"ENSG00000269113.3"
1338,"ENSG00000186891.9"
1339,"ENSG00000109163.6"
1340,"ENSG00000154767.10"
1341,"ENSG00000260468.1"
1342,"ENSG00000253853.1"
1343,"ENSG00000119915.4"
1344,"ENSG00000254087.3"

1345,"ENSG00000186907.3"
1346,"ENSG00000267855.1"
1347,"ENSG00000110092.3"
1348,"ENSG00000266145.1"
1349,"ENSG00000179588.4"
1350,"ENSG00000146223.10"
1351,"ENSG00000124006.10"
1352,"ENSG00000233926.1"
1353,"ENSG00000270248.1"
1354,"ENSG00000139668.7"
1355,"ENSG00000183733.6"
1356,"ENSG00000272636.1"
1357,"ENSG00000164828.13"
1358,"ENSG00000244142.1"
1359,"ENSG00000259120.2"
1360,"ENSG00000186417.9"
1361,"ENSG00000225506.2"
1362,"ENSG00000224687.1"
1363,"ENSG00000146085.7"
1364,"ENSG00000183281.10"
1365,"ENSG00000128617.2"
1366,"ENSG00000227578.1"
1367,"ENSG00000153531.8"
1368,"ENSG00000115155.12"
1369,"ENSG00000139687.9"
1370,"ENSG00000255408.2"
1371,"ENSG00000099219.9"
1372,"ENSG00000126456.11"
1373,"ENSG00000129195.11"
1374,"ENSG00000196586.9"
1375,"ENSG00000151458.7"
1376,"ENSG00000232931.1"
1377,"ENSG00000169554.12"
1378,"ENSG00000171530.9"
1379,"ENSG00000167232.9"
1380,"ENSG00000066230.6"
1381,"ENSG00000006747.10"
1382,"ENSG00000167461.7"
1383,"ENSG00000141401.7"
1384,"ENSG00000068078.13"
1385,"ENSG00000257595.2"
1386,"ENSG00000272361.1"
1387,"ENSG00000273015.1"
1388,"ENSG00000157045.4"
1389,"ENSG00000181092.5"
1390,"ENSG00000177674.11"
1391,"ENSG00000152433.10"
1392,"ENSG00000147853.12"

1393,"ENSG00000137285.9"
1394,"ENSG00000086598.6"
1395,"ENSG00000270074.1"
1396,"ENSG00000118137.5"
1397,"ENSG00000224939.1"
1398,"ENSG00000130479.6"
1399,"ENSG00000254319.1"
1400,"ENSG00000177595.13"
1401,"ENSG00000164172.14"
1402,"ENSG00000027869.7"
1403,"ENSG00000248626.1"
1404,"ENSG00000167920.4"
1405,"ENSG00000136718.5"
1406,"ENSG00000010292.8"
1407,"ENSG00000231386.1"
1408,"ENSG00000105404.6"
1409,"ENSG00000239839.1"
1410,"ENSG00000231027.1"
1411,"ENSG00000181027.6"
1412,"ENSG00000227531.1"
1413,"ENSG00000187609.11"
1414,"ENSG00000263470.1"
1415,"ENSG00000170356.8"
1416,"ENSG00000176268.5"
1417,"ENSG00000259494.1"
1418,"ENSG00000227066.1"
1419,"ENSG00000235241.1"
1420,"ENSG00000187045.12"
1421,"ENSG00000138030.8"
1422,"ENSG00000135472.4"
1423,"ENSG00000272005.1"
1424,"ENSG00000233266.1"
1425,"ENSG00000186523.10"
1426,"ENSG00000106536.15"
1427,"ENSG00000143954.8"
1428,"ENSG00000167216.12"
1429,"ENSG00000109911.13"
1430,"ENSG00000230438.5"
1431,"ENSG00000148297.11"
1432,"ENSG00000237988.2"
1433,"ENSG00000116044.11"
1434,"ENSG00000120725.8"
1435,"ENSG00000064787.8"
1436,"ENSG00000213593.5"
1437,"ENSG00000270810.1"
1438,"ENSG00000176358.11"
1439,"ENSG00000079739.11"
1440,"ENSG00000172748.8"

1441,"ENSG00000131203.8"
1442,"ENSG00000101343.10"
1443,"ENSG00000244301.1"
1444,"ENSG00000232098.2"
1445,"ENSG00000013561.13"
1446,"ENSG00000131495.4"
1447,"ENSG00000069998.8"
1448,"ENSG00000160752.10"
1449,"ENSG00000253745.1"
1450,"ENSG00000115216.9"
1451,"ENSG00000185022.7"
1452,"ENSG00000268535.1"
1453,"ENSG00000272047.1"
1454,"ENSG00000169203.12"
1455,"ENSG00000243811.3"
1456,"ENSG00000226816.2"
1457,"ENSG00000164344.11"
1458,"ENSG00000213612.3"
1459,"ENSG00000160888.6"
1460,"ENSG00000198830.6"
1461,"ENSG00000255284.1"
1462,"ENSG00000108298.5"
1463,"ENSG00000120519.10"
1464,"ENSG00000027847.9"
1465,"ENSG00000100418.7"
1466,"ENSG00000184313.15"
1467,"ENSG00000104388.10"
1468,"ENSG00000115816.9"
1469,"ENSG00000224307.1"
1470,"ENSG00000242282.2"
1471,"ENSG00000171759.4"
1472,"ENSG00000196262.9"
1473,"ENSG00000157992.8"
1474,"ENSG00000228696.4"
1475,"ENSG00000101695.4"
1476,"ENSG00000151806.9"
1477,"ENSG00000090432.5"
1478,"ENSG00000183066.10"
1479,"ENSG00000188573.7"
1480,"ENSG00000267575.2"
1481,"ENSG00000204323.5"
1482,"ENSG00000166908.13"
1483,"ENSG00000182534.9"
1484,"ENSG00000236039.1"
1485,"ENSG00000159267.10"
1486,"ENSG00000106785.10"
1487,"ENSG00000182372.6"
1488,"ENSG00000168000.10"

1489,"ENSG00000037280.11"
1490,"ENSG00000196209.8"
1491,"ENSG00000138041.11"
1492,"ENSG00000119686.5"
1493,"ENSG00000171160.13"
1494,"ENSG00000130244.8"
1495,"ENSG00000125633.6"
1496,"ENSG00000267534.1"
1497,"ENSG00000107779.7"
1498,"ENSG00000109743.6"
1499,"ENSG00000110218.4"
1500,"ENSG00000254531.1"
1501,"ENSG00000221990.2"
1502,"ENSG00000232063.1"
1503,"ENSG00000152254.6"
1504,"ENSG00000088448.10"
1505,"ENSG00000119431.5"
1506,"ENSG00000254838.4"
1507,"ENSG00000123374.6"
1508,"ENSG00000162852.9"
1509,"ENSG00000213830.3"
1510,"ENSG00000186777.7"
1511,"ENSG00000117601.9"
1512,"ENSG00000111231.4"
1513,"ENSG00000021826.10"
1514,"ENSG00000101457.8"
1515,"ENSG00000146476.6"
1516,"ENSG00000084463.3"
1517,"ENSG00000229657.2"
1518,"ENSG00000270876.1"
1519,"ENSG00000251459.1"
1520,"ENSG00000051009.6"
1521,"ENSG00000205047.3"
1522,"ENSG00000206013.2"
1523,"ENSG00000181291.5"
1524,"ENSG00000177236.3"
1525,"ENSG00000177990.7"
1526,"ENSG00000185345.14"
1527,"ENSG00000261037.1"
1528,"ENSG00000128655.12"
1529,"ENSG00000124116.14"
1530,"ENSG00000111405.4"
1531,"ENSG00000149418.6"
1532,"ENSG00000108395.9"
1533,"ENSG00000188316.9"
1534,"ENSG00000160213.5"
1535,"ENSG00000183624.9"
1536,"ENSG00000138777.15"

1537,"ENSG00000183798.4"
1538,"ENSG00000167755.9"
1539,"ENSG00000250942.1"
1540,"ENSG00000169139.7"
1541,"ENSG00000123297.12"
1542,"ENSG00000264015.1"
1543,"ENSG00000133028.6"
1544,"ENSG00000188729.2"
1545,"ENSG00000164983.6"
1546,"ENSG00000160221.12"
1547,"ENSG00000159263.11"
1548,"ENSG00000267676.1"
1549,"ENSG00000132432.9"
1550,"ENSG00000138085.12"
1551,"ENSG00000226363.3"
1552,"ENSG00000184014.3"
1553,"ENSG00000149679.7"
1554,"ENSG00000166888.6"
1555,"ENSG00000213199.3"
1556,"ENSG00000261795.1"
1557,"ENSG00000249664.1"
1558,"ENSG00000215158.5"
1559,"ENSG00000250490.1"
1560,"ENSG00000271303.1"
1561,"ENSG00000071967.7"
1562,"ENSG00000204899.5"
1563,"ENSG00000104313.13"
1564,"ENSG00000163449.6"
1565,"ENSG00000147650.7"
1566,"ENSG00000186522.10"
1567,"ENSG00000116127.13"
1568,"ENSG00000179580.5"
1569,"ENSG00000068885.10"
1570,"ENSG00000182218.5"
1571,"ENSG00000189366.5"
1572,"ENSG00000198874.8"
1573,"ENSG00000040275.12"
1574,"ENSG00000270164.1"
1575,"ENSG00000231848.1"
1576,"ENSG00000271119.1"
1577,"ENSG00000112299.7"
1578,"ENSG00000104219.8"
1579,"ENSG00000187953.6"
1580,"ENSG00000162722.8"
1581,"ENSG00000204963.4"
1582,"ENSG00000005379.11"
1583,"ENSG00000047365.7"
1584,"ENSG00000246523.3"

1585,"ENSG00000205038.7"
1586,"ENSG00000270773.1"
1587,"ENSG00000014914.15"
1588,"ENSG00000182903.11"
1589,"ENSG00000157653.7"
1590,"ENSG00000167280.12"
1591,"ENSG00000115598.5"
1592,"ENSG00000236296.3"
1593,"ENSG00000184995.6"
1594,"ENSG00000156521.9"
1595,"ENSG00000088053.7"
1596,"ENSG00000213171.2"
1597,"ENSG00000231698.2"
1598,"ENSG00000261652.2"
1599,"ENSG00000220583.1"
1600,"ENSG00000123427.11"
1601,"ENSG00000188199.6"
1602,"ENSG00000110057.3"
1603,"ENSG00000213642.3"
1604,"ENSG00000149527.13"
1605,"ENSG00000197822.6"
1606,"ENSG00000121766.10"
1607,"ENSG00000066427.17"
1608,"ENSG00000149488.11"
1609,"ENSG00000234147.1"
1610,"ENSG00000056291.13"
1611,"ENSG00000226822.1"
1612,"ENSG00000185245.6"
1613,"ENSG00000184508.6"
1614,"ENSG00000249911.1"
1615,"ENSG00000157326.14"
1616,"ENSG00000239556.2"
1617,"ENSG00000142920.12"
1618,"ENSG00000251432.2"
1619,"ENSG00000263753.2"
1620,"ENSG00000111237.14"
1621,"ENSG00000189046.6"
1622,"ENSG00000185418.11"
1623,"ENSG00000270210.1"
1624,"ENSG00000235421.1"
1625,"ENSG00000150337.9"
1626,"ENSG00000259774.1"
1627,"ENSG00000162377.4"
1628,"ENSG00000132975.6"
1629,"ENSG00000164972.8"
1630,"ENSG00000124614.9"
1631,"ENSG00000181315.6"
1632,"ENSG00000108771.8"

1633,"ENSG00000144118.9"
1634,"ENSG00000145780.6"
1635,"ENSG00000254353.1"
1636,"ENSG00000180855.11"
1637,"ENSG00000158258.11"
1638,"ENSG00000084676.11"
1639,"ENSG00000073008.10"
1640,"ENSG00000186301.7"
1641,"ENSG00000177590.6"
1642,"ENSG00000271738.1"
1643,"ENSG00000198948.7"
1644,"ENSG00000131389.12"
1645,"ENSG00000249742.1"
1646,"ENSG00000163162.4"
1647,"ENSG00000094804.5"
1648,"ENSG00000211584.9"
1649,"ENSG00000169764.10"
1650,"ENSG00000140505.6"
1651,"ENSG00000136449.9"
1652,"ENSG00000085719.7"
1653,"ENSG00000138696.6"
1654,"ENSG00000236740.2"
1655,"ENSG00000170615.10"
1656,"ENSG00000226180.2"
1657,"ENSG00000127838.9"
1658,"ENSG00000115523.12"
1659,"ENSG00000064666.10"
1660,"ENSG00000074755.10"
1661,"ENSG00000134440.7"
1662,"ENSG00000075420.8"
1663,"ENSG00000253558.1"
1664,"ENSG00000122547.6"
1665,"ENSG00000100243.16"
1666,"ENSG00000039650.5"
1667,"ENSG00000178922.12"
1668,"ENSG00000235374.1"
1669,"ENSG00000272455.1"
1670,"ENSG00000227359.1"
1671,"ENSG00000116663.6"
1672,"ENSG00000104883.3"
1673,"ENSG00000204929.7"
1674,"ENSG00000267108.1"
1675,"ENSG00000197261.7"
1676,"ENSG00000135624.11"
1677,"ENSG00000092529.18"

1,"ENSG00000225127.2"
2,"ENSG00000214900.4"
3,"ENSG00000251247.5"
4,"ENSG00000135702.10"
5,"ENSG00000170571.7"
6,"ENSG00000125510.11"
7,"ENSG00000108381.6"
8,"ENSG00000065665.16"
9,"ENSG00000065457.6"
10,"ENSG00000182054.5"
11,"ENSG00000174004.5"
12,"ENSG00000141255.8"
13,"ENSG00000203880.7"
14,"ENSG00000234232.2"
15,"ENSG00000168778.7"
16,"ENSG00000092094.6"
17,"ENSG00000213985.4"
18,"ENSG00000188868.9"
19,"ENSG00000157823.12"
20,"ENSG00000205084.6"
21,"ENSG00000035687.9"
22,"ENSG00000112992.12"
23,"ENSG00000103148.11"
24,"ENSG00000179218.9"
25,"ENSG00000154719.9"
26,"ENSG00000123143.8"
27,"ENSG00000243566.2"
28,"ENSG00000265933.1"
29,"ENSG00000100994.7"
30,"ENSG00000118058.16"
31,"ENSG00000161944.12"
32,"ENSG00000266173.1"
33,"ENSG00000183486.8"
34,"ENSG00000130758.3"
35,"ENSG00000224557.3"
36,"ENSG00000177025.3"
37,"ENSG00000251532.1"
38,"ENSG00000162604.8"
39,"ENSG00000266935.1"
40,"ENSG00000173548.8"
41,"ENSG00000104450.8"
42,"ENSG00000089327.10"
43,"ENSG00000176095.7"
44,"ENSG00000141577.9"
45,"ENSG00000196922.6"
46,"ENSG00000081320.6"
47,"ENSG00000167562.7"
48,"ENSG00000163728.6"

49,"ENSG00000065060.12"
50,"ENSG00000161180.6"
51,"ENSG00000167617.2"
52,"ENSG00000270020.1"
53,"ENSG00000204778.4"
54,"ENSG00000144134.14"
55,"ENSG00000108666.5"
56,"ENSG00000015133.14"
57,"ENSG00000271581.1"
58,"ENSG00000007516.9"
59,"ENSG00000188599.13"
60,"ENSG00000232721.1"
61,"ENSG00000186642.11"
62,"ENSG00000180385.4"
63,"ENSG00000091490.6"
64,"ENSG00000266648.1"
65,"ENSG00000111300.5"
66,"ENSG00000100116.12"
67,"ENSG00000166407.9"
68,"ENSG00000197062.7"
69,"ENSG00000197157.6"
70,"ENSG00000141337.8"
71,"ENSG00000179776.13"
72,"ENSG00000118004.13"
73,"ENSG00000188493.10"
74,"ENSG00000197279.3"
75,"ENSG00000145029.7"
76,"ENSG00000137824.11"
77,"ENSG00000156876.8"
78,"ENSG00000165806.15"
79,"ENSG00000270547.1"
80,"ENSG00000140263.9"
81,"ENSG00000100156.6"
82,"ENSG00000226942.2"
83,"ENSG00000179698.9"
84,"ENSG00000118507.11"
85,"ENSG00000146909.3"
86,"ENSG00000242498.3"
87,"ENSG00000131697.13"
88,"ENSG00000197586.8"
89,"ENSG00000267018.1"
90,"ENSG00000093167.13"
91,"ENSG00000138600.5"
92,"ENSG00000226629.1"
93,"ENSG00000124875.5"
94,"ENSG00000171700.9"
95,"ENSG00000103035.6"
96,"ENSG00000184207.8"

97,"ENSG00000269552.1"
98,"ENSG00000053108.12"
99,"ENSG00000114670.9"
100,"ENSG00000269067.1"
101,"ENSG00000182103.3"
102,"ENSG00000182255.6"
103,"ENSG00000104714.9"
104,"ENSG00000250317.4"
105,"ENSG00000178381.7"
106,"ENSG00000127957.12"
107,"ENSG00000151690.10"
108,"ENSG00000126467.6"
109,"ENSG00000117090.10"
110,"ENSG00000170954.7"
111,"ENSG00000248587.2"
112,"ENSG00000198750.7"
113,"ENSG00000164889.8"
114,"ENSG00000171103.6"
115,"ENSG00000133704.5"
116,"ENSG00000230305.2"
117,"ENSG00000100234.11"
118,"ENSG00000090054.9"
119,"ENSG00000105497.3"
120,"ENSG00000072736.14"
121,"ENSG00000204351.7"
122,"ENSG00000224132.2"
123,"ENSG00000143702.11"
124,"ENSG00000260454.1"
125,"ENSG00000133243.4"
126,"ENSG00000151665.8"
127,"ENSG00000134962.6"
128,"ENSG00000108244.12"
129,"ENSG00000185040.11"
130,"ENSG00000188295.10"
131,"ENSG00000113119.8"
132,"ENSG00000122085.12"
133,"ENSG00000256673.1"
134,"ENSG00000182685.3"
135,"ENSG00000078053.12"

1,"ENSG00000269051.1"
2,"ENSG00000229694.2"
3,"ENSG00000204709.4"
4,"ENSG00000263508.1"
5,"ENSG00000162704.11"
6,"ENSG00000255298.2"
7,"ENSG00000186814.8"
8,"ENSG00000205057.3"
9,"ENSG00000271390.1"
10,"ENSG00000241106.2"
11,"ENSG00000101197.8"
12,"ENSG00000120306.5"
13,"ENSG00000179397.13"
14,"ENSG00000166359.6"
15,"ENSG00000229212.3"
16,"ENSG00000163684.7"
17,"ENSG00000169116.7"
18,"ENSG00000084090.9"
19,"ENSG00000122873.7"
20,"ENSG00000272316.1"
21,"ENSG00000134996.11"
22,"ENSG00000156162.12"
23,"ENSG00000172466.11"
24,"ENSG00000178075.15"
25,"ENSG00000204267.9"
26,"ENSG00000177483.7"
27,"ENSG00000162614.14"
28,"ENSG00000258359.1"
29,"ENSG00000155660.6"
30,"ENSG00000146109.3"
31,"ENSG00000100889.7"
32,"ENSG00000235641.3"
33,"ENSG00000076554.11"
34,"ENSG00000162998.4"
35,"ENSG00000225706.1"
36,"ENSG00000116701.10"
37,"ENSG00000100554.7"
38,"ENSG00000111850.6"
39,"ENSG00000180773.10"
40,"ENSG00000267838.1"
41,"ENSG00000166341.6"
42,"ENSG00000213221.4"
43,"ENSG00000154127.5"
44,"ENSG00000116260.12"
45,"ENSG00000174325.4"
46,"ENSG00000185339.4"
47,"ENSG00000213906.5"
48,"ENSG00000188282.8"

49,"ENSG00000177946.4"
50,"ENSG00000105894.7"
51,"ENSG00000071564.10"
52,"ENSG00000181555.15"
53,"ENSG00000176635.13"
54,"ENSG00000152487.6"
55,"ENSG00000265139.1"
56,"ENSG00000254333.1"
57,"ENSG00000186150.3"
58,"ENSG00000166913.8"
59,"ENSG00000269001.1"
60,"ENSG00000176101.7"
61,"ENSG00000073331.13"
62,"ENSG00000182511.7"
63,"ENSG00000132016.7"
64,"ENSG00000133466.9"
65,"ENSG00000242220.2"
66,"ENSG00000170260.4"
67,"ENSG00000257315.1"
68,"ENSG00000188738.9"
69,"ENSG00000197948.6"
70,"ENSG00000054219.9"
71,"ENSG00000197768.6"
72,"ENSG00000241058.1"
73,"ENSG00000061936.5"
74,"ENSG00000153814.7"
75,"ENSG00000161281.6"
76,"ENSG00000239650.3"
77,"ENSG00000267013.1"
78,"ENSG00000111142.9"
79,"ENSG00000112799.4"
80,"ENSG00000004468.8"
81,"ENSG00000229205.2"
82,"ENSG00000129467.9"
83,"ENSG00000108064.6"
84,"ENSG00000064545.10"
85,"ENSG00000133313.10"
86,"ENSG00000272541.1"
87,"ENSG00000272091.1"
88,"ENSG00000230147.2"
89,"ENSG00000223561.2"
90,"ENSG00000170561.8"
91,"ENSG00000165752.12"
92,"ENSG00000151883.12"
93,"ENSG00000204348.5"
94,"ENSG00000185324.17"
95,"ENSG00000262583.1"
96,"ENSG00000178802.13"

97,"ENSG00000143315.5"
98,"ENSG00000184752.8"
99,"ENSG00000167972.9"
100,"ENSG00000102699.5"
101,"ENSG00000124508.12"
102,"ENSG00000179115.6"
103,"ENSG00000091436.12"
104,"ENSG00000162729.9"
105,"ENSG00000162458.8"
106,"ENSG00000114770.12"
107,"ENSG00000182606.10"
108,"ENSG00000249601.2"
109,"ENSG00000187151.3"
110,"ENSG00000165478.6"
111,"ENSG00000177425.6"
112,"ENSG00000229891.1"
113,"ENSG00000168291.8"
114,"ENSG00000265750.1"
115,"ENSG00000082068.4"
116,"ENSG00000100034.9"
117,"ENSG00000146540.10"
118,"ENSG00000169609.9"
119,"ENSG00000213903.4"
120,"ENSG00000148719.10"
121,"ENSG00000196867.3"
122,"ENSG00000260027.3"
123,"ENSG00000152240.8"
124,"ENSG00000242110.3"
125,"ENSG00000154096.9"
126,"ENSG00000127903.12"
127,"ENSG00000204252.8"
128,"ENSG00000144214.5"
129,"ENSG00000169228.9"
130,"ENSG00000227877.2"
131,"ENSG00000170915.8"
132,"ENSG00000258405.5"
133,"ENSG00000104894.7"
134,"ENSG00000075975.11"
135,"ENSG00000233473.2"
136,"ENSG00000104901.2"
137,"ENSG00000225329.1"
138,"ENSG00000205595.3"
139,"ENSG00000172348.10"
140,"ENSG00000148482.7"
141,"ENSG00000166262.11"
142,"ENSG00000182472.4"
143,"ENSG00000197265.4"
144,"ENSG00000257239.1"

145,"ENSG00000137106.13"
146,"ENSG00000171056.6"
147,"ENSG00000242715.3"
148,"ENSG00000215808.2"
149,"ENSG00000186832.4"
150,"ENSG00000259205.2"
151,"ENSG00000226642.1"
152,"ENSG00000250427.1"
153,"ENSG00000247121.2"
154,"ENSG00000107959.11"
155,"ENSG00000167525.9"
156,"ENSG00000171476.17"
157,"ENSG00000136982.5"
158,"ENSG00000164068.11"
159,"ENSG00000089063.10"
160,"ENSG00000104324.11"
161,"ENSG00000260265.1"
162,"ENSG00000178750.2"
163,"ENSG00000270237.1"
164,"ENSG00000010610.5"
165,"ENSG00000251169.2"
166,"ENSG00000132563.11"
167,"ENSG00000256612.3"
168,"ENSG00000156603.10"
169,"ENSG00000126785.8"
170,"ENSG00000258593.2"
171,"ENSG00000254901.3"
172,"ENSG00000158528.7"
173,"ENSG00000106443.10"
174,"ENSG00000128607.9"
175,"ENSG00000150977.9"
176,"ENSG00000216901.1"
177,"ENSG00000139187.5"
178,"ENSG00000188986.4"
179,"ENSG00000220161.4"
180,"ENSG00000256667.2"
181,"ENSG00000133103.12"
182,"ENSG00000264176.1"
183,"ENSG00000271584.1"
184,"ENSG00000146457.10"
185,"ENSG00000165091.11"
186,"ENSG00000228600.1"
187,"ENSG00000223508.5"
188,"ENSG00000079974.13"
189,"ENSG00000076924.7"
190,"ENSG00000197245.4"
191,"ENSG00000254667.1"
192,"ENSG00000134375.6"

193,"ENSG00000133983.10"
194,"ENSG00000146386.7"
195,"ENSG00000251000.1"
196,"ENSG00000270409.1"
197,"ENSG00000198682.8"
198,"ENSG00000270127.1"
199,"ENSG00000170776.15"
200,"ENSG00000175749.11"
201,"ENSG00000254731.1"
202,"ENSG00000177764.6"
203,"ENSG00000113734.13"
204,"ENSG00000150403.13"
205,"ENSG00000164924.13"
206,"ENSG00000119669.3"
207,"ENSG00000111186.8"
208,"ENSG00000126500.3"
209,"ENSG00000131778.13"
210,"ENSG00000105270.10"
211,"ENSG00000243710.3"
212,"ENSG00000178761.10"
213,"ENSG00000128791.7"
214,"ENSG00000166510.9"
215,"ENSG00000222009.4"
216,"ENSG00000038532.10"
217,"ENSG00000270093.1"
218,"ENSG00000234918.1"
219,"ENSG00000198860.7"
220,"ENSG00000163938.12"
221,"ENSG00000164062.8"
222,"ENSG00000187987.5"
223,"ENSG00000131941.3"
224,"ENSG00000162600.7"
225,"ENSG00000122870.7"
226,"ENSG00000115750.12"
227,"ENSG00000163155.7"
228,"ENSG00000063127.11"
229,"ENSG00000223501.4"
230,"ENSG00000137185.7"
231,"ENSG00000272275.1"
232,"ENSG00000165832.4"
233,"ENSG00000136319.7"
234,"ENSG00000103168.12"
235,"ENSG00000130489.8"
236,"ENSG00000106591.3"
237,"ENSG00000185619.13"
238,"ENSG00000108813.9"
239,"ENSG00000100814.13"
240,"ENSG00000237595.2"

241,"ENSG00000088888.13"
242,"ENSG00000273139.1"
243,"ENSG00000131401.7"
244,"ENSG00000100065.10"
245,"ENSG00000100523.10"
246,"ENSG00000226676.1"
247,"ENSG00000105298.9"
248,"ENSG00000167799.5"
249,"ENSG00000151151.5"
250,"ENSG00000155761.9"
251,"ENSG00000226015.2"
252,"ENSG00000164588.4"
253,"ENSG00000158805.7"
254,"ENSG00000180257.8"
255,"ENSG00000188687.11"
256,"ENSG00000230487.3"
257,"ENSG00000228570.3"
258,"ENSG00000272864.1"
259,"ENSG00000108963.13"
260,"ENSG00000079689.9"
261,"ENSG00000132600.12"
262,"ENSG00000204428.8"
263,"ENSG00000105607.8"
264,"ENSG00000124532.10"
265,"ENSG00000161202.13"
266,"ENSG00000231742.1"
267,"ENSG00000009950.11"
268,"ENSG00000188517.10"
269,"ENSG00000176681.10"
270,"ENSG00000072163.14"
271,"ENSG00000272523.1"
272,"ENSG00000148296.5"
273,"ENSG00000225490.1"
274,"ENSG00000187984.8"
275,"ENSG00000168404.8"
276,"ENSG00000164944.7"
277,"ENSG00000119698.7"
278,"ENSG00000204228.3"
279,"ENSG00000142230.7"
280,"ENSG00000173540.8"
281,"ENSG00000184602.5"
282,"ENSG00000143434.11"
283,"ENSG00000008441.12"
284,"ENSG00000161203.9"
285,"ENSG00000150867.9"
286,"ENSG00000267939.1"
287,"ENSG00000213983.7"
288,"ENSG00000114446.4"

289,"ENSG00000162076.8"
290,"ENSG00000109113.13"
291,"ENSG00000119326.10"
292,"ENSG00000269973.1"
293,"ENSG00000023445.9"
294,"ENSG00000175395.11"
295,"ENSG00000126749.10"
296,"ENSG00000125826.15"
297,"ENSG00000108511.8"
298,"ENSG00000260075.1"
299,"ENSG00000250412.1"
300,"ENSG00000267088.1"
301,"ENSG00000176834.9"
302,"ENSG00000271590.1"
303,"ENSG00000214826.4"
304,"ENSG00000175003.8"
305,"ENSG00000113558.14"
306,"ENSG00000157017.11"
307,"ENSG00000138050.10"
308,"ENSG00000137806.4"
309,"ENSG00000148832.10"
310,"ENSG00000104129.5"
311,"ENSG00000176209.7"
312,"ENSG00000224014.1"
313,"ENSG00000197951.4"
314,"ENSG00000271538.1"
315,"ENSG00000143811.12"
316,"ENSG00000272360.1"
317,"ENSG00000101198.10"
318,"ENSG00000231702.2"
319,"ENSG00000137714.2"
320,"ENSG00000251383.1"
321,"ENSG00000249258.2"
322,"ENSG00000155016.13"
323,"ENSG00000196961.8"
324,"ENSG00000160953.10"
325,"ENSG00000237624.1"
326,"ENSG00000106610.10"
327,"ENSG00000221823.6"
328,"ENSG00000145725.15"
329,"ENSG00000115295.15"
330,"ENSG00000140320.7"
331,"ENSG00000260804.2"
332,"ENSG00000080345.13"
333,"ENSG00000063245.10"
334,"ENSG00000168995.9"
335,"ENSG00000151692.10"
336,"ENSG00000170647.2"

337,"ENSG00000259630.2"
338,"ENSG00000254772.5"
339,"ENSG00000178538.5"
340,"ENSG00000140612.9"
341,"ENSG00000143921.6"
342,"ENSG00000197045.8"
343,"ENSG00000177311.6"
344,"ENSG00000124299.9"
345,"ENSG00000196821.5"
346,"ENSG00000244480.1"
347,"ENSG00000184206.7"
348,"ENSG00000144230.12"
349,"ENSG00000110717.6"
350,"ENSG00000163568.9"
351,"ENSG00000162545.5"
352,"ENSG00000173805.11"
353,"ENSG00000196663.11"
354,"ENSG00000165684.3"
355,"ENSG00000113068.5"
356,"ENSG00000120093.7"
357,"ENSG00000163554.7"
358,"ENSG00000133101.5"
359,"ENSG00000129455.11"
360,"ENSG00000105982.12"
361,"ENSG00000132823.6"
362,"ENSG00000109929.5"
363,"ENSG00000157150.4"
364,"ENSG00000076650.2"
365,"ENSG00000272690.1"
366,"ENSG00000161609.5"
367,"ENSG00000110063.4"
368,"ENSG00000188163.6"
369,"ENSG00000160321.10"
370,"ENSG00000233916.1"
371,"ENSG00000108219.10"
372,"ENSG00000170092.10"
373,"ENSG00000198093.6"
374,"ENSG00000173145.7"
375,"ENSG00000175305.12"
376,"ENSG00000177432.6"
377,"ENSG00000244026.2"
378,"ENSG00000234073.1"
379,"ENSG00000087206.12"
380,"ENSG00000198919.8"
381,"ENSG00000152223.8"
382,"ENSG00000077238.9"
383,"ENSG00000138459.4"
384,"ENSG00000272444.1"

385,"ENSG00000124678.13"
386,"ENSG00000113621.10"
387,"ENSG00000245468.3"
388,"ENSG00000078898.6"
389,"ENSG00000219435.3"
390,"ENSG00000231952.3"
391,"ENSG00000185838.9"
392,"ENSG00000129480.8"
393,"ENSG00000237419.1"
394,"ENSG00000244753.2"
395,"ENSG00000162366.3"
396,"ENSG00000258231.1"
397,"ENSG00000173214.5"
398,"ENSG00000104331.4"
399,"ENSG00000145439.7"
400,"ENSG00000114904.8"
401,"ENSG00000164136.12"
402,"ENSG00000230611.1"
403,"ENSG00000196263.3"
404,"ENSG00000089169.10"
405,"ENSG00000254685.2"
406,"ENSG00000219200.6"
407,"ENSG00000133597.5"
408,"ENSG00000273033.1"
409,"ENSG00000137672.8"
410,"ENSG00000137275.9"
411,"ENSG00000230772.1"
412,"ENSG00000033100.10"
413,"ENSG00000260973.1"
414,"ENSG00000204767.3"
415,"ENSG00000145824.8"
416,"ENSG00000181523.8"
417,"ENSG00000104522.11"
418,"ENSG00000050327.10"
419,"ENSG00000112137.12"
420,"ENSG00000134873.5"
421,"ENSG00000063438.12"
422,"ENSG00000149743.9"
423,"ENSG00000163904.8"
424,"ENSG00000072694.14"
425,"ENSG00000170689.8"
426,"ENSG00000204843.8"
427,"ENSG00000171421.8"
428,"ENSG00000196199.9"
429,"ENSG00000204592.5"
430,"ENSG00000184115.12"
431,"ENSG00000213853.5"
432,"ENSG00000137513.5"

433,"ENSG00000111271.10"
434,"ENSG00000099251.10"
435,"ENSG00000129484.9"
436,"ENSG00000064309.10"
437,"ENSG00000178397.8"
438,"ENSG00000226874.1"
439,"ENSG00000169035.7"
440,"ENSG00000178425.9"
441,"ENSG00000078269.9"
442,"ENSG00000182141.5"
443,"ENSG00000141639.7"
444,"ENSG00000196167.5"
445,"ENSG00000102580.10"
446,"ENSG00000230373.4"
447,"ENSG00000250778.1"
448,"ENSG00000185742.6"
449,"ENSG00000125144.9"
450,"ENSG00000205583.9"
451,"ENSG00000166825.9"
452,"ENSG00000180353.6"
453,"ENSG00000100129.13"
454,"ENSG00000188707.4"
455,"ENSG00000248290.1"
456,"ENSG00000187824.4"
457,"ENSG00000272777.1"
458,"ENSG00000108799.8"
459,"ENSG00000203872.6"
460,"ENSG00000101306.6"
461,"ENSG00000088833.13"
462,"ENSG00000113719.11"
463,"ENSG00000225422.3"
464,"ENSG00000148908.10"
465,"ENSG00000204920.6"
466,"ENSG00000164342.8"
467,"ENSG00000227755.1"
468,"ENSG00000090857.9"
469,"ENSG00000247157.2"
470,"ENSG00000258429.1"
471,"ENSG00000260459.2"
472,"ENSG00000121406.4"
473,"ENSG00000124357.8"
474,"ENSG00000225507.1"
475,"ENSG00000151893.10"
476,"ENSG00000178917.10"
477,"ENSG00000185880.8"
478,"ENSG00000119446.9"
479,"ENSG00000058085.10"
480,"ENSG00000115310.13"

481,"ENSG00000272902.1"
482,"ENSG00000175220.7"
483,"ENSG00000132128.12"
484,"ENSG00000187634.6"
485,"ENSG00000122643.14"
486,"ENSG00000154122.8"
487,"ENSG00000272130.1"
488,"ENSG00000259005.1"
489,"ENSG00000163331.6"
490,"ENSG00000016602.8"
491,"ENSG00000156170.8"
492,"ENSG00000196460.8"
493,"ENSG00000213453.3"
494,"ENSG00000114841.13"
495,"ENSG00000227671.3"
496,"ENSG00000227558.4"
497,"ENSG00000197614.6"
498,"ENSG00000125912.6"
499,"ENSG00000106070.13"
500,"ENSG00000021762.15"
501,"ENSG00000272195.1"
502,"ENSG00000152601.13"
503,"ENSG00000180881.15"
504,"ENSG00000113946.3"
505,"ENSG00000178226.6"
506,"ENSG00000170837.2"
507,"ENSG00000160325.10"
508,"ENSG00000071243.11"
509,"ENSG00000214402.6"
510,"ENSG00000101158.8"
511,"ENSG00000132406.7"
512,"ENSG00000172014.11"
513,"ENSG00000163737.3"
