# Supplementary material for: The transcriptome-wide association search for genes and genetic variants which associate with BMI and gestational weight gain in women with type 1 diabetes
Source: Mol Med. 2021 Jan 20;27:6. doi: 10.1186/s10020-020-00266-z (PMC7818927; doi:10.1186/s10020-020-00266-z)
Supplement: Supplementary file 9 — Additional file 9: Table S6. The list of variants associated with GWG in BMI associated genes in T2D&ARIC cohorts. [file 10020_2020_266_MOESM9_ESM.pdf]

snpID	n	MAF	Est	SE	Wald.Stat	Wald.pval
rs621846	316	0.42405063	-1.552104	0.464081	11.18546	0.0008244077
rs11585118	316	0.17879747	-2.03711	0.6090763	11.18627	0.0008240477
rs10493173	316	0.125	-2.580306	0.7105768	13.18622	0.0002820145
rs702491	316	0.17879747	-2.146745	0.6190861	12.02427	0.0005251227
rs959518	316	0.11867089	2.429284	0.7230337	11.28859	0.0007798507
rs678456	316	0.08386076	3.078746	0.8327461	13.66855	0.0002180762
rs978763	316	0.10917722	-2.348772	0.7001947	11.25237	0.0007952147
rs6665548	316	0.25	-1.693111	0.5131295	10.88722	0.0009683011
rs6702384	316	0.45396825	1.583314	0.4620104	11.7444	0.0006102666
rs12060035	316	0.13132911	-2.494724	0.6849298	13.26639	0.0002702069
rs851351	316	0.26582278	1.702315	0.5154805	10.90574	0.0009586665
rs851436	316	0.26582278	1.726331	0.5152062	11.22758	0.0008059077
rs12987284	316	0.23101266	1.834595	0.5495549	11.14443	0.0008428425
rs6747327	316	0.35601266	2.036711	0.468009	18.93872	1.349851E-05
rs6548032	316	0.25949367	1.973582	0.5429384	13.21324	0.0002779784
rs17324843	316	0.29272152	1.765985	0.5099903	11.99085	0.0005346234
rs4952179	316	0.24841772	1.864245	0.5339735	12.18895	0.0004807334
rs1568403	316	0.30063291	1.745458	0.5139835	11.53242	0.0006839297
rs1520321	316	0.37025316	1.79297	0.4724982	14.39945	0.0001478457
rs4597573	316	0.36550633	1.775802	0.4733124	14.07646	0.0001755268
rs4597574	316	0.37025316	1.79297	0.4724982	14.39945	0.0001478457
rs840952	316	0.48734177	1.570343	0.4339844	13.09304	0.0002963945
rs10196975	316	0.09493671	2.637602	0.7364717	12.82645	0.0003417545
rs6546217	316	0.11708861	2.637946	0.6775753	15.15712	9.892482E-05
rs7609526	316	0.11075949	2.679548	0.6886125	15.14165	9.973858E-05
rs9309384	316	0.10601266	2.623875	0.7081573	13.72862	0.0002112115
rs4849630	316	0.15981013	-2.192236	0.5858343	14.00313	0.0001825066
rs1439877	316	0.23575949	1.811162	0.524657	11.91691	0.0005562656
rs11680615	316	0.22468354	1.918587	0.5349857	12.86111	0.0003354812
rs1439887	316	0.24367089	1.769877	0.5237252	11.42034	0.0007264442
rs7424417	316	0.16297468	2.167374	0.5843778	13.75563	0.0002081959
rs17709220	316	0.18037975	2.107084	0.564128	13.95109	0.0001876284
rs7564856	316	0.34493671	-1.594202	0.4828842	10.89935	0.0009619777
rs1541725	316	0.46031746	1.609382	0.4541729	12.5567	0.0003947876
rs9833854	316	0.33702532	-1.568948	0.4755667	10.88415	0.0009699086
rs6769400	316	0.46360759	-1.661965	0.4542471	13.38627	0.0002534736
rs11714248	316	0.43037975	-1.860051	0.4552677	16.69229	4.395933E-05
rs11926768	316	0.44533762	-1.615852	0.4468906	13.07377	0.0002994597
rs6768108	316	0.44462025	-1.643018	0.4481933	13.4386	0.0002464995
rs7611106	316	0.44462025	-1.570544	0.4513008	12.11066	0.0005013434
rs1046512	316	0.44462025	-1.570544	0.4513008	12.11066	0.0005013434
rs3172297	316	0.44462025	-1.570544	0.4513008	12.11066	0.0005013434
rs1799977	316	0.32120253	-1.600973	0.4826939	11.00079	0.0009107282
rs1799977	316	0.32120253	-1.600973	0.4826939	11.00079	0.0009107282
rs1558529	316	0.44462025	-1.570544	0.4513008	12.11066	0.0005013434
rs9846039	316	0.44462025	-1.570544	0.4513008	12.11066	0.0005013434
rs6809297	316	0.44462025	-1.570544	0.4513008	12.11066	0.0005013434
rs6550457	316	0.44462025	-1.570544	0.4513008	12.11066	0.0005013434
rs10154916	316	0.44462025	-1.570544	0.4513008	12.11066	0.0005013434
rs9869432	316	0.44462025	-1.570544	0.4513008	12.11066	0.0005013434
rs1398095	316	0.25632911	2.14798	0.528676	16.50749	4.845817E-05
rs2669833	316	0.11550633	-2.436516	0.7053998	11.93076	0.0005521474

rs9876387	316	0.33702532	1.729647	0.4933783	12.29009	0.0004553708
rs9993370	316	0.45094937	-1.573198	0.4642435	11.48351	0.0007021622
rs1465496	316	0.16349206	2.095495	0.6088748	11.84451	0.0005783158
rs6815557	316	0.39240506	1.819159	0.4620436	15.50157	8.243671E-05
rs1372088	316	0.09968354	-2.493294	0.7494627	11.06743	0.0008785724
rs7668422	316	0.12658228	-2.44271	0.6831079	12.7869	0.0003490546
rs13101487	316	0.09968354	-2.493294	0.7494627	11.06743	0.0008785724
rs11097465	316	0.49050633	-1.704944	0.4429809	14.81325	0.0001186985
rs17021755	316	0.25949367	1.684546	0.5101181	10.90497	0.0009590669
rs13139212	316	0.16134185	2.042184	0.6005975	11.56173	0.000673232
rs9292503	316	0.38765823	-1.47348	0.4476394	10.83508	0.0009959504
rs2063254	316	0.05696203	-3.281054	0.969504	11.45322	0.0007137023
rs115235886	316	0.06487342	3.106646	0.9340727	11.06171	0.0008812884
rs6892053	316	0.18829114	-1.976708	0.579375	11.64033	0.00064537
rs381658	316	0.41613924	-1.530967	0.4592081	11.11508	0.0008562847
rs4661	316	0.09177215	2.683645	0.8022558	11.18985	0.0008224593
rs2421052	316	0.43037975	-1.855451	0.469069	15.64681	7.634131E-05
rs10476298	316	0.39240506	-1.596676	0.4760945	11.24727	0.0007974007
rs2431423	316	0.34810127	1.775153	0.4755223	13.93573	0.0001891679
rs11249632	316	0.11392405	2.566318	0.7217085	12.64438	0.0003766969
rs9393623	316	0.31012658	2.18292	0.473948	21.21359	4.108419E-06
rs17338569	316	0.12341772	-2.515559	0.680366	13.67048	0.0002178519
rs9348827	316	0.11392405	-2.513897	0.7148816	12.36593	0.0004372404
rs4148876	316	0.13765823	-2.24029	0.6588764	11.56114	0.0006734466
rs154977	316	0.38607595	-1.676775	0.4693828	12.76132	0.0003538619
rs9470848	316	0.30379747	1.624258	0.4854755	11.19374	0.0008207375
rs6905637	316	0.3	1.821733	0.494071	13.59536	0.0002267457
rs13207730	316	0.06962025	-3.164894	0.8991359	12.38989	0.0004316639
rs181710	316	0.45253165	-1.479211	0.4387975	11.36402	0.0007488063
rs243765	316	0.45253165	-1.479211	0.4387975	11.36402	0.0007488063
rs4629710	316	0.17879747	-1.956202	0.5895404	11.01033	0.0009060562
rs3757298	316	0.10576923	-2.724327	0.7765476	12.30785	0.000451056
rs2057181	316	0.39398734	-1.550558	0.4648551	11.12605	0.0008512378
rs9478395	316	0.25158228	-1.764933	0.5310115	11.0471	0.000888258
rs9347707	316	0.35126582	1.942668	0.4775613	16.54775	4.744017E-05
rs4329088	316	0.46202532	1.625315	0.4618746	12.38303	0.0004332539
rs909635	316	0.49683544	1.516776	0.4500583	11.35809	0.0007512
rs2072767	316	0.26424051	1.779887	0.4922738	13.07289	0.0002996012
rs2072767	316	0.26424051	1.779887	0.4922738	13.07289	0.0002996012
rs2528913	316	0.20253165	2.046457	0.5786211	12.50883	0.0004050335
rs2686528	316	0.25316456	2.00512	0.536574	13.96438	0.0001863076
rs1419767	316	0.36392405	1.545076	0.4671045	10.94137	0.0009404045
rs245934	316	0.36507937	1.529189	0.4600033	11.05098	0.000886404
rs245952	316	0.36392405	1.562525	0.461996	11.43873	0.0007192894
rs11761049	316	0.24050633	-1.902492	0.503399	14.28305	0.0001572749
rs10233502	316	0.43037975	-1.555536	0.4571785	11.57679	0.0006677999
rs2392581	316	0.35601266	1.580159	0.4529554	12.17001	0.0004856414
rs1978202	316	0.08702532	3.507174	0.7648172	21.02807	0.000004526
rs13340504	316	0.14398734	2.324001	0.6307253	13.57663	0.000229019
rs11465293	316	0.05696203	4.45694	0.9565992	21.70768	3.175173E-06
rs11465293	316	0.05696203	4.45694	0.9565992	21.70768	3.175173E-06
rs205763	316	0.16772152	2.122136	0.5982532	12.58278	0.000389318
rs9690213	316	0.1835443	2.675336	0.5466046	23.95577	9.857424E-07

rs12534221	316	0.17721519	2.553932	0.563551	20.53773	5.846745E-06
rs6966462	316	0.13765823	2.217322	0.652051	11.56364	0.0006725423
rs4875864	316	0.45727848	-1.602163	0.4489159	12.73747	0.0003584022
rs2720770	316	0.1028481	2.385362	0.7240537	10.85343	0.0009861304
rs3896235	316	0.23734177	-1.749202	0.5167708	11.45735	0.0007121196
rs7008755	316	0.2278481	1.895249	0.5467588	12.01547	0.0005276084
rs6468523	316	0.46677215	1.539729	0.4665549	10.89138	0.0009661271
rs10464982	316	0.31962025	1.652988	0.4917958	11.29716	0.0007762554
rs4317547	316	0.24683544	1.855989	0.558487	11.04396	0.000889767
rs4075647	316	0.21993671	2.160202	0.5593748	14.91361	0.0001125482
rs3739586	316	0.35917722	1.809453	0.4744613	14.5443	0.0001369018
rs10815610	316	0.42380952	1.728755	0.4586755	14.20547	0.0001638931
rs10758866	316	0.42246835	1.742075	0.4570163	14.53016	0.0001379334
rs4742339	316	0.37816456	1.969111	0.4715662	17.43633	2.970933E-05
rs6477206	316	0.41455696	1.592388	0.4558325	12.20358	0.0004769796
rs10746806	316	0.47310127	1.562272	0.4589396	11.58783	0.0006638482
rs7025567	316	0.07911392	2.962352	0.8521265	12.08552	0.0005081506
rs16907311	316	0.06170886	3.424125	0.9360086	13.38257	0.0002539735
rs12238387	316	0.06170886	3.424125	0.9360086	13.38257	0.0002539735
rs7868771	316	0.07436709	2.98141	0.8737019	11.64439	0.0006439649
rs11788257	316	0.06329114	-3.475038	0.9541116	13.26541	0.0002703479
rs11185726	316	0.13291139	2.229035	0.6688718	11.10575	0.0008606031
rs10997908	316	0.1693038	2.183185	0.612643	12.6989	0.0003658715
rs923799	316	0.47626582	-1.509509	0.4506519	11.2199	0.0008092465
rs7085310	316	0.32380952	1.744423	0.4787122	13.27867	0.0002684424
rs422316	316	0.47626582	-1.550569	0.4562883	11.54791	0.0006782559
rs12419084	316	0.24525316	-1.78285	0.5034151	12.5423	0.0003978417
rs11030828	316	0.18670886	1.959714	0.5668145	11.95373	0.0005453813
rs10835608	316	0.21835443	-1.840516	0.5450723	11.40174	0.0007337553
rs12788347	316	0.10759494	2.923941	0.7451781	15.39633	8.715741E-05
rs11037234	316	0.33174603	1.661543	0.479799	11.99235	0.0005341936
rs7950814	316	0.25632911	-1.714053	0.5049574	11.52229	0.0006876668
rs11235519	316	0.24208861	-2.020203	0.5156264	15.35041	8.930156E-05
rs2125362	316	0.33544304	-1.673878	0.4826739	12.02652	0.0005244904
rs2605611	316	0.17405063	-2.040316	0.6155436	10.98695	0.0009175557
rs4753472	316	0.4556962	1.592788	0.4653303	11.71638	0.0006195249
rs17102906	316	0.07436709	2.844775	0.816704	12.13295	0.0004953864
rs4937504	316	0.37816456	-1.598661	0.4610699	12.02207	0.000525742
rs11222121	316	0.08702532	3.026174	0.7897074	14.68438	0.0001270955
rs12321232	316	0.23417722	-2.081865	0.556885	13.97572	0.0001851871
rs10843050	316	0.23101266	-2.06852	0.5580638	13.7389	0.0002100587
rs4763682	316	0.32539683	1.642634	0.4619907	12.64199	0.0003771789
rs10845334	316	0.32278481	1.652339	0.4568253	13.08271	0.0002980332
rs7300030	316	0.31962025	1.710418	0.4616141	13.72923	0.0002111427
rs876373	316	0.4335443	1.504626	0.4453836	11.4127	0.0007294367
rs886131	316	0.41025641	-1.595995	0.4569148	12.20093	0.000477658
rs7961894	316	0.08544304	2.746108	0.8261708	11.0483	0.000887685
rs7961894	316	0.08544304	2.746108	0.8261708	11.0483	0.000887685
rs7990975	316	0.07436709	3.075672	0.8927775	11.86843	0.0005709337
rs11841745	316	0.05537975	3.495036	1.0117923	11.9322	0.0005517194
rs9576925	316	0.21993671	-1.901715	0.5663725	11.27422	0.0007859073
rs644061	316	0.34018987	1.822644	0.4737972	14.79854	0.0001196282
rs12867132	316	0.4952381	1.471779	0.4428	11.04765	0.0008879961

rs7338389	316	0.33860759	1.79431	0.4735841	14.35491	0.0001513843
rs2180451	316	0.19936709	2.140322	0.5749232	13.85921	0.0001970286
rs1706804	316	0.37296417	-1.72594	0.4939786	12.20774	0.000475916
rs753414	316	0.34968354	-1.900617	0.5181603	13.45429	0.0002444463
rs753414	316	0.35048232	-1.903263	0.5195334	13.42056	0.0002488818
rs3959644	316	0.38449367	-1.79173	0.5008214	12.79911	0.000346785
rs1648302	316	0.36392405	-1.623216	0.4794247	11.46336	0.0007098186
rs3743395	316	0.21202532	-1.865213	0.5608123	11.0617	0.0008812939
rs4932444	316	0.37816456	-1.528711	0.4619548	10.95095	0.000935558
rs2641805	316	0.28164557	-1.706911	0.5100376	11.19998	0.0008179841
rs2738808	316	0.28164557	-1.706911	0.5100376	11.19998	0.0008179841
rs12149458	316	0.07120253	-3.027004	0.8853977	11.68824	0.0006289626
rs9940705	316	0.06170886	3.610458	0.9073319	15.83406	6.914671E-05
rs9911465	316	0.19462025	1.872588	0.5580125	11.26151	0.0007913097
rs12944658	316	0.22151899	1.809334	0.5338671	11.48606	0.0007012
rs8080053	316	0.23101266	2.334525	0.5139842	20.62992	5.571841E-06
rs4796675	316	0.22151899	2.38109	0.5185764	21.0827	4.398809E-06
rs1840549	316	0.28639241	1.635449	0.4831002	11.46039	0.0007109528
rs11079002	316	0.3085443	1.61186	0.4741587	11.55599	0.0006753134
rs12103535	316	0.4047619	-1.711877	0.4848798	12.46456	0.0004147456
rs6501226	316	0.36234177	1.688555	0.4778236	12.48807	0.0004095583
rs2120660	316	0.41297468	-1.818764	0.4812658	14.28179	0.0001573803
rs671972	316	0.36234177	1.637314	0.4777415	11.74567	0.000609848
rs72849841	316	0.17088608	-2.18794	0.5703468	14.7161	0.0001249746
rs1153773	316	0.48259494	-1.516091	0.4403826	11.85195	0.0005760087
rs10409452	316	0.18670886	-1.92094	0.5624052	11.66619	0.0006364615
rs10416524	316	0.26424051	-1.857069	0.5027884	13.64224	0.0002211537
rs2238630	316	0.31803797	1.649445	0.4748028	12.06838	0.0005128417
rs537188	316	0.15189873	2.079094	0.6240714	11.09889	0.0008637932
rs4808209	316	0.06962025	3.067108	0.8778806	12.20639	0.0004762618
rs4808209	316	0.0686901	3.000343	0.8864614	11.45571	0.0007127467
rs12972060	316	0.24525316	1.717324	0.5187446	10.95967	0.0009311645
rs7246814	316	0.33544304	1.659825	0.4675679	12.60188	0.0003853595
rs10423969	316	0.22468354	1.760397	0.5276895	11.12921	0.0008497894
rs3761072	316	0.4031746	-1.510289	0.4463973	11.44662	0.0007162427
rs2451994	316	0.18037975	2.046453	0.5734236	12.73656	0.0003585757
rs8099939	316	0.4047619	1.701353	0.4627134	13.51962	0.0002360818
rs4803523	316	0.19303797	1.854141	0.5575812	11.05782	0.0008831405
rs321938	316	0.28322785	1.78907	0.5109829	12.25863	0.0004631107
rs459710	316	0.36234177	-1.630107	0.4918548	10.98395	0.0009190443
rs4813406	316	0.25791139	-1.797599	0.5278528	11.59738	0.0006604478
rs6132792	316	0.2056962	2.084369	0.5572853	13.98924	0.0001838599
rs4809745	316	0.07436709	-3.031202	0.8723389	12.07422	0.0005112376
rs8122282	316	0.45727848	-1.494234	0.4379063	11.64326	0.0006443549
rs6062267	316	0.45253165	-1.485704	0.442846	11.25536	0.0007939346
rs2838358	316	0.28322785	1.718725	0.4967494	11.97122	0.000540287
rs362043	316	0.29272152	1.691509	0.5066568	11.14604	0.0008421102
rs16997918	316	0.12974684	2.328503	0.6639763	12.29839	0.0004533489
rs5765085	316	0.19462025	1.847151	0.5532276	11.148	0.0008412213
