# Supplementary material for: The transcriptome-wide association search for genes and genetic variants which associate with BMI and gestational weight gain in women with type 1 diabetes
Source: Mol Med. 2021 Jan 20;27:6. doi: 10.1186/s10020-020-00266-z (PMC7818927; doi:10.1186/s10020-020-00266-z)
Supplement: Supplementary file 11 — Additional file 11: Table S7. The FUMA analysis on BMI associated variants in GWG associated genes in T1DM cohort. [file 10020_2020_266_MOESM11_ESM.pdf]

GeneSet	N	n	P-value	adjusted P	genes
Body mass index	1358	363	1,69E-138	3,07E-135	PRDX1, AKR1A1, NASP, CCDC17, GPBP1L1, RPS15AP10, TMEM69, IPP, MAST2, PIK3R3, TSPAN1, POMGNT1, LURAP1, RAD54L, LRRC41, UQCRH, NSUN4, FAAH, DMBX1, TAL1, SNORA58, INADL, ATG4C, LRRIQ3, FPGT, FPGT-TNNI3K, TNNI3K, C1orf173, CRYZ, TYW3, AK5, ZZZ3, FUBP1, DNAJB4, GIPC2, GOLPH3L, HORMAD1, CTSS, CTSK, ARNT, SETDB1, CERS2, ANXA9, FAM63A, BNIPL, GABPB2, SEMA6C, TNFAIP8L2, PIP5K1A, PSMD4, ZNF687, PI4KB, RFX5, PSMB4, POGZ, CGN, TUFT1, MIR554, SNX27, NAV1, SHISA4, LMOD1, EIF3F, TUB, RIC3, LMO1, STK33, TRIM66, RPL27A, SNORA3, SNORA45, ST5, AKIP1, C11orf16, ASCL3, TMEM9B, TMEM9B-AS1, ARAP1, CNTN1, PDZRN4, SNORA3, TCTN1, MYL2, CUX2, FAM109A, SH2B3, ATXN2, BRAP, ACAD10, ALDH2, MAPKAPK5-AS1, MAPKAPK5, ADAM1A, TMEM116, ERP29, NAA25, TRAFD1, HECTD4, RPL6, PTPN11, RPH3A, OAS1, OAS3, OAS2, DTX1, RASAL1, CCDC42B, DDX54, RITA1, IQCD, SLC8B1, PLBD2, SDS, SDSL, LHX5, SFSWAP, MMP17, ULK1, PUS1, EP400, SNORA49, EP400NL, DDX51, NOC4L, GALNT9, FBRSL1, LRCOL1, P2RX2, POLE, GOLGA3, DNAJC3, UGGT2, HS6ST3, ZNF839, CINP, TECPR2, ANKRD9, MIR4309, RCOR1, TRAF3, AMN, CDC42BPB, EXOC3L4, TNFAIP2, LINC00605, EIF5, SNORA28, MARK3, CKB, TRMT61A, BAG5, KLC1, APOPT1, XRCC3, ZFYVE21, PPP1R13B, SNORD51, DDX11L10, POLR3K, SNRNP25, RHBDF1, HBZ, HBM, HBA2, HBA1, ITFG3, ITFG3, RGS11, ARHGDIG, PDIA2, AXIN1, MRPL28, TMEM8A, NME4, DECR2, CAPN15, MIR5587, MIR3176, FAM195A, WDR90, RHOT2, RHBDL1, MSLN, MIR662, RPUSD1, CHTF18, PDXDC1, NTAN1, INO80E, ZNF646, KAT8, SLC7A6, PRMT7, TANGO6, HAS3, CHTF8, CHTF8, CIRH1A, SNTB2, VPS4A, COG8, PDF, RP11-343C2.12, NIP7, TMED6, TERF2, CYB5B, MIR1538, NQO1, NOB1, WWP2, MIR140, CLEC18A, PDXDC2P, SMG6, CDC27, MYL4, ITGB3, ITGB3, EFCAB13, MRPL45P2, NPEPPS, KPNB1, TBKBP1, TBX21, OSBPL7, MRPL10, LRRC46, SP6, SP2, PNPO, PRR15L, CDK5RAP3, COPZ2, MIR152, CBX1, SNX11, SKAP1, MIR1203, HOXB1, HOXB2, HOXB3, HOXB4, DDX42, RBFOX3, RPTOR, RIT2, SYT4, REXO1, CSNK1G2, LSM4, PGPEP1, GDF15, CRTC1, ZNF101, CHST8, KCTD15, LSM14A, TMEM160, ZC3H4, SNORD51, USP37, PLK1S1, SNORA3, VGLL4, PPARG, IQSEC1, DAG1, BSN-AS2, BSN, APEH, MST1, RNF123, IP6K1, CDHR4, FAM212A, UBA7, MST1R, MON1A, RBM6, RBM5, SEMA3F, GNAT1, SLC38A3, GNAI2, LSMEM2, IFRD2, HYAL3, HYAL2, NPRL2, LINC00882, CD47, ADCY5, MRPL3, SNORA58, CPNE4, MIR5704, ACPP, DNAJC13, NPHP3-AS1, RASA2, GK5, HTR3D, ECE2, PSMD2, EIF4G1, IGF2BP2, TRA2B, ETV5, DGKG, SNORA3, ELL2, MIR583, PCSK1, CAST, ERAP1, ERAP2, VPS52, RPS18, B3GALT4, WDR46, KIFC1, PHF1, CUTA, SYNGAP1, BAK1, GGNBP1, LINC00336, ITPR3, UQCC2, IP6K3, LEMD2, MLN, MIR1275, GRM4, HMGA1, C6orf1, NUDT3, PACSIN1, C6orf106, UHRF1BP1, MAS1, IGF2R, AIRN, SLC22A1, SLC22A2, SLC22A3, LPAL2, LPA, PLG, PARK2, VWC2, ZPBP, C7orf72, IKZF1, FIGNL1, DDC, GRB10, COBL, HIP1, UPK3B, DTX2P1, DTX2P1-UPK3BP1-PMS2P11, PMS2P11, PMS2P9, CCDC146, MAGI2, ASB4, SNORA3, MIR5708, ZBTB10, FRRS1L, EPB41L4B
Regular attendance at a gym or sports club	42	33	1,27E-32	1,15E-29	INADL, L1TD1, KANK4, USP1, DOCK7, ANGPTL3, BSN, APEH, MST1, RNF123, AMIGO3, GMPPB, IP6K1, CDHR4, FAM212A, UBA7, MIR5193, TRAIP, CAMKV, MST1R, MON1A, RBM6, RBM5, SEMA3F, ITPR3, UQCC2, IP6K3, LEMD2, MLN, MIR1275, GRM4, HMGA1, C6orf1
Sleep duration (short sleep)	99	45	1,27E-28	7,70E-26	NAV1, IPO9, SHISA4, LMOD1, USP4, GPX1, RHOA, TCTA, AMT, NICN1, DAG1, BSN, APEH, MST1, RNF123, AMIGO3, GMPPB, IP6K1, FAM212A, UBA7, TRAIP, CAMKV, ACTBP13, MST1R, MON1A, RBM6, RBM5, SEMA3F, GNAT1, SLC38A3, GNAI2, SEMA3B, LSMEM2, IFRD2, HYAL3, NAT6, HYAL1, HYAL2, TUSC2, RASSF1, ZMYND10, NPRL2, CYB561D2, TMEM115, CACNA2D2, XXcos-LUCA11.4
Bipolar II disorder	18	18	1,09E-22	4,95E-20	MEF2BNB-MEF2B, MEF2BNB, RFXANK, NR2C2AP, NCAN, HAPLN4, TM6SF2, SUGP1, MAU2, GATAD2A, TSSK6, NDUFA13, YJEFN3, CILP2, PBX4, LPAR2, GMIP, ATP13A1
Body mass index (age <50)	52	29	3,47E-22	1,26E-19	LRRIQ3, FPGT, FPGT-TNNI3K, TNNI3K, C1orf173, CRYZ, TYW3, EIF3F, TUB, RIC3, LMO1, STK33, TRIM66, RPL27A, SNORA3, SNORA45, ST5, AKIP1, C11orf16, ASCL3, TMEM9B, TMEM9B-AS1, SNORA3, SNORA3, SNORA3, ZPBP, C7orf72, IKZF1, FIGNL1, DDC, GRB10, COBL, SNORA3
Body mass index x sex x age interaction (4df test)	106	38	5,58E-20	1,69E-17	LRRIQ3, FPGT, FPGT-TNNI3K, TNNI3K, C1orf173, CRYZ, TYW3, EIF3F, TUB, RIC3, LMO1, STK33, TRIM66, RPL27A, SNORA3, SNORA45, ST5, AKIP1, C11orf16, ASCL3, TMEM9B, TMEM9B-AS1, SNORA3, SNORA3, SNORA3, MAS1, IGF2R, AIRN, SLC22A1, SLC22A2, SLC22A3, LPAL2, LPA, PLG, ZPBP, C7orf72, IKZF1, FIGNL1, DDC, GRB10, COBL, SNORA3

Regular attendance at a religious group	78	27	3,53E-14	9,14E-12	NFIA, RHOA, TCTA, AMT, NICN1, DAG1, BSN-AS2, BSN, APEH, MST1, RNF123, AMIGO3, GMPPB, IP6K1, CDHR4, FAM212A, UBA7, MIR5193, TRAIP, CAMKV, MST1R, MON1A, RBM6, CD47, IFT57, HHLA2, MYH15
Extremely high intelligence	81	26	7,89E-13	1,79E-10	CKB, TRMT61A, GPX1, RHOA, TCTA, AMT, NICN1, DAG1, BSN, APEH, MST1, RNF123, AMIGO3, GMPPB, IP6K1, CDHR4, FAM212A, UBA7, TRAIP, CAMKV, MST1R, MON1A, RBM6, RBM5, SEMA3F, GNAT1
Schizophrenia	801	100	5,12E-12	1,03E-09	MKNK1, MOB3C, LRRIQ3, VPS45, PLEKHO1, ANP32E, CA14, APH1A, C1orf54, C1orf51, MRPS21, RPRD2, ECM1, FAM86C1, PITPNM2, MPHOSPH9, C12orf65, CDK2AP1, SBNO1, hsa-mir-8072, SETD8, RILPL2, CKB, TRMT61A, BAG5, KLC1, APOPT1, XRCC3, ZFYVE21, PPP1R13B, INO80E, DOC2A, C16orf92, FAM57B, ALDOA, PPP4C, TBX6, YPEL3, GDPD3, MAPK3, RLTPR, ACD, PARD6A, ENKD1, C16orf86, GFOD2, RANBP10, TSNAXIP1, CENPT, THAP11, NUTF2, EDC4, NRN1L, PSKH1, CTRL, PSMB10, LCAT, SLC12A4, CTC-479C5.17, DPEP3, DPEP2, DUS2, DDX28, NFATC3, ESRP2, PLA2G15, SLC7A6, SLC7A6OS, YWHAE, SMG6, SRR, TSR1, SNORD91B, SNORD91A, SGSM2, NCAN, HAPLN4, TM6SF2, SUGP1, MAU2, GATAD2A, MIR640, TSSK6, NDUFA13, YJEFN3, CILP2, PBX4, LPAR2, GMIP, CEBPG, PEPD, GNAT1, HYAL3, RASSF1, KALRN, SYNGAP1, BAK1, LEMD2, HSPB1, YWHAG, MAGI2
Crohn's disease	600	80	2,84E-11	5,02E-09	USP1, DOCK7, ANGPTL3, RORC, C1orf106, KIF21B, CACNA1S, STARD10, ATG16L2, FCHSD2, LRRK2, MUC19, SH2B3, ATXN2, BRAP, ACAD10, ALDH2, MAPKAPK5, TMEM116, ERP29, NAA25, PSMB10, GNGT2, KCNH6, HMHA1, GPX4, SBNO2, STK11, LMNB2, PPP5C, CCDC8, PNMAL1, PPP5D1, PNMAL2, CALM3, PTGIR, GNG8, DNPEP, SLC25A20, ARIH2OS, ARIH2, P4HTM, WDR6, DALRD3, NDUFAF3, IMPDH2, QRICH1, QARS, USP19, LAMB2, CCDC71, KLHDC8B, C3orf84, CCDC36, C3orf62, USP4, GPX1, RHOA, TCTA, AMT, NICN1, DAG1, BSN, APEH, MST1, RNF123, ZBTB38, RNF7, ERAP2, LNPEP, ITPR3, IP6K3, LEMD2, MLN, ZPBP, C7orf72, IKZF1, FIGNL1, FGL2, GSAP
Blood protein levels	1776	177	3,04E-11	5,02E-09	TESK2, CCDC163P, MMACHC, PRDX1, AKR1A1, NASP, CCDC17, GPBP1L1, RPS15AP10, TMEM69, IPP, MAST2, ANGPTL3, CRYZ, ST6GALNAC5, MRPS21, PRPF3, RPRD2, TARS2, ECM1, LINC00568, MCL1, GOLPH3L, HORMAD1, RNU6-1042P, CTSS, CTSK, ARNT, C1orf56, CSRP1, RNPEP, RIC3, SWAP70, FOLR3, PHOX2A, RELT, FAM168A, HMGN2P38, CNTN1, GXYLT1, SNORA48, SH2B3, BRAP, OAS1, CDK2AP1, ZNF839, CINP, TECPR2, RNU6-244P, ANKRD9, SNORA48, SNORA48, MPG, HBZ, TMEM8A, WFIKKN1, CACNA1H, TPSG1, TPSB2, TPSAB1, TPSD1, PRSS29P, SNORA48, MAPK3, LRRC36, TPPP3, RNU1-123P, ZDHHC1, HSD11B2, ATP6V0D1, RP11-297D21.4, AGRP, FAM65A, SNORA48, SMPD3, NQO1, MYO1C, SCARF1, PRPF8, SERPINF2, SERPINF1, SNORA48, CALCOCO2, MRC2, ACE, ACE, ENPP7, GAA, NPTX1, ENTHD2, SNORA48, HMHA1, AMH, LMNB2, IL12RB1, GDF15, CRLF1, HAPLN4, NAPA, SULT2A1, SNORA48, SNORA48, TIMP4, SLC25A20, ARIH2OS, ARIH2, P4HTM, WDR6, DALRD3, NDUFAF3, IMPDH2, QRICH1, RN7SL182P, QARS, USP19, LAMB2, CCDC71, KLHDC8B, C3orf84, CCDC36, USP4, GPX1, RHOA, RHOA-IT1, TCTA, AMT, NICN1, RNA5SP130, DAG1, BSN-AS2, BSN, BSN-AS1, APEH, MST1, RNF123, AMIGO3, GMPPB, IP6K1, TRAIP, CAMKV, RN7SL217P, ACTBP13, RBM6, RBM5, NPRL2, C3orf18, MAPKAPK3, DOCK3, ST13P14, PDIA5, DNAJC13, ACAD11, NPHP3, ACKR4, HSPA8P19, UBA5, PSMD2, EHHADH, DNAJB11, AHSG, FETUB, HRG, KNG1, PSMD10P2, ADIPOQ, ST6GAL1, SNORA48, RFESD, PCSK1, CAST, ERAP1, ERAP2, LNPEP, TAPBP, MLN, CLPS, MAS1, IGF2R, RP11-288H12.3, SLC22A1, LPA, PLG, MAP3K4, VWC2, HSPB1, PON1, SNORA48, FABP4
Body mass index (joint analysis main effects and physical activity interaction)	87	25	3,35E-11	5,06E-09	PIK3R3, TNNI3K, ZZZ3, DNAJB4, NAV1, LMOD1, STK33, TRIM66, RPL27A, TBKBP1, TBX21, CRTC1, KCTD15, ZC3H4, RASA2, ETV5, DGKG, LEMD2, PACSIN1, UHRF1BP1, PARK2, HIP1, UPK3B, DTX2P1-UPK3BP1-PMS2P11, PMS2P11
Rhegmatogenous retinal detachment	20	12	1,85E-10	2,59E-08	ADAMTSL4, MCL1, ENSA, GOLPH3L, HORMAD1, CTSS, CTSK, ARNT, CERS2, ANXA9, FAM63A, PRUNE

Bipolar I disorder	90	24	4,53E-10	5,87E-08	VKORC1, BCKDK, KAT8, PRSS8, PRSS36, FUS, MEF2BNB-MEF2B, MEF2BNB, RFXANK, NR2C2AP, NCAN, HAPLN4, TM6SF2, SUGP1, MAU2, GATAD2A, TSSK6, NDUFA13, YJEFN3, CILP2, PBX4, LPAR2, GMIP, ATP13A1
Body mass index in physically active individuals	76	21	2,71E-09	3,28E-07	PIK3R3, TNNI3K, ZZZ3, DNAJB4, NAV1, LMOD1, STK33, TRIM66, RPL27A, CRTC1, KCTD15, ZC3H4, RASA2, ETV5, PACSIN1, UHRF1BP1, PARK2, HIP1, UPK3B, DTX2P1-UPK3BP1-PMS2P11, PMS2P11
Alcohol consumption (max-drinks)	17	10	8,41E-09	9,54E-07	MYL2, CUX2, BRAP, ACAD10, ALDH2, NAA25, TRAFD1, HECTD4, PTPN11, RPH3A
Chronic obstructive pulmonary disease or resting heart rate (pleiotropy)	74	20	9,78E-09	1,04E-06	DYNC1H1, HSP90AA1, WDR20, MOK, ZNF839, TNS1, KIFC1, PHF1, CUTA, SYNGAP1, MIR5004, ZBTB9, BAK1, GGNBP1, LINC00336, ITPR3, UQCC2, IP6K3, LEMD2, MLN
Ulcerative colitis	366	49	1,65E-07	1,65E-05	RORC, C1orf106, MROH3P, KIF21B, CACNA1S, ITGAL, ZFP90, CDH3, CDH1, LMNB2, CALM3, CXCR2, CXCR1, AAMP, SLC11A1, DNPEP, SLC25A20, ARIH2OS, ARIH2, P4HTM, WDR6, DALRD3, NDUFAF3, IMPDH2, QRICH1, QARS, USP19, LAMB2, CCDC71, KLHDC8B, C3orf84, CCDC36, C3orf62, USP4, GPX1, RHOA, TCTA, AMT, NICN1, DAG1, BSN, APEH, MST1, RNF123, AMIGO3, GMPPB, IP6K1, UBA7, MST1R
Response to alcohol consumption (flushing response)	13	8	1,73E-07	1,65E-05	CUX2, BRAP, ACAD10, ALDH2, NAA25, TRAFD1, HECTD4, PTPN11
Body fat distribution (arm fat ratio)	129	25	2,04E-07	1,85E-05	INO80E, DOC2A, FAM57B, ALDOA, PPP4C, TBX6, YPEL3, GDPD3, ZNF668, ZNF668, ZNF646, PRSS53, VKORC1, NFAT5, NQO1, NOB1, WWP2, ZNF652, ZC3H4, RBM6, DOCK3, ZBTB38, RASA2, C6orf106, SNRPC, UHRF1BP1
Coronary heart disease	73	18	2,44E-07	2,11E-05	PPP1R12B, MYL2, CUX2, SH2B3, ACAD10, ALDH2, HECTD4, SMG6, SRR, ATP5G1, UBE2Z, SNF8, GIP, GNAI2, ANKS1A, SLC22A3, LPAL2, LPA
Height	869	90	4,82E-07	3,91E-05	GIPC2, IGFN1, TNNT2, LAD1, SBNO1, RILPL2, FAM101A, DDX51, RP13-977J11.2, CKB, NPRL3, RAB40C, WFIKKN1, HAGHL, NARFL, MKL2, PARN, PDXDC1, NTAN1, DOC2A, WWP2, ABR, NPEPPS, OSBPL7, AC003665.1, ATP5G1, ZNF652, DCAF7, MAP3K3, STRADA, CSH2, CSH1, CD79B, SCN4A, AP3D1, DOT1L, C19orf35, MAP1S, PGPEP1, MEF2BNB-MEF2B, MEF2BNB, GATAD2A, TNS1, RUFY4, CXCR2P1, PNKD, USP37, PLCD4, TTLL4, FEV, CRYBA2, IHH, NHEJ1, SLC23A3, TUBA4A, DNPEP, C20orf26, RALGAPA2, PLK1S1, VGLL4, SYN2, CCDC36, RBM6, HEMK1, DOCK3, ACPL2, ZBTB38, KRT18P35, RASA2, SENP2, IGF2BP2, C3orf65, IP6K3, KRT18P9, HMGA1, C6orf1, NUDT3, SPDEF, C6orf106, ANKS1A, TCP11, SCUBE3, ZNF76, DEF6, PPARD, FANCE, IKZF1, RSBN1L, SLC25A13, PALM2-AKAP2
Lipoprotein (a) levels	41	13	4,96E-07	3,91E-05	SOD2, ACAT2, PNLDC1, MAS1, IGF2R, SLC22A1, SLC22A2, SLC22A3, LPA, PLG, MAP3K4, AGPAT4, PARK2
Low HDL-cholesterol levels	5	5	8,05E-07	6,09E-05	PTPN11, RPH3A, OAS1, OAS3, OAS2
Coffee consumption	104	21	9,29E-07	6,74E-05	SETDB1, MYL2, CUX2, BRAP, ACAD10, ALDH2, MAPKAPK5, NAA25, TRAFD1, HECTD4, RPL6, OAS2, DTX1, SYT4, GDF15, ZC3H4, CCDC36, CACNA2D2, POR, SNORA14A, TMEM120A
Serum uric acid levels in response to allopurinol in gout	31	11	1,05E-06	7,36E-05	RP5-1109J22.2, LINC00505, DMBX1, THEM5, THEM4, NDE1, MYH11, BAIAP2-AS1, BAIAP2, AATK, PRKD2
Body fat distribution (leg fat ratio)	225	33	2,25E-06	1,51E-04	PITPNM2, NFAT5, NQO1, NOB1, WWP2, OSBPL7, ZNF652, TM6SF2, SUGP1, MAU2, GATAD2A, TSSK6, NDUFA13, CILP2, ARIH2OS, ARIH2, P4HTM, WDR6, DALRD3, NDUFAF3, IMPDH2, QRICH1, QARS, RBM6, ZBTB38, RASA2, NUDT3, RPS10, PACSIN1, SPDEF, C6orf106, SNRPC, UHRF1BP1

Inflammatory bowel disease	640	69	2,58E-06	1,67E-04	RORC, C1orf106, KIF21B, CACNA1S, LRRK2, MUC19, SH2B3, ATXN2, BRAP, ACAD10, ALDH2, MAPKAPK5, TMEM116, ERP29, NAA25, GPX4, SBNO2, STK11, LMNB2, CEBPG, RUFY4, CXCR2, CXCR1, ARPC2, GPBAR1, AAMP, PNKD, TMBIM1, SLC11A1, CTDSP1, DNPEP, SLC25A20, ARIH2OS, ARIH2, P4HTM, WDR6, DALRD3, NDUFAF3, IMPDH2, QRICH1, QARS, USP19, LAMB2, CCDC71, KLHDC8B, C3orf84, CCDC36, C3orf62, USP4, GPX1, RHOA, TCTA, AMT, NICN1, DAG1, BSN, APEH, MST1, RNF123, IP6K1, MST1R, ERAP1, ERAP2, LNPEP, PACSIN1, SPDEF, ZPBP, C7orf72, IKZF1
Alcohol consumption (drinkers vs non-drinkers)	6	5	4,59E-06	2,87E-04	CCDC63, MYL2, ALDH2, HECTD4, OAS3
Body mass index (joint analysis main effects and smoking interaction)	81	17	5,71E-06	3,34E-04	INADL, TNNI3K, FUBP1, NAV1, TRIM66, INO80E, KAT8, LSM4, PGPEP1, KCTD15, ZC3H4, RASA2, ETV5, C6orf106, PARK2, HIP1, ASB4
BMI (adjusted for smoking behaviour)	81	17	5,71E-06	3,34E-04	INADL, TNNI3K, FUBP1, NAV1, TRIM66, INO80E, KAT8, LSM4, PGPEP1, KCTD15, ZC3H4, RASA2, ETV5, C6orf106, PARK2, HIP1, ASB4
Alcohol dependence symptom count	20	8	1,15E-05	6,53E-04	ARAP1, BRAP, ACAD10, ALDH2, NAA25, TRAFD1, HECTD4, PTPN11
Cholangiocarcinoma in primary sclerosing cholangitis (time to event)	16	7	2,06E-05	1,13E-03	CACNA2D2, C3orf18, HEMK1, CISH, MAPKAPK3, MIR4787, DOCK3
Platelet count	339	40	4,39E-05	2,35E-03	TESK2, IPP, PRPF3, ST5, FAM109A, SH2B3, ATXN2, BRAP, ACAD10, TMEM116, NAA25, PTPN11, RPH3A, PITPNM2, MPHOSPH9, ABCC4, TECPR2, RCOR1, EXOC3L4, RAB11FIP3, HIC1, SMG6, NFE2L1, BTBD2, NDUFA13, YJEFN3, ATP13A1, ARPC2, SYN2, PPARG, PDIA5, KALRN, ABCC5, EIF2B5, THPO, CHRD, IGF2BP2, BAK1, GGNBP1, UHRF1BP1
Empathy quotient	18	7	5,15E-05	2,67E-03	ZDHHC1, HSD11B2, ATP6V0D1, ENKD1, GFOD2, RANBP10, TSNAXIP1
HDL cholesterol	241	31	6,20E-05	3,13E-03	DOCK7, CERS2, MYL2, ATXN2, HECTD4, OAS3, SBNO1, CCDC92, ZNF664, SCARB1, CTCF, GFOD2, RANBP10, EDC4, PSKH1, LCAT, VPS4A, KPNB1, SP6, PEPD, PRKAG3, ATG7, PPARG, RBM6, RBM5, ACAD11, DGKG, C6orf106, SNRPC, LPA, IKZF1
Coronary artery disease	454	49	6,71E-05	3,27E-03	ADAMTSL4, ADAMTSL4-AS1, MCL1, TDRKH, LINGO4, LMOD1, DENND5A, SWAP70, PPHLN1, SH2B3, ATXN2, NAA25, DNAH10, CCDC92, SCARB1, MARK3, CKB, MYH11, SMG6, EFCAB13, GIP, B4GALNT2, ZNF652, CSNK1G2, CSNK1G2-AS1, MAP1S, FCHO1, PGPEP1, ELL, PRKD2, MIR320E, TNS1, QRICH1, QARS, RHOA, TCTA, AMT, GNAI2, DNAJC13, IGF2BP2, C6orf106, UHRF1BP1, ANKS1A, FKBP5, IGF2R, SLC22A3, LPAL2, LPA, PLG
High density lipoprotein cholesterol levels	79	15	6,86E-05	3,27E-03	MYL2, CUX2, HECTD4, OAS3, SETD8, RILPL2, SCARB1, UBC, LCAT, DPEP2, INSL3, PPARG, PACSIN1, C7orf72, IKZF1
BMI in non-smokers	62	13	7,10E-05	3,30E-03	INADL, FUBP1, NAV1, INO80E, KAT8, LSM4, KCTD15, ZC3H4, RASA2, ETV5, C6orf106, PARK2, HIP1
Generalized epilepsy	25	8	7,53E-05	3,42E-03	PNPO, PRR15L, CDK5RAP3, COPZ2, NFE2L1, CBX1, SNX11, SKAP1
Hemoglobin levels	39	10	8,06E-05	3,57E-03	SH2B3, MPG, NPRL3, HBZ, HBM, HBA2, HBA1, HBQ1, ITFG3, ITFG3, IKZF1
Plateletcrit	213	28	9,41E-05	4,07E-03	TESK2, PRPF3, ST5, SH2B3, ATXN2, BRAP, NAA25, MPHOSPH9, ABCC4, RCOR1, EXOC3L4, HIC1, SMG6, NFE2L1, MAU2, ATP13A1, PNKD, PDIA5, KALRN, EIF2B5, POLR2H, THPO, CHRD, IGF2BP2, BAK1, GGNBP1, IP6K3, UHRF1BP1

Response to cognitive-behavioural therapy in major depressive disorder	40	10	1,02E-04	4,25E-03	POM121C, AC018720.10, PMS2P3, HIP1, CCL26, CCL24, RHBDD2, POR, MIR4651, SNORA14A
Hodgkin's lymphoma	26	8	1,03E-04	4,25E-03	MAPK3, CORO1A, TCF3, CLSTN2, ERAP1, ITPR3, UQCC2, IP6K3
Response to fenofibrate (adiponectin levels)	20	7	1,13E-04	4,54E-03	OAS1, OAS3, OAS2, DDX54, TPCN1, SLC8B1, IGF2BP1
Body fat distribution (trunk fat ratio)	238	30	1,17E-04	4,61E-03	PITPNM2, OSBPL7, ZNF652, TM6SF2, SUGP1, MAU2, GATAD2A, TSSK6, NDUFA13, CILP2, ARIH2OS, ARIH2, P4HTM, WDR6, DALRD3, NDUFAF3, IMPDH2, QRICH1, QARS, RBM6, DOCK3, ZBTB38, RASA2, NUDT3, RPS10, PACSIN1, SPDEF, C6orf106, SNRPC, UHRF1BP1
Thyroid peroxidase antibody levels	6	4	1,81E-04	6,95E-03	SH2B3, ATXN2, KALRN, THPO
Very long-chain saturated fatty acid levels (fatty acid 20:0)	28	8	1,84E-04	6,95E-03	USP1, DOCK7, ATG4C, ITGB3, ITGB3, EFCAB13, NPEPPS, KPNB1, TBKBP1
Morning person	201	26	2,13E-04	7,89E-03	INADL, PIGK, AK5, ANP32E, CA14, PRPF3, TARS2, ENSA, GOLPH3L, ANXA9, ARIH2, QARS, RHOA, BSN, RNF123, CAMKV, RBM6, RBM5, SEMA3F, GNAI2, CACNA2D2, DOCK3, DGKG, DDC, GRB10, SHFM1, C7orf76
Plasma omega-6 polyunsaturated fatty acid levels (dihomo-gamma-linolenic acid)	22	7	2,23E-04	7,93E-03	PARN, PDXDC1, NTAN1, RRN3, C16orf45, KIAA0430, MYH11
Blood osmolality (transformed sodium)	22	7	2,23E-04	7,93E-03	CCDC63, CUX2, ATXN2, CYB5B, NFAT5, NQO1, NOB1
Esophageal cancer	16	6	2,29E-04	8,01E-03	BRAP, ACAD10, ALDH2, HECTD4, RPL6, SEMA5B
Age at first birth	29	8	2,41E-04	8,24E-03	RNF123, CAMKV, MST1R, MON1A, RBM6, RBM5, SEMA3F, HYAL3
Calcium levels	61	12	2,52E-04	8,48E-03	ATG4C, PRPF8, WDR81, SERPINF2, SERPINF1, SMYD4, CCDC58, FAM162A, WDR5B, KPNA1, RNF7, PHTF2
Parental longevity (combined parental age at death)	11	5	2,73E-04	9,02E-03	FPGT, TNNI3K, SH2B3, ATXN2, LPA
Mean corpuscular hemoglobin concentration	120	18	3,21E-04	1,04E-02	SWAP70, CUX2, SH2B3, ALDH2, NPRL3, HBZ, HBA2, HBA1, HBQ1, LUC7L, ITFG3, ITFG3, AXIN1, PIGQ, NHLRC4, ABCA7, ELL, CTDSP1, MIR26B
Estimated glomerular filtration rate	527	52	3,83E-04	1,22E-02	AKR1A1, PIK3R3, FOXD2, AK5, MRPS21, CERS2, ANXA9, CACNA1S, CUX2, ATXN2, ACAD10, ALDH2, TMEM116, NAA25, CLDN10, ABCC1, NFATC3, SLC7A6, NFAT5, NQO1, WWP2, SMG6, KIAA1683, JUND, LSM4, CRTC1, SLC7A9, CEP89, VIL1, SPEG, SYN2, DAG1, CACNA2D2, DOCK3, ZBTB38, RNF7, TFDP2, SENP2, ETV5, KNG1, HMGA1, C6orf1, NUDT3, RPS10, SLC22A2, SLC22A3, GRB10, POR, PTPN12, TMEM60, PHTF2, PTPN3
Acute anterior uveitis (with or without ankylosing spondylitis)	12	5	4,45E-04	1,39E-02	C1orf106, KIF21B, ERAP1, ERAP2, LNPEP
Autism spectrum disorder	18	6	4,79E-04	1,47E-02	MNT, RIT2, PLK1S1, XRN2, NKX2-4, NKX2-2

Plasma omega-6 polyunsaturated fatty acid levels (gamma-linolenic acid)	25	7	5,35E-04	1,62E-02	PARN, PDXDC1, NTAN1, RRN3, C16orf45, KIAA0430, MYH11
Autism spectrum disorder or schizophrenia	563	54	5,89E-04	1,75E-02	PITPNM2, MPHOSPH9, C12orf65, CDK2AP1, SBNO1, hsa-mir-8072, SETD8, RILPL2, EIF5, SNORA28, MARK3, CKB, TRMT61A, BAG5, KLC1, APOPT1, XRCC3, ZFYVE21, PPP1R13B, INO80E, DOC2A, C16orf92, FAM57B, ALDOA, PPP4C, TBX6, YPEL3, GDPD3, MAPK3, SMG6, SRR, TSR1, SNORD91B, SNORD91A, SGSM2, HCG25, VPS52, RPS18, B3GALT4, WDR46, PFDN6, RGL2, TAPBP, ZBTB22, DAXX, KIFC1, PHF1, CUTA, SYNGAP1, MIR5004, ZBTB9, BAK1, GGNBP1, LINC00336
Immunoglobulin light chain (AL) amyloidosis	13	5	6,87E-04	1,97E-02	PRR14, FBRS, SRCAP, RNF40, BCL7C
Body mass index in physically inactive individuals	26	7	6,94E-04	1,97E-02	TBKBP1, TBX21, ETV5, DGKG, UPK3B, DTX2P1-UPK3BP1-PMS2P11, PMS2P11
Plasma omega-6 polyunsaturated fatty acid levels (linoleic acid)	26	7	6,94E-04	1,97E-02	PARN, PDXDC1, NTAN1, RRN3, C16orf45, KIAA0430, MYH11
Facial attractiveness (female raters)	8	4	7,66E-04	2,14E-02	CERS2, ANXA9, PDZRN4, GXYLT1
Waist-to-hip ratio adjusted for BMI (age >50)	232	27	8,76E-04	2,41E-02	RILPL2, SNRNP35, RILPL1, MIR3908, TMED2, DDX55, EIF2B1, GTF2H3, TCTN2, ATP6V0A2, DNAH10, CCDC92, ZNF664, FAM101A, NCOR2, SP6, SYN2, TIMP4, PPARG, TSEN2, MKRN2, RAF1, TMEM40, CAND2, RPL32, SNORA7A, IQSEC1
Mood instability	61	11	9,75E-04	2,64E-02	LMNB2, NDUFAF3, QARS, LAMB2, CCDC36, GPX1, RHOA, NICN1, DAG1, BSN, AMIGO3
Waist circumference	165	21	1,02E-03	2,72E-02	DNAJB4, NAV1, OVCH2, TRIM66, ARAP1, STARD10, PDXDC1, MAPK3, ZNF771, RN7SL33P, HOXB1, RIT2, ZC3H4, GSTO3P, ATP2C1, ZBTB38, ETV5, PCSK1, UHRF1BP1, SLC22A2, PPP1R9A
Chronic kidney disease	133	18	1,12E-03	2,93E-02	SETDB1, CERS2, ANXA9, FAM63A, PRUNE, BNIPL, ATXN2, NFAT5, TDRD12, SLC7A9, CEP89, ADCY5, PTPLB, TFDP2, SLC22A2, RSBN1L, TMEM60, PHTF2
Multisite chronic pain	36	8	1,16E-03	3,01E-02	LINC00568, RNF123, AMIGO3, GMPPB, BBX, MLN, C6orf106, DYNC1I1
Wegener's granulomatosis	9	4	1,31E-03	3,36E-02	RXRB, HSD17B8, RING1, COBL
Menarche (age at onset)	216	25	1,44E-03	3,58E-02	FPGT, TNNI3K, TYW3, TRIM66, MKL2, PARN, TBX6, NFAT5, WWP2, COG4, CRTC1, WDR6, KLHDC8B, UBA7, RBM6, ACAD11, NPHP3, ECE2, EIF4G1, IGF2BP2, TRA2B, ETV5, PCSK1, GTF2I, TMEM245
Weight	181	22	1,44E-03	3,58E-02	ATG4C, PDXDC1, NTAN1, MAPK3, PRMT7, COG4, RIT2, PGPEP1, CHST8, KCTD15, HIF3A, NEK11, ACPL2, ZBTB38, RASA2, TRA2B, ETV5, DGKG, RPL39L, SPDEF, C6orf106, PALM2
Serum total protein level	55	10	1,53E-03	3,76E-02	ADAMTSL4-AS1, FCHSD2, ATXN2, TRAF3, TBC1D16, ZBTB38, ELL2, C7orf72, IKZF1, PAG1
Red blood cell traits	84	13	1,55E-03	3,76E-02	SH2B3, ATXN2, EIF5, POLR3K, MPG, NPRL3, ITFG3, ITFG3, NME4, WDR90, RHOT2, LMF1, RP11-161M6.2, RASA2, IKZF1
Male-pattern baldness	254	28	1,63E-03	3,89E-02	FOXD2, S100A11, TCHHL1, TCHH, MKL2, PARN, DHX34, VIL1, USP37, RQCD1, PLCD4, ZNF142, BCS1L, RNF25, STK36, TTLL4, CYP27A1, PRKAG3, WNT6, WNT10A, CDK5R2, FEV, PAX1, ATG7, VGLL4, BBX, ZBTB38, RASA2
Alcohol consumption	23	6	2,01E-03	4,66E-02	LMO1, CCDC63, MYL2, ALDH2, HECTD4, OAS3

Thyroid peroxidase antibody positivity	5	3	2,01E-03	4,66E-02	SH2B3, ATXN2, THPO
Myocardial infarction	57	10	2,03E-03	4,66E-02	SH2B3, ALDH2, SMG6, UBE2Z, EIF4A3, AP3D1, DOT1L, SF3A2, LPA, PLG
Medication use (HMG CoA reductase inhibitors)	77	12	2,18E-03	4,95E-02	SNORD112, SNORD112, DOCK7, SNORD112, SNORD112, SNORD112, SNORD112, SNORD112, SNORD112, SNORD112, SNORD112, SNORD112, SNORD112, SNORD112, SNORD112, SCARB1, SNORD112, SNORD112, SNORD112, SNORD112, SNORD112, SNORD112, SNORD112, SNORD112, SNORD112, SNORD112, SNORD112, BCL7C, SNORD112, NQO1, SNORD112, KPNB1, SP6, SNORD112, SNORD112, TM6SF2, SNORD112, SNORD112, SNORD112, SNORD112, SNORD112, PPARG, SNORD112, SNORD112, IGF2BP2, SNORD112, SNORD112, SNORD112, SNORD112, SNORD112, SLC22A1, LPA, SNORD112, SNORD112, SNORD112, SNORD112, SNORD112, SNORD112, SNORD112
