# Supplementary material for: The transcriptome-wide association search for genes and genetic variants which associate with BMI and gestational weight gain in women with type 1 diabetes
Source: Mol Med. 2021 Jan 20;27:6. doi: 10.1186/s10020-020-00266-z (PMC7818927; doi:10.1186/s10020-020-00266-z)
Supplement: Supplementary file 12 — Additional file 12:Table S8a. Go Enrichment analysis on the overlap between BMI and GWG genes in the Giant cohort. b. Go Enrichment analysis on BMI only associated genes in the Giant cohort. c. Go Enrichment analysis on GWG only associated genes in the Giant cohort. [file 10020_2020_266_MOESM12_ESM.zip › Table S8a.pdf]

	GO.ID	Term	Annotated	Significant	Expected	fisher p
1	GO:1903749	positive regulation of establishment of protein localization to mitochondrion	27	2	0,04	0,00072
2	GO:1903747	regulation of establishment of protein localization to mitochondrion	33	2	0,05	0,00108
3	GO:0010822	positive regulation of mitochondrion organization	56	2	0,09	0,0031
4	GO:0070585	protein localization to mitochondrion	57	2	0,09	0,00321
5	GO:0072655	establishment of protein localization to mitochondrion	57	2	0,09	0,00321
6	GO:0010821	regulation of mitochondrion organization	83	2	0,13	0,00671
7	GO:0051683	establishment of Golgi localization	5	1	0,01	0,00763
8	GO:0060074	synapse maturation	5	1	0,01	0,00763
9	GO:1903311	regulation of mRNA metabolic process	97	2	0,15	0,00907
10	GO:0051151	negative regulation of smooth muscle cell differentiation	6	1	0,01	0,00915
11	GO:1903044	protein localization to membrane raft	6	1	0,01	0,00915
12	GO:0016071	mRNA metabolic process	318	3	0,49	0,0106
13	GO:0021602	cranial nerve morphogenesis	7	1	0,01	0,01066
14	GO:0048308	organelle inheritance	7	1	0,01	0,01066
15	GO:0048313	Golgi inheritance	7	1	0,01	0,01066
16	GO:0051645	Golgi localization	7	1	0,01	0,01066
17	GO:0060216	definitive hemopoiesis	7	1	0,01	0,01066
18	GO:0080009	mRNA methylation	7	1	0,01	0,01066
19	GO:1900363	regulation of mRNA polyadenylation	7	1	0,01	0,01066
20	GO:0006839	mitochondrial transport	106	2	0,16	0,01076
21	GO:0016556	mRNA modification	8	1	0,01	0,01218
22	GO:0060341	regulation of cellular localization	343	3	0,52	0,01303
23	GO:0021545	cranial nerve development	9	1	0,01	0,01369
24	GO:0030878	thyroid gland development	9	1	0,01	0,01369
25	GO:0010468	regulation of gene expression	1528	6	2,34	0,01404
26	GO:0090150	establishment of protein localization to membrane	125	2	0,19	0,01476
27	GO:0071786	endoplasmic reticulum tubular network organization	10	1	0,02	0,0152
28	GO:0006886	intracellular protein transport	364	3	0,56	0,01532
29	GO:1903829	positive regulation of cellular protein localization	132	2	0,2	0,01637
30	GO:0031440	regulation of mRNA 3'-end processing	11	1	0,02	0,01671

31	GO:0032594	protein transport within lipid bilayer	11	1	0,02	0,01671
32	GO:0051150	regulation of smooth muscle cell differentiation	11	1	0,02	0,01671
33	GO:0006402	mRNA catabolic process	138	2	0,21	0,01781
34	GO:0016070	RNA metabolic process	1632	6	2,5	0,0195
35	GO:0000291	nuclear-transcribed mRNA catabolic process, exonucleolytic	13	1	0,02	0,01972
36	GO:0001844	protein insertion into mitochondrial membrane involved in apoptotic signaling pathway	13	1	0,02	0,01972
37	GO:0043928	exonucleolytic catabolism of deadenylated mRNA	13	1	0,02	0,01972
38	GO:0050913	sensory perception of bitter taste	13	1	0,02	0,01972
39	GO:0051145	smooth muscle cell differentiation	13	1	0,02	0,01972
40	GO:0051204	protein insertion into mitochondrial membrane	13	1	0,02	0,01972
41	GO:1900739	regulation of protein insertion into mitochondrial membrane involved in apoptotic signaling pathway	13	1	0,02	0,01972
42	GO:1900740	positive regulation of protein insertion into mitochondrial membrane involved in apoptotic signaling pathway	13	1	0,02	0,01972
43	GO:0006401	RNA catabolic process	151	2	0,23	0,02112
44	GO:0008038	neuron recognition	14	1	0,02	0,02122
45	GO:1902893	regulation of pri-miRNA transcription by RNA polymerase II	14	1	0,02	0,02122
46	GO:1903955	positive regulation of protein targeting to mitochondrion	14	1	0,02	0,02122
47	GO:0006378	mRNA polyadenylation	15	1	0,02	0,02272
48	GO:0043631	RNA polyadenylation	15	1	0,02	0,02272
49	GO:0032386	regulation of intracellular transport	159	2	0,24	0,02327
50	GO:0061614	pri-miRNA transcription by RNA polymerase II	16	1	0,02	0,02422

BMI_only

	GO.ID	Term	Annotated	Significant	Expected	fisher p
1	GO:0034641	cellular nitrogen compound metabolic process	2443	58	40,73	0,0005
2	GO:0043921	modulation by host of viral transcription	11	3	0,18	0,00067
3	GO:0052312	modulation of transcription in other organism involved in symbiotic interaction	11	3	0,18	0,00067
4	GO:0052472	modulation by host of symbiont transcription	11	3	0,18	0,00067
5	GO:0006139	nucleobase-containing compound metabolic process	2177	52	36,29	0,00116
6	GO:0010972	negative regulation of G2/M transition of mitotic cell cycle	30	4	0,5	0,00143
7	GO:0071347	cellular response to interleukin-1	51	5	0,85	0,0015
8	GO:0010604	positive regulation of macromolecule metabolic process	1099	31	18,32	0,00155
9	GO:0022618	ribonucleoprotein complex assembly	77	6	1,28	0,0017
10	GO:0046626	regulation of insulin receptor signaling pathway	15	3	0,25	0,00177
11	GO:0009308	amine metabolic process	53	5	0,88	0,00178
12	GO:0060218	hematopoietic stem cell differentiation	33	4	0,55	0,00206
13	GO:0048863	stem cell differentiation	82	6	1,37	0,00235
14	GO:0010467	gene expression	1913	46	31,89	0,0025
15	GO:0006325	chromatin organization	246	11	4,1	0,00252
16	GO:0070498	interleukin-1-mediated signaling pathway	35	4	0,58	0,00257
17	GO:1902750	negative regulation of cell cycle G2/M phase transition	35	4	0,58	0,00257
18	GO:0046483	heterocycle metabolic process	2249	52	37,49	0,00261
19	GO:0016579	protein deubiquitination	84	6	1,4	0,00265
20	GO:0034310	primary alcohol catabolic process	5	2	0,08	0,00267
21	GO:2000341	regulation of chemokine (C-X-C motif) ligand 2 production	5	2	0,08	0,00267
22	GO:0071826	ribonucleoprotein complex subunit organization	86	6	1,43	0,00298
23	GO:0070555	response to interleukin-1	61	5	1,02	0,00333
24	GO:0090304	nucleic acid metabolic process	1885	45	31,43	0,00336
25	GO:0006725	cellular aromatic compound metabolic process	2274	52	37,91	0,0034
26	GO:1900076	regulation of cellular response to insulin stimulus	19	3	0,32	0,0036
27	GO:0009967	positive regulation of signal transduction	593	19	9,89	0,00391
28	GO:0001732	formation of cytoplasmic translation initiation complex	6	2	0,1	0,00395
29	GO:0010692	regulation of alkaline phosphatase activity	6	2	0,1	0,00395
30	GO:0010888	negative regulation of lipid storage	6	2	0,1	0,00395
31	GO:0072567	chemokine (C-X-C motif) ligand 2 production	6	2	0,1	0,00395
32	GO:1901033	positive regulation of response to reactive oxygen species	6	2	0,1	0,00395

33	GO:0070646	protein modification by small protein removal	91	6	1,52	0,00396
34	GO:0006521	regulation of cellular amino acid metabolic process	20	3	0,33	0,00418
35	GO:0038061	NIK/NF-kappaB signaling	65	5	1,08	0,00439
36	GO:0010647	positive regulation of cell communication	646	20	10,77	0,00452
37	GO:0023056	positive regulation of signaling	647	20	10,79	0,0046
38	GO:0002244	hematopoietic progenitor cell differentiation	66	5	1,1	0,00468
39	GO:1901360	organic cyclic compound metabolic process	2365	53	39,43	0,00483
40	GO:0009891	positive regulation of biosynthetic process	611	19	10,19	0,00542
41	GO:1900409	positive regulation of cellular response to oxidative stress	7	2	0,12	0,00548
42	GO:1902680	positive regulation of RNA biosynthetic process	479	16	7,99	0,00553
43	GO:1903508	positive regulation of nucleic acid-templated transcription	479	16	7,99	0,00553
44	GO:0010557	positive regulation of macromolecule biosynthetic process	569	18	9,49	0,00572
45	GO:0051173	positive regulation of nitrogen compound metabolic process	1044	28	17,41	0,0058
46	GO:0031325	positive regulation of cellular metabolic process	1095	29	18,26	0,00589
47	GO:0050731	positive regulation of peptidyl-tyrosine phosphorylation	70	5	1,17	0,00602
48	GO:0050807	regulation of synapse organization	70	5	1,17	0,00602
49	GO:0048584	positive regulation of response to stimulus	852	24	14,2	0,0061
50	GO:0051851	modulation by host of symbiont process	23	3	0,38	0,00626
51	GO:0002474	antigen processing and presentation of peptide antigen via MHC class I	45	4	0,75	0,00644
52	GO:0009893	positive regulation of metabolic process	1204	31	20,07	0,00653
53	GO:0000165	MAPK cascade	320	12	5,33	0,00672
54	GO:0050803	regulation of synapse structure or activity	72	5	1,2	0,00678
55	GO:0023014	signal transduction by protein phosphorylation	321	12	5,35	0,00689
56	GO:0090263	positive regulation of canonical Wnt signaling pathway	46	4	0,77	0,00697
57	GO:0043923	positive regulation by host of viral transcription	8	2	0,13	0,00722
58	GO:0071108	protein K48-linked deubiquitination	8	2	0,13	0,00722
59	GO:0071526	semaphorin-plexin signaling pathway	8	2	0,13	0,00722
60	GO:1902884	positive regulation of response to oxidative stress	8	2	0,13	0,00722
61	GO:0031146	SCF-dependent proteasomal ubiquitin-dependent protein catabolic process	25	3	0,42	0,00794
62	GO:0061418	regulation of transcription from RNA polymerase II promoter in response to hypoxia	25	3	0,42	0,00794
63	GO:0045944	positive regulation of transcription by RNA polymerase II	328	12	5,47	0,00813

64	GO:0071236	cellular response to antibiotic	49	4	0,82	0,00871
65	GO:0045935	positive regulation of nucleobase-containing compound metabolic process	593	18	9,89	0,00871
66	GO:0038034	signal transduction in absence of ligand	26	3	0,43	0,00887
67	GO:0051702	interaction with symbiont	26	3	0,43	0,00887
68	GO:0097192	extrinsic apoptotic signaling pathway in absence of ligand	26	3	0,43	0,00887
69	GO:1902036	regulation of hematopoietic stem cell differentiation	26	3	0,43	0,00887
70	GO:0010629	negative regulation of gene expression	596	18	9,94	0,00916
71	GO:0002183	cytoplasmic translational initiation	9	2	0,15	0,00919
72	GO:0006067	ethanol metabolic process	9	2	0,15	0,00919
73	GO:1901889	negative regulation of cell junction assembly	9	2	0,15	0,00919
74	GO:0014065	phosphatidylinositol 3-kinase signaling	50	4	0,83	0,00934
75	GO:0031328	positive regulation of cellular biosynthetic process	599	18	9,99	0,00963
76	GO:0051254	positive regulation of RNA metabolic process	509	16	8,49	0,00976
77	GO:0032270	positive regulation of cellular protein metabolic process	563	17	9,39	0,01134
78	GO:0008210	estrogen metabolic process	10	2	0,17	0,01136
79	GO:0043984	histone H4-K16 acetylation	10	2	0,17	0,01136
80	GO:0051923	sulfation	10	2	0,17	0,01136
81	GO:0034612	response to tumor necrosis factor	114	6	1,9	0,01165
82	GO:0014068	positive regulation of phosphatidylinositol 3-kinase signaling	29	3	0,48	0,01202
83	GO:0031145	anaphase-promoting complex-dependent catabolic process	29	3	0,48	0,01202
84	GO:0046777	protein autophosphorylation	83	5	1,38	0,01218
85	GO:0043170	macromolecule metabolic process	3418	69	56,98	0,01256
86	GO:0016070	RNA metabolic process	1632	38	27,21	0,01275
87	GO:0033238	regulation of cellular amine metabolic process	30	3	0,5	0,0132
88	GO:0006807	nitrogen compound metabolic process	3798	75	63,32	0,01336
89	GO:0048522	positive regulation of cellular process	1855	42	30,93	0,01338
90	GO:0090049	regulation of cell migration involved in sprouting angiogenesis	11	2	0,18	0,01373
91	GO:0034113	heterotypic cell-cell adhesion	31	3	0,52	0,01444
92	GO:0046782	regulation of viral transcription	31	3	0,52	0,01444
93	GO:0030177	positive regulation of Wnt signaling pathway	57	4	0,95	0,01467
94	GO:0048015	phosphatidylinositol-mediated signaling	57	4	0,95	0,01467
95	GO:0050730	regulation of peptidyl-tyrosine phosphorylation	87	5	1,45	0,01472
96	GO:0045893	positive regulation of transcription, DNA-templated	444	14	7,4	0,01511

97	GO:0042073	intraciliary transport	32	3	0,53	0,01574
98	GO:0016486	peptide hormone processing	12	2	0,2	0,0163
99	GO:0032800	receptor biosynthetic process	12	2	0,2	0,0163
100	GO:0043552	positive regulation of phosphatidylinositol 3-kinase activity	12	2	0,2	0,0163

	GO.ID	Term	Annotated	Significant	Expected	fisher p
1	GO:0030225	macrophage differentiation	18	6	1,13	0,00058
2	GO:0046330	positive regulation of JNK cascade	58	11	3,65	0,00084
3	GO:0009226	nucleotide-sugar biosynthetic process	8	4	0,5	0,00089
4	GO:0043507	positive regulation of JUN kinase activity	35	8	2,21	0,00121
5	GO:0070266	necroptotic process	21	6	1,32	0,00145
6	GO:0097300	programmed necrotic cell death	22	6	1,39	0,00189
7	GO:2001040	positive regulation of cellular response to drug	5	3	0,32	0,00226
8	GO:0032874	positive regulation of stress-activated MAPK cascade	67	11	4,22	0,00283
9	GO:0070304	positive regulation of stress-activated protein kinase signaling cascade	68	11	4,29	0,00319
10	GO:0070265	necrotic cell death	25	6	1,58	0,00383
11	GO:0043506	regulation of JUN kinase activity	42	8	2,65	0,00411
12	GO:0002763	positive regulation of myeloid leukocyte differentiation	18	5	1,13	0,00419
13	GO:0030889	negative regulation of B cell proliferation	6	3	0,38	0,00431
14	GO:0050858	negative regulation of antigen receptor-mediated signaling pathway	12	4	0,76	0,00513
15	GO:0002886	regulation of myeloid leukocyte mediated immunity	27	6	1,7	0,00575
16	GO:0033866	nucleoside bisphosphate biosynthetic process	27	6	1,7	0,00575
17	GO:0034030	ribonucleoside bisphosphate biosynthetic process	27	6	1,7	0,00575
18	GO:0034033	purine nucleoside bisphosphate biosynthetic process	27	6	1,7	0,00575
19	GO:0002578	negative regulation of antigen processing and presentation	7	3	0,44	0,00718
20	GO:0007256	activation of JNKK activity	7	3	0,44	0,00718
21	GO:0032680	regulation of tumor necrosis factor production	56	9	3,53	0,00771
22	GO:0007254	JNK cascade	87	12	5,48	0,00796
23	GO:0032271	regulation of protein polymerization	77	11	4,85	0,00837
24	GO:0035384	thioester biosynthetic process	21	5	1,32	0,00851
25	GO:0071616	acyl-CoA biosynthetic process	21	5	1,32	0,00851
26	GO:0002761	regulation of myeloid leukocyte differentiation	38	7	2,39	0,00862
27	GO:0051291	protein heterooligomerization	38	7	2,39	0,00862
28	GO:0032640	tumor necrosis factor production	58	9	3,65	0,0097
29	GO:1903555	regulation of tumor necrosis factor superfamily cytokine production	58	9	3,65	0,0097
30	GO:0045892	negative regulation of transcription, DNA-templated	332	32	20,92	0,00993

31	GO:0046328	regulation of JNK cascade	79	11	4,98	0,01012
32	GO:0007257	activation of JUN kinase activity	22	5	1,39	0,01045
33	GO:0002573	myeloid leukocyte differentiation	69	10	4,35	0,01059
34	GO:0016556	mRNA modification	8	3	0,5	0,01096
35	GO:0032727	positive regulation of interferon-alpha production	8	3	0,5	0,01096
36	GO:0060219	camera-type eye photoreceptor cell differentiation	8	3	0,5	0,01096
37	GO:0002478	antigen processing and presentation of exogenous peptide antigen	80	11	5,04	0,01109
38	GO:0033108	mitochondrial respiratory chain complex assembly	50	8	3,15	0,01205
39	GO:0071706	tumor necrosis factor superfamily cytokine production	60	9	3,78	0,01206
40	GO:0019884	antigen processing and presentation of exogenous antigen	81	11	5,1	0,01213
41	GO:0050670	regulation of lymphocyte proliferation	81	11	5,1	0,01213
42	GO:0009225	nucleotide-sugar metabolic process	15	4	0,95	0,01216
43	GO:2001025	positive regulation of response to drug	15	4	0,95	0,01216
44	GO:0006468	protein phosphorylation	676	57	42,6	0,01222
45	GO:0031098	stress-activated protein kinase signaling cascade	115	14	7,25	0,01285
46	GO:0001932	regulation of protein phosphorylation	472	42	29,74	0,01313
47	GO:0032944	regulation of mononuclear cell proliferation	82	11	5,17	0,01324
48	GO:0060341	regulation of cellular localization	343	32	21,61	0,01552
49	GO:1902105	regulation of leukocyte differentiation	95	12	5,99	0,01561
50	GO:0019730	antimicrobial humoral response	33	6	2,08	0,01564
