# Supplementary material for: The transcriptome-wide association search for genes and genetic variants which associate with BMI and gestational weight gain in women with type 1 diabetes
Source: Mol Med. 2021 Jan 20;27:6. doi: 10.1186/s10020-020-00266-z (PMC7818927; doi:10.1186/s10020-020-00266-z)
Supplement: Supplementary file 14 — Additional file 14: Table S9a. Go Enrichment analysis on the overlap between BMI and GWG genes in T2D and ARIC cohorts.b. Go Enrichment analysis on BMI only associated genes in T2D and ARIC cohorts. c. Go Enrichment analysis on GWG only associated genes in T2D and ARIC cohorts. [file 10020_2020_266_MOESM14_ESM.zip › Table S9b.pdf]

BMI_GWG_overlap

	GO.ID	Term	Annotated	Significant	Expected	fisher p
1	GO:0006509	membrane protein ectodomain proteolysis	5	3	0,3	0,002
2	GO:0009066	aspartate family amino acid metabolic process	6	3	0,36	0,0038
3	GO:0001776	leukocyte homeostasis	7	3	0,42	0,0063
4	GO:0033619	membrane protein proteolysis	7	3	0,42	0,0063
5	GO:0019751	polyol metabolic process	14	4	0,85	0,0079
6	GO:0046173	polyol biosynthetic process	8	3	0,49	0,0097
7	GO:1901532	regulation of hematopoietic progenitor cell differentiation	9	3	0,55	0,0139
8	GO:1902036	regulation of hematopoietic stem cell differentiatio	9	3	0,55	0,0139
9	GO:0043648	dicarboxylic acid metabolic process	10	3	0,61	0,019
10	GO:0060218	hematopoietic stem cell differentiation	11	3	0,67	0,025
11	GO:2000736	regulation of stem cell differentiation	12	3	0,73	0,0319
12	GO:0002260	lymphocyte homeostasis	5	2	0,3	0,0323
13	GO:0006099	tricarboxylic acid cycle	5	2	0,3	0,0323
14	GO:0031167	rRNA methylation	5	2	0,3	0,0323
15	GO:0035904	aorta development	5	2	0,3	0,0323
16	GO:0061028	establishment of endothelial barrier	5	2	0,3	0,0323
17	GO:0071559	response to transforming growth factor beta	13	3	0,79	0,0397
18	GO:0071560	cellular response to transforming growth factor beta stimulus	13	3	0,79	0,0397
19	GO:0120031	plasma membrane bounded cell projection assembly	55	7	3,34	0,0447
20	GO:0000154	rRNA modification	6	2	0,36	0,0466

	GO.ID	Term	Annotated	Significant	Expected	fisher p
1	GO:0050877	nervous system process	84	74	60,62	0,00028
2	GO:0009628	response to abiotic stimulus	107	91	77,22	0,0009
3	GO:0007600	sensory perception	47	43	33,92	0,00097
4	GO:0003008	system process	133	109	95,98	0,00439
5	GO:0009056	catabolic process	231	183	166,7	0,00507
6	GO:0007265	Ras protein signal transduction	40	36	28,87	0,00538
7	GO:0006511	ubiquitin-dependent protein catabolic process	50	44	36,08	0,00569
8	GO:0019941	modification-dependent protein catabolic process	50	44	36,08	0,00569
9	GO:0043632	modification-dependent macromolecule catabolic process	50	44	36,08	0,00569
10	GO:1901575	organic substance catabolic process	185	148	133,5	0,00605
11	GO:0070482	response to oxygen levels	39	35	28,14	0,00681
12	GO:0009266	response to temperature stimulus	22	21	15,88	0,00695
13	GO:0009408	response to heat	15	15	10,82	0,0073
14	GO:0050954	sensory perception of mechanical stimulus	15	15	10,82	0,0073
15	GO:0042176	regulation of protein catabolic process	38	34	27,42	0,0086
16	GO:0051606	detection of stimulus	27	25	19,48	0,00905
17	GO:1903362	regulation of cellular protein catabolic process	27	25	19,48	0,00905
18	GO:0001666	response to hypoxia	37	33	26,7	0,01083
19	GO:0036293	response to decreased oxygen levels	37	33	26,7	0,01083
20	GO:0070997	neuron death	26	24	18,76	0,01176

GWG_only

	GO.ID	Term	Annotated	Significant	Expected	fisher p
1	GO:0060341	regulation of cellular localization	77	31	16,76	0,00013
2	GO:0007005	mitochondrion organization	44	18	9,58	0,00287
3	GO:0051291	protein heterooligomerization	11	7	2,39	0,00318
4	GO:1903827	regulation of cellular protein localization	49	19	10,67	0,00454
5	GO:0070266	necroptotic process	9	6	1,96	0,0047
6	GO:0097300	programmed necrotic cell death	9	6	1,96	0,0047
7	GO:0016525	negative regulation of angiogenesis	7	5	1,52	0,00676
8	GO:2000181	negative regulation of blood vessel morphogenesis	7	5	1,52	0,00676
9	GO:0009225	nucleotide-sugar metabolic process	5	4	1,09	0,00916
10	GO:0009226	nucleotide-sugar biosynthetic process	5	4	1,09	0,00916
11	GO:0033280	response to vitamin D	5	4	1,09	0,00916
12	GO:0032880	regulation of protein localization	85	28	18,5	0,00949
13	GO:0002495	antigen processing and presentation of peptide antigen via MHC class II	10	6	2,18	0,00962
14	GO:0002504	antigen processing and presentation of peptide or polysaccharide antigen via MHC class II	10	6	2,18	0,00962
15	GO:0019886	antigen processing and presentation of exogenous peptide antigen via MHC class II	10	6	2,18	0,00962
16	GO:0033866	nucleoside bisphosphate biosynthetic process	10	6	2,18	0,00962
17	GO:0034030	ribonucleoside bisphosphate biosynthetic process	10	6	2,18	0,00962
18	GO:0034033	purine nucleoside bisphosphate biosynthetic process	10	6	2,18	0,00962
19	GO:0033865	nucleoside bisphosphate metabolic process	13	7	2,83	0,01101
20	GO:0033875	ribonucleoside bisphosphate metabolic process	13	7	2,83	0,01101
